# Post-Transcriptional Gene Regulation by MicroRNAs During Barley Malting

**DOI:** 10.3390/genes17060676

**Published:** 2026-06-09

**Authors:** Sarah J. Whitcomb, Marcus A. Vinje, Ramamurthy Mahalingam

**Affiliations:** United States Department of Agriculture—Agricultural Research Service (USDA—ARS), 502 Walnut Street, Madison, WI 53726, USA; sarah.whitcomb@usda.gov (S.J.W.); marcus.vinje@usda.gov (M.A.V.)

**Keywords:** barley, microRNAs, malting, degradome, phytohormones

## Abstract

Background/Objectives: Barley malting is an agro-industrial process that produces malt, an essential ingredient for the brewing and distilling industries. Previously, tran-scriptome profiling has revealed mRNA changes during malting but less is known about their regulation. Methods: The spring 2-row barley variety ‘Conrad’ was sampled at five stages of malt-ing. Using small RNA (sRNA)-sequencing and degradome-sequencing data from these malting stages, *de novo* discovery of mature microRNA (miRNA), as well as cognate mRNAs targeted for slicing, was identified. ShortStack v4.1.0 was used to map sRNA reads to the *Hordeum vulgare* Morex V3 genome. Results: In total, 33 expressed MIRs were identified, six of which may be novel. Using the degradome-sequencing data from the same malting stages, CleaveLand4 v4.5 pre-dicted 64 sliced mRNA targets, predominantly transcription factors associated with root development. Conclusions: This study provides an overview of post-transcriptional modulations of miRNAs-cognate mRNA targets, as well as plausible interactions between miRNAs during barley malting.

## 1. Background

Post-transcriptional regulation of gene expression refers to the control of gene expression that occurs after RNA has been transcribed from DNA but before it is translated into protein. It encompasses multiple aspects of RNA metabolism, including splicing, export, stability and decay, and is central for various cellular processes in all organisms [1]. Currently, two major post-transcriptional gene regulatory mechanisms are known, namely RNA-binding proteins [2] and RNA interference, also known as post-transcriptional gene silencing [3]. Two types of RNA interference have been reported in the literature, microRNAs (miRNAs) and small interfering RNAs (siRNAs) [4].

In plants, miRNA genes (MIR) are transcribed by RNA polymerase II, and the resulting primary miRNA (pri-miRNA) transcripts form a hairpin-like structure, which is processed by the enzyme Dicer-like 1 (DCL1) [5]. A double-stranded RNA binding protein (HYL1, Hyponastic Leaves 1), a C2H2 zinc-finger protein (SERRATE), and a conserved forked domain containing protein (DDL, Dawdle) help DCL1 trim the pri-miRNA into the precursor miRNA (pre-miRNA) hairpin and to process the pre-miRNA into the mature miRNA duplex (miRNA and miRNA*). This duplex is stabilized by the addition of a methyl group to the 3′ ends of the miRNA duplex by a methyltransferase enzyme (HUA Enhancer 1, HEN1). The methylated miRNA duplex is then exported to the cytoplasm by a plant ortholog of exportin 5 (HASTY5), where one strand of the duplex is loaded into the RNA-induced Silencing Complex (RISC) containing Argonaute protein. The complementarity between miRNA and its cognate messenger RNA (mRNA) target has been considered a major factor determining the silencing outcome [3]. Later studies have established that a distinctive RNA secondary structure abutting the miRNA binding site plays a crucial role in determining the targeting efficiency [6]. When the central miRNA region is not completely complementary to the target mRNA, it leads to translational repression rather than mRNA slicing [7]. Two major mechanisms of action for miRNAs have been revealed so far. Some miRNAs refine or restrict the expression patterns of target mRNAs or proteins such that the targets are only expressed in cells where the miRNAs are absent. Other miRNAs are co-expressed with their targets and dampen their expression [5]. In plants, most validated targets of miRNAs code for transcription factors (TFs) belonging to families with crucial developmental functions, including the control of root and shoot architecture, vegetative to reproductive phase transitions, and leaf and flower morphogenesis [8].

Identification of *bona fide* miRNAs amongst a vast small RNA (sRNA) population, mostly composed of siRNAs, is a major challenge [9]. In a comprehensive analysis of sRNAs from 47 plant species, it was reported that in barley, miRNAs constituted about 15% and siRNAs were around 80% of sRNA reads [10]. Furthermore, miRNAs and siRNAs are very similar in length (both classes may be 20–24 nucleotides (nt), so distinguishing these two major sRNA classes relies principally on identifying their genomic origin. An siRNA locus produces several overlapping siRNAs, whereas the pri-miRNA encoded by a MIR gene usually produces two miRNAs (miRNA and miRNA*) from an imperfect RNA hairpin [11]. With the advent of the next-generation sequencing (NGS), there was an enormous surge in sRNA research [12]. Later, it was observed that many sRNAs were erroneously annotated as miRNAs in databases and publications, and stringent criteria were proposed to help classify genuine miRNAs from sRNA NGS libraries [13]. The features of the miRNA/miRNA* duplex that are important for Dicer recognition and consistent processing were clearly defined and included (i) typically a single miRNA/miRNA*duplex, (ii) the region of foldback that gives rise to the miRNA duplex should not contain secondary stems or large internal loops (>5 nt) that interrupt the miRNA/miRNA* duplex, (iii) foldbacks longer than 300 nt should not be annotated as MIR, (iv) expression of one (most typical)—three (max) miRNA/miRNA* duplexes must be observed in small RNA sequencing (sRNA-seq) data, and (v) less than six mismatched positions between miRNA and miRNA*, a maximum of three of which can be asymmetrically bulged. Novel annotations must meet all the above criteria in at least two sRNA-seq libraries (biological replicates). miRNA must be 20–24 nt in length; for 23–24 nt candidates, miRNA should have more evidence: precise miRNA/miRNA* accumulation in four or more libraries and demonstration that they post-transcriptionally regulate target RNAs in a miRNA-like manner (e.g., target cleavage, translational repression). Following these stringent criteria would greatly minimize the number of sRNAs that are classified as miRNAs and ensure the veracity of the miRNA sequences deposited into databases.

Barley is ranked fourth among cereal grains and is gaining popularity in the food and feed industry owing to its high nutritive value, including fiber quality, especially ß-glucan [14], and health-promoting compounds such as the tocochromonols [15]. In the US, most barley produced is used by the malting and brewing industry. The malting process involves three consecutive stages: steeping, germination, and kilning [16]. Steeping involves soaking barley seeds in water (for 24–36 h) to reach a desired moisture content (~45%). At the end of the steep stage, *senso stricto* seed germination is completed [17]. During the germination phase of malting, steeped samples are subjected to growth conditions that bring about the degradation of stored carbohydrates and proteins by the synthesis or activation of a number of enzymes [18]. The end-product of the germination stage is referred to as green malt. During the kilning process, green malt is dried slowly at high temperatures so that all biochemical activities are suspended, and most of the moisture is removed to less than 4%.

Understanding the molecular genetic pathways associated with the malting process is important for aiding sustainable solutions for the malting and brewing industry. Detailed transcriptome and proteome analysis during different malting stages has been previously reported [17,19]. RNA-binding proteins associated with barley malting that could potentially be involved in the post-transcriptional gene regulation were reported [20]. The role of miRNAs during malting is unknown. Barley miRNAs were first identified experimentally in 2011 through NGS and Northern blot hybridization [21]. Since then, more miRNAs have been identified using NGS in multiple barley cultivars under various conditions and summarized in a review [22]. Two very recent studies on abiotic stress in barley identified drought [23] and heat-responsive miRNAs [24], while a third study focused on virus-responsive miRNAs [25]. In this study, we report the expressed MIR and associated miRNAs during the barley malting process. We also validate the in silico predicted target genes of these miRNAs via degradome libraries generated from the same tissue samples used to generate the sRNA libraries.

## 2. Materials and Methods

### 2.1. Micromalting

Samples of Conrad, a North American 2-row spring malting cultivar, were obtained from the Cereal Crops Research Unit’s Malt Quality Laboratory in Madison, WI, USA and originated from two different crop years grown in four different environments (Aberdeen, ID (2014 and 2015); Bozeman, MT (2015); Ray, ND (2014); and Sidney, MT (2015)). Seeds from different geographical locations were pooled together equally (120 g *w*/*w*) and homogenized. Three replicate samples were drawn from the pooled seed sample, and 110 g (dry wt.) of each replicate was micromalted. Micromalting was performed using the CCRU’s Traditional Malting System, which briefly consists of steeping (4 h water immersion (16 °C) followed by 4 h air rest (18 °C) repeated until seed moisture is at 45% (38 h total steep duration)), germinating (120 h (17 °C) while rotating every 3 min/30 min), and kilning (lowers moisture to ~4% using increasingly hotter air). Kilned samples were obtained for malt quality analysis only and not used in this study. Dry samples refer to the unmalted grains. Out of steep samples were considered 0 days of germination (Day0). The remaining samples (Day1, Day3, Day5) were collected 24 h, 72 h, and 120 h after steeping (Day0), respectively. Micromalted samples were collected at the same time every day, flash frozen in liquid nitrogen, and stored at −80 °C.

### 2.2. Samples, RNA Isolation, and Library Preparation

Three micromalting replicates from each time point (Dry, Day0, Day1, Day3, and Day5) were used to prepare Illumina TruSeq Small RNA libraries, and one micromalting replication was used from each time point to prepare the Parallel Analysis of RNA Ends (PARE) degradome libraries.

For the TruSeq Small RNA libraries, sRNA was isolated from ~ 100 mg seeds (ground in liquid nitrogen) using the Concert Plant RNA Reagent (Invitrogen, Carlsbad, CA, USA) and the miRNeasy Mini Kit (Qiagen, Valencia, CA, USA), following the manufacturer’s protocols with the following modifications. Volumes of Concert Plant RNA Reagent, NaCl, and chloroform were 40% more than the manufacturer’s suggestion, owing to high levels of polysaccharides. The RNA was prepared for binding to a RNeasy mini spin column by mixing the aqueous layer with 1.5 volumes of 100% ethanol. Further purification and removal of genomic DNA was accomplished by following the miRNeasy Mini Kit on-column DNase I digestion protocol (Qiagen).

For the PARE-libraries, a large-scale RNA isolation was performed as described above with the following changes: sample amount was ~450 mg, 4.5 mL of Concert Plant RNA reagent was used with 112 mg of polyvinylpyrrolidone (MW 40,000), and followed by two chloroform/ethanol precipitations in lieu of the miRNAeasy kit step.

The Illumina TruSeq Small RNA libraries were prepared by the University of Wisconsin-Madison Biotechnology Center (UWBC) Gene Expression Center following the manufacturer’s preparation guide (https://support.illumina.com/content/dam/illumina-support/documents/documentation/chemistry_documentation/samplepreps_truseq/truseqsmallrna/truseq-small-rna-library-prep-kit-reference-guide-15004197-02.pdf, accessed on 18 May 2026) including gel purification by excision of a gel slice from 145 to 160 bp, corresponding to insert sRNAs approximately 20–35 nt. The sRNA libraries were quantified on a fluorometer, and quality was assessed using Agilent’s (Santa Clara, CA, USA) High Sensitivity DNA Analysis assay following the manufacturer’s protocol at the UWBC Gene Expression Center. PARE-libraries, aka degradomes, were prepared following a previously established protocol [26].

The 15 TruSeq Small RNA libraries were sequenced on the Illumina (San Diego, CA, USA) HiSeq 2500 on one 1 × 100 lane, and the five PARE libraries were sequenced on one 1 × 100 lane at the UWBC Next Generation Sequencing Core using the PARE sequencing primer (5′-CCACCGACAGGTTCA GAGTTCTACAGTCCGAC-3′).

### 2.3. sRNA Read Processing

The quality of raw reads from the 15 TruSeq Small RNA libraries was assessed with FastQC version 0.12.1 (https://www.bioinformatics.babraham.ac.uk/projects/fastqc/, accessed on 18 May 2026) and MultiQC version 1.14 [27]. The percent of bases with a Q score ≥30 was higher than 88 in all libraries, and the mean quality score of reads in each library was >34.

Clean reads were obtained using Atropos version 1.1.31 [28] with the following parameters, “ -q 28 -a TGGAATTCTCGGGTGCCAAGG --overlap 6 --discard-untrimmed -m 1 -se”. Specifically, reads were quality trimmed from the 3′ end with a quality cutoff of 28, and the TruSeq RA3 adapter was trimmed from the 3′ end of reads. Reads without the RA3 adapter and those with a length of zero after trimming were discarded.

Size filtering was then applied to the clean reads with Atropos to retain reads 18–24 nt in length using the following parameters, “-m 18 -M 24 -se”. Final quality checks were performed with FastQC version 0.12.1 (https://www.bioinformatics.babraham.ac.uk/projects/fastqc/, accessed on 18 May 2026), MultiQC version 1.14 [27], and the SeqKit version 2.4.0 stats command [29].

### 2.4. sRNA Alignment and MIR Loci Identification

ShortStack version 4.1.0 [30] was used for genomic alignment of sRNA-seq data and for annotation of MIR with the following settings: --mmap u --dn_mirna --known_miRNAs <known miRNA FASTA> --dicermin 20 --dicermax 24 --strand_cutoff 0.8 --mincov 1 --pad 200 --make_bigwigs. Briefly, clean and size-filtered sRNA-seq reads were aligned to the *Hv* cv Morex V3 reference genome assembly [31] (https://ftp.ebi.ac.uk/ensemblgenomes/pub/release-56/plants/fasta/hordeum_vulgare/cdna/Hordeum_vulgare.MorexV3_pseudomolecules_assembly.cdna.all.fa.gz, accessed on 18 May 2026) with ShortStack in two phases: (1) reads were mapped to the genome using bowtie [32] with settings -v 1 -k 50 -S --best --strata -x. These settings allowed zero or one mismatch and kept only the alignments with zero mismatches if both zero and one-mismatch cases exist. Up to 50 alignments were stored for multi-mapping reads. (2) for multi-mapping reads, a single location for each was decided based upon a local weighting scheme, specified by the --mmap u setting, using only the uniquely mapping alignments in an area.

A custom set of known miRNA sequences (Section 2.5) was used to nucleate searches for genomic regions that met expression- and secondary structure-based requirements for MIR loci. Specifically, genomic intervals where the depth of sRNA coverage, in reads-per-million, is greater than or equal to --mincov 1 were identified, and each of these intervals was then extended in both directions by 200 bp, as given by the setting --pad. Regions that overlapped after extension were merged and the final regions were assigned a cluster ID and correspond to putative MIR loci. These local regions were searched by ShortStack to find miRNA/miRNA*-like patterns of read accumulation: two peaks of read coverage on the same genomic strand. For locations with such a read pattern, the secondary structure of the putative miRNA hairpin precursor was predicted, and in combination with the sRNA-seq alignments, these locations were assessed by stringent criteria for MIR and miRNA calling [13] (Figure S1). Further, to be classified as a MIR, ≥80% of sRNA reads aligned to the cluster must have lengths between the bounds of --dicermin 20 and --dicermax 24.

### 2.5. Known miRNAs

**Figure 1:**
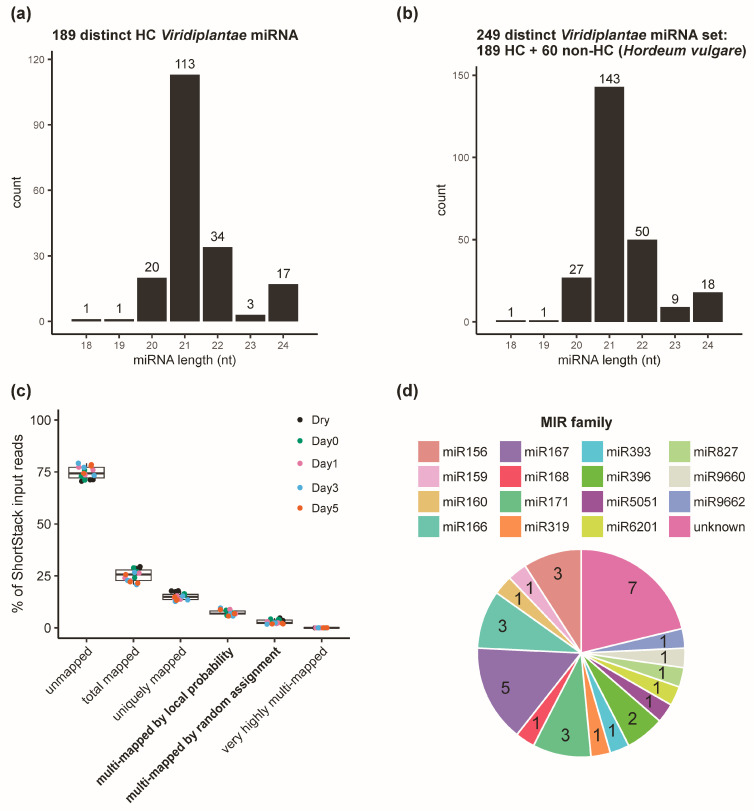
Small RNA (sRNA) alignment to *Hv* cv Morex V3 genome and identification of expressed miRNA loci (MIR) with ShortStack. (**a**) Length distribution of the 189 known distinct high-confidence (HC) mature microRNA (miRNA) from green plant (*Viridiplantae*) species. (**b**) Length distribution of the 249 distinct previously known mature miRNA sequences used in this study to nucleate searches for genomic regions that met expression- and secondary structure-based requirements for MIR loci. A set of 249 consists of 189 HC miRNAs and 60 non-HC *Hv* mature miRNAs. (**c**) ShortStack sRNA reads alignment statistics. Each point represents a library. Point color corresponds to the malting stage. Alignment categories in bold correspond to reads mapped to *de novo* identified MIR loci. (**d**) MIR family assignment of the 33 MIR loci identified in this study. Sector color corresponds to MIR family assignment, and the number of loci identified as expressing mature miRNA is indicated.

A custom set of known mature plant miRNAs was assembled to aid MIR loci identification by ShortStack. The known miRNA set was composed of all green plant (*Viridiplantae*) high-confidence (HC) mature miRNAs in the miRBase [12,33] version 2.1 (https://www.mirbase.org) and all *Hv* mature miRNAs in miRBase. FASTA headers were modified to conform to a simplified format (three-letter species code)-(miRNAname). Sequence duplicates were identified, and the miRNA list was deduplicated with SeqKit version 2.4.0 rmdup command. Deduplication example: osa-miR166a-3p = osa-miR166b-3p = osa-miR166c-3p = osa-miR166d-3p = zma-miR166a-3p = vvi-miR166d = atr-miR166c = atr-miR166d. The sequences of these eight miRNAs are identical in miRBase. Those eight identical sequences were deduplicated to be listed only once in the FASTA file and manually given a new miRNA ID: osa-miR166abcd-3p_zma-miR166a-3p_vvi-miR166d_atr-miR166cd. There are 66 mature miRNAs from *Hv* (miRBase v22.1). Six of those 66 *Hv* miRNAs have an identical sequence to a HC *Viridiplantae* miRNA and were added to the deduplicated *Viridiplantae* HC miRNA list, resulting in 189 distinct sequences. The remaining 60 non-HC *Hv* mature miRNAs were added to the 189 HC distinct miRNAs to give the final set of 249 distinct previously known miRNA sequences for this study (Figure 1b and Figure S2).

### 2.6. Mature miRNA Identification and miRNA Family Assignment

To identify mature miRNA sequences corresponding to the peaks of sRNA alignments in identified MIRs, the ShortStack Results.gff3 file was loaded in the genome browser JBrowse2 version 2.16.1 [34], and the sequences of -5p and -3p MIR sub-features were copied to a separate FASTA file (Figure S3). 

Mature miRNA originating from MIR loci corresponding to known miRNAs were assigned to the miRNA family of the respective known miRNA. For those MIR with no known miRNAs aligned, three miRNA databases were queried with the ShortStack determined miRNA-5p and miRNA-3p MIR sub-feature sequences to determine whether the miRNAs could be novel: (1) miRBase v22.1 (https://mirbase.org/rnacentral_search/, accessed on 18 May 2026) with nhmmer alignment, (2) Plant miRNA Encyclopedia (PmiREN) v2.0 (https://ngdc.cncb.ac.cn/databasecommons/database/id/7132, accessed on 18 May 2026) with BLASTN using the mature miRNA database for all species, (3) plant sRNA gene server (https://plantsmallrnagenes.science.psu.edu/search.php, accessed on 18 May 2026) with BLASTN using small RNA loci database for all genomes. If the miRNA-5p and/or miRNA-3p sub-feature sequences were found in at least one database, then they were assigned the corresponding miRNA family. If not, they were assigned as “unknown” miRNA family (Table S1).

### 2.7. miRNA Expression

The aligned read counts to each sRNA cluster ID from ShortStack Counts.txt were normalized by median-of-ratios (Table S2). Median-of-ratios normalization was used because it considers differences in sequencing depth and sRNA composition among libraries. For each library, the sum of the normalized counts (nCts) aligned to loci in the same miRNA family was determined. To identify differentially expressed miRNA families across the five malting stages, the mean nCts from the three replicates for each stage was calculated. To aid visualization of the subtle differences in expression of these miRNAs, Log10 nCts and z-score nCts were computed and were used for generating the heatmaps.

### 2.8. Degradome Library Read Processing

The quality of raw reads from the five PARE libraries was assessed with FastQC version 0.12.1 (https://www.bioinformatics.babraham.ac.uk/projects/fastqc/, accessed on 18 May 2026) and MultiQC version 1.14 [27]. The percent of bases with a Q score ≥30 was higher than 90 in all libraries, and the mean quality score of reads in each library was >35. 

Clean degradome reads were obtained from raw reads with Atropos version 1.1.31 [28] using the following parameters, “ -q 28 -a TGGAATTCTCGGGTGCCAAGG --overlap 6 --discard-untrimmed -m 1 -se”. Specifically, raw reads were quality trimmed from the 3′ end with a quality cutoff of 28, the TruSeq RA3 adapter was trimmed from the 3′ end of reads. Reads without the RA3 adapter and/or with a length of 0 after trimming were discarded. Clean reads were further size-filtered with Atropos to retain only those reads 19–21 nt in length using the following parameters, “-m 19 -M 21 -se”. Final quality checks on clean and size-filtered reads were performed with FastQC version 0.12.1 (https://www.bioinformatics.babraham.ac.uk/projects/fastqc/, accessed on 18 May 2026) and MultiQC version 1.14 [27]. 

### 2.9. Prediction of miRNA-Directed Slice Sites in Degradome Data

CleaveLand4 version 4.5 (https://github.com/MikeAxtell/CleaveLand4, accessed on 18 May 2026) was used to predict miRNA-5p and miRNA-3p directed slice sites in the clean and size-filtered degradome data. CleaveLand4 mode 1 analysis was performed with the following three steps: (1) degradome reads were aligned with Bowtie v1.3.1 [32] to the forward strand of the Morex V3 reference transcriptome [31] (https://ftp.ebi.ac.uk/ensemblgenomes/pub/release-56/plants/fasta/hordeum_vulgare/cdna/Hordeum_vulgare.MorexV3_pseudomolecules_assembly.cdna.all.fa.gz, accessed on 18 May 2026), allowing a maximum of one mismatch, and in the case of multiple valid alignments, one was randomly selected and reported. The specific command was “bowtie -f -v 1 --best -k 1 --norc -S”. The degradome alignments were parsed to quantify the density of observed 5′ ends at each nt of the transcriptome. (2) The expressed mature miRNA-5p and miRNA-3p sequences (Figure S3) were aligned to the reverse complement strand of the Morex V3 reference transcriptome with Generic Small RNA Transcriptome Aligner GSTAr v 1.0 (https://github.com/MikeAxtell/GSTAr, accessed on 18 May 2026) to identify potential target sites (Figure S4). GSTAr uses thermodynamic predictions rather than sequence similarity to identify miRNA: transcript alignments. Alignments for each miRNA were ranked based on their MFE ratio (minimum free energy of the alignment/minimum free energy of a perfectly matched site). (3) CleaveLand4 looks for evidence of slicing at position 10 relative to the aligned small RNA. The degradome density results and ranked miRNA: transcript alignments were cross-referenced to assign *p*-values to predicted slicing sites, and only those with *p*-value < 0.05 were retained. Those degradome peaks assigned to category 0 or 1 by CleaveLand4 (>1 read, equal to the maximum on the transcript) can be found in Table S3, and T-plots for all category 0 and category 1 sites in each stage can be found in Figure S5. For subsequent analysis, only the category 0 sites were considered predicted miRNA-directed slice sites.

Predicted functional annotations for miRNA-targeted transcripts were pulled from the Morex V3 gene models in BARLEX [35] and the Morex BPGv2 gene models in panBARLEX [36] databases. Morex transcript ID conversions between V3 and BPGv2 were accomplished via a lookup table (https://panbarlex.ipk-gatersleben.de/downloads/liftover_morex_v3_to_BPGv2_gene_ids_only_on_chromosomes.tsv, accessed on 18 May 2026). Expression of predicted target transcripts across the five malting stages was assessed using previously published transcriptome data [17] (Figure S6, Table S4). The normalized read count data used in this publication are publicly available at NCBI’s Gene Expression Omnibus [37] through GEO Series accession number GSE 295574 (https://www.ncbi.nlm.nih.gov/geo/query/acc.cgi?acc=GSE295574, accessed on 18 May 2026).

## 3. Results and Discussion

### 3.1. Overview of sRNA Libraries from Different Barley Malting Stages

As a first step towards the identification of miRNAs present during barley malting, we prepared and sequenced sRNA libraries from dry seeds and four different malting stages (Table 1). Most genuine plant miRNAs are between 20 and 22 nucleotides in length [13], and the size distribution of the 189 distinct high-confidence *Viridiplantae* miRNAs in miRBase v22.1 also reflects this dominant size range for miRNAs (Figure 1a). Adapter removal and low-quality base trimming resulted in clean reads with a mean length of 30–35 nt, suggesting that true miRNA reads represent the minority of reads in the libraries, which is expected from previous reports. In developing barley caryopsis, 21 nt sRNAs constituted about 7% of the small RNA libraries [38]. In germinating barley grains (1 and 5 days), 16–18% of the sRNA library reads were reported to be 21 nucleotides [39]. To enrich for miRNAs in the 15 libraries, clean reads were size-filtered to exclude reads <18 nt or >24 nt, resulting in retention of 9–22% of clean reads. Among clean and size-filtered reads, approximately 90% of reads were identical to others in the same library (duplicates), an indication of high enrichment for miRNAs.

### 3.2. miRNAs Associated with Barley Malting

Although critical for distinguishing miRNAs from other classes of sRNAs, alignment of sRNA-seq data to a reference genome is tricky due to the prevalence of multi-mapping (MMAP) reads in sRNA-seq data. MMAP reads predominate in sRNA-seq data due to the fact that siRNAs are often encoded by repetitive genomic regions [40] and identical miRNAs are often encoded by multiple paralogous loci [41]. The ShortStack program uses the local genomic context of aligned reads to guide decisions on the proper placement of MMAP sRNA-seq reads [30]. It has been widely used in sRNA library analysis in multiple plant species [42,43,44,45,46]. About 13–18% of the cleaned and size-filtered (18–24 nt) reads were mapped to a unique locus in the first phase of read assignment, and about 8–12% were mapped to multiple loci. Using local probability calculations, 58–84% of MMAP reads were finally assigned to a single locus, while 16–42% were finally randomly assigned to a single locus by ShortStack (Figure 1c). Nearly 70–80% of the cleaned and size-filtered reads did not cluster with sufficient density and so remained unmapped to a sRNA locus (Table 2).

In total, 6393 sRNA loci were identified. Among these, 38 loci met all the ShortStack MIR loci criteria [30]. Thirty-three MIR loci were identified when imposing the criterion that more than 80% of the reads assigned to the locus must be 21–24 nt in length, which corresponds to the lengths of sRNA generated by the Dicer enzyme [47] (Table S1). Each MIR produces two major RNAs, a miRNA-5p and a miRNA-3p, one from each arm of the predicted hairpin precursor RNA. Among those miRNA sequences, 67% are 21 nt, 24% are 23 nt, and the remaining 9% are 20 nt in length (Figure S3), consistent with previous reports on plant miRNAs [13].

When compared with two other studies on barley seed germination [39,48] the number of novel miRNAs identified is much lower. However, in previous studies, the novel miRNAs were annotated based only on the predicted hairpin structures of their inferred precursors. By using ShortStack for sRNA alignment and *de novo* MIR loci identification, the current study additionally incorporated characterization of identified MIR by testing for the repeating arrangement of aligned sRNAs (“phasing”, which is not characteristic of true MIR), for sRNA size composition, strandedness, and repetitiveness [49]. Furthermore, in ShortStack4, used here, the unique weighting scheme for MMAP reads gives the highest precision and was used in our analysis [30]. For each of the 33 loci, the coverage and density of aligned sRNA reads were mapped onto the predicted secondary structure of the inferred miRNA-hairpin precursor to allow inspection of each predicted MIR locus (Figure S1).

**Figure 2:**
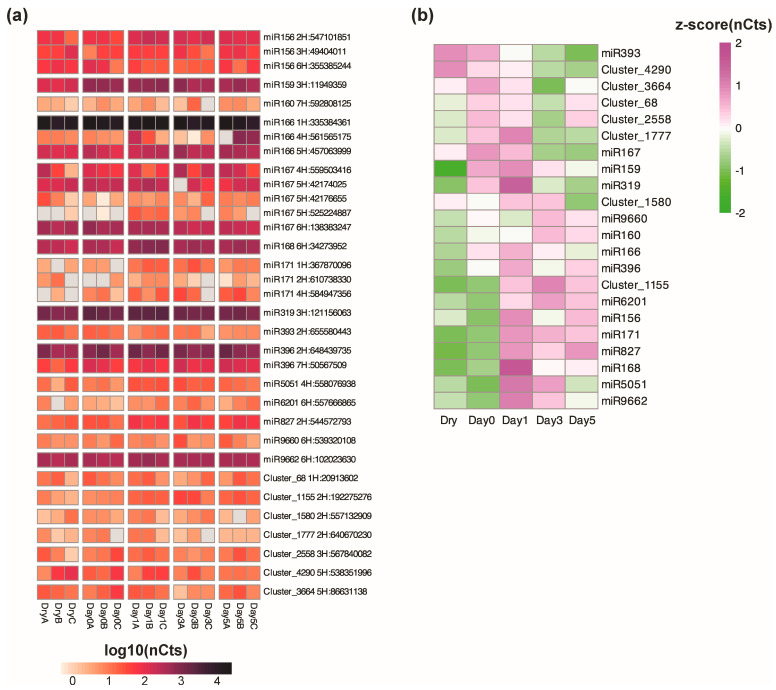
MicroRNA (miRNA) expression. (**a**) Heatmap representation of log10-transformed normalized read counts (nCts) aligned to the 33 identified miRNA loci (MIR) in each small RNA (sRNA) library. For each locus, the sum of reads aligned to the -5p and -3p arms of the putative MIR hairpin was used. Gray color corresponds to zero nCts. The chromosome and left coordinate are given for all loci, as well as the MIR family or the ShortStack ClusterID for loci expressing miRNA of unknown family. (**b**) Heatmap representation of the dynamic expression of each MIR family in the five malting stages. The mean nCts from libraries of the same stage were used, and for MIR families with multiple expressed loci, the sum of the nCts aligned to those loci was calculated prior to calculating the mean. Color corresponds to the z-score across stages of nCts.

There were 15 known MIR families and seven unknowns represented in these 33 loci (Figure 1d). Seven of these known miRNA families (miR156, miR159, miR166, miR167, miR168, miR171, and miR393) are found in both monocots and eudicots [50]. Seven (miR156, miR159, miR166, miR168, miR171, miR5051, and miR6201) of the 11 miRNA families that were expressed in both low viability and high viability barley seeds [51] were among the 15 miRNA families identified here (Figure 2b). Eight (miR156, miR159, miR166, miR167, miR168, miR171, miR393, and miR5051) of these 15 miRNA families were also reported in early developing barley seeds (1–15 days post anthesis) [38]. Expression of miRNAs from these families across different developmental stages and despite differences in samples used for sRNA library construction, suggests their crucial role in barley seed development and/or viability. The miR167 family was the most predominant family represented by five loci (Figure 2a). miRNAs belonging to the miR156, miR166, and miR171 families were expressed from three loci each. The miR396 family was represented by two distinct loci, while the remaining 10 miRNA families were singletons.

### 3.3. Expression of miRNAs During Barley Malting

To compare the expression of miRNAs within and among libraries, median-of-ratios normalization was performed on aligned read counts. Median-of-ratios normalization predicates that most genes are expressed at equal levels across all the libraries in our dataset. That is, each library has a small number of genes that are expressed differently from other samples. This assumption is valid when using all alignments (to 6393 sRNA loci), and not just alignments to MIR genes [33].

Loci belonging to several MIR families (miR166, miR167, miR396) were expressed at very different levels from each locus in the same family, while for other MIR families (miR156 and miR171), expression was similar from all loci in the family (Figure 2a). The miR166 locus on chromosome 1 was strongly expressed across the five malting stages, and its expression was approximately 90-times higher than the two other miR166 loci localized on chromosomes 4 and 5 (Figure 2a, Table S2). miR166 was also the most highly expressed family during maize seed germination [52]. Differential expression of miR166 family members in two Tibetan hulless barley varieties one day after seed imbibition has been reported [48]. Of the five miR167 loci, three were localized on chromosome 5 and exhibited very different levels of expression (Figure 2a). Abundance comparisons of different members in a miRNA family can provide valuable information about the role that MIR loci play in specific developmental stages or in response to specific external perturbations. This specificity could be determined by the cis elements in the promoters of these MIR loci and the cognate TFs that bind to them.

miR156, miR159, miR167, miR168, miR319, miR396, and miR9662 were classified as moderately expressed families (Figure 2a). The miR156 and miR168 families were reported to be the most abundantly expressed miRNAs at 1 and 5 days after germination in the barley variety Golden Promise [39]. We speculate these differences in the abundances of the known miRNAs could be due to varietal differences, tissue sampled for analysis (embryos versus whole seeds), or germination on filter paper versus malting procedure [53]. The seven MIR loci with an unknown family association were expressed at relatively low levels during malting (Figure 2a).

Differences in miRNA expression across malting stages were less pronounced than differences among miRNA families. A few miRNAs with differential expression across malting stages were identified (Figure 2a,b). miR159 family had higher expression at Day0 and Day1, while miR171 and miR827 showed higher abundance at Day1, Day3, and Day5. Expression of miR160 increased slowly during the five malting stages, while the novel miRNA on chromosome 5 showed the opposite pattern, with higher expression in dry seeds and lower expression levels as malting progressed. Several miRNA families showed stage-specific downregulation. This included miR6201 at Day0, four novel miRNAs at Day3, two novel miRNAs at Day5. These stage-specific expression trends implicate a role for these miRNAs during malting.

### 3.4. Targets of Malting Associated miRNAs

A reliable approach to establish the veracity of a miRNA is to detect its post-transcriptional regulatory activity on a target mRNA. Since most miRNAs show slicing activity on their targets, degradome analysis was pursued using the PARE technique [54]. The rationale was that having sRNA and degradome libraries from the same tissue samples or developmental stage would increase confidence in identified miRNA-mediated cleavage products of the cognate target mRNAs. Approximately 40 million reads corresponding to the uncapped 5′ ends of mRNAs were obtained from each of the five degradome libraries (Table 3). After the trimming of adapter sequences, most sequences were expected to be 20–21 nt, and indeed the mean length of clean reads in the five libraries was 20.2 nt (Table 3). Since miRNA-mediated slicing is site-specific, the relatively low percentage of unique reads is an indication of good degradome library quality.

**Figure 3:**
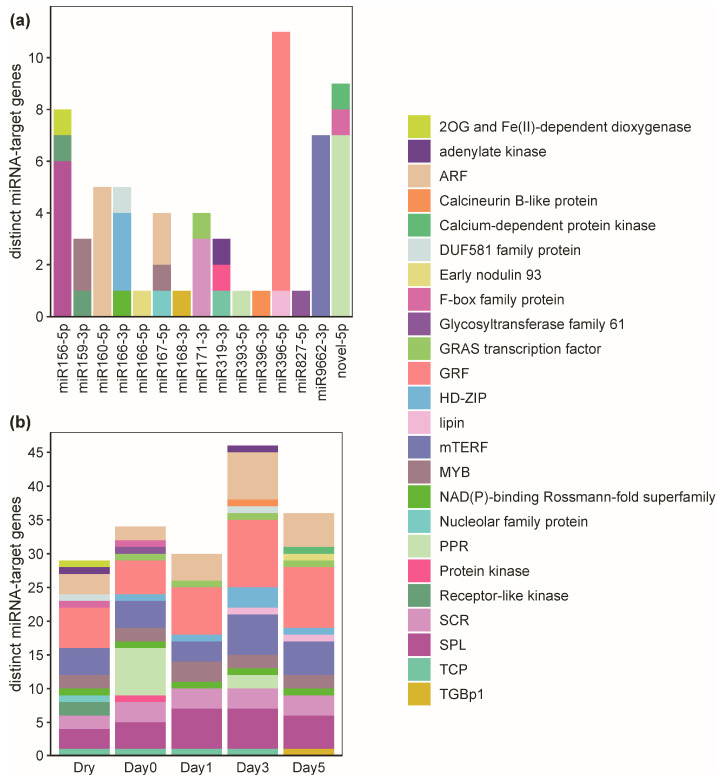
Stacked bar plots of distinct genes targeted by microRNA (miRNA) for slicing during malting. Color corresponds to gene description. (**a**) Genes targeted by each miRNA type (MIR family-hairpin arm position) and (**b**) Genes targeted in each stage are shown.

CleaveLand4 was used to predict miRNA-directed slice sites in the clean and size-filtered degradome data (Table S3). Of the 22 expressed miRNA families (15 known and 7 unknown/novel), 15 families (12 known and 3 unknown/novel) were predicted by CleaveLand4 to mediate slicing at specific mRNAs during malting (Figure 3a). Seven miRNA families were specific for a single-gene target family, while the other eight miRNA families targeted two or three gene families. Notably, although miRNA from both -5p and -3p arms of each MIR locus hairpin were detected by sRNA-seq, we only found evidence of mRNA slicing directed by the miRNA from a single arm of each MIR family, except for miR166 and miR396 family loci (Figure 3a).

**Figure 4:**
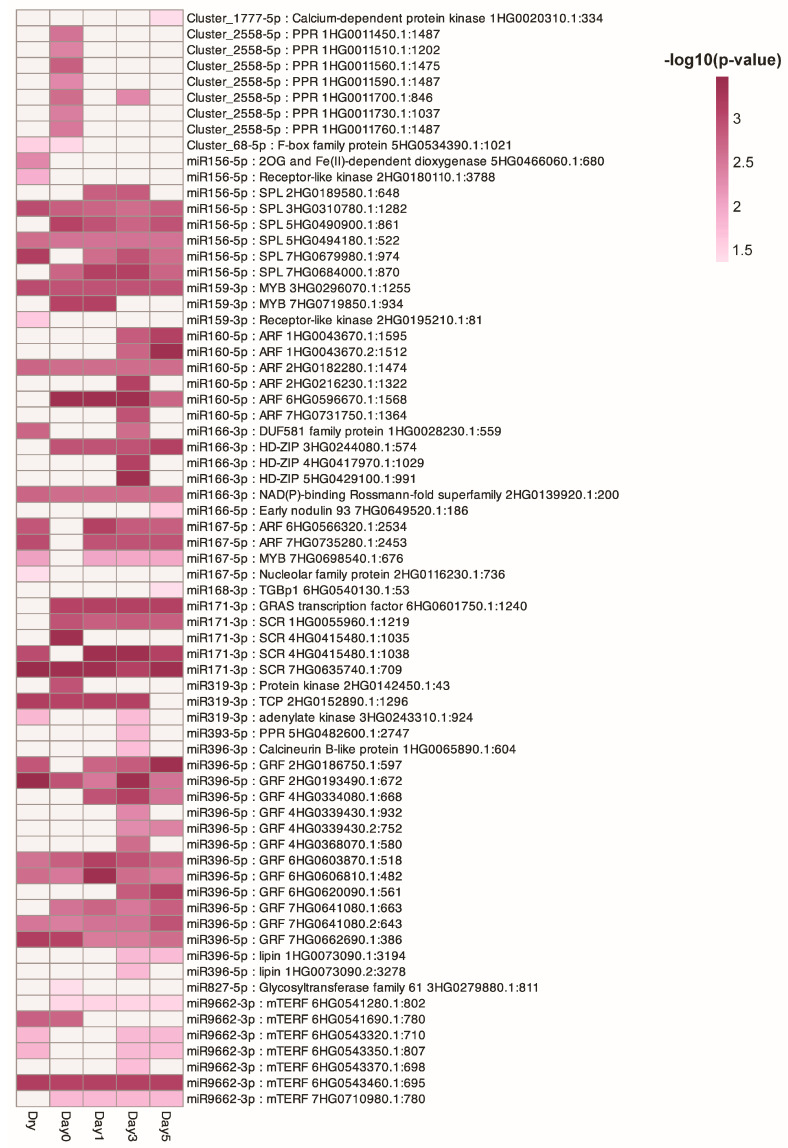
Transcript slice sites directed by expressed microRNA (miRNA) in each malting stage. Slice sites are specified by the targeting miRNA type (MIR family or ClusterID-hairpin arm position), target gene description, targeted messenger RNA transcript, and slice position in the transcript. Heatmap color corresponds to -log10 transformed *p*-value of the slice site in the given malting stage. Gray corresponds to *p*-value > 0.05.

The number of distinct miRNA-targeted genes was similar across the malting stages, with the least in dry seed (29) and the most in Day3 (46) (Figure 3b). Target genes encoding several TFs were consistently observed across all the malting stages, while some gene families (e.g., Pentatricopeptide repeat (PPR)) were predominantly identified as miRNA targets at Day0. The 69 predicted slice sites were in 64 distinct genes, due to five genes having two predicted slice sites (Figure 4, Table S3). Of those five, four genes (*HORVU.MOREX.r3.1HG004670*, *HORVU.MOREX.r3.1HG0073090*, *HORVU.MOREX.r3.4HG033940*, *HORVU.MOREX.r3.7HG064080*) have slice sites in two different transcripts, and one gene (a scarecrow TF *HORVU.MOREX.r3.4HG0415480*) has two closely spaced sites in the same transcript.

### 3.5. Post-Transcriptional Gene Regulation During Malting

Based on the annotations of the 64 target genes identified in this study, two cellular component ontologies, mitochondria and nucleus, were predominant. Initiating and efficiently utilizing stored reserves in the endosperm for energy production is pivotal for germination [55]. This involves rapid organellar reorganization processes, especially the mitochondria. Energy metabolism is complemented by dynamic biochemical changes, especially nucleic acid synthesis, which is vital for cell division, differentiation, and growth. These rapid biochemical changes enable the establishment of stem cell niches to aid organ development, regulated by an array of TFs [56]. Interactions between hormone receptors and TFs mediated by phytohormones play a key role during germination [57].

Malting of barley grain for brewing is a germination process under controlled conditions. It is important to point out that the microenvironment during malting is significantly different from normal germination processes in laboratory or field conditions. During the first stage of malting, the steeping regime involves alternating cycles of submergence in water (hypoxia) and air rests (reoxygenation), at the end of which *senso stricto* germination is completed with the emergence of tiny rootlets (called chits) (Day0 time point). Starch degradation in normally germinating seeds fuels rapid heterotrophic growth needed for transitioning to the autotrophic phase. On the other hand, in malting, heterotrophic growth (robust development of shoots and roots) is undesirable [53]. The germination regimen in malting involves conditions such as darkness, crowding, and intermittent rotation, all of which are inhibitory for heterotrophic growth but permissive for the proliferation of hydrolytic enzymes and for the breakdown of cell walls into their component part, β-glucan.

### 3.6. Energy Metabolism

Pathways associated with energy metabolism are some of the earliest biochemical responses triggered during the seed imbibition/steeping stage. Studies in rice indicate components required to build or import mitochondrial proteins are present in promitochondrial structures and activated immediately upon imbibition to aid the rapid mitochondrial biogenesis and associated increases in respiration observed in the first 24 h after imbibition [58,59]. The transition from the protomitochondrial structures in the dormant seeds to a mature functional mitochondrion during seed imbibition most likely involves signals from this organelle to the nucleus. The identification of the novel MIR locus cluster_2558 and miR393 targeting the PPR-containing protein family associated with organellar RNA metabolism [60] was very interesting. All seven target PPR genes on chromosome 1 show higher expression levels at Day0 compared with dry seeds (Figure 4, Table S3) and overlap with the degradome data and the expression of novel cluster_2558-5p miRNA (Figure 1b). Common phenotypes associated with mutations in PPR genes are seedling lethality and slow growth, resulting from deficits in energy supply [60]. Post-transcriptional regulation of PPRs by novel cluster_2558-5p miRNA and miR393 at Day0 may be involved in energy homeostasis during early stages of malting. It has been reported that miR393 expression leads to a reduction in coleoptile length in response to submergence in rice seeds and is triggered by abscisic acid (ABA) [61]. Similarly, the submergence of barley seeds during the steeping process could lead to a reduction in coleoptile length mediated by miR393 and its cognate PPR (see discussion on phytohormones later).

Seven genes annotated as mitochondrial transcription termination factor (mTERF) were identified as targets of miR9662-3p (Figure 3a and Figure 4). miRNA9662 was expressed most highly at Day3, and the expression of the seven target genes was reduced at Day3 (compared to Day1 expression) (Figure S6). It is well known that m*TERFs* regulate the expression of mitochondrial genes [62] and are also targeted to the chloroplasts [63]. Arabidopsis *mTERF4* has been suggested to be a key factor mediating communication between chloroplasts/mitochondria and the nucleus [64]. In barley, 60 *mTERF*s have been identified and shown to be involved in the development process, tolerance to diverse abiotic stresses, and phytohormone responses [65]. We speculate the miRNA-triggered downregulation of inter-organellar crosstalk may be unique to the malting process, since two other reports on normal barley germination do not report these targets [38,39]. The precise role of mTERFs and miR9662 during barley malting/germination warrants further investigation.

Identification of Early Nodulin 93 (ENOD93) as a target of miR166-5p (minor arm) during barley malting is fascinating. In cereals, ENOD93 has been shown to be associated with nitrogen use efficiency [66]. Recently, it was reported that the absence of ENOD93 caused a mitochondrial complex IV–dependent progressive loss of respiration rate, affecting membrane potential and cellular ATP production. Furthermore, adenylate levels and metabolic pathways were perturbed in the absence of ENOD93, leading to reduced root growth [67]. Even though miR166 was reported in two other barley germination reports [39,68] ENOD93 has not been identified as a target, suggesting that regulation of ENOD93 by miR166-5p may be unique to the malting process.

### 3.7. Transcriptional Regulation and Phytohormones

Precise spatial and temporal gene expression is a must to ensure proper growth and development in plants [69,70]. Hormonal gradients play a key role in the spatial regulation of the TFs and miRNAs that can only be determined by using cell-type-specific analysis. Nonetheless, previous studies have established that miRNAs and TFs are among the key regulators that determine gene expression, thus impacting the physiology and phenotype of the plant [71]. During germination, expression of several TF families with an explicit connection to phytohormones [72] such as auxin response factors (ARFs) [73], MYBs [74,75], squamosa promoter binding element proteins (SPLs) [76], growth-regulating factors (GRFs) [77], teosinte branched 1/cycloidea/proliferating cell factors (TCPs) [78], homeodomain leucine zippers (HD-ZIPs) [79], and scarecrow (SCR) [80] have been reported. In the degradome analysis, we identified 40 TF encoding genes from various malting stages that belonged to these seven different TF families.

**Figure 5:**
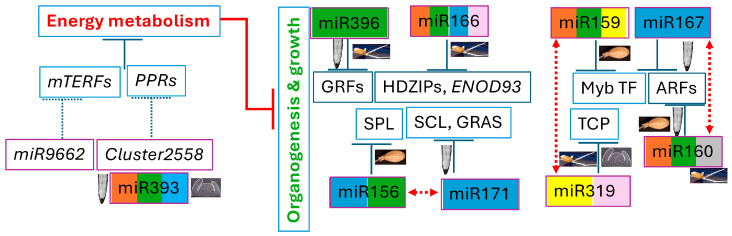
Model showing the interactions between microRNAs (miRNAs), target genes, and phytohormones during barley malting. MicroRNAs are in blocks with a pink border, and target genes are in boxes with blue borders. Novel miRNAs and novel targets are shown in italics. The colors inside the boxes indicate various phytohormones associated with each miRNA. ABA—brown, GA—green, Auxin—blue, Ethylene—yellow, JA—pink and brassinosteroids—gray. Red dotted lines indicate interactions between the miRNAs. Inset pictures next to the miRNAs represent seed germination, root growth, root meristem, and shoot apical meristem.

Using the Kyoto Encyclopedia of Genes and Genomes (KEGG) pathway analysis, ten miRNAs identified during barley seed development and germination have been predicted to be involved in phytohormone signaling: miR156, miR159, miR160, miR164, miR167, miR172, miR319, miR390, miR393, and miR396 [39]. In another germination study using Tibetan hulless barley, miR166 was identified along with miR156, miR159, miR160, miR164, miR172, miR319, and miR393 [48]. Nine of these known miRNAs were also identified during the various barley malting stages and were associated with signaling by ABA (miR156, miR159, miR167, and miR393), auxin (miR160, miR166, miR167 and miR393), ethylene (miR156, miR159, miR164, miR390), gibberellic acid (GA) (miR156, miR159, and miR319) and JA (miR319 and miR396) [39,81]. It is evident that most miRNAs are involved in at least two different hormone signaling pathways, highlighting the complex interplay among these phytohormones in regulating seed germination and the post-germinative phase of development in barley (Figure 5).

MYB TFs are positive regulators of ABA responses during germination and are subject to ABA-dependent miR159 regulation [75]. The first identified GA signaling component in the barley aleurone, GAMYB [82], positively transduces the GA signal to activate expression of α-amylase and other hydrolytic enzymes [83], as well as promote programmed cell death in the aleurone [74,84]. Thus, miR159 plays a key role in regulating the dynamic germination process via modulation of the GA and ABA phytohormone signaling cascades. Since miR159 has regulatory effects on ABA and GA, and ethylene exhibits cross-talk with ABA and GA, it is hypothesized that this miRNA may have direct or indirect control over ethylene-mediated regulation during seed germination and dormancy [85].

The establishment of the stem cell niche, cell polarity, abaxial/adaxial symmetry, and meristem proliferation is regulated by site-specific hormonal responses in which developmentally regulated TFs play a regulatory role in specific cell types. Establishment of these niches/boundaries is crucial for the proper growth and development of the roots and shoots following the seed imbibition process. However, as pointed out earlier, in the context of malting, the microenvironment conditions are not permissive for heterotrophic growth. It was thus supportive to identify a high number of TF-miRNAs associated with regulating the root meristem establishment, proliferation, and root growth. The GRF-miR396 as a regulator of auxin gradient and local signaling through gibberellins [86] and SPL-miR156 together with auxin [87] are crucial players in determining root meristem size and proliferation. ARFs-miR160-miR167, along with auxin [88] and ABA [73,89], SCR-miR171, along with GA, are involved in root morphogenesis and development [90]. The HDZips-miR166-auxin module is involved in primary root growth [91]. In contrast to the number of miRNAs-targets associated with root meristem organization and development, only one was identified for the shoot meristem. The TCP-miR319-auxin-ABA has been shown to be important in the organization of the shoot apical meristem and leaf developmental processes [92]. This study shows the post-transcriptional gene regulation mediated by miRNAs on their cognate target genes, probable interactions between different miRNAs and phytohormone signaling, and/or responsive genes to dampen the growth processes in response to the microenvironmental conditions prevailing during barley malting. Future research using single-cell RNA sequencing, integrating in situ spatial techniques, will enable a precise understanding of the fine-tuning at a molecular level.

## Figures and Tables

**Table 1:** Small RNA library read processing statistics.

Stage	Rep ^a^	Raw	Clean		Clean and Size-Filtered (18–24 nt ^b^)			
		Reads	Reads	Mean Length	Reads	Mean Length	Duplicate	Unique
		M ^c^	M	Nt	M	nt	%	%
Dry								
	A	7.4	6.7	29.8	1.1	21.0	83.2	16.8
	B	10.8	10.0	32.7	1.4	21.1	88.8	11.2
	C	14.8	13.8	35.1	1.7	21.2	90.5	9.5
Day0								
	A	16.1	15.0	34.7	1.9	21.5	89.1	10.9
	B	24.7	21.9	30.8	3.2	21.0	91.8	8.2
	C	17.2	16.2	36.1	1.4	21.6	90.0	10.0
Day1								
	A	26.4	24.4	30.6	4.6	21.4	93.3	6.7
	B	21.2	19.9	32.8	2.7	21.5	92.0	8.0
	C	17.1	14.8	31.9	2.4	21.4	91.7	8.3
Day3								
	A	11.7	10.6	34.6	1.6	21.6	86.9	13.1
	B	22.2	21.0	31.5	4.1	21.1	94.4	5.6
	C	4.1	3.8	30.6	0.7	21.3	87.8	12.2
Day5								
	A	18.1	17.2	31.3	3.7	21.4	93.6	6.4
	B	9.1	8.5	35.1	1.2	21.8	89.6	10.4
	C	39.6	31.5	29.8	6.0	21.1	95.9	4.1

^a^ replicate (Rep). ^b^ nucleotide (nt). ^c^ million (M).

**Table 2:** Small RNA read mapping to Morex V3 genome assembly with ShortStack4.

Stage	Rep ^a^	Input ^b^	Mapped		Mapped		Mapped		Mapped		Unmapped	
			Total		Uniquely		MMAP ^c^		MMAP		Total	
							Local ^d^		Random ^e^			
		M ^f^	M	% ^g^	M	%	M	%	M	%	M	%
Dry												
	A	1.13	0.32	28.7	0.20	17.8	0.08	7.3	0.04	3.6	0.80	71.3
	B	1.39	0.40	28.8	0.24	17.4	0.11	7.8	0.05	3.6	0.99	71.2
	C	1.69	0.50	29.5	0.30	17.8	0.12	6.9	0.08	4.8	1.19	70.5
Day0												
	A	1.89	0.51	27.1	0.30	15.8	0.13	7.1	0.08	4.1	1.38	72.9
	B	3.19	0.92	28.9	0.53	16.5	0.27	8.5	0.13	3.9	2.27	71.1
	C	1.43	0.35	24.2	0.20	13.9	0.09	6.0	0.06	4.3	1.08	75.8
Day1												
	A	4.61	1.05	22.7	0.64	13.8	0.30	6.5	0.11	2.4	3.56	77.3
	B	2.66	0.70	26.4	0.41	15.4	0.24	8.9	0.05	2.1	1.96	73.6
	C	2.38	0.57	24.0	0.34	14.2	0.16	6.8	0.07	3.0	1.81	76.0
Day3												
	A	1.62	0.34	20.8	0.21	12.7	0.09	5.7	0.04	2.4	1.28	79.2
	B	4.11	1.10	26.7	0.63	15.3	0.39	9.6	0.07	1.8	3.01	73.3
	C	0.73	0.17	22.8	0.10	13.5	0.05	6.8	0.02	2.4	0.57	77.2
Day5												
	A	3.65	0.81	22.2	0.49	13.4	0.25	6.9	0.07	1.9	2.84	77.8
	B	1.19	0.26	21.5	0.16	13.6	0.07	5.7	0.03	2.2	0.93	78.5
	C	5.98	1.54	25.7	0.89	14.9	0.53	8.8	0.11	1.9	4.45	74.3

^a^ replicate (Rep); ^b^ clean and size-filtered (18–24 nucleotides); ^c^ multi-mapped (MMAP); ^d^ multi-mapping reads placed by local probability, only uniquely mapped reads used as weights, corresponding to ShortStack alignment default parameter “u”; ^e^ multi-mapping reads placed at random to one of their multi-mapping locations; ^f^ million (M) reads; ^g^ % of input reads.

**Table 3:** Parallel analysis of RNA ends (PARE) library read processing statistics.

Stage	Raw	Clean		Clean and Size-Filtered (19–21 nt ^a^)			
	Reads	Reads	Mean Length	Reads	Mean Length	Duplicate	Unique
	M ^b^	M	Nt	M	nt	%	%
Dry	32.4	32.2	20.2	31.5	20.3	79.0	21.0
Day0	44.0	43.8	19.8	41.4	20.1	79.7	20.3
Day1	35.5	35.2	19.2	30.3	20.1	78.2	21.8
Day3	47.3	47.2	20.2	45.7	20.3	70.3	29.7
Day5	41.2	41.0	19.8	37.7	20.3	73.4	26.6

^a^ nucleotide (nt), ^b^ million (M).

## Data Availability

The datasets generated and analyzed for the current study are available in the SRA repository, under BioProject ID PRJNA1024431. The BioProject and associated SRA metadata are available at https://www.ncbi.nlm.nih.gov/sra/PRJNA1024431, accessed on 18 May 2026.

## Supplementary Materials

The following supporting information can be downloaded at: https://www.mdpi.com/article/10.3390/genes17060676/s1.

## Abbreviations

ABA, abscisic acid; GA, gibberellic acid; HC, high-confidence; Hv, *Hordeum vulgare*; MIR, microRNA locus; miRNA, microRNA mRNA, messenger RNA; NGS, next generation sequencing; nCts, normalized counts; nt, nucleotide; PARE, parallel analysis of RNA ends; pre-miRNA, precursor microRNA; pri-miRNA, primary microRNA; siRNA, small interfering RNA; sRNA, small RNA; sRNA-seq, small RNA sequencing; TF, transcription factor.
