# Supplementary material for: Post-Transcriptional Gene Regulation by MicroRNAs During Barley Malting
Source: Genes (Basel). 2026 Jun 9;17(6):676. doi: 10.3390/genes17060676 (PMC13299409; doi:10.3390/genes17060676)

Figure S6

Expression across the five malting stages of the 64 genes with CleaveLand4 category 0 predicted slice sites. Expression is represented as the mean transcripts per million (TPM) +/- standard deviation, n = 3. The normalized read count data are publicly available at NCBI's Gene Expression Omnibus through GEO Series accession number GSE 295574 (https://www.ncbi.nlm.nih.gov/geo/query/acc.cgi?acc=GSE295574).

expression of CleaveLand4 category 0 predicted targets of miRNA-mediated slicing
page 1 of 6

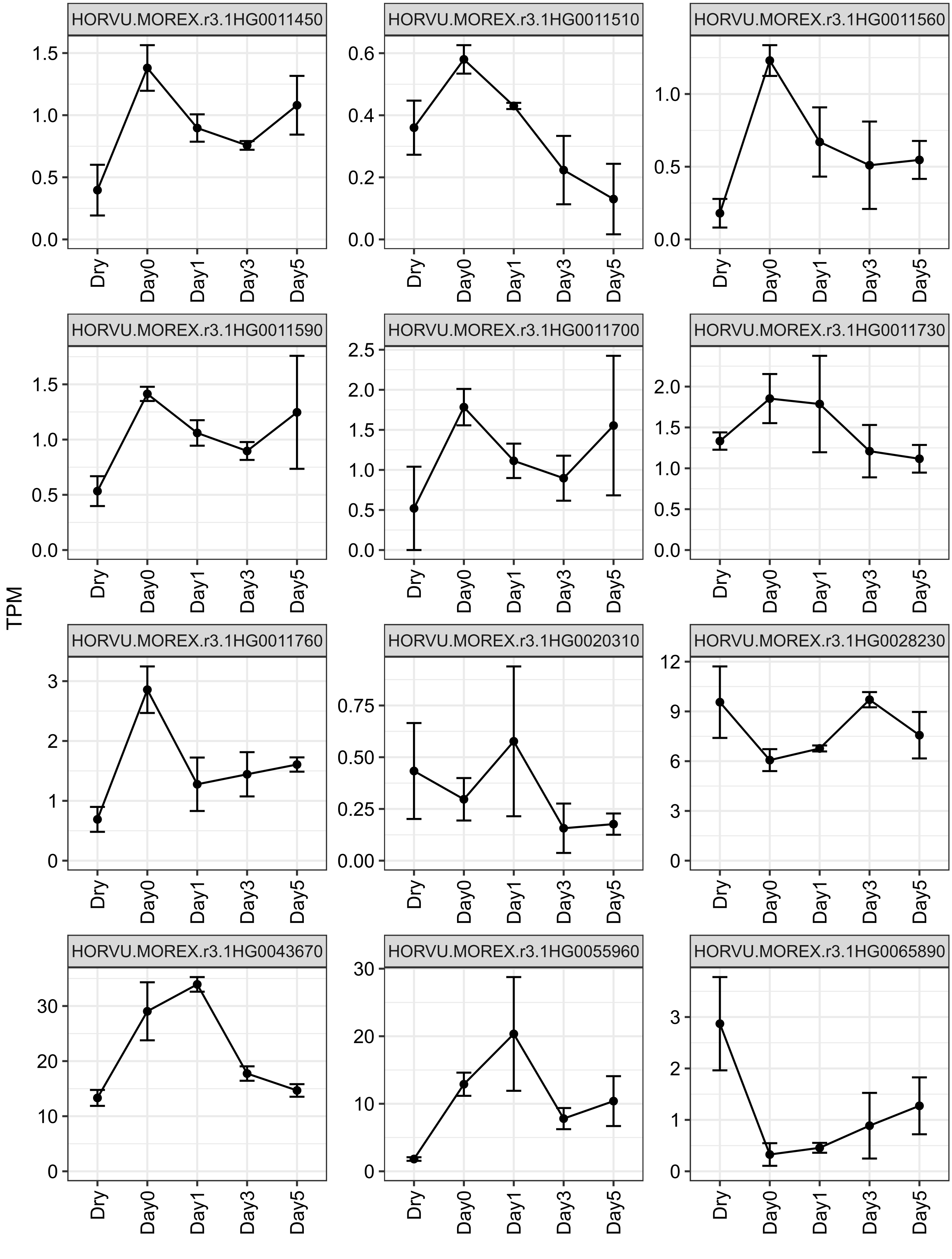

expression of CleaveLand4 category 0 predicted targets of miRNA-mediated slicing

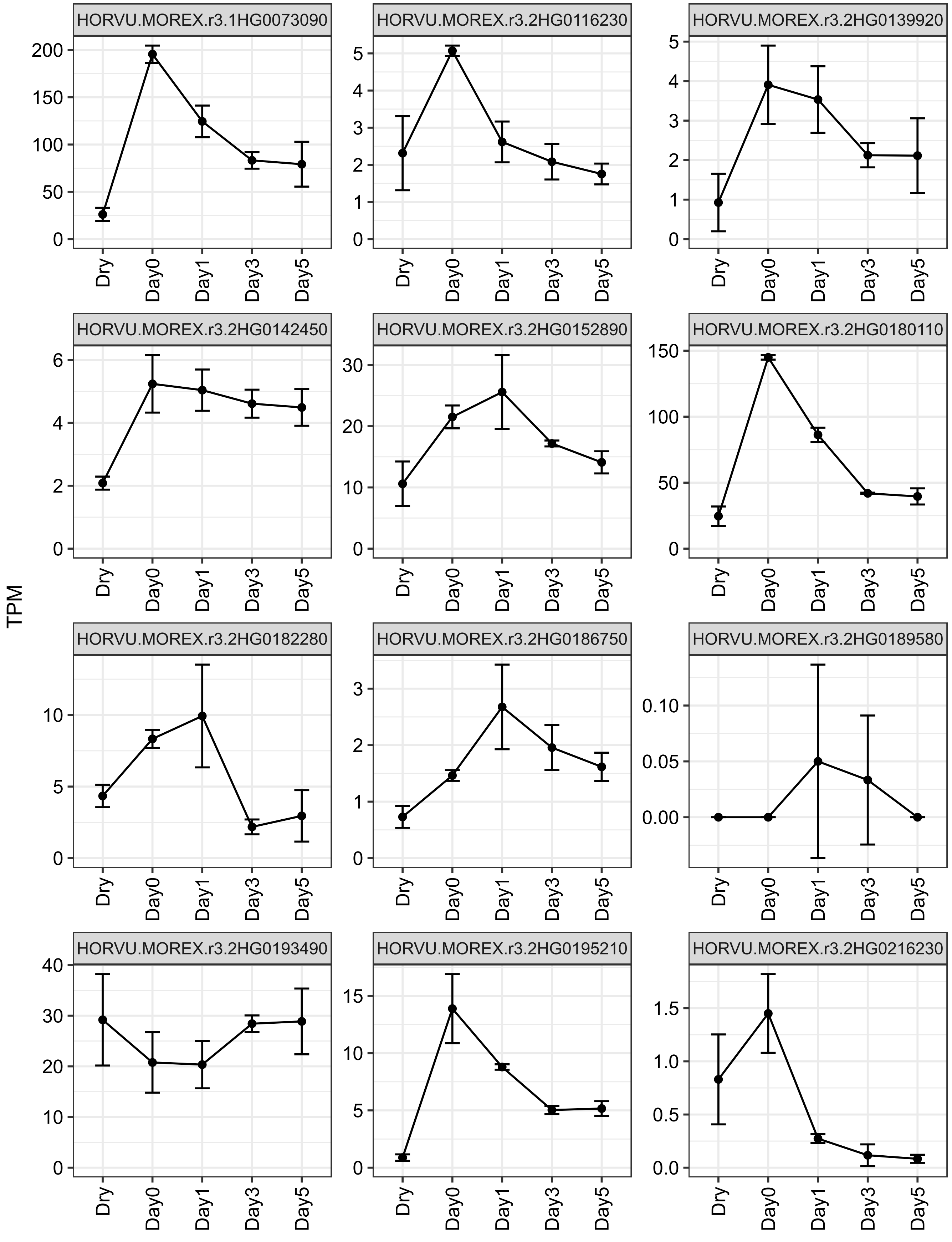

expression of CleaveLand4 category 0 predicted targets of miRNA-mediated slicing
page 3 of 6

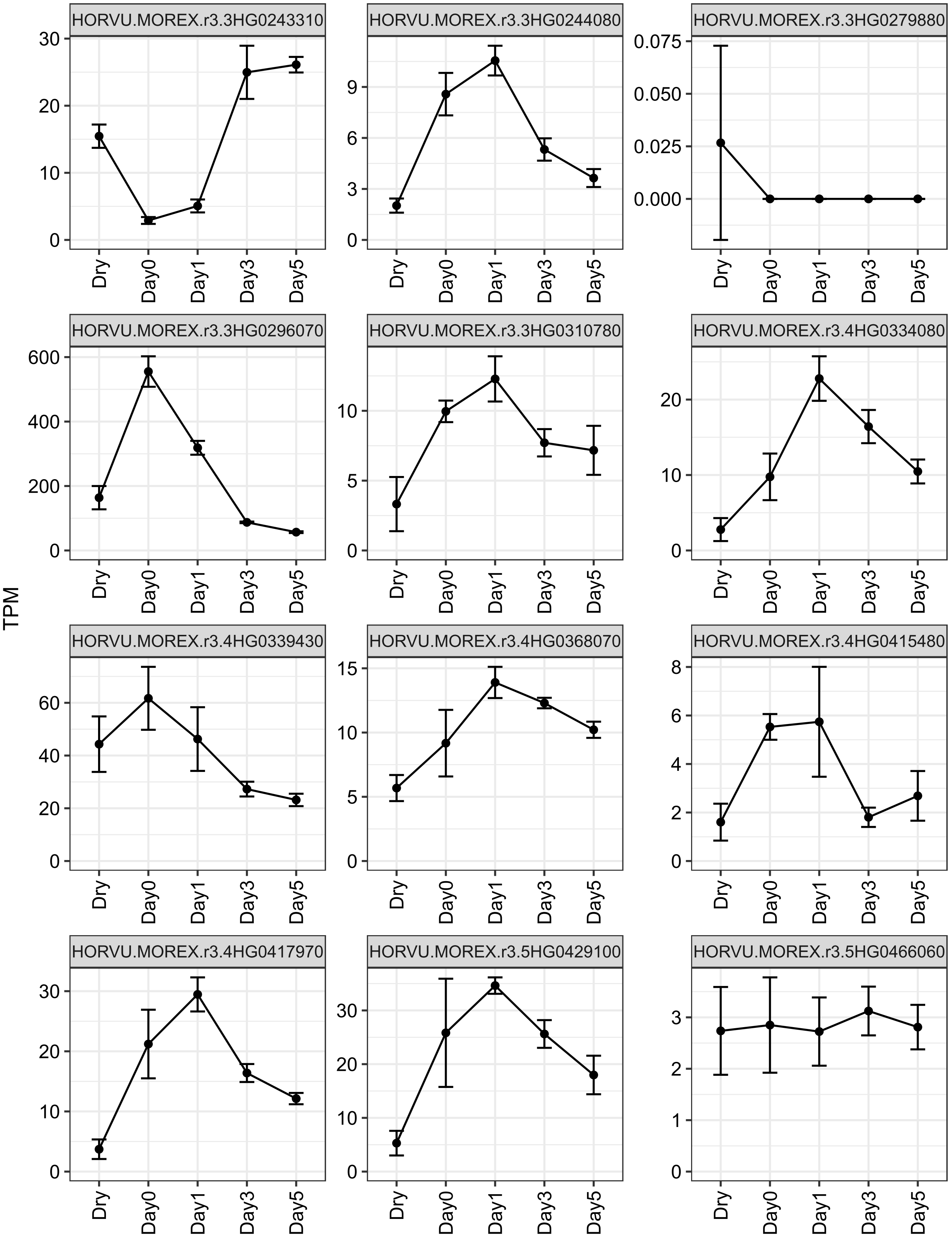

expression of CleaveLand4 category 0 predicted targets of miRNA-mediated slicing

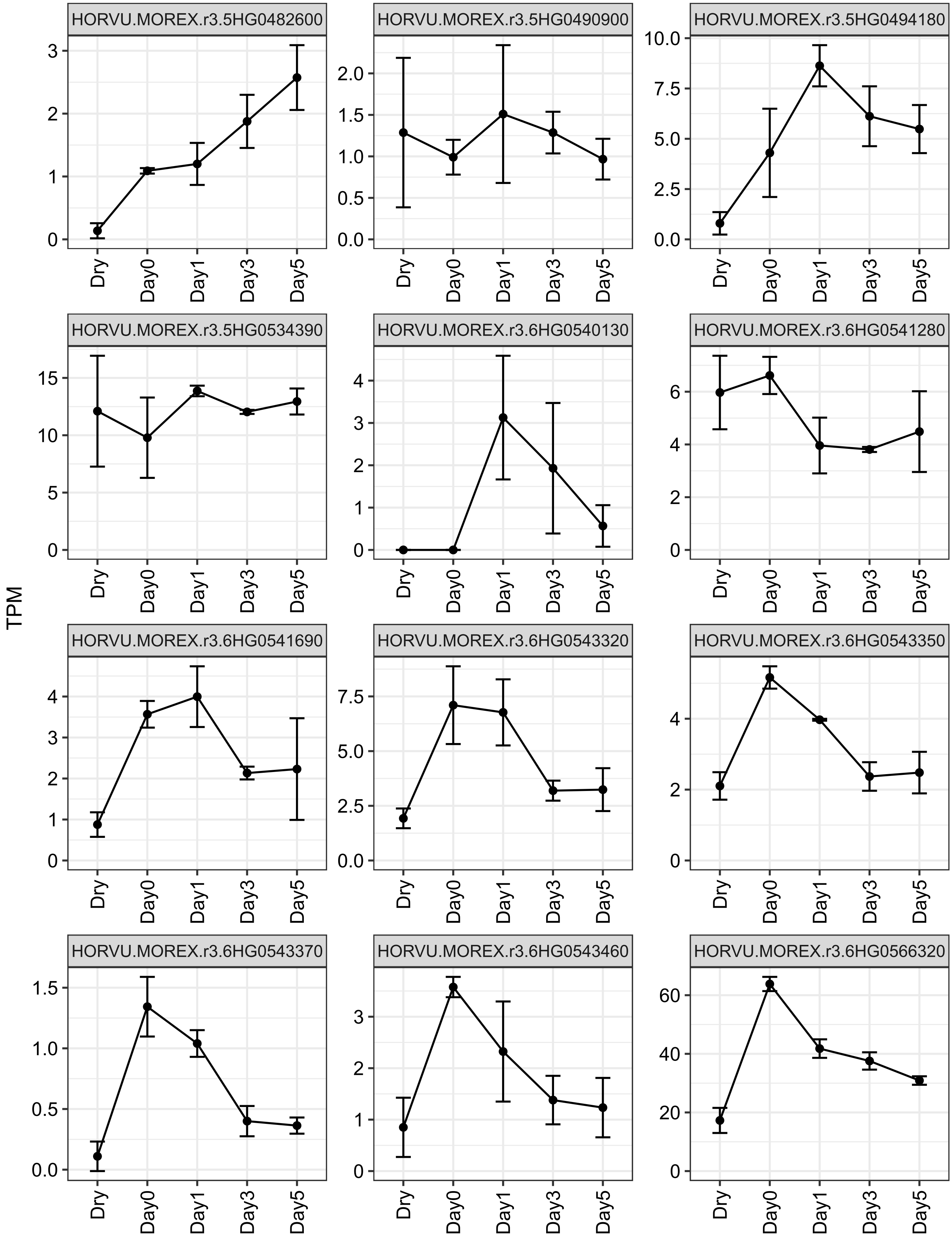

expression of CleaveLand4 category 0 predicted targets of miRNA-mediated slicing
page 5 of 6

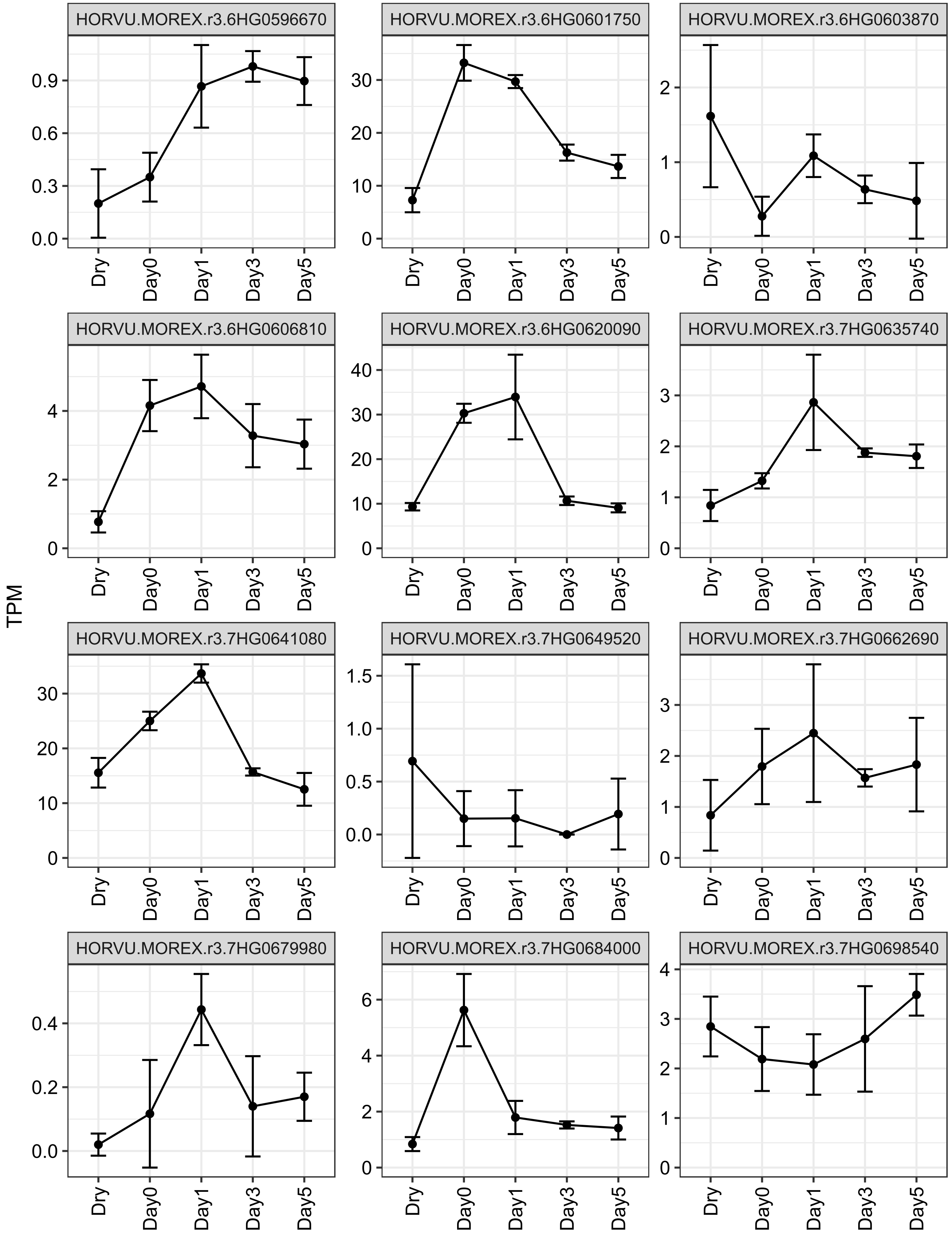

expression of CleaveLand4 category 0 predicted targets of miRNA-mediated slicing

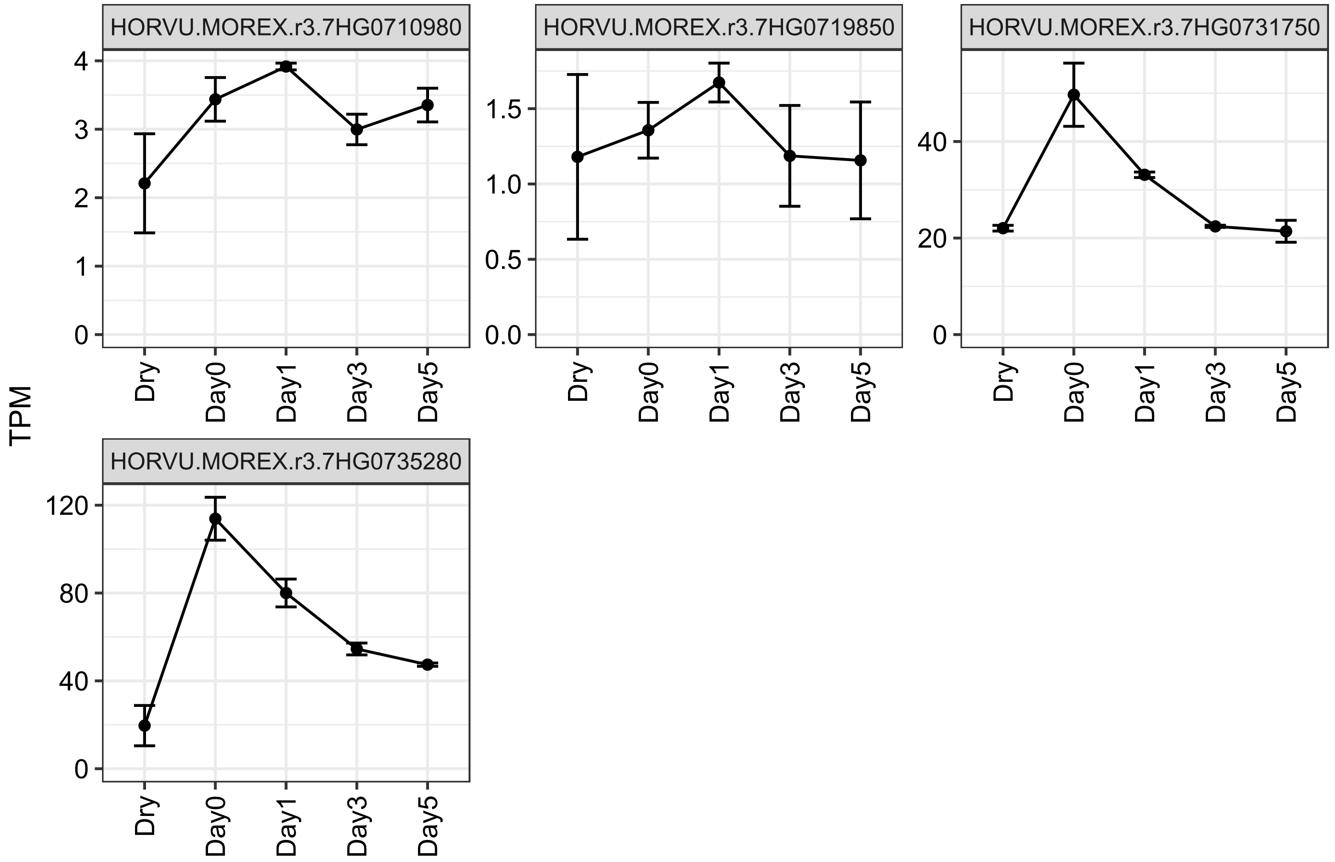

Supplement: Supplementary file 1 [file genes-17-00676-s001.zip › supplemental_figures1-6_tables1-4_20250513/FigureS6_expression_cat0targets.pdf]
