# Supplementary material for: Post-Transcriptional Gene Regulation by MicroRNAs During Barley Malting
Source: Genes (Basel). 2026 Jun 9;17(6):676. doi: 10.3390/genes17060676 (PMC13299409; doi:10.3390/genes17060676)
Supplement: Supplementary file 1 [file genes-17-00676-s001.zip › supplemental_figures1-6_tables1-4_20250513/FigureS5_T-plots.pdf]

Figure S5

T-plots of all CleaveLand4 category 0 and category 1 slice sites

D = degradome
T = transcript
Q = query, targeting miRNA
S = slice site position in transcript
category = CleaveLand4 slice site category
p = p-value of slice site

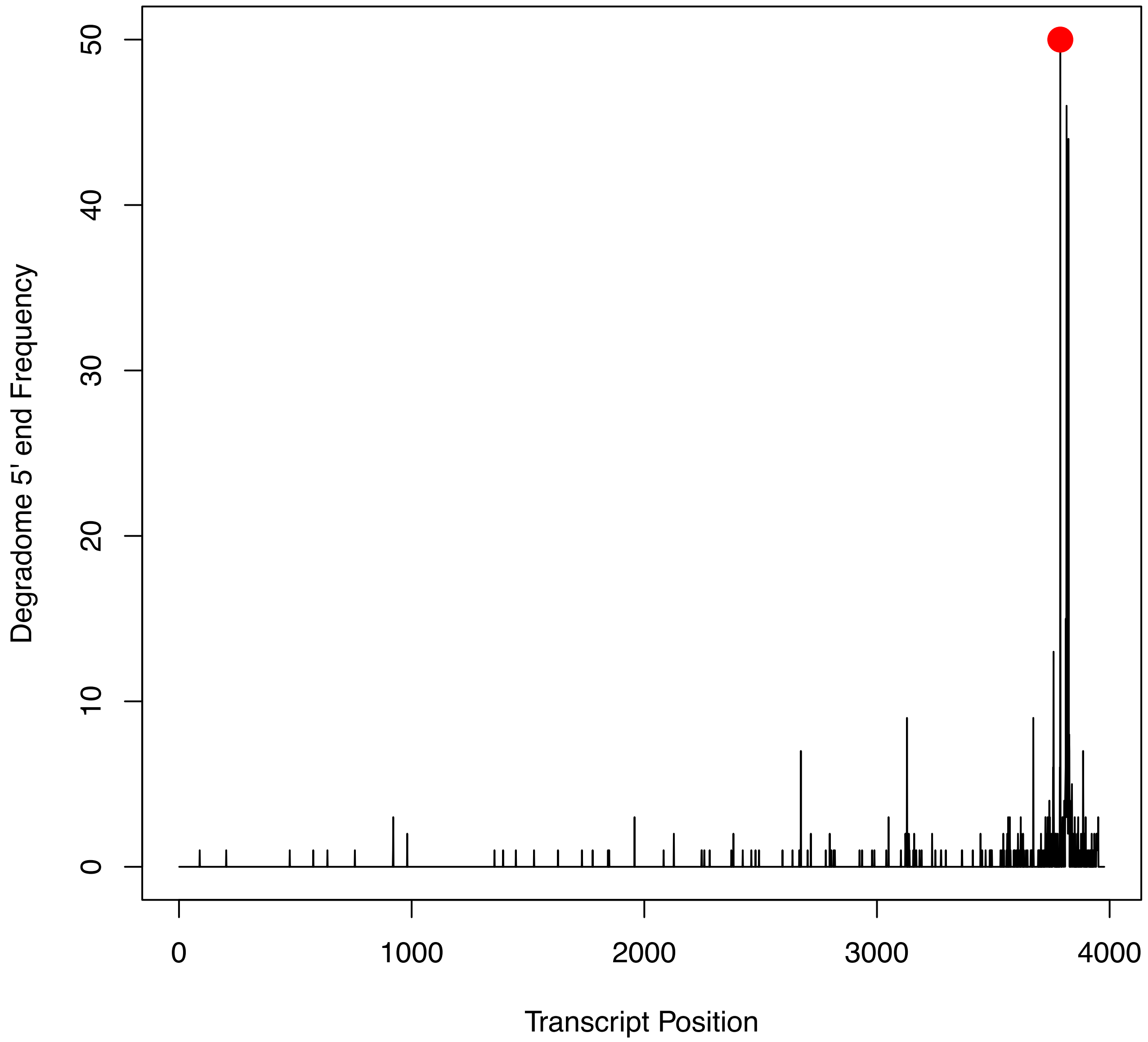

D=Dry
T=HORVU.MOREX.r3.2HG0180110.1
Q=miR156–5p.Cluster_1557.Cluster_1973.Cluster_4963
S=3788
category=0
p=0.0125013214601848

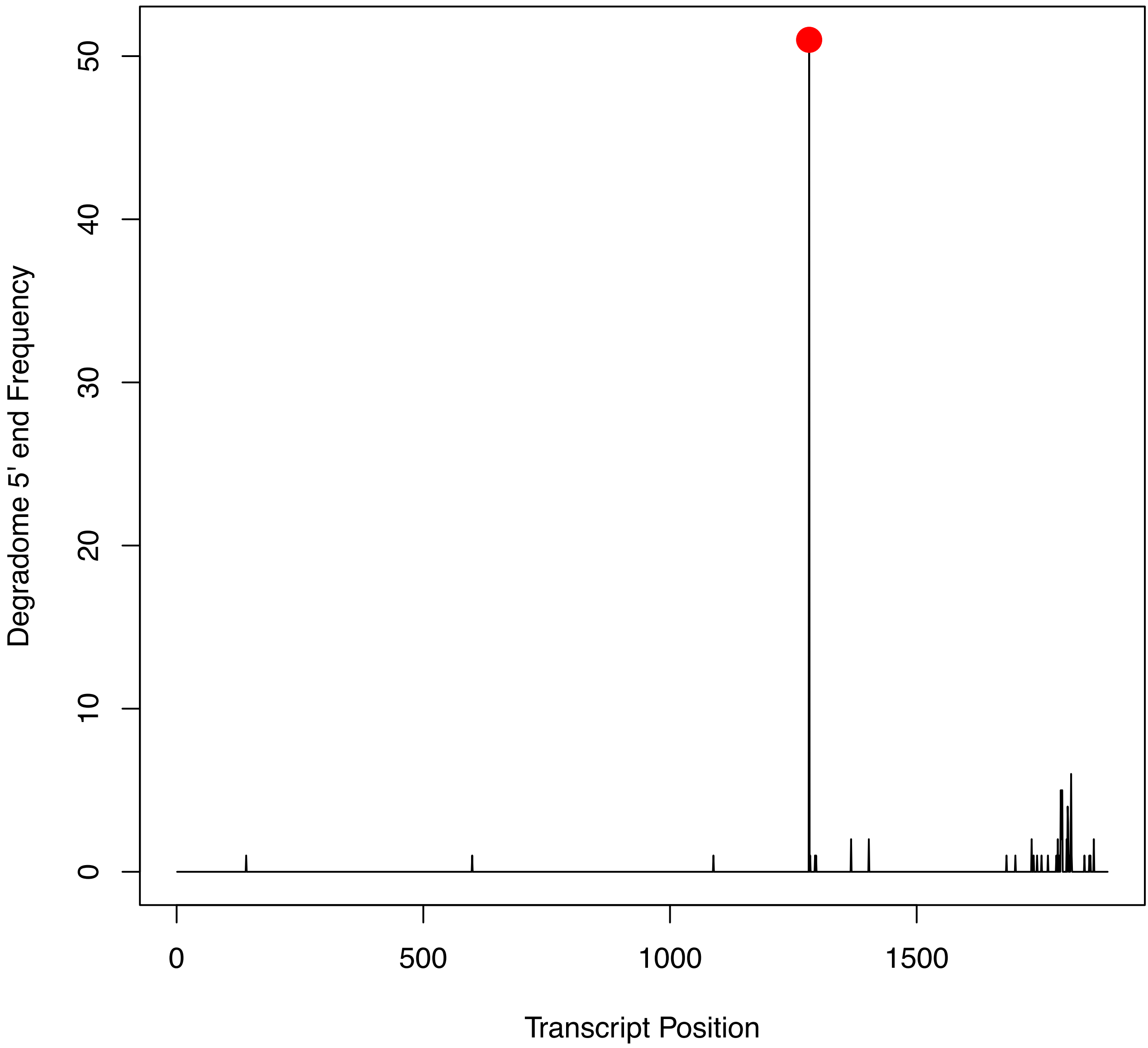

D=Dry
T=HORVU.MOREX.r3.3HG0310780.1
Q=miR156–5p.Cluster_1557.Cluster_1973.Cluster_4963
S=1282
category=0
p=0.00101948972865074

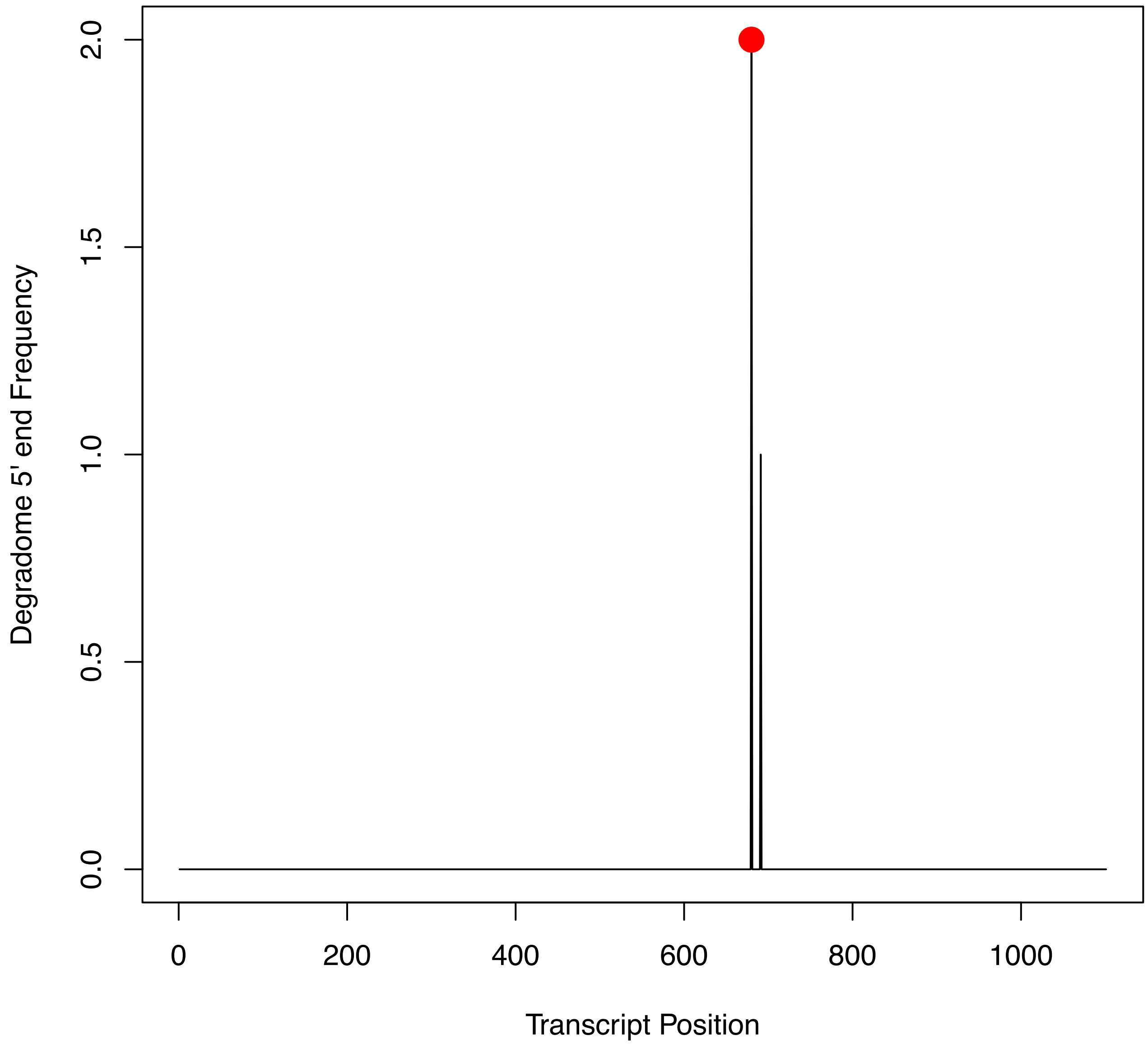

D=Dry
T=HORVU.MOREX.r3.5HG0466060.1
Q=miR156–5p.Cluster_1557.Cluster_1973.Cluster_4963
S=680
category=0
p=0.0047487344923044

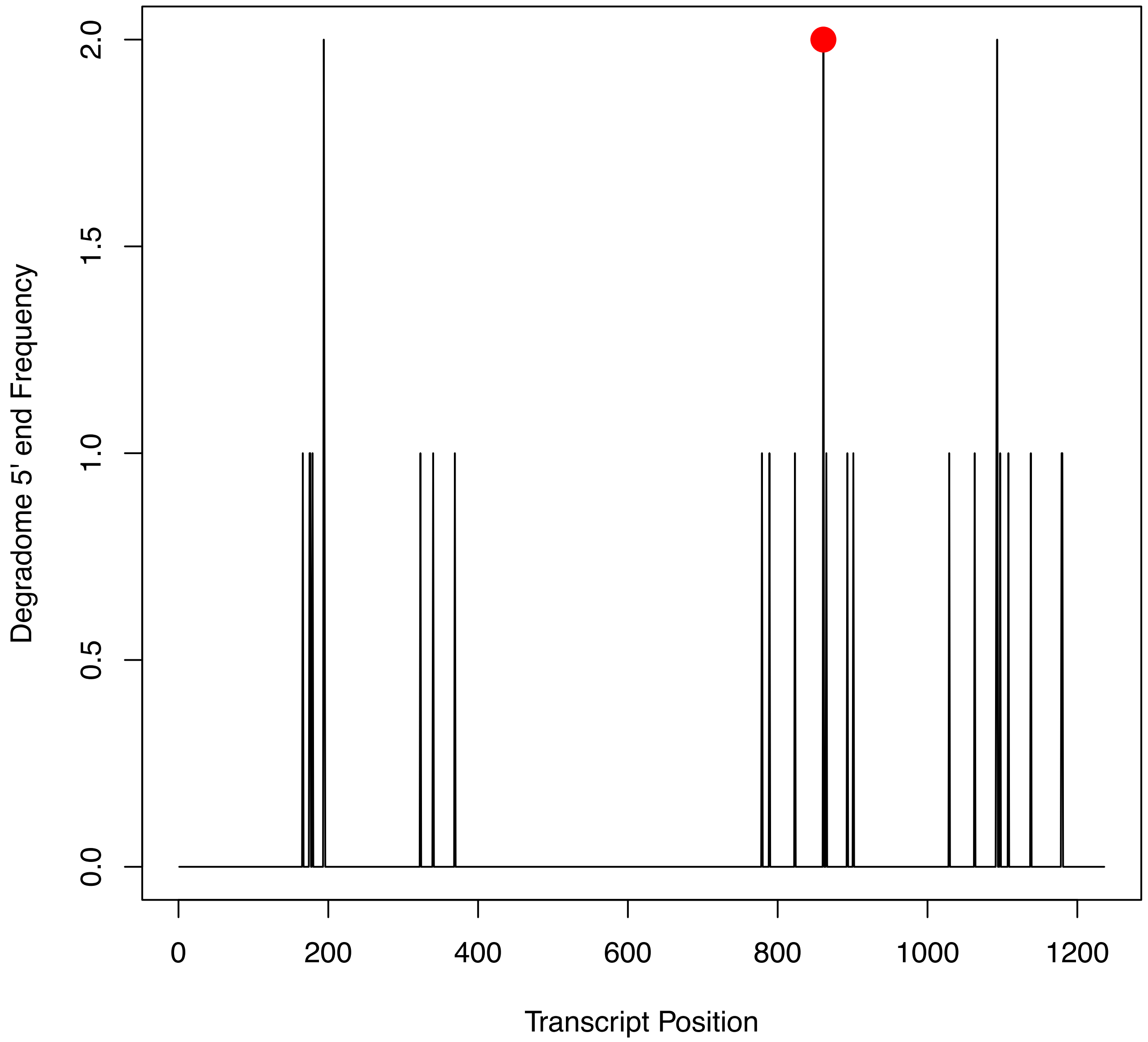

D=Dry
T=HORVU.MOREX.r3.5HG0490900.1
Q=miR156–5p.Cluster_1557.Cluster_1973.Cluster_4963
S=861
category=1
p=0.00101775970573414

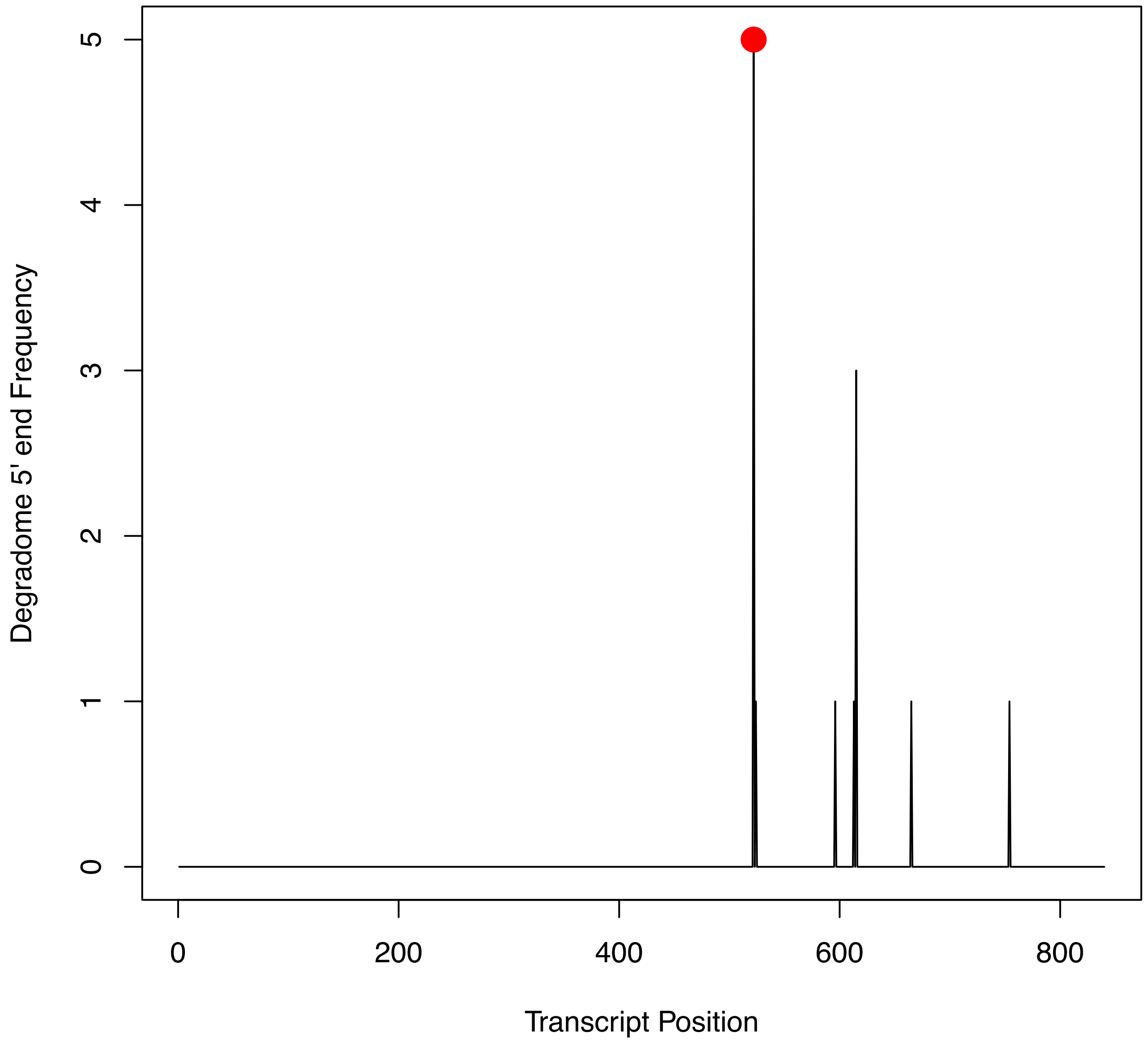

D=Dry
T=HORVU.MOREX.r3.5HG0494180.1
Q=miR156–5p.Cluster_1557.Cluster_1973.Cluster_4963
S=522
category=0
p=0.00237719276888249

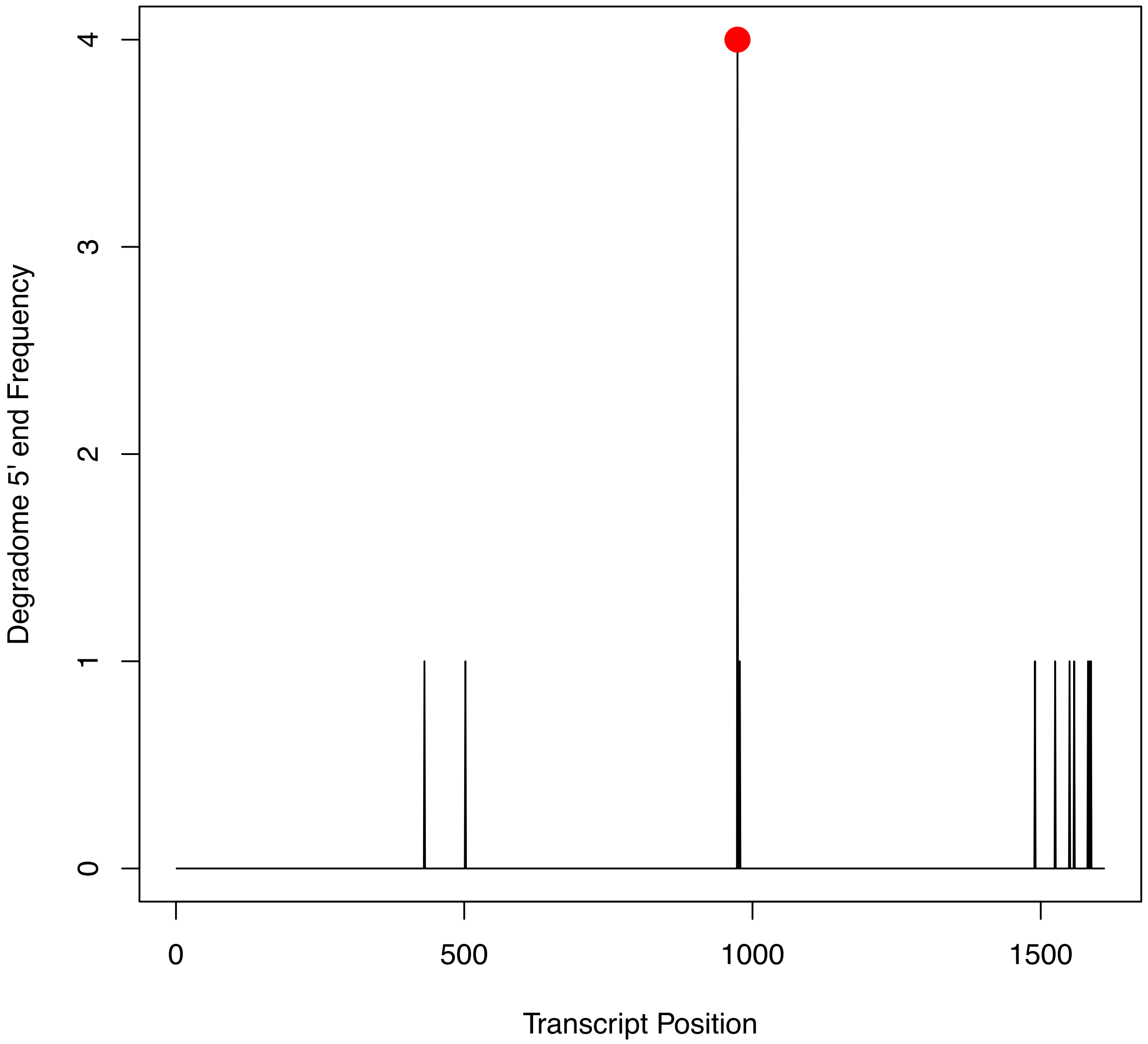

D=Dry
T=HORVU.MOREX.r3.7HG0679980.1
Q=miR156–5p.Cluster_1557.Cluster_1973.Cluster_4963
S=974
category=0
p=0.000679775355825751

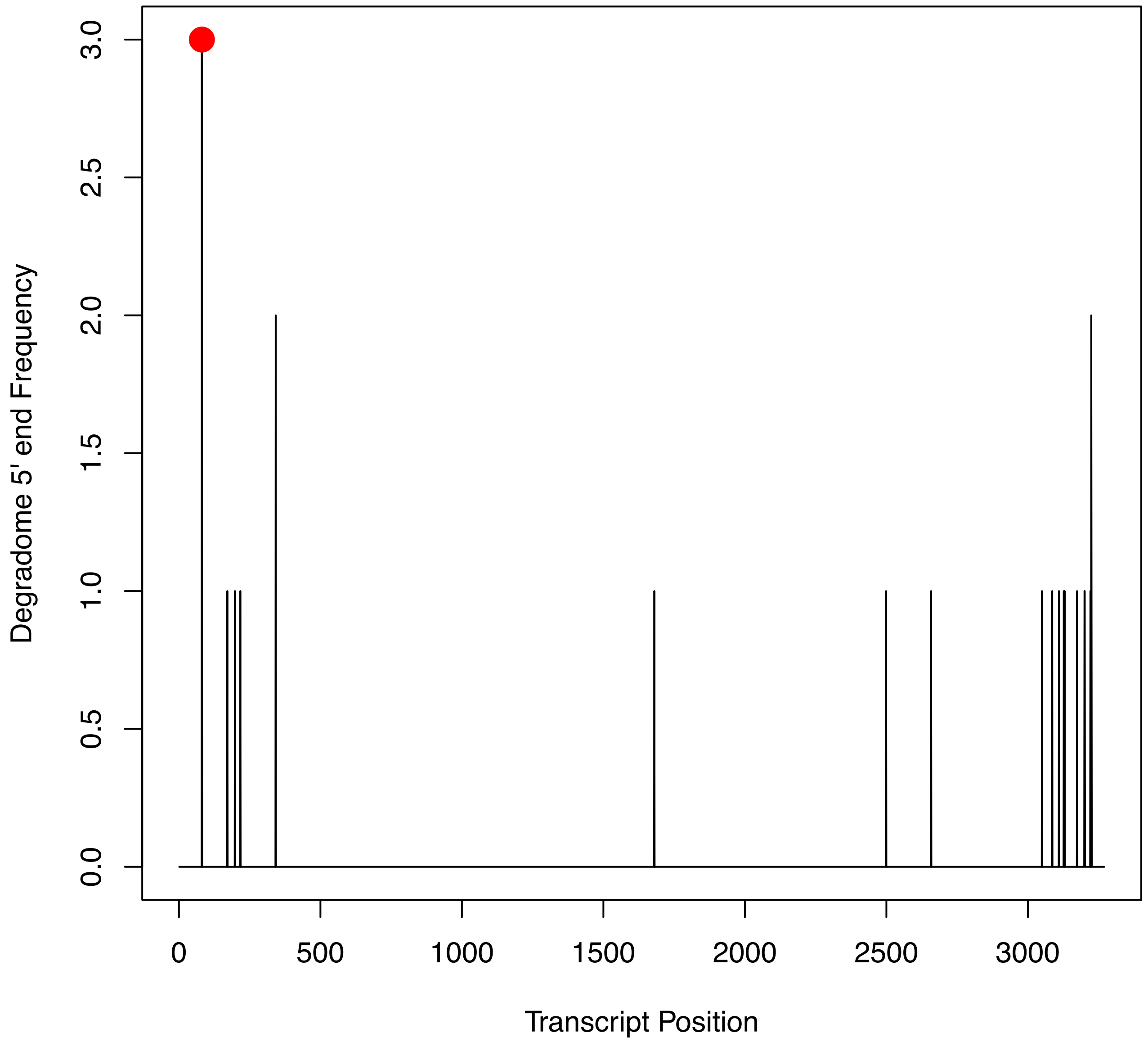

D=Dry
T=HORVU.MOREX.r3.2HG0195210.1
Q=miR159–3p.Cluster_1875
S=81
category=0
p=0.0248463598821187

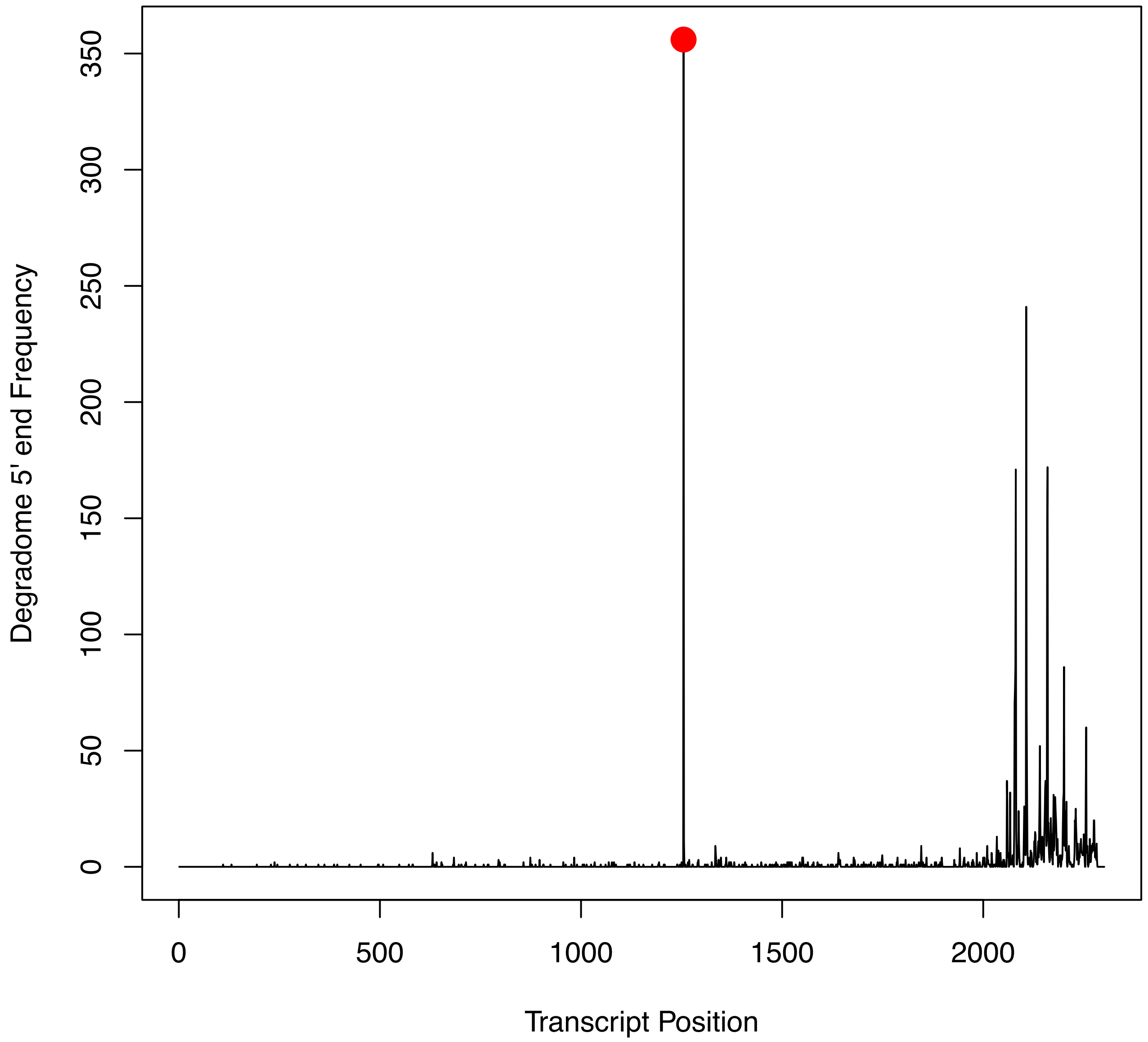

D=Dry
T=HORVU.MOREX.r3.3HG0296070.1
Q=miR159–3p.Cluster_1875
S=1255
category=0
p=0.00101948972865074

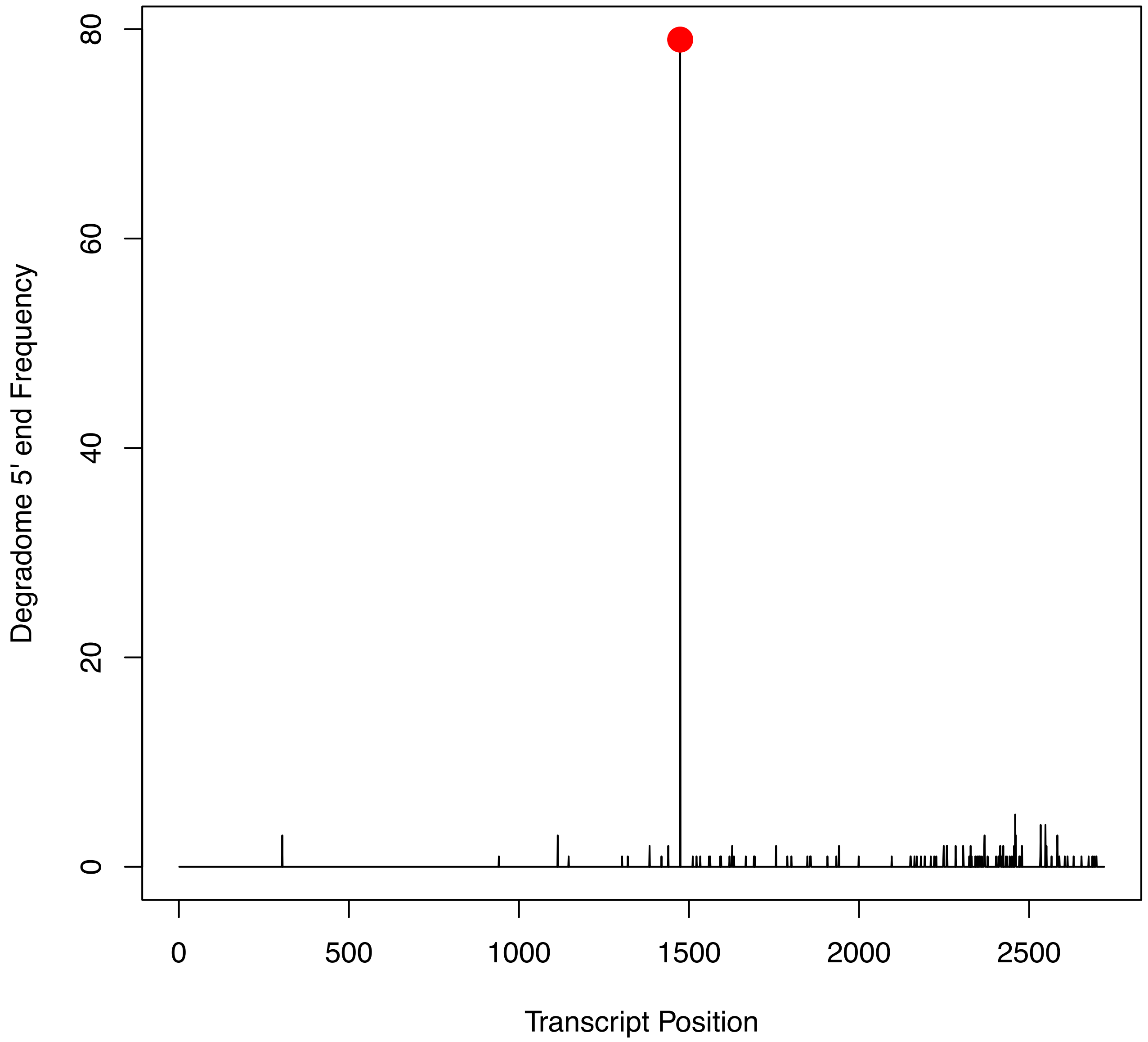

D=Dry
T=HORVU.MOREX.r3.2HG0182280.1
Q=miR160–5p.Cluster_6224
S=1474
category=0
p=0.00203794009799463

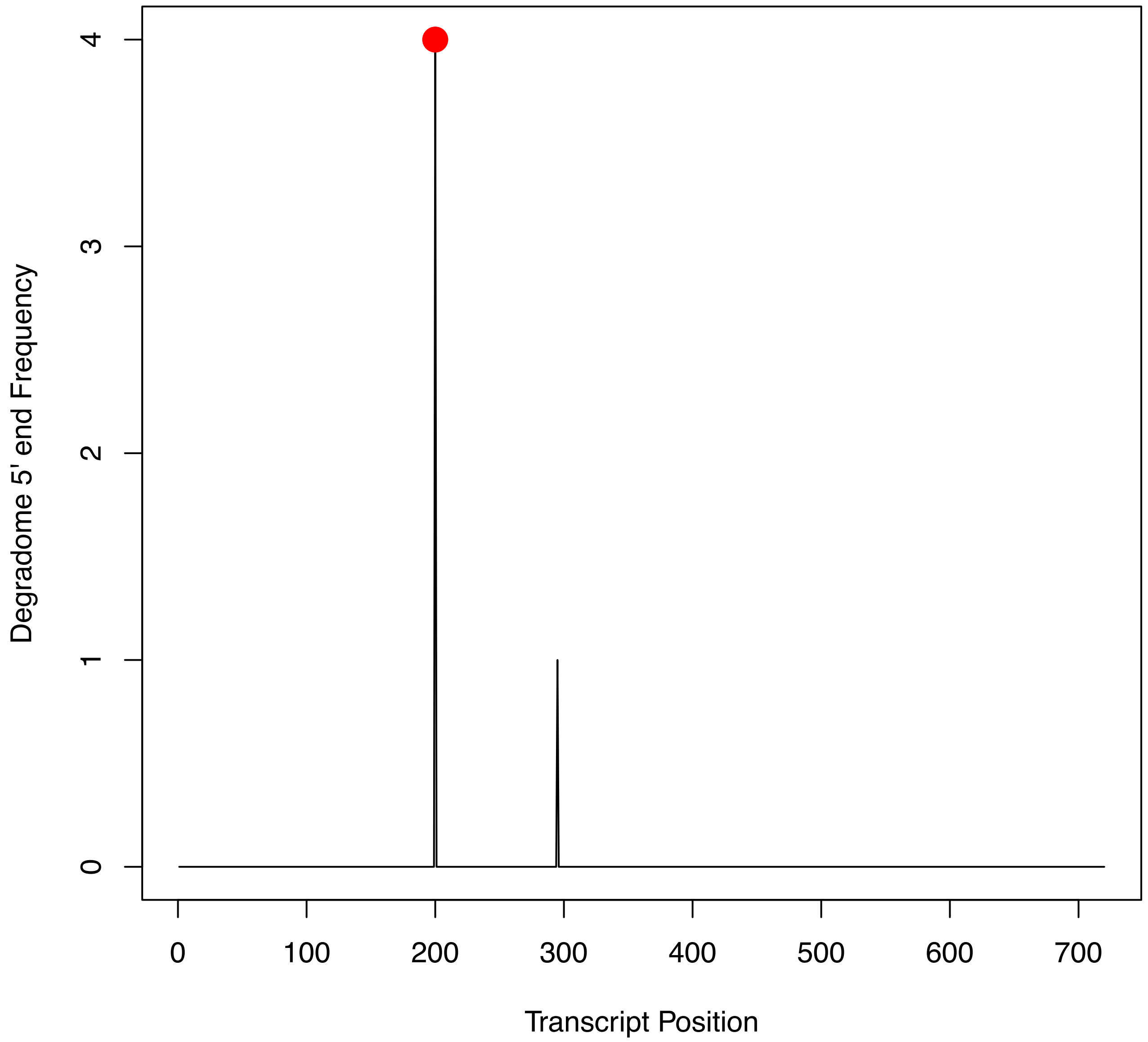

D=Dry
T=HORVU.MOREX.r3.2HG0139920.1
Q=miR166–3p.Cluster_426.Cluster_3396
S=200
category=0
p=0.00203794009799463

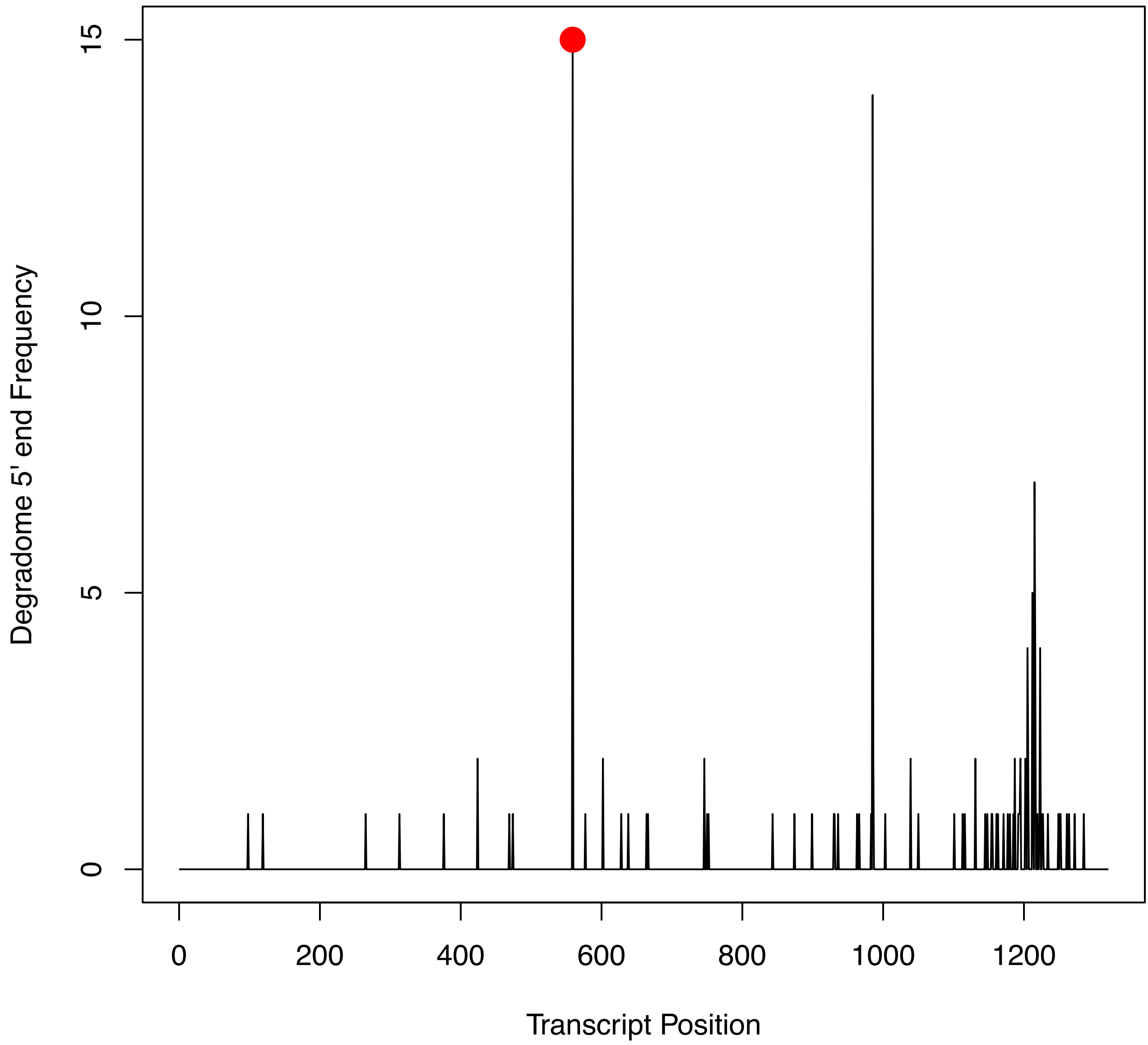

D=Dry
T=HORVU.MOREX.r3.1HG0028230.1
Q=miR166–3p.Cluster_4051
S=559
category=0
p=0.00203794009799463

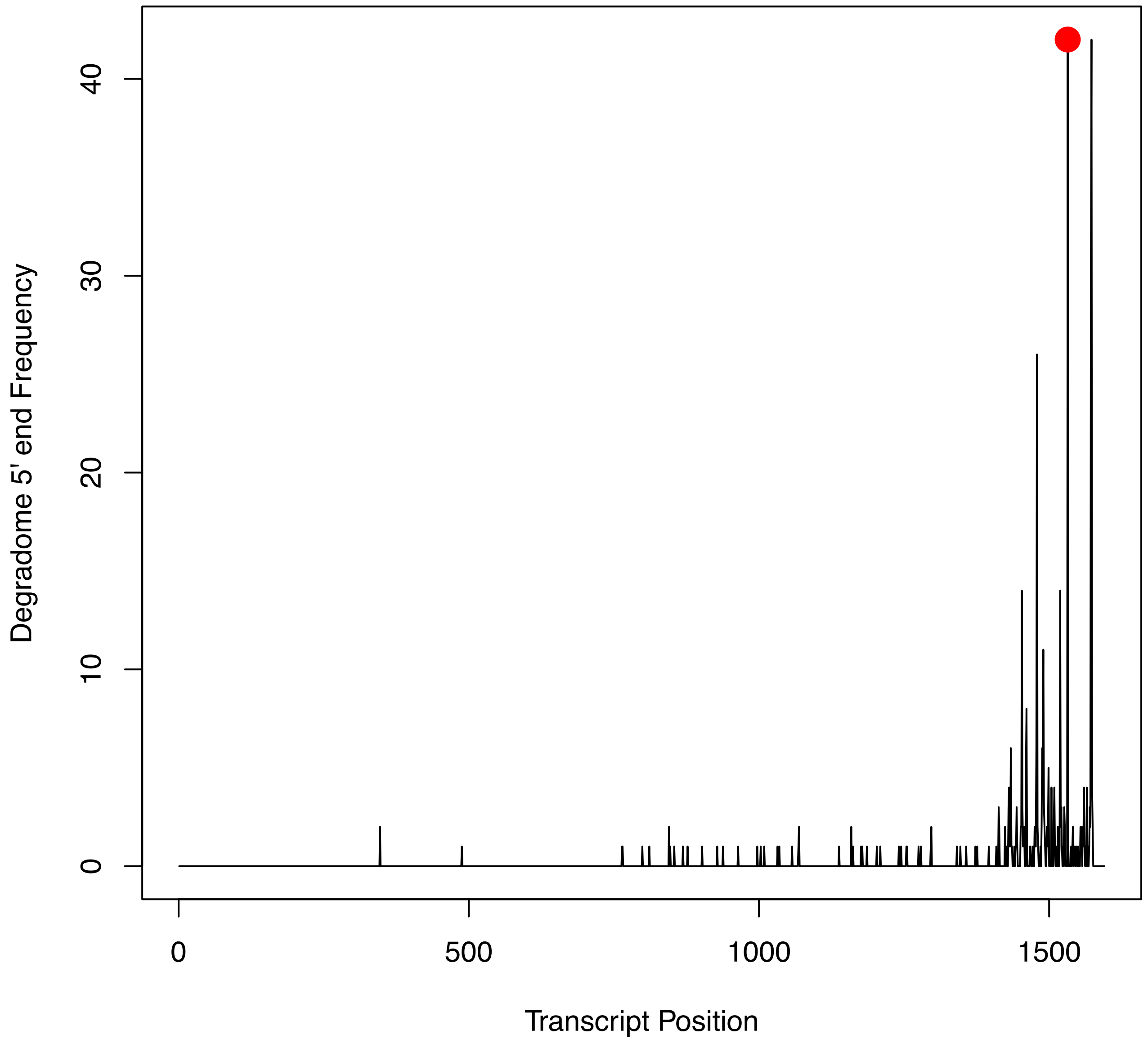

D=Dry
T=HORVU.MOREX.r3.7HG0688280.1
Q=miR166–3p.Cluster_4051
S=1532
category=1
p=0.0266235411509872

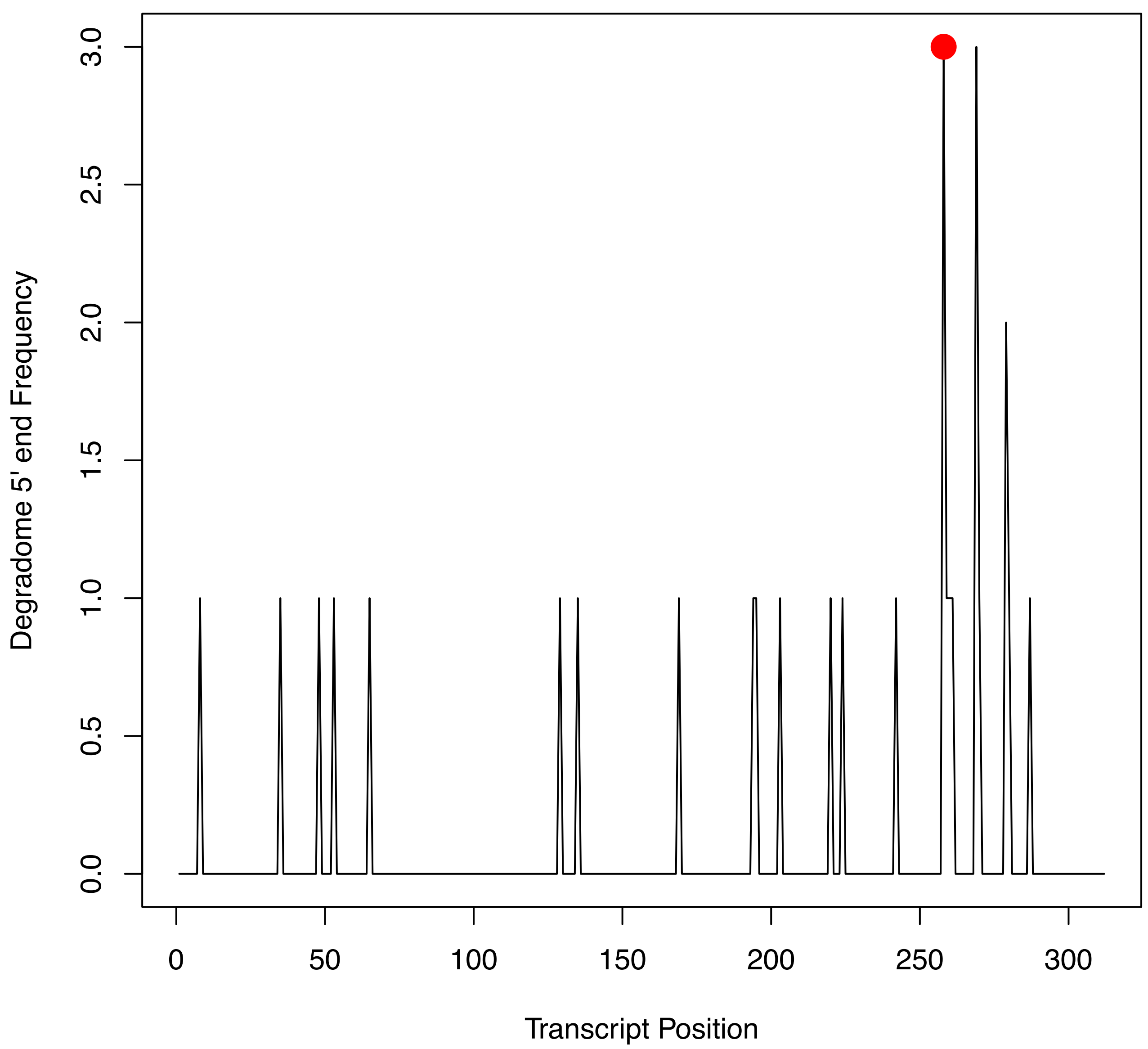

D=Dry
T=HORVU.MOREX.r3.7HG0649490.1
Q=miR166–5p.Cluster_3396
S=258
category=1
p=0.0119773167097942

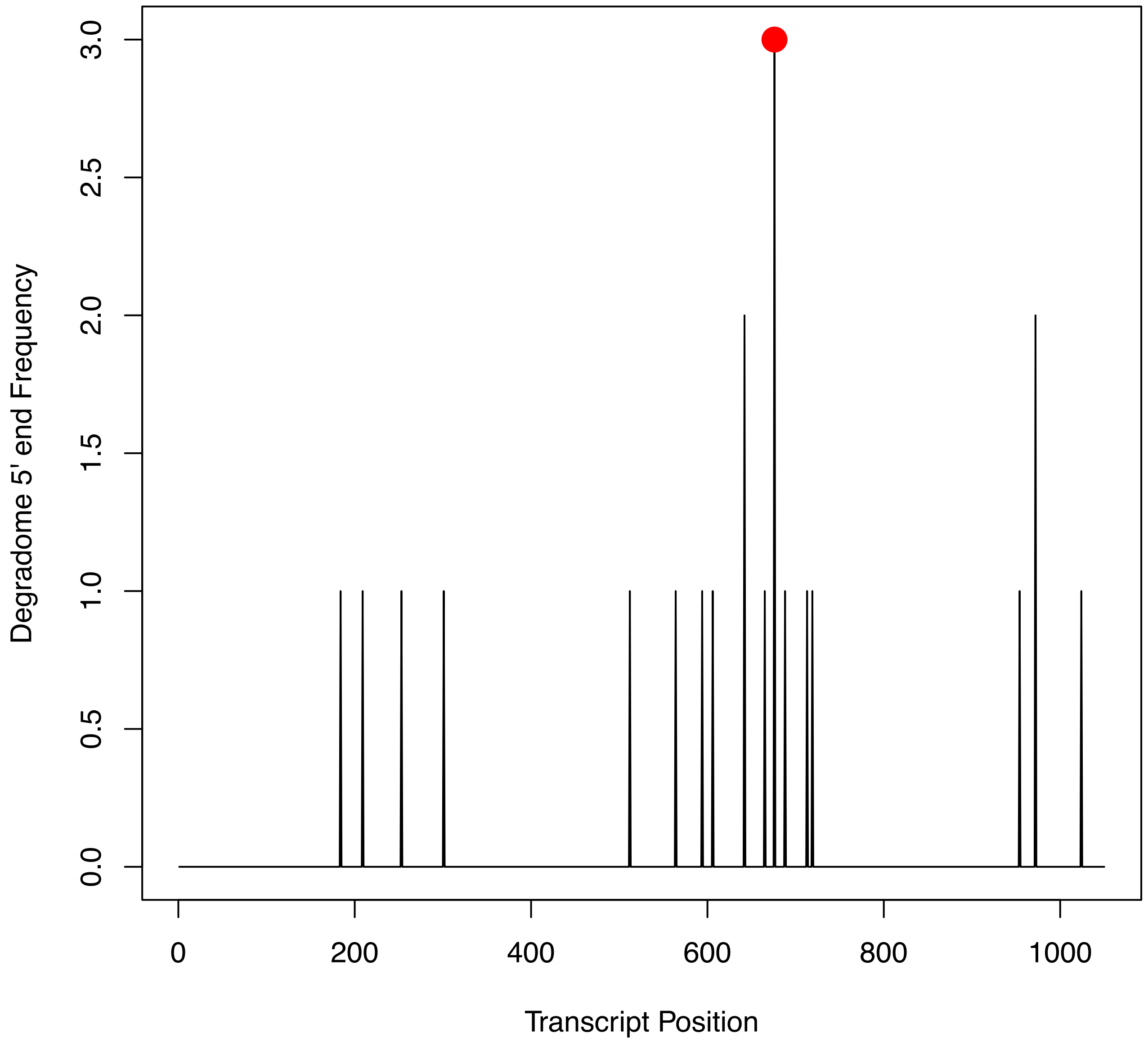

D=Dry
T=HORVU.MOREX.r3.7HG0698540.1
Q=miR167–5p.Cluster_3392.Cluster_3623
S=676
category=0
p=0.00846405779666615

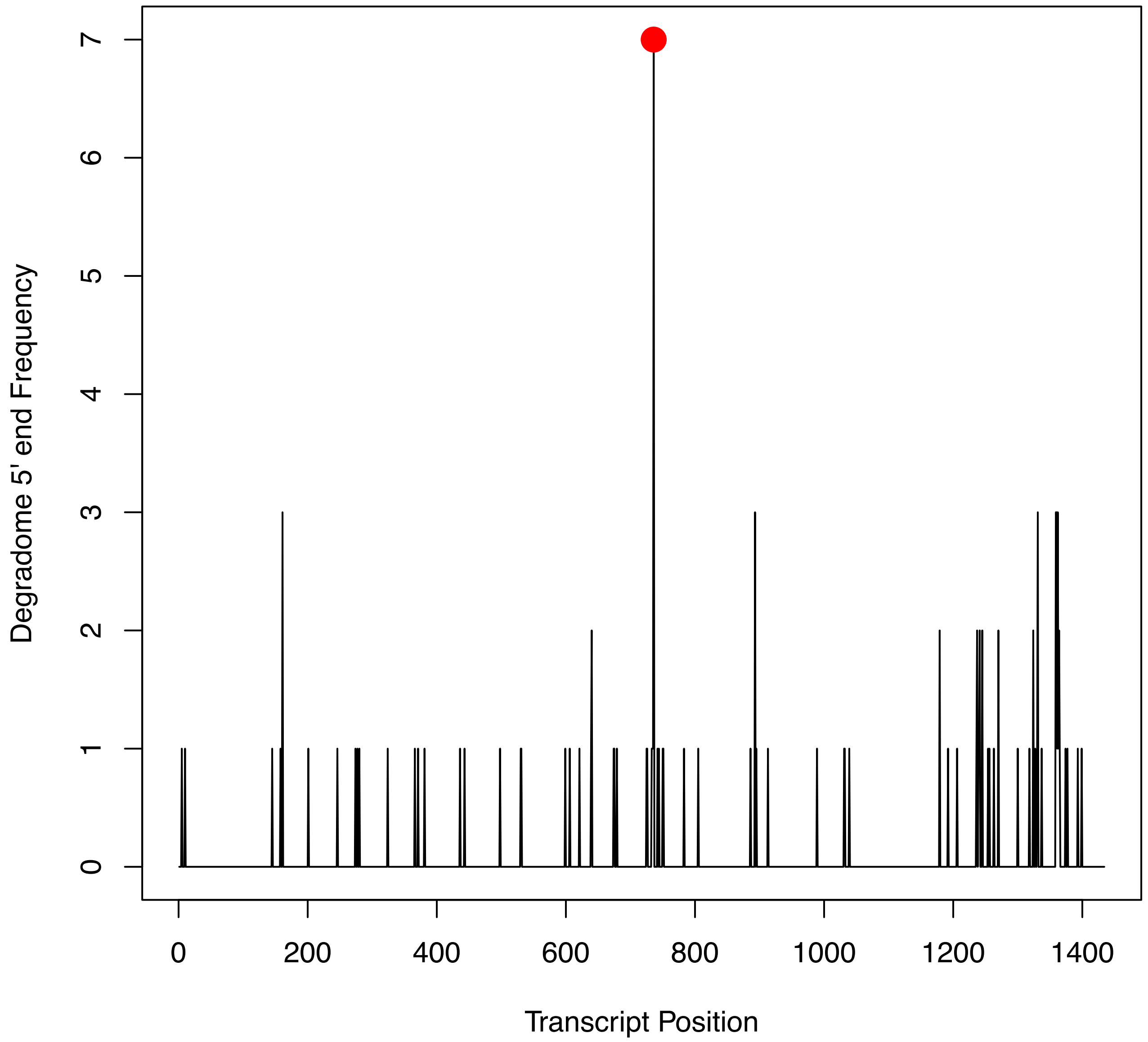

D=Dry
T=HORVU.MOREX.r3.2HG0116230.1
Q=miR167–5p.Cluster_4244
S=736
category=0
p=0.040631858329074 8

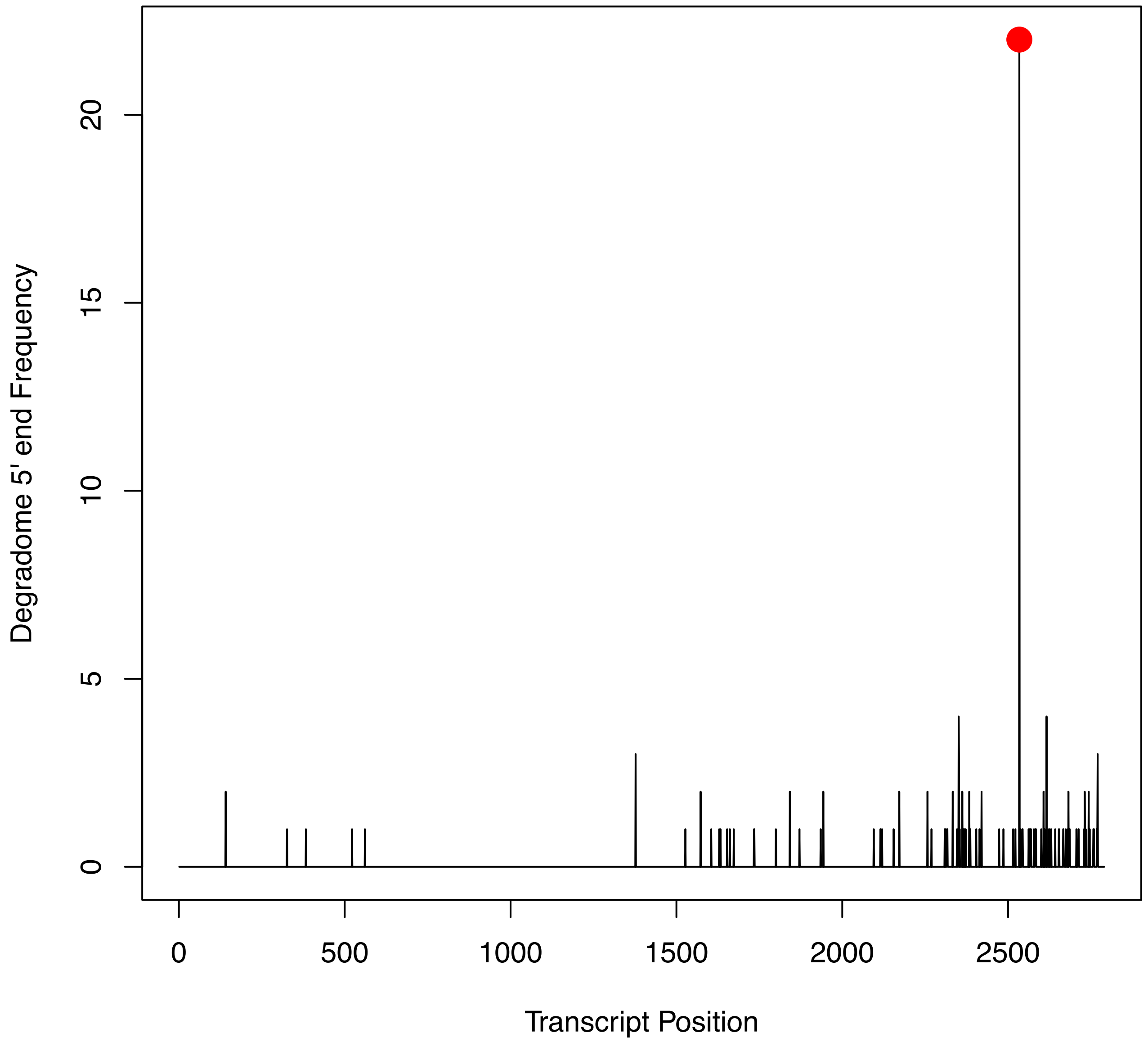

T=HORVU.MOREX.r3.6HG0566320.1
Q=miR167–5p.Cluster_4244
S=2534
category=0
p=0.00135908861711709

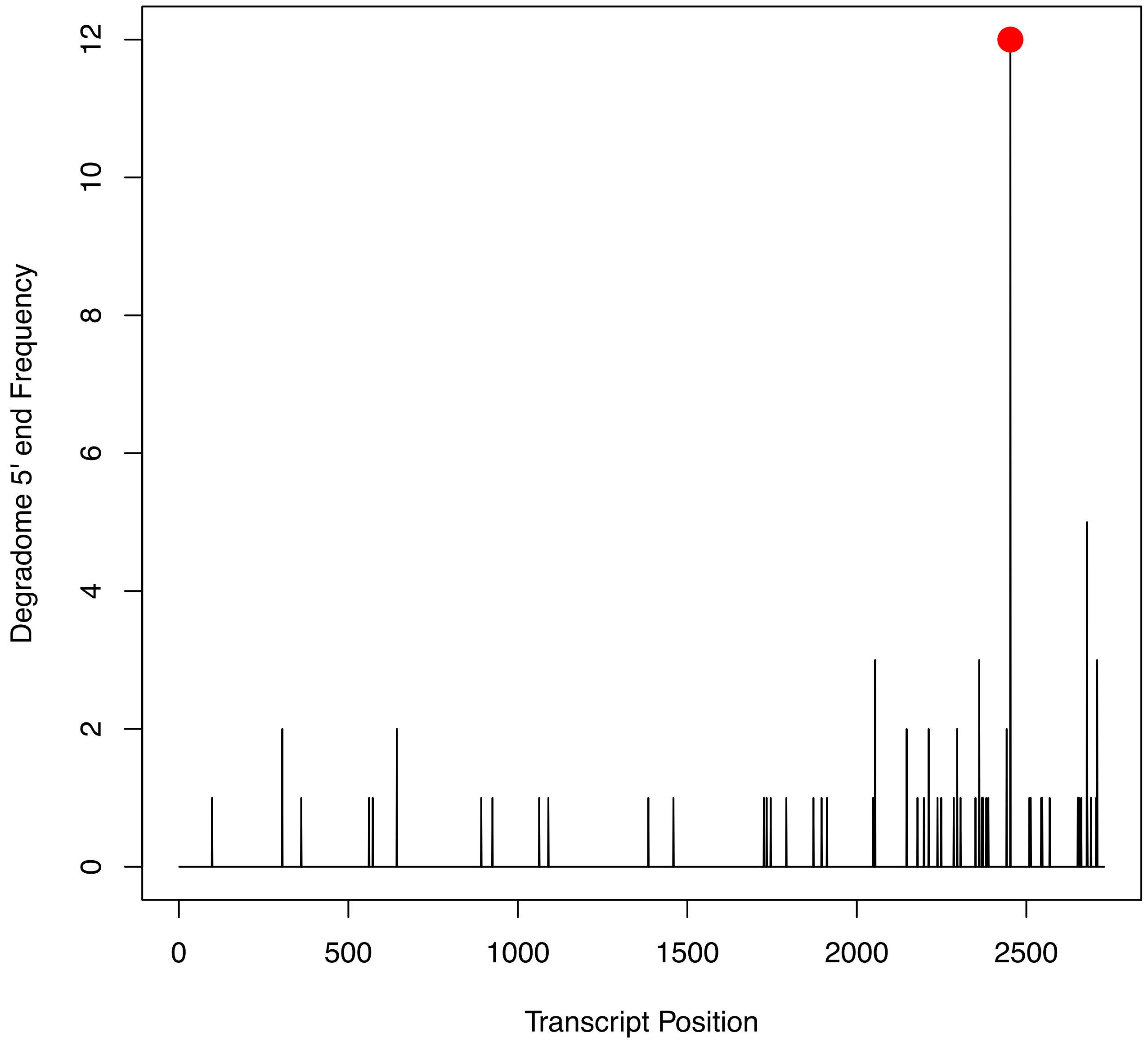

D=Dry
T=HORVU.MOREX.r3.7HG0735280.1
Q=miR167–5p.Cluster_4244
S=2453
category=0
p=0.00101948972865074

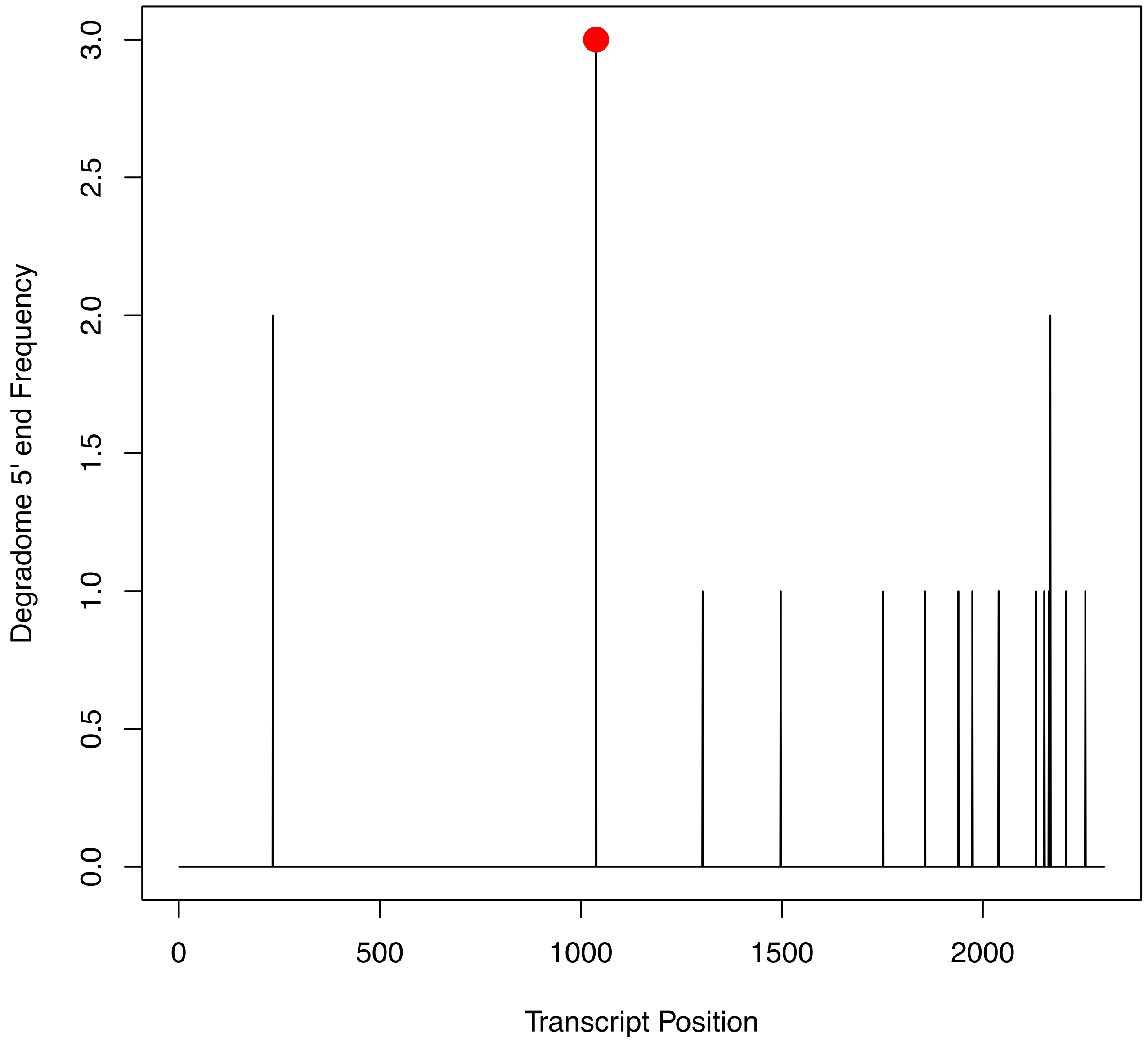

D=Dry
T=HORVU.MOREX.r3.4HG0415480.1
Q=miR171–3p.Cluster_3461
S=1038
category=0
p=0.00101948972865074

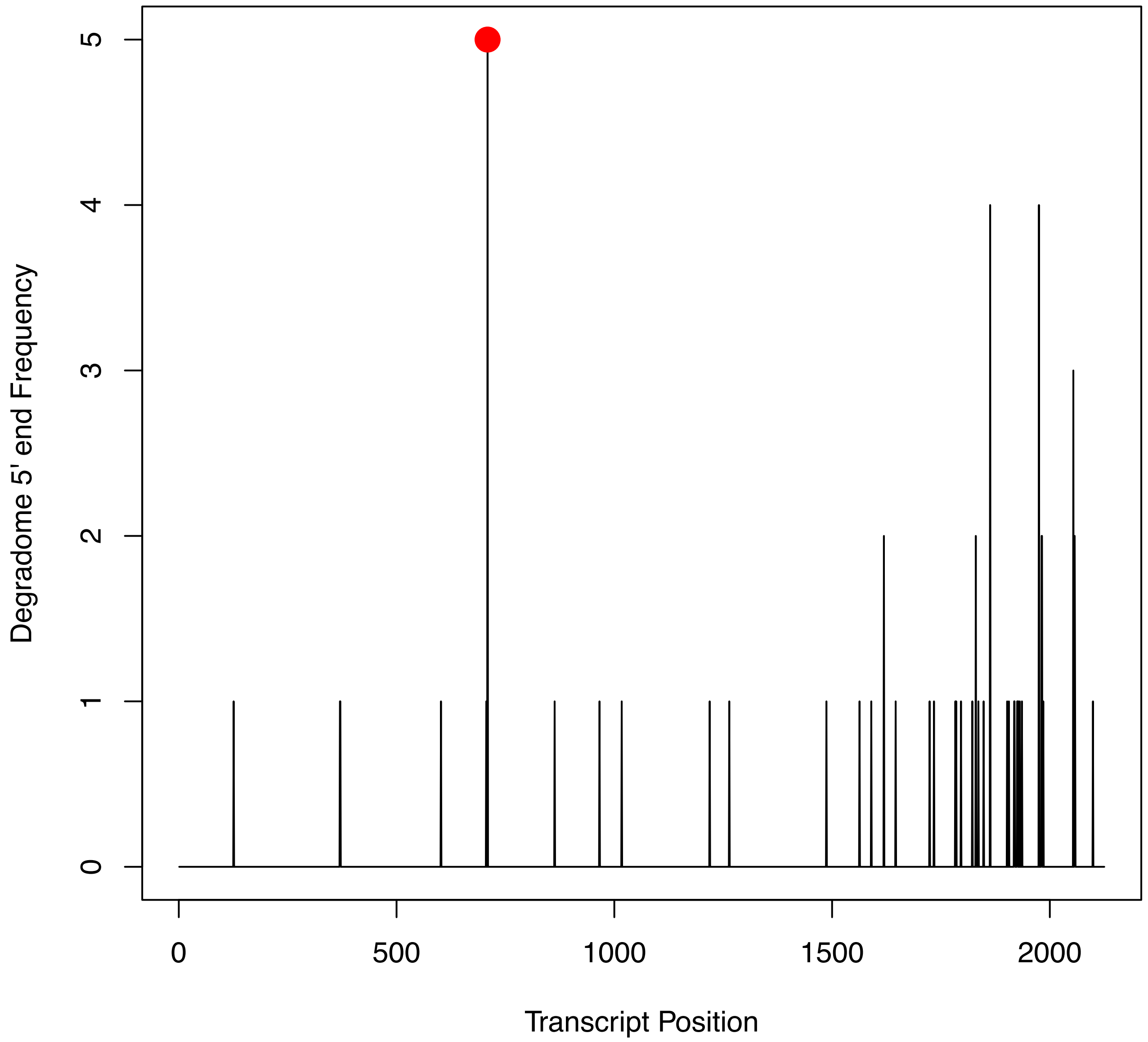

D=Dry
T=HORVU.MOREX.r3.7HG0635740.1
Q=miR171–3p.Cluster_3461
S=709
category=0
p=0.00033994545937055

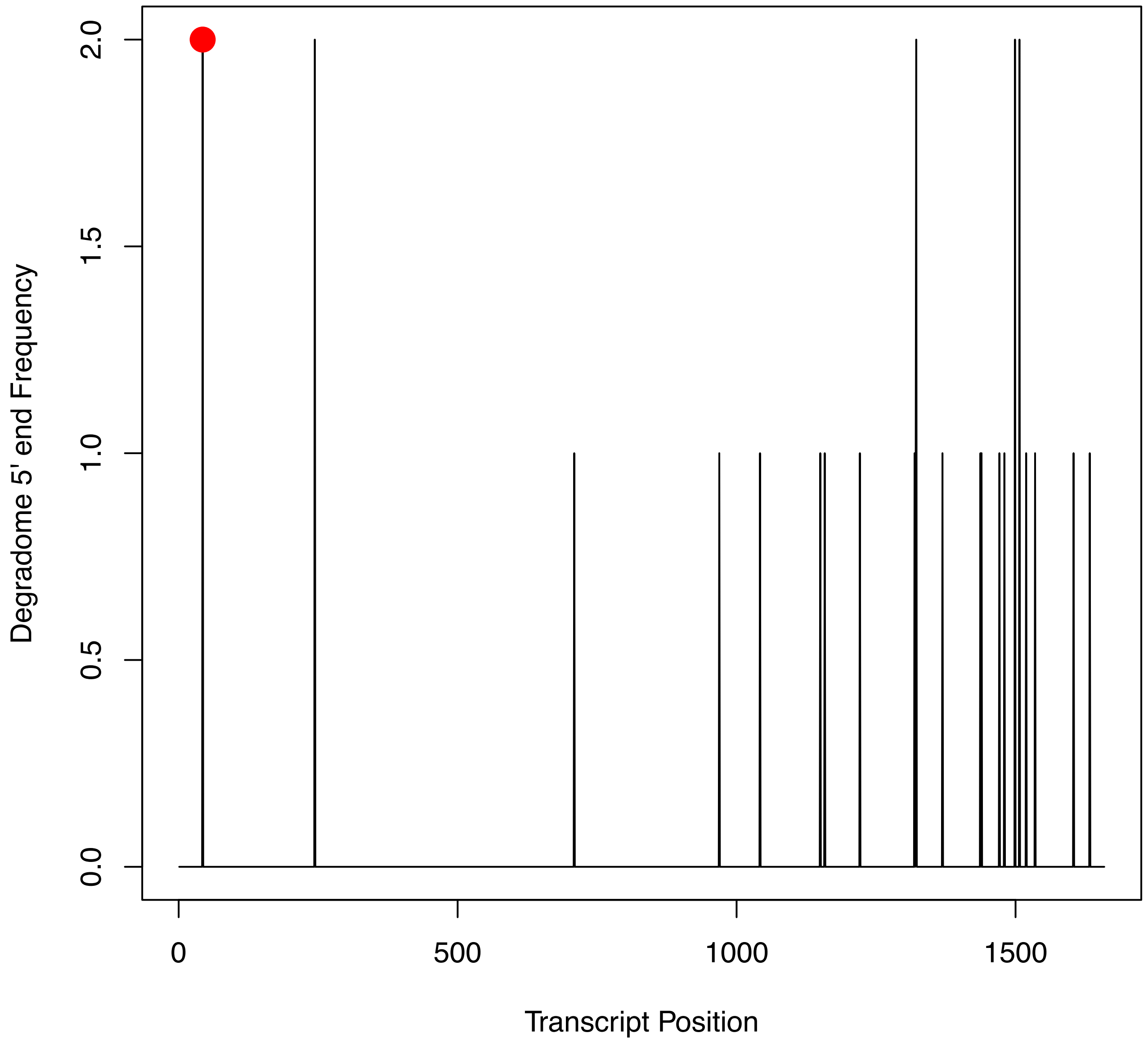

D=Dry
T=HORVU.MOREX.r3.2HG0142450.1
Q=miR319–3p.Cluster_2046
S=43
category=1
p=0.000509009398150795

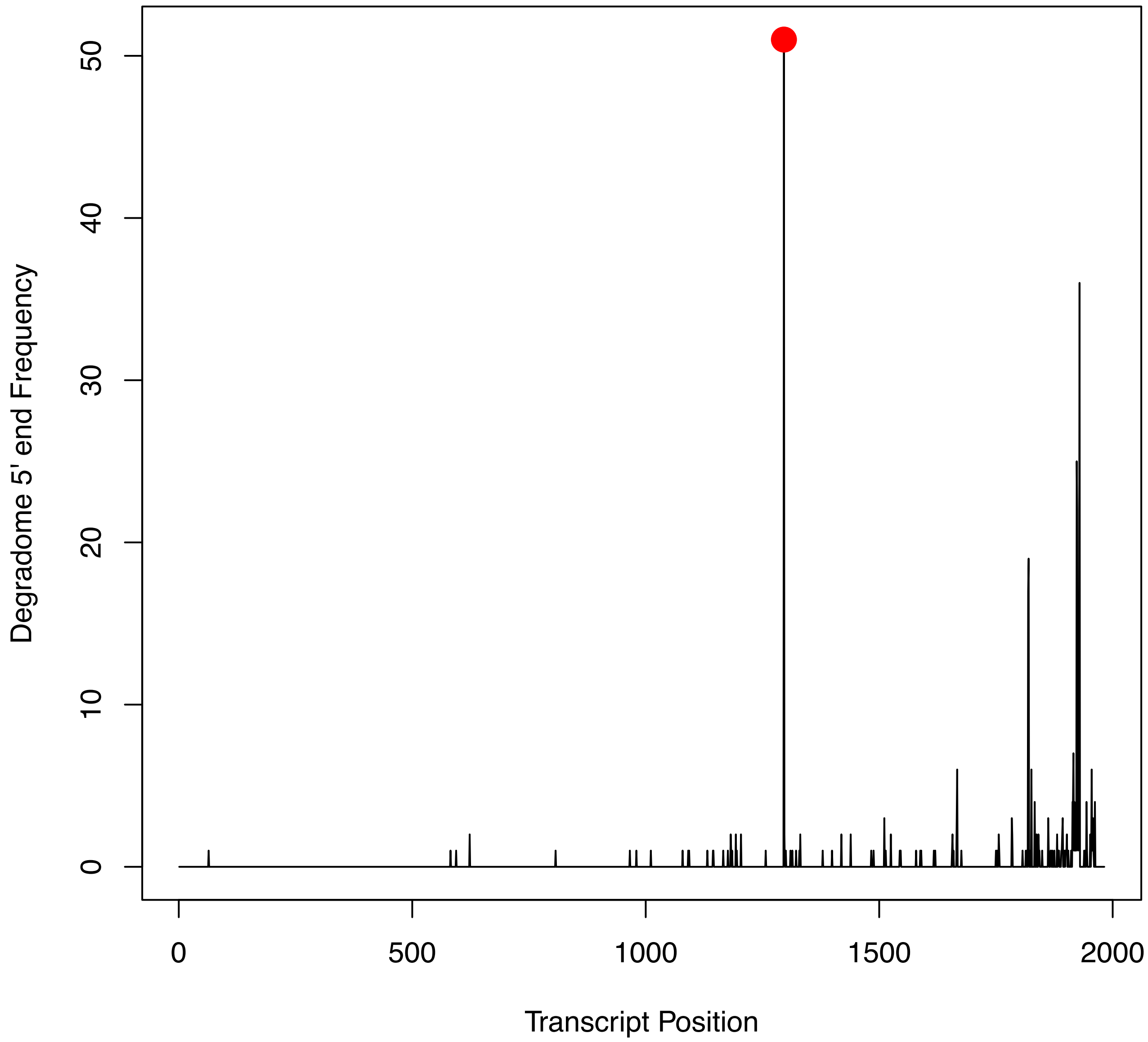

D=Dry
T=HORVU.MOREX.r3.2HG0152890.1
Q=miR319–3p.Cluster_2046
S=1296
category=0
p=0.000679775355825751

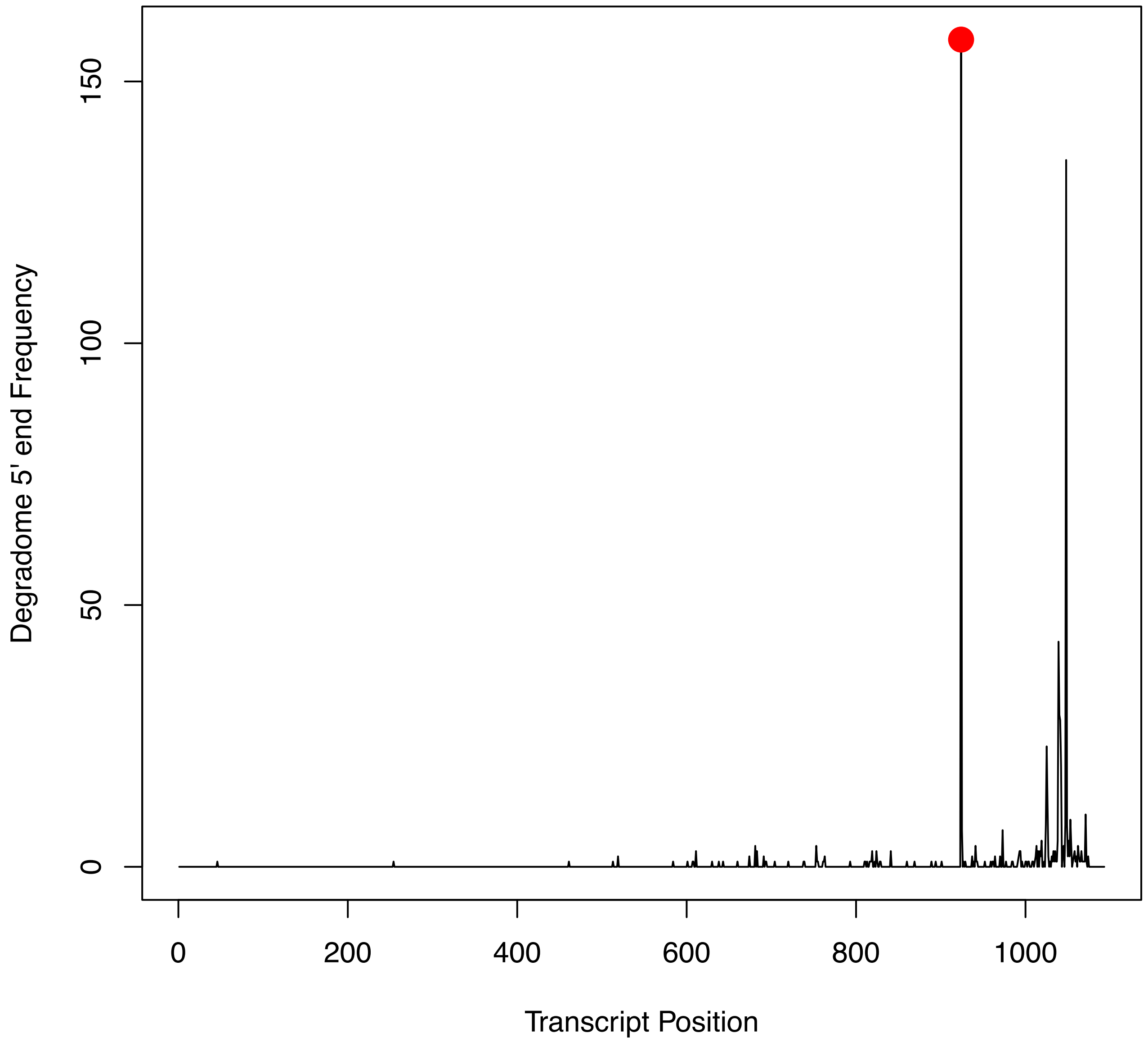

D=Dry
T=HORVU.MOREX.r3.3HG0243310.1
Q=miR319–3p.Cluster_2046
S=924
category=0
p=0.0151836938566232

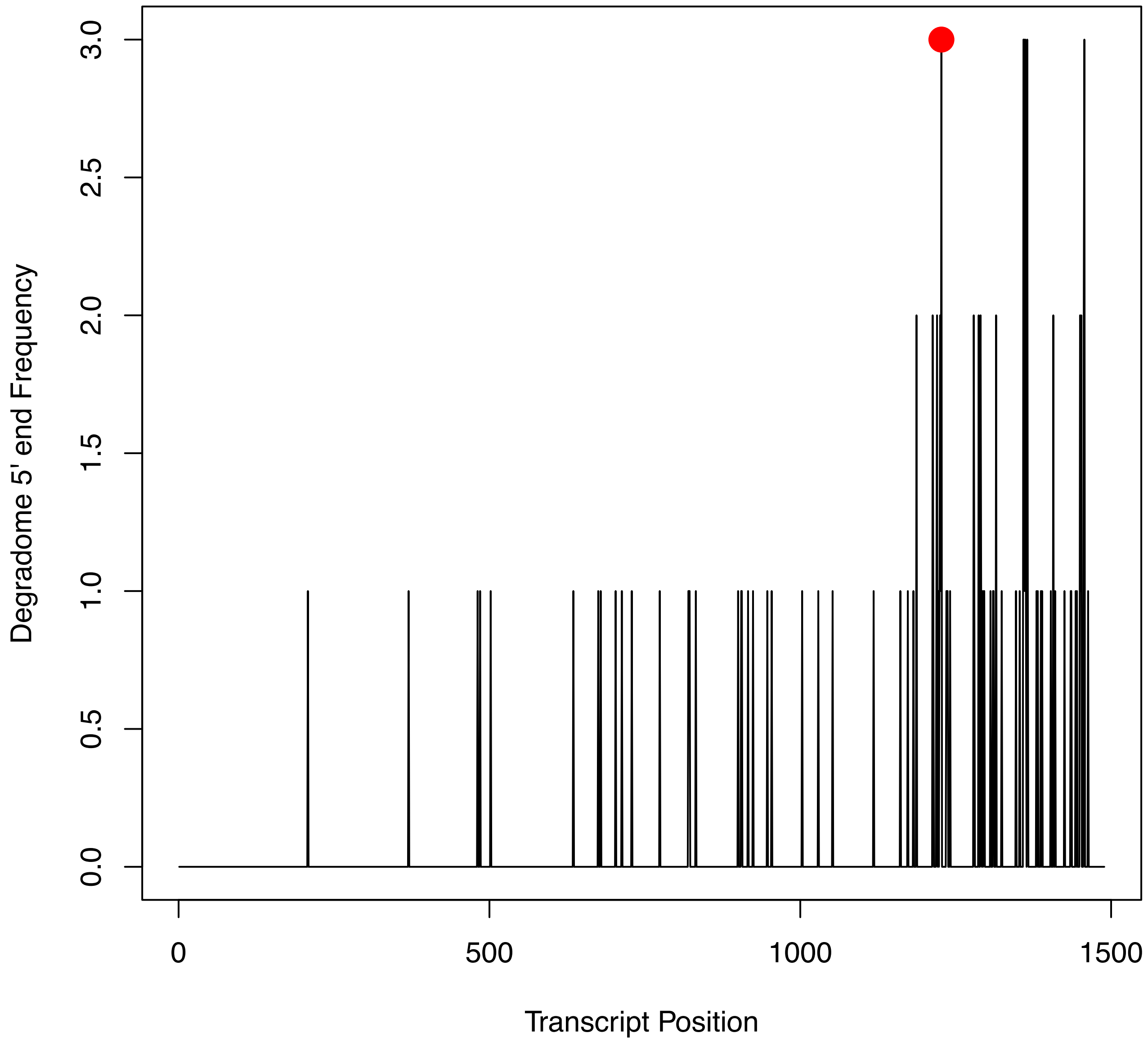

D=Dry
T=HORVU.MOREX.r3.1HG0017280.1
Q=miR396–3p.Cluster_1803
S=1227
category=1
p=0.0447885112850732

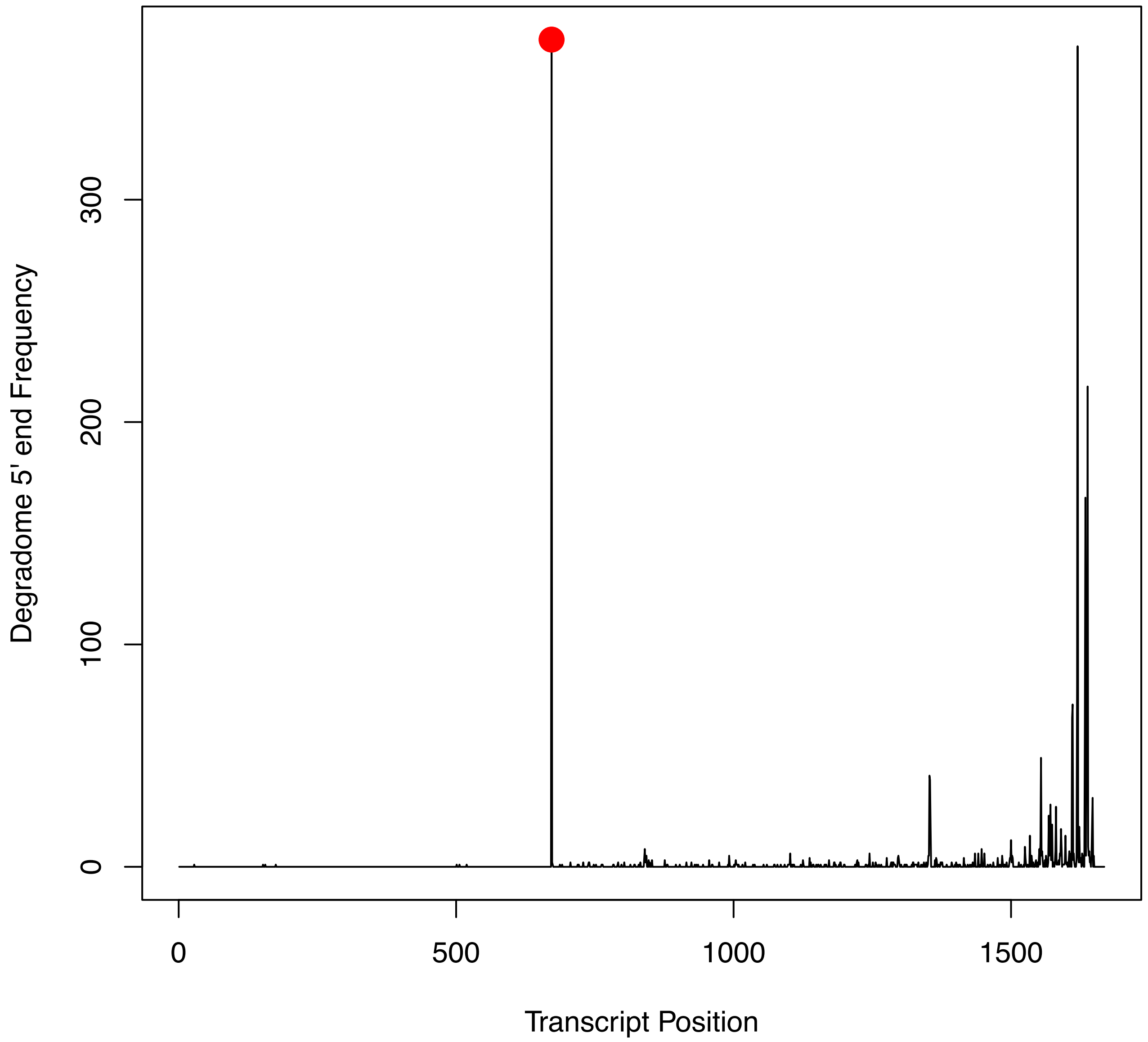

D=Dry
T=HORVU.MOREX.r3.2HG0193490.1
Q=miR396–5p.Cluster_1803
S=672
category=0
p=0.00033994545937055

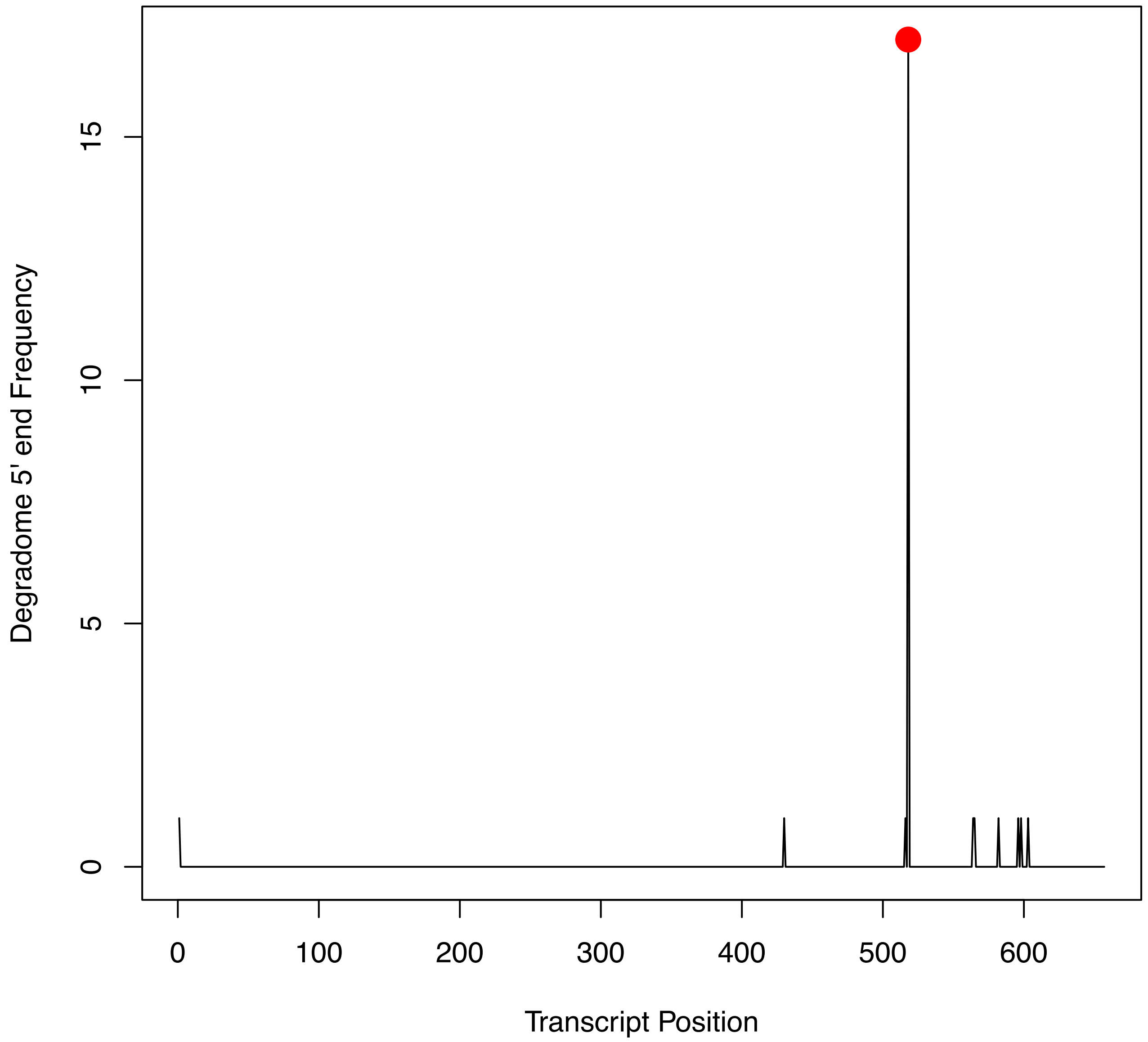

D=Dry
T=HORVU.MOREX.r3.6HG0603870.1
Q=miR396–5p.Cluster_1803
S=518
category=0
p=0.00271633011236516

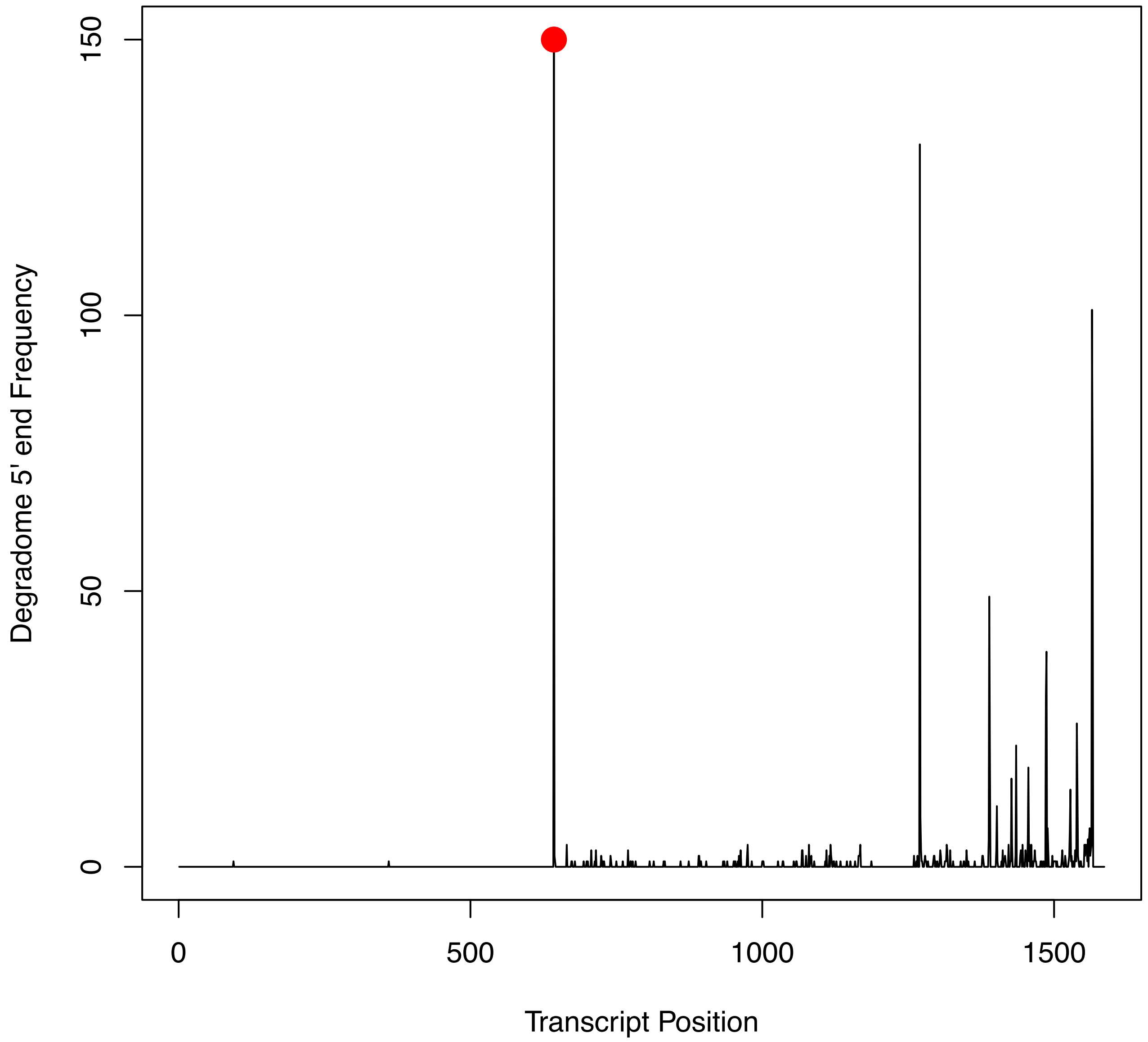

D=Dry
T=HORVU.MOREX.r3.7HG0641080.2
Q=miR396–5p.Cluster_1803
S=643
category=0
p=0.00305535216764785

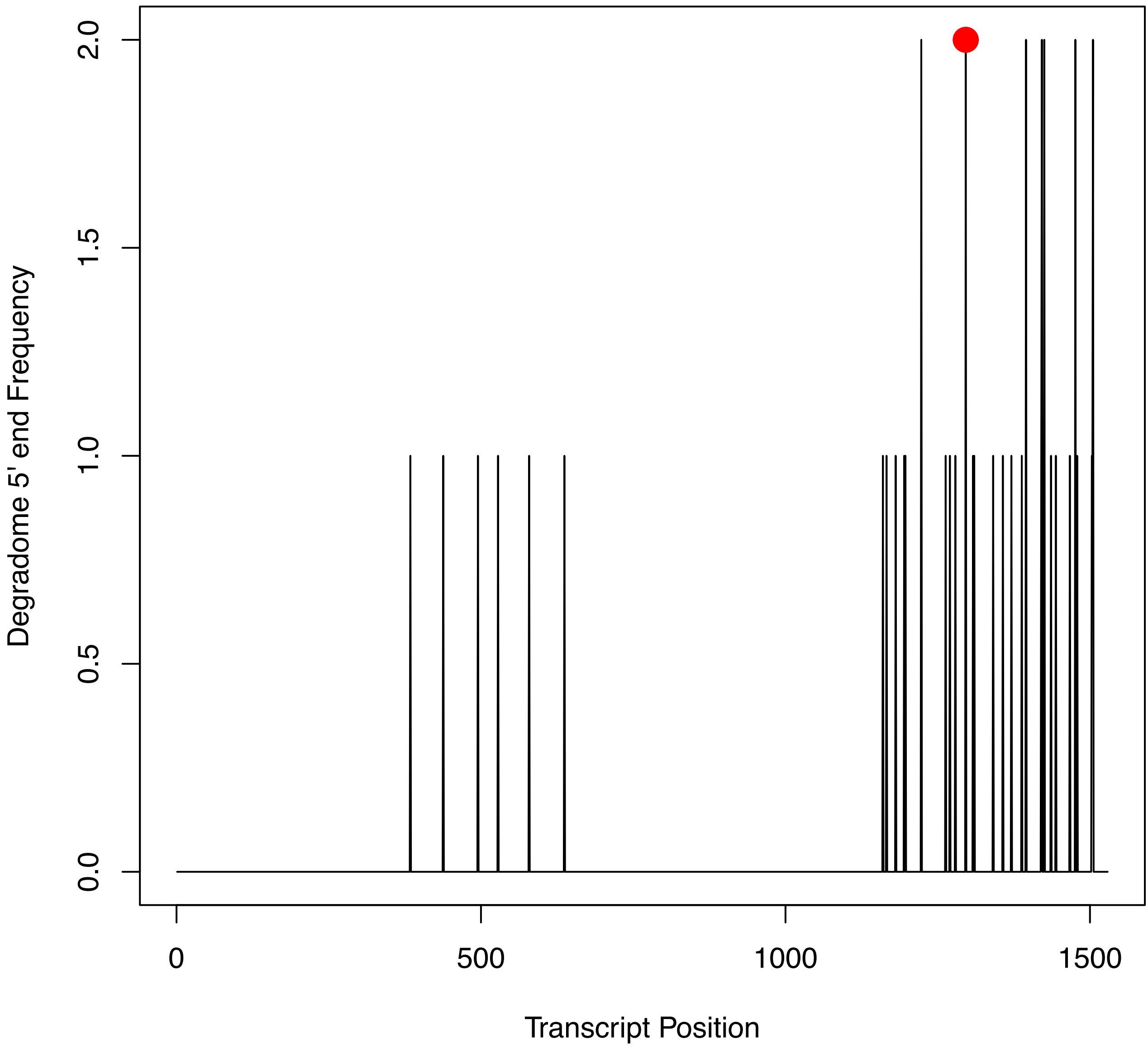

D=Dry
T=HORVU.MOREX.r3.7HG0642820.1
Q=miR396–5p.Cluster_1803
S=1296
category=1
p=0.0343568057536483

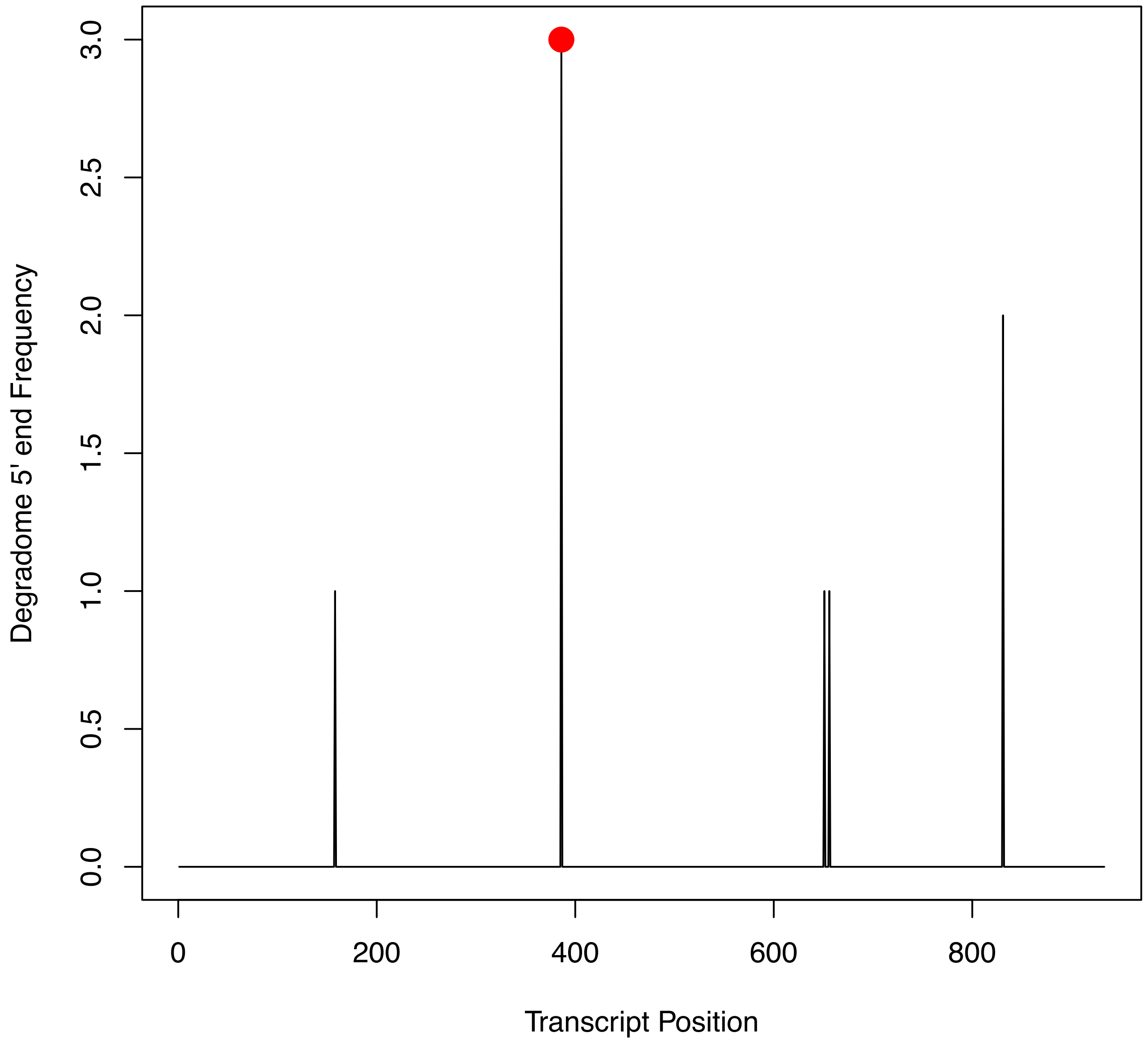

D=Dry
T=HORVU.MOREX.r3.7HG0662690.1
Q=miR396−5p.Cluster_1803
S=386
category=0
p=0.000679775355825751

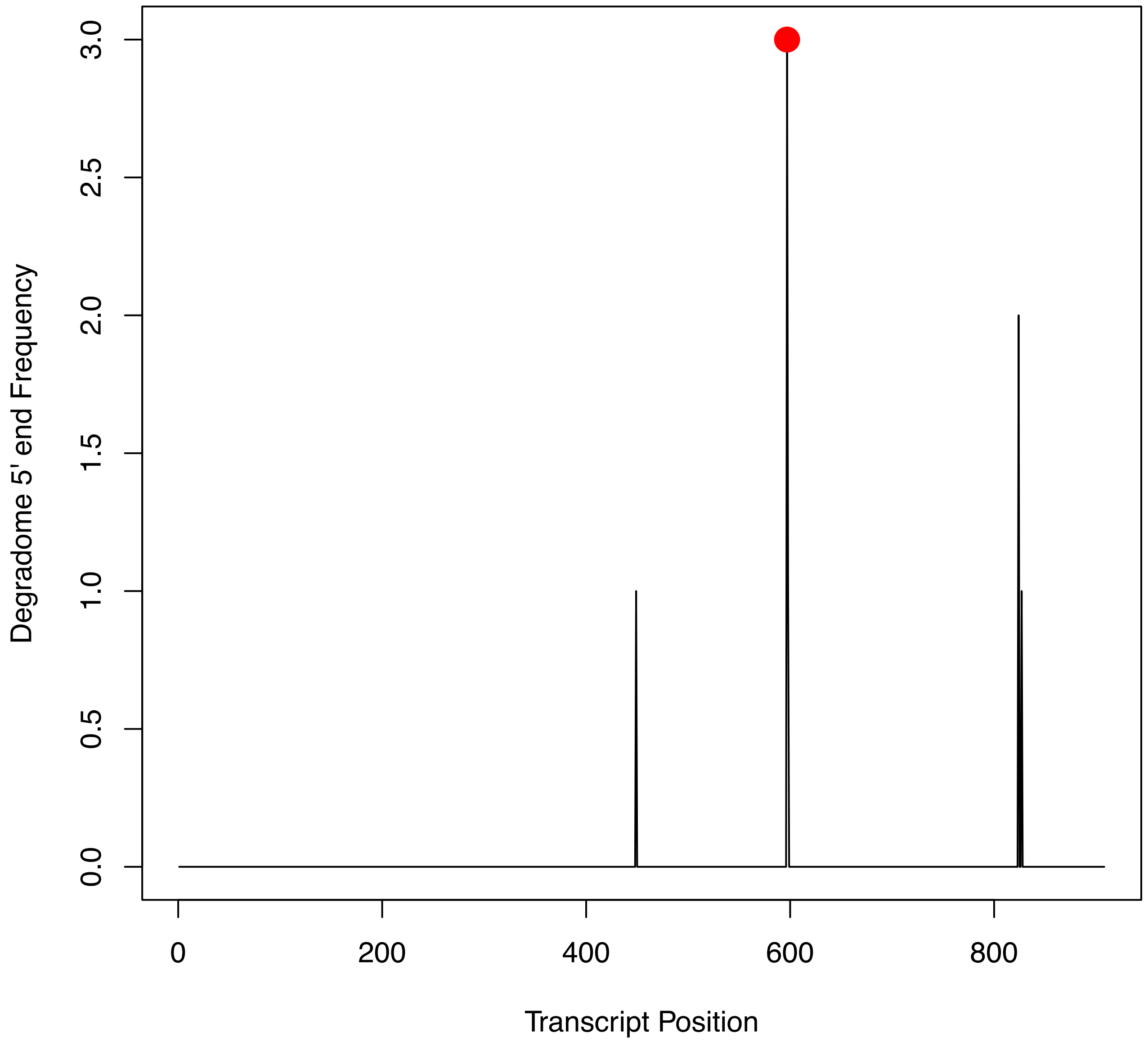

D=Dry
T=HORVU.MOREX.r3.2HG0186750.1
Q=miR396–5p.Cluster_5480
S=597
category=0
p=0.00135908861711709

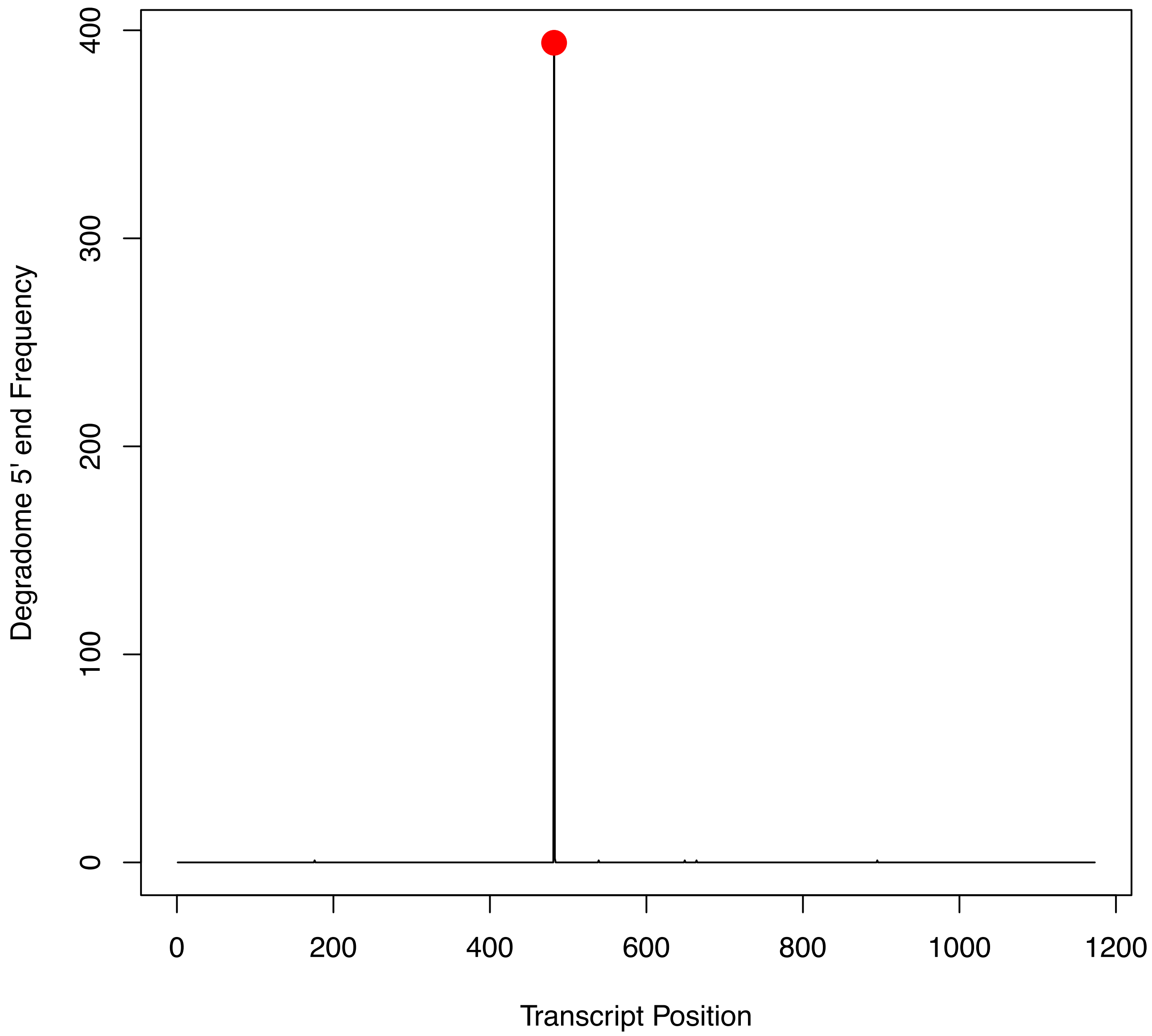

D=Dry
T=HORVU.MOREX.r3.6HG0606810.1
Q=miR396–5p.Cluster_5480
S=482
category=0
p=0.00237719276888249

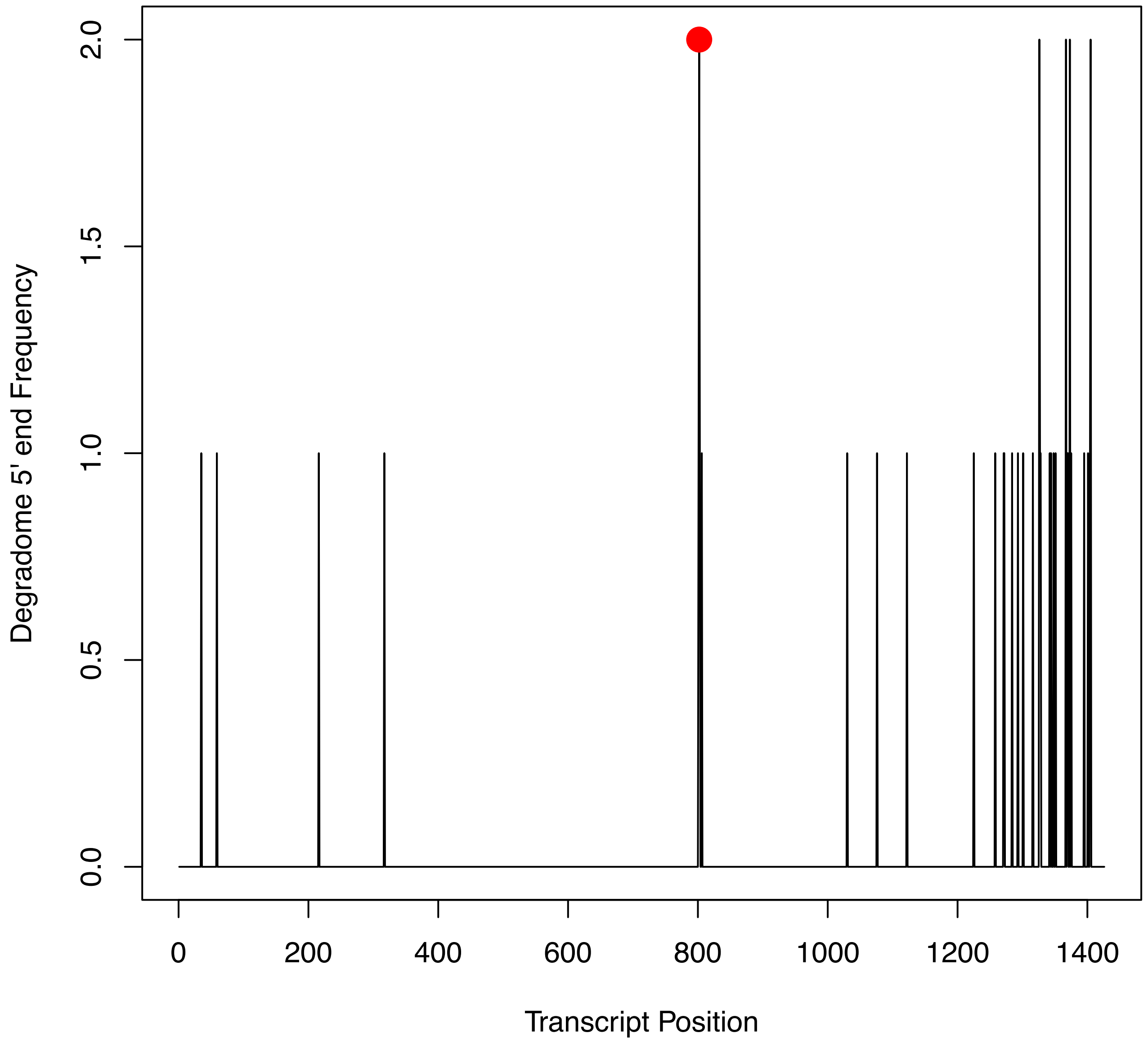

D=Dry
T=HORVU.MOREX.r3.6HG0541280.1
Q=miR9662–3p.Cluster_4660
S=802
category=1
p=0.0143220541328917

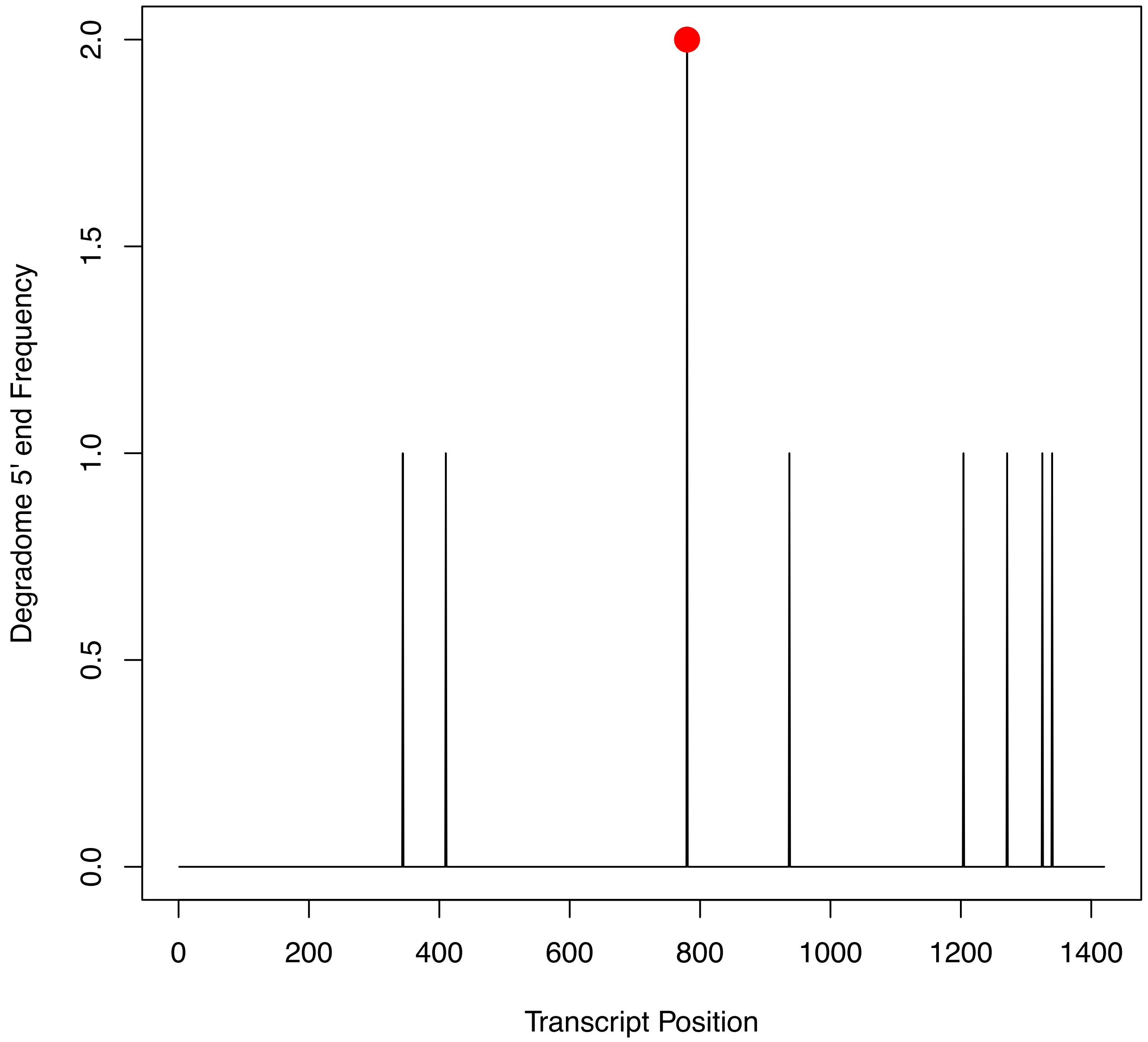

D=Dry
T=HORVU.MOREX.r3.6HG0541690.1
Q=miR9662–3p.Cluster_4660
S=780
category=0
p=0.00169857206048341

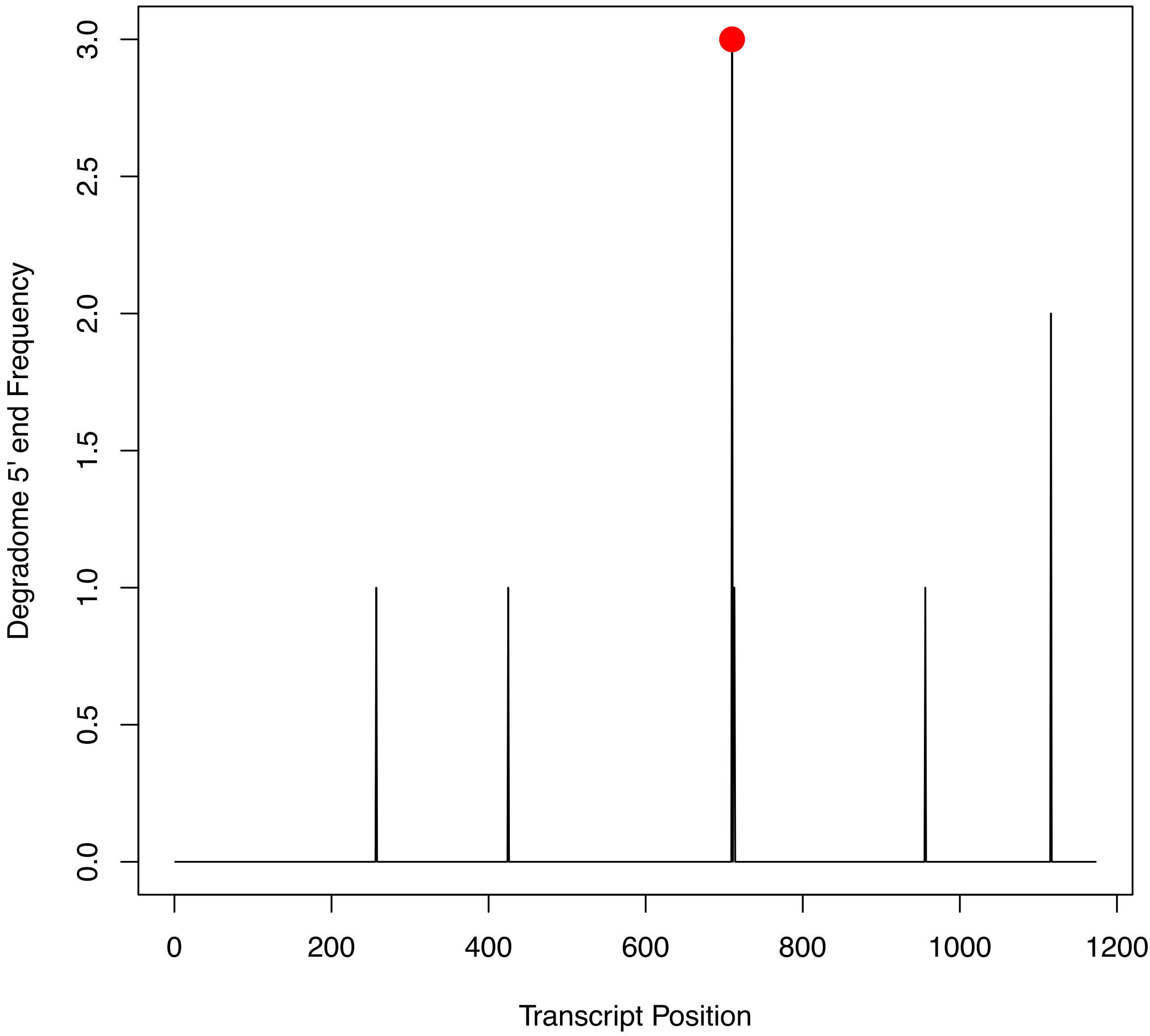

D=Dry
T=HORVU.MOREX.r3.6HG0543320.1
Q=miR9662–3p.Cluster_4660
S=710
category=0
p=0.0155184776882108

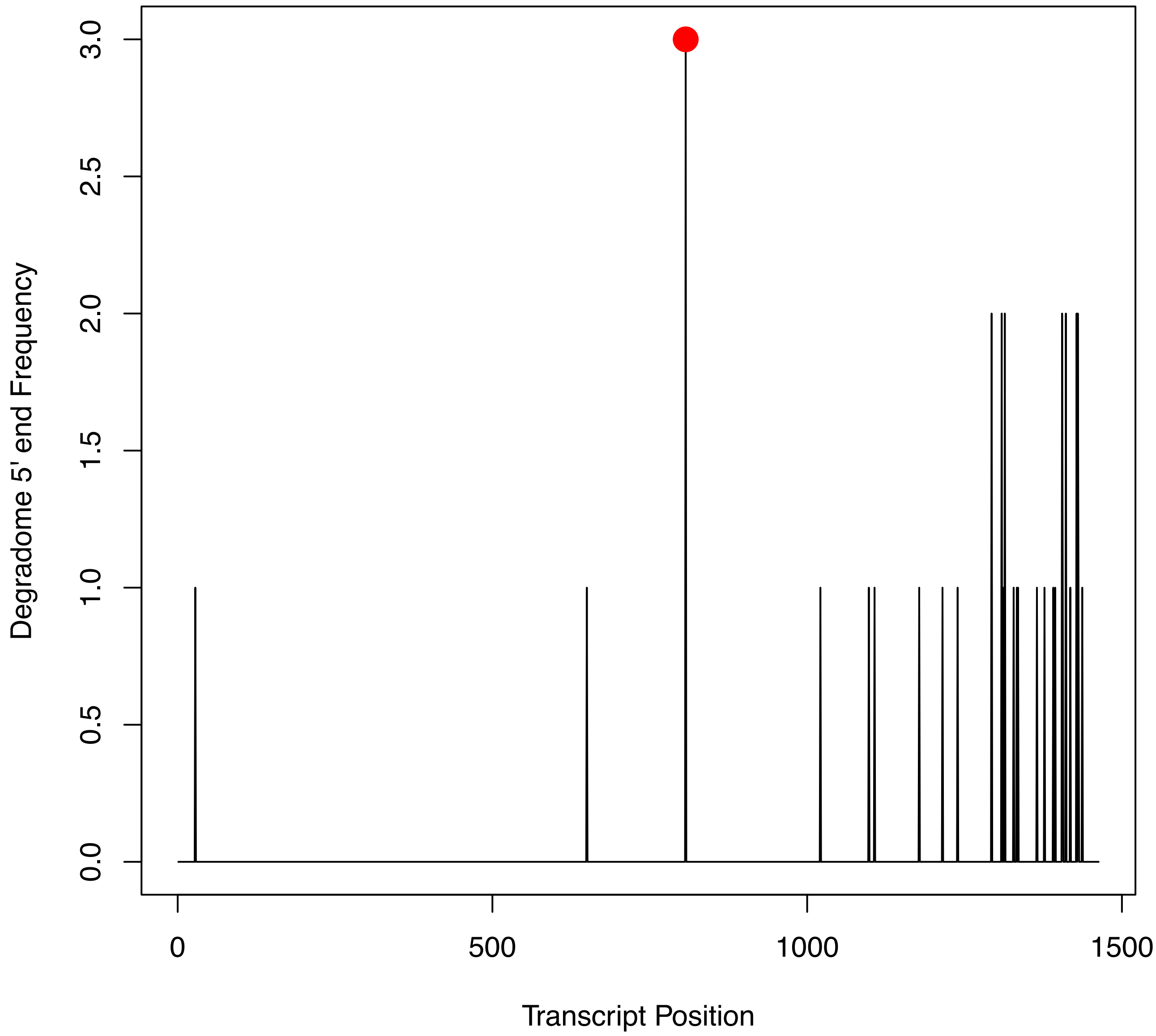

D=Dry
T=HORVU.MOREX.r3.6HG0543350.1
Q=miR9662−3p.Cluster_4660
S=807
category=0
p=0.0141786591253168

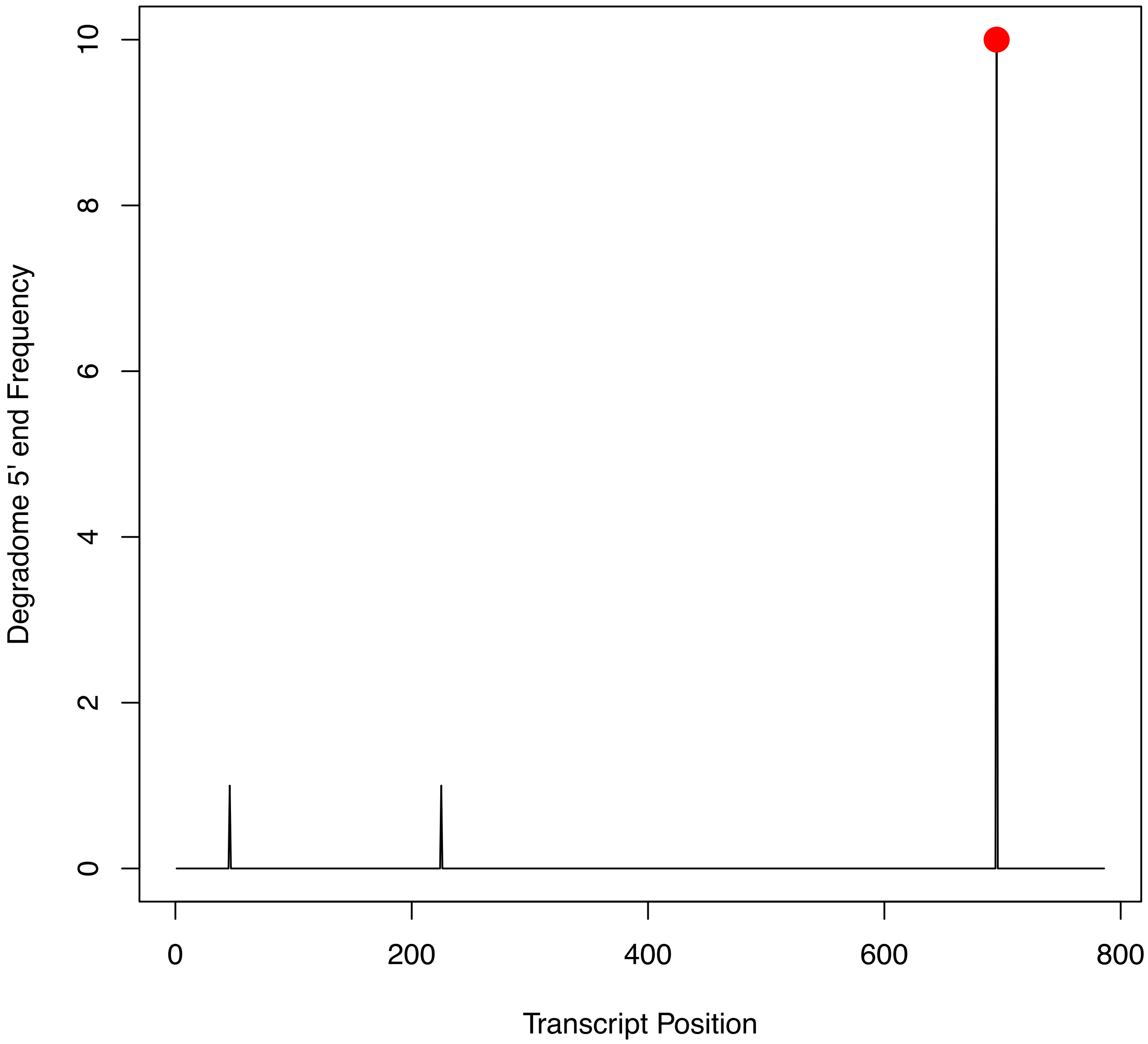

D=Dry
T=HORVU.MOREX.r3.6HG0543460.1
Q=miR9662–3p.Cluster_4660
S=695
category=0
p=0.000679775355825751

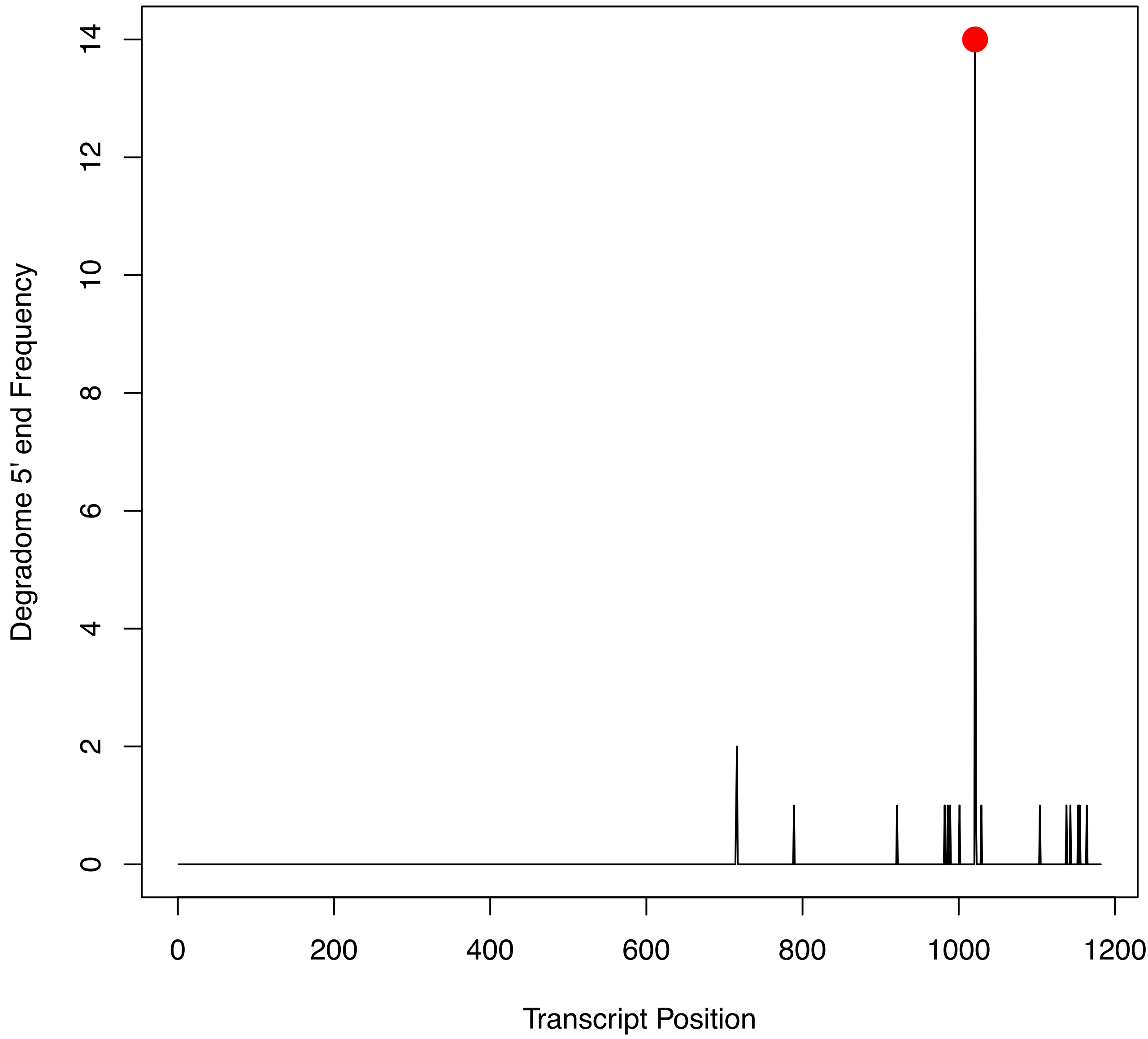

D=Dry
T=HORVU.MOREX.r3.5HG0534390.1
Q=novel–5p.Cluster_68
S=1021
category=0
p=0.0288169192839769

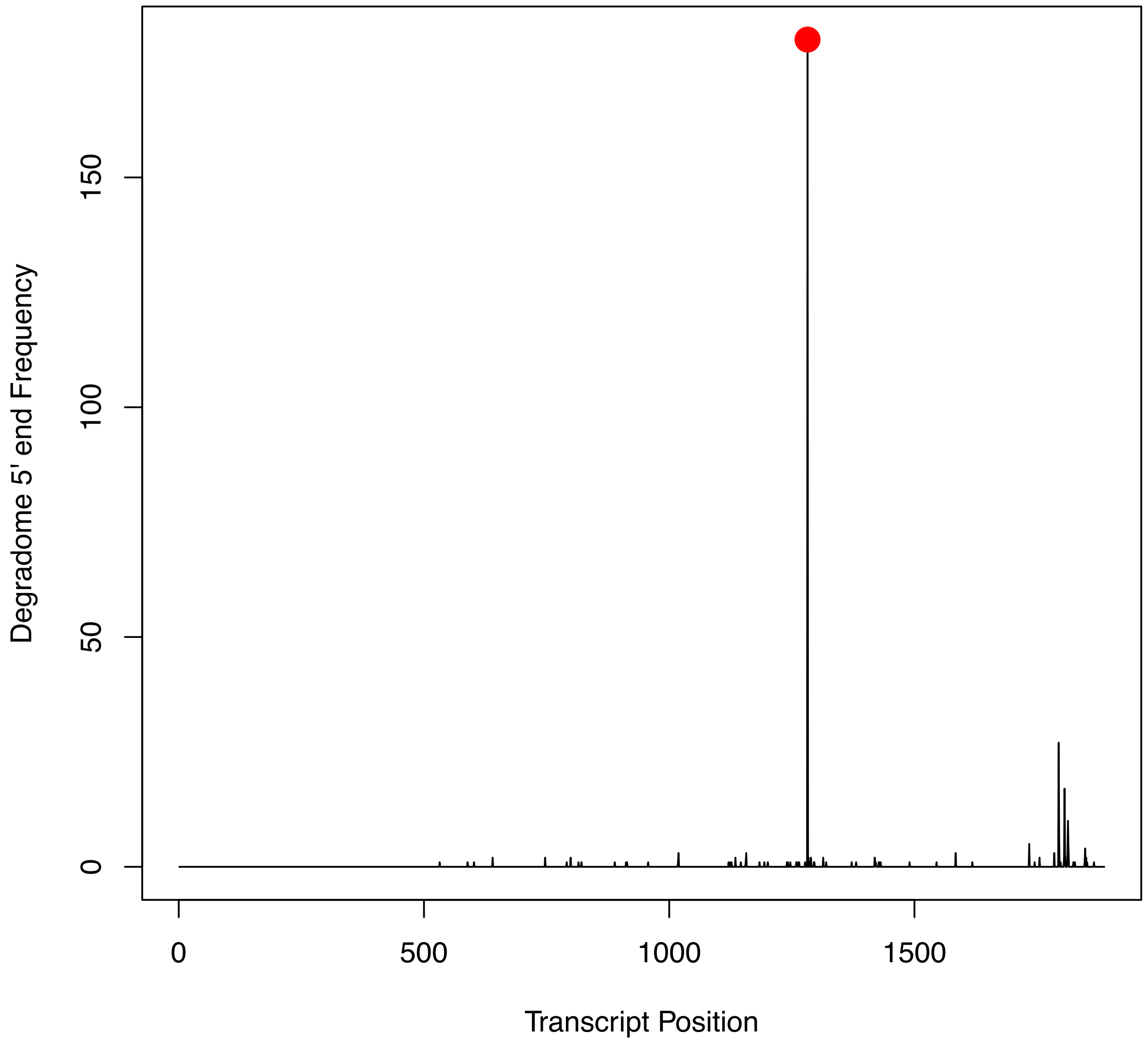

D=Day0
T=HORVU.MOREX.r3.3HG0310780.1
Q=miR156–5p.Cluster_1557.Cluster_1973.Cluster_4963
S=1282
category=0
p=0.00163601204878971

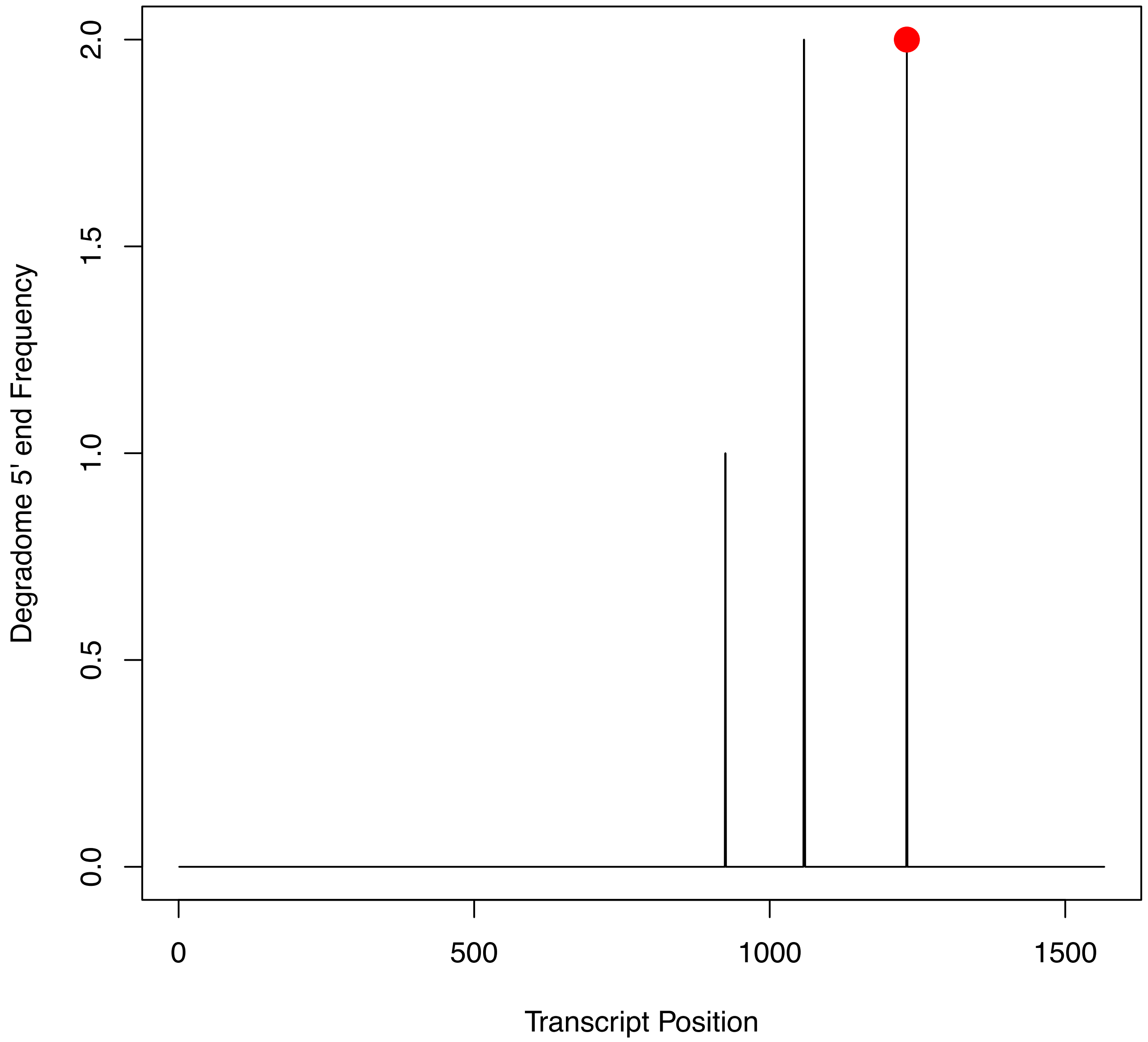

D=Day0
T=HORVU.MOREX.r3.4HG0408490.1
Q=miR156–5p.Cluster_1557.Cluster_1973.Cluster_4963
S=1232
category=1
p=0.0300167421341063

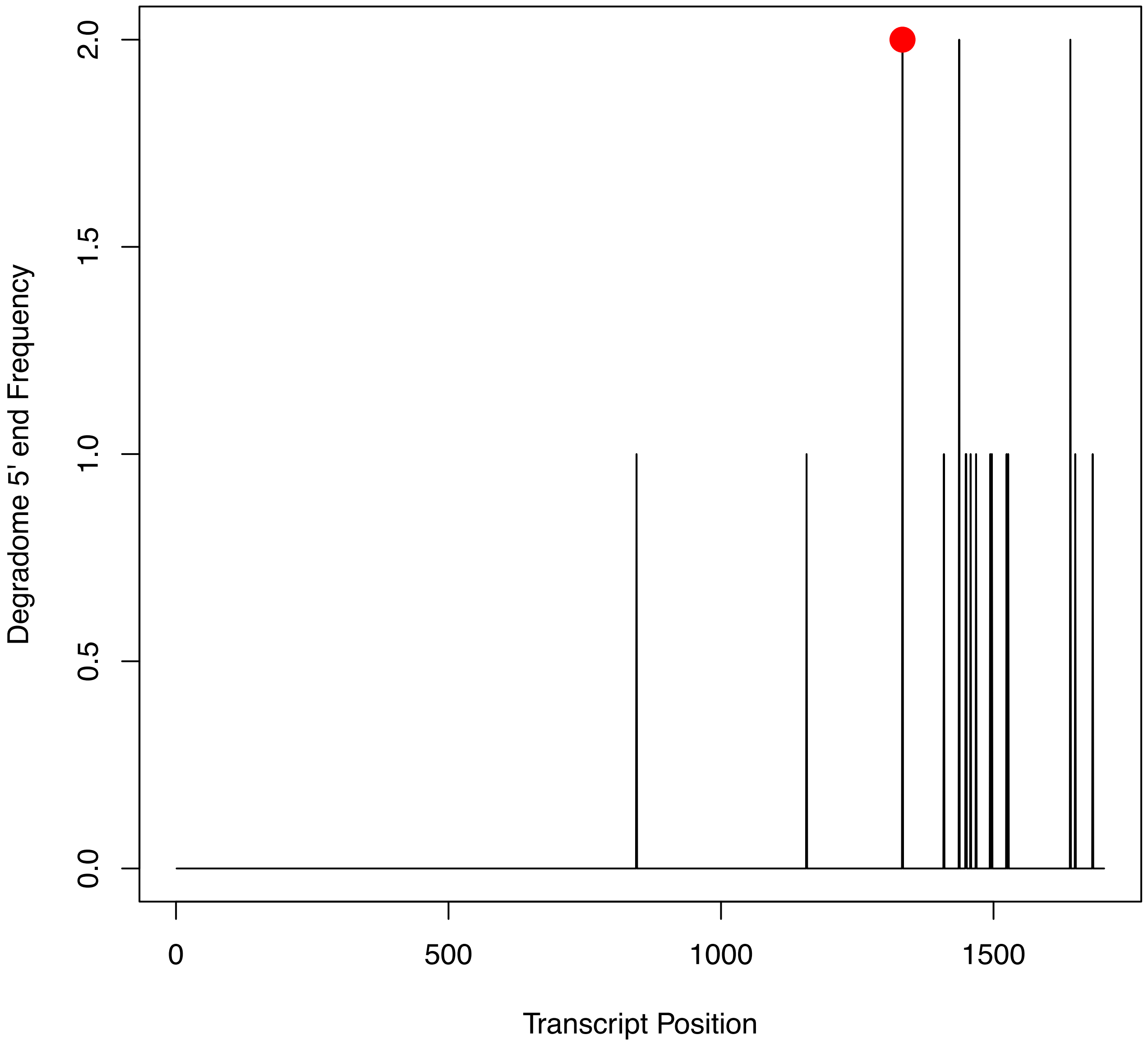

D=Day0
T=HORVU.MOREX.r3.4HG0410750.1
Q=miR156–5p.Cluster_1557.Cluster_1973.Cluster_4963
S=1333
category=1
p=0.00238745666270268

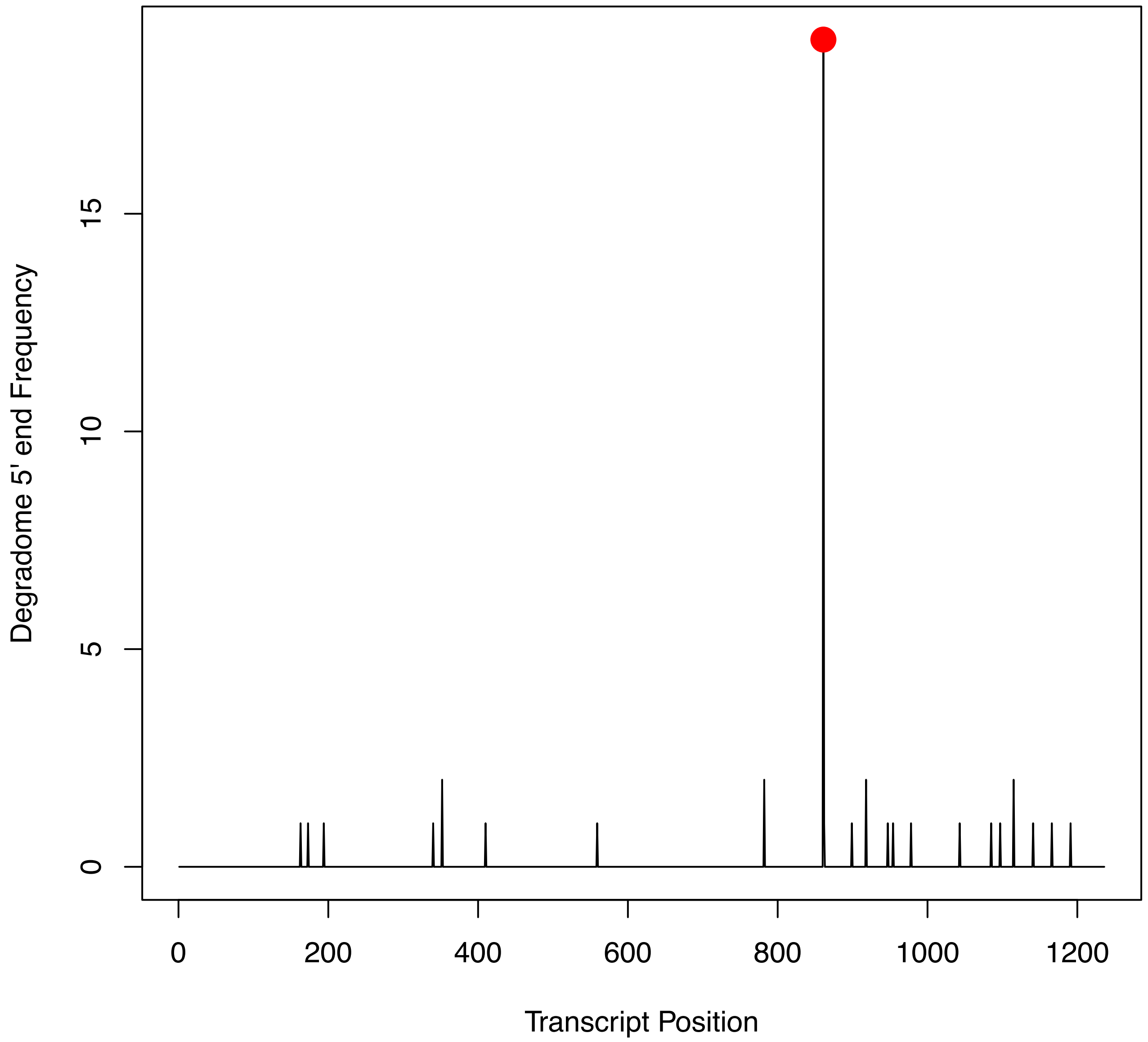

D=Day0
T=HORVU.MOREX.r3.5HG0490900.1
Q=miR156–5p.Cluster_1557.Cluster_1973.Cluster_4963
S=861
category=0
p=0.000818340865280653

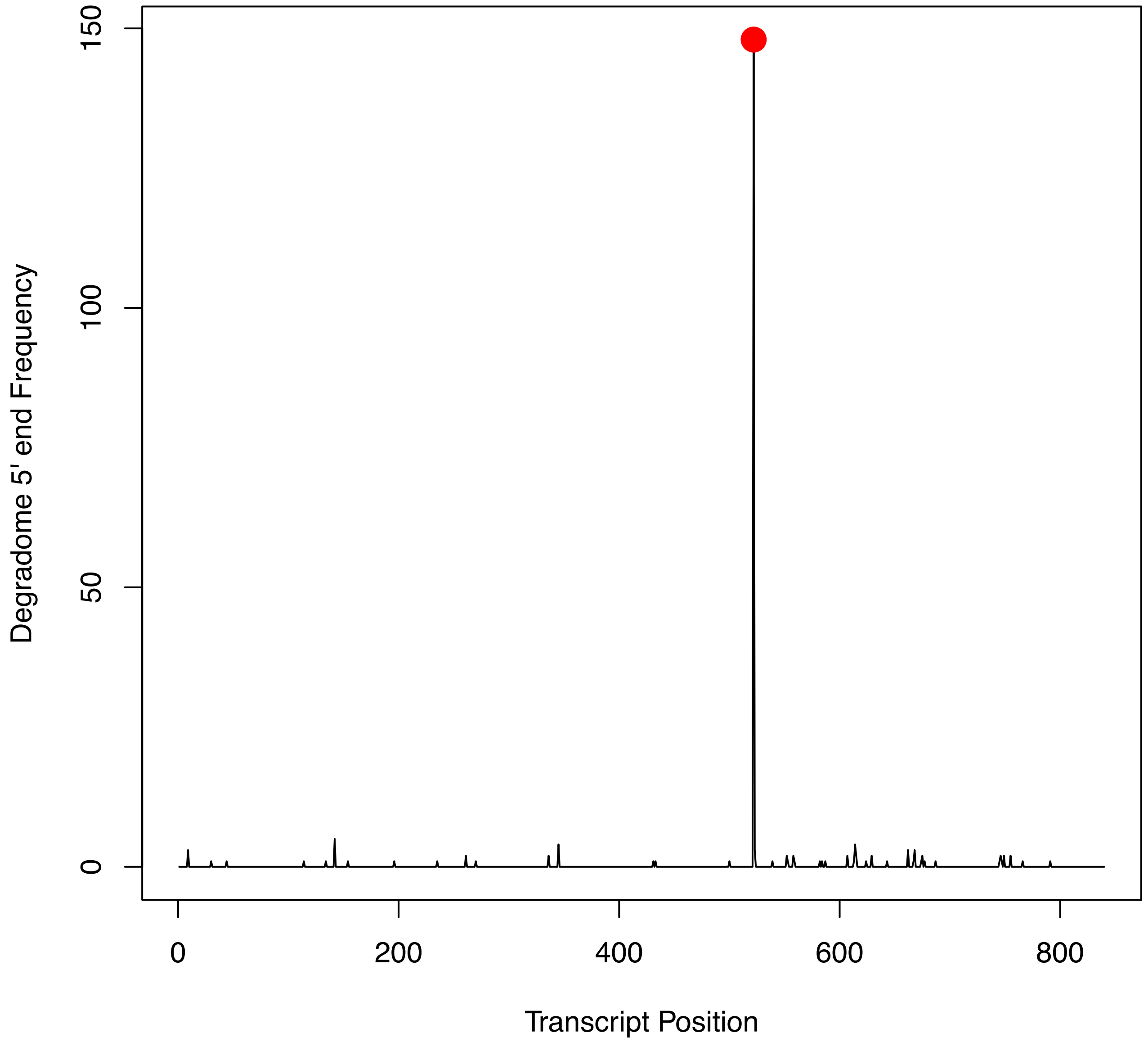

D=Day0
T=HORVU.MOREX.r3.5HG0494180.1
Q=miR156–5p.Cluster_1557.Cluster_1973.Cluster_4963
S=522
category=0
p=0.00286126436941947

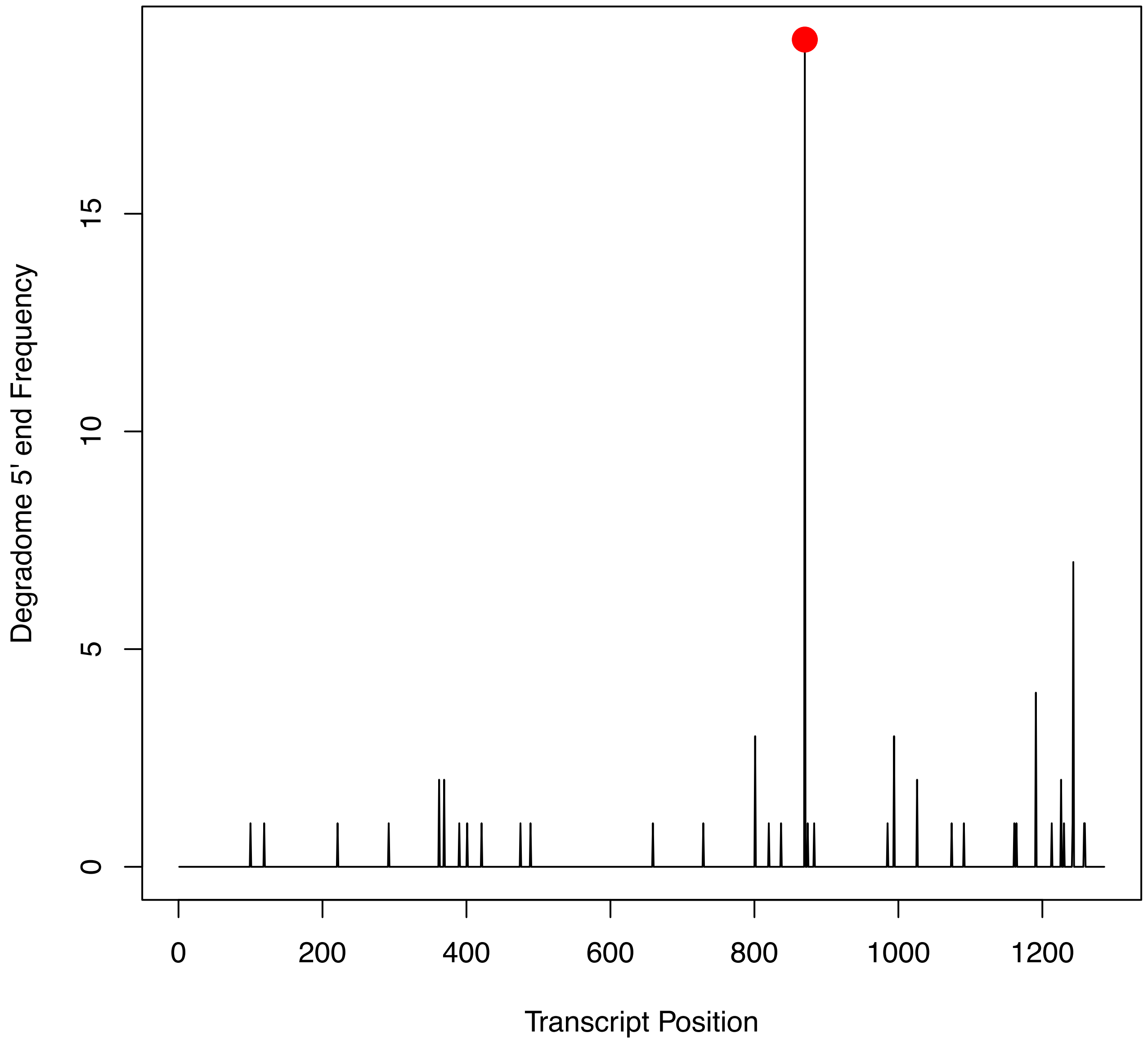

D=Day0
T=HORVU.MOREX.r3.7HG0684000.1
Q=miR156–5p.Cluster_1557.Cluster_1973.Cluster_4963
S=870
category=0
p=0.00204459668115597

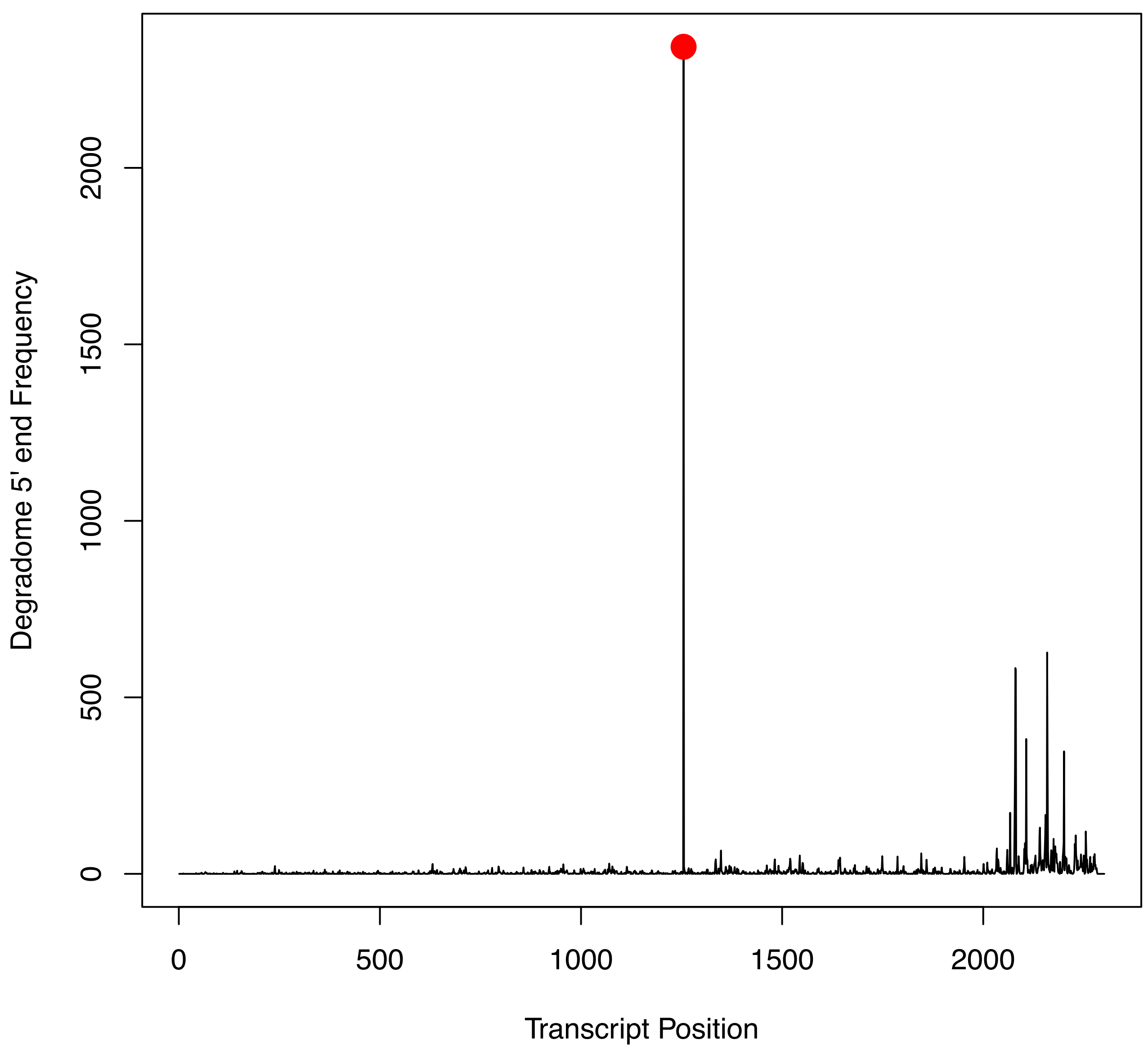

D=Day0
T=HORVU.MOREX.r3.3HG0296070.1
Q=miR159–3p.Cluster_1875
S=1255
category=0
p=0.00122726013299435

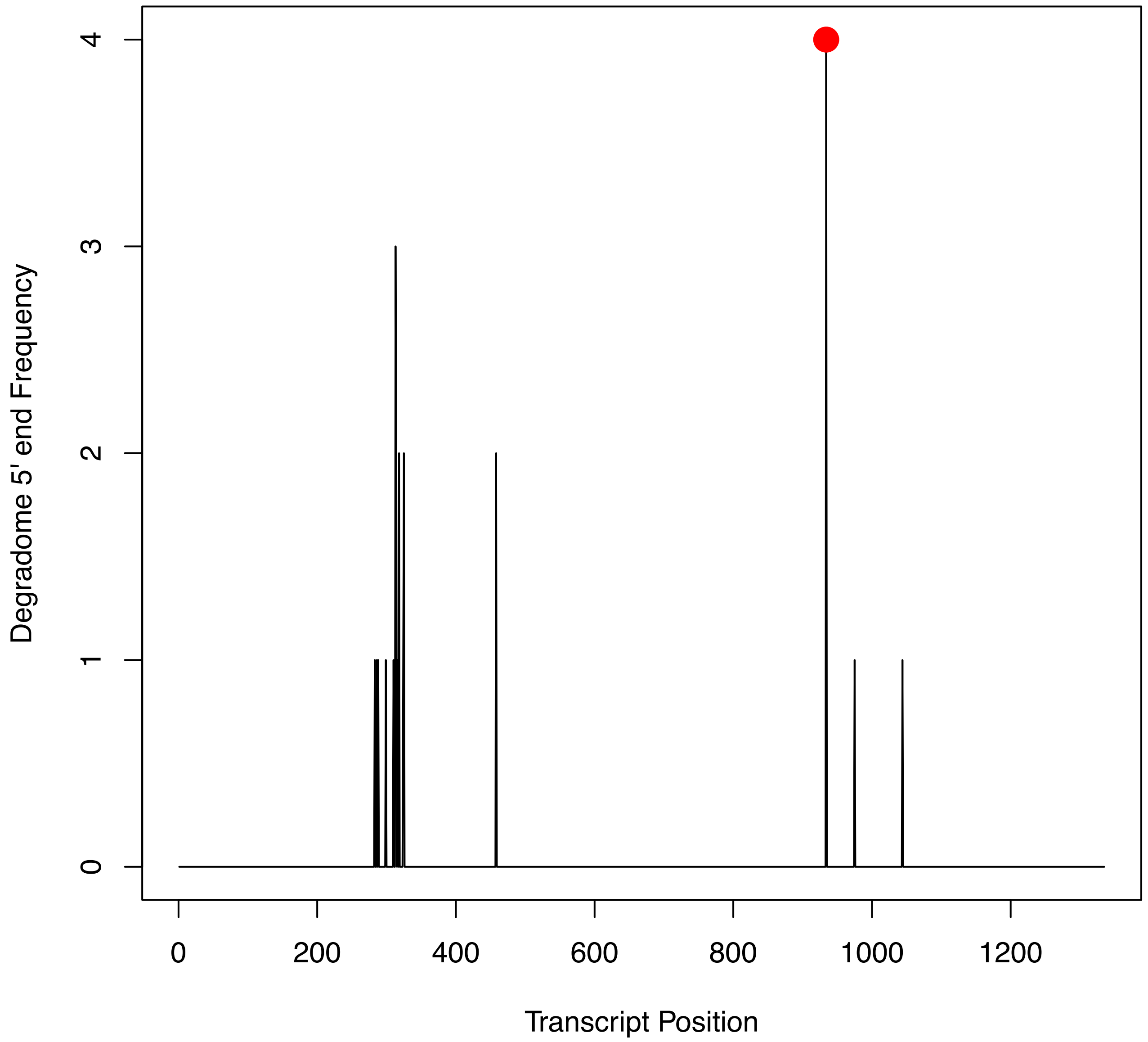

D=Day0
T=HORVU.MOREX.r3.7HG0719850.1
Q=miR159–3p.Cluster_1875
S=934
category=0
p=0.000818340865280653

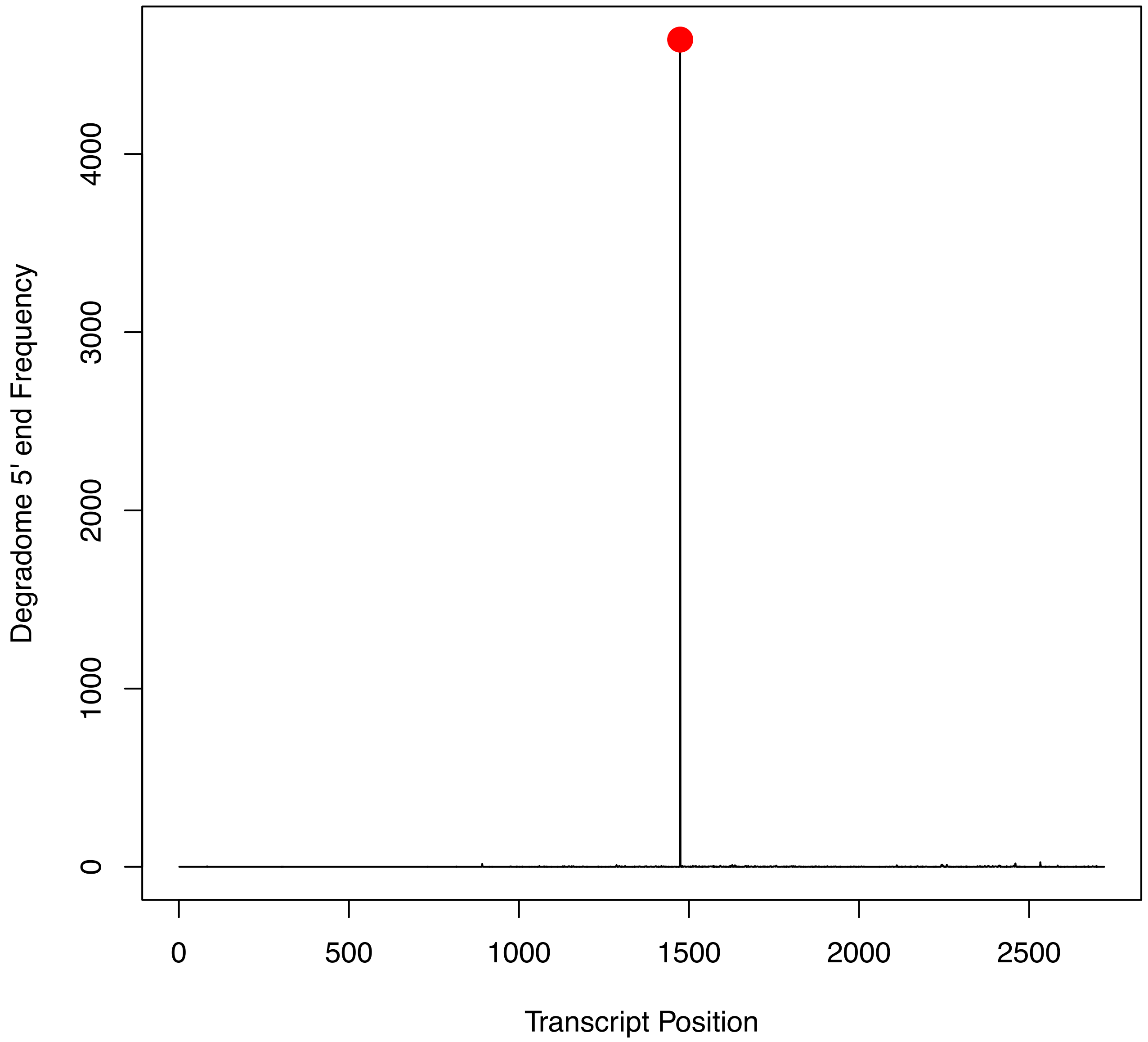

D=Day0
T=HORVU.MOREX.r3.2HG0182280.1
Q=miR160–5p.Cluster_6224
S=1474
category=0
p=0.00245301409855481

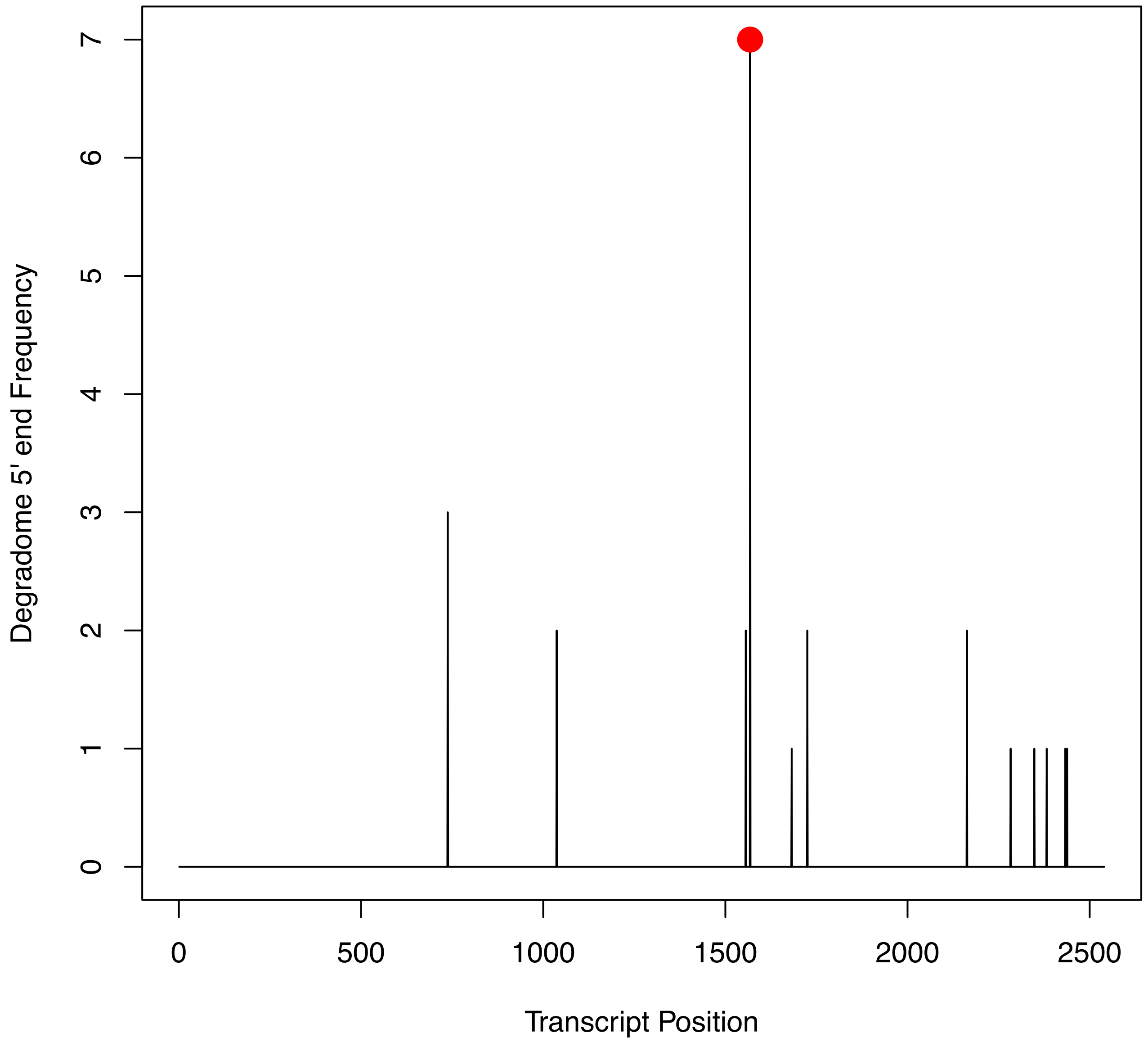

D=Day0
T=HORVU.MOREX.r3.6HG0596670.1
Q=miR160–5p.Cluster_6224
S=1568
category=0
p=0.000409254177131113

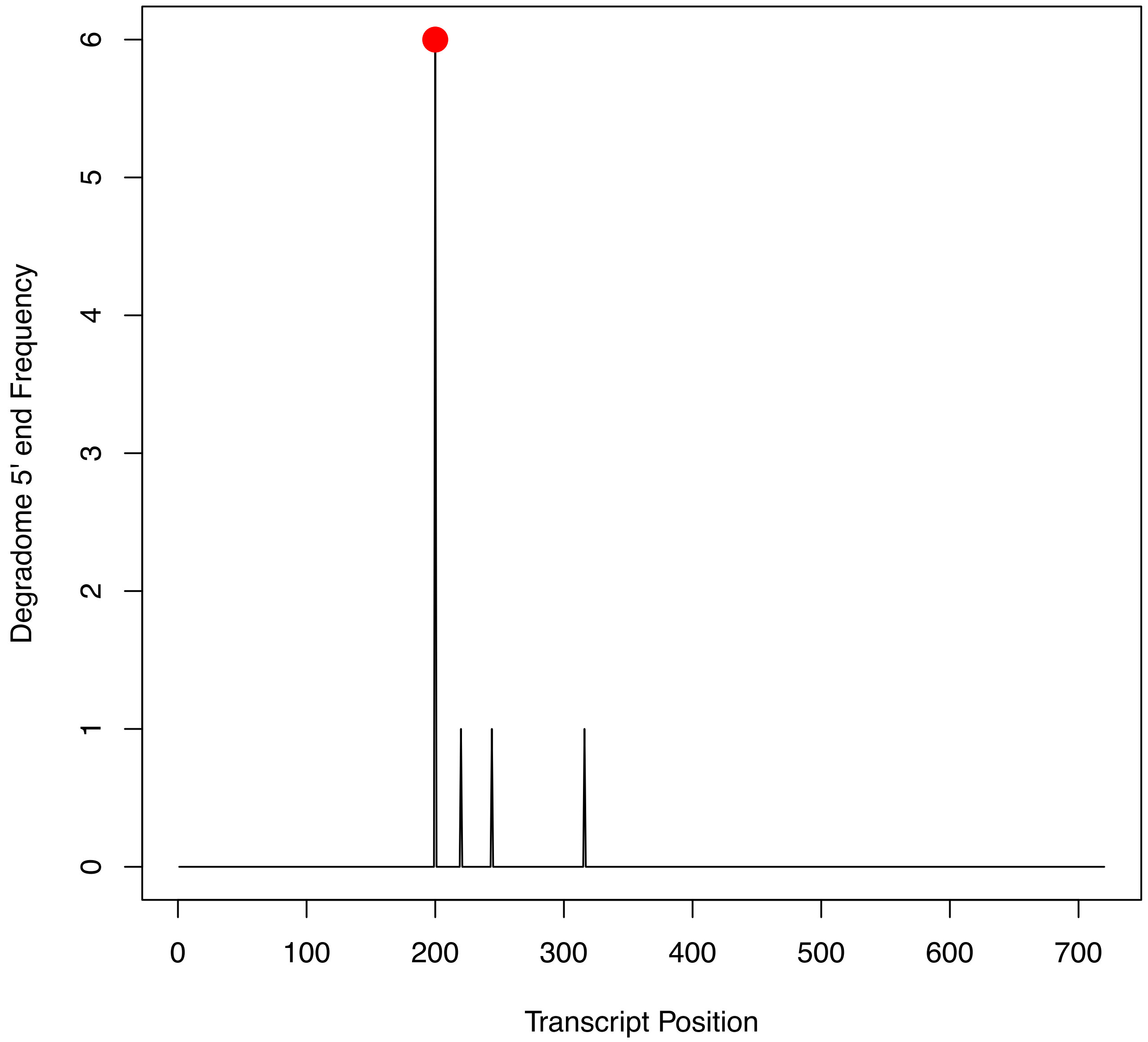

D=Day0
T=HORVU.MOREX.r3.2HG0139920.1
Q=miR166–3p.Cluster_426.Cluster_3396
S=200
category=0
p=0.00245301409855481

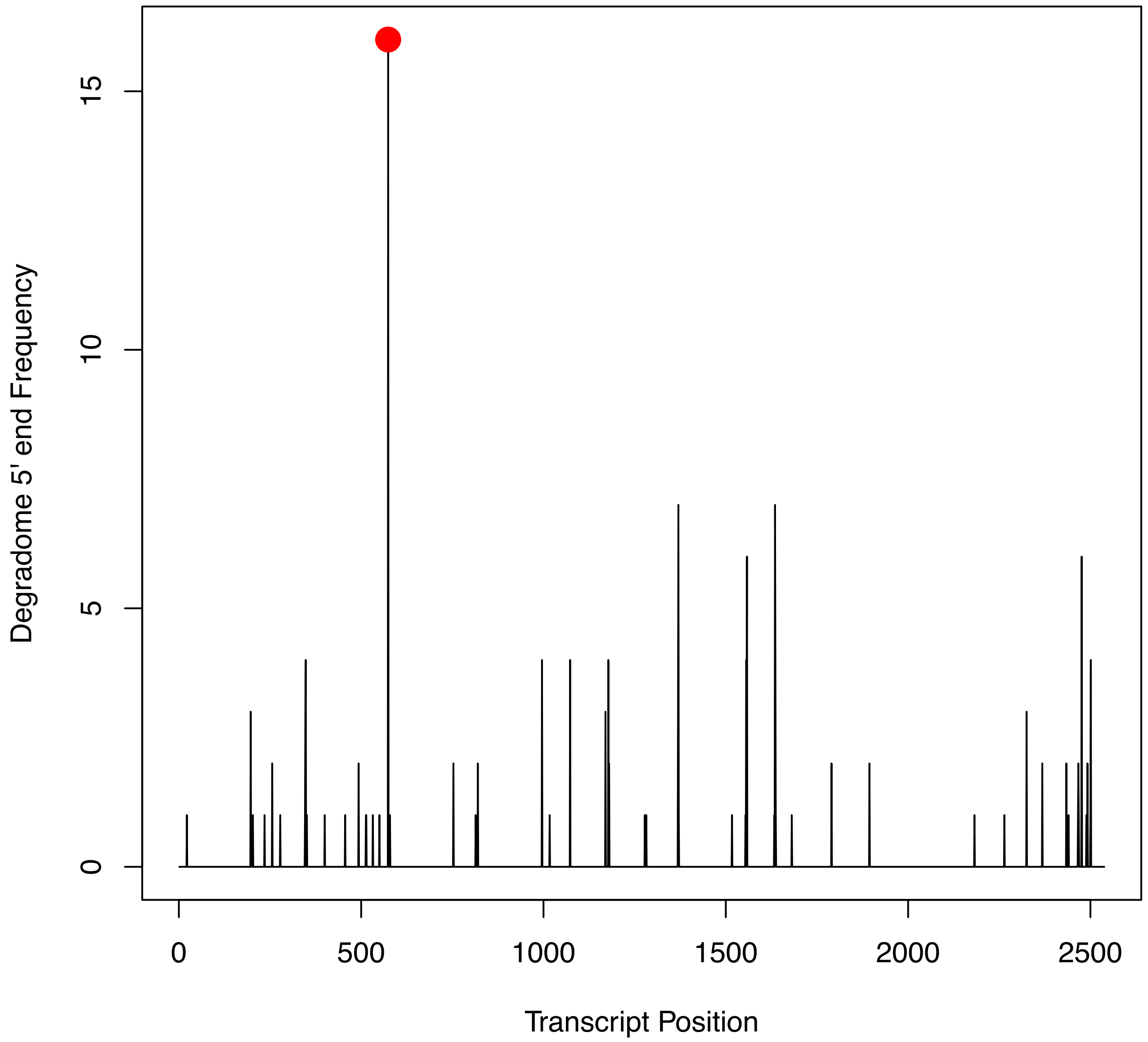

D=Day0
T=HORVU.MOREX.r3.3HG0244080.1
Q=miR166–3p.Cluster_426.Cluster_3396
S=574
category=0
p=0.0012272601329943

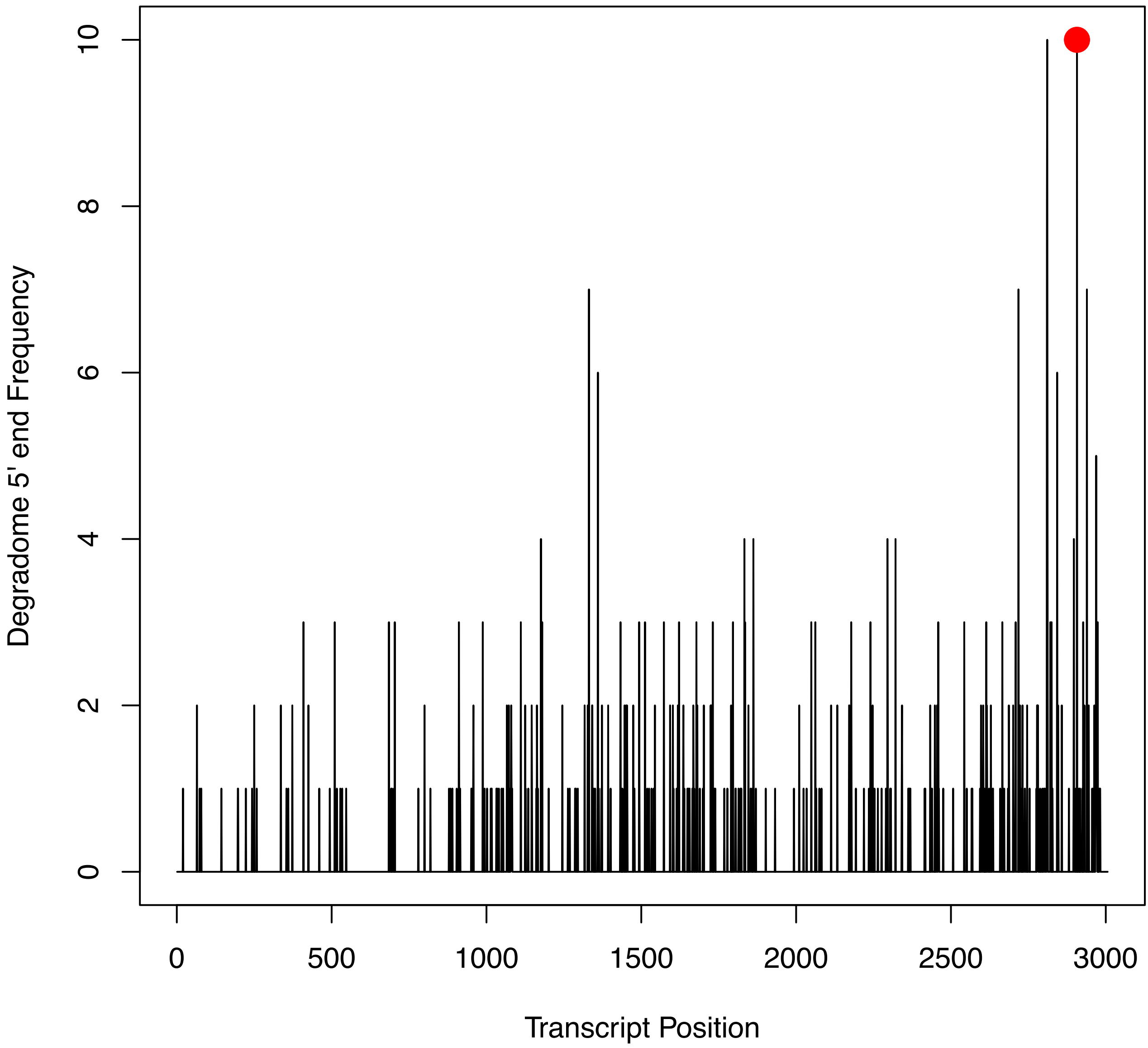

D=Day0
T=HORVU.MOREX.r3.7HG0636350.1
Q=miR167–3p.Cluster_4715
S=2907
category=1
p=0.0396294673610366

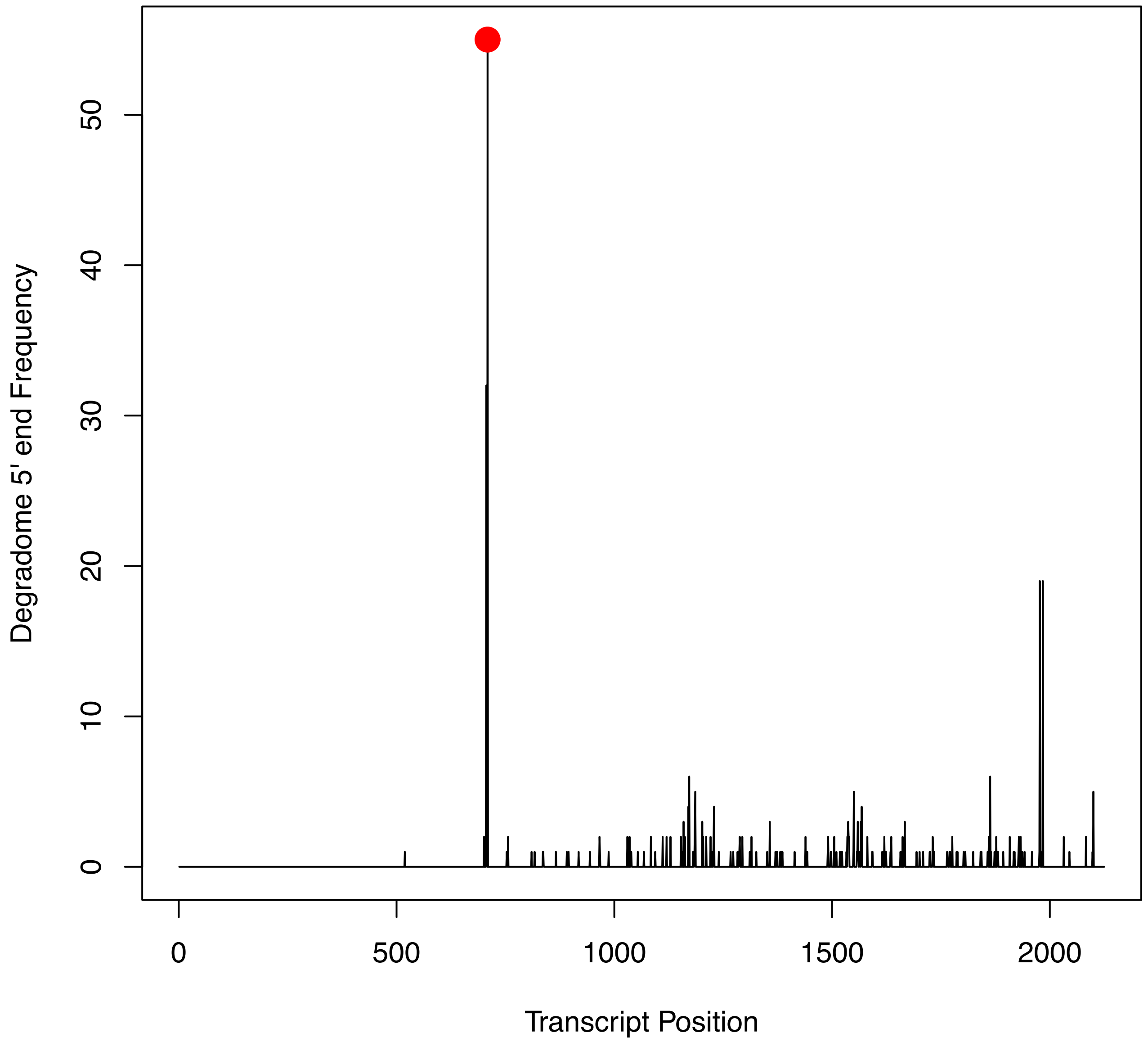

D=Day0
T=HORVU.MOREX.r3.7HG0635740.1
Q=miR171–3p.Cluster_456
S=709
category=0
p=0.000409254177131113

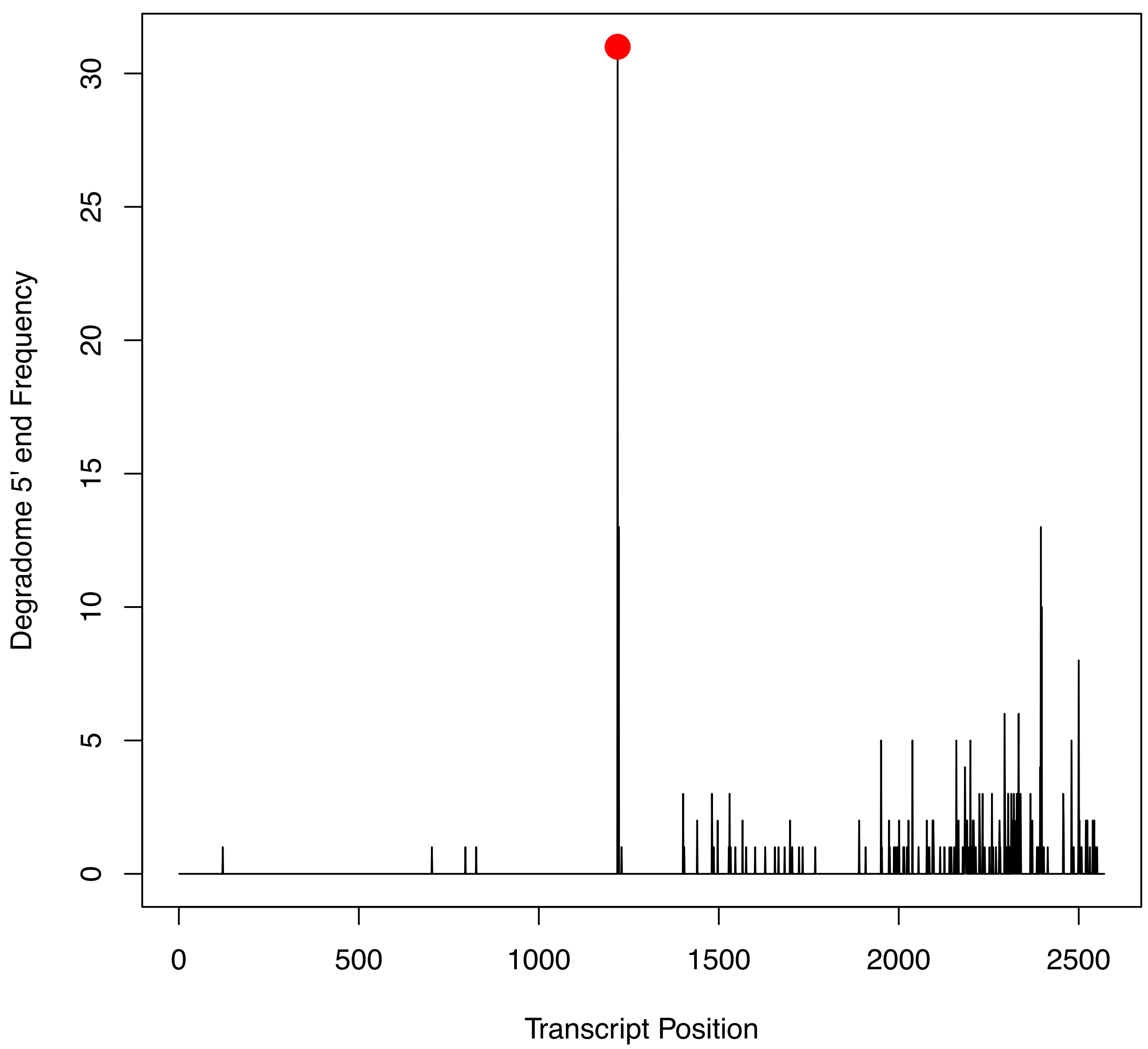

D=Day0
T=HORVU.MOREX.r3.1HG0055960.1
Q=miR171–3p.Cluster_1682
S=1219
category=0
p=0.00122726013299435

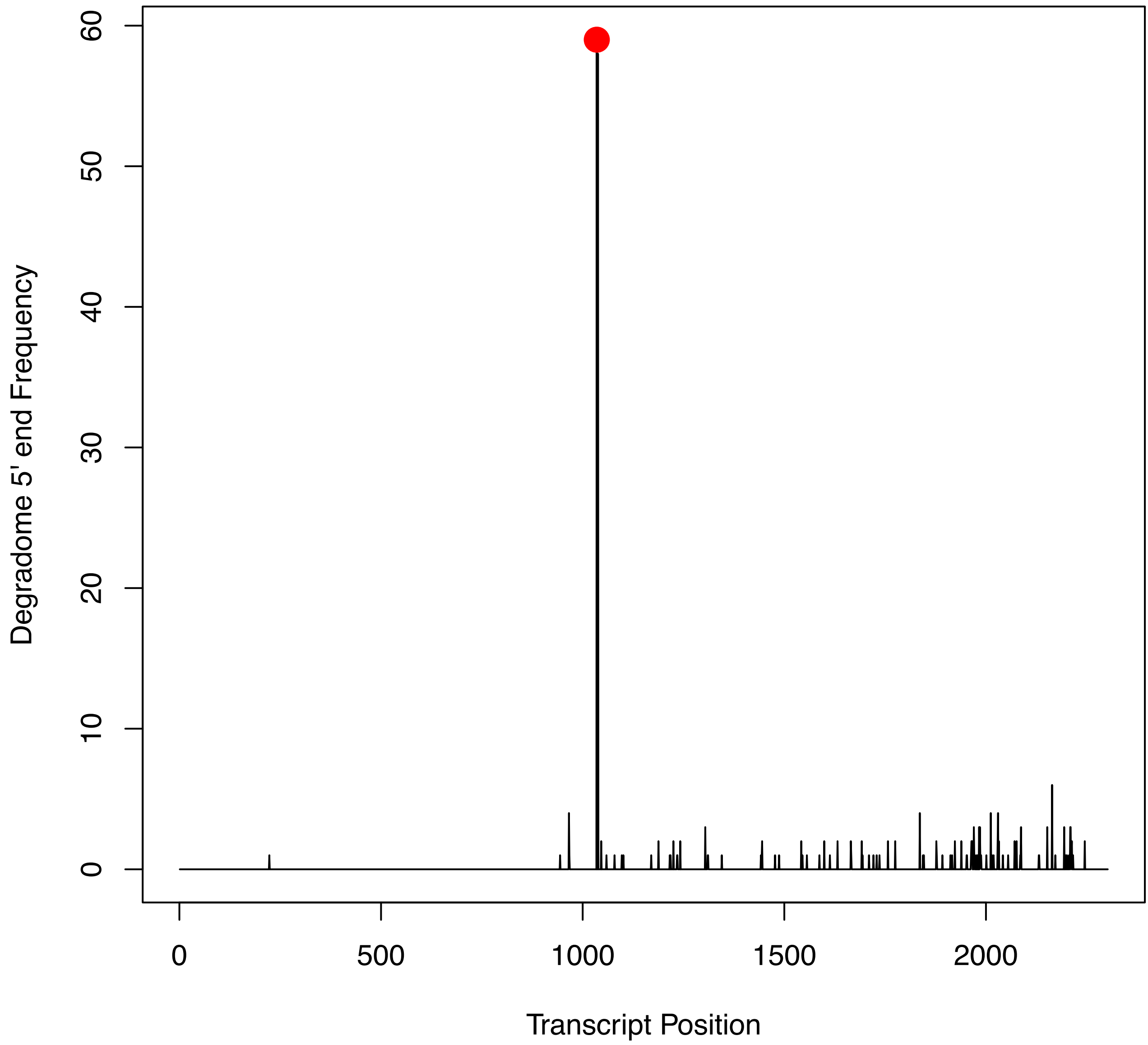

D=Day0
T=HORVU.MOREX.r3.4HG0415480.1
Q=miR171–3p.Cluster_1682
S=1035
category=0
p=0.000409254177131113

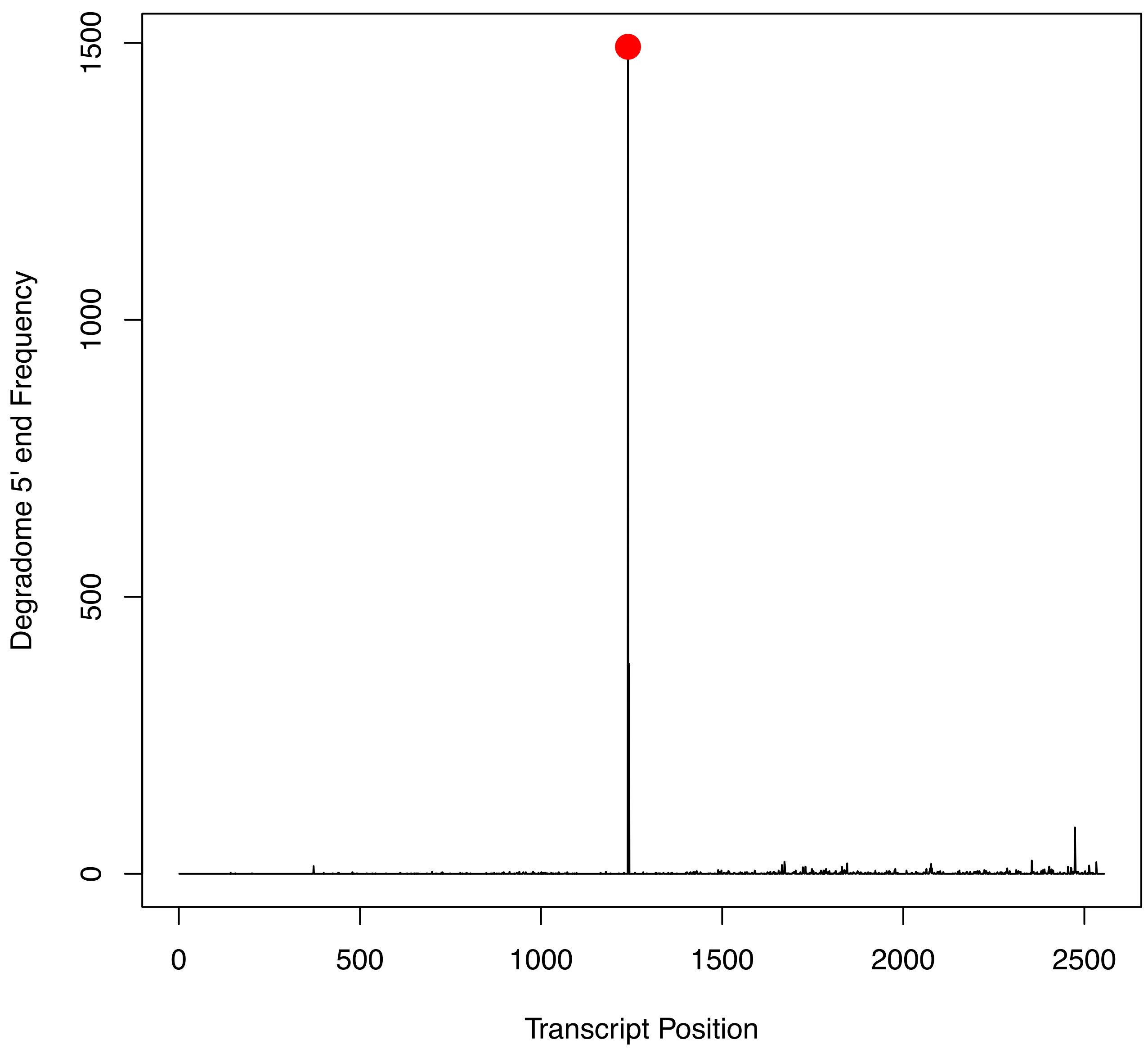

D=Day0
T=HORVU.MOREX.r3.6HG0601750.1
Q=miR171–3p.Cluster_1682
S=1240
category=0
p=0.000818340865280653

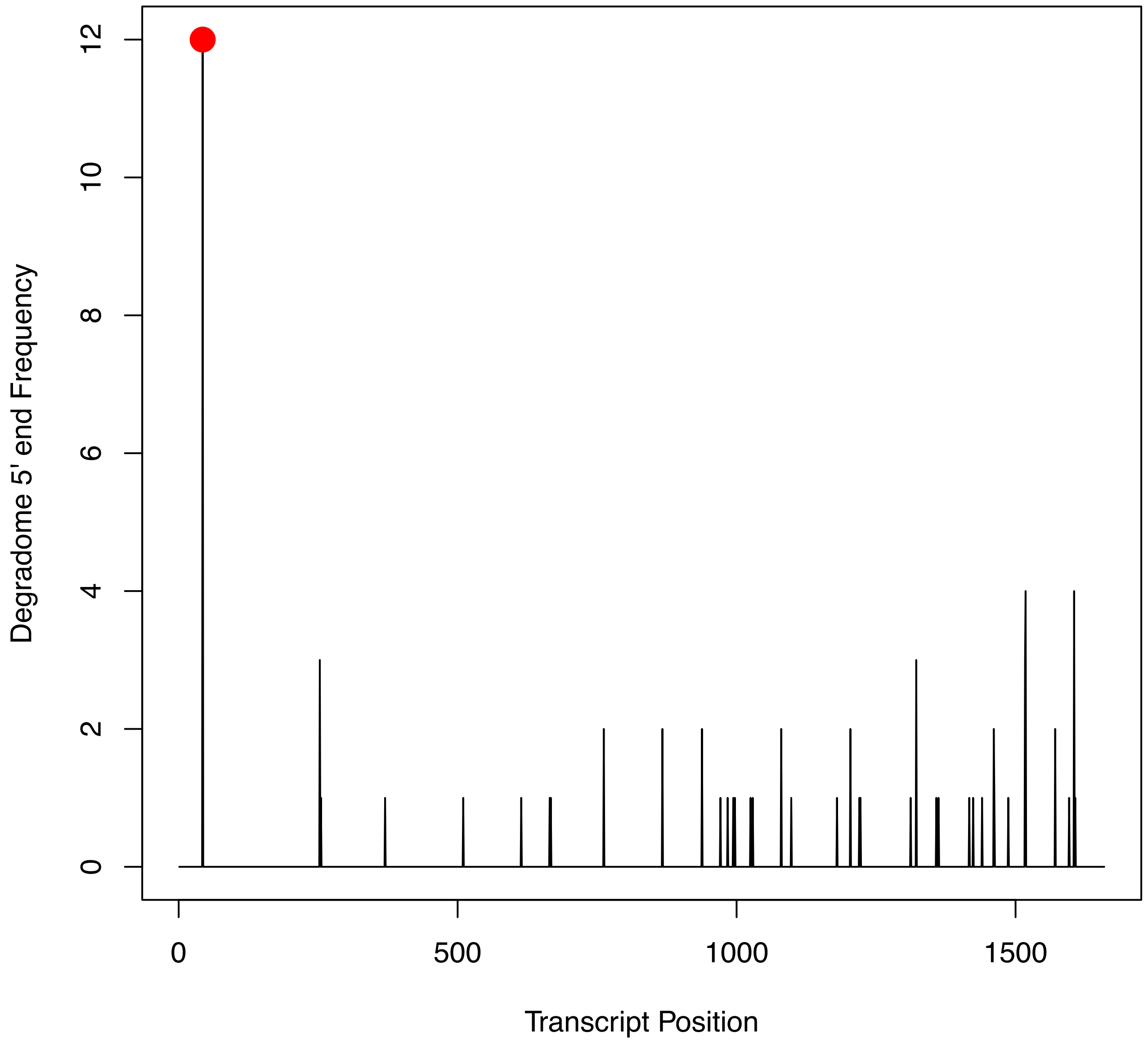

D=Day0
T=HORVU.MOREX.r3.2HG0142450.1
Q=miR319–3p.Cluster_2046
S=43
category=0
p=0.00122726013299435

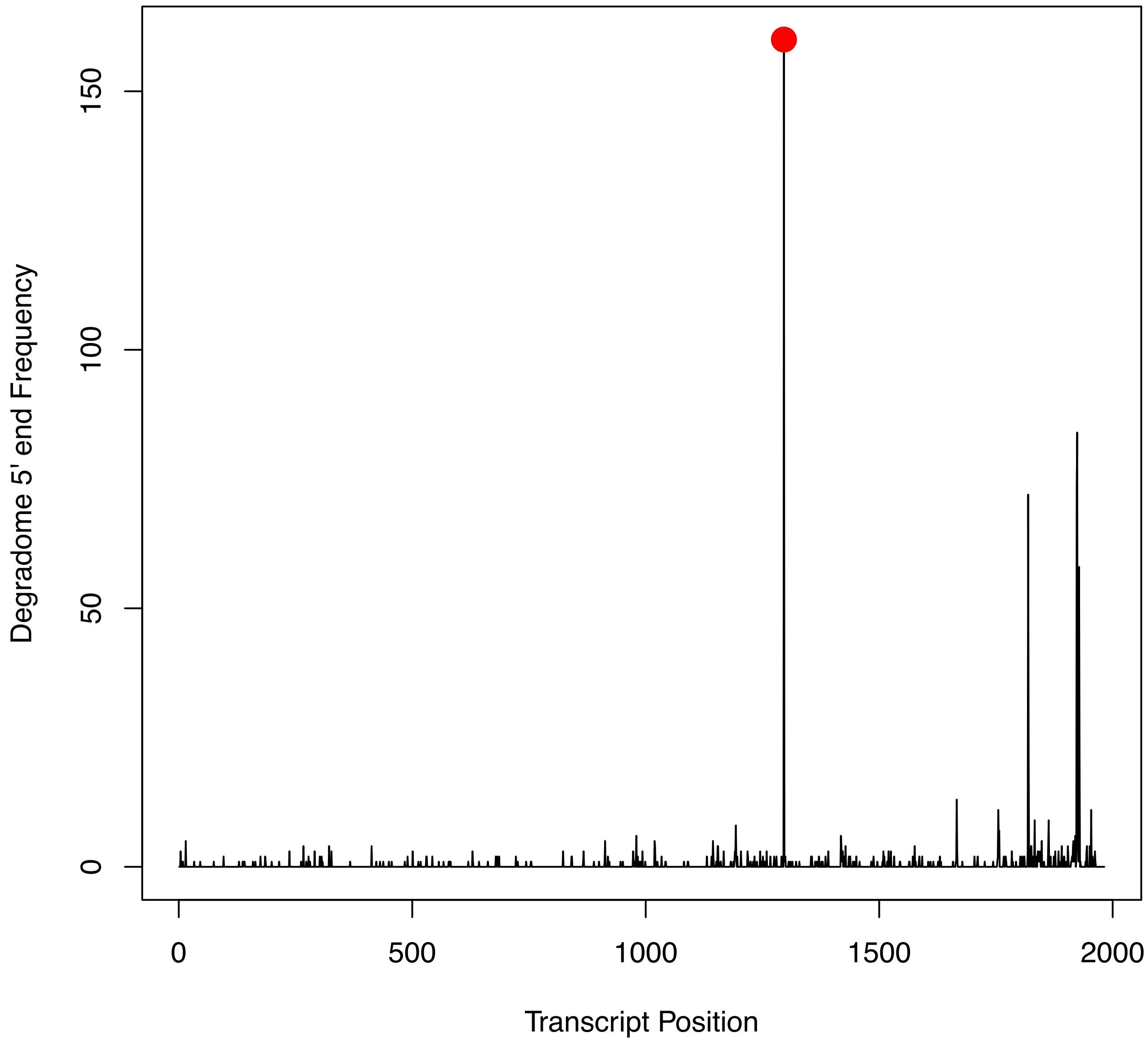

D=Day0
T=HORVU.MOREX.r3.2HG0152890.1
Q=miR319–3p.Cluster_2046
S=1296
category=0
p=0.000818340865280653

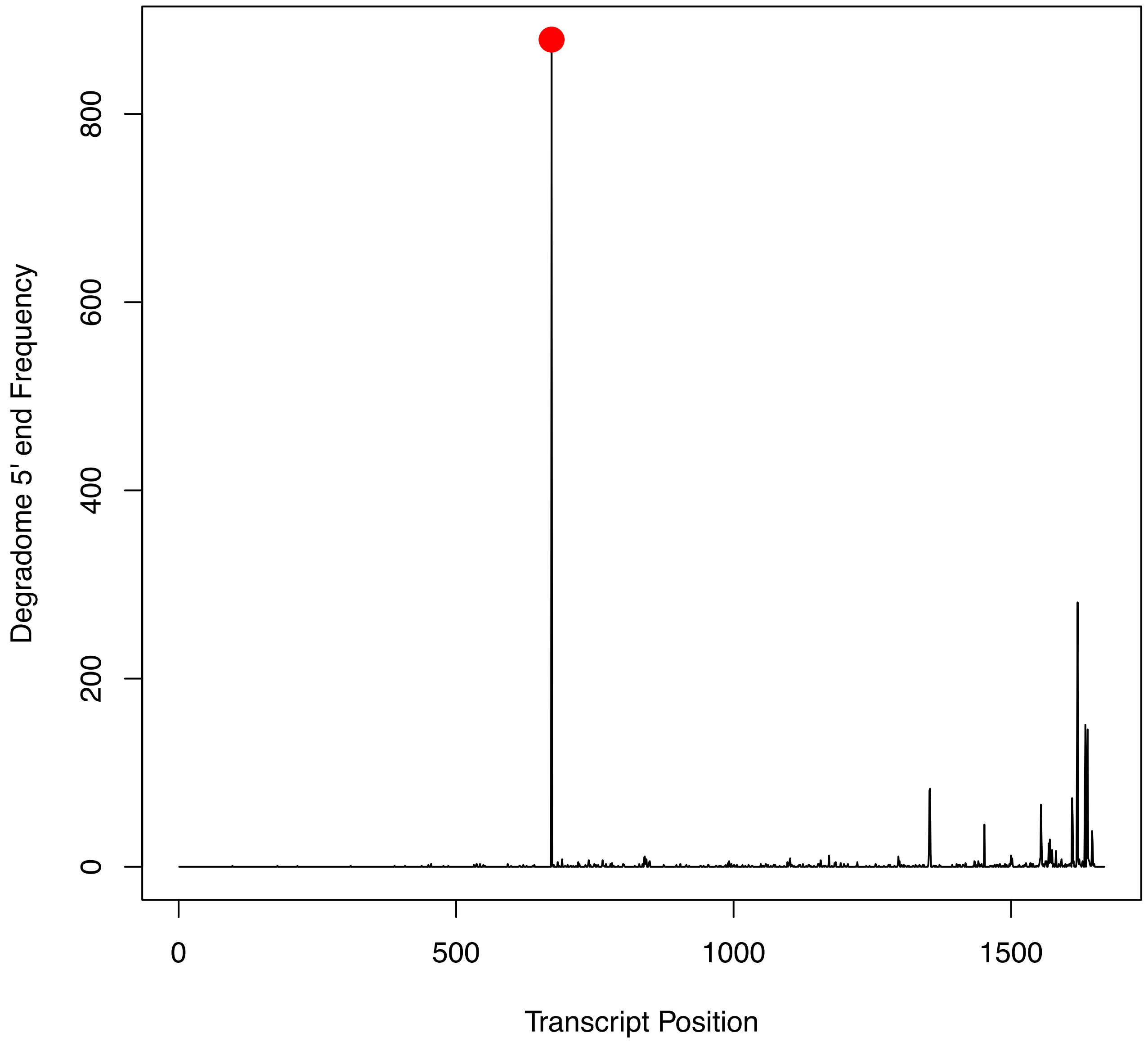

D=Day0
T=HORVU.MOREX.r3.2HG0193490.1
Q=miR396–5p.Cluster_1803
S=672
category=0
p=0.00122726013299435

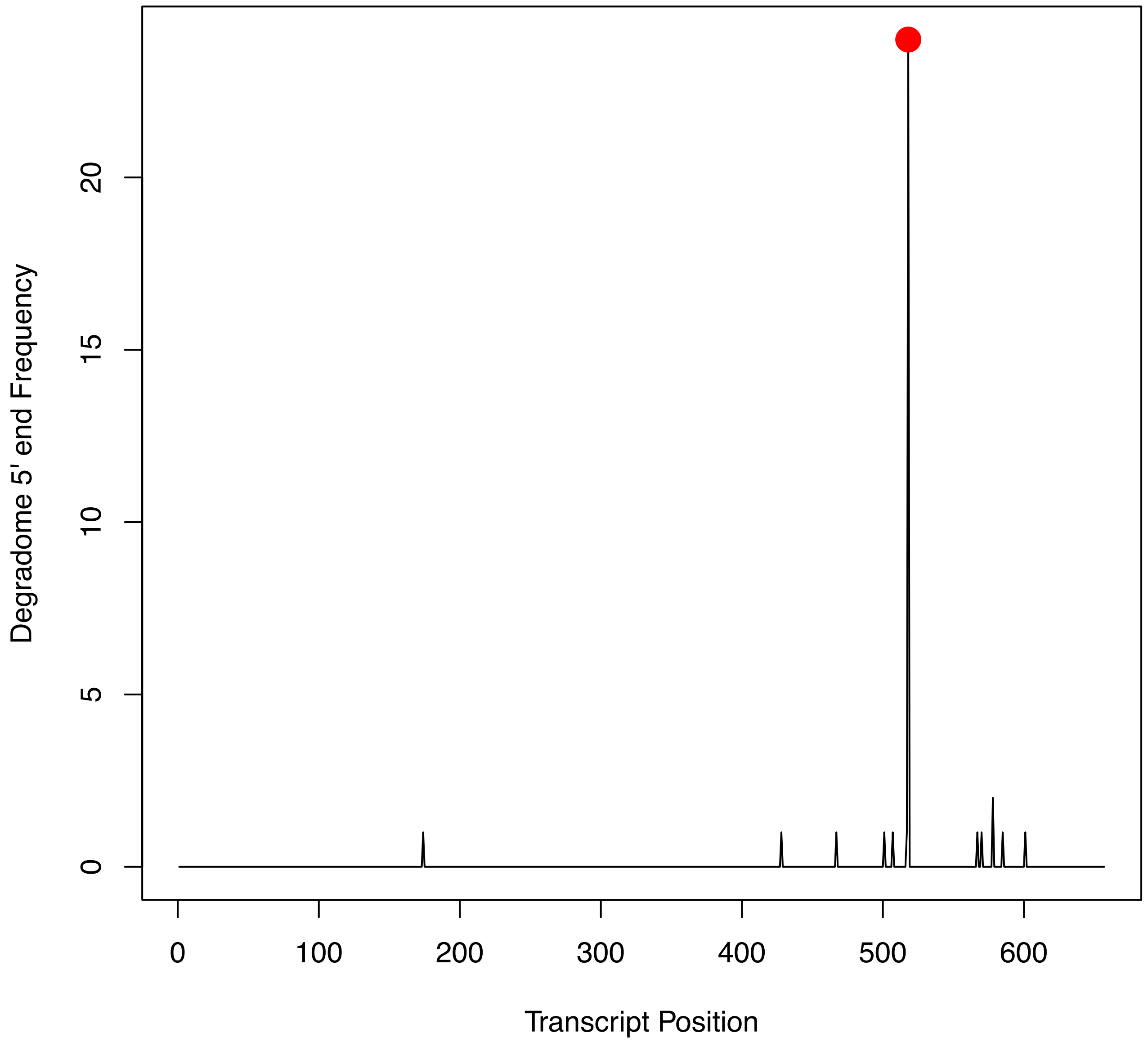

D=Day0
T=HORVU.MOREX.r3.6HG0603870.1
Q=miR396–5p.Cluster_1803
S=518
category=0
p=0.00163601204878971

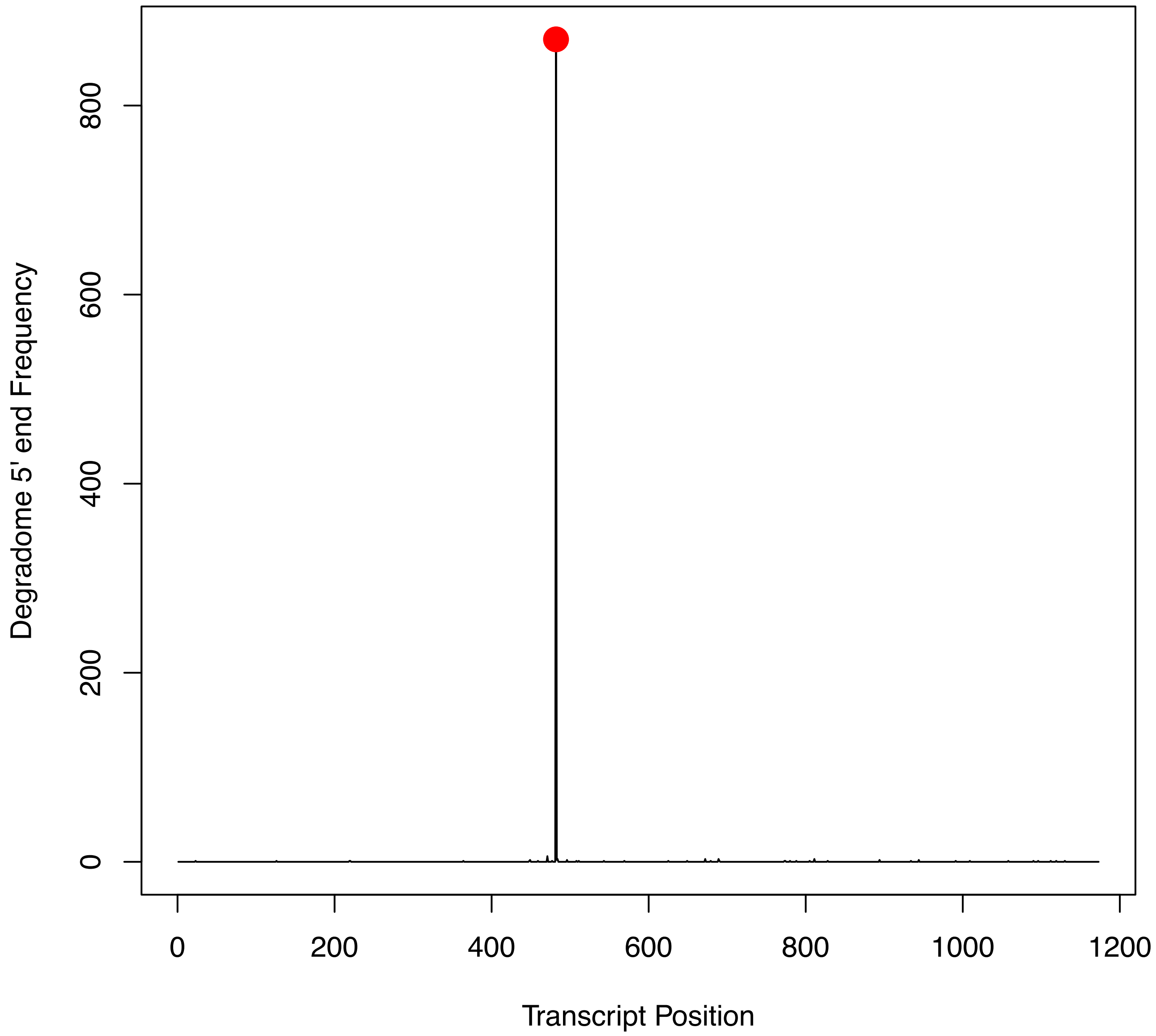

D=Day0
T=HORVU.MOREX.r3.6HG0606810.1
Q=miR396–5p.Cluster_1803
S=482
category=0
p=0.00326934756215558

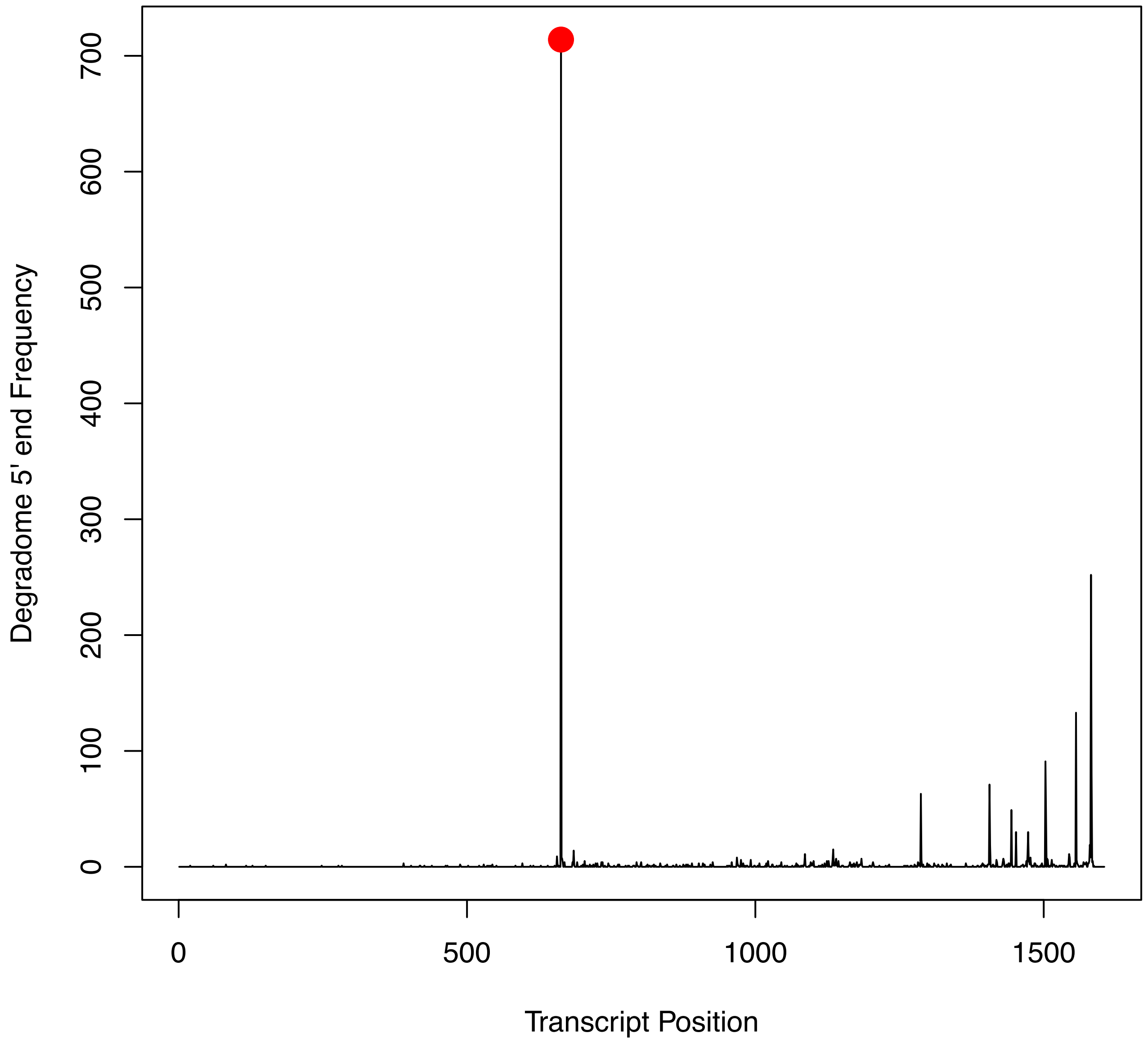

D=Day0
T=HORVU.MOREX.r3.7HG0641080.1
Q=miR396–5p.Cluster_1803
S=663
category=0
p=0.00286126436941947

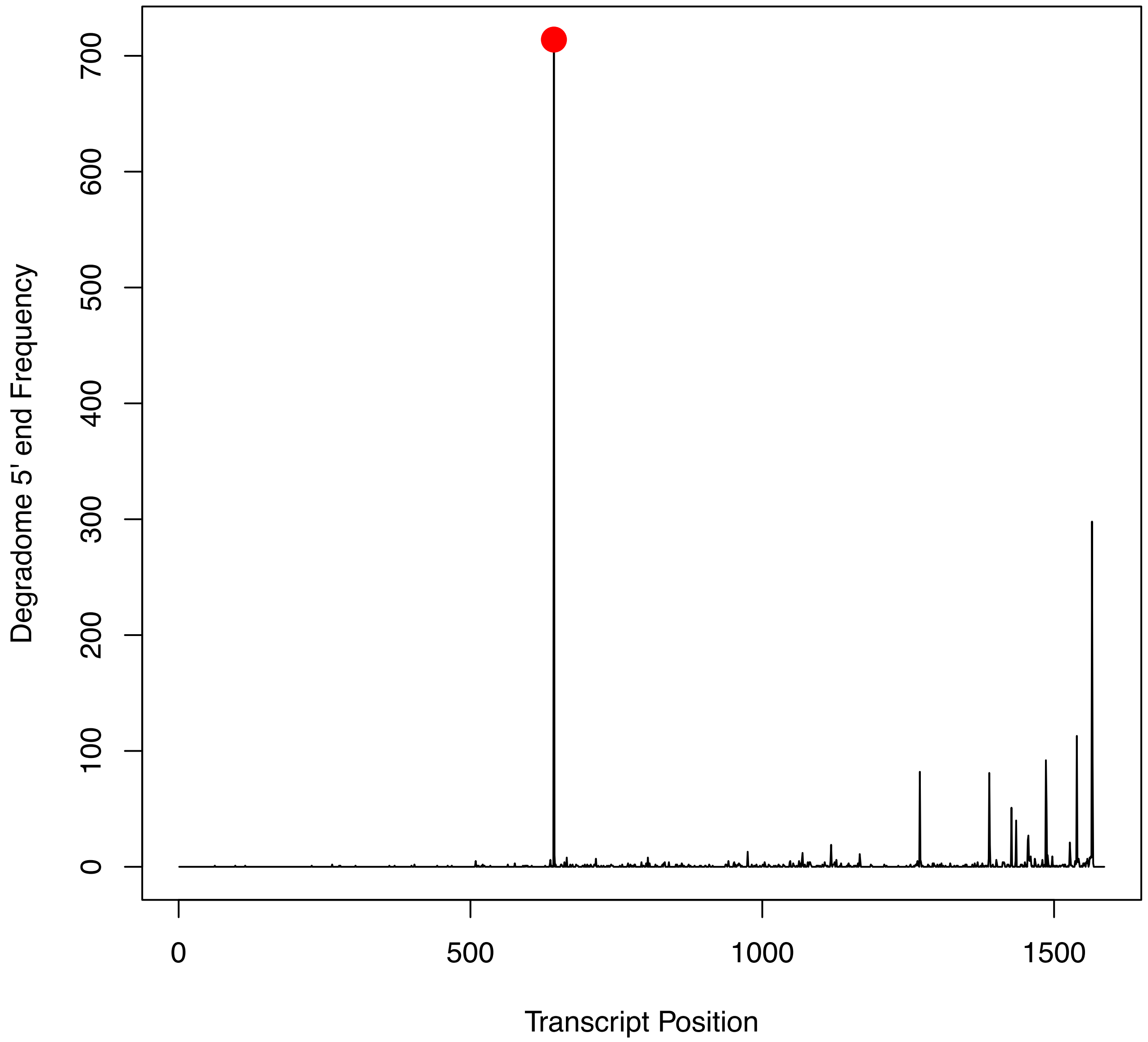

D=Day0
T=HORVU.MOREX.r3.7HG0641080.2
Q=miR396–5p.Cluster_1803
S=643
category=0
p=0.00367726374514032

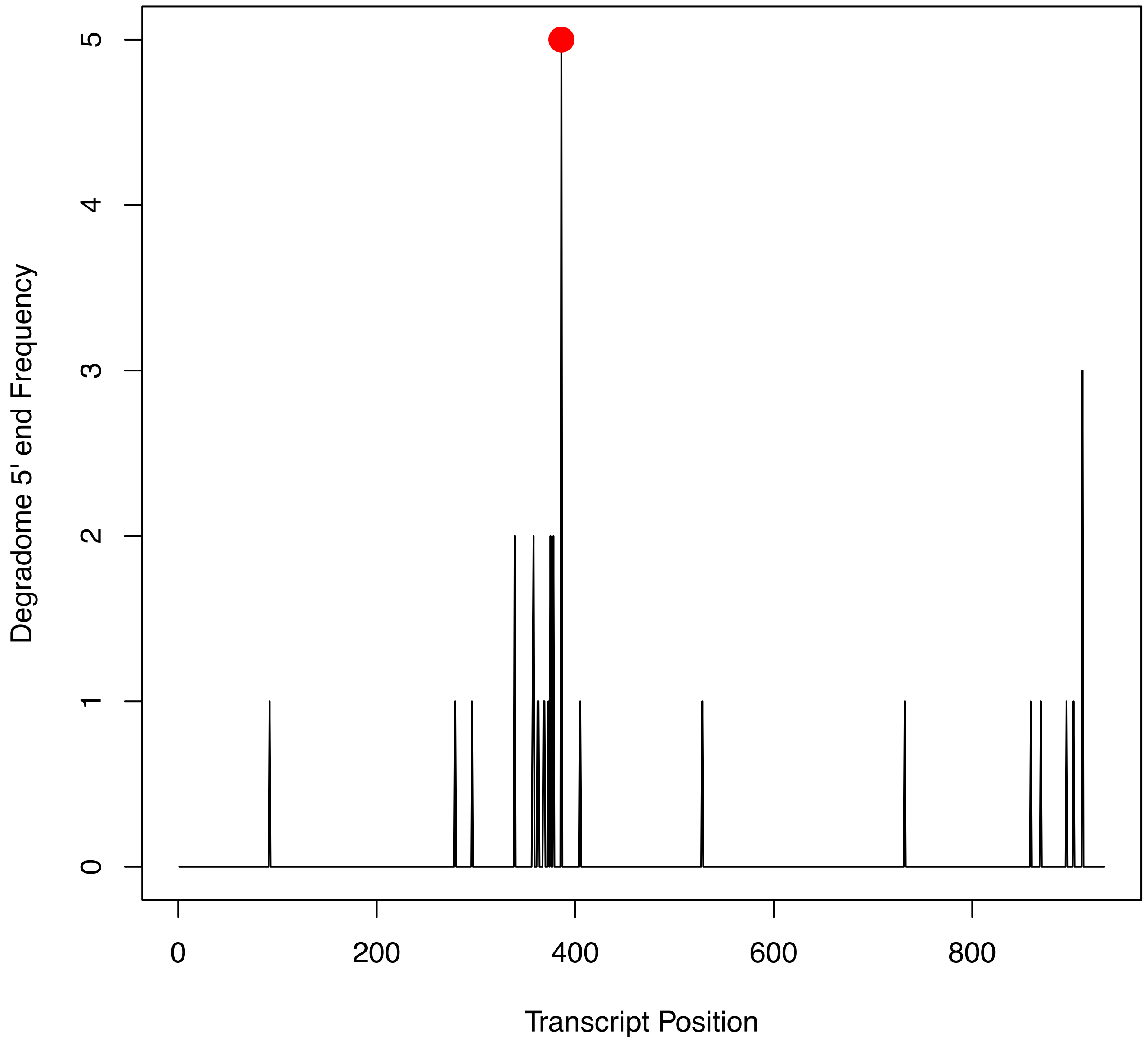

D=Day0
T=HORVU.MOREX.r3.7HG0662690.1
Q=miR396–5p.Cluster_1803
S=386
category=0
p=0.000818340865280653

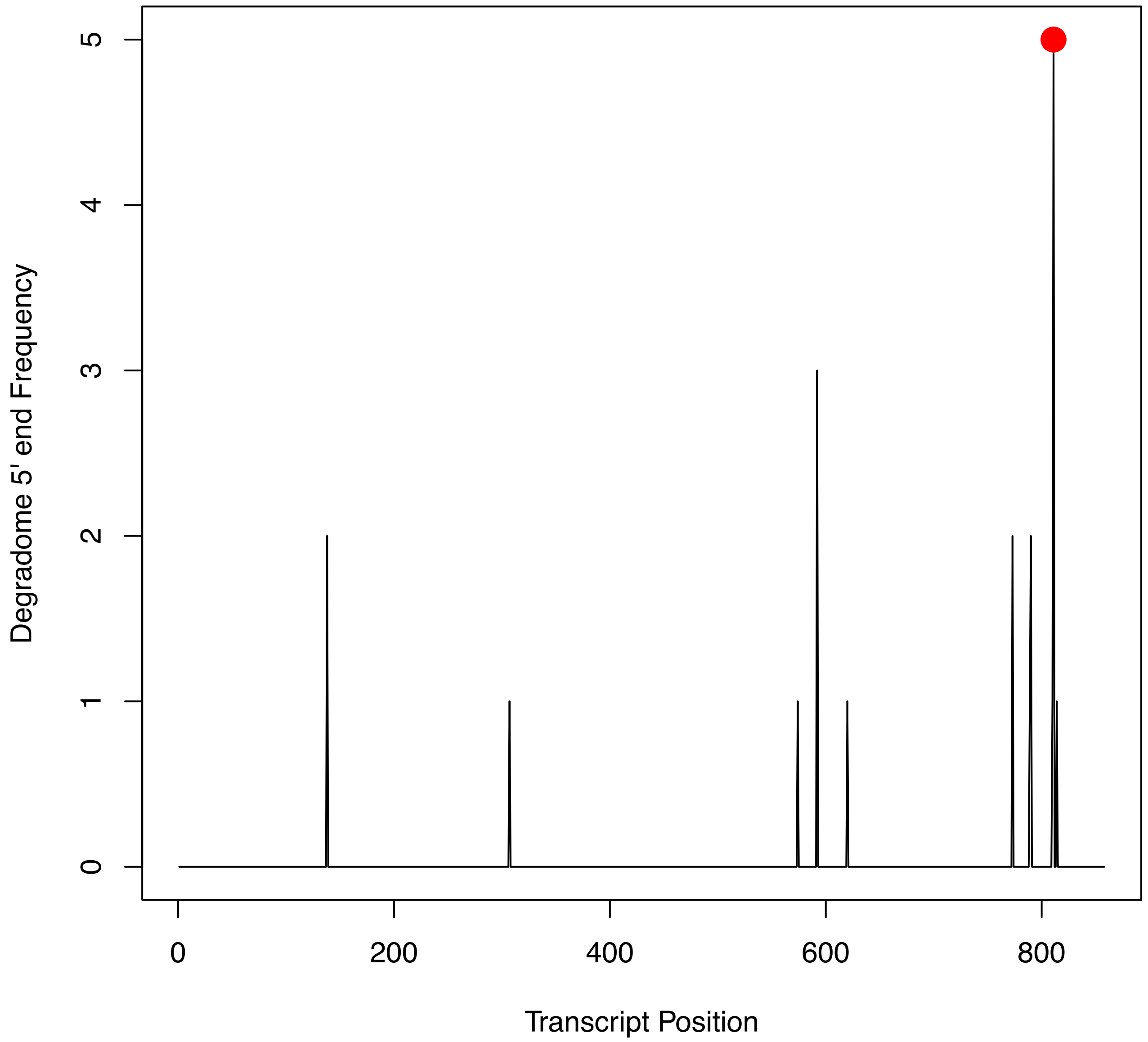

D=Day0
T=HORVU.MOREX.r3.3HG0279880.1
Q=miR827–5p.Cluster_1545
S=811
category=0
p=0.0420699153148275

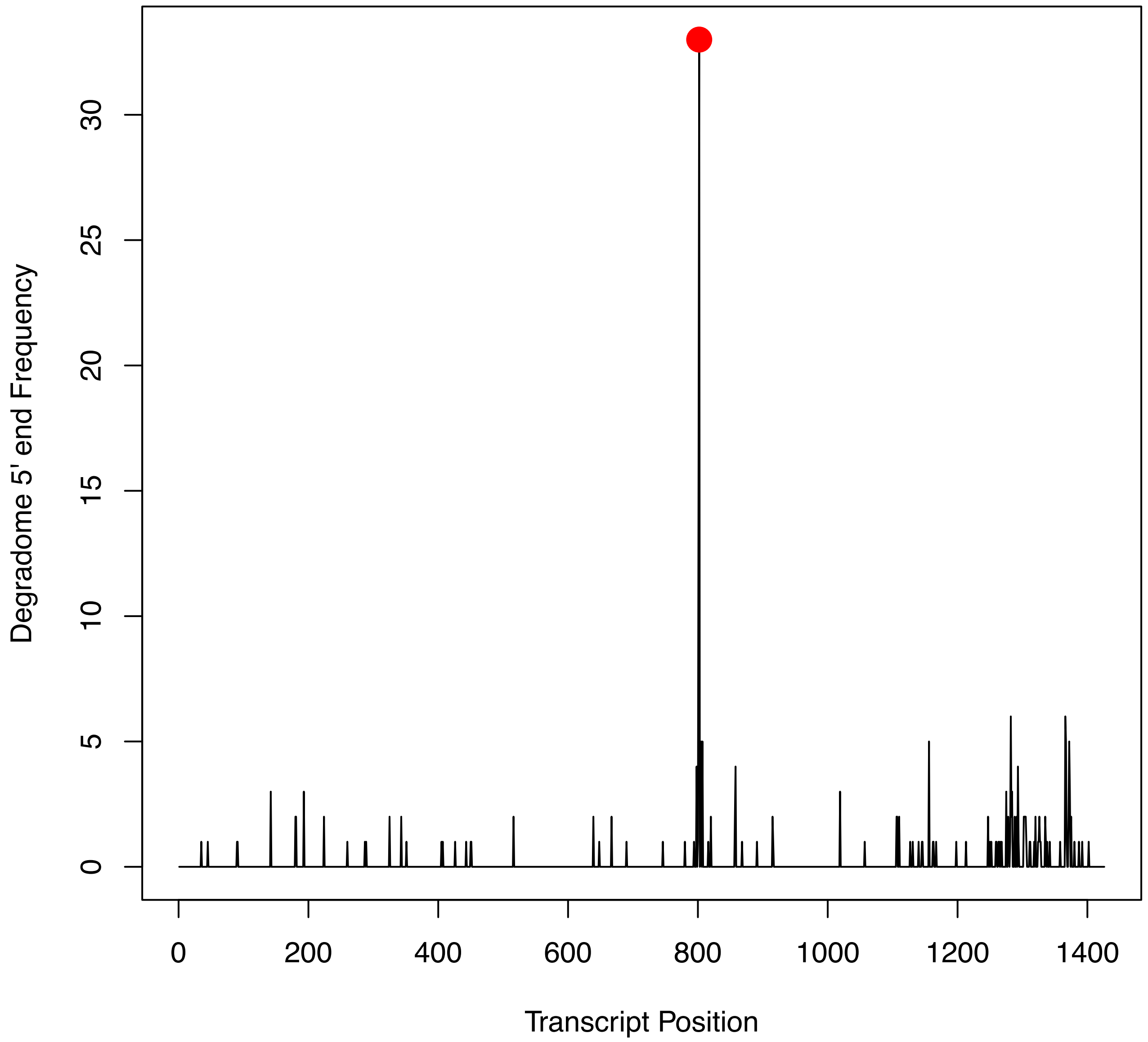

D=Day0
T=HORVU.MOREX.r3.6HG0541280.1
Q=miR9662–3p.Cluster_4660
S=802
category=0
p=0.0345906427855263

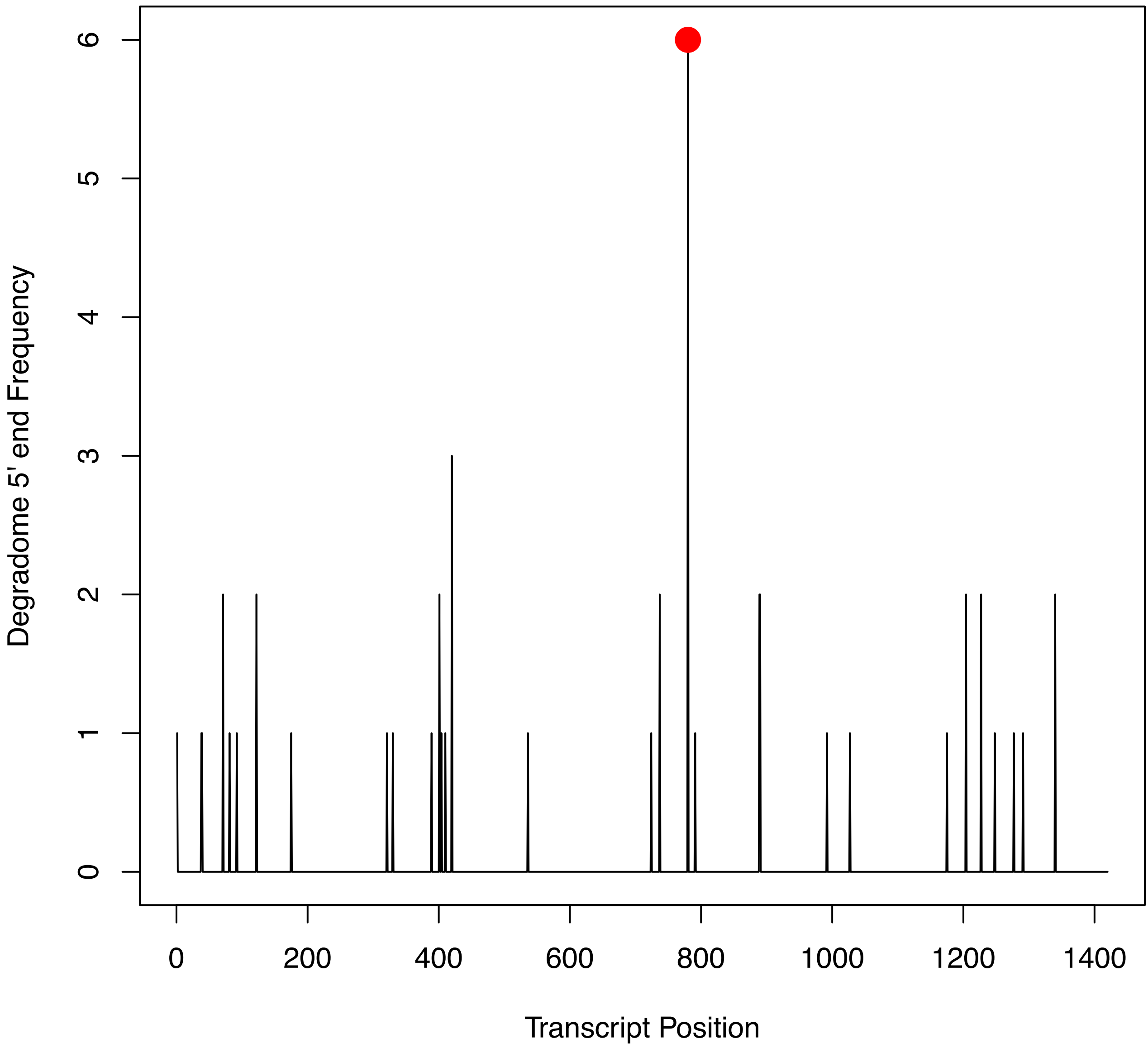

D=Day0
T=HORVU.MOREX.r3.6HG0541690.1
Q=miR9662–3p.Cluster_4660
S=780
category=0
p=0.00204459668115597

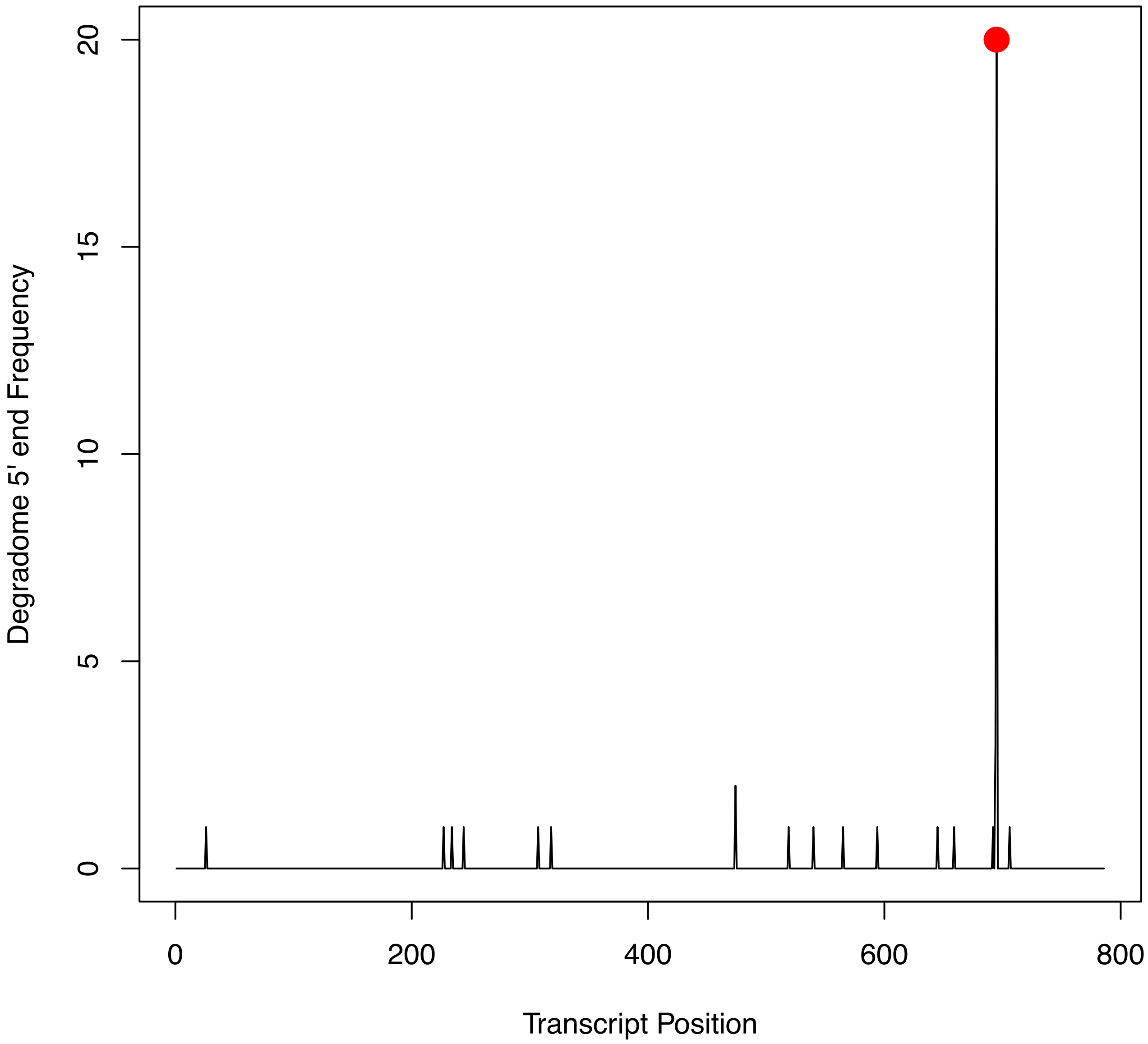

D=Day0
T=HORVU.MOREX.r3.6HG0543460.1
Q=miR9662–3p.Cluster_4660
S=695
category=0
p=0.00818340865280653

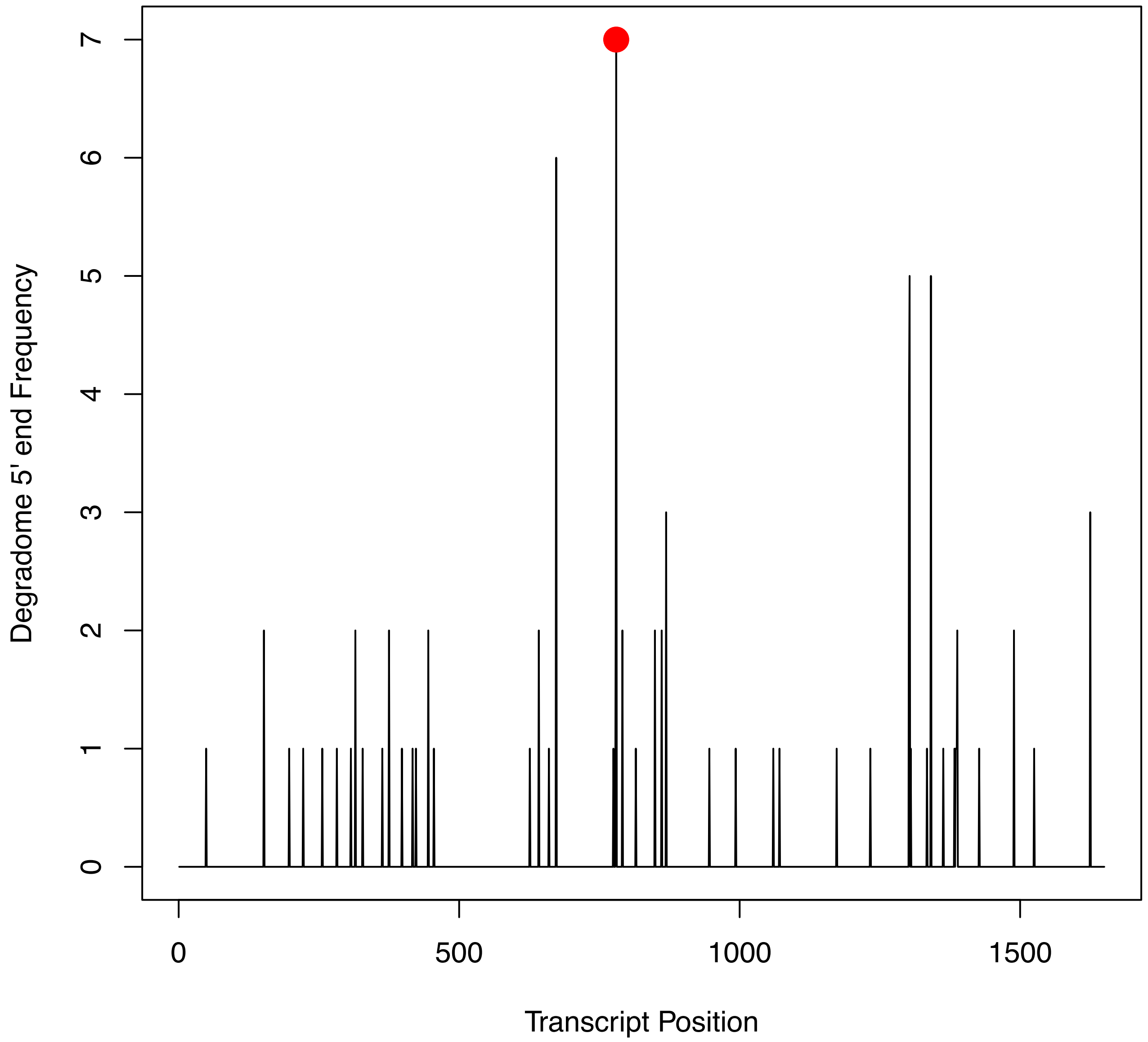

D=Day0
T=HORVU.MOREX.r3.7HG0710980.1
Q=miR9662–3p.Cluster_4660
S=780
category=0
p=0.0158374277099875

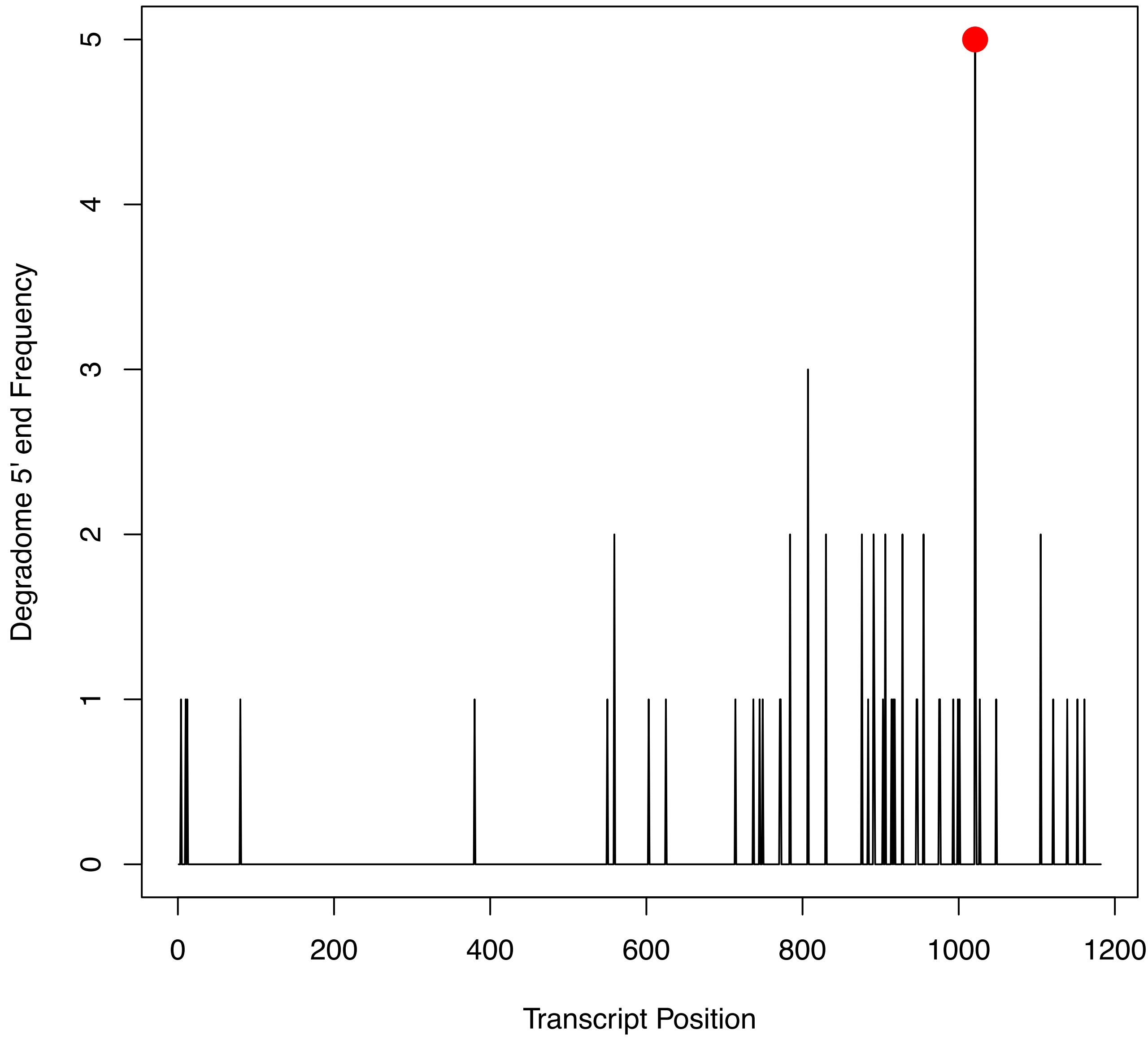

D=Day0
T=HORVU.MOREX.r3.5HG0534390.1
Q=novel–5p.Cluster_68
S=1021
category=0
p=0.0349857405976077

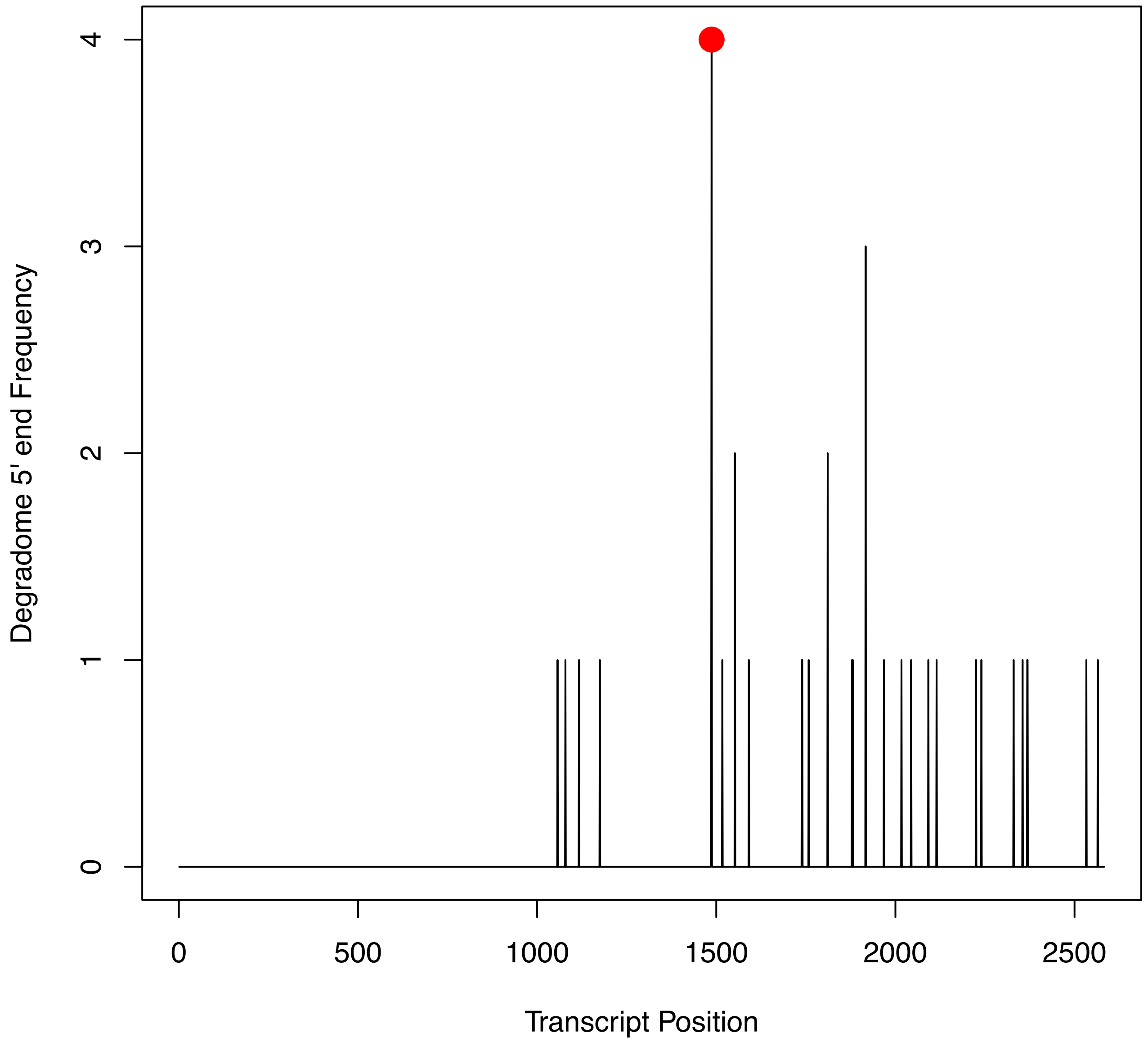

D=Day0
T=HORVU.MOREX.r3.1HG0011450.1
Q=novel–5p.Cluster_2558
S=1487
category=0
p=0.00286126436941947

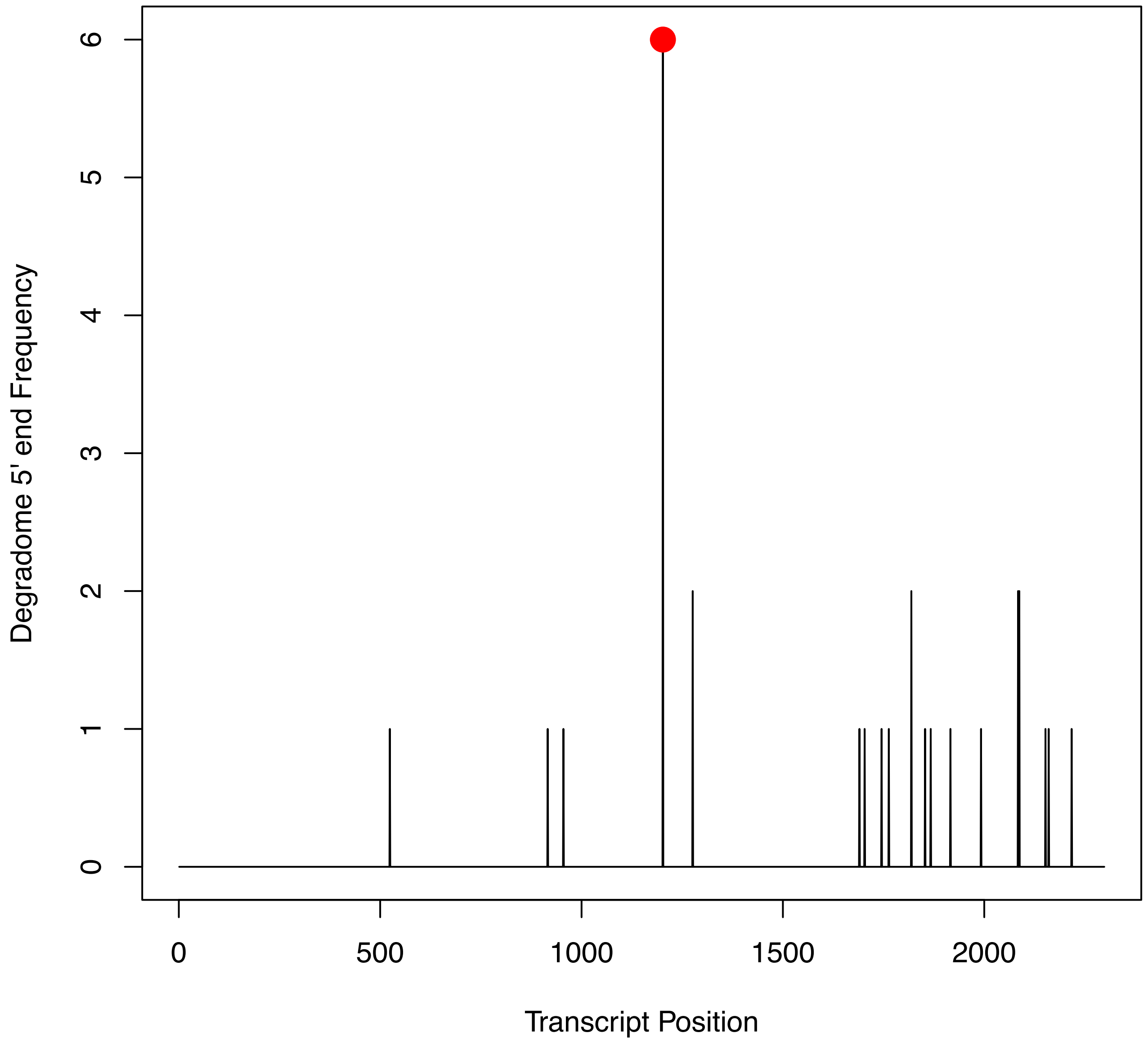

D=Day0
T=HORVU.MOREX.r3.1HG0011510.1
Q=novel–5p.Cluster_2558
S=1202
category=0
p=0.00408501298672337

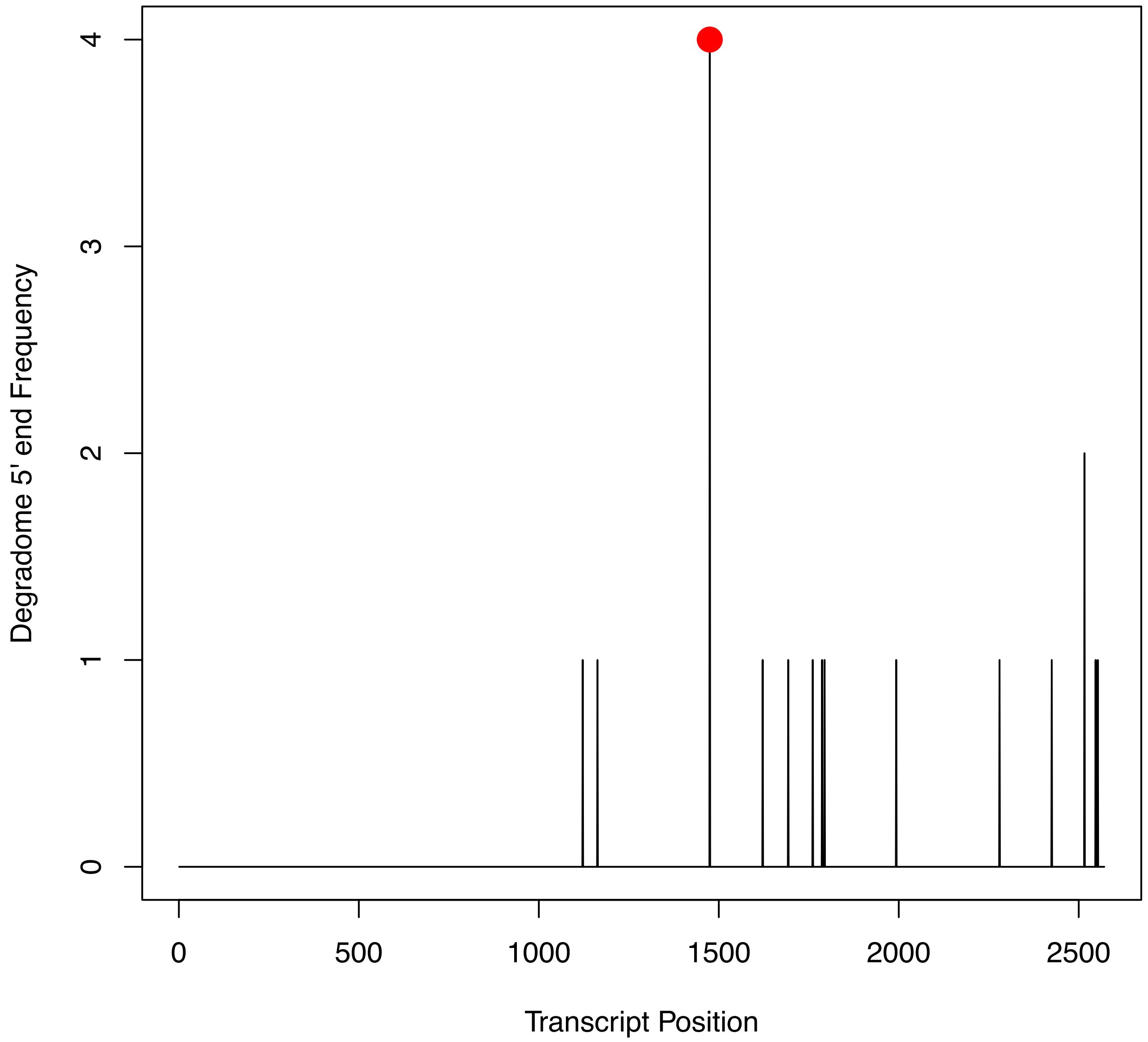

D=Day0
T=HORVU.MOREX.r3.1HG0011560.1
Q=novel–5p.Cluster_2558
S=1475
category=0
p=0.00163601204878971

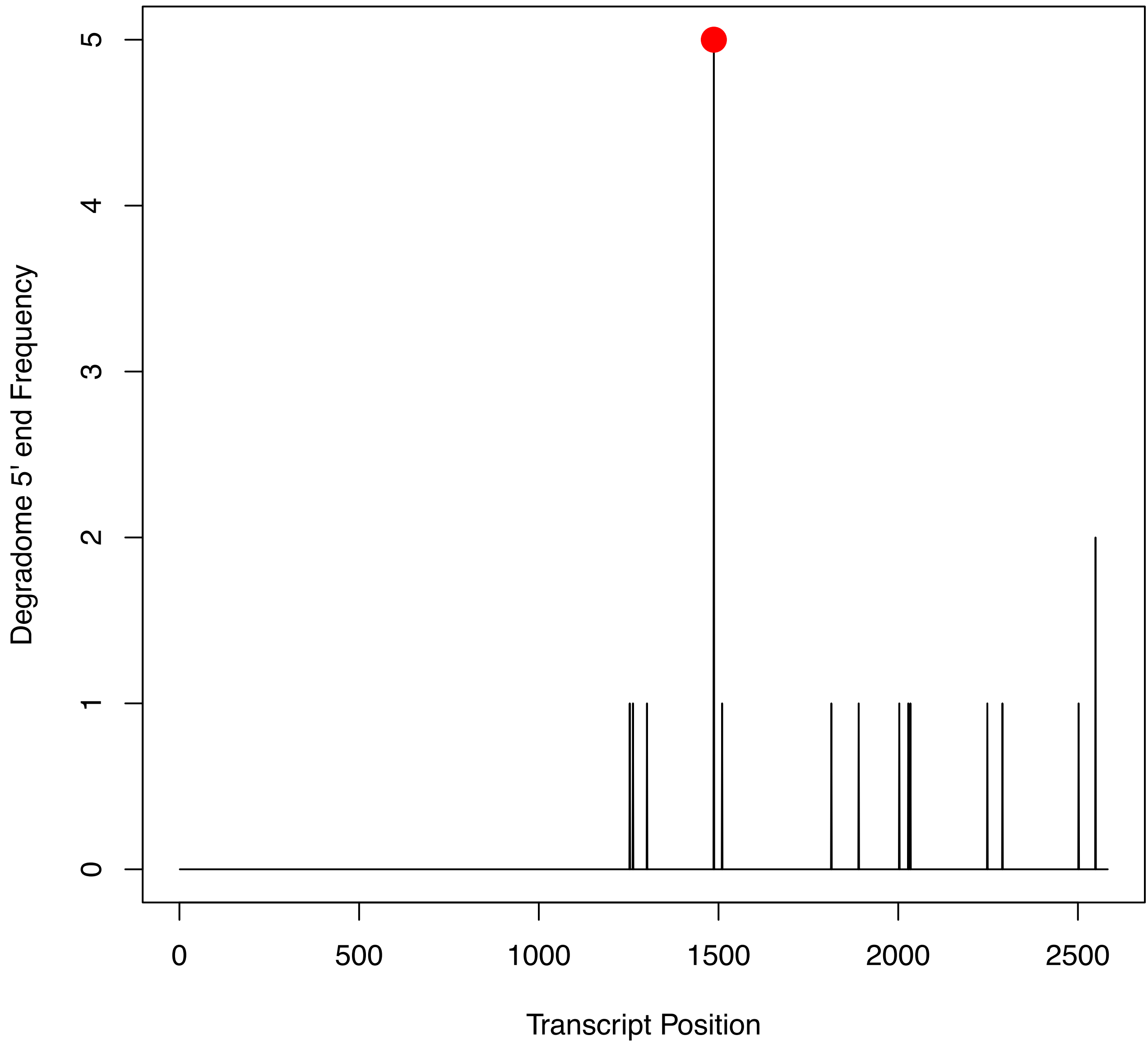

D=Day0
T=HORVU.MOREX.r3.1HG0011590.1
Q=novel–5p.Cluster_2558
S=1487
category=0
p=0.00490001091894188

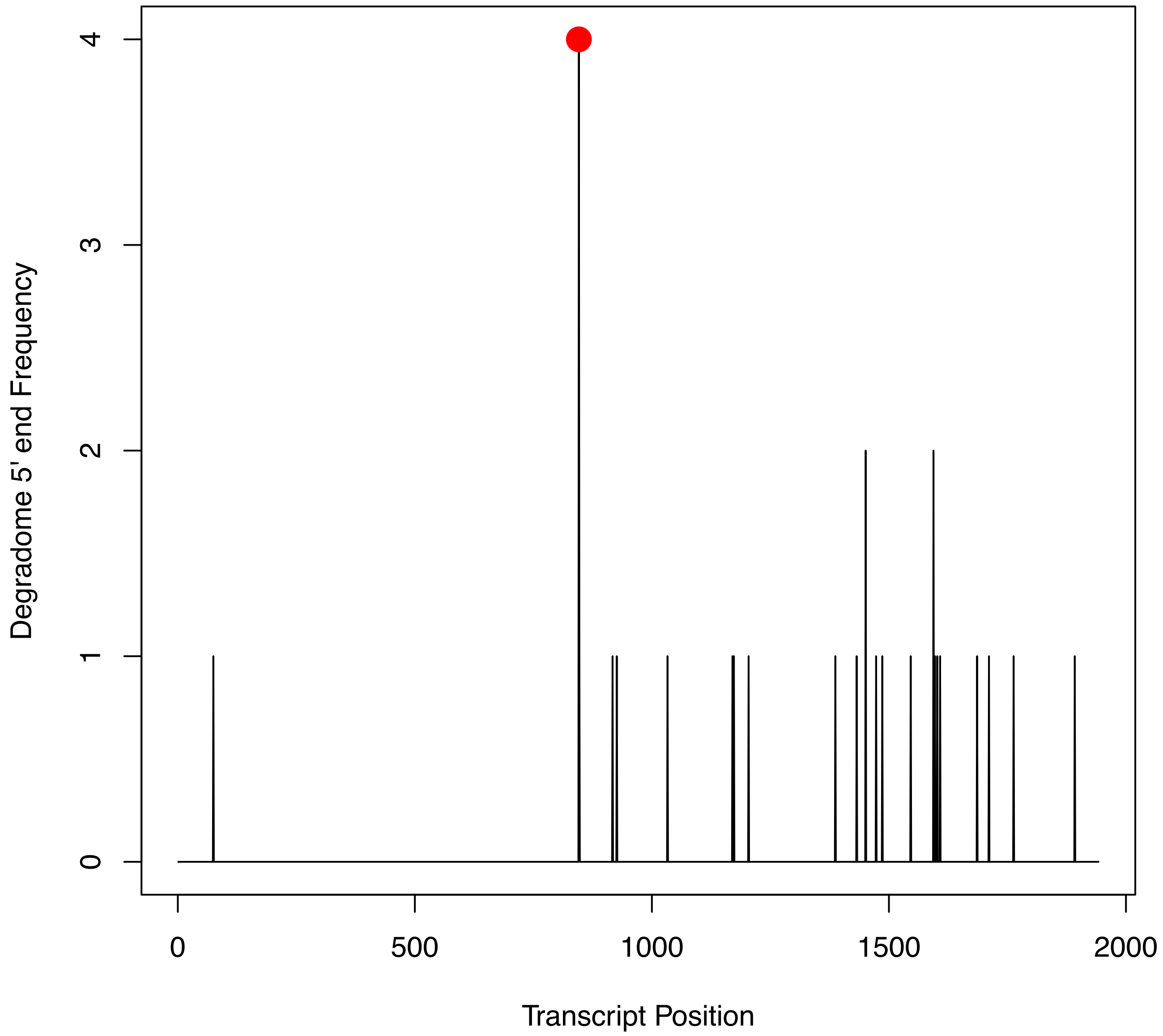

D=Day0
T=HORVU.MOREX.r3.1HG0011700.1
Q=novel–5p.Cluster_2558
S=846
category=0
p=0.00245301409855481

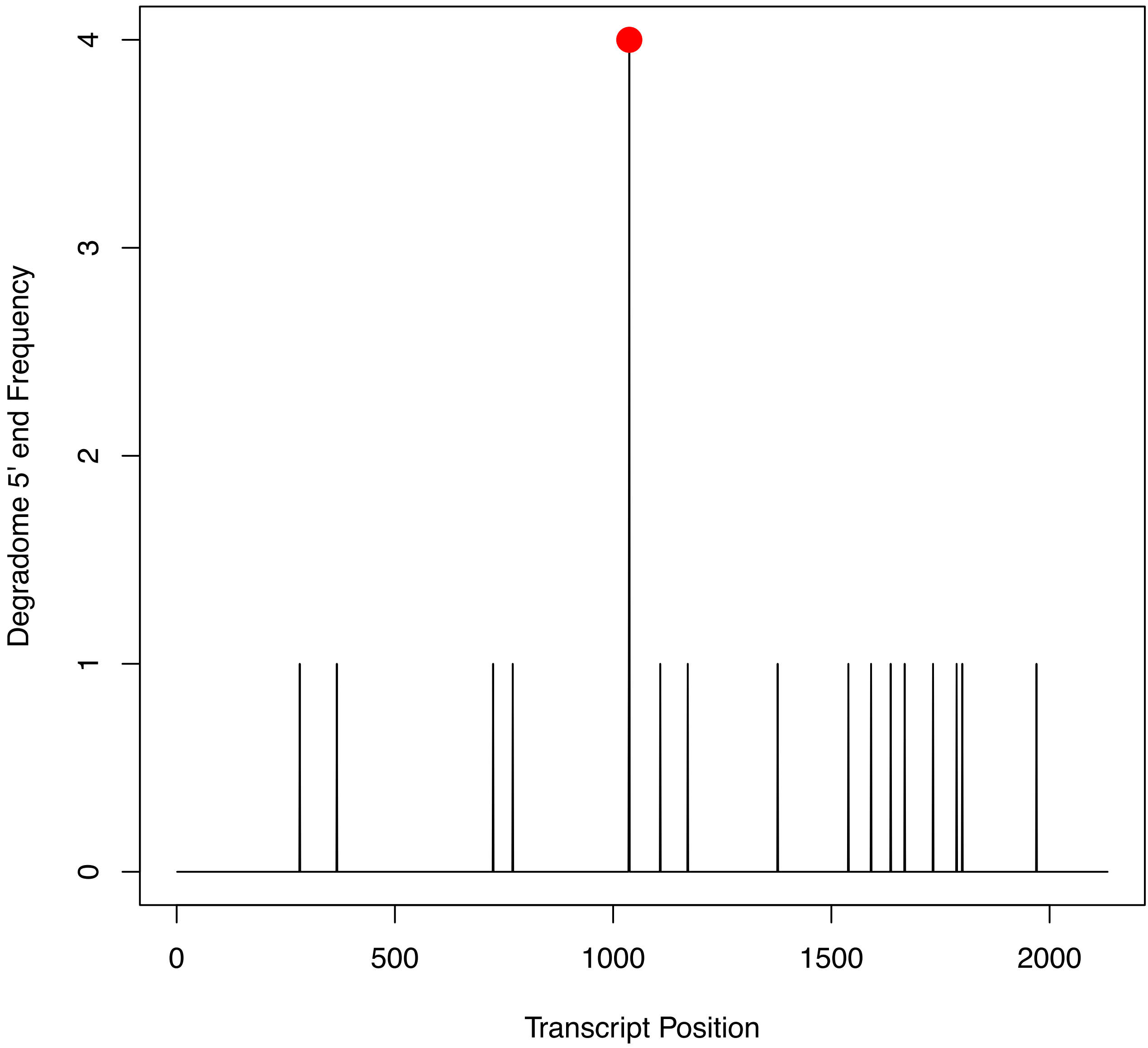

D=Day0
T=HORVU.MOREX.r3.1HG0011730.1
Q=novel–5p.Cluster_2558
S=1037
category=0
p=0.00367726374514032

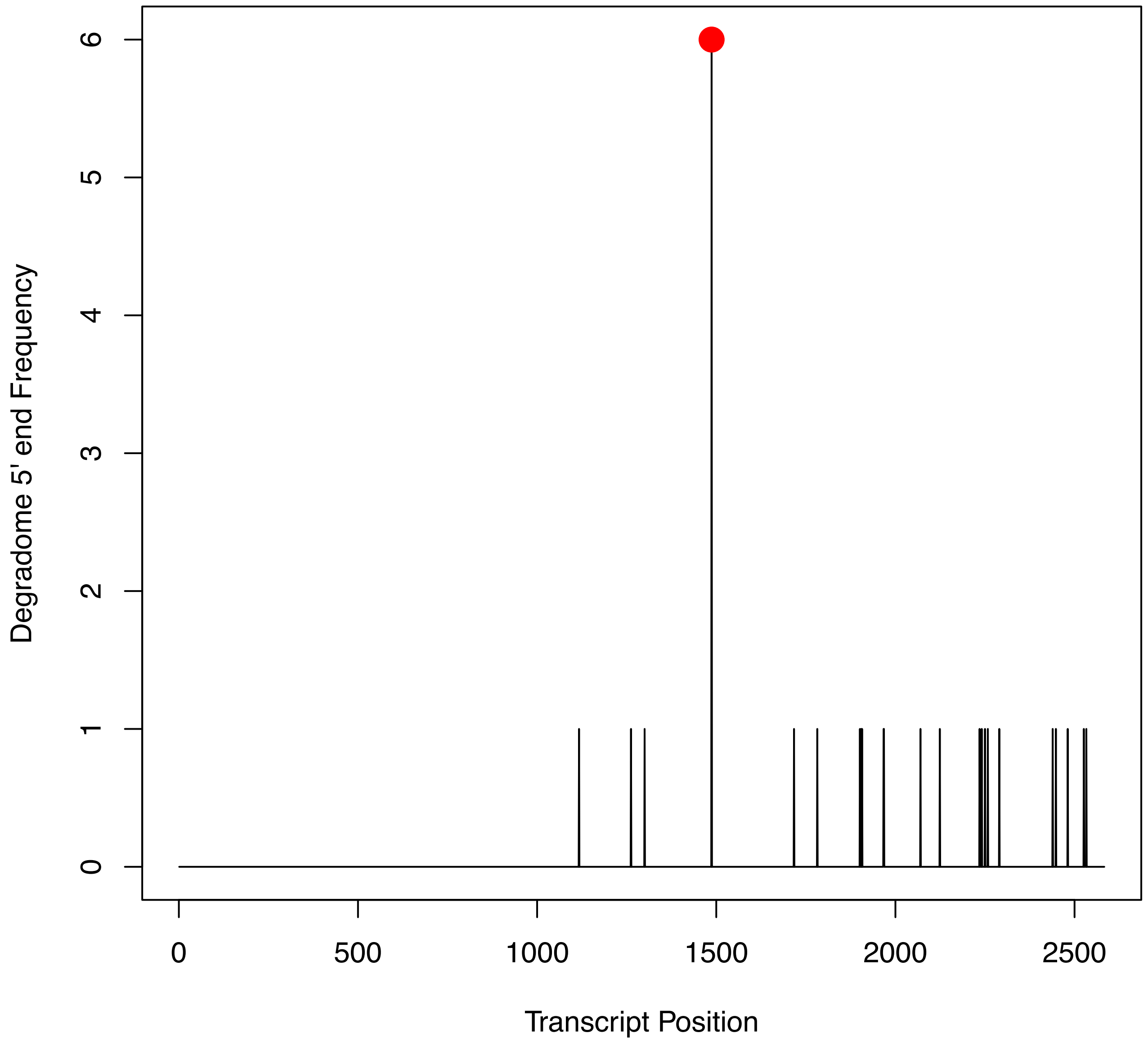

D=Day0
T=HORVU.MOREX.r3.1HG0011760.1
Q=novel–5p.Cluster_2558
S=1487
category=0
p=0.00326934756215558

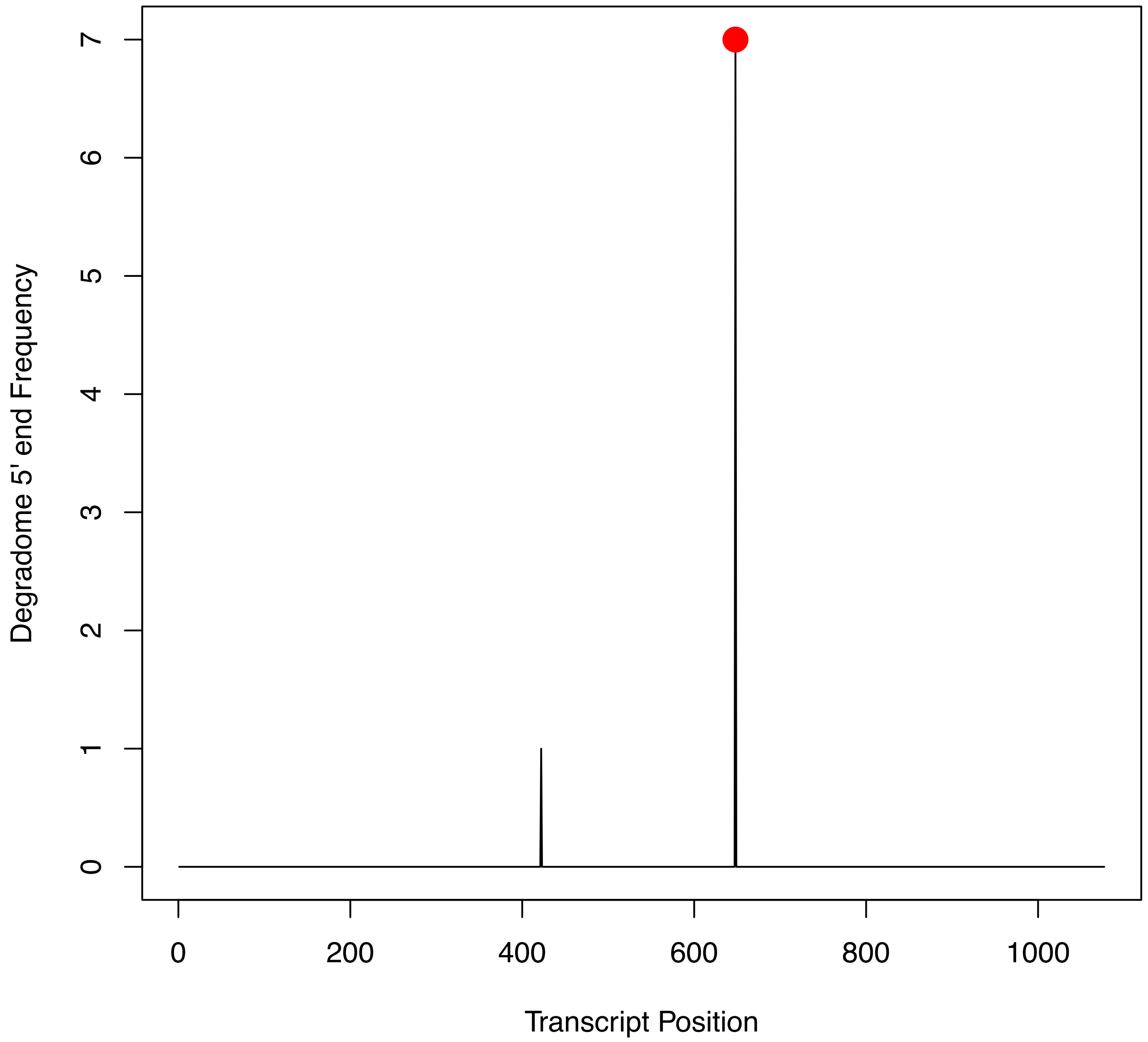

D=Day1
T=HORVU.MOREX.r3.2HG0189580.1
Q=miR156–5p.Cluster_1557.Cluster_1973.Cluster_4963
S=648
category=0
p=0.0016273260186368

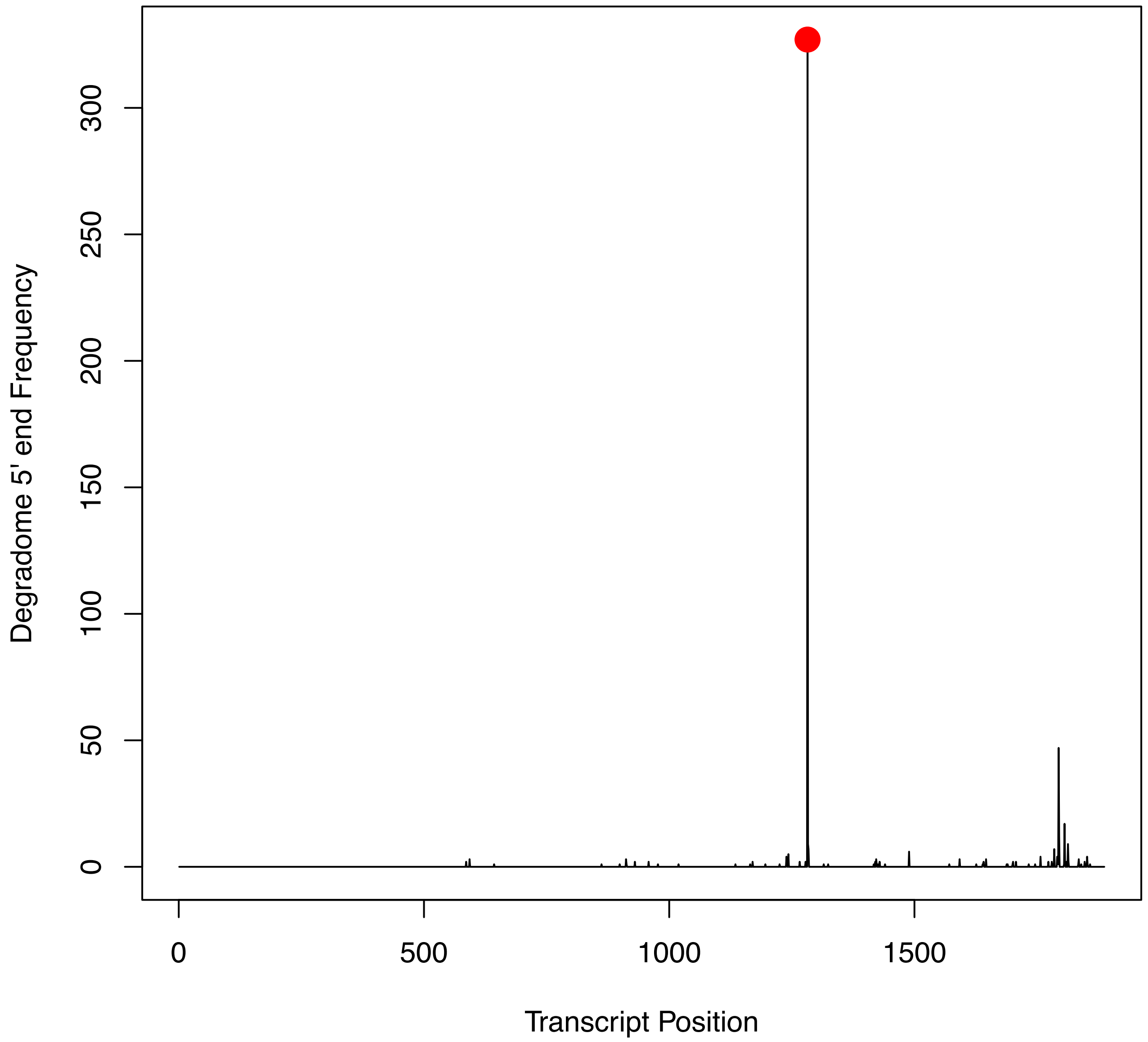

D=Day1
T=HORVU.MOREX.r3.3HG0310780.1
Q=miR156–5p.Cluster_1557.Cluster_1973.Cluster_4963
S=1282
category=0
p=0.0020337435751545

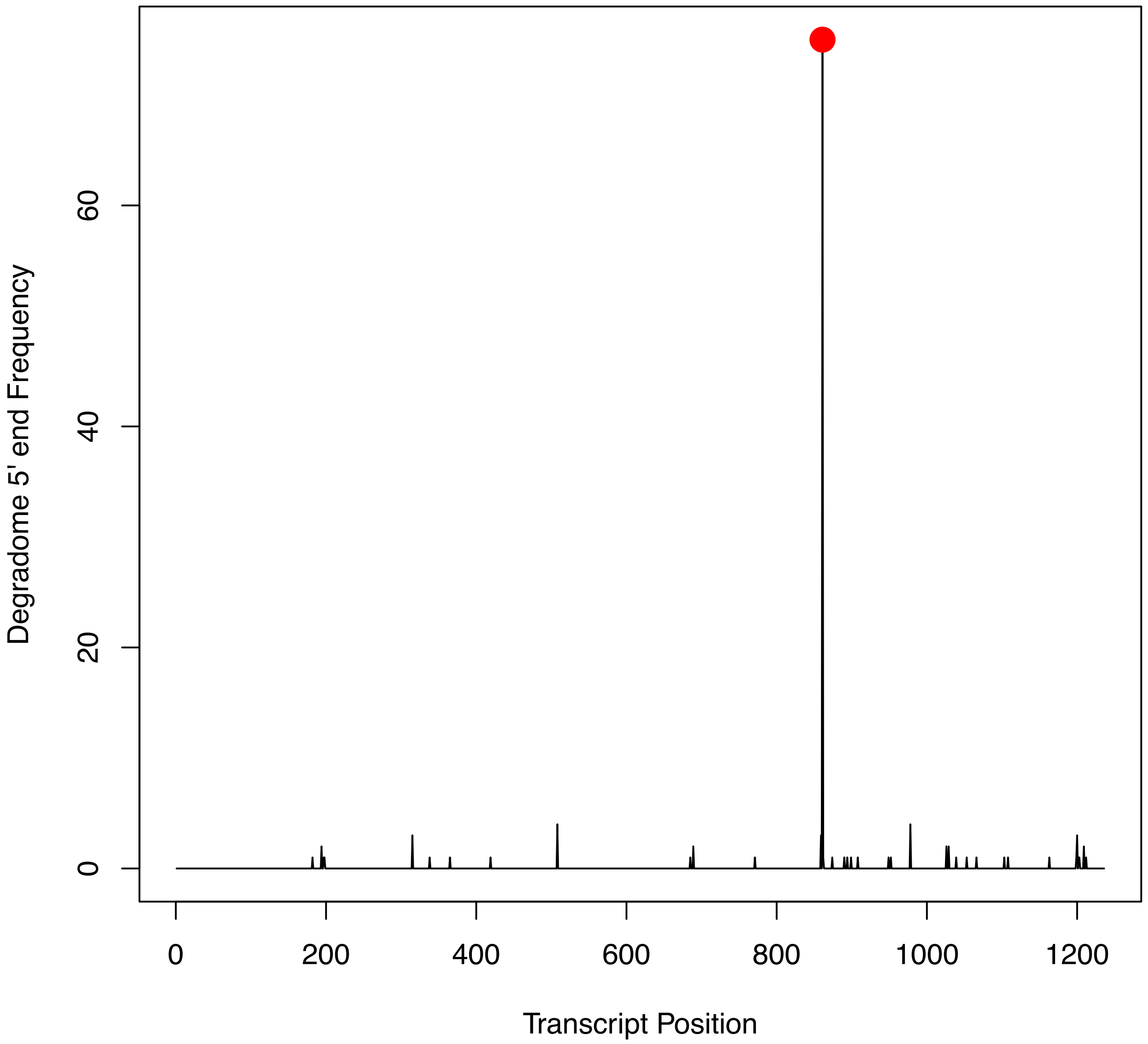

D=Day1
T=HORVU.MOREX.r3.5HG0490900.1
Q=miR156–5p.Cluster_1557.Cluster_1973.Cluster_4963
S=861
category=0
p=0.0012207429502803

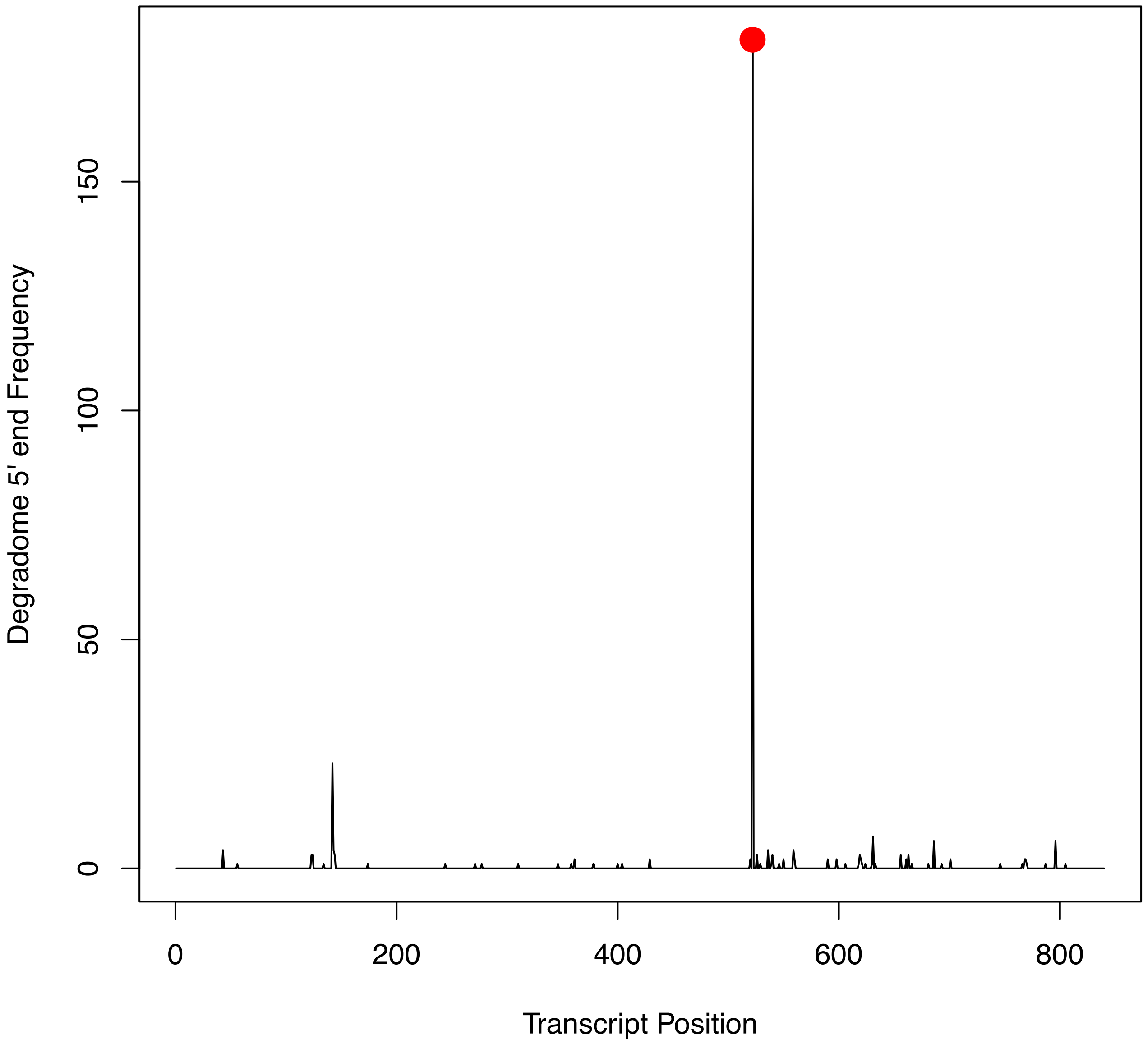

D=Day1
T=HORVU.MOREX.r3.5HG0494180.1
Q=miR156–5p.Cluster_1557.Cluster_1973.Cluster_4963
S=522
category=0
p=0.00284608242215212

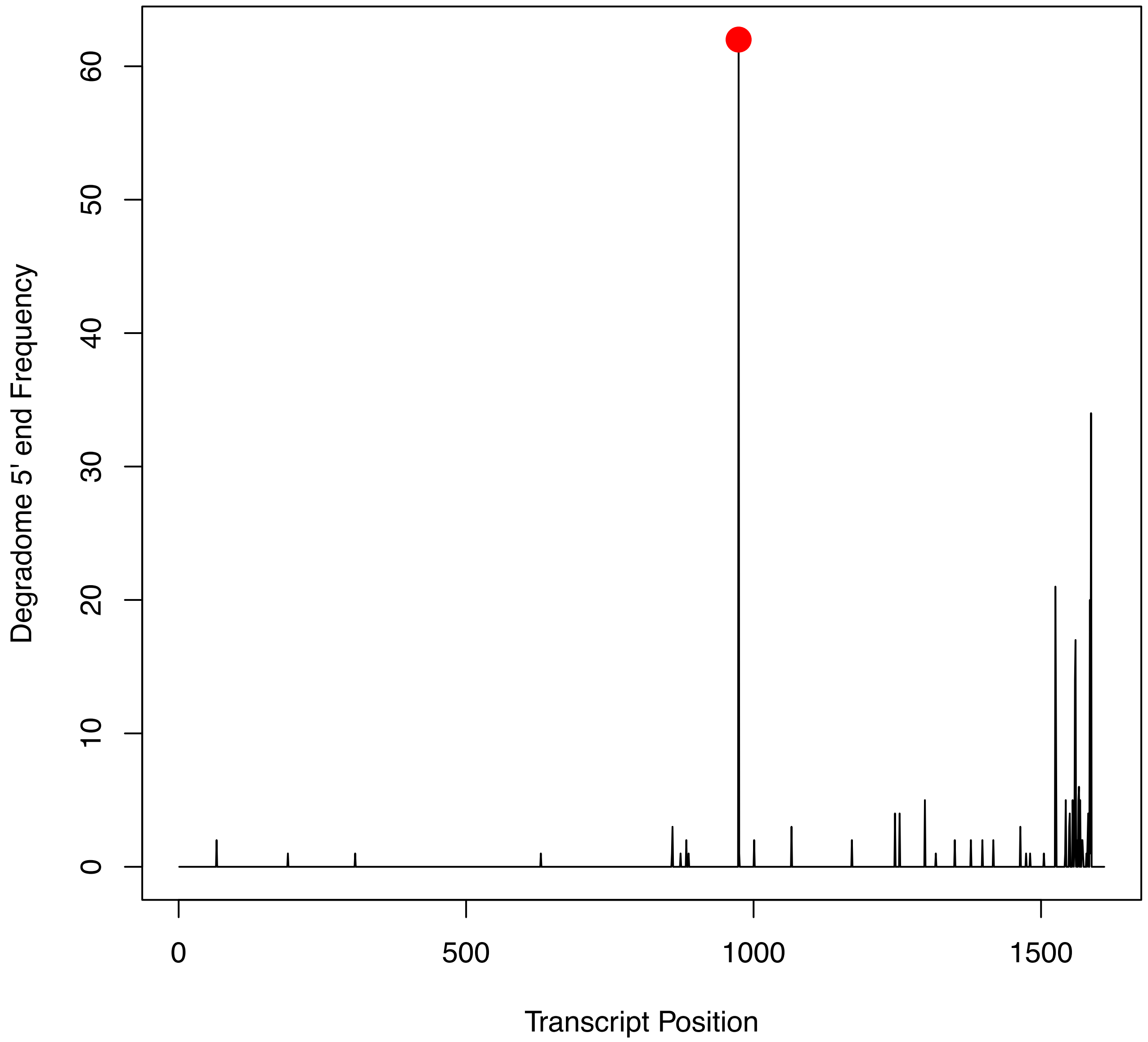

D=Day1
T=HORVU.MOREX.r3.7HG0679980.1
Q=miR156–5p.Cluster_1557.Cluster_1973.Cluster_4963
S=974
category=0
p=0.00243999568720987

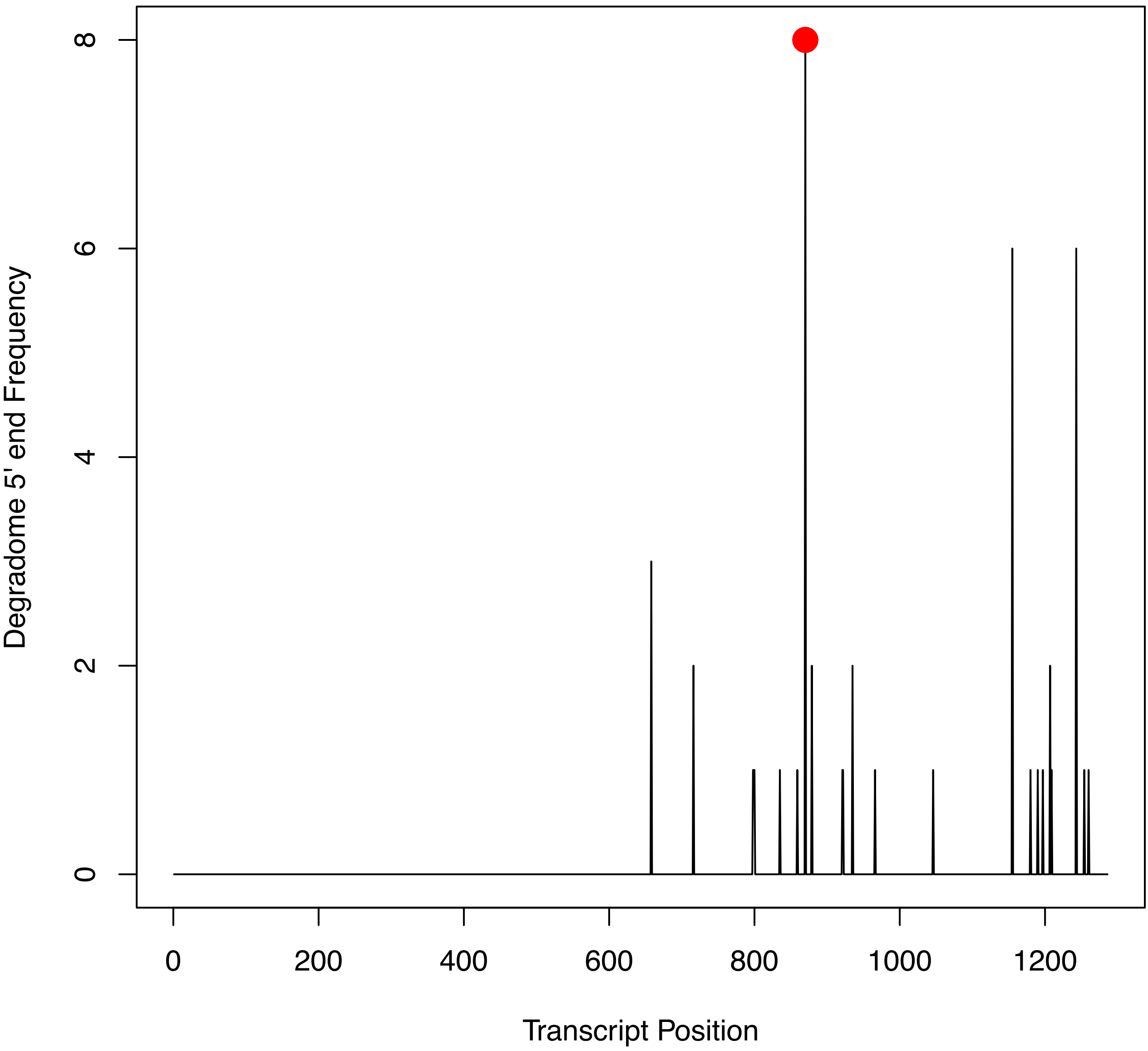

D=Day1
T=HORVU.MOREX.r3.7HG0684000.1
Q=miR156–5p.Cluster_1557.Cluster_1973.Cluster_4963
S=870
category=0
p=0.000813994302680809

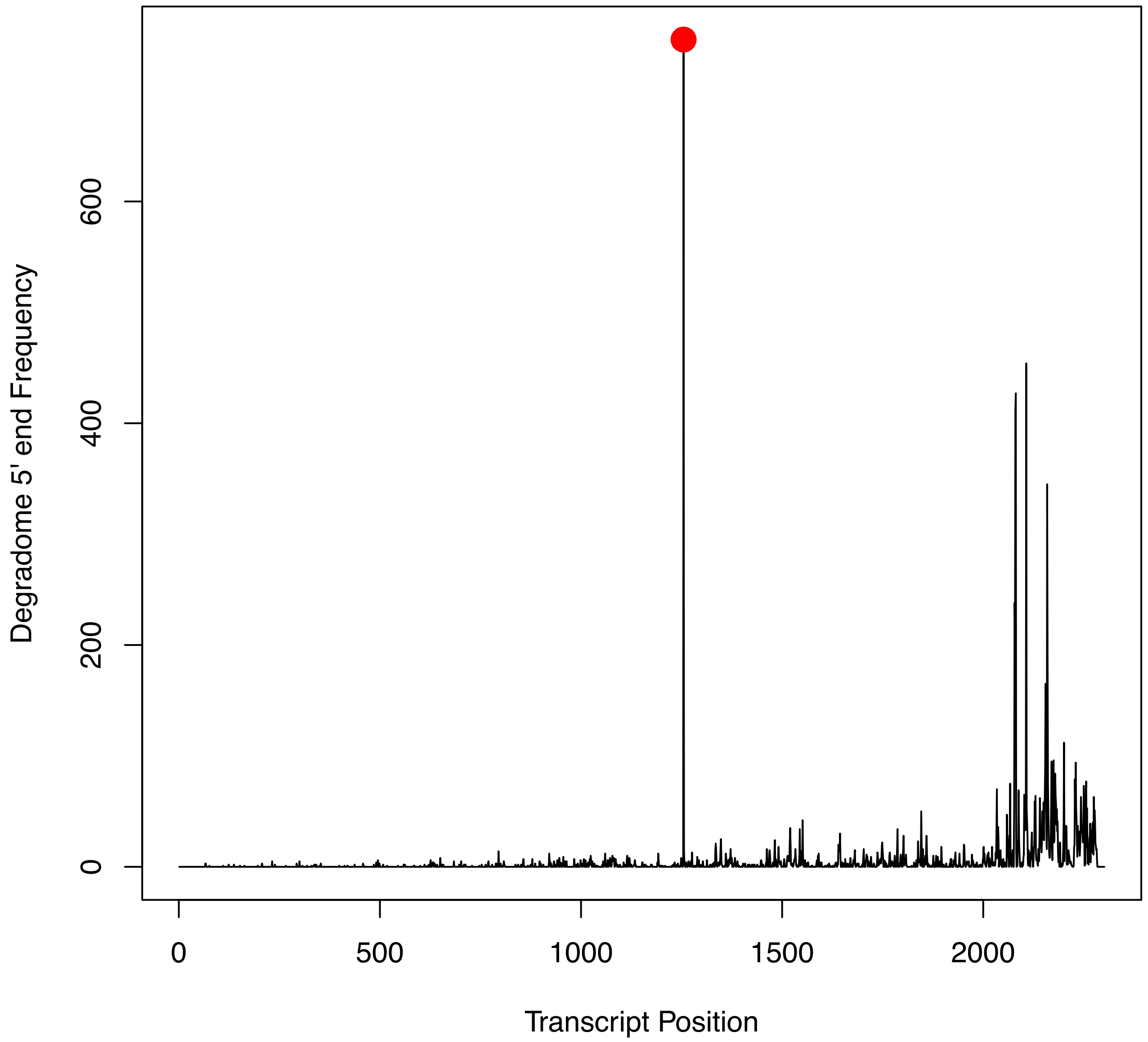

D=Day1
T=HORVU.MOREX.r3.3HG0296070.1
Q=miR159–3p.Cluster_1875
S=1255
category=0
p=0.0012207429502803

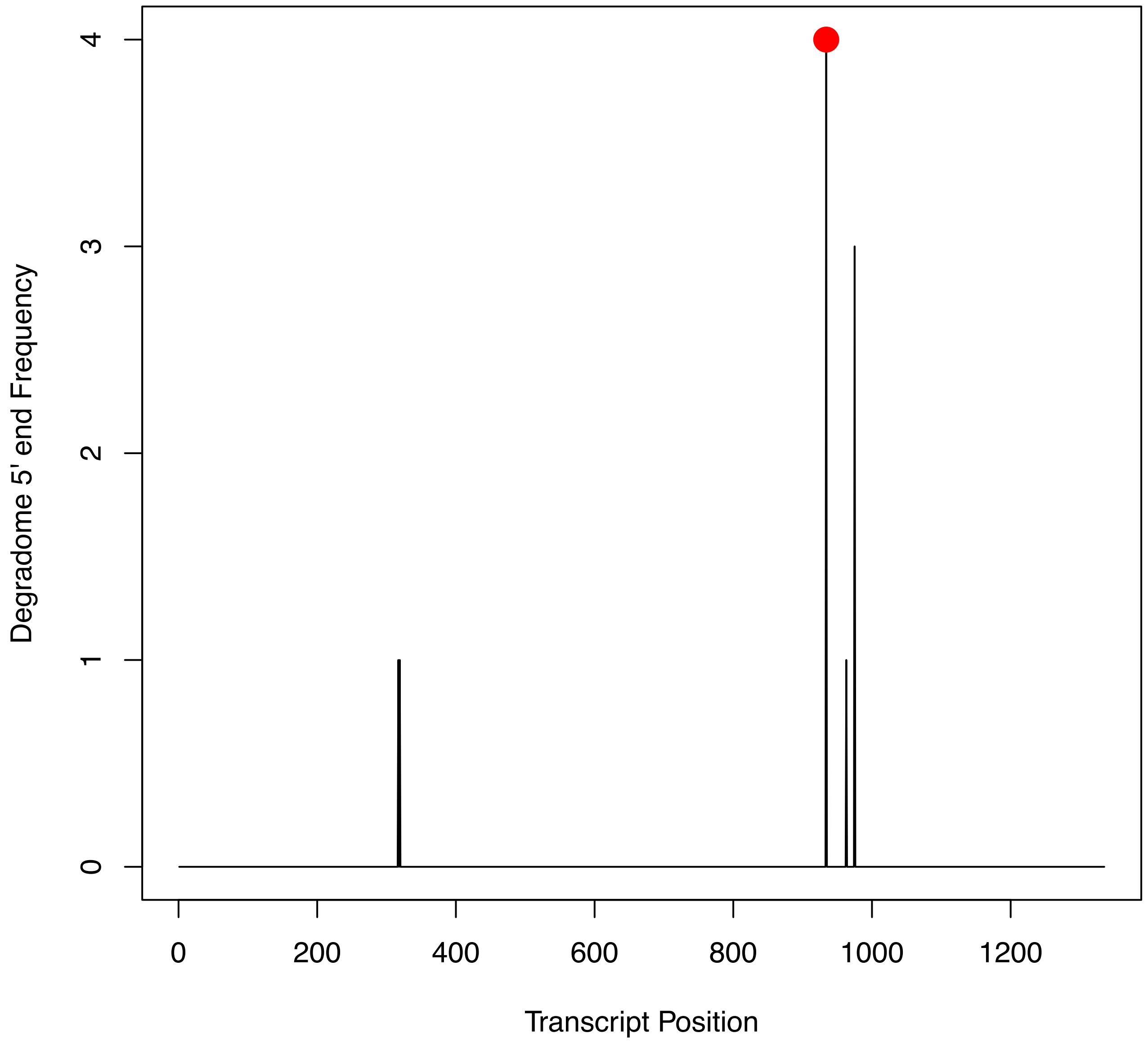

D=Day1
T=HORVU.MOREX.r3.7HG0719850.1
Q=miR159–3p.Cluster_1875
S=934
category=0
p=0.000813994302680809

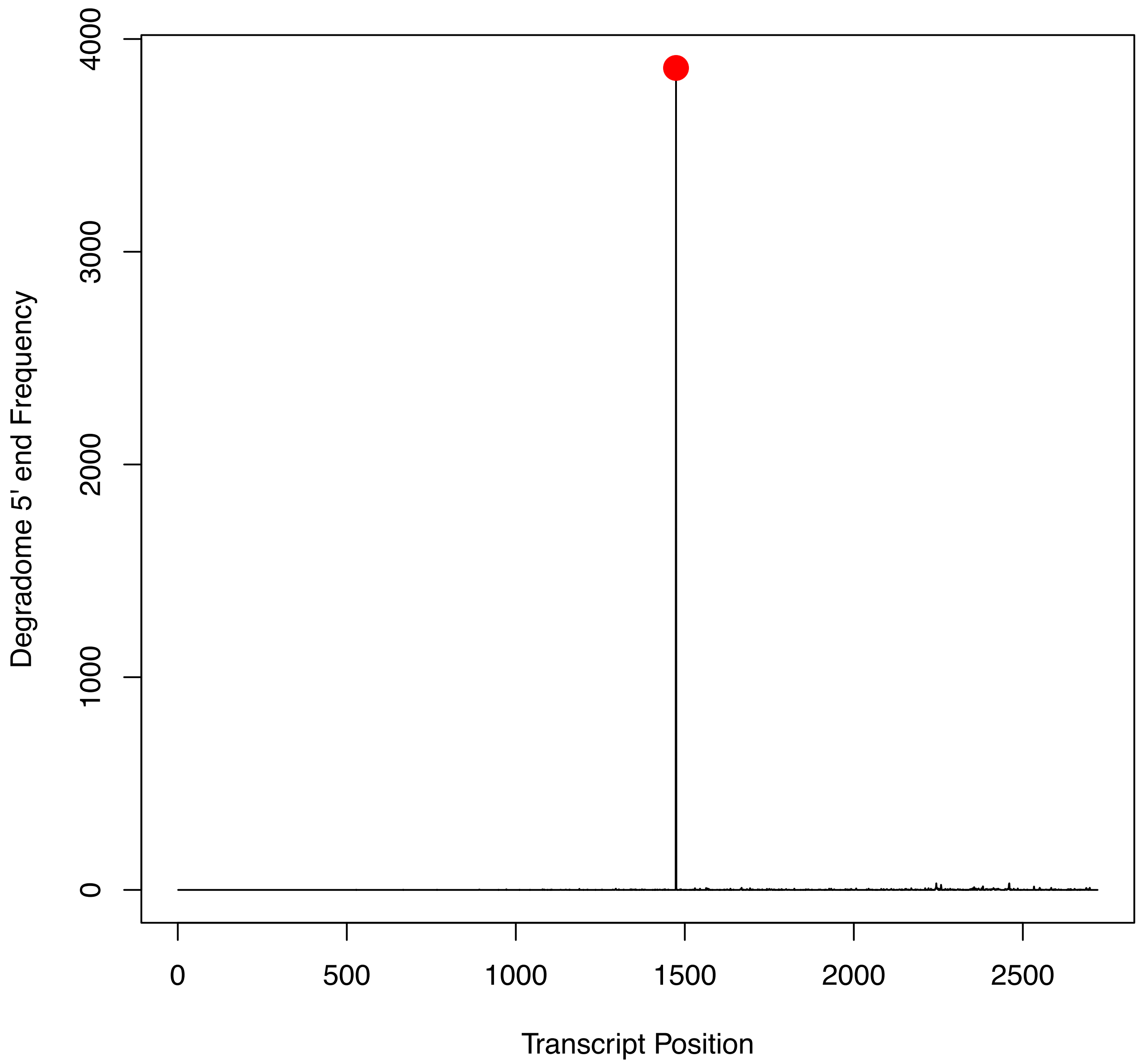

D=Day1
T=HORVU.MOREX.r3.2HG0182280.1
Q=miR160–5p.Cluster_6224
S=1474
category=0
p=0.00243999568720987

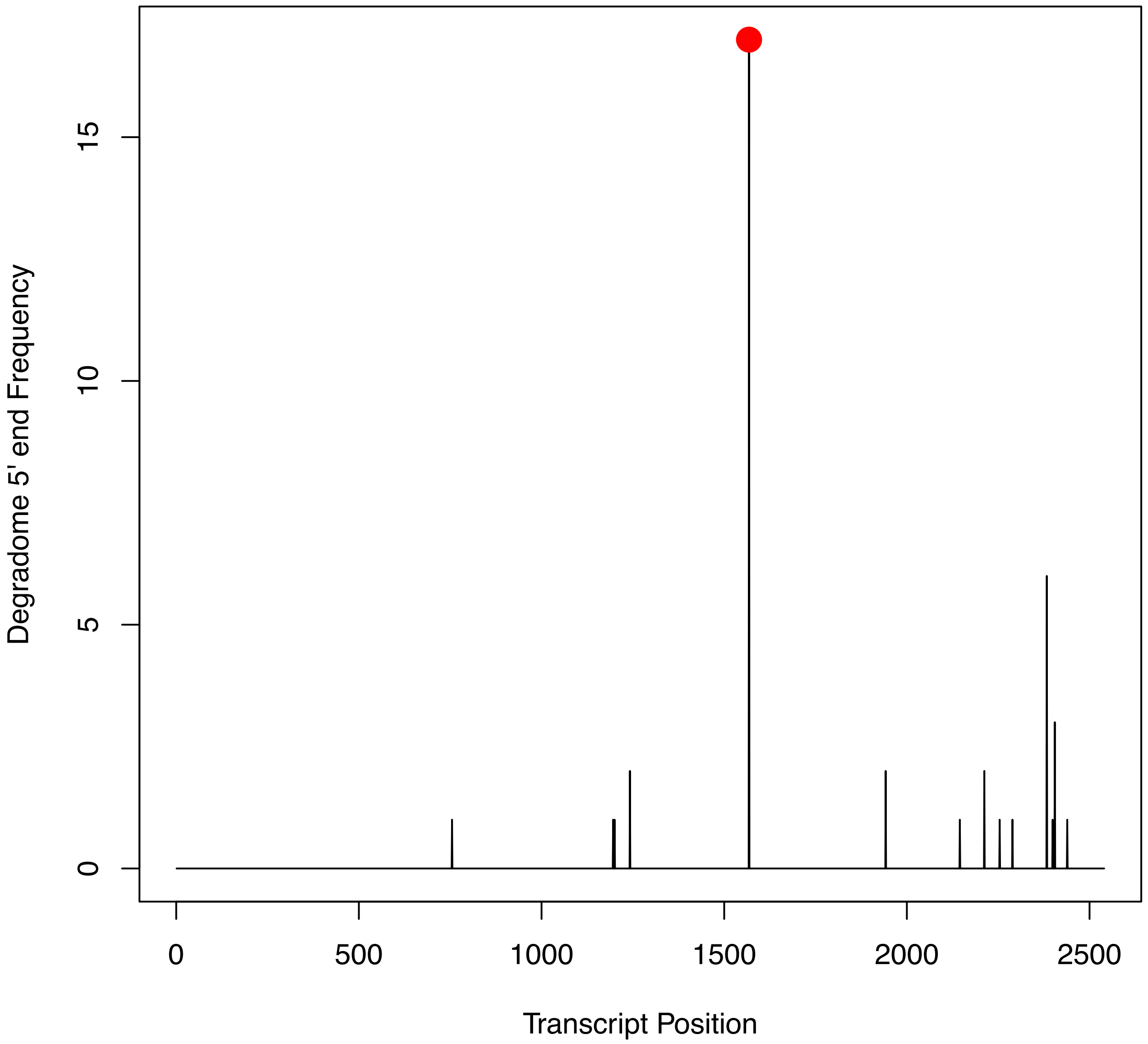

D=Day1
T=HORVU.MOREX.r3.6HG0596670.1
Q=miR160–5p.Cluster_6224
S=1568
category=0
p=0.000407080008407057

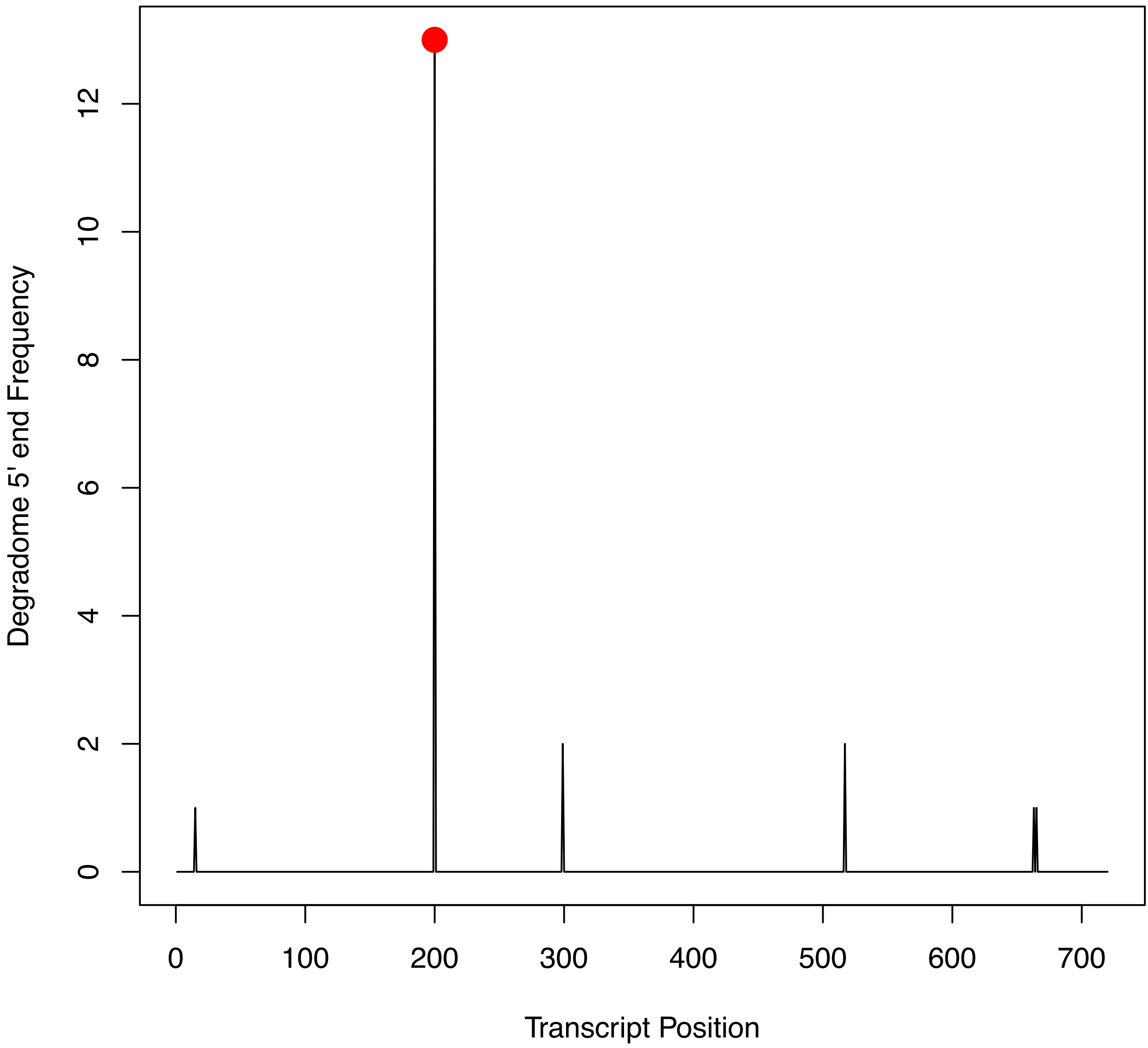

D=Day1
T=HORVU.MOREX.r3.2HG0139920.1
Q=miR166–3p.Cluster_426.Cluster_3396
S=200
category=0
p=0.00243999568720987

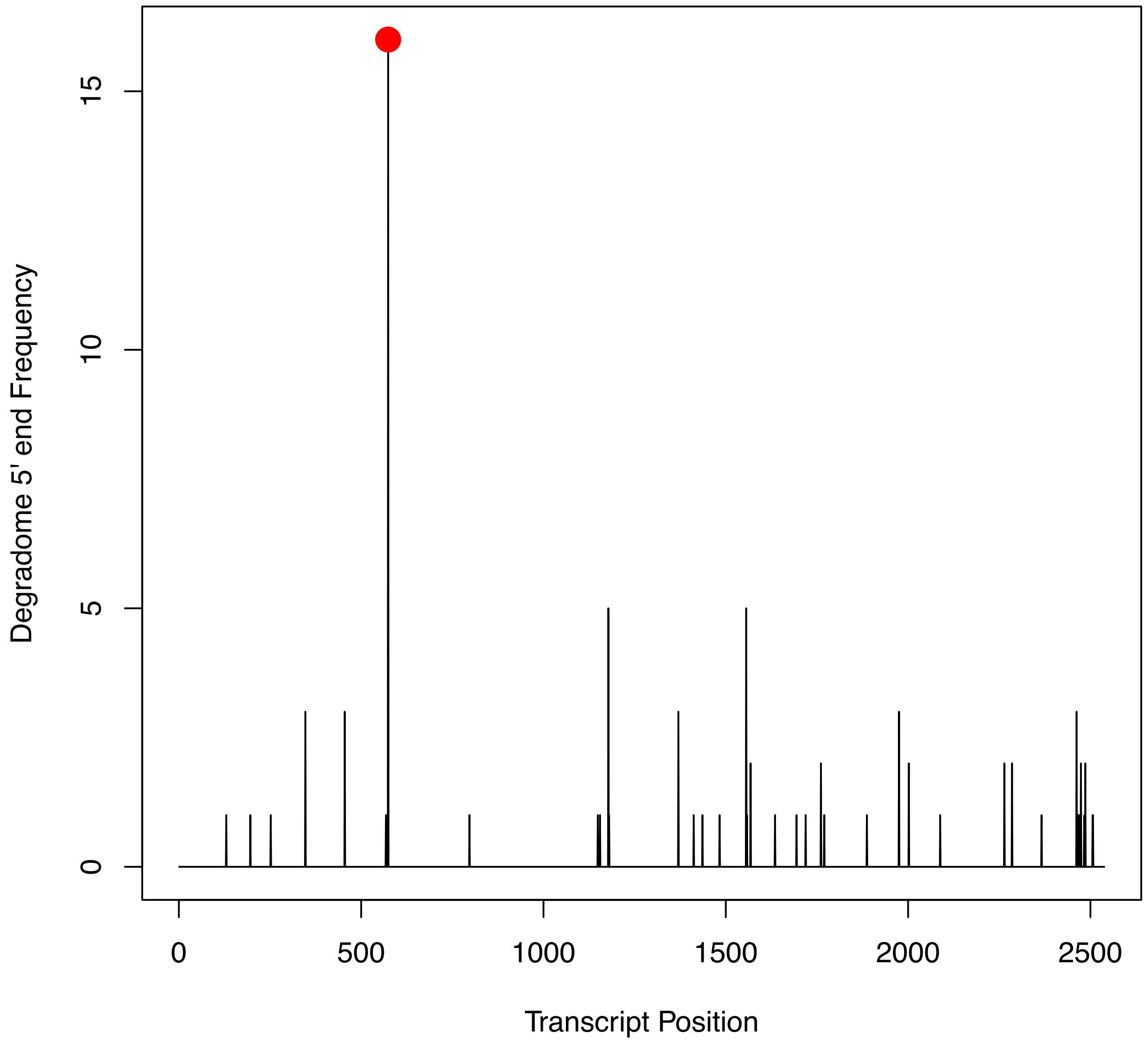

D=Day1
T=HORVU.MOREX.r3.3HG0244080.1
Q=miR166–3p.Cluster_426.Cluster_3396
S=574
category=0
p=0.0012207429502803

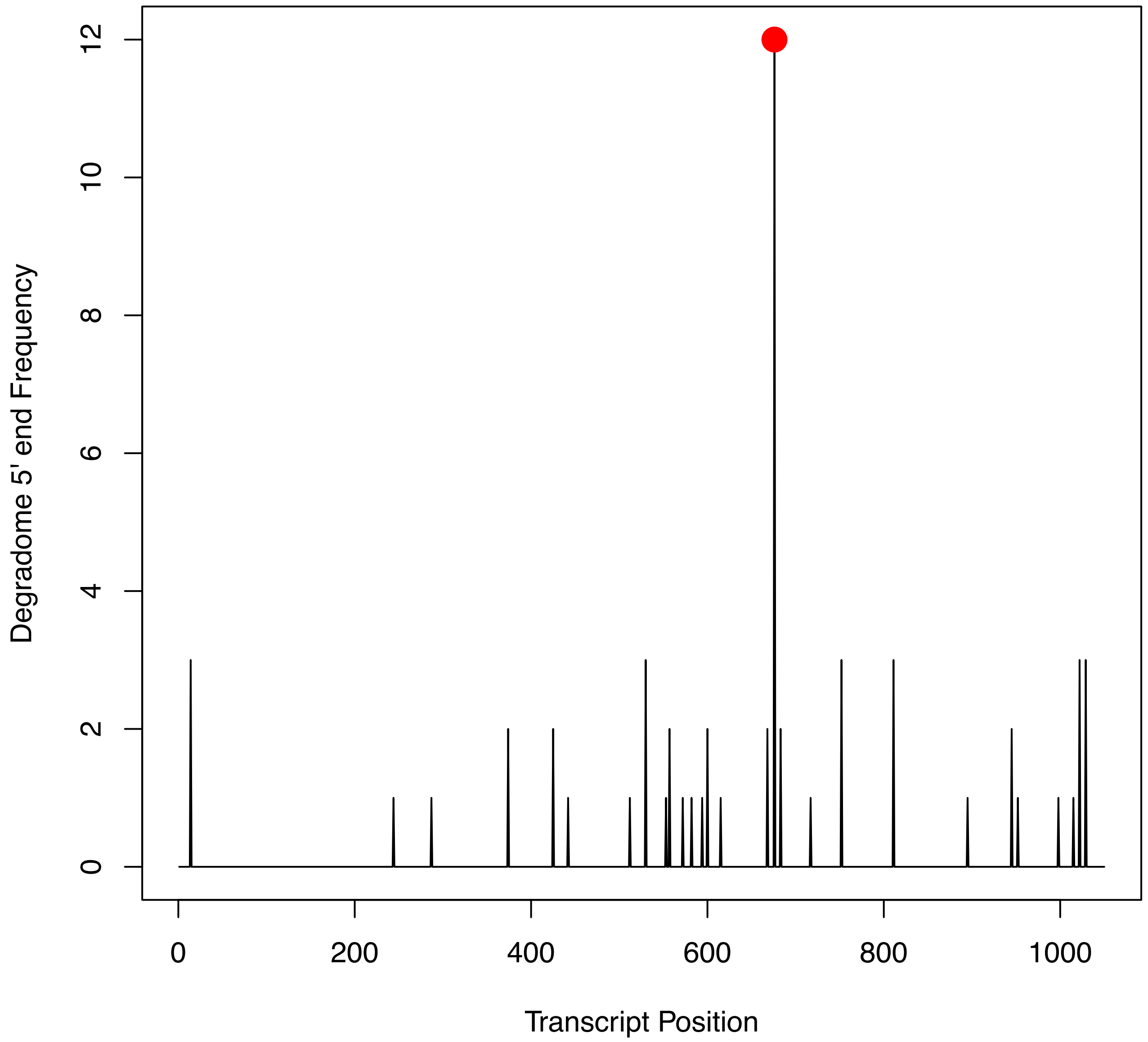

D=Day1
T=HORVU.MOREX.r3.7HG0698540.1
Q=miR167–5p.Cluster_3392.Cluster_3623
S=676
category=0
p=0.00972431934650086

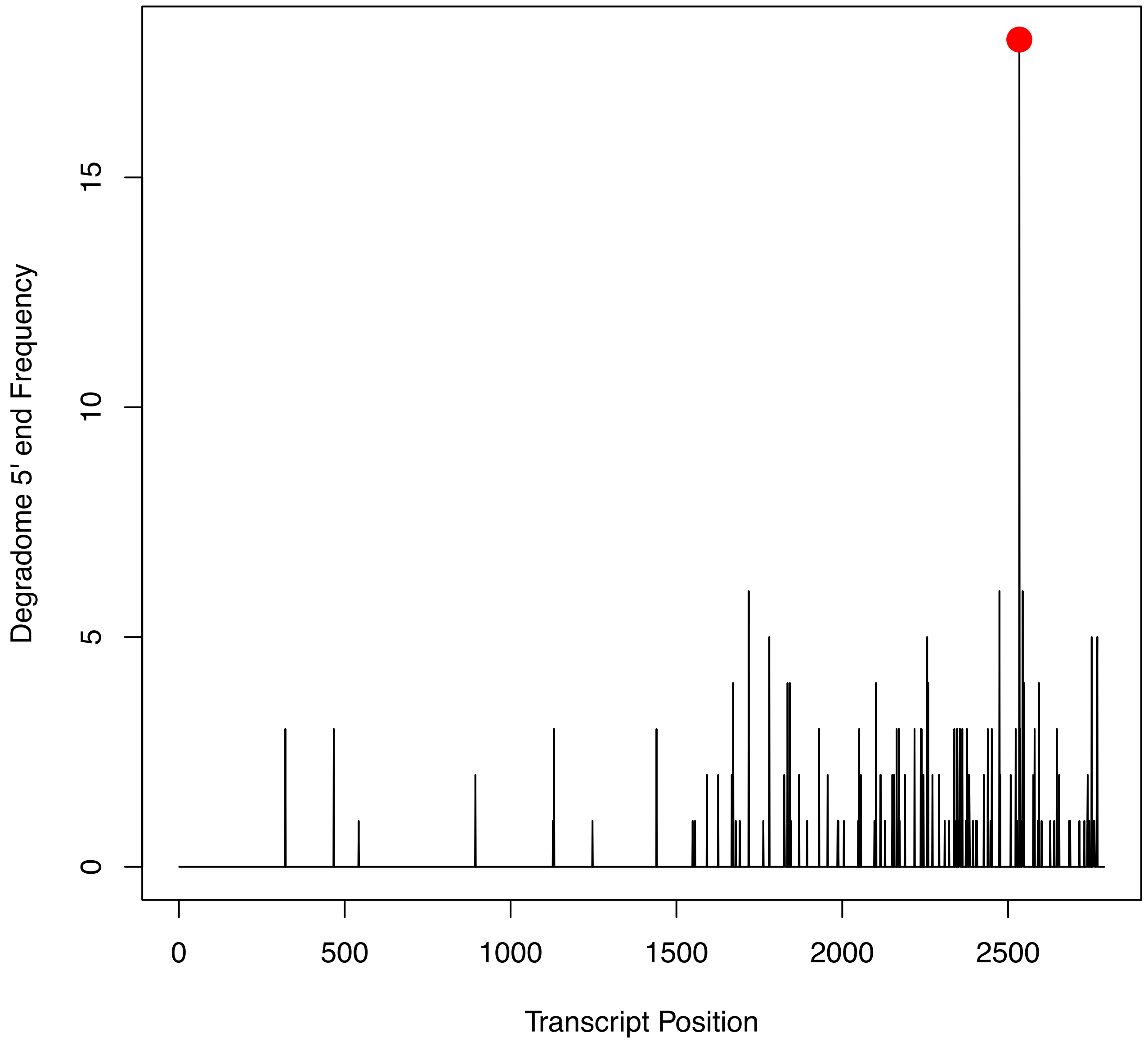

D=Day1
T=HORVU.MOREX.r3.6HG0566320.1
Q=miR167–5p.Cluster_4244
S=2534
category=0
p=0.00813994302680809

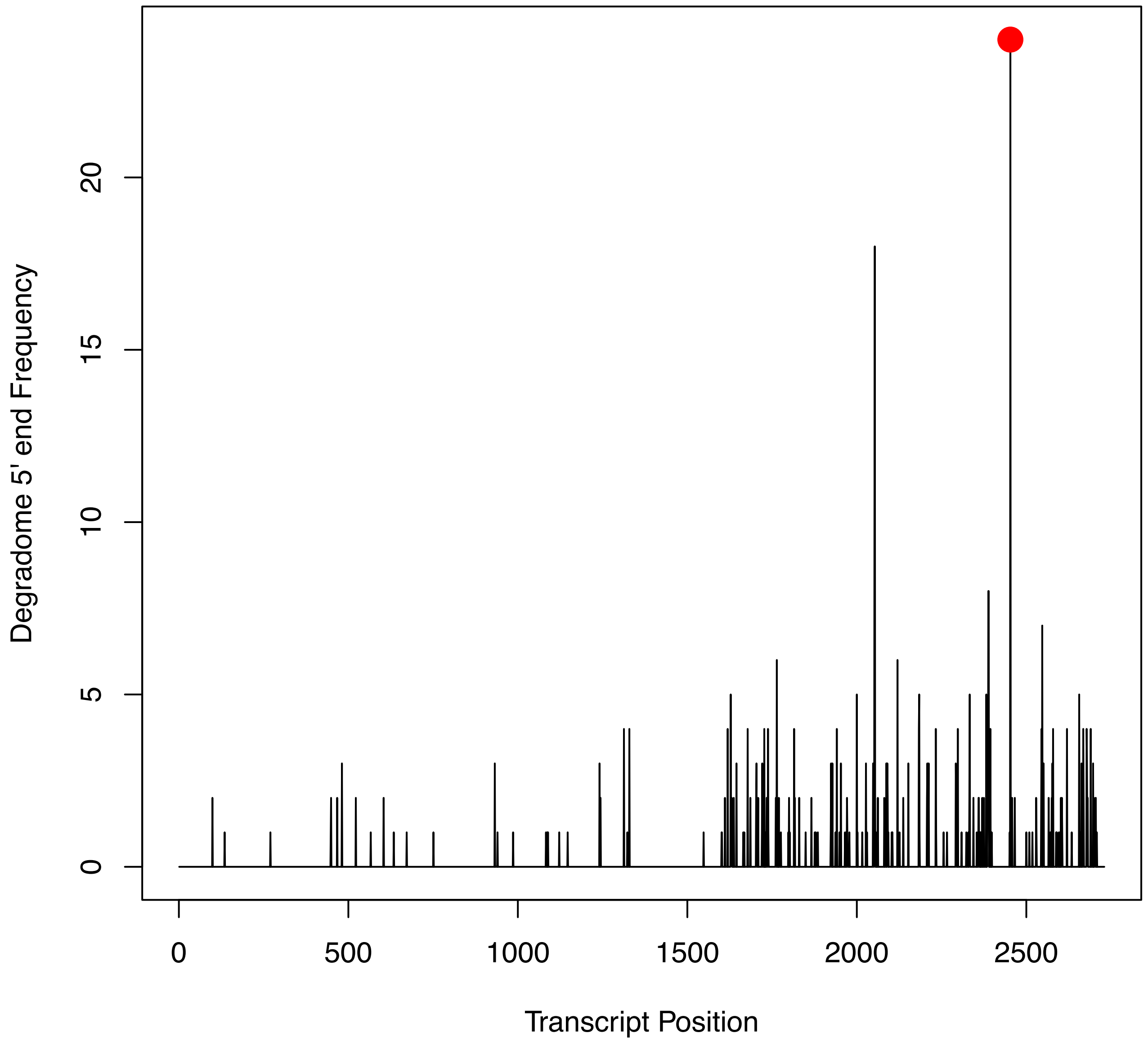

D=Day1
T=HORVU.MOREX.r3.7HG0735280.1
Q=miR167–5p.Cluster_4244
S=2453
category=0
p=0.0012207429502803

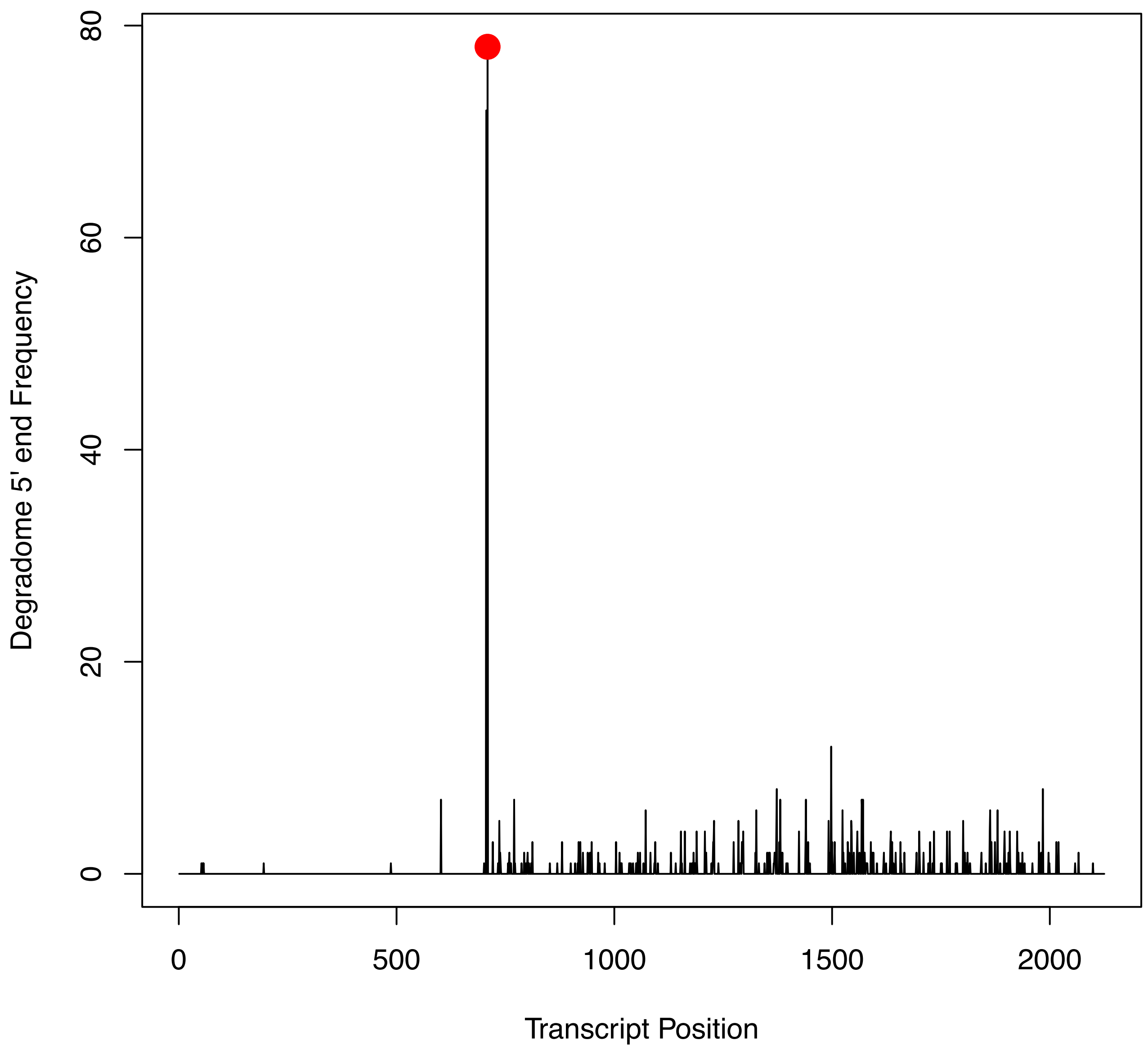

D=Day1
T=HORVU.MOREX.r3.7HG0635740.1
Q=miR171–3p.Cluster_456
S=709
category=0
p=0.000407080008407057

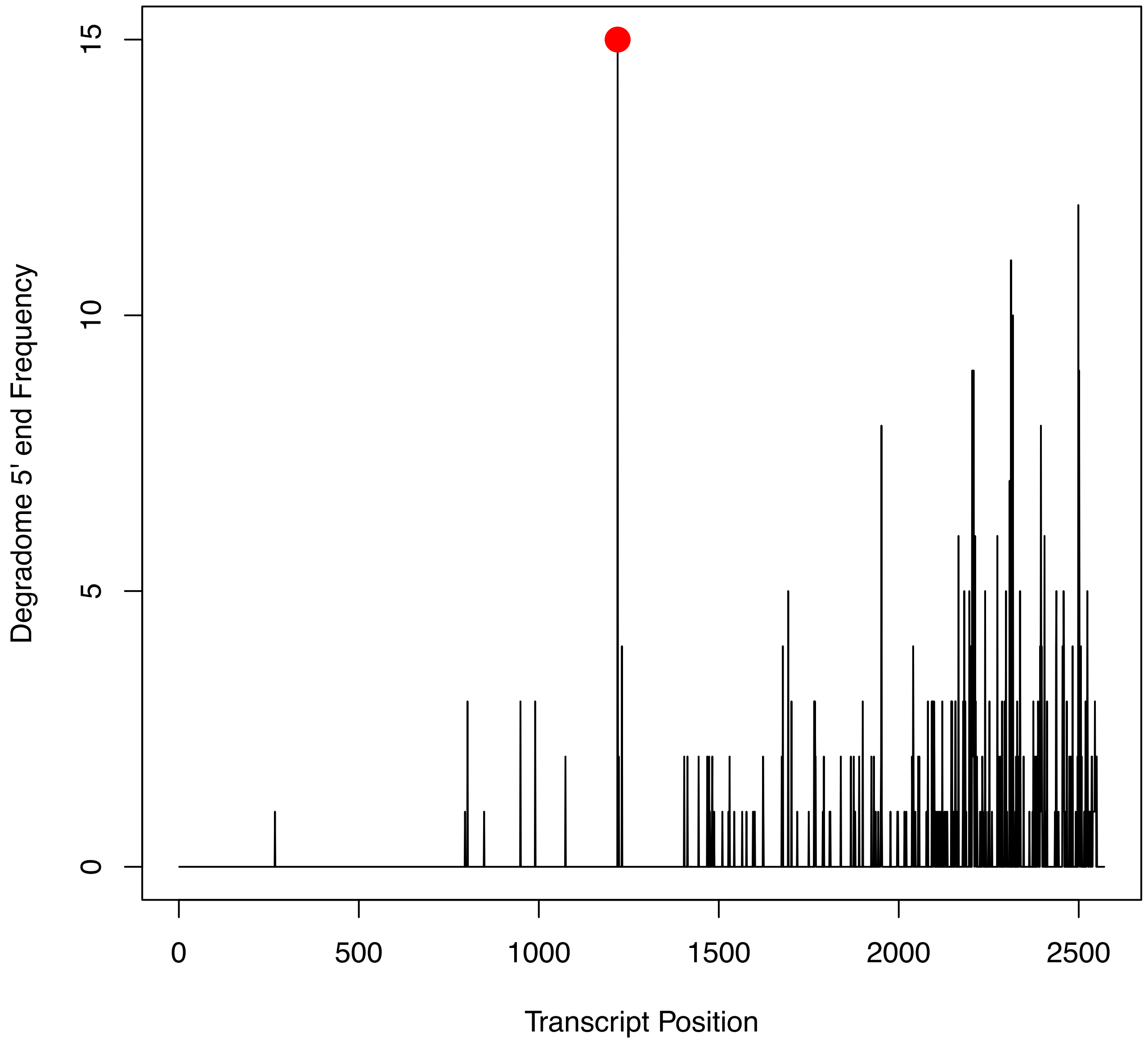

D=Day1
T=HORVU.MOREX.r3.1HG0055960.1
Q=miR171–3p.Cluster_1682
S=1219
category=0
p=0.0016273260186368

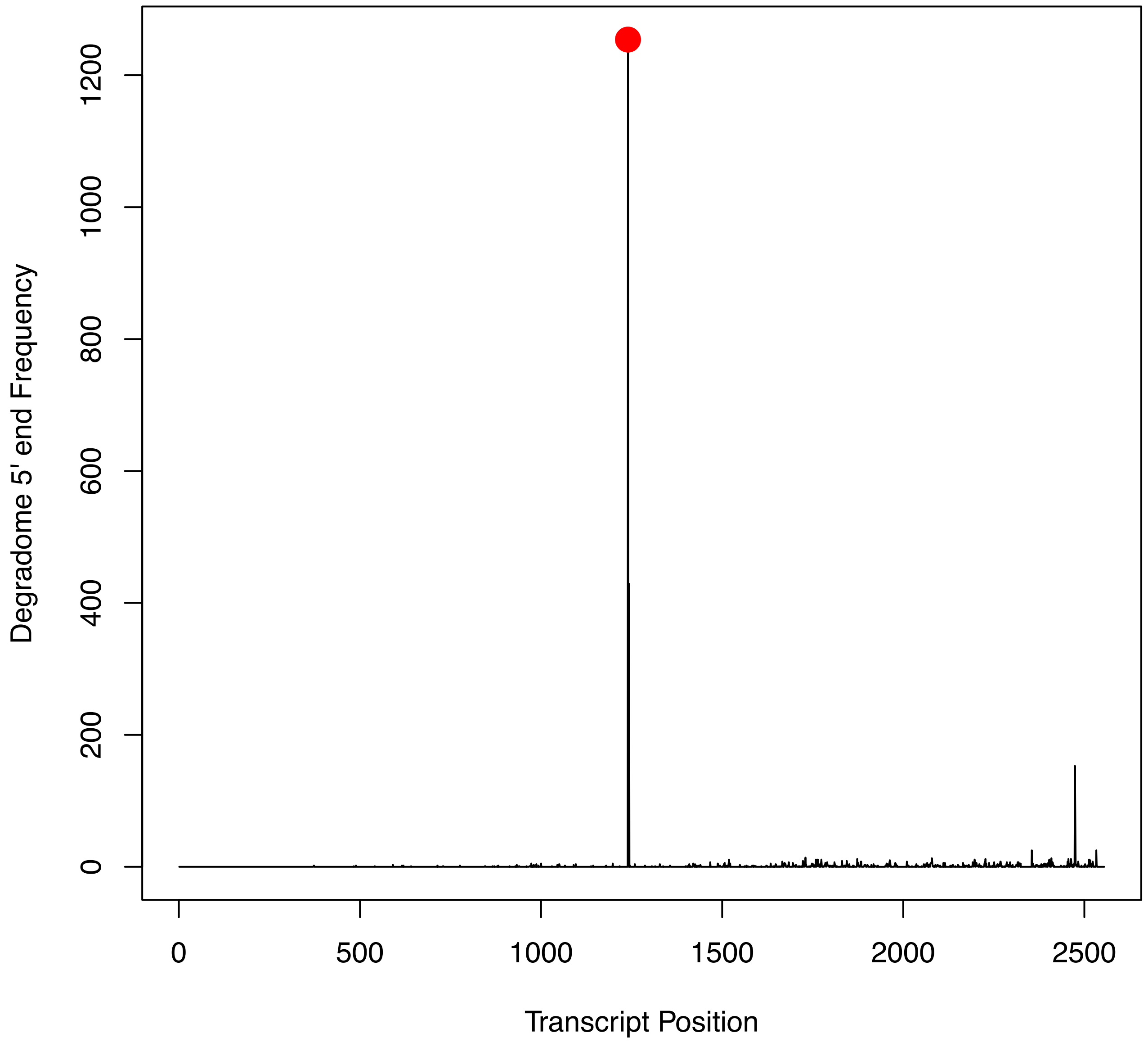

D=Day1
T=HORVU.MOREX.r3.6HG0601750.1
Q=miR171–3p.Cluster_1682
S=1240
category=0
p=0.00813994302680809

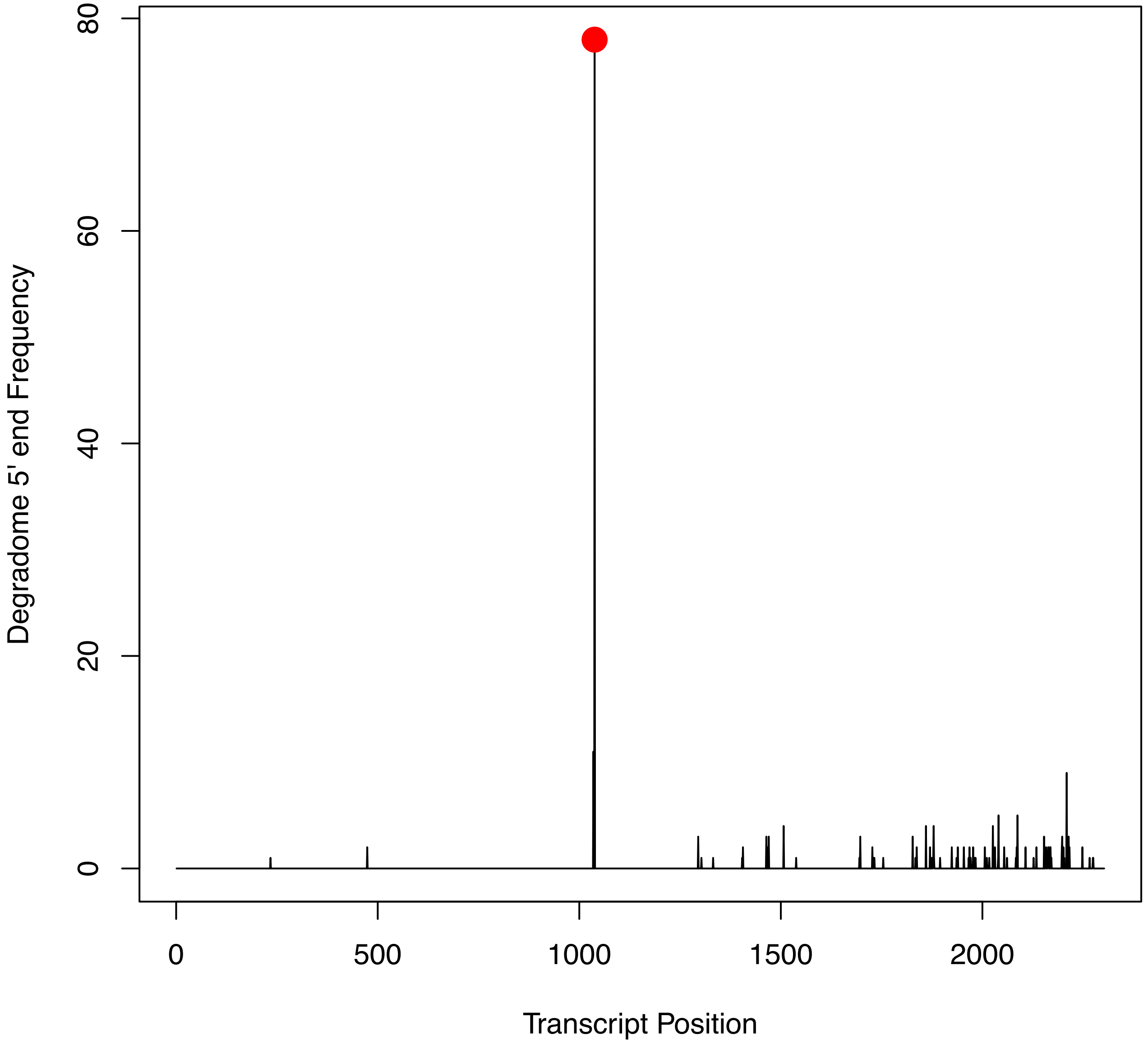

D=Day1
T=HORVU.MOREX.r3.4HG0415480.1
Q=miR171–3p.Cluster_3461
S=1038
category=0
p=0.000407080008407057

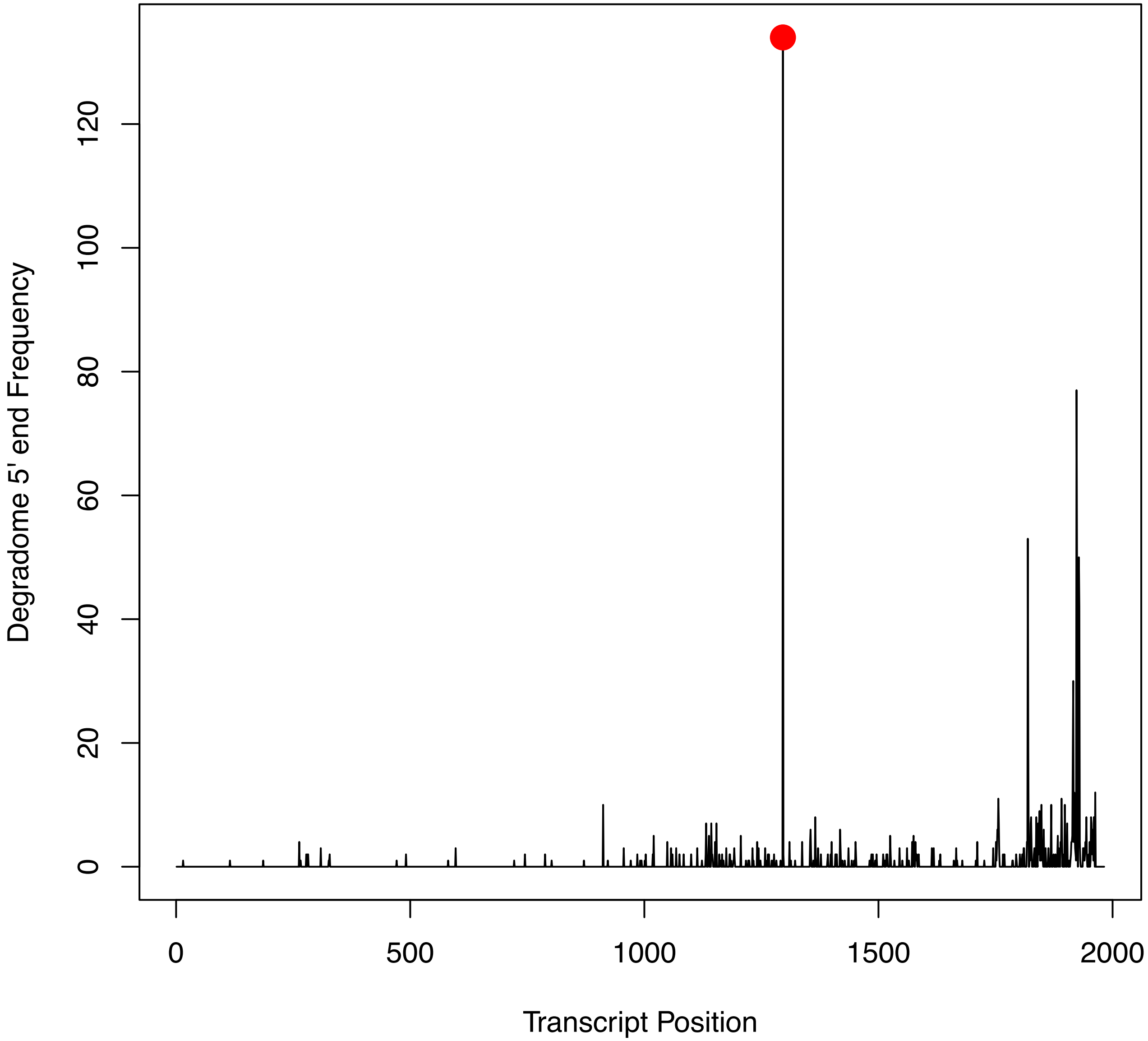

D=Day1
T=HORVU.MOREX.r3.2HG0152890.1
Q=miR319–3p.Cluster_2046
S=1296
category=0
p=0.000813994302680809

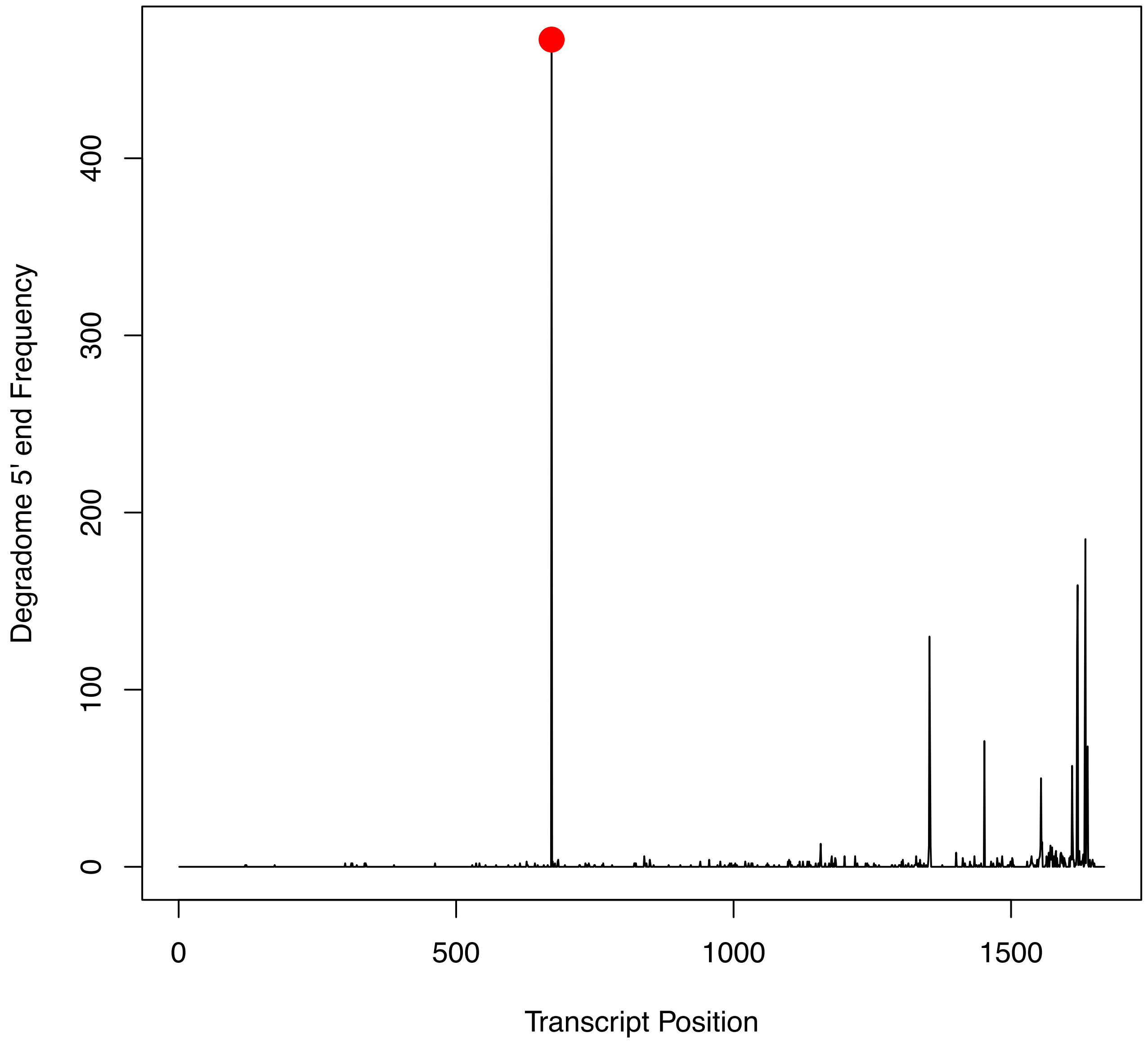

D=Day1
T=HORVU.MOREX.r3.2HG0193490.1
Q=miR396–5p.Cluster_1803
S=672
category=0
p=0.00325200384730273

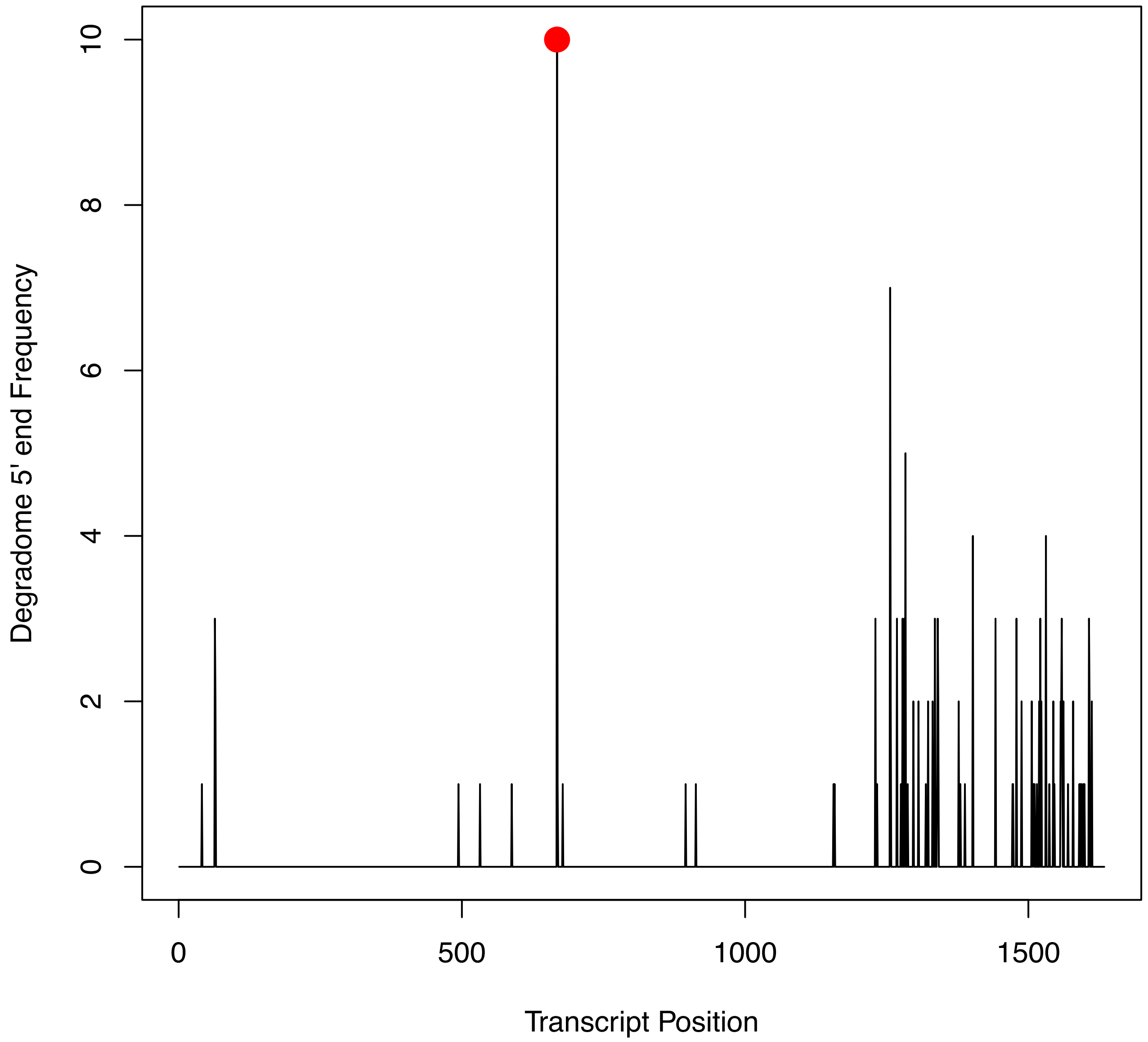

D=Day1
T=HORVU.MOREX.r3.4HG0334080.1
Q=miR396–5p.Cluster_1803
S=668
category=0
p=0.0012207429502803

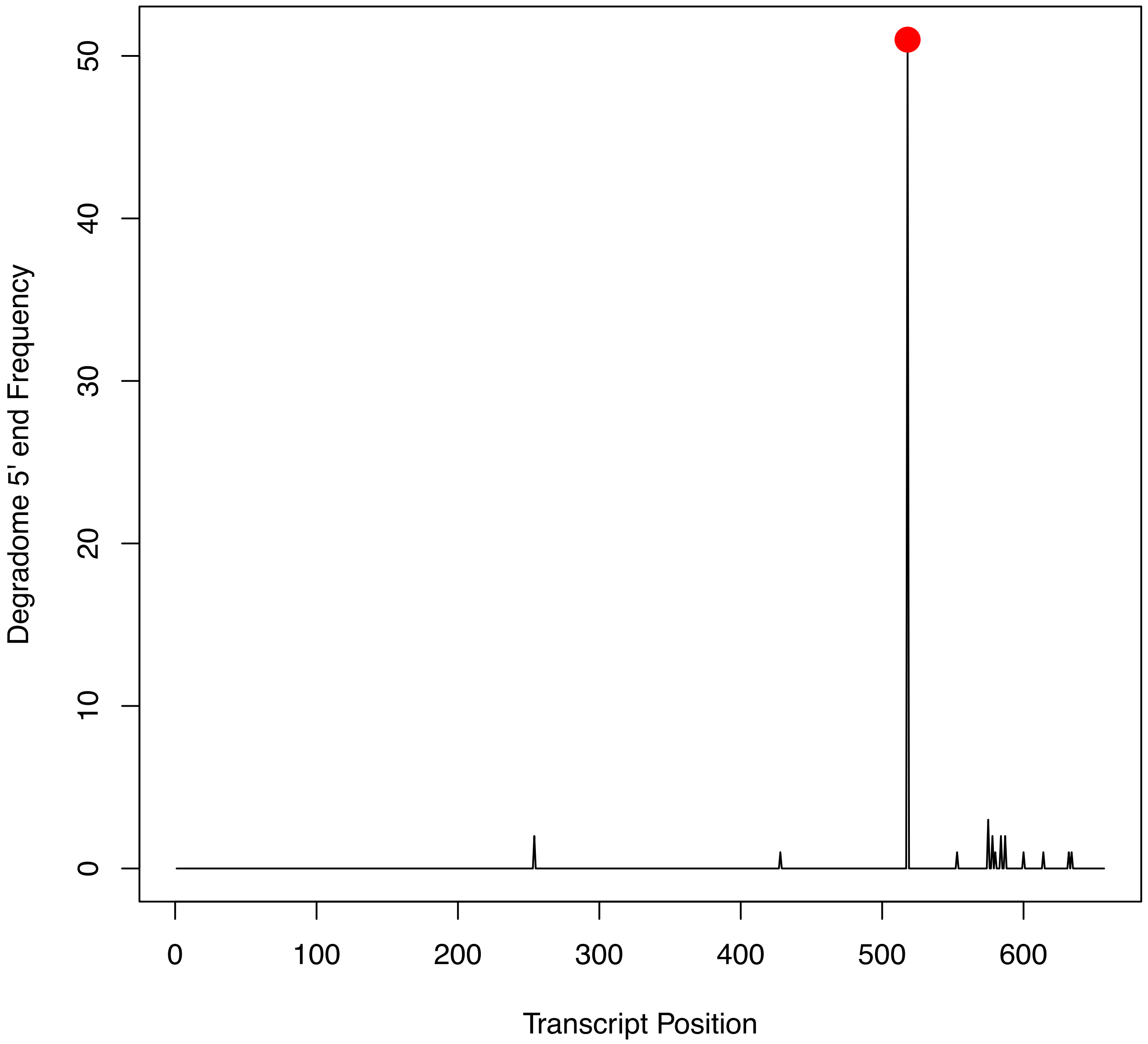

D=Day1
T=HORVU.MOREX.r3.6HG0603870.1
Q=miR396–5p.Cluster_1803
S=518
category=0
p=0.000813994302680809

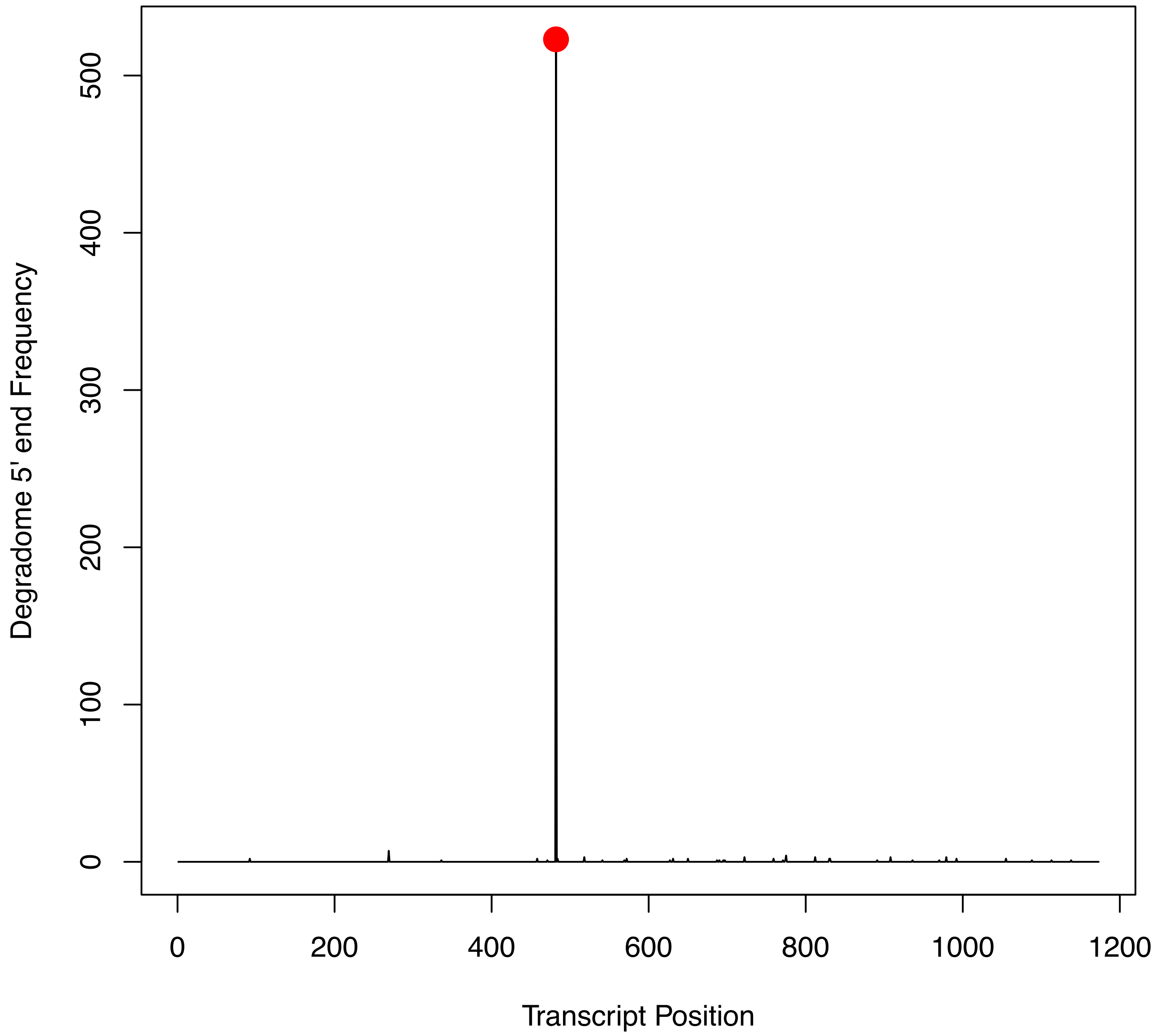

D=Day1
T=HORVU.MOREX.r3.6HG0606810.1
Q=miR396–5p.Cluster_1803
S=482
category=0
p=0.000407080008407057

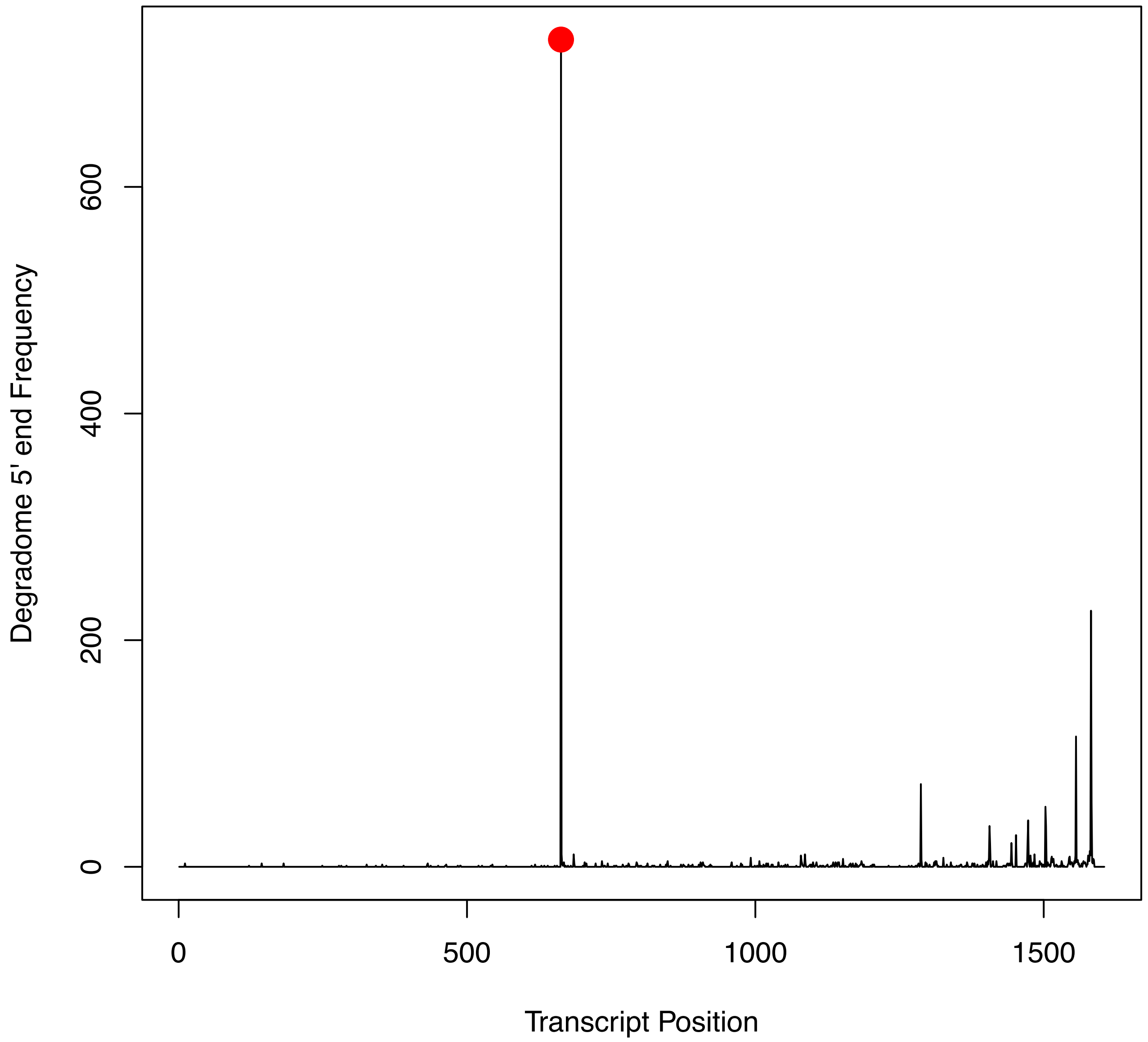

D=Day1
T=HORVU.MOREX.r3.7HG0641080.1
Q=miR396–5p.Cluster_1803
S=663
category=0
p=0.00203374357515451

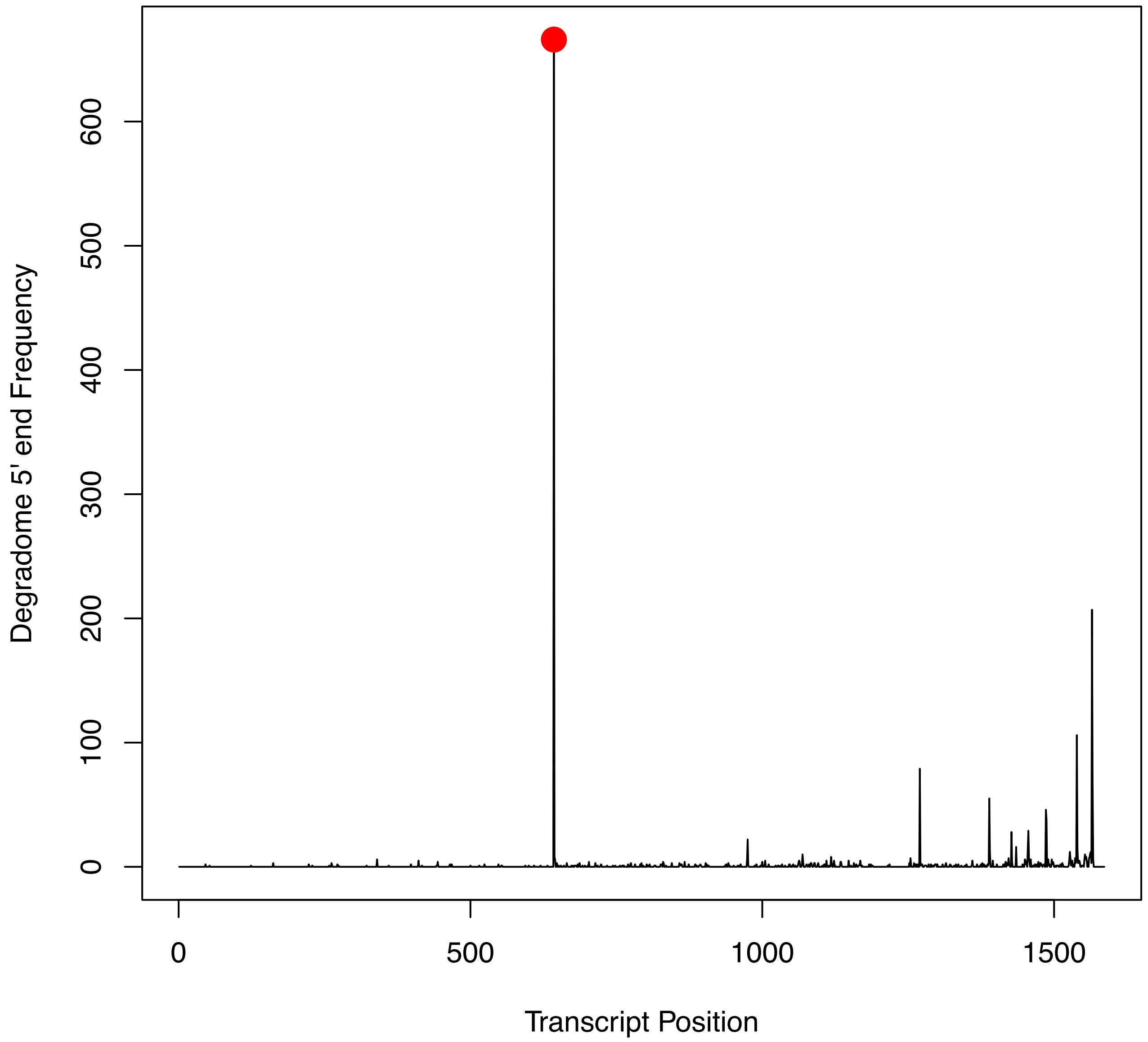

D=Day1
T=HORVU.MOREX.r3.7HG0641080.2
Q=miR396–5p.Cluster_1803
S=643
category=0
p=0.00284608242215212

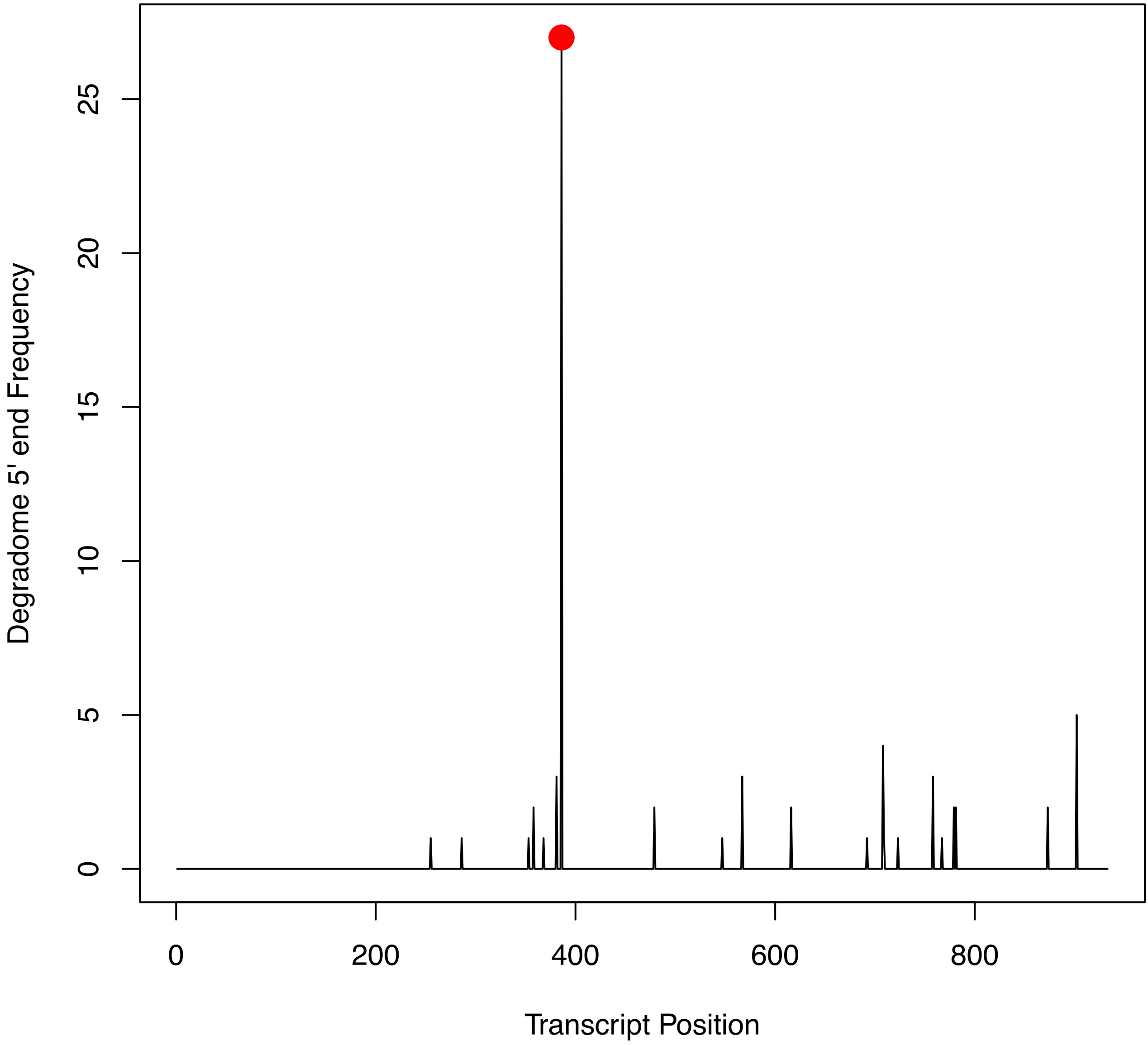

D=Day1
T=HORVU.MOREX.r3.7HG0662690.1
Q=miR396–5p.Cluster_1803
S=386
category=0
p=0.00365776002995633

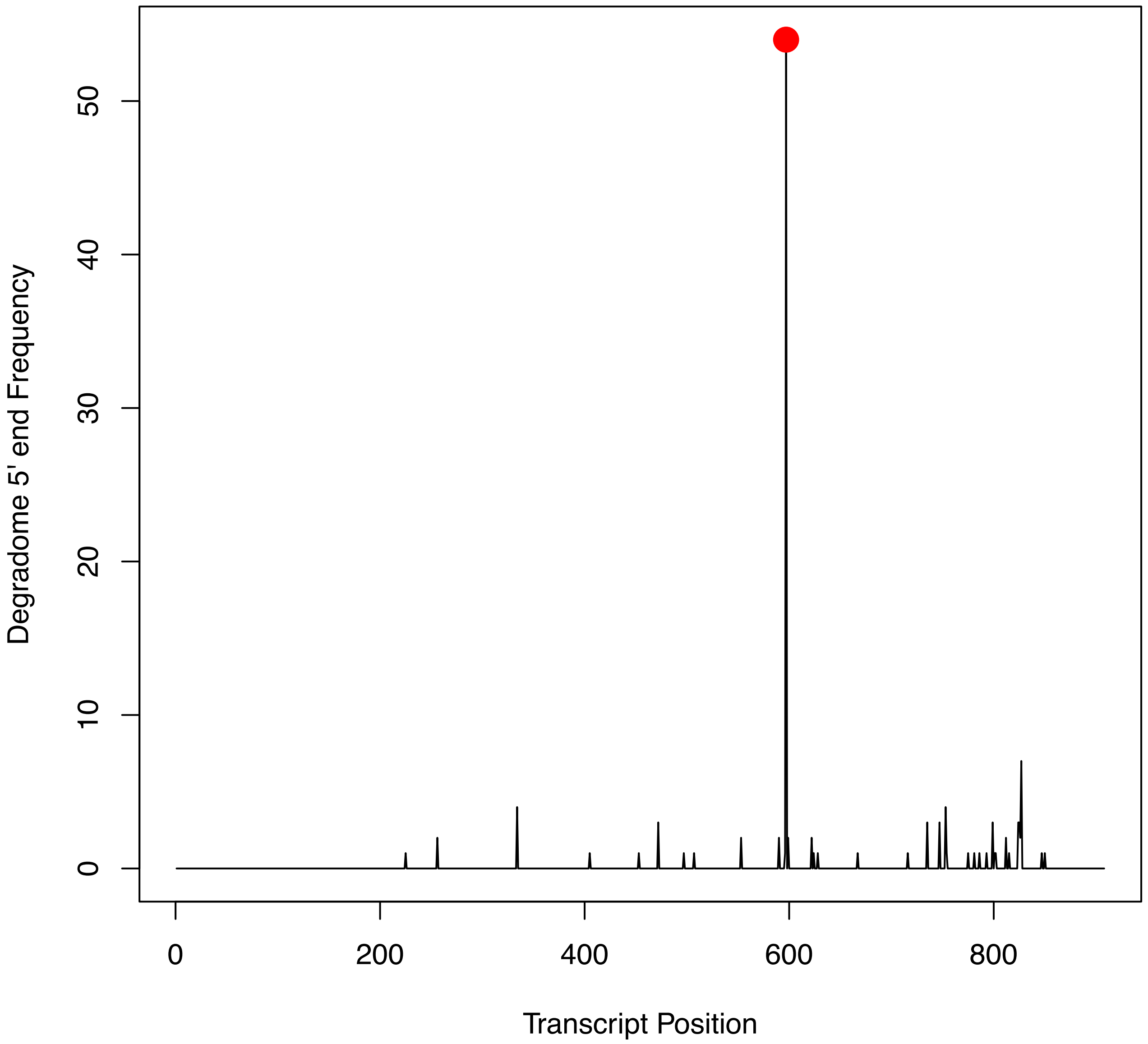

D=Day1
T=HORVU.MOREX.r3.2HG0186750.1
Q=miR396–5p.Cluster_5480
S=597
category=0
p=0.00203374357515451

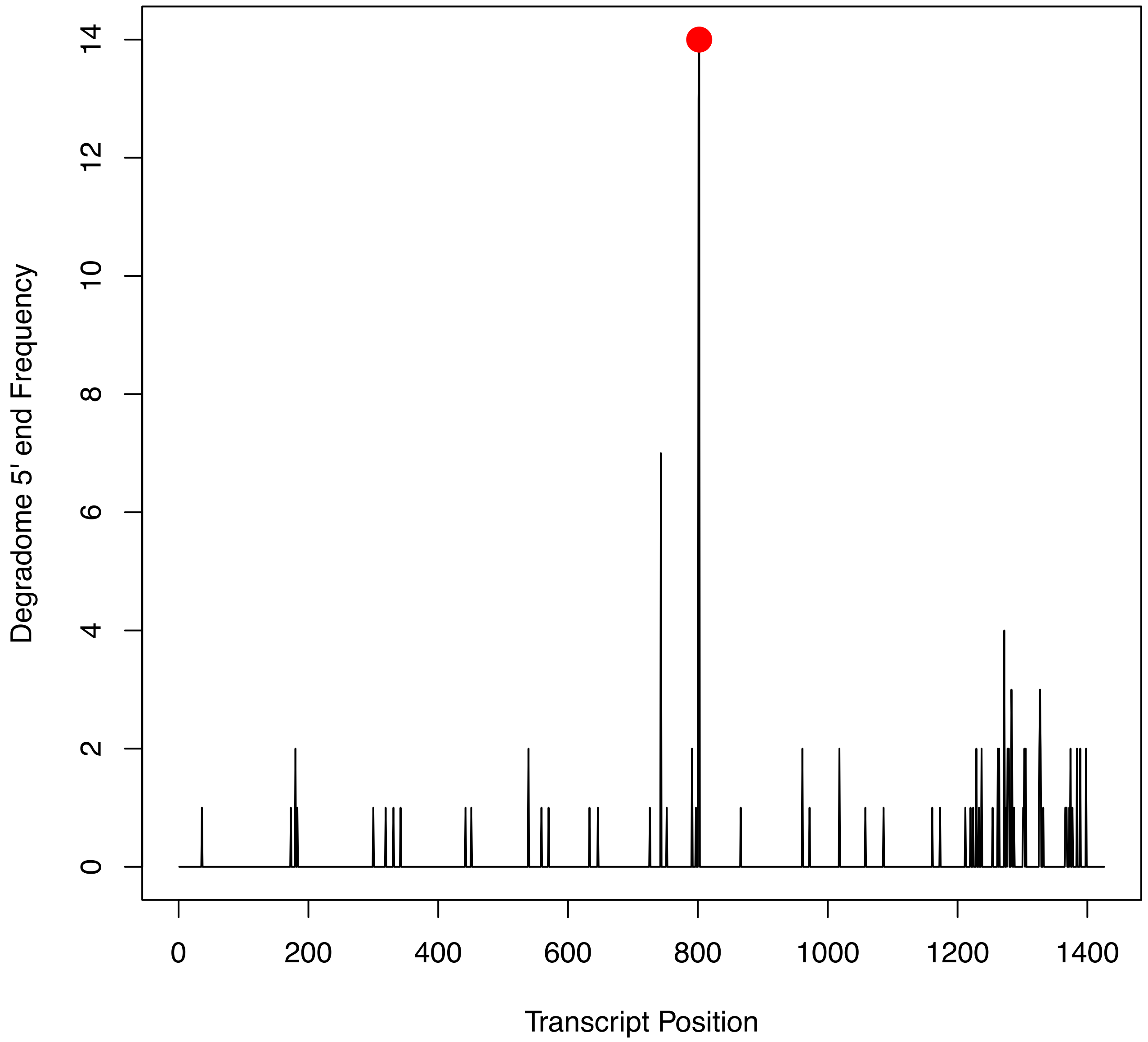

D=Day1
T=HORVU.MOREX.r3.6HG0541280.1
Q=miR9662–3p.Cluster_4660
S=802
category=0
p=0.0340168089372603

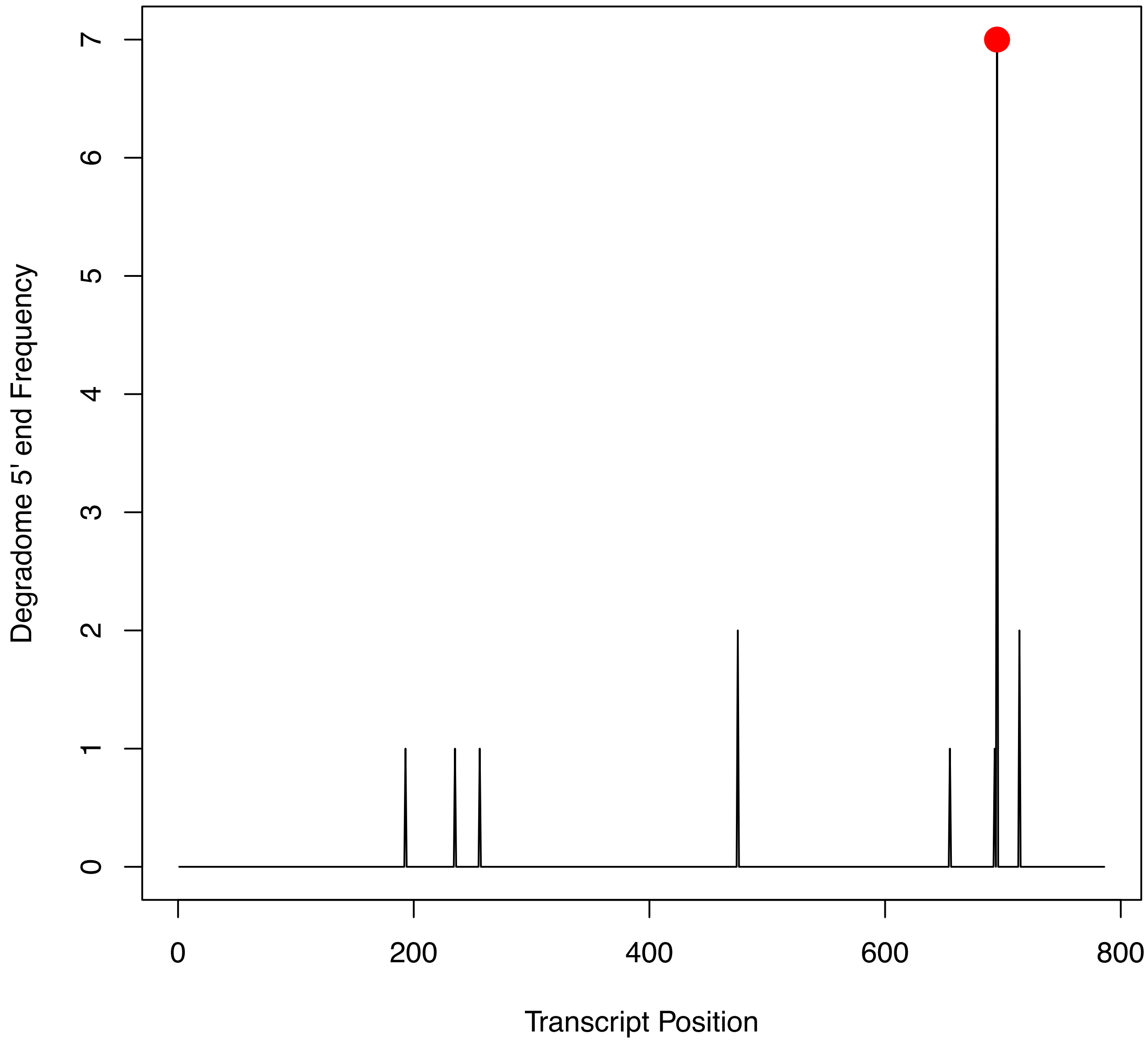

D=Day1
T=HORVU.MOREX.r3.6HG0543460.1
Q=miR9662–3p.Cluster_4660
S=695
category=0
p=0.000813994302680809

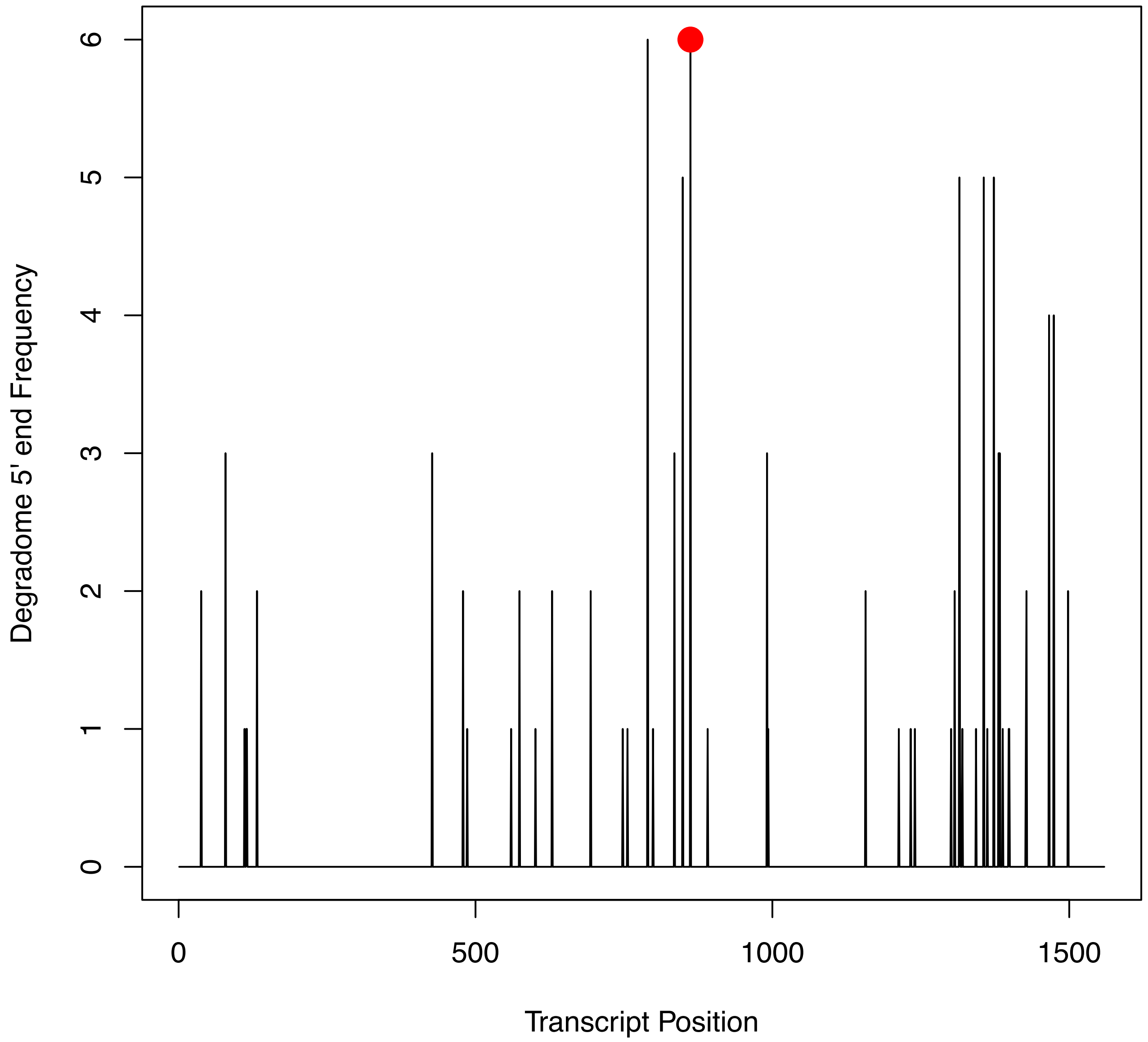

D=Day1
T=HORVU.MOREX.r3.7HG0665830.1
Q=miR9662–3p.Cluster_4660
S=862
category=1
p=0.00485412302595578

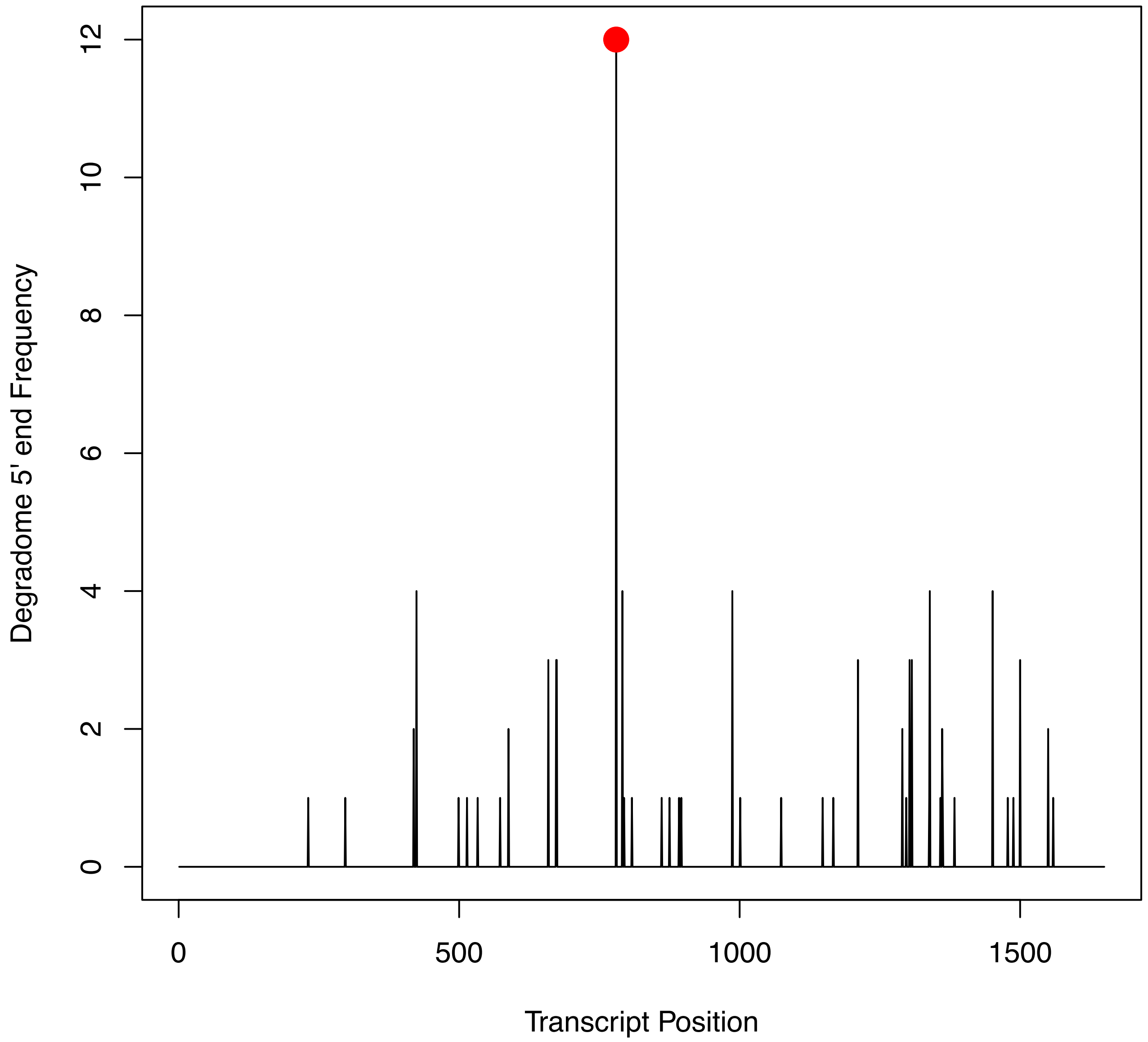

D=Day1
T=HORVU.MOREX.r3.7HG0710980.1
Q=miR9662–3p.Cluster_4660
S=780
category=0
p=0.0161546073040506

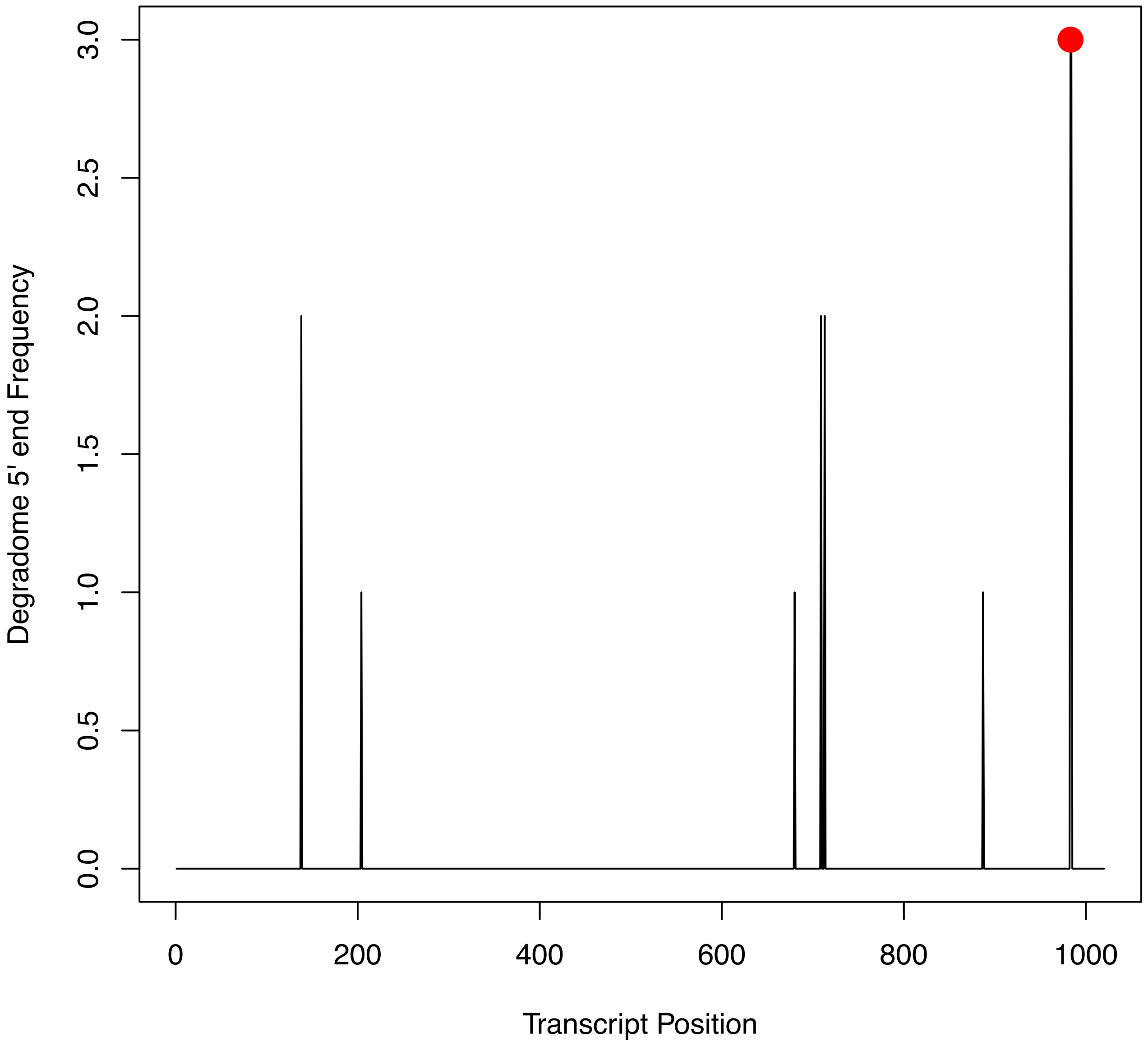

D=Day1
T=HORVU.MOREX.r3.2HG0184480.1
Q=novel–3p.Cluster_3664
S=983
category=1
p=0.0430424352977843

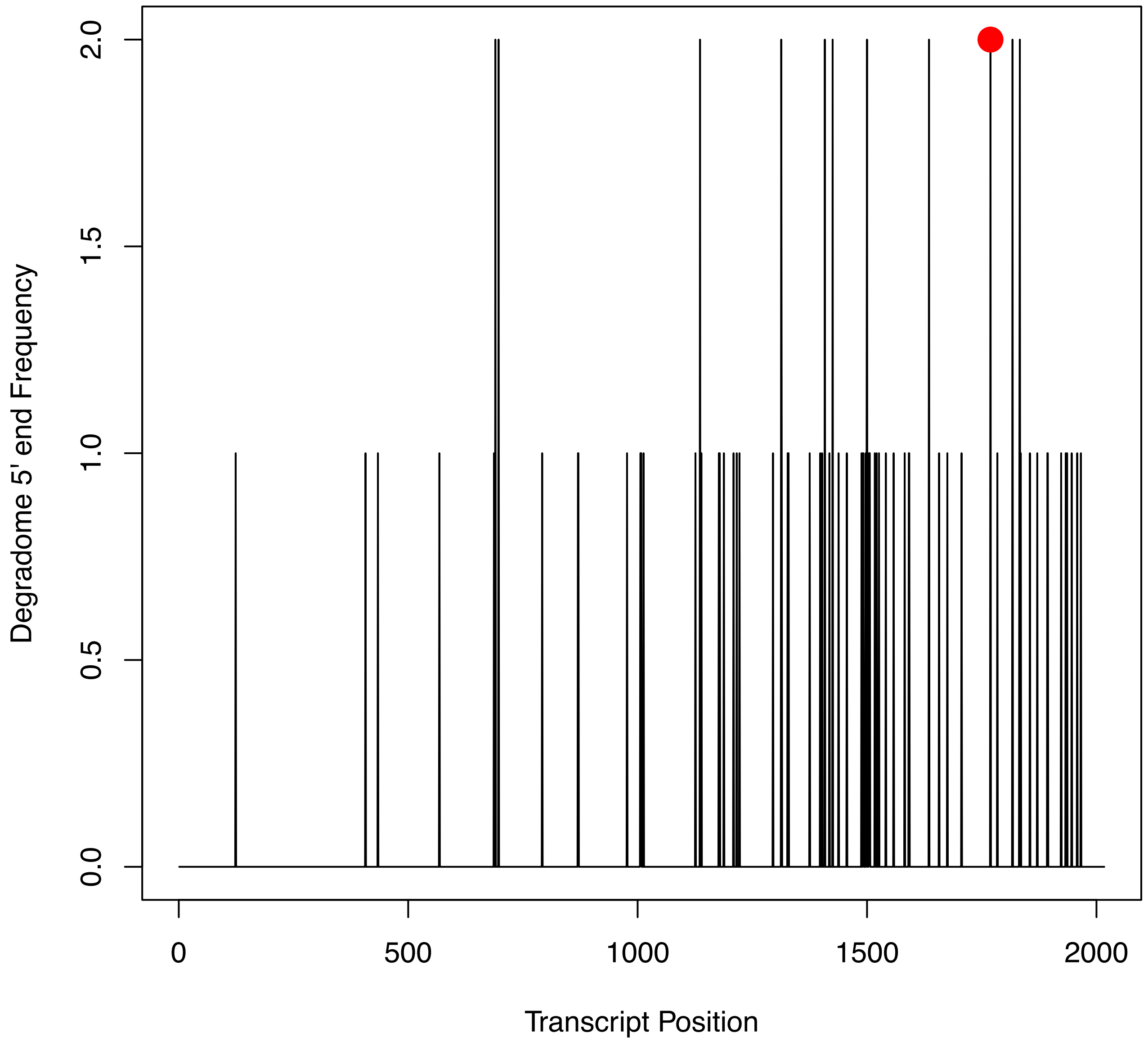

D=Day3
T=HORVU.MOREX.r3.5HG0440170.1
Q=miR156–3p.Cluster_1973
S=1769
category=1
p=0.046095685553886

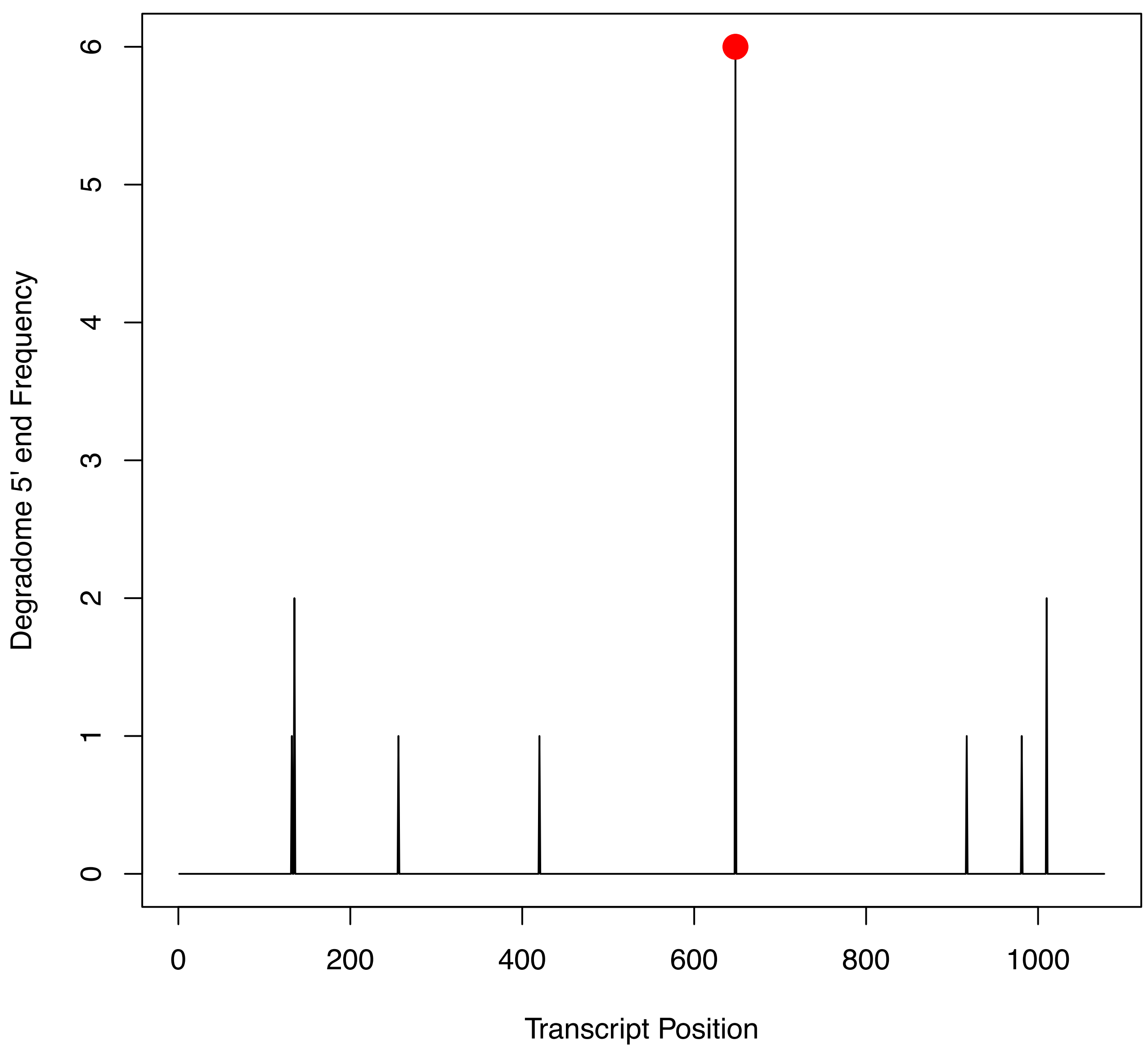

D=Day3
T=HORVU.MOREX.r3.2HG0189580.1
Q=miR156–5p.Cluster_1557.Cluster_1973.Cluster_4963
S=648
category=0
p=0.00160391122025372

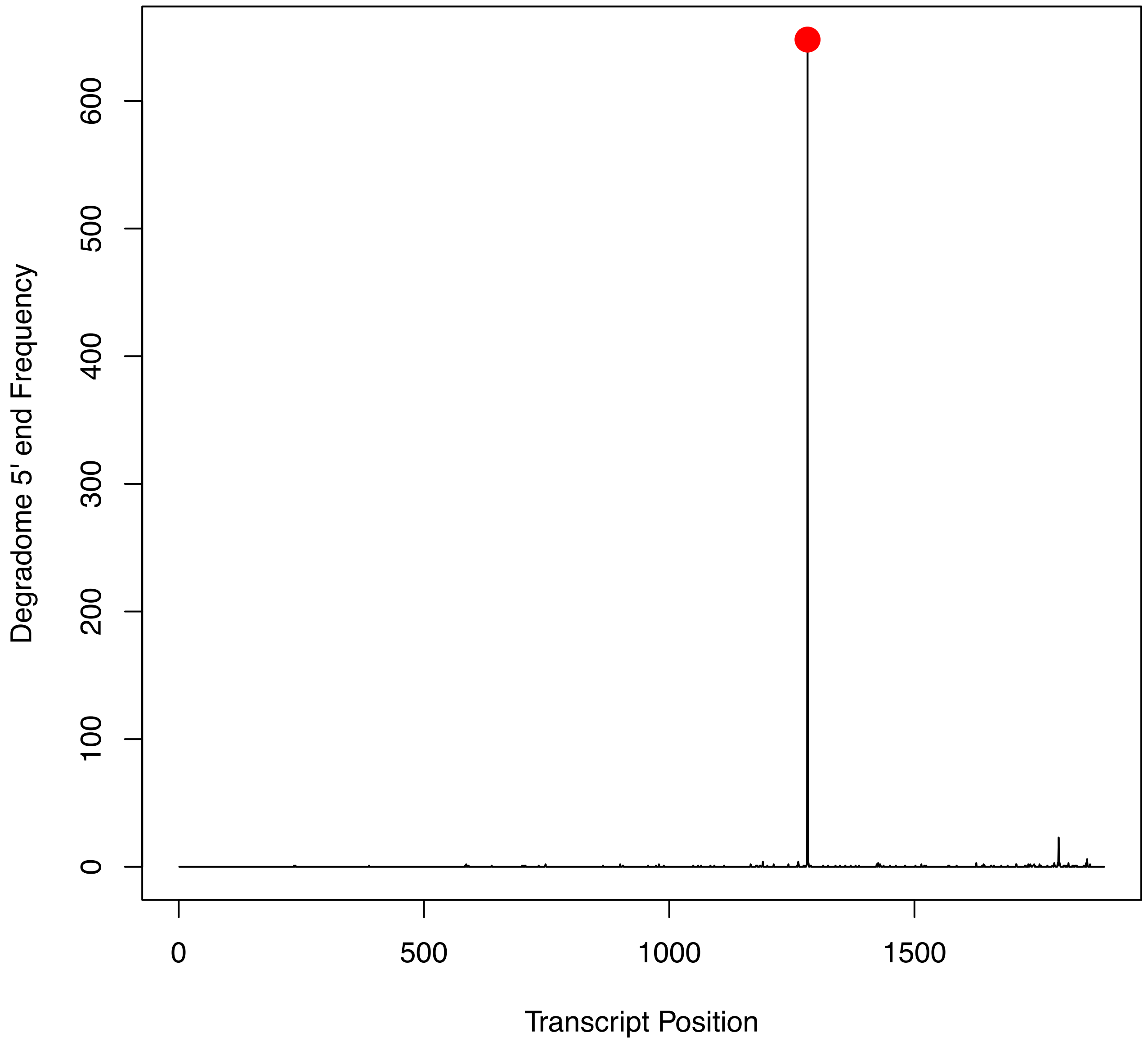

D=Day3
T=HORVU.MOREX.r3.3HG0310780.1
Q=miR156–5p.Cluster_1557.Cluster_1973.Cluster_4963
S=1282
category=0
p=0.00240490187314257

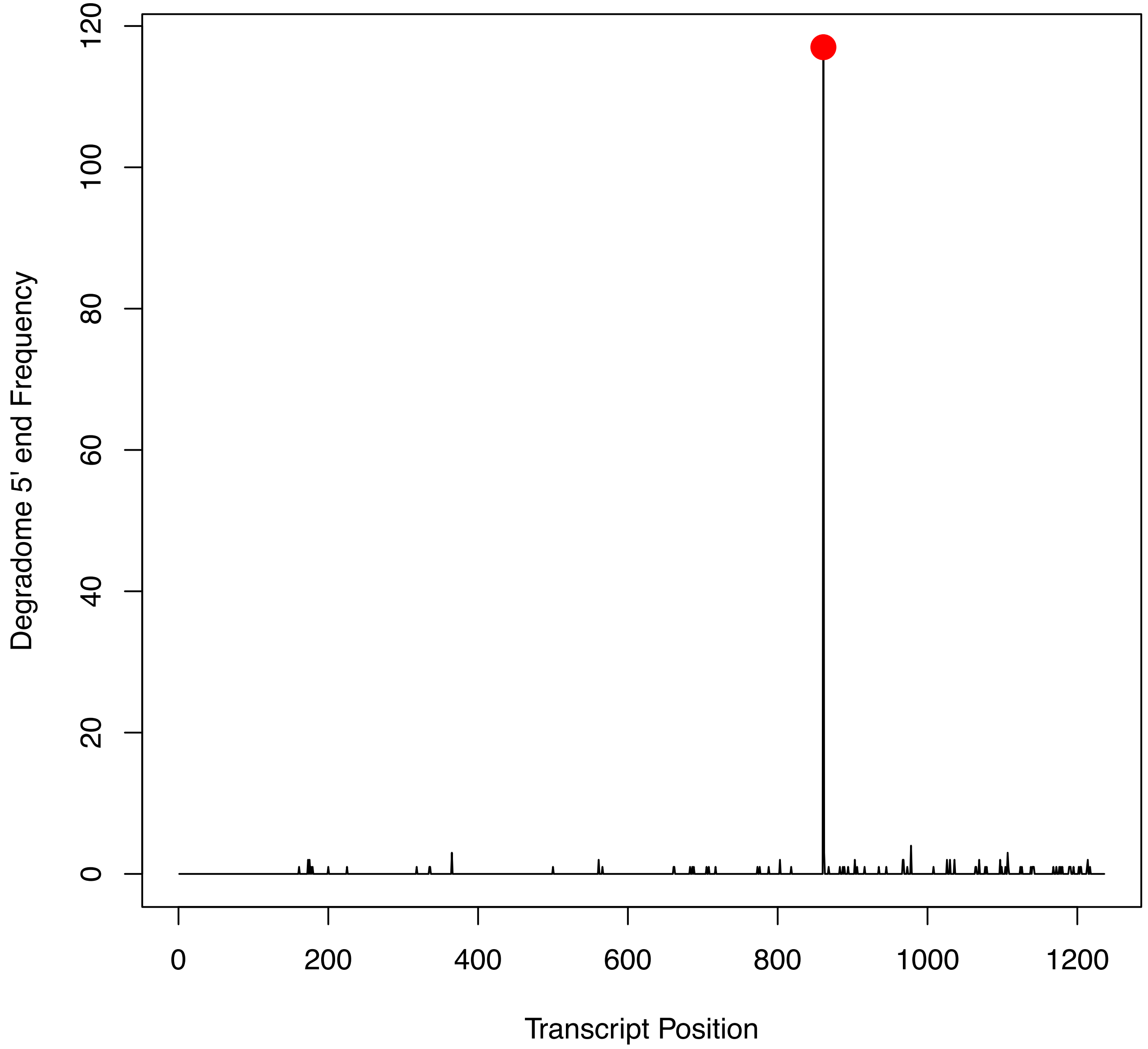

D=Day3
T=HORVU.MOREX.r3.5HG0490900.1
Q=miR156–5p.Cluster_1557.Cluster_1973.Cluster_4963
S=861
category=0
p=0.00200448690602739

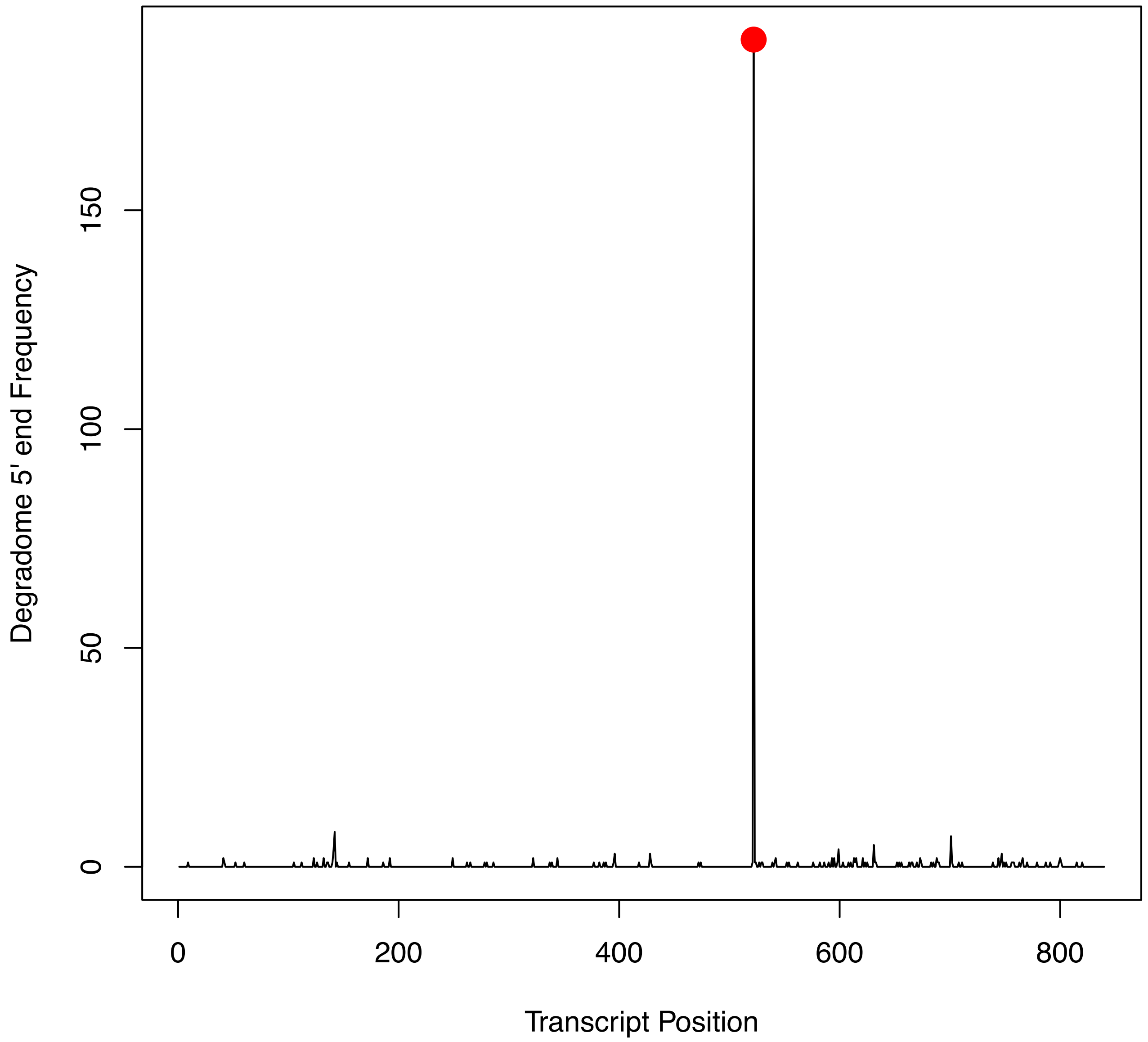

D=Day3
T=HORVU.MOREX.r3.5HG0494180.1
Q=miR156–5p.Cluster_1557.Cluster_1973.Cluster_4963
S=522
category=0
p=0.00280515618608268

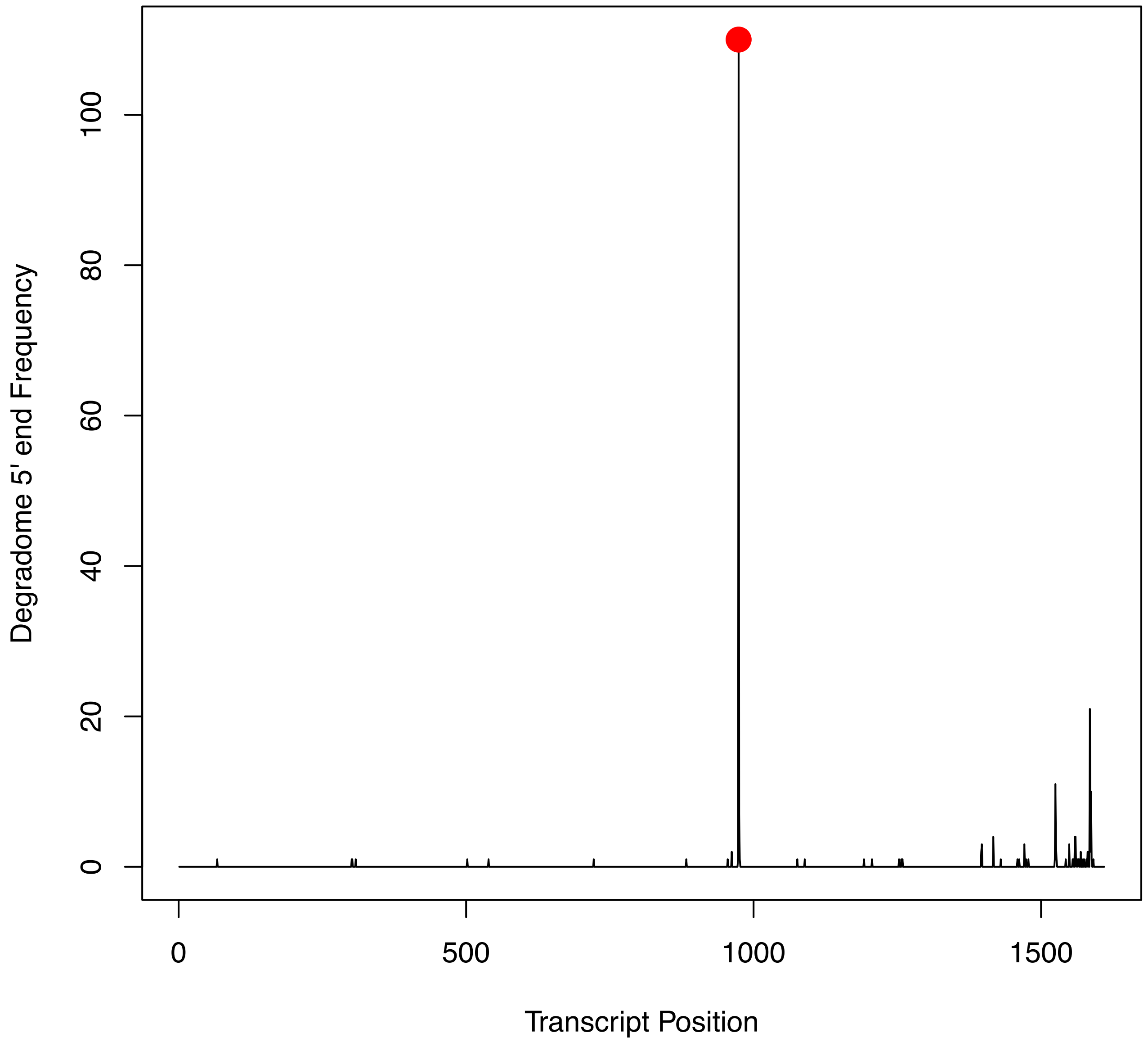

D=Day3
T=HORVU.MOREX.r3.7HG0679980.1
Q=miR156–5p.Cluster_1557.Cluster_1973.Cluster_4963
S=974
category=0
p=0.00120317475131237

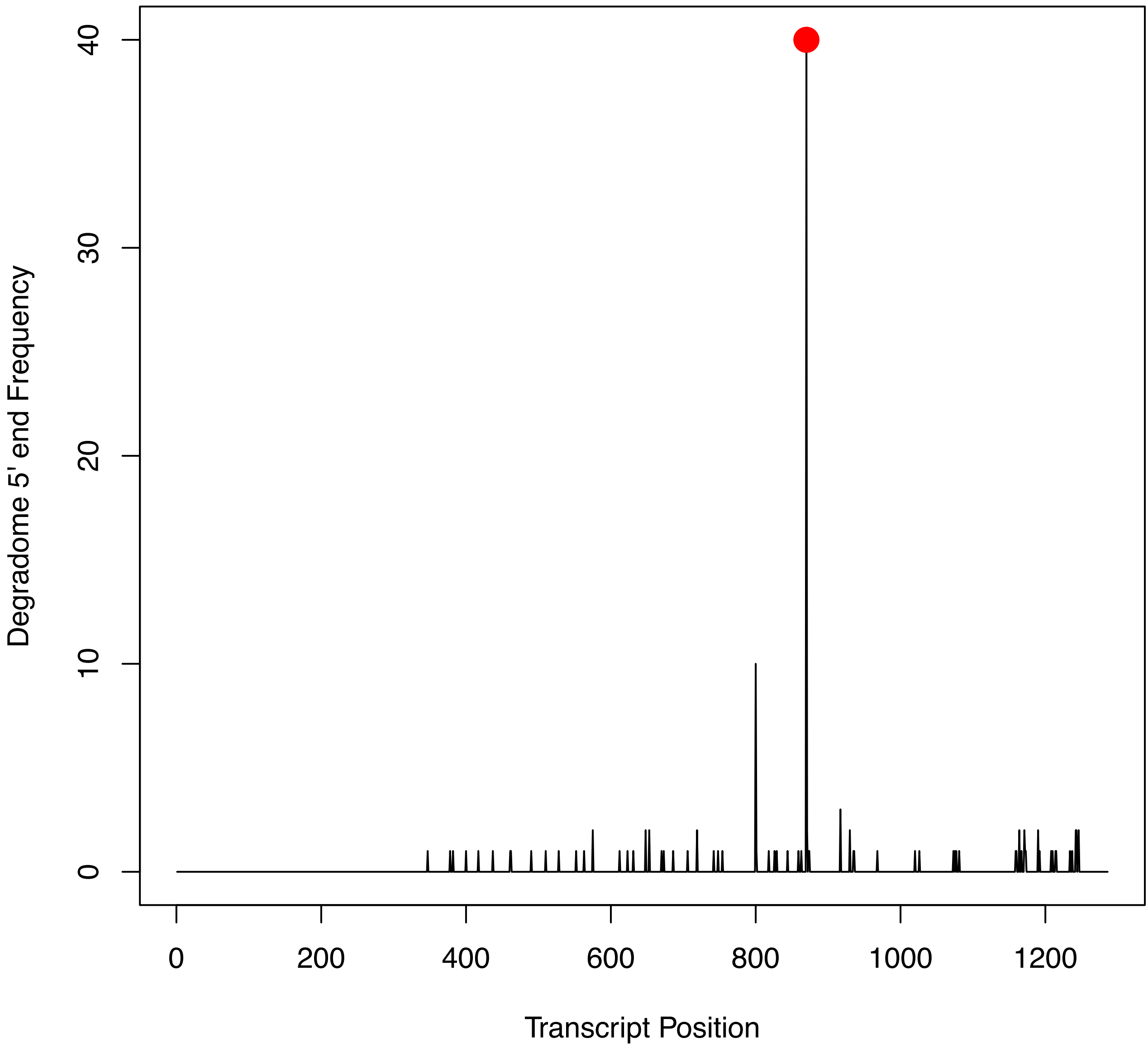

D=Day3
T=HORVU.MOREX.r3.7HG0684000.1
Q=miR156–5p.Cluster_1557.Cluster_1973.Cluster_4963
S=870
category=0
p=0.00802277434667986

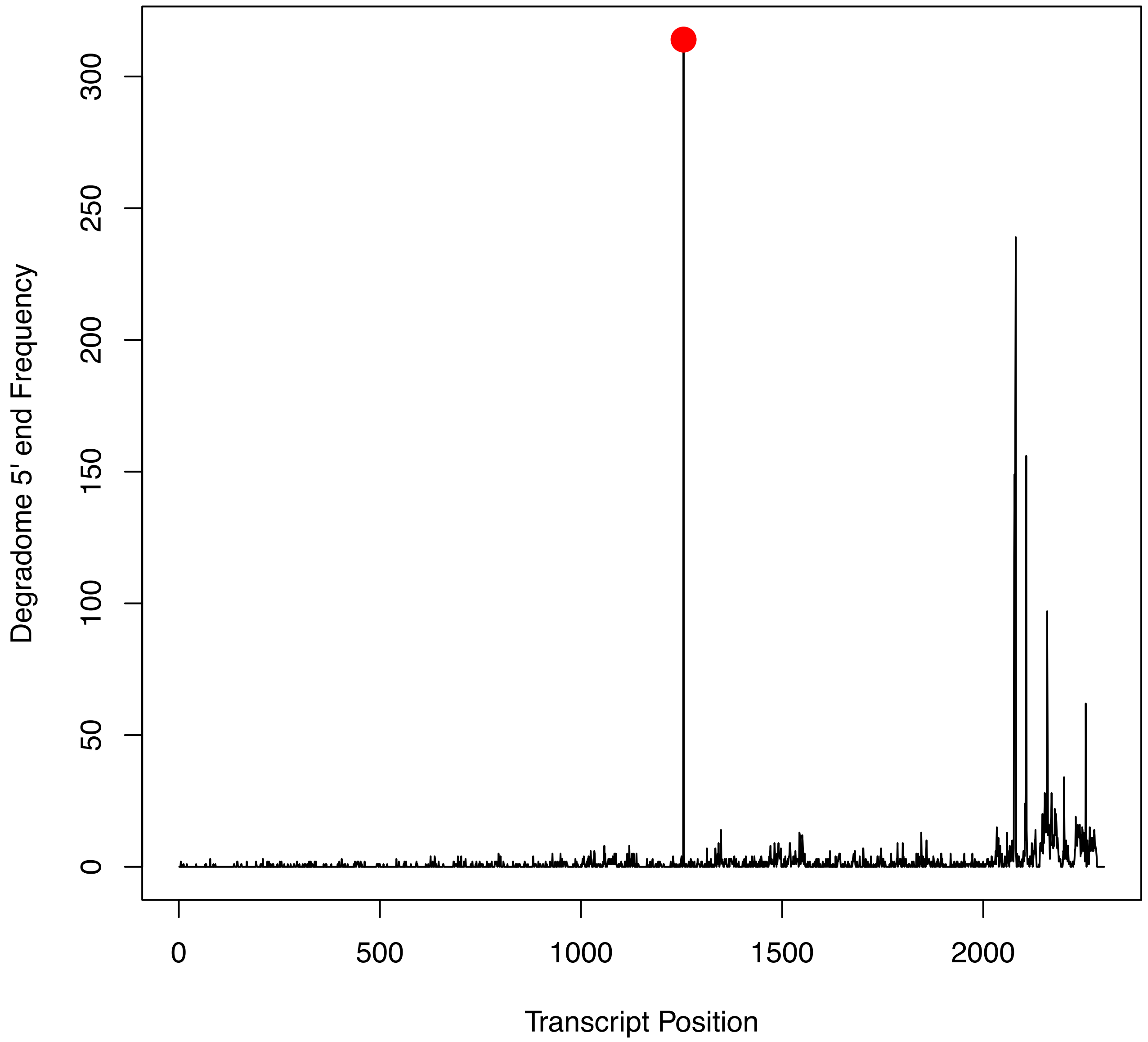

D=Day3
T=HORVU.MOREX.r3.3HG0296070.1
Q=miR159–3p.Cluster_1875
S=1255
category=0
p=0.00120317475131237

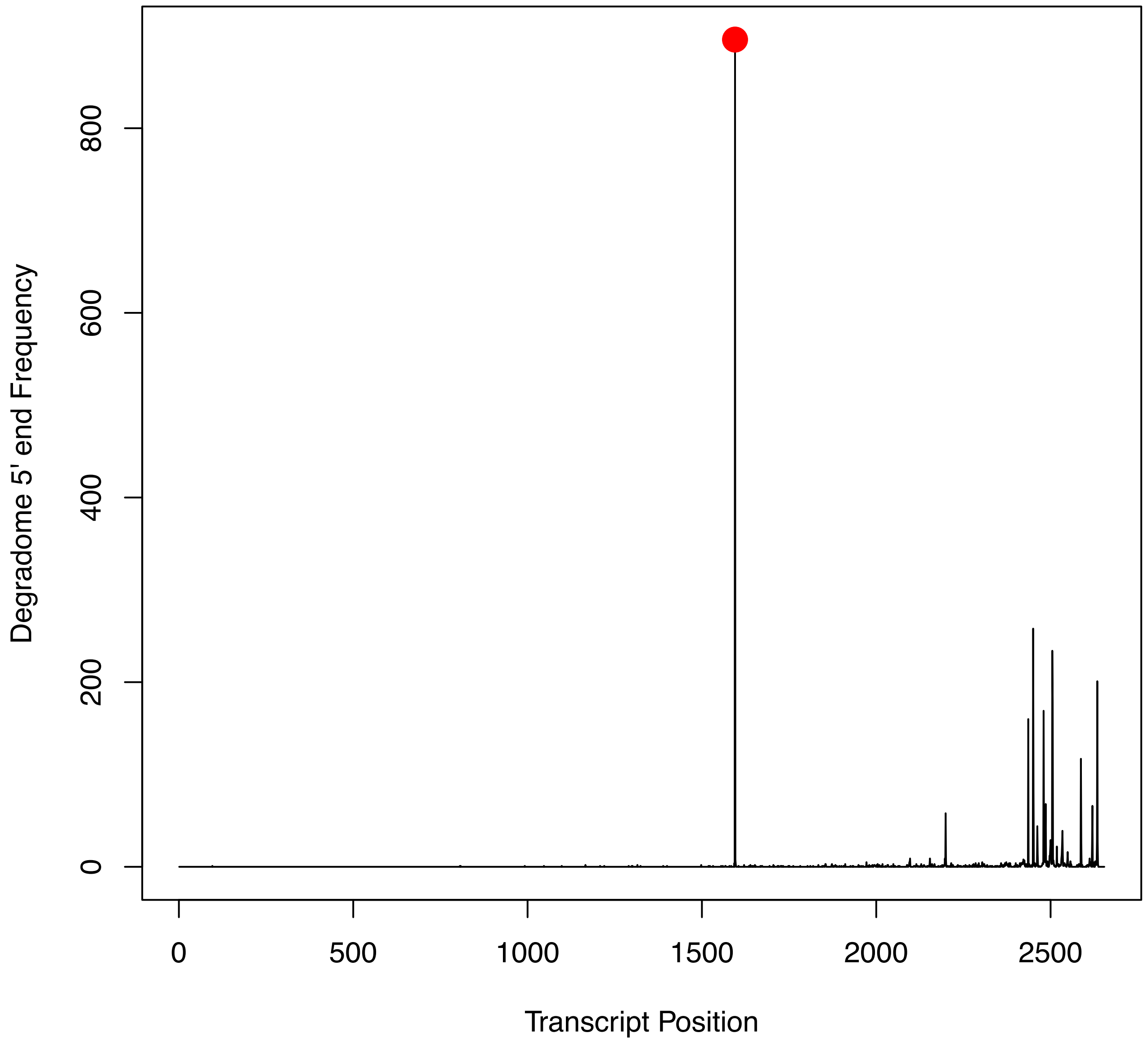

D=Day3
T=HORVU.MOREX.r3.1HG0043670.1
Q=miR160–5p.Cluster_6224
S=1595
category=0
p=0.00160391122025372

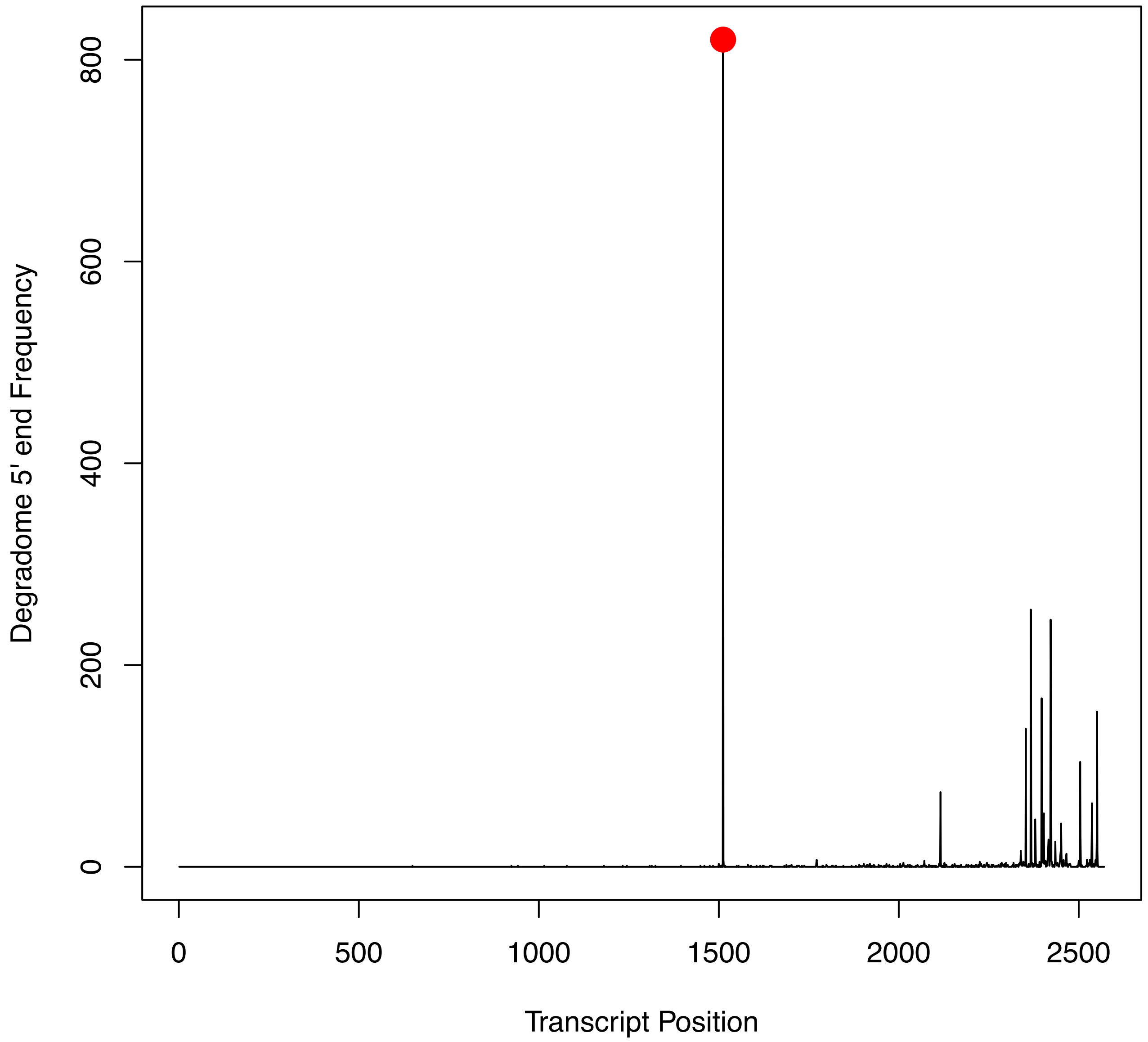

D=Day3
T=HORVU.MOREX.r3.1HG0043670.2
Q=miR160–5p.Cluster_6224
S=1512
category=0
p=0.00200448690602739

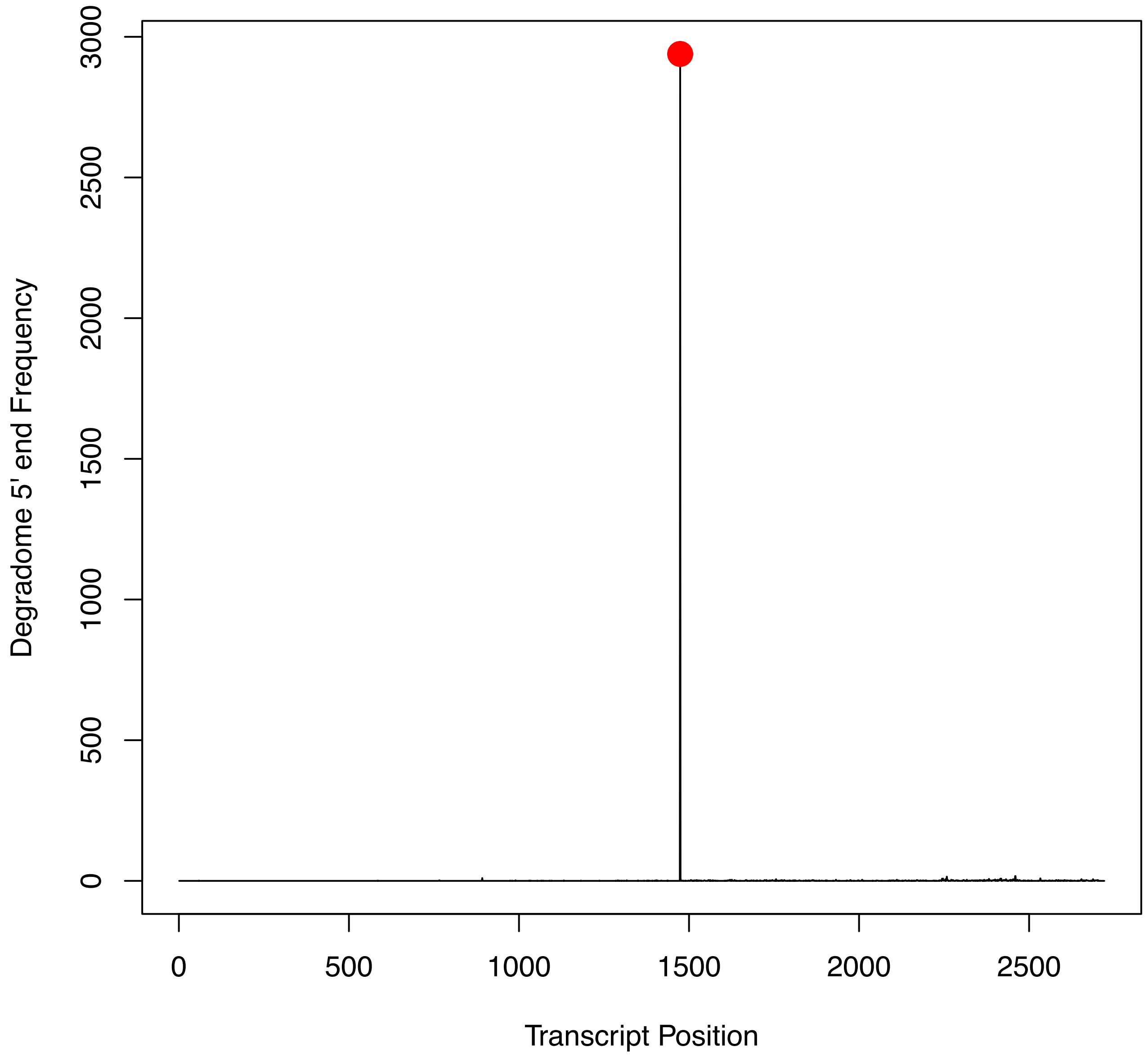

D=Day3
T=HORVU.MOREX.r3.2HG0182280.1
Q=miR160–5p.Cluster_6224
S=1474
category=0
p=0.00240490187314257

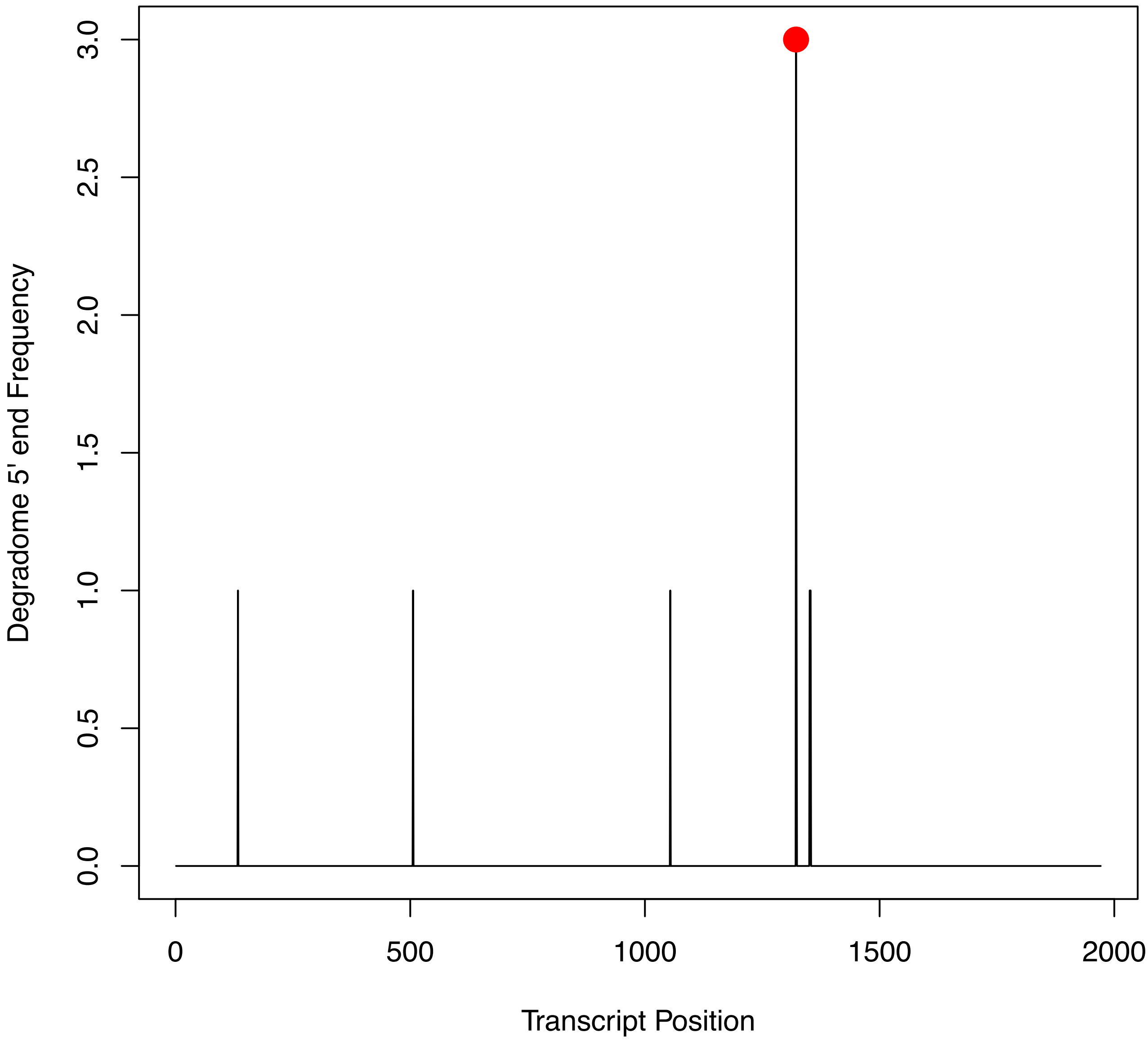

D=Day3
T=HORVU.MOREX.r3.2HG0216230.1
Q=miR160–5p.Cluster_6224
S=1322
category=0
p=0.000802277434667986

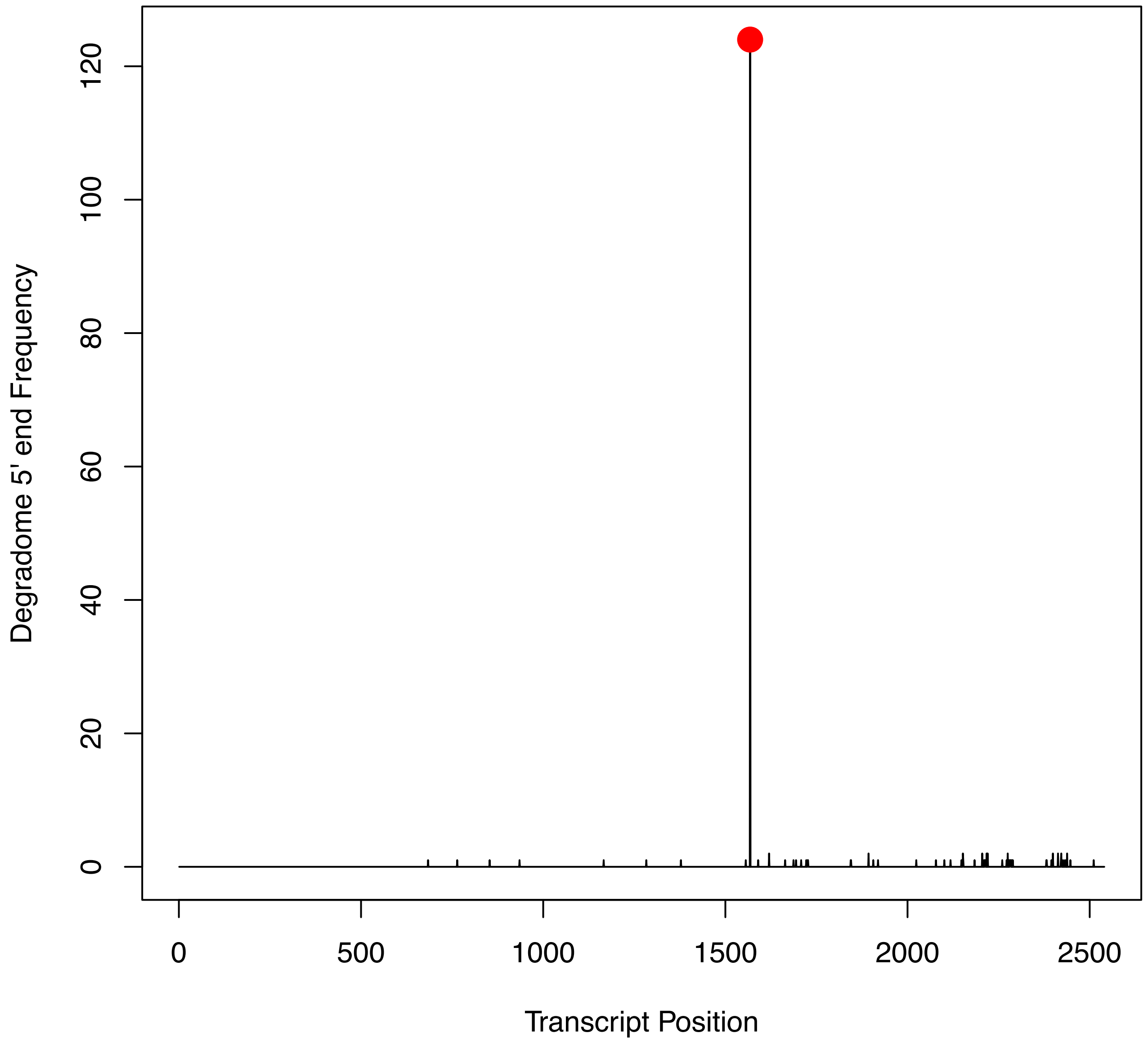

D=Day3
T=HORVU.MOREX.r3.6HG0596670.1
Q=miR160–5p.Cluster_6224
S=1568
category=0
p=0.000401219205759529

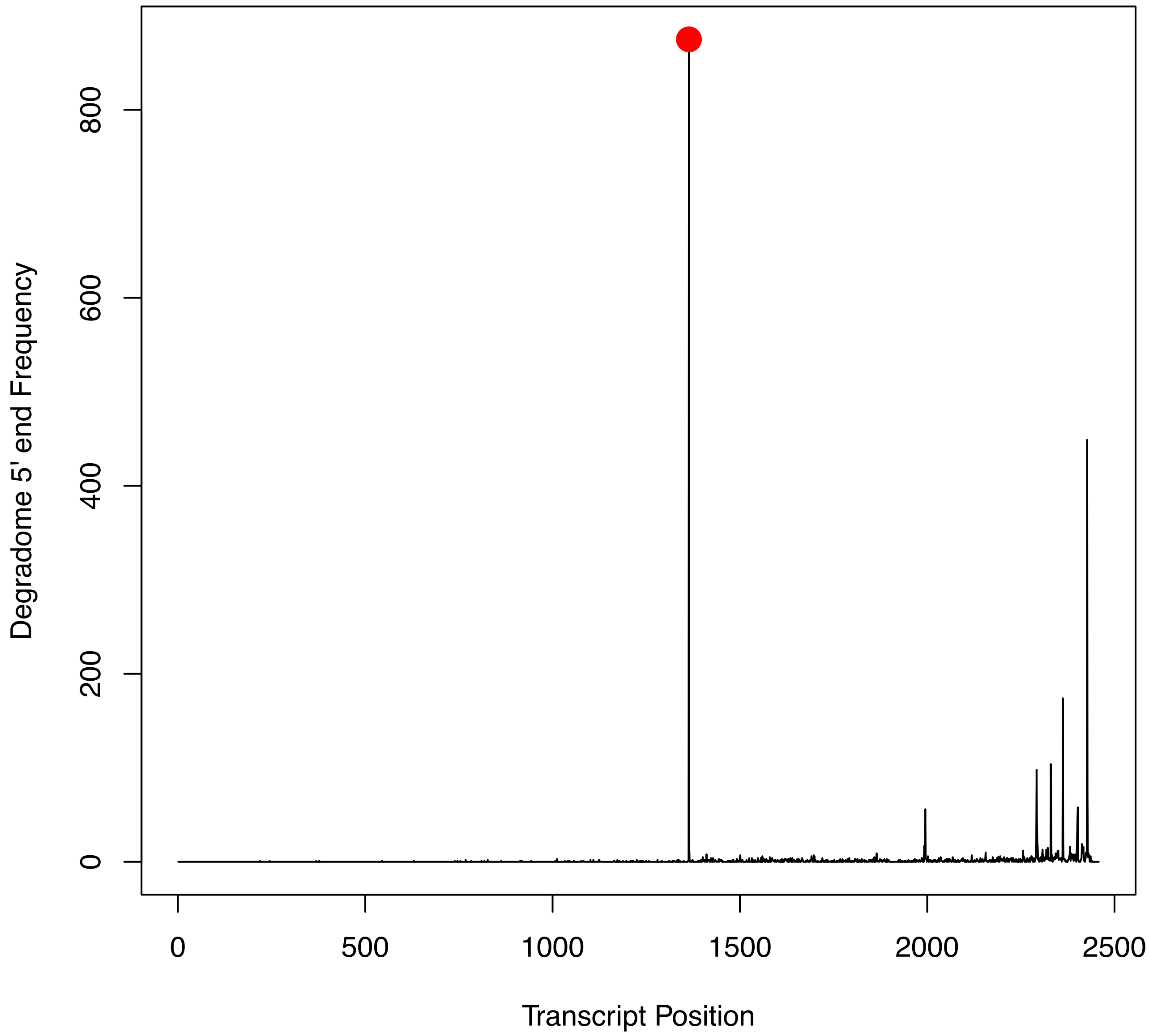

D=Day3
T=HORVU.MOREX.r3.7HG0731750.1
Q=miR160–5p.Cluster_6224
S=1364
category=0
p=0.00120317475131237

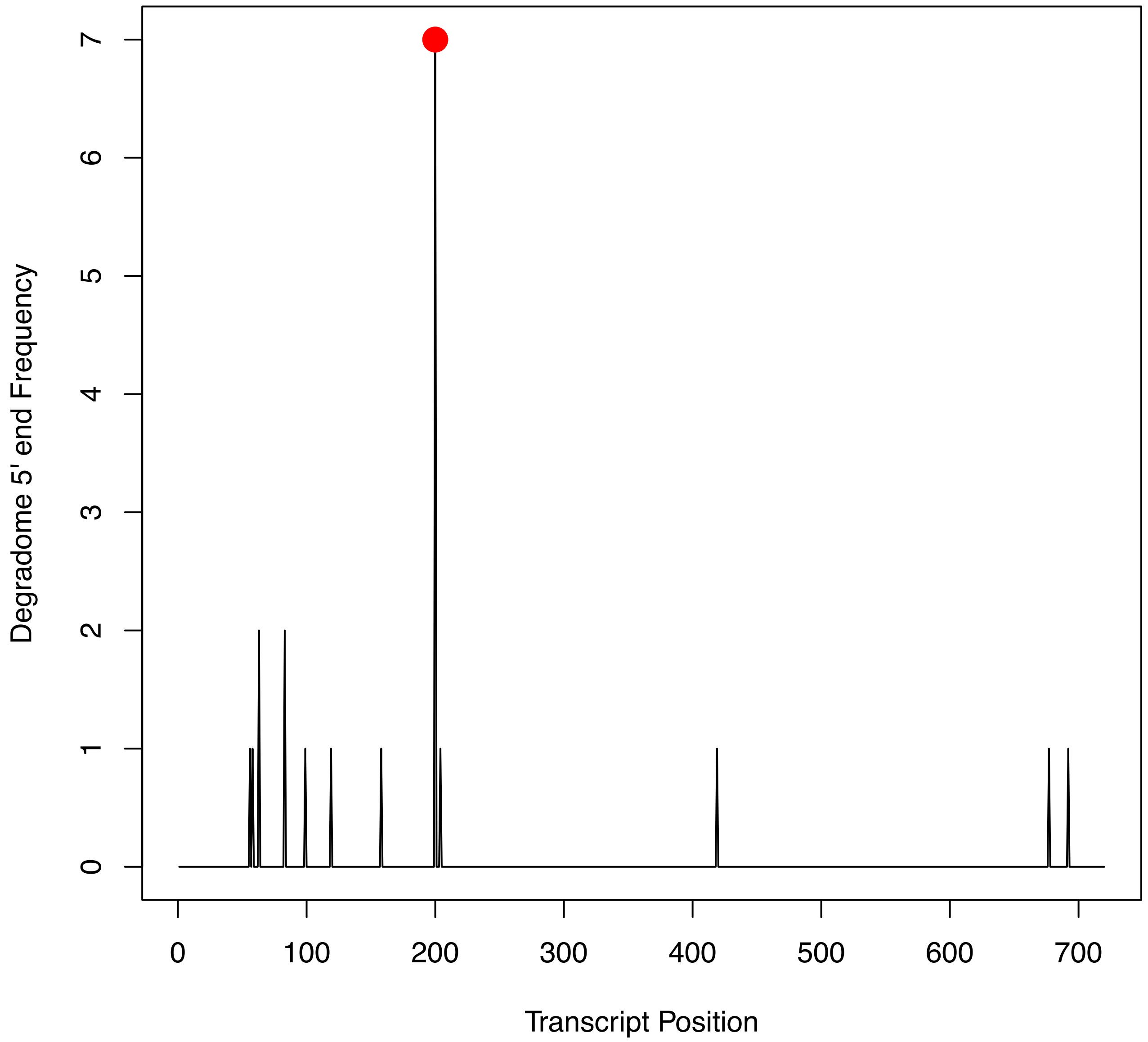

D=Day3
T=HORVU.MOREX.r3.2HG0139920.1
Q=miR166–3p.Cluster_426.Cluster_3396
S=200
category=0
p=0.00240490187314257

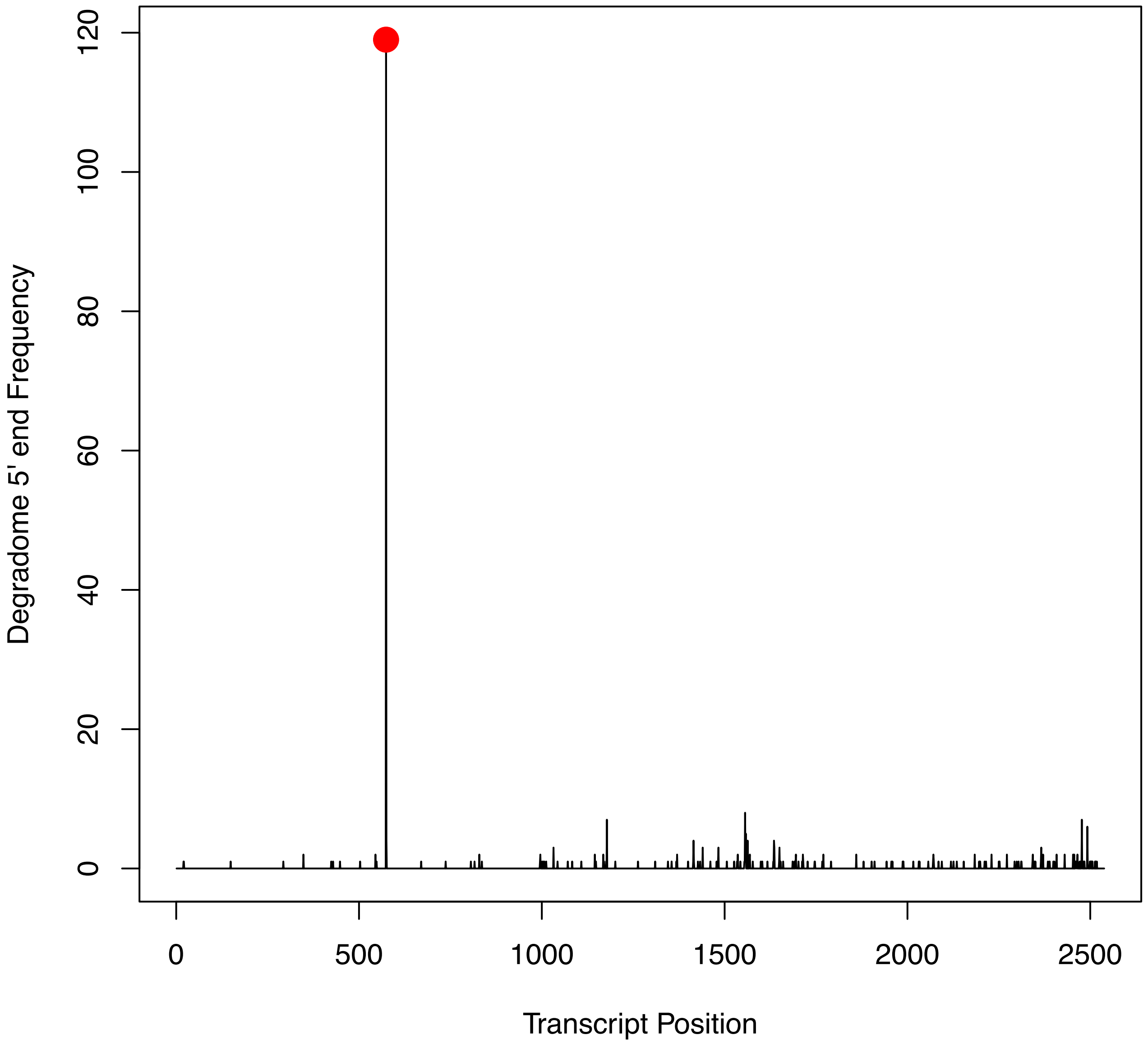

D=Day3
T=HORVU.MOREX.r3.3HG0244080.1
Q=miR166–3p.Cluster_426.Cluster_3396
S=574
category=0
p=0.00120317475131237

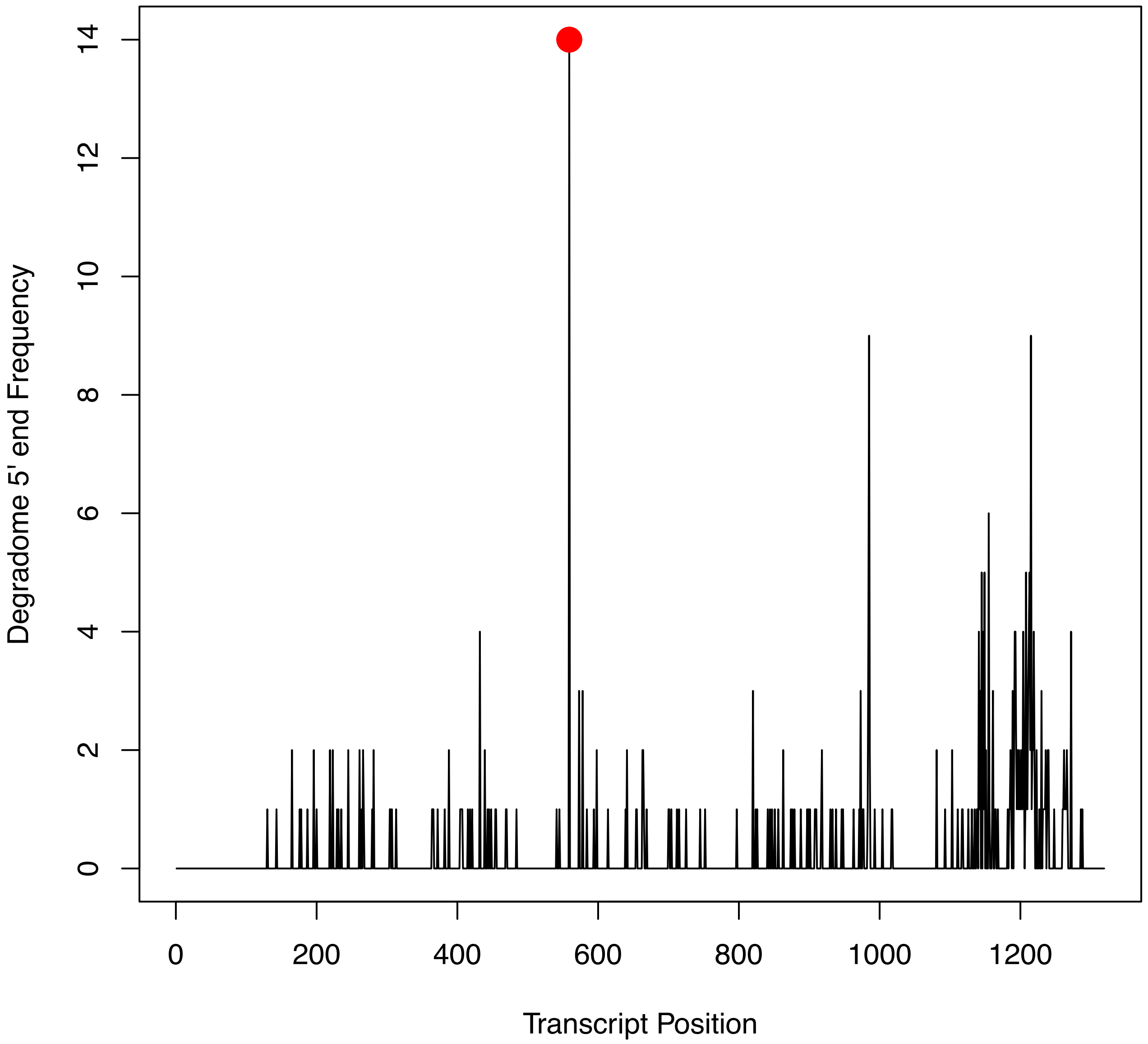

D=Day3
T=HORVU.MOREX.r3.1HG0028230.1
Q=miR166–3p.Cluster_4051
S=559
category=0
p=0.00240490187314257

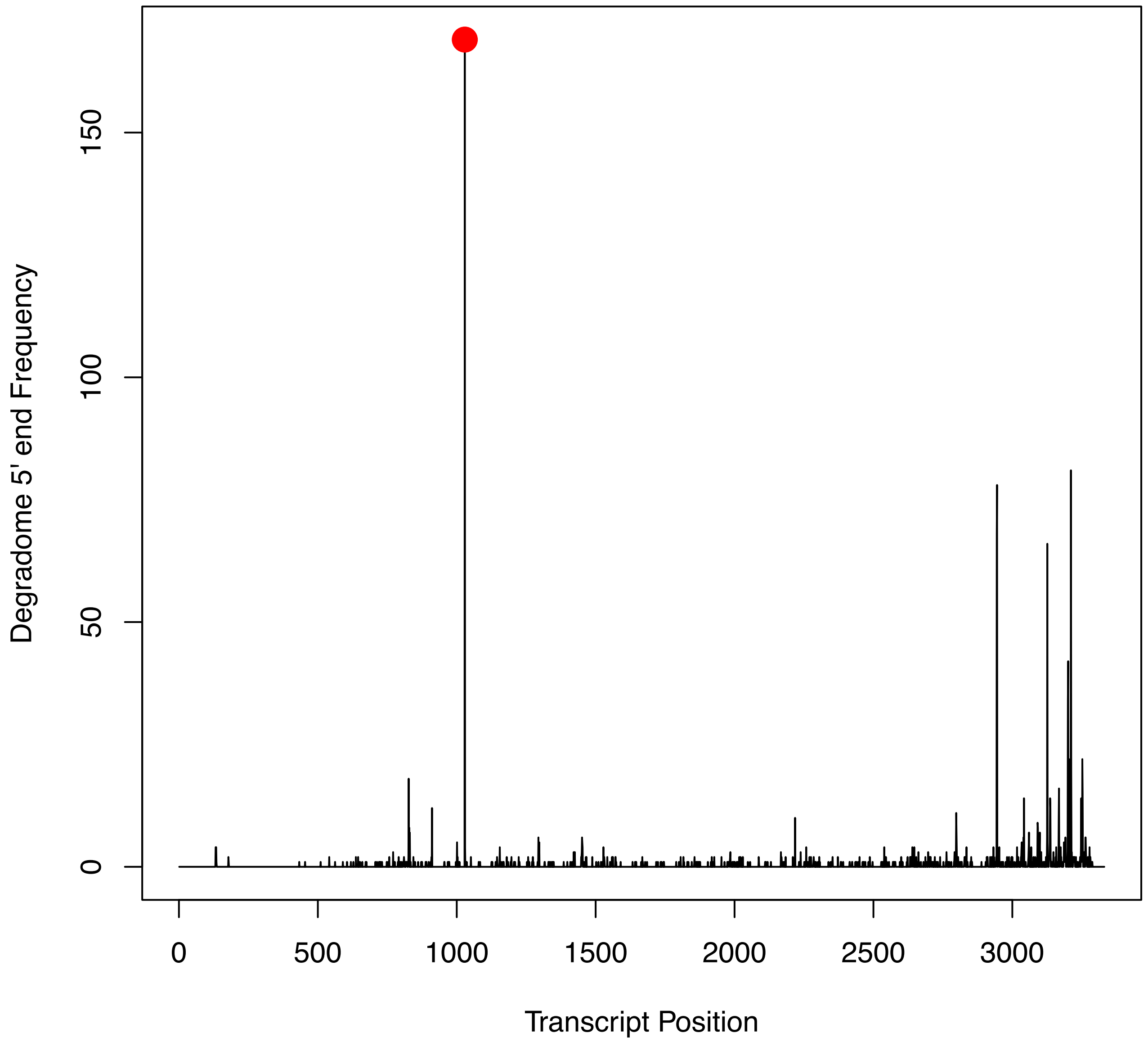

D=Day3
T=HORVU.MOREX.r3.4HG0417970.1
Q=miR166–3p.Cluster_4051
S=1029
category=0
p=0.00802277434667986

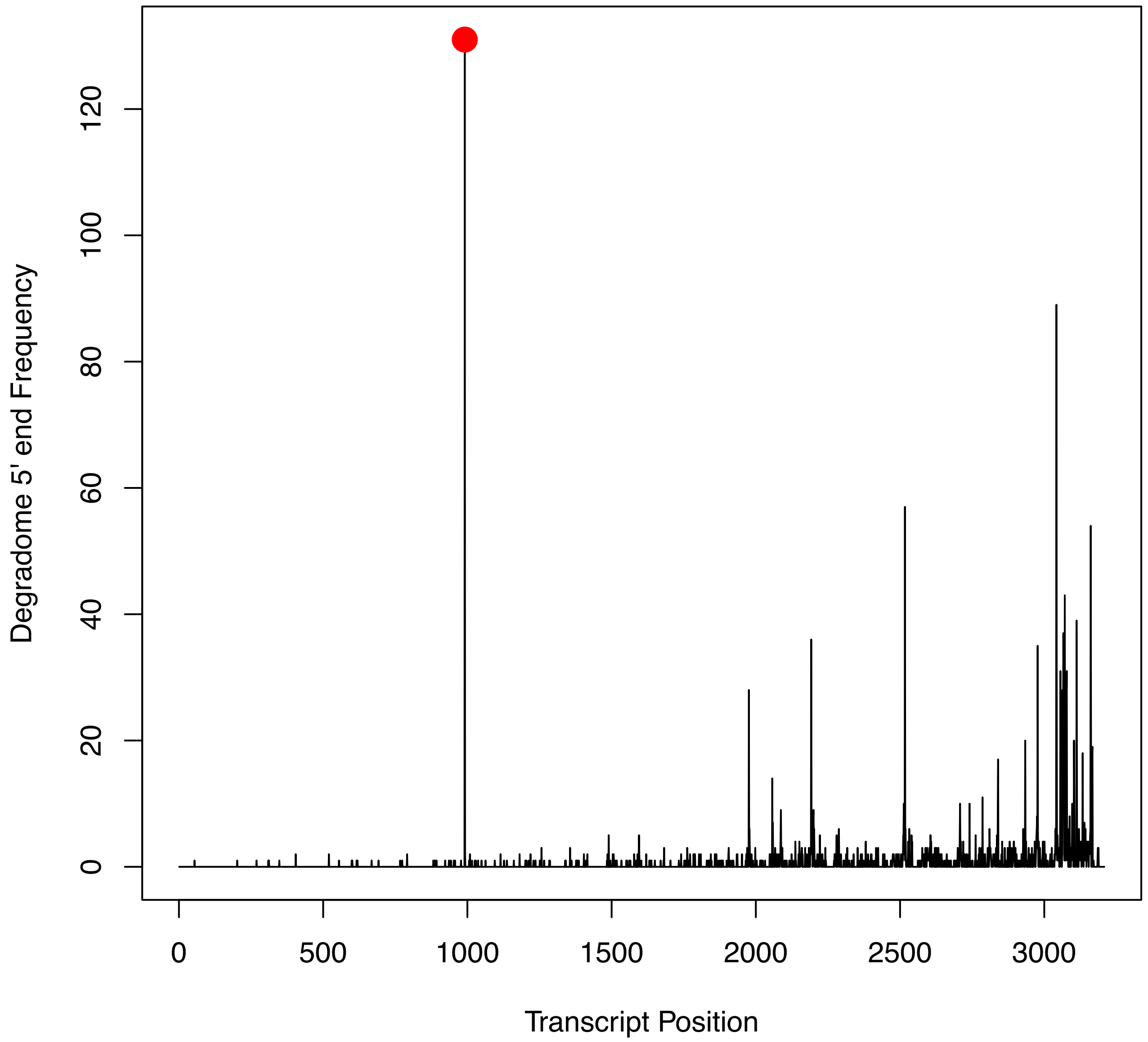

D=Day3
T=HORVU.MOREX.r3.5HG0429100.1
Q=miR166–3p.Cluster_4051
S=991
category=0
p=0.000401219205759529

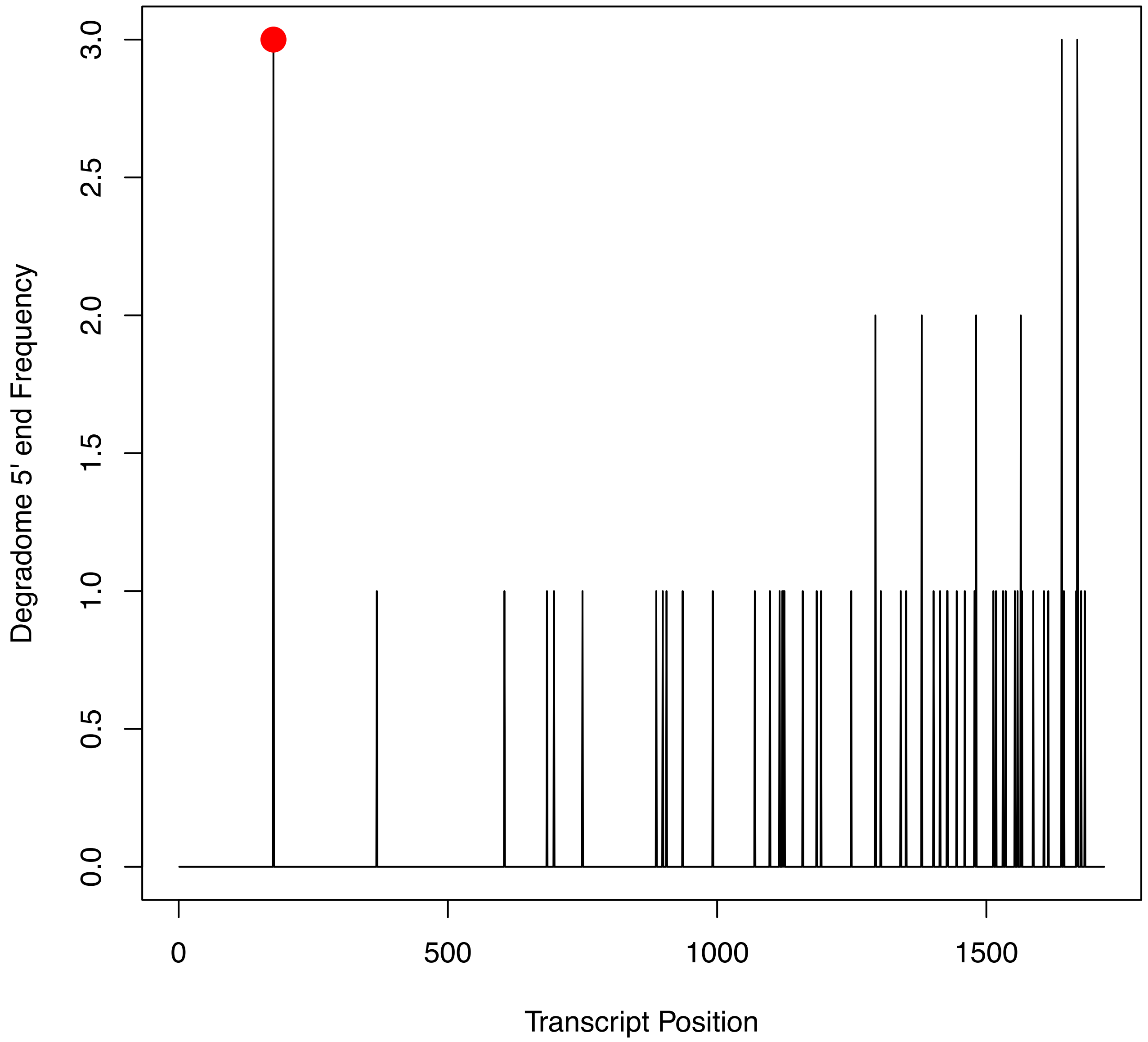

D=Day3
T=HORVU.MOREX.r3.6HG0603530.1
Q=miR166–3p.Cluster_4051
S=176
category=1
p=0.00280510070632434

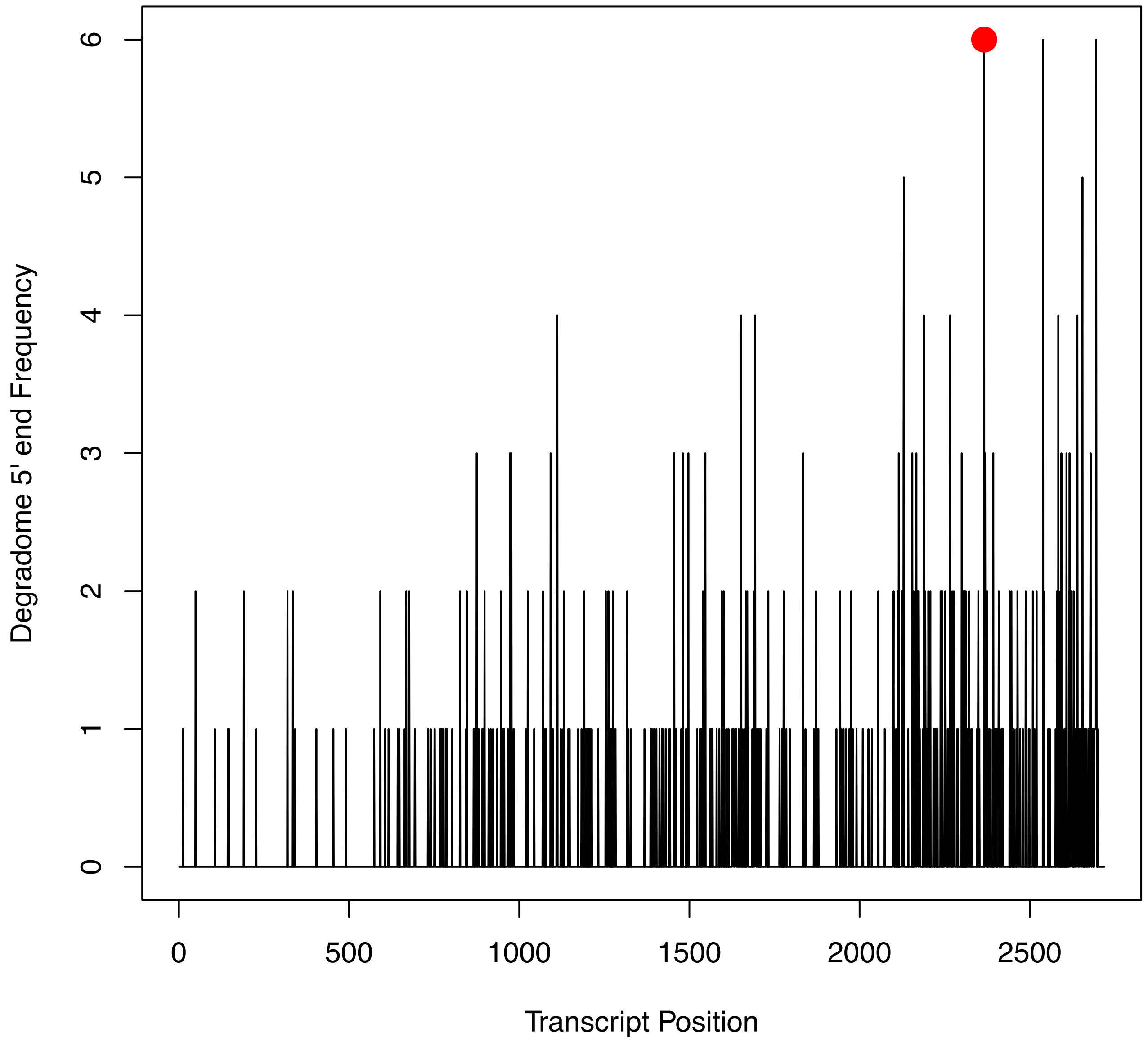

D=Day3
T=HORVU.MOREX.r3.5HG0428840.1
Q=miR167–3p.Cluster_3392
S=2366
category=1
p=0.0304269568801376

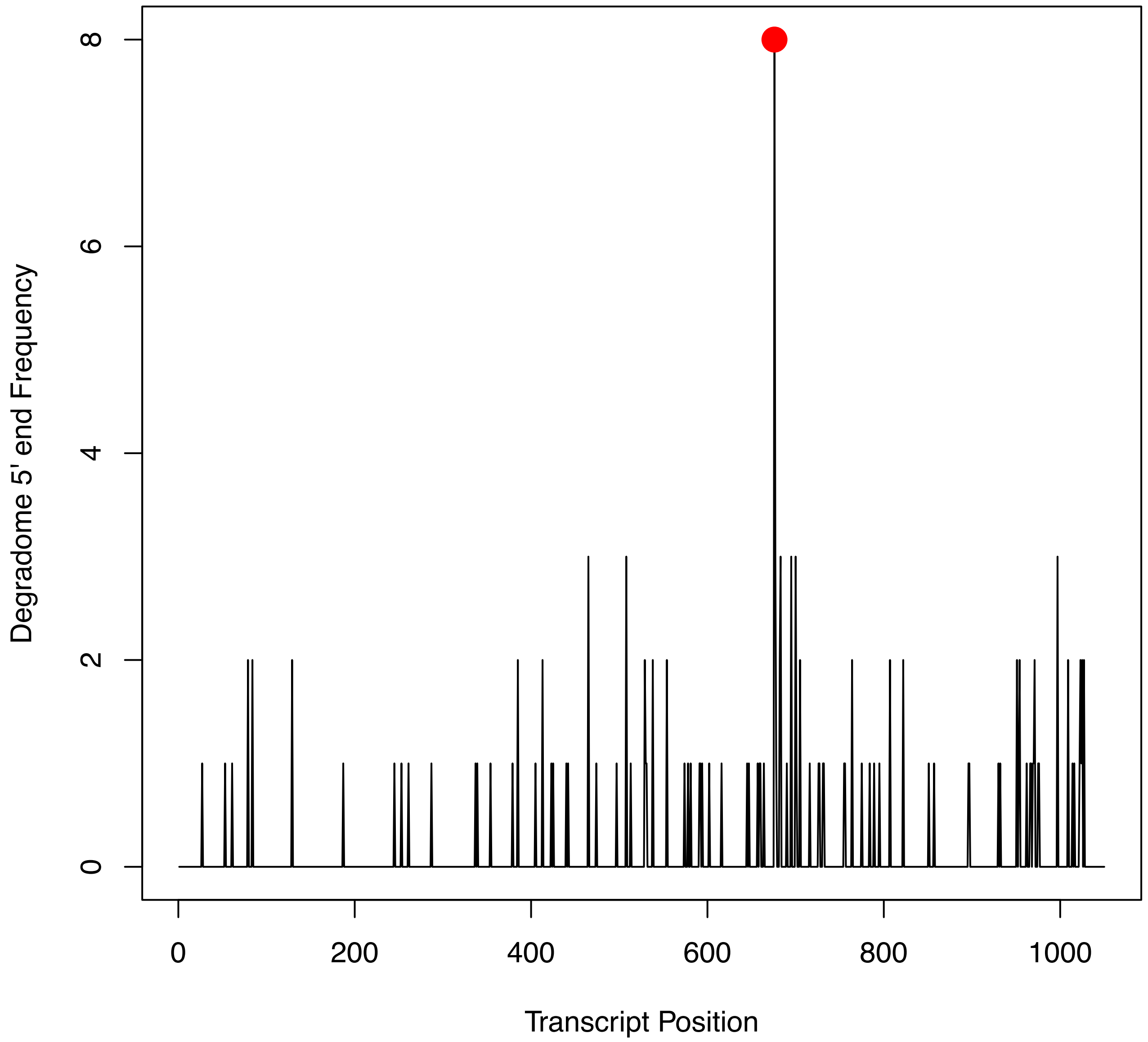

D=Day3
T=HORVU.MOREX.r3.7HG0698540.1
Q=miR167–5p.Cluster_3392.Cluster_3623
S=676
category=0
p=0.0103795494126366

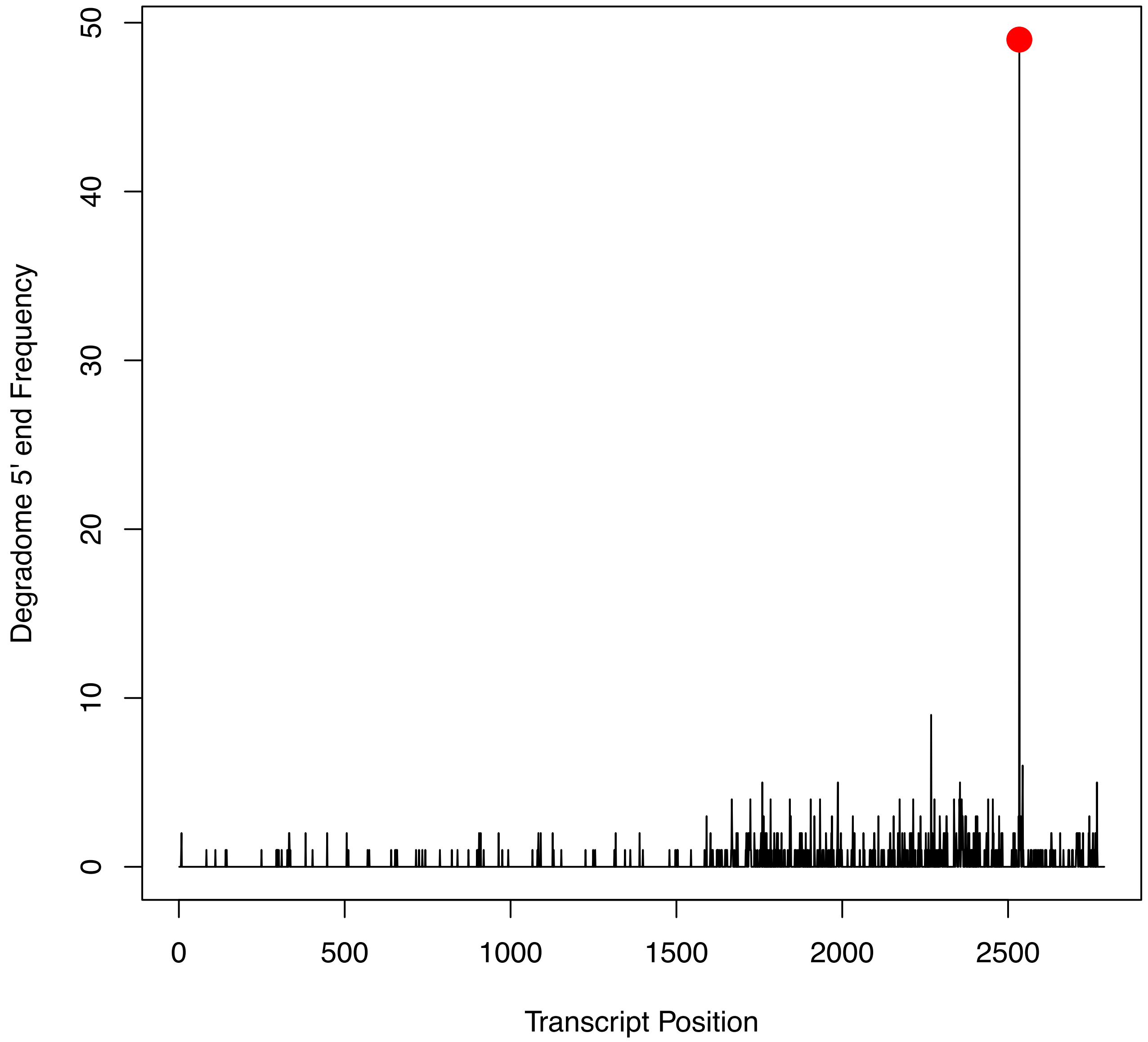

D=Day3
T=HORVU.MOREX.r3.6HG0566320.1
Q=miR167–5p.Cluster_4244
S=2534
category=0
p=0.00160391122025372

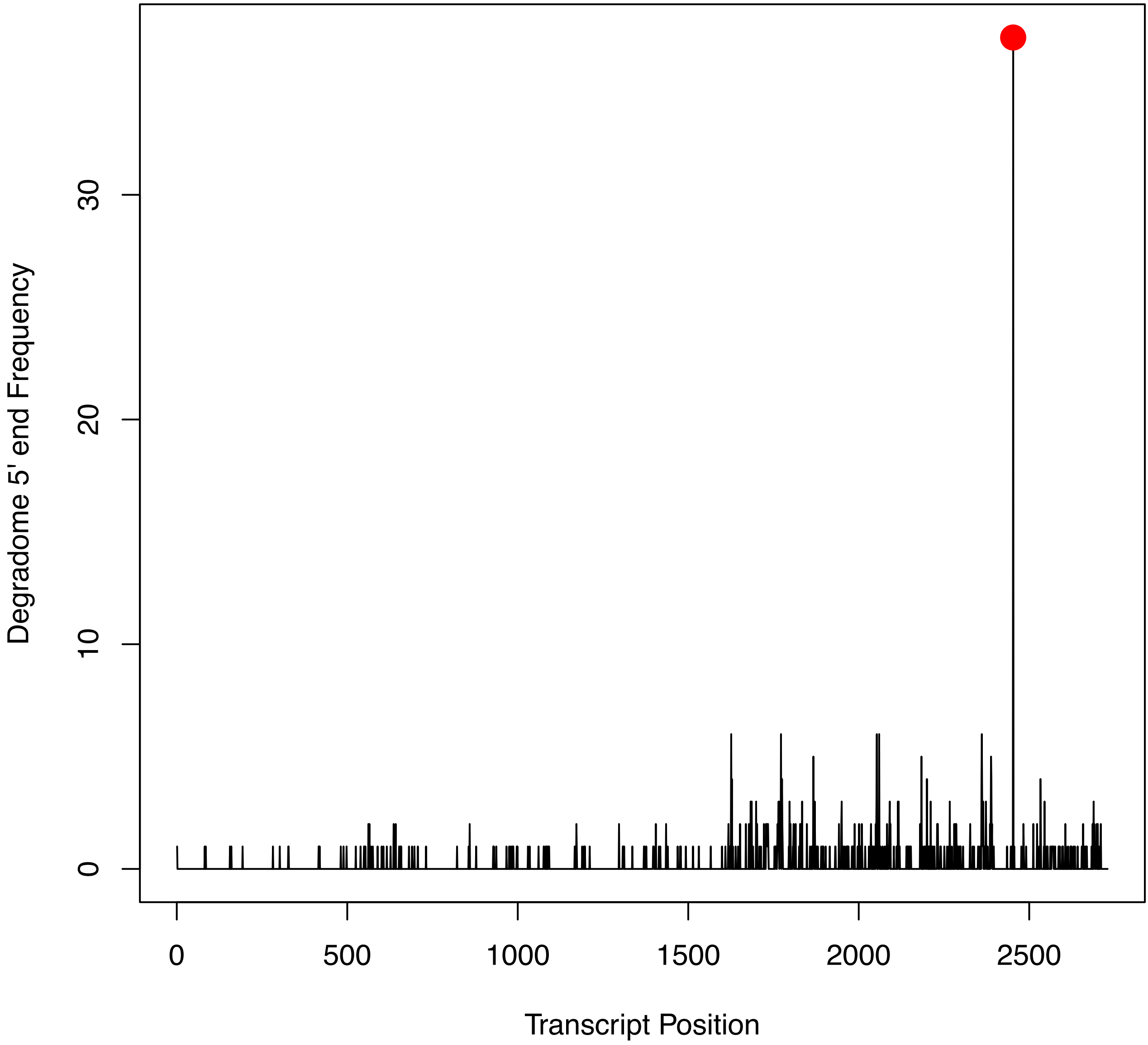

D=Day3
T=HORVU.MOREX.r3.7HG0735280.1
Q=miR167–5p.Cluster_4244
S=2453
category=0
p=0.00120317475131237

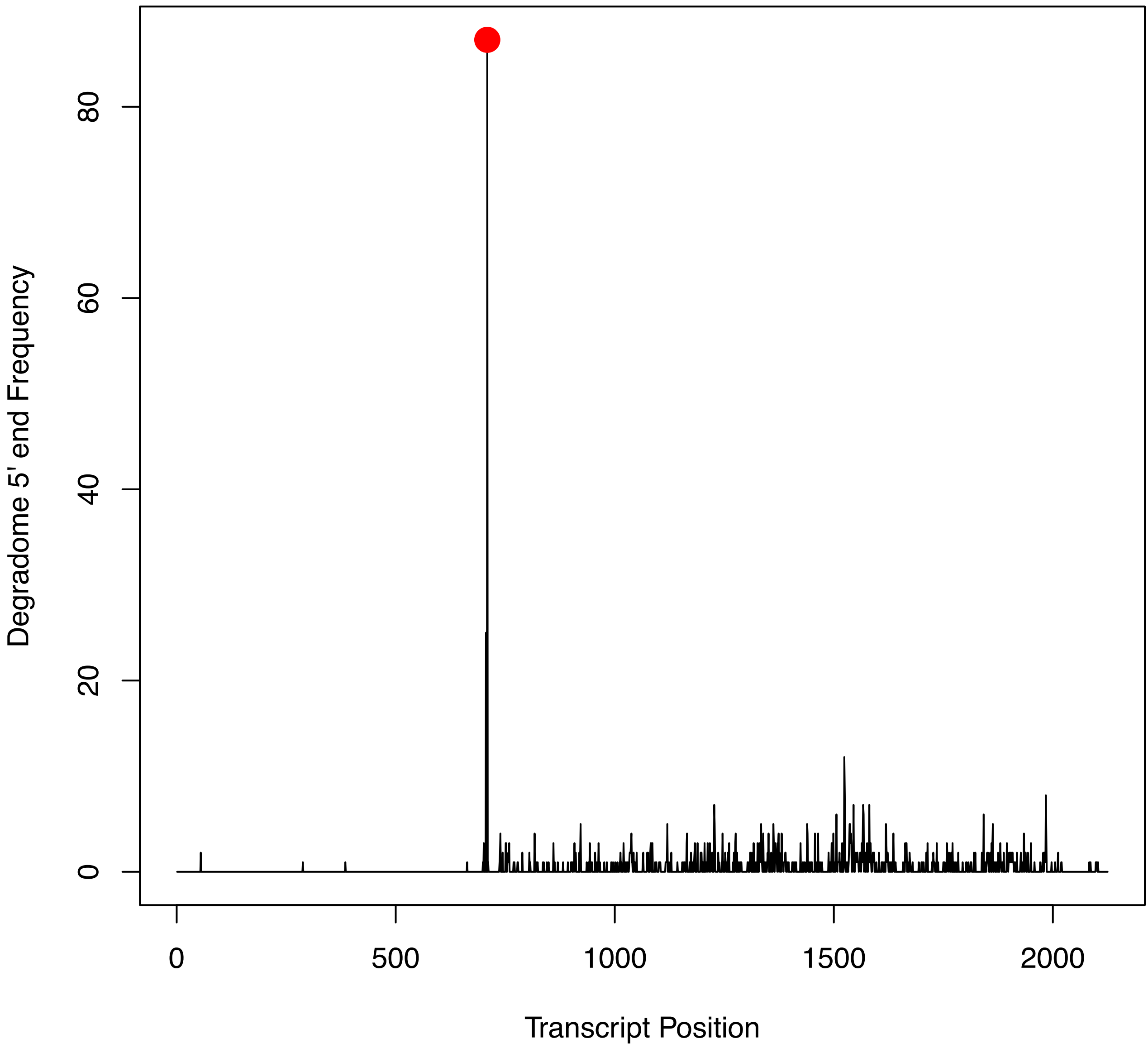

D=Day3
T=HORVU.MOREX.r3.7HG0635740.1
Q=miR171–3p.Cluster_456
S=709
category=0
p=0.000802277434667986

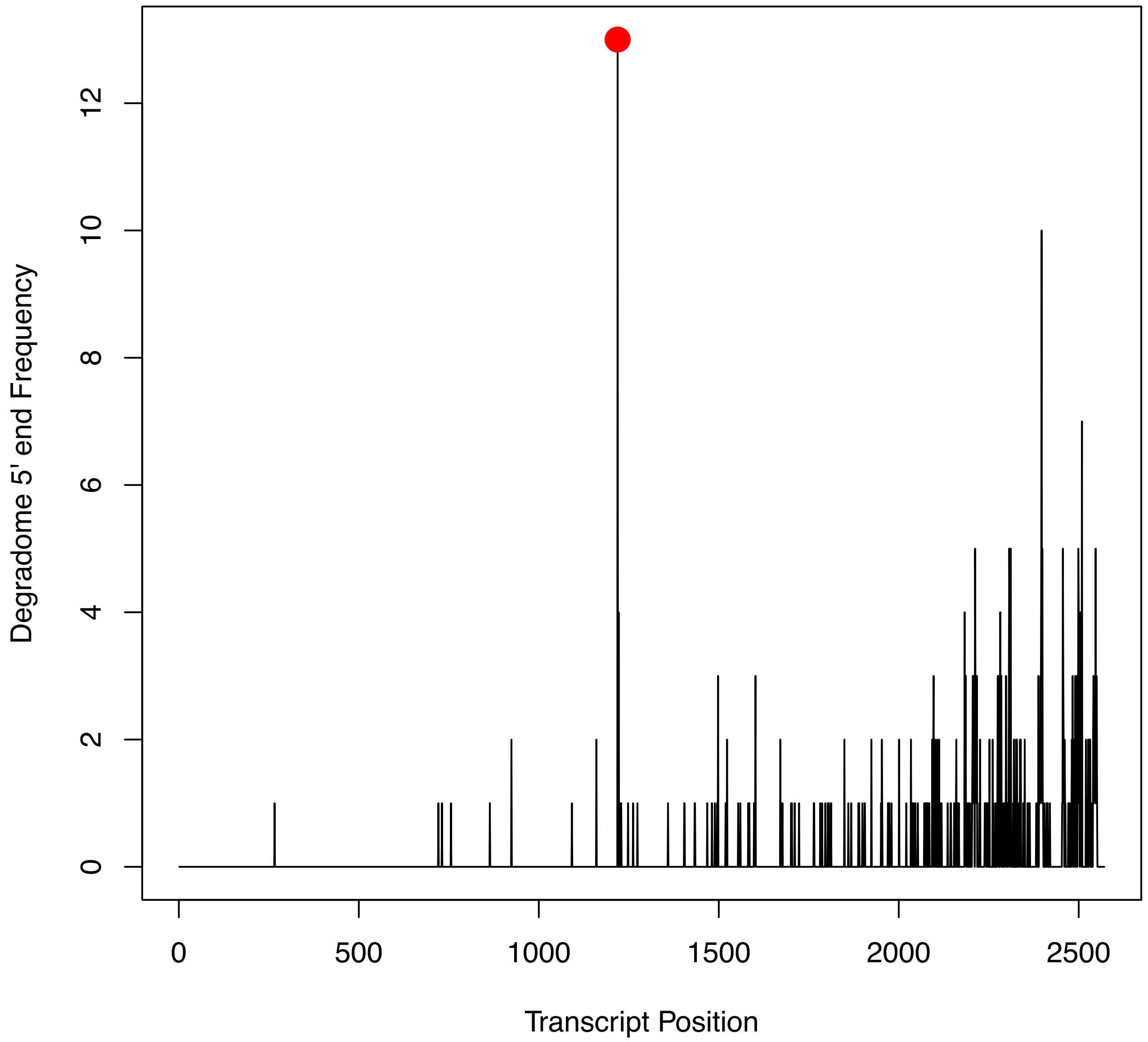

D-Day3
T=HORVU.MOREX.r3.1HG0055960.1
Q=miR171–3p.Cluster_1682
S=1219
category=0
p=0.00160391122025372

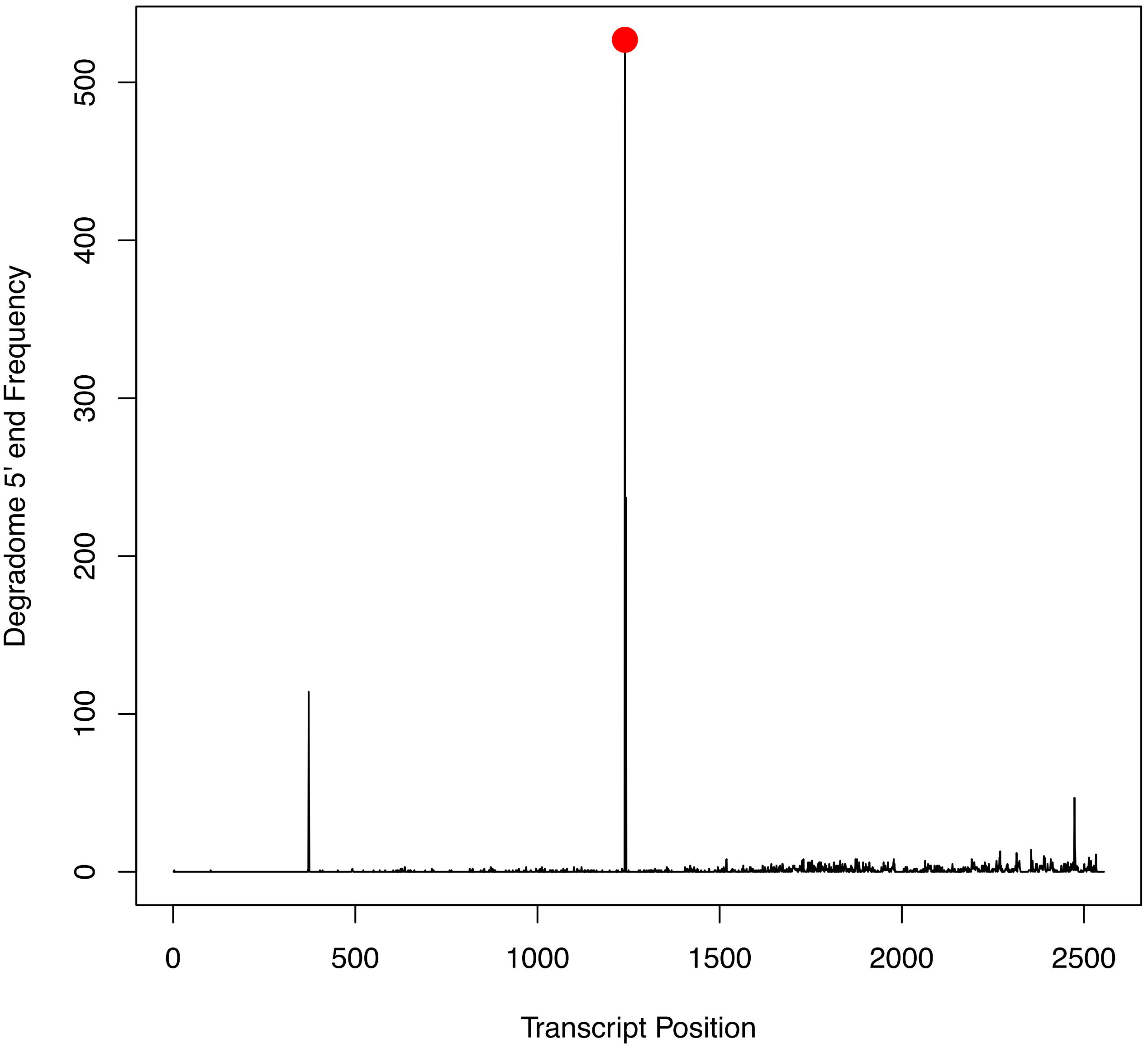

D=Day3
T=HORVU.MOREX.r3.6HG0601750.1
Q=miR171–3p.Cluster_1682
S=1240
category=0
p=0.000802277434667986

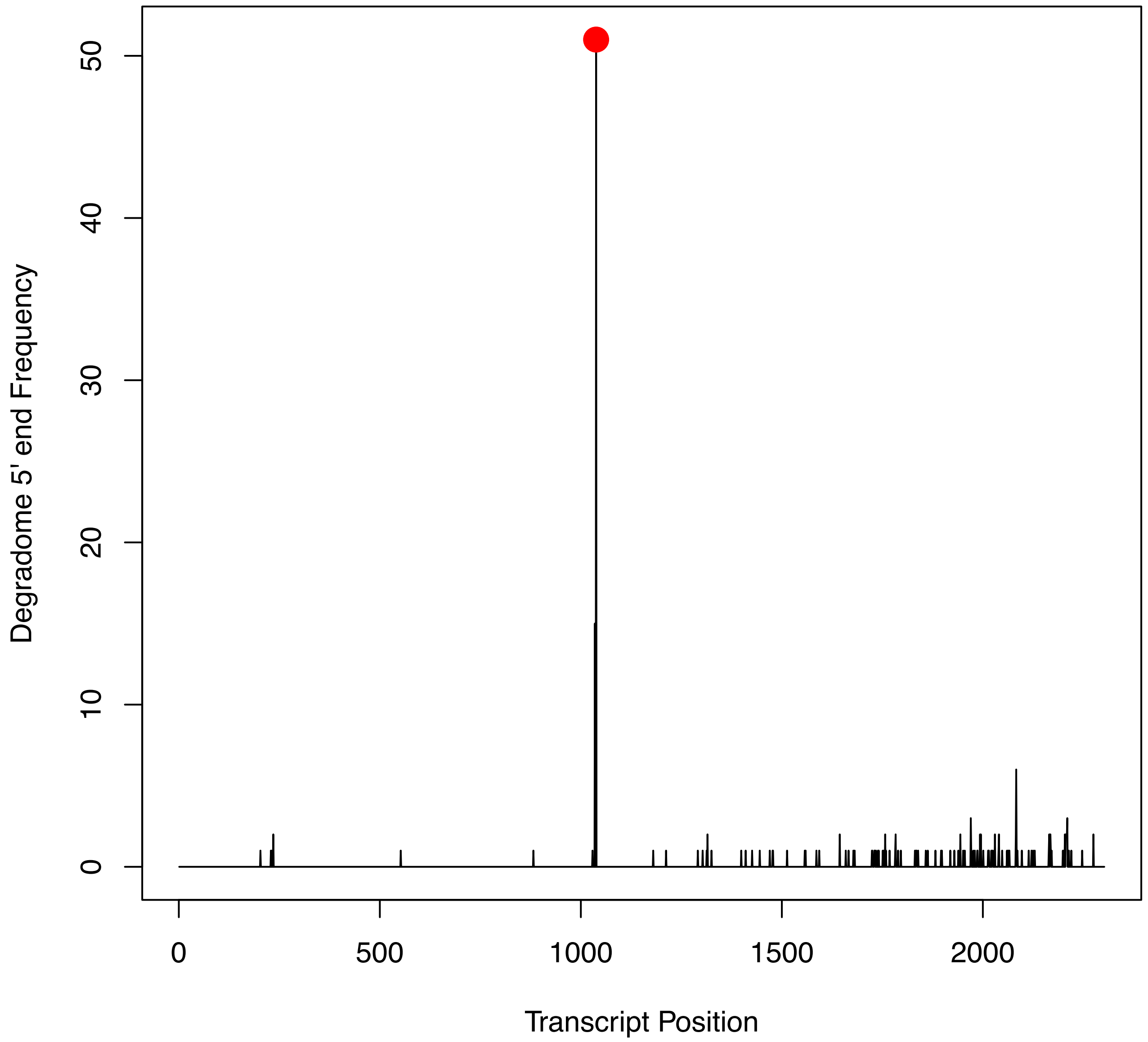

D=Day3
T=HORVU.MOREX.r3.4HG0415480.1
Q=miR171–3p.Cluster_3461
S=1038
category=0
p=0.000401219205759529

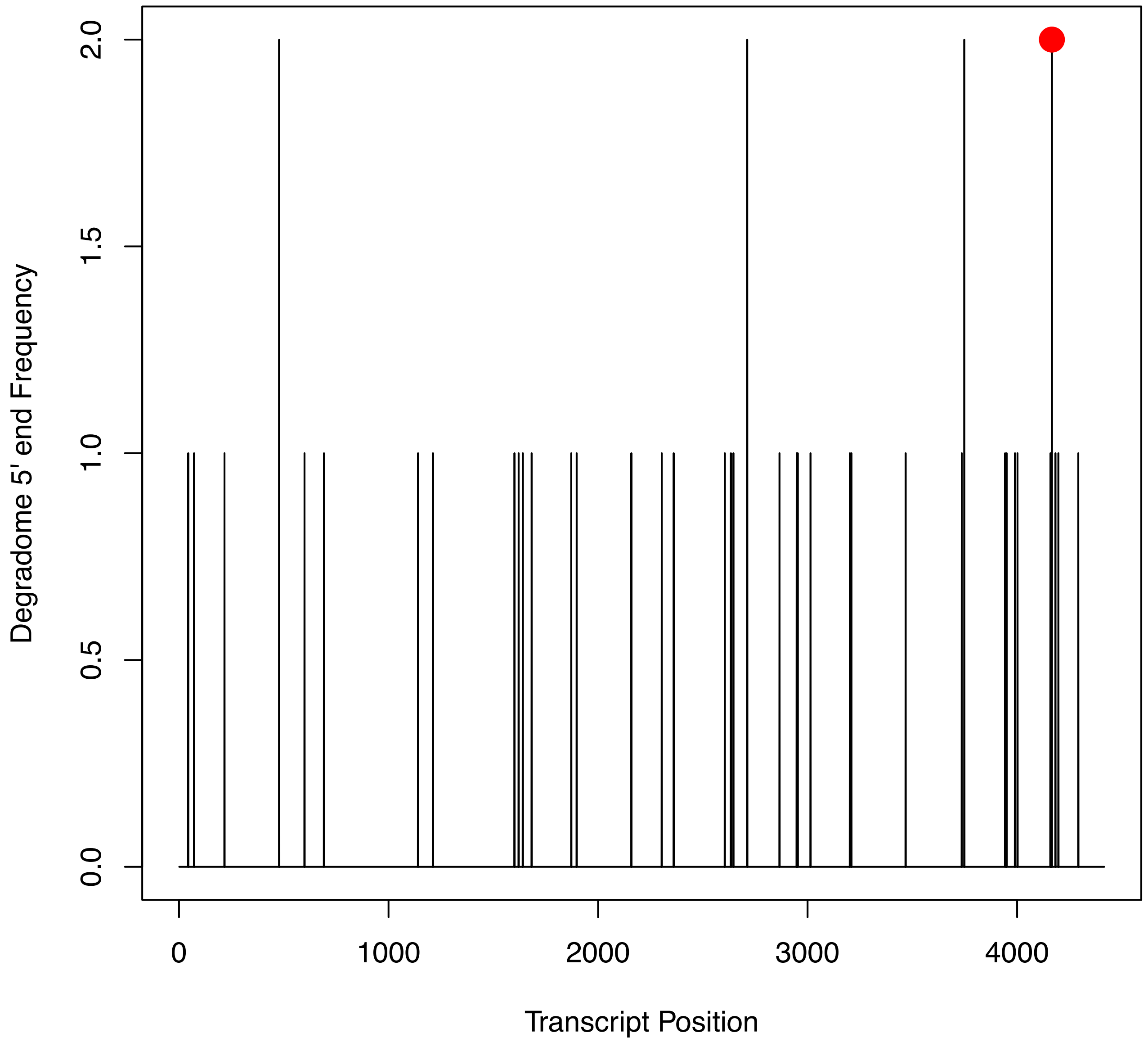

D=Day3
T=HORVU.MOREX.r3.2HG0096080.1
Q=miR319–3p.Cluster_2046
S=4166
category=1
p=0.0136700126820556

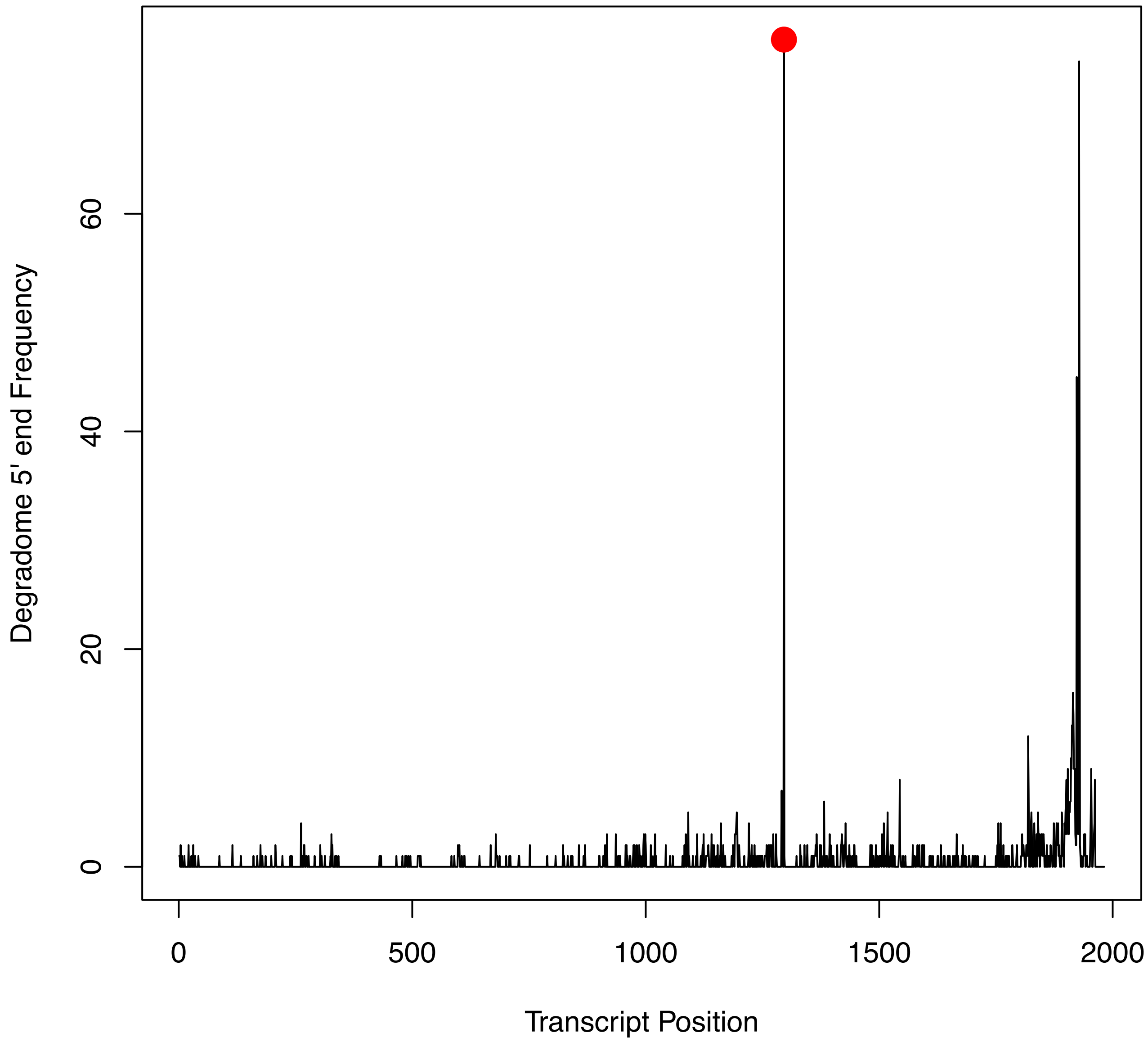

D=Day3
T=HORVU.MOREX.r3.2HG0152890.1
Q=miR319–3p.Cluster_2046
S=1296
category=0
p=0.000802277434667986

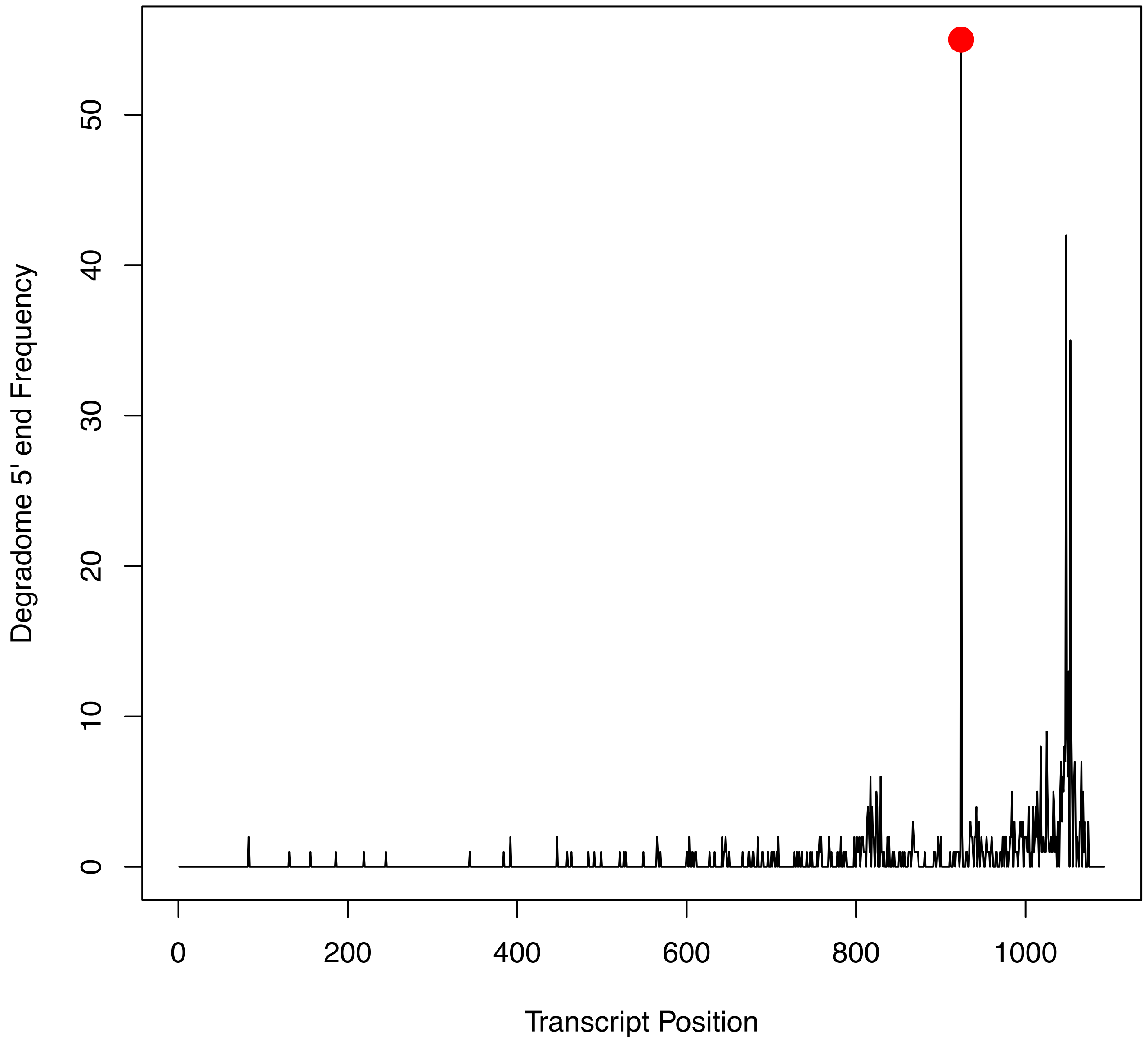

D=Day3
T=HORVU.MOREX.r3.3HG0243310.1
Q=miR319–3p.Cluster_2046
S=924
category=0
p=0.0178964098178904

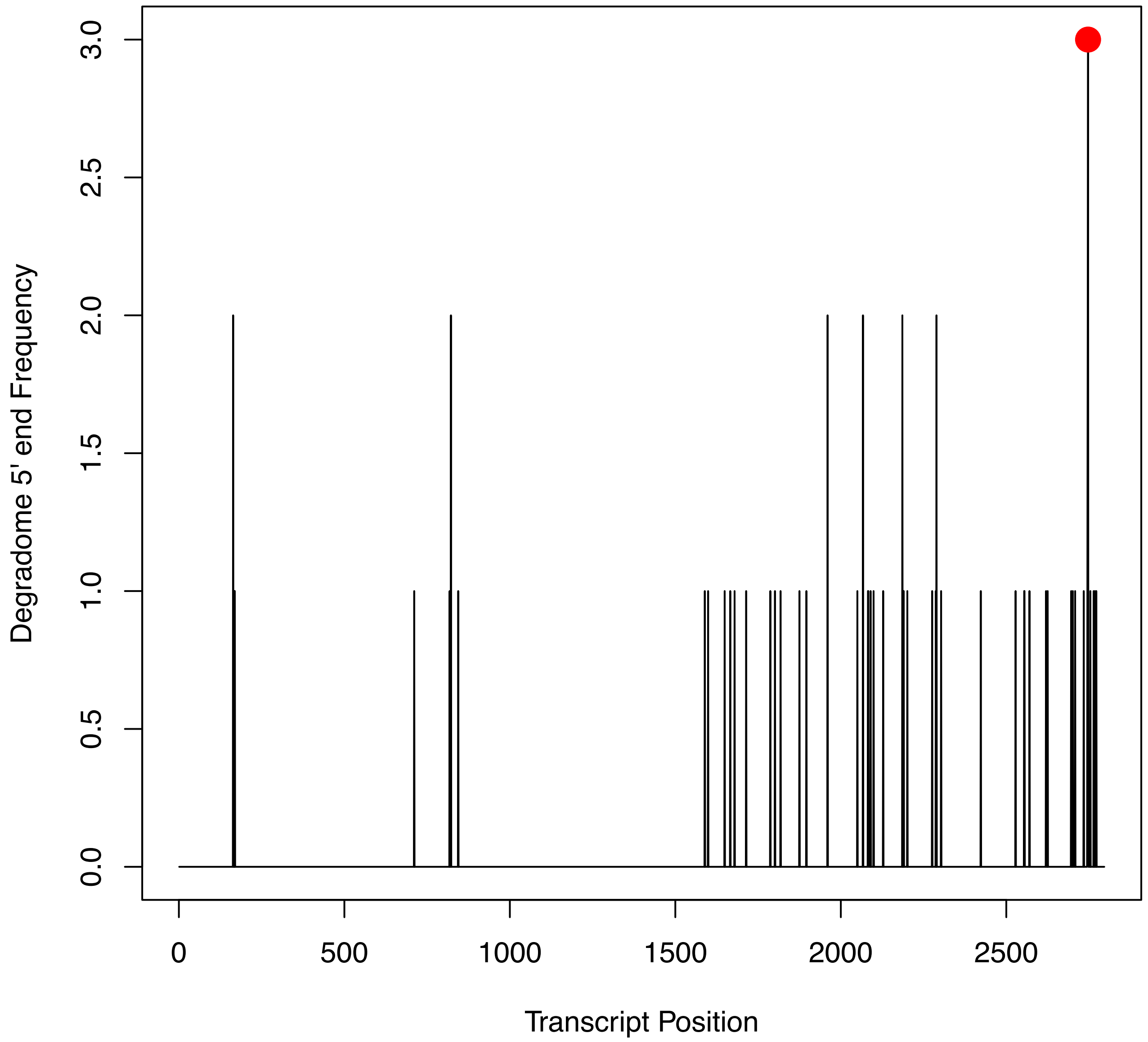

D=Day3
T=HORVU.MOREX.r3.5HG0482600.1
Q=miR393–5p.Cluster_1822
S=2747
category=0
p=0.0159238420447354

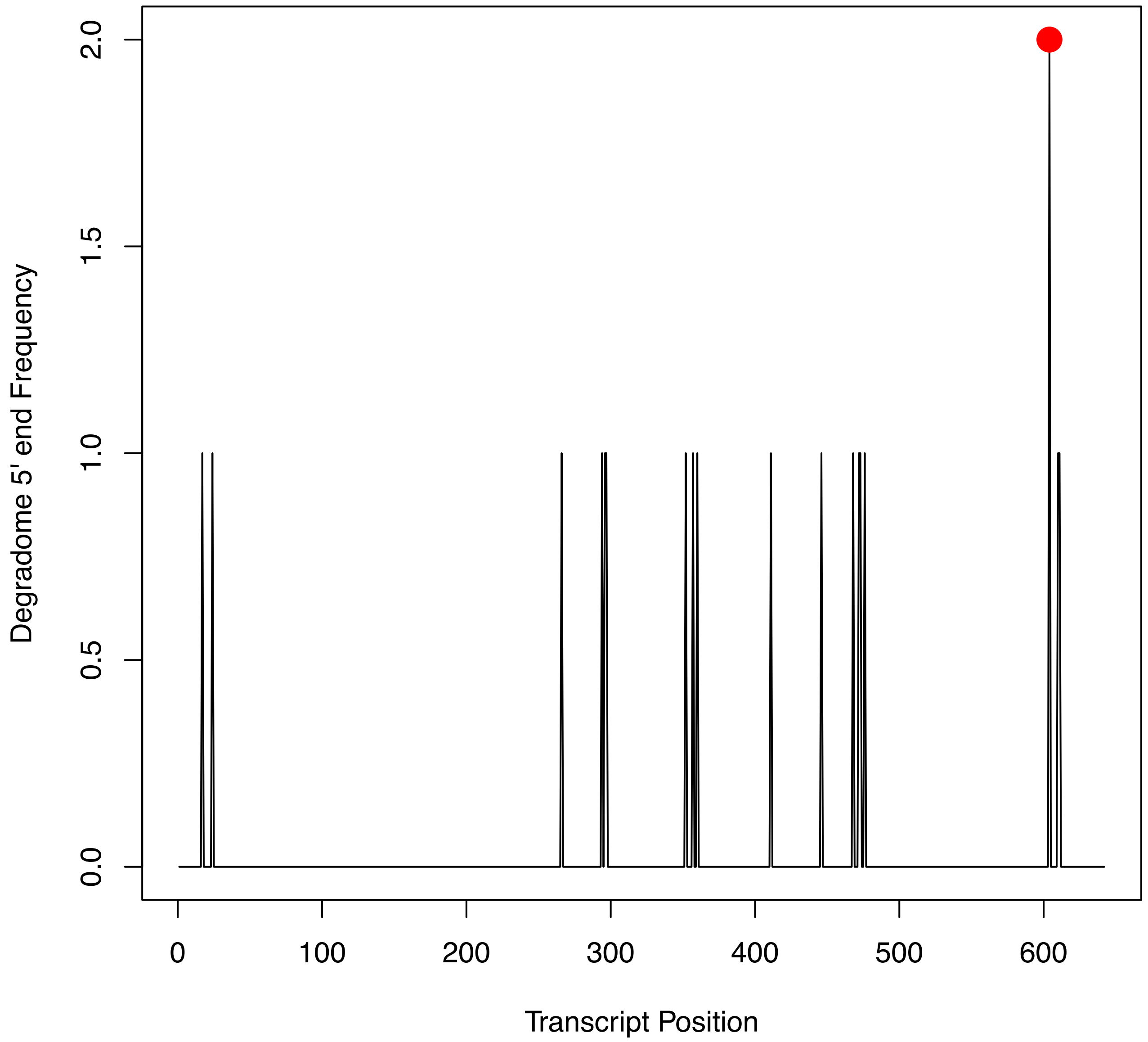

D=Day3
T=HORVU.MOREX.r3.1HG0065890.1
Q=miR396–3p.Cluster_5480
S=604
category=0
p=0.0190780520607708

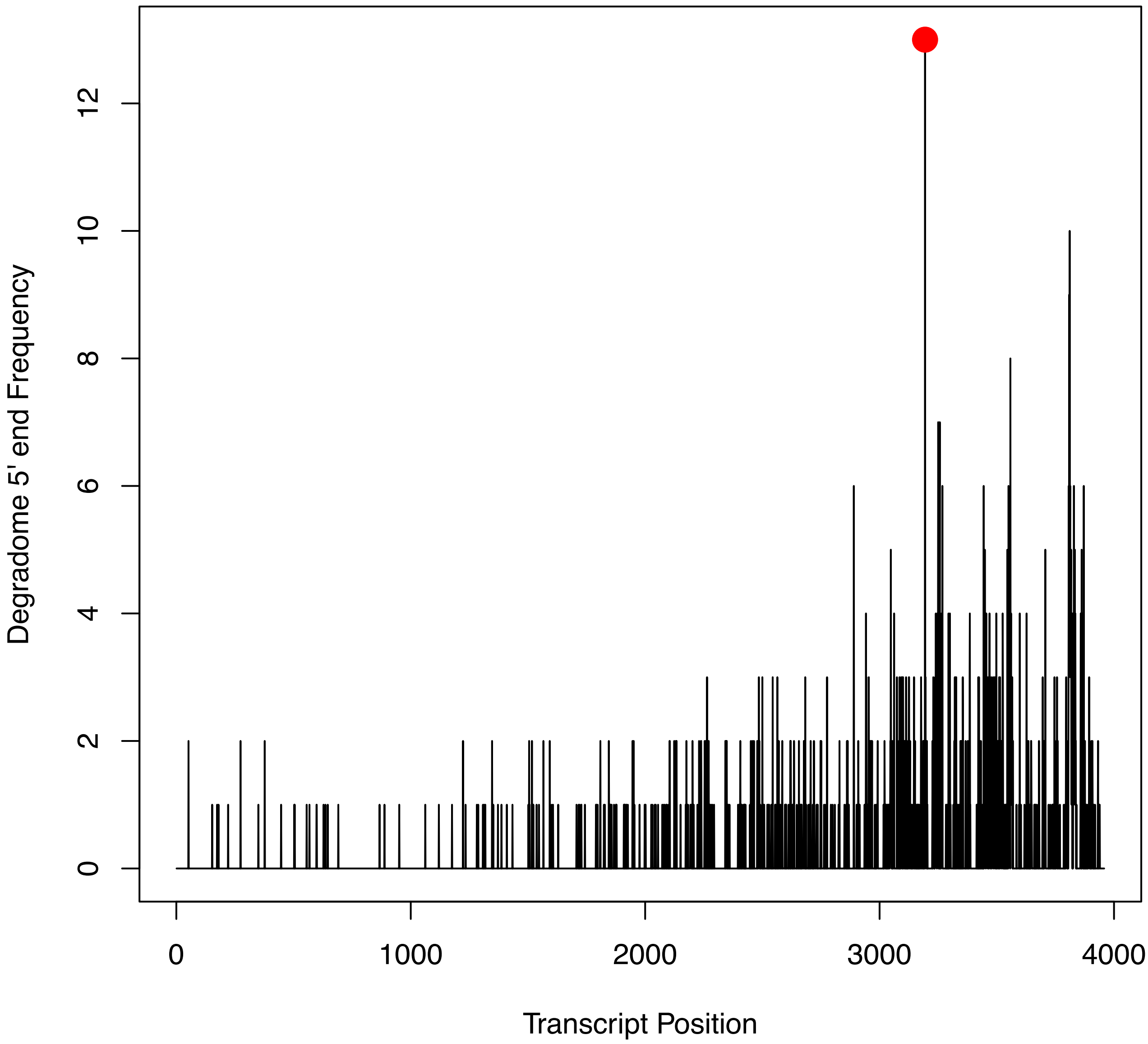

D=Day3
T=HORVU.MOREX.r3.1HG0073090.1
Q=miR396–5p.Cluster_1803
S=3194
category=0
p=0.0171078576313557

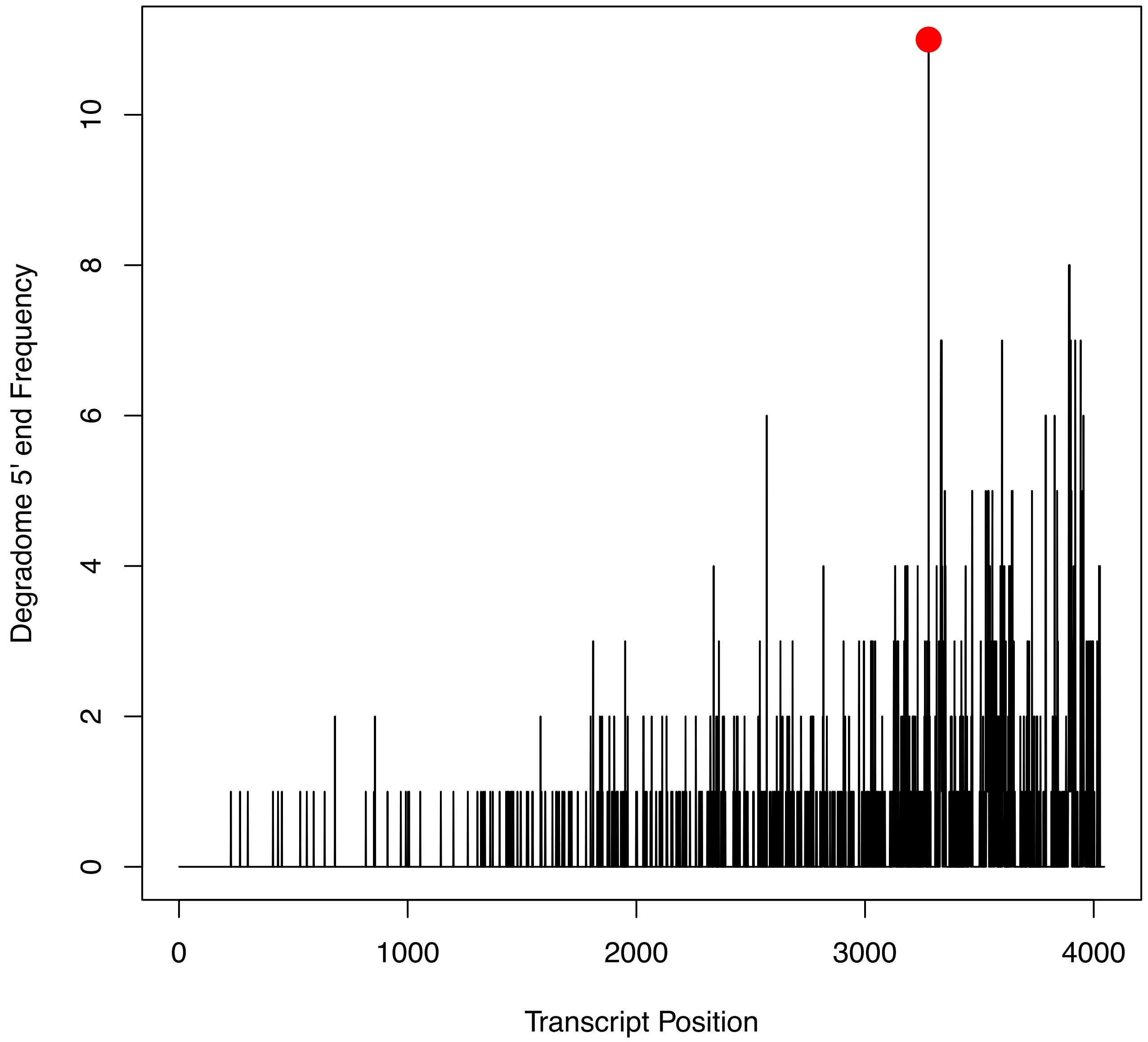

D=Day3
T=HORVU.MOREX.r3.1HG0073090.2
Q=miR396–5p.Cluster_1803
S=3278
category=0
p=0.0159238420447354

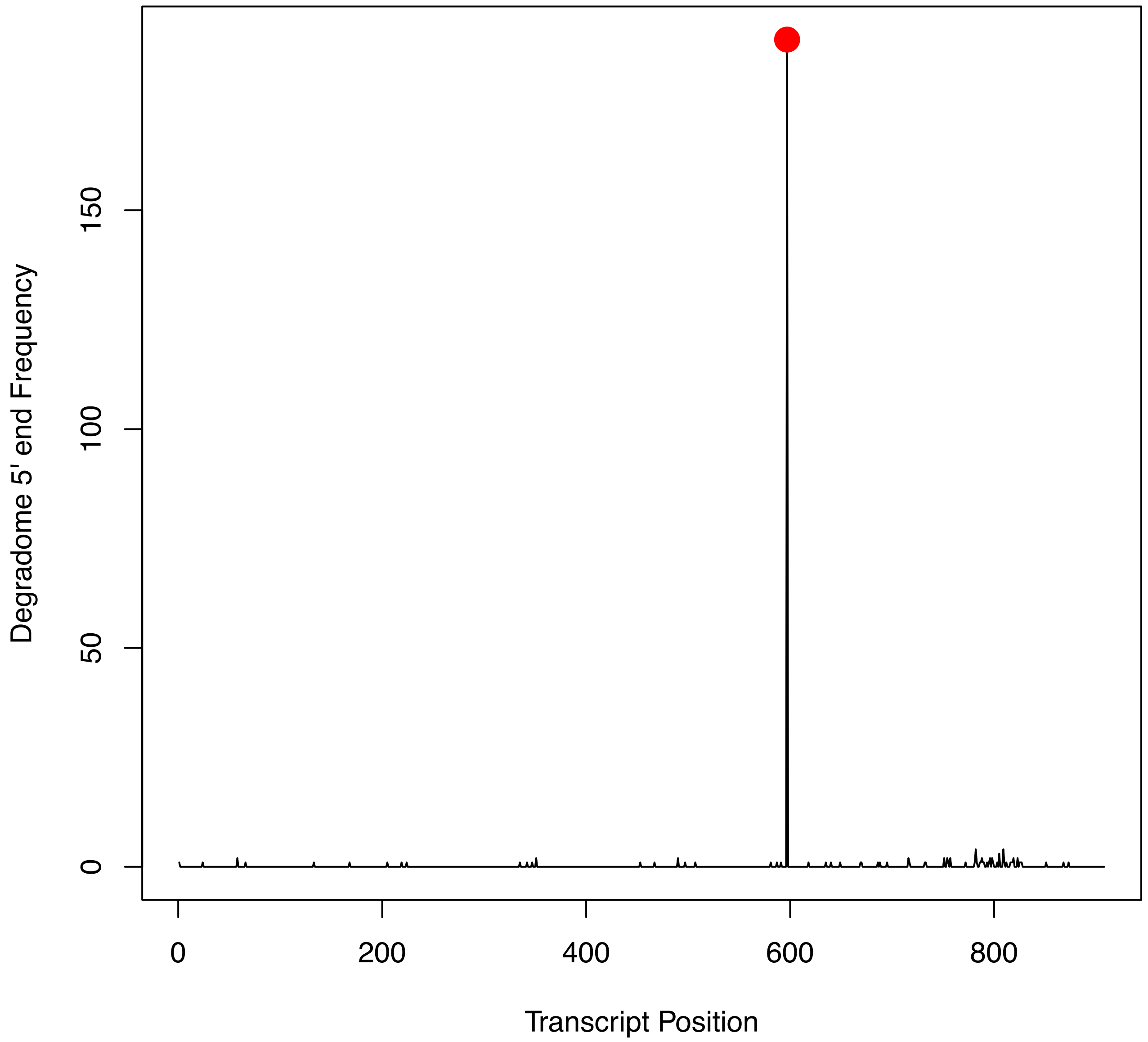

D=Day3
T=HORVU.MOREX.r3.2HG0186750.1
Q=miR396–5p.Cluster_1803
S=597
category=0
p=0.00160391122025372

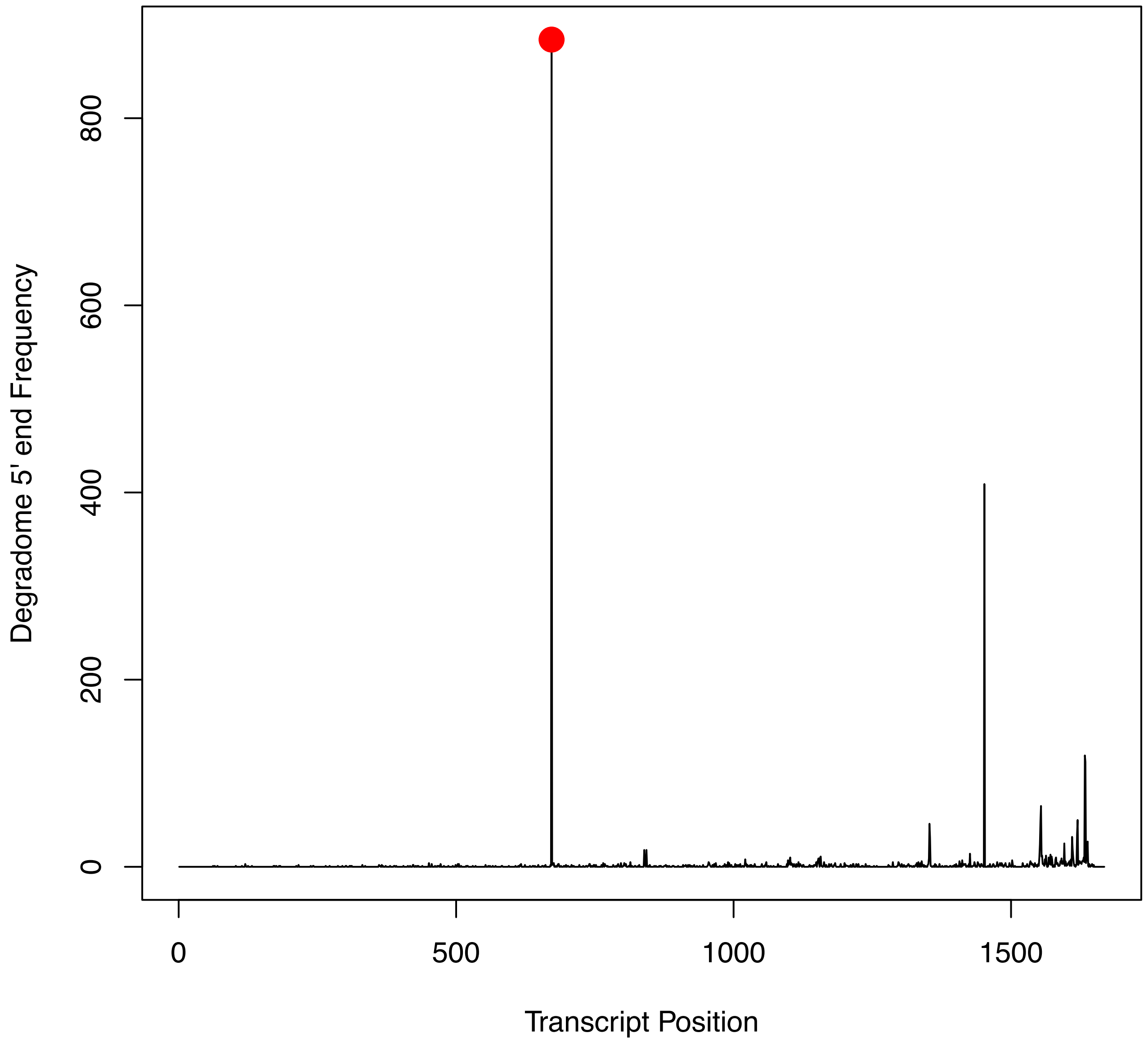

D=Day3
T=HORVU.MOREX.r3.2HG0193490.1
Q=miR396–5p.Cluster_1803
S=672
category=0
p=0.000401219205759529

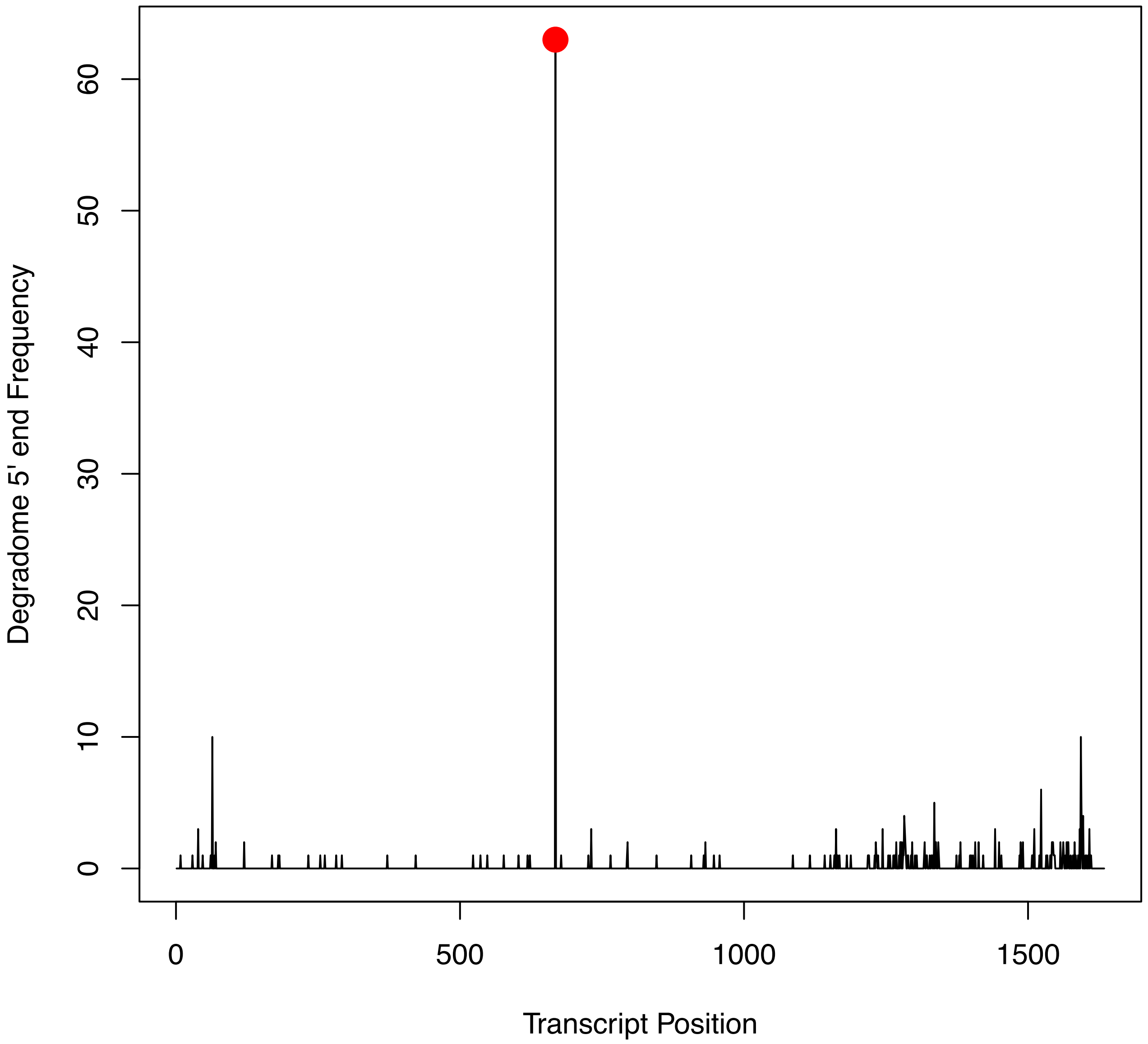

D=Day3
T=HORVU.MOREX.r3.4HG0334080.1
Q=miR396–5p.Cluster_1803
S=668
category=0
p=0.000802277434667986

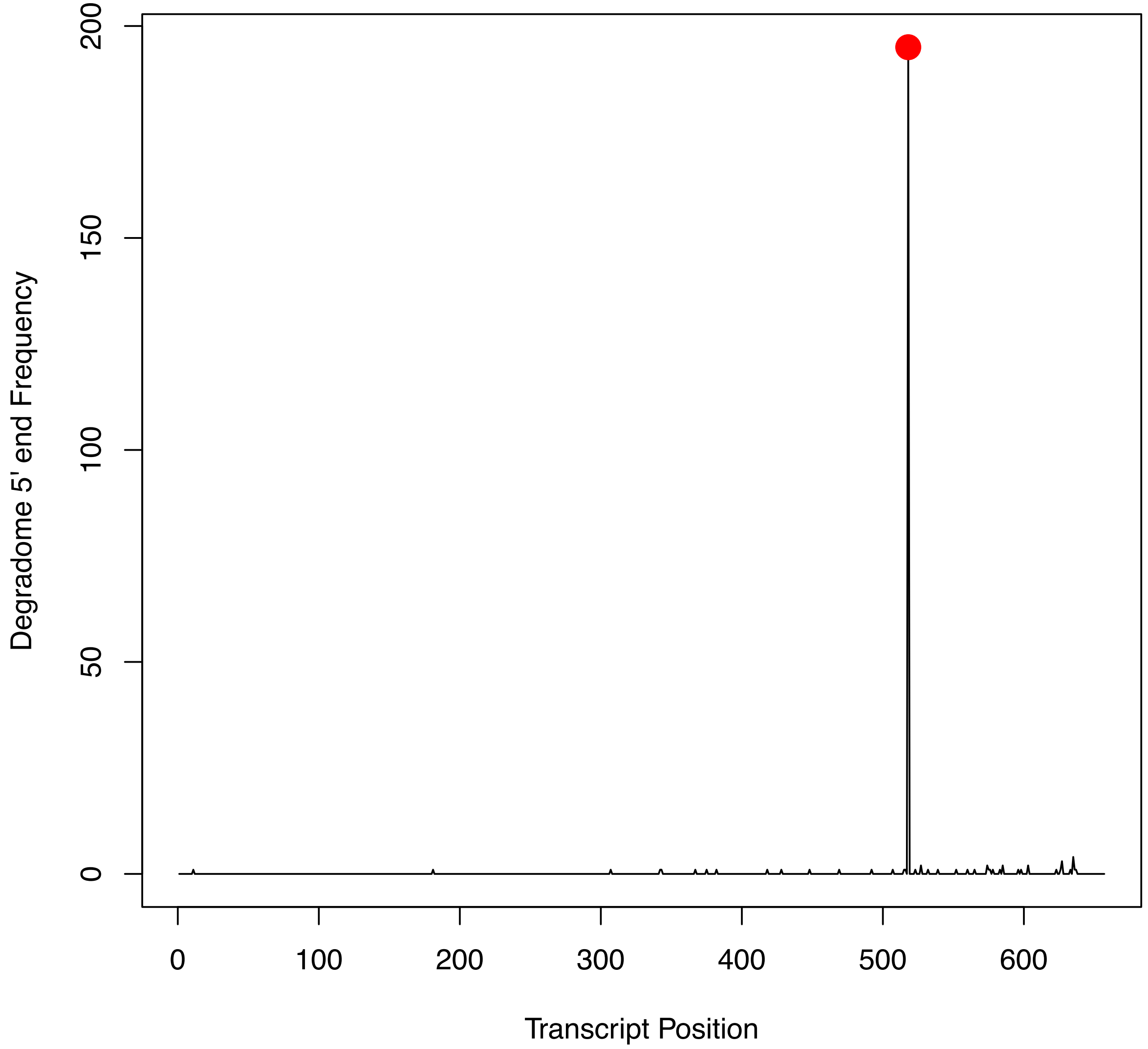

D=Day3
T=HORVU.MOREX.r3.6HG0603870.1
Q=miR396–5p.Cluster_1803
S=518
category=0
p=0.00120317475131237

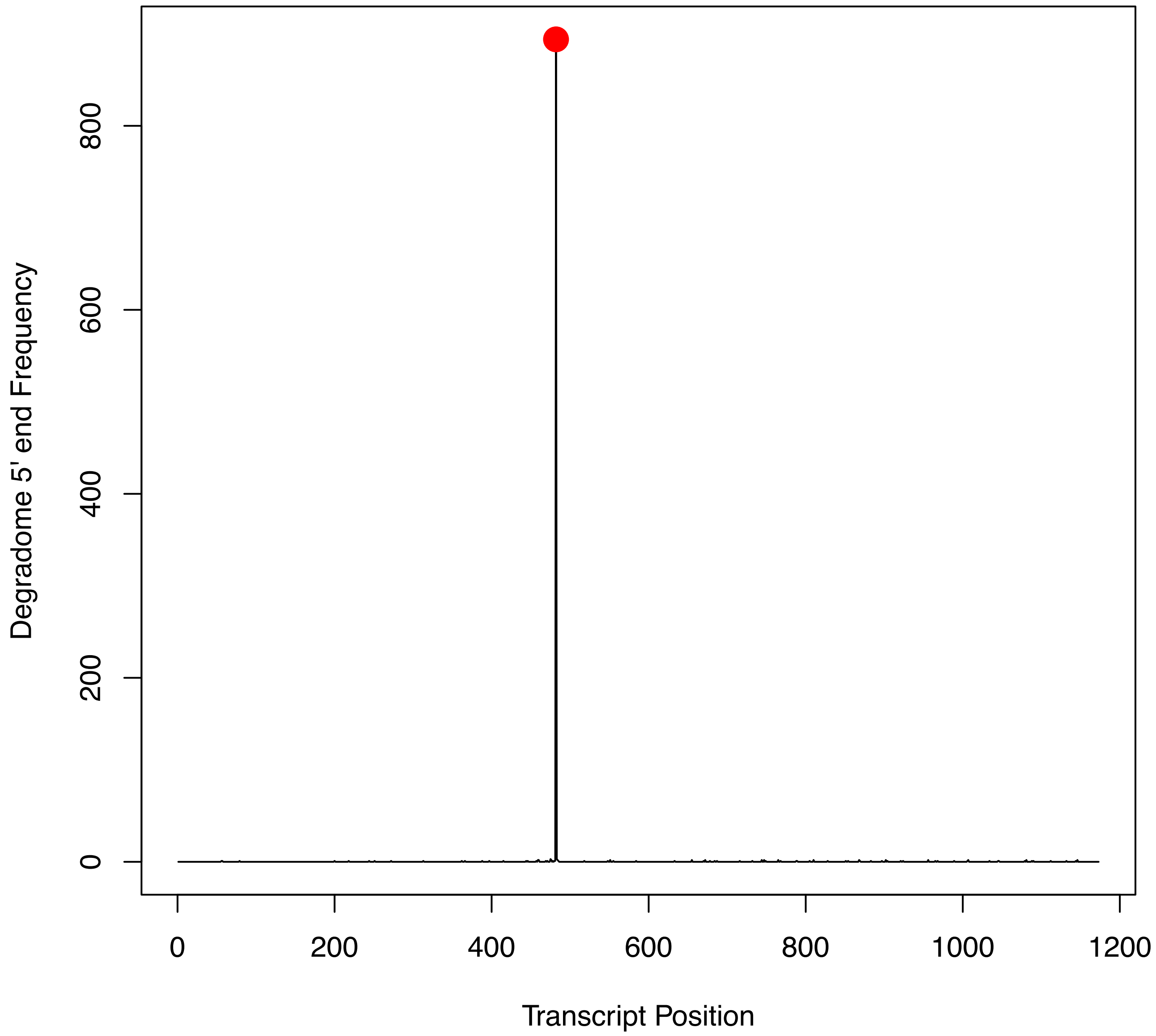

D=Day3
T=HORVU.MOREX.r3.6HG0606810.1
Q=miR396–5p.Cluster_1803
S=482
category=0
p=0.00240490187314257

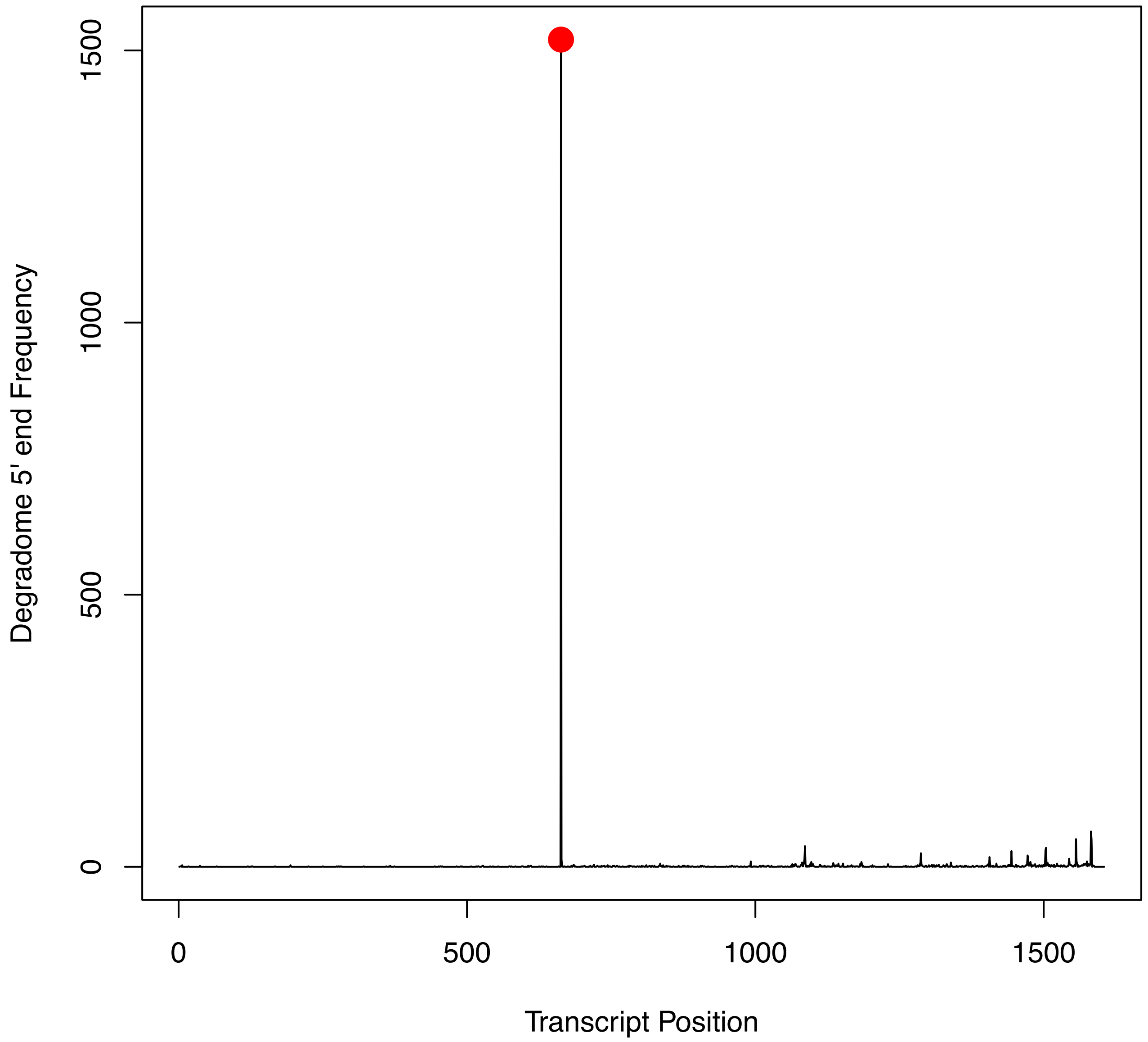

D=Day3
T=HORVU.MOREX.r3.7HG0641080.1
Q=miR396–5p.Cluster_1803
S=663
category=0
p=0.00320524990930515

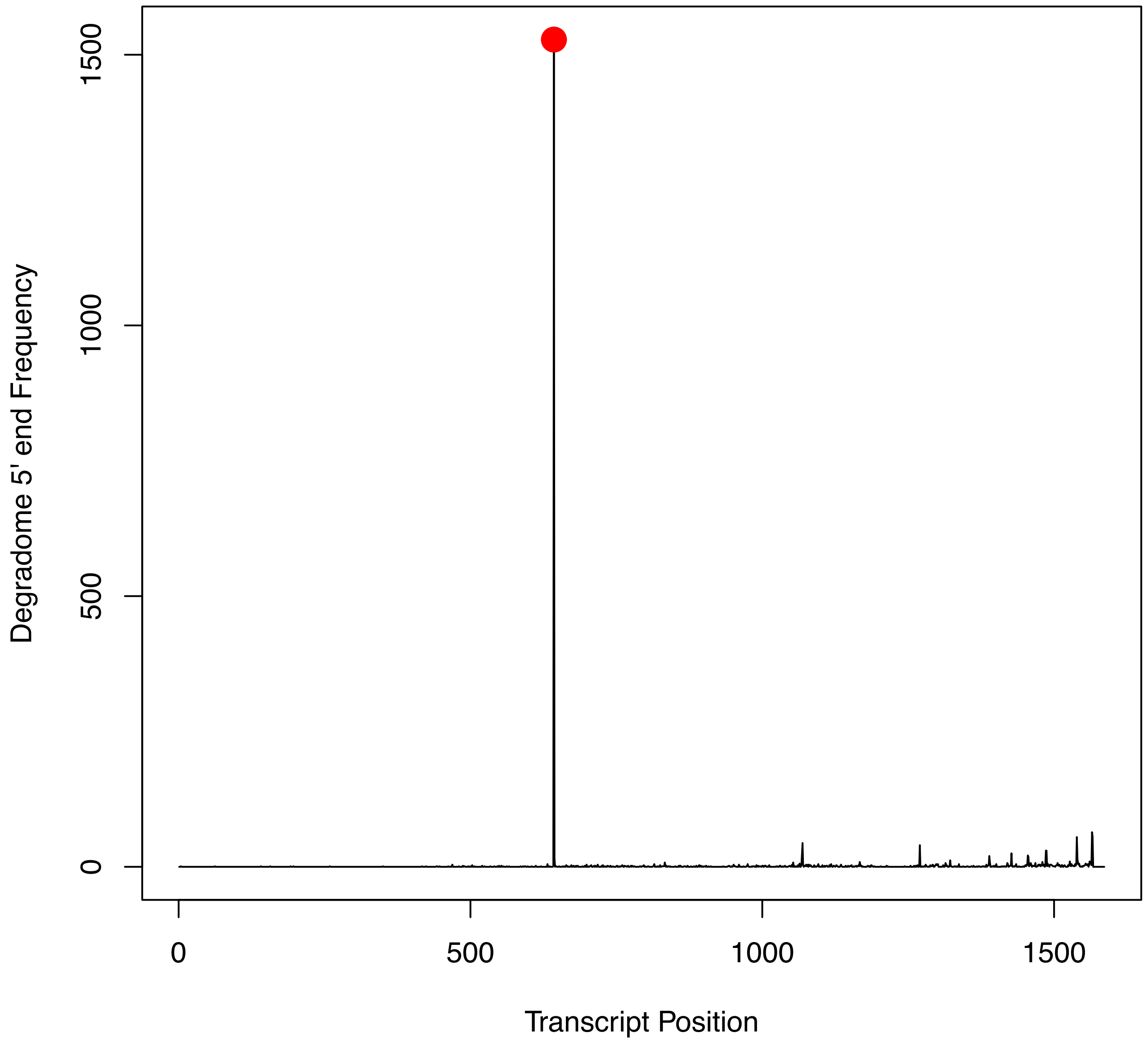

D=Day3
T=HORVU.MOREX.r3.7HG0641080.2
Q=miR396–5p.Cluster_1803
S=643
category=0
p=0.00280515618608268

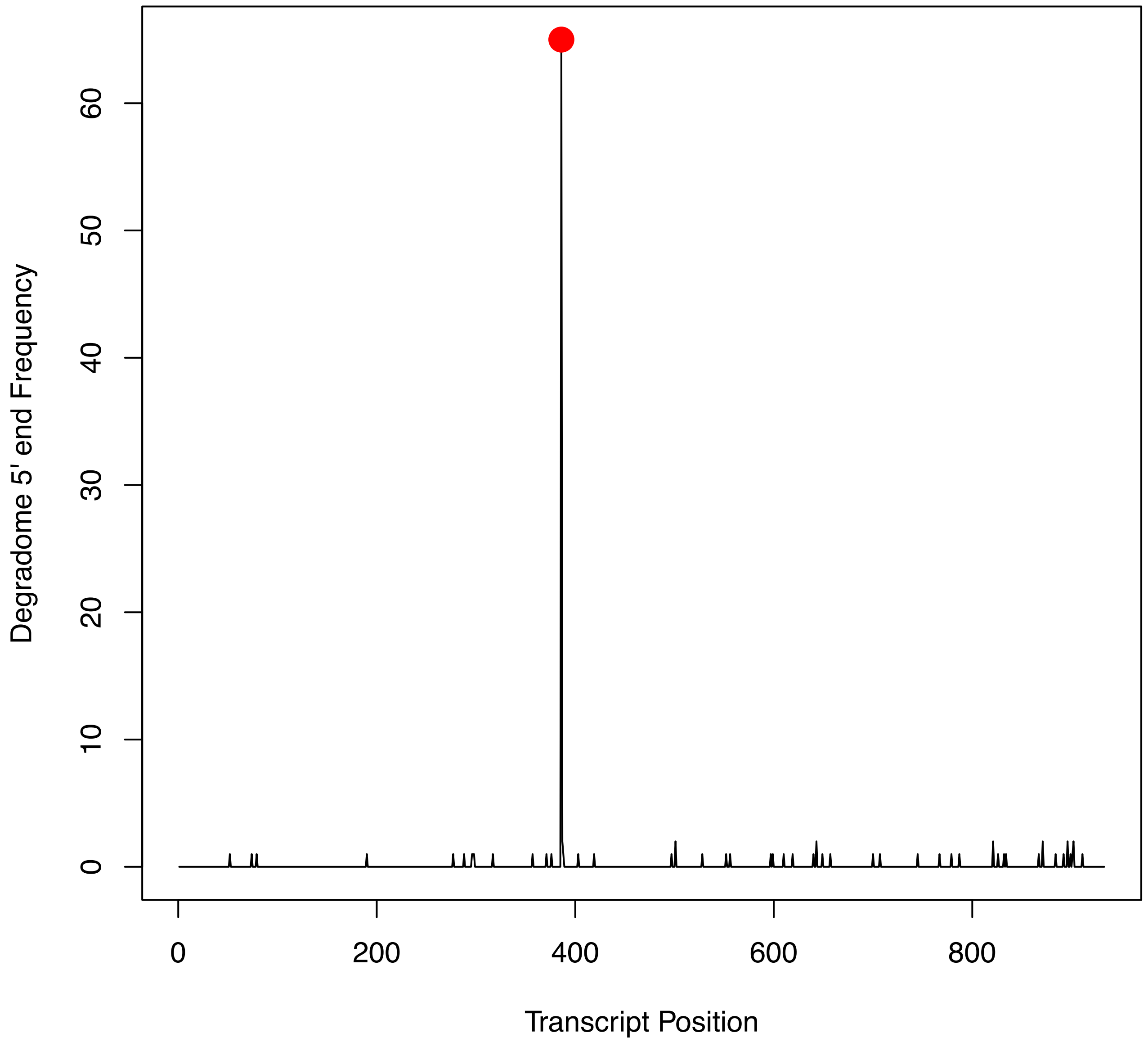

D=Day3
T=HORVU.MOREX.r3.7HG0662690.1
Q=miR396–5p.Cluster_1803
S=386
category=0
p=0.00360518310724178

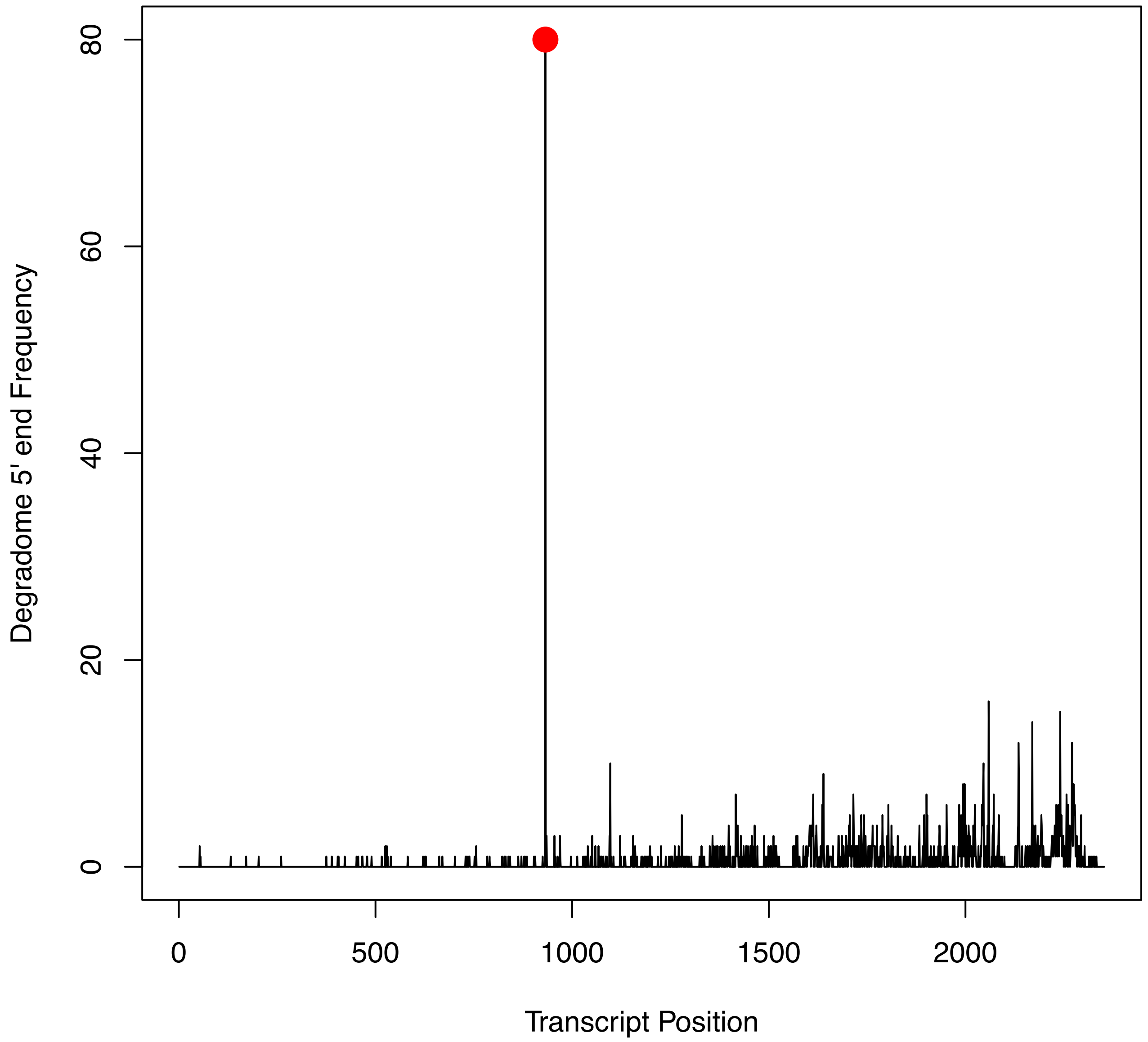

D=Day3
T=HORVU.MOREX.r3.4HG0339430.1
Q=miR396–5p.Cluster_5480
S=932
category=0
p=0.00480402019326576

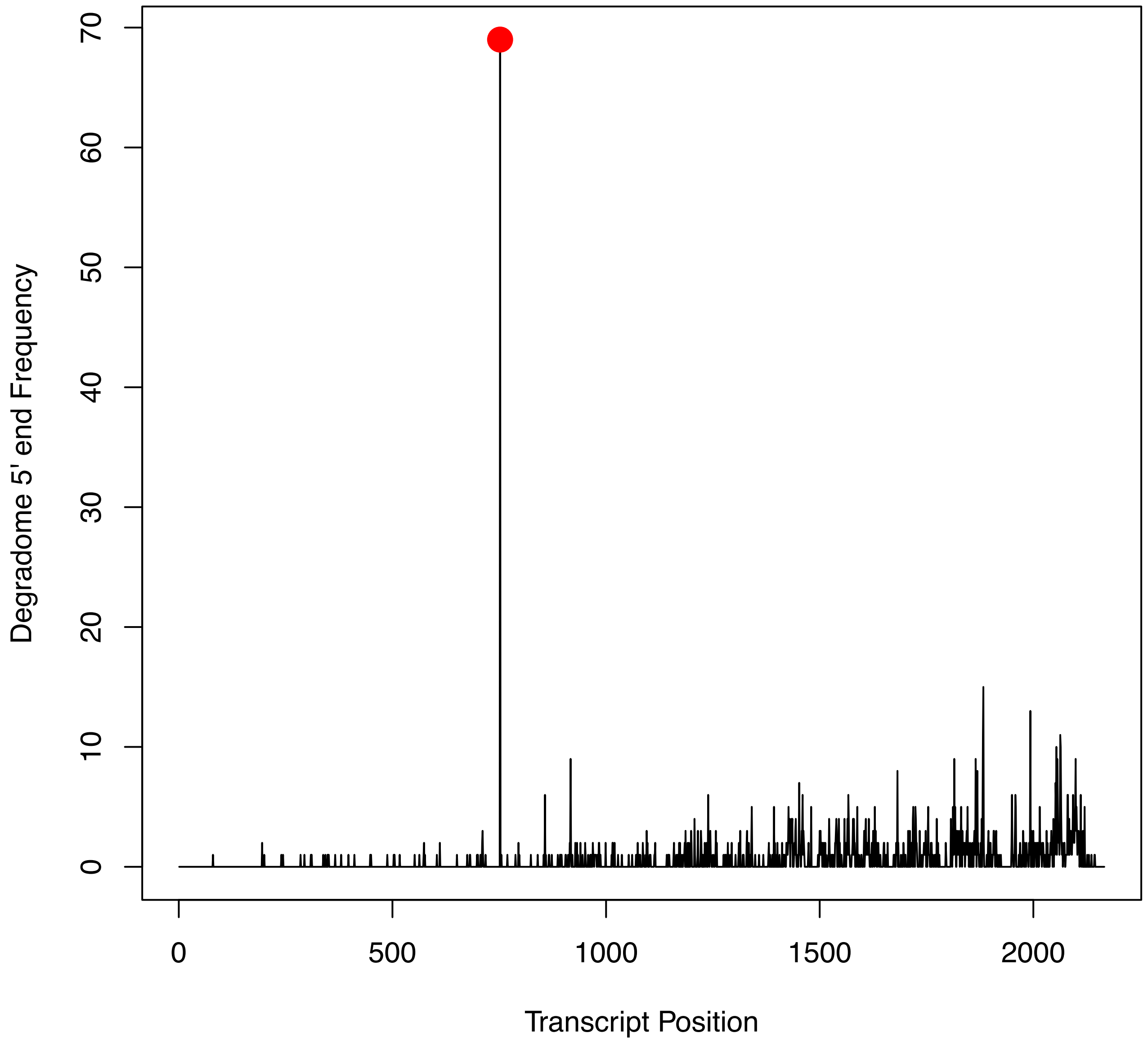

D=Day3
T=HORVU.MOREX.r3.4HG0339430.2
Q=miR396–5p.Cluster_5480
S=752
category=0
p=0.00520331193385881

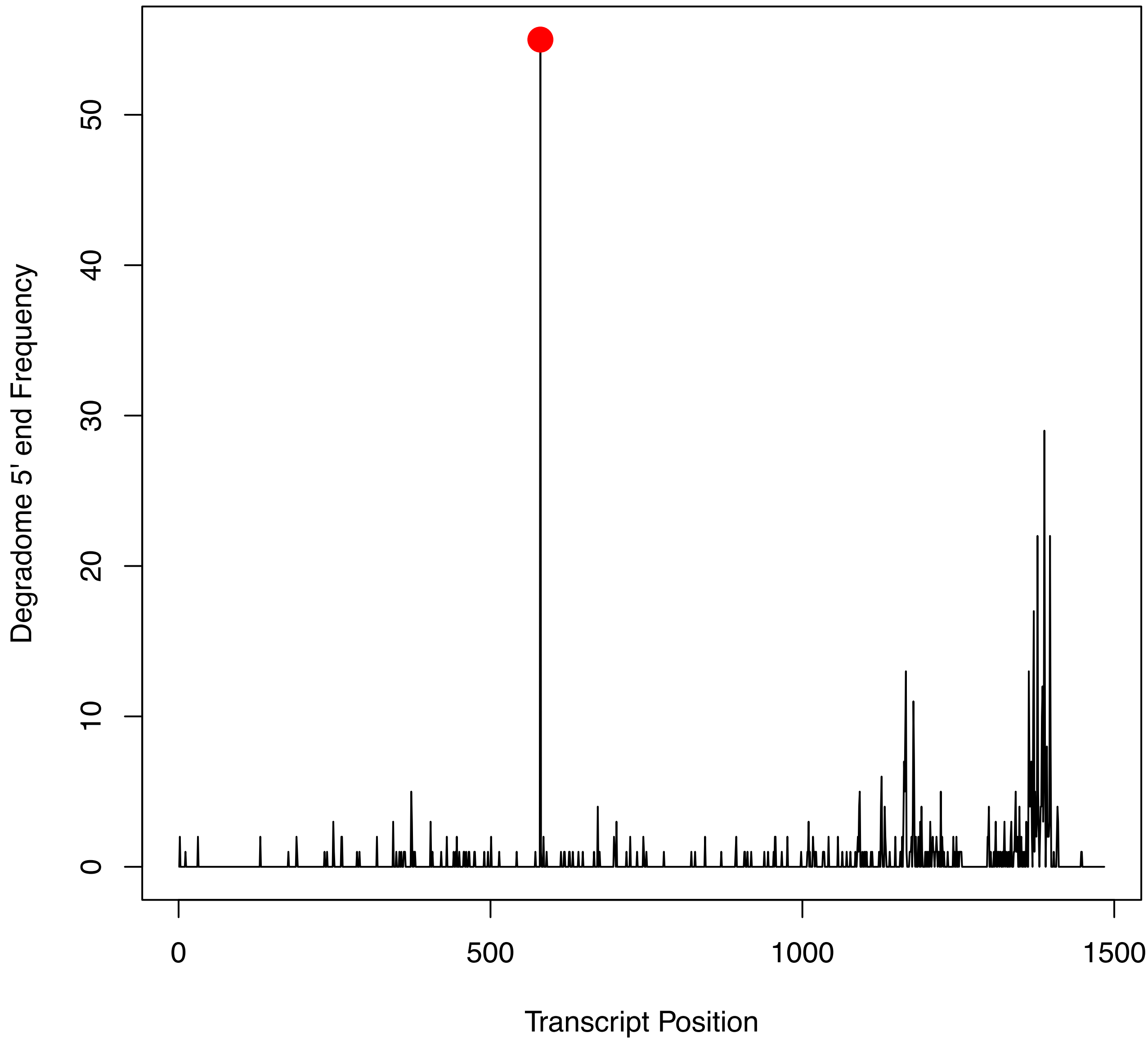

D=Day3
T=HORVU.MOREX.r3.4HG0368070.1
Q=miR396–5p.Cluster_5480
S=580
category=0
p=0.00240490187314257

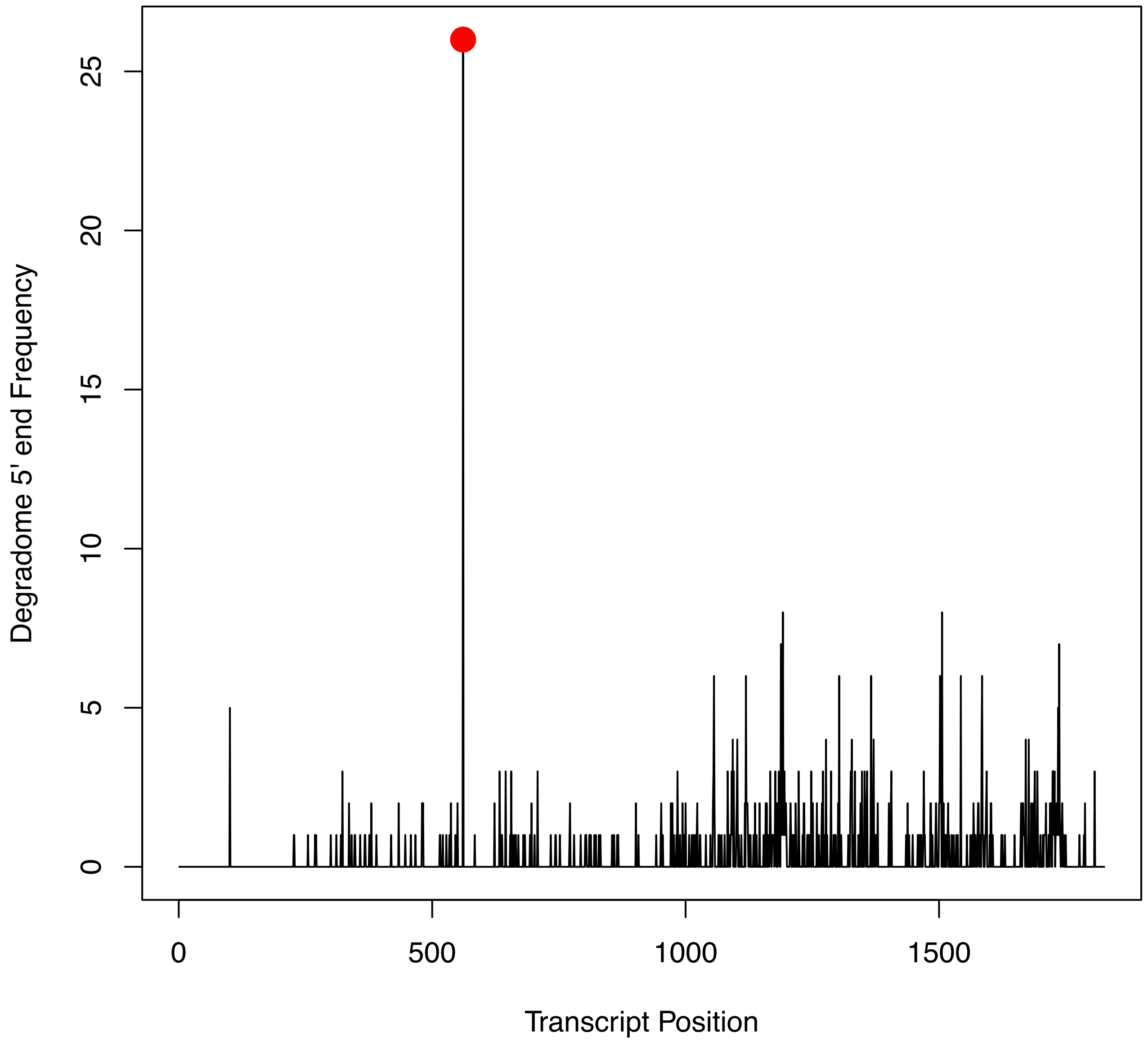

D=Day3
T=HORVU.MOREX.r3.6HG0620090.1
Q=miR396–5p.Cluster_5480
S=561
category=0
p=0.00160391122025372

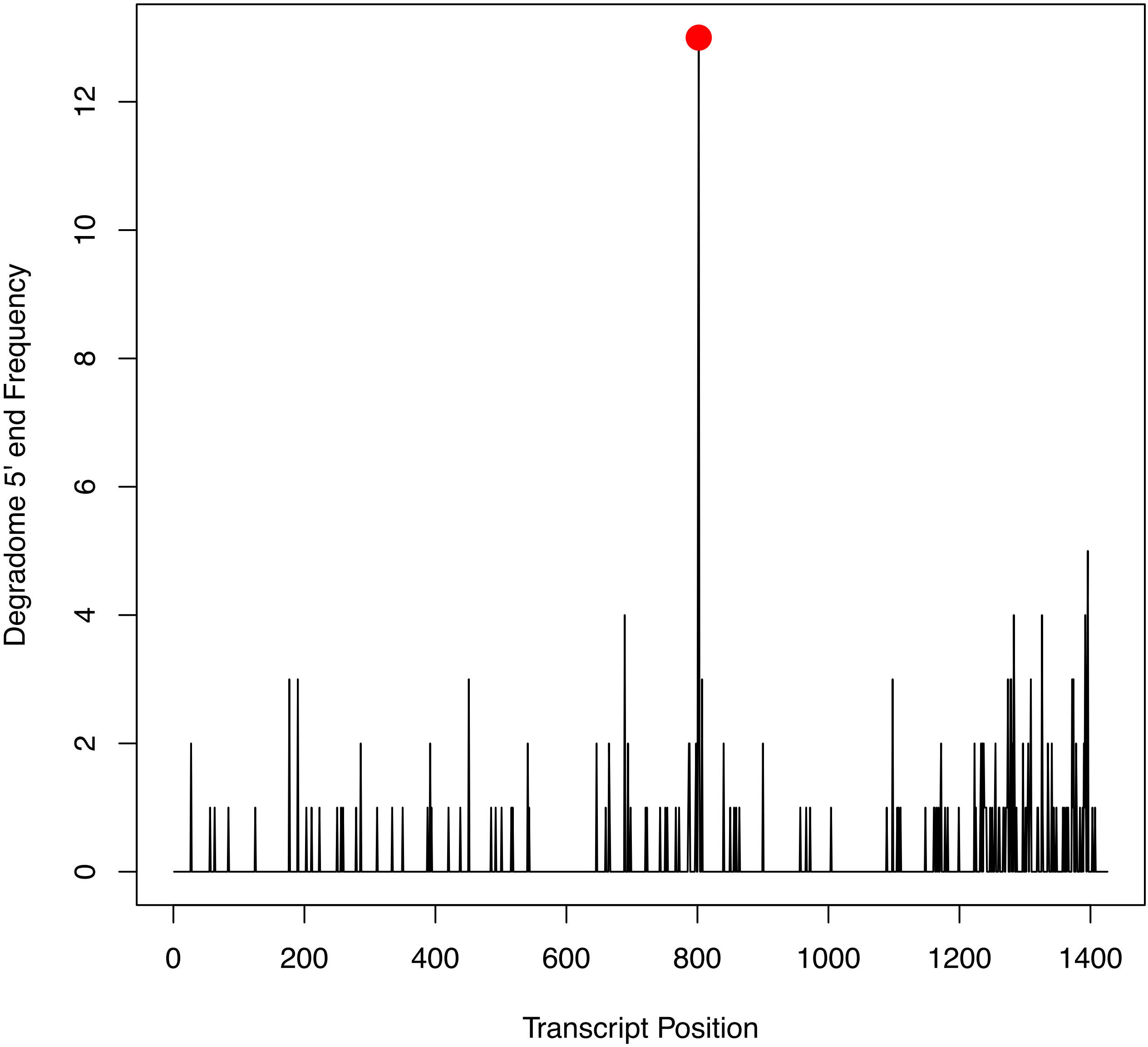

D=Day3
T=HORVU.MOREX.r3.6HG0541280.1
Q=miR9662–3p.Cluster_4660
S=802
category=0
p=0.0327592768534455

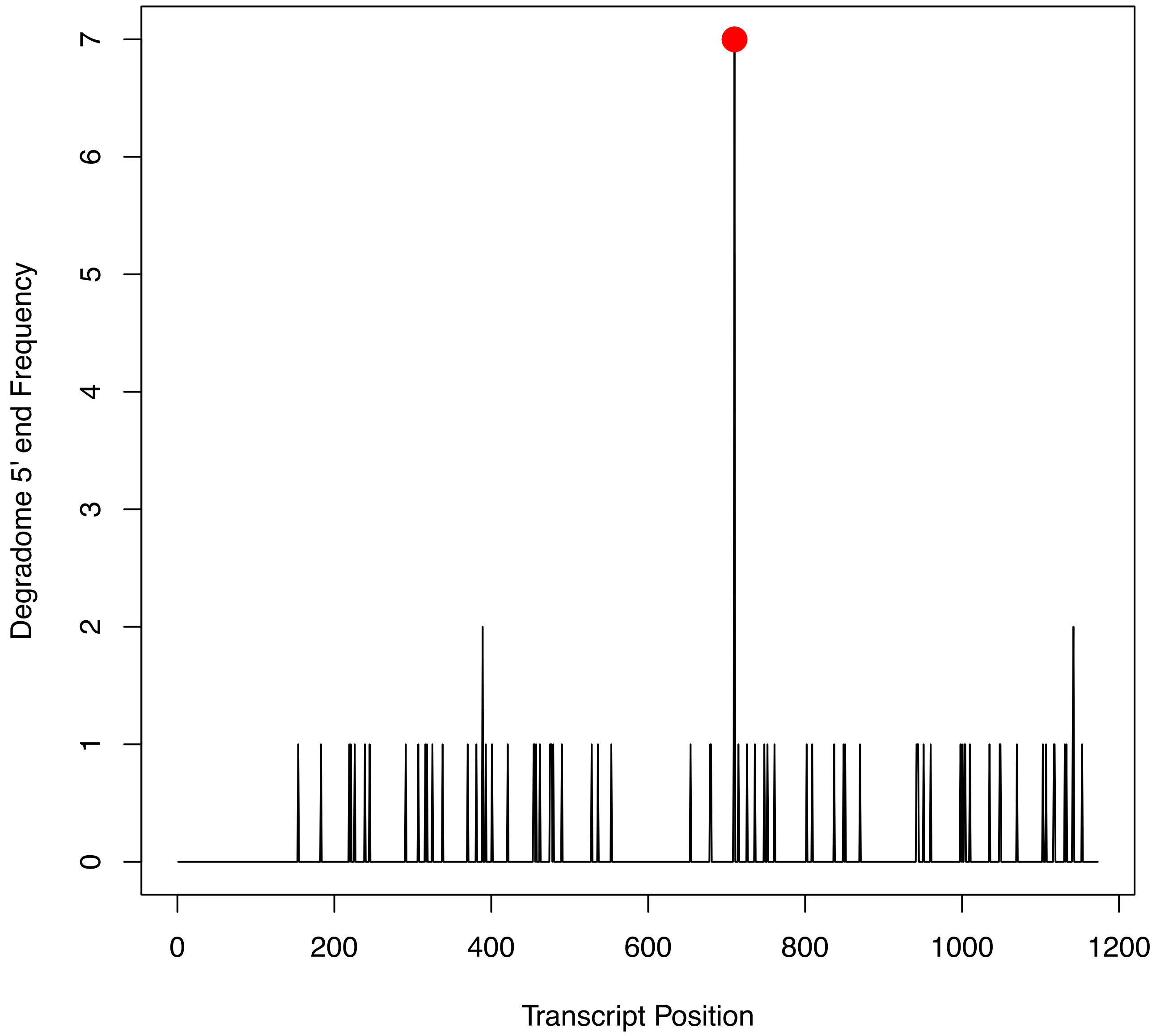

D=Day3
T=HORVU.MOREX.r3.6HG0543320.1
Q=miR9662–3p.Cluster_4660
S=710
category=0
p=0.0171078576313557

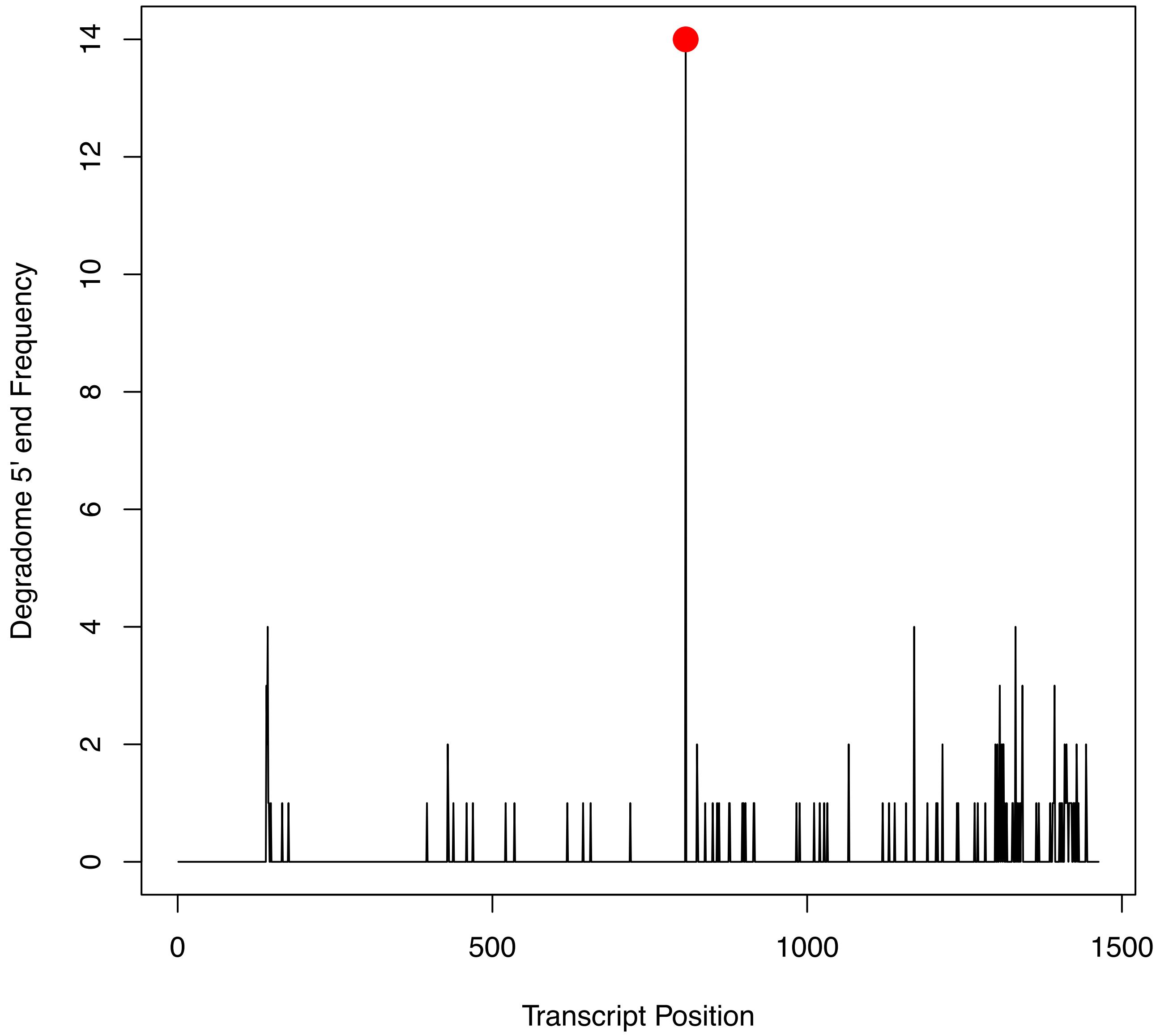

D=Day3
T=HORVU.MOREX.r3.6HG0543350.1
Q=miR9662–3p.Cluster_4660
S=807
category=0
p=0.0167133441402577

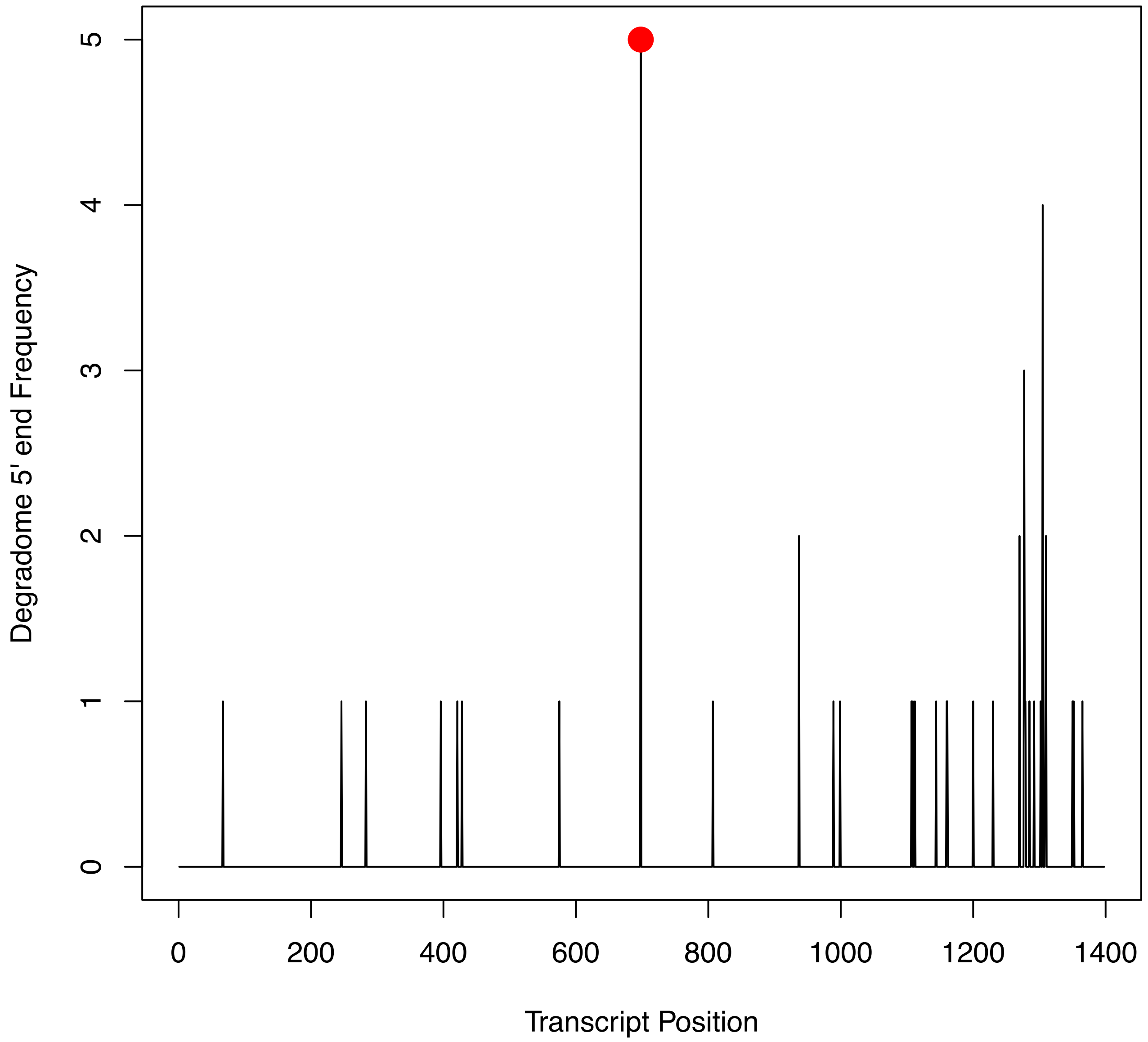

D=Day3
T=HORVU.MOREX.r3.6HG0543370.1
Q=miR9662–3p.Cluster_4660
S=698
category=0
p=0.0182904486403169

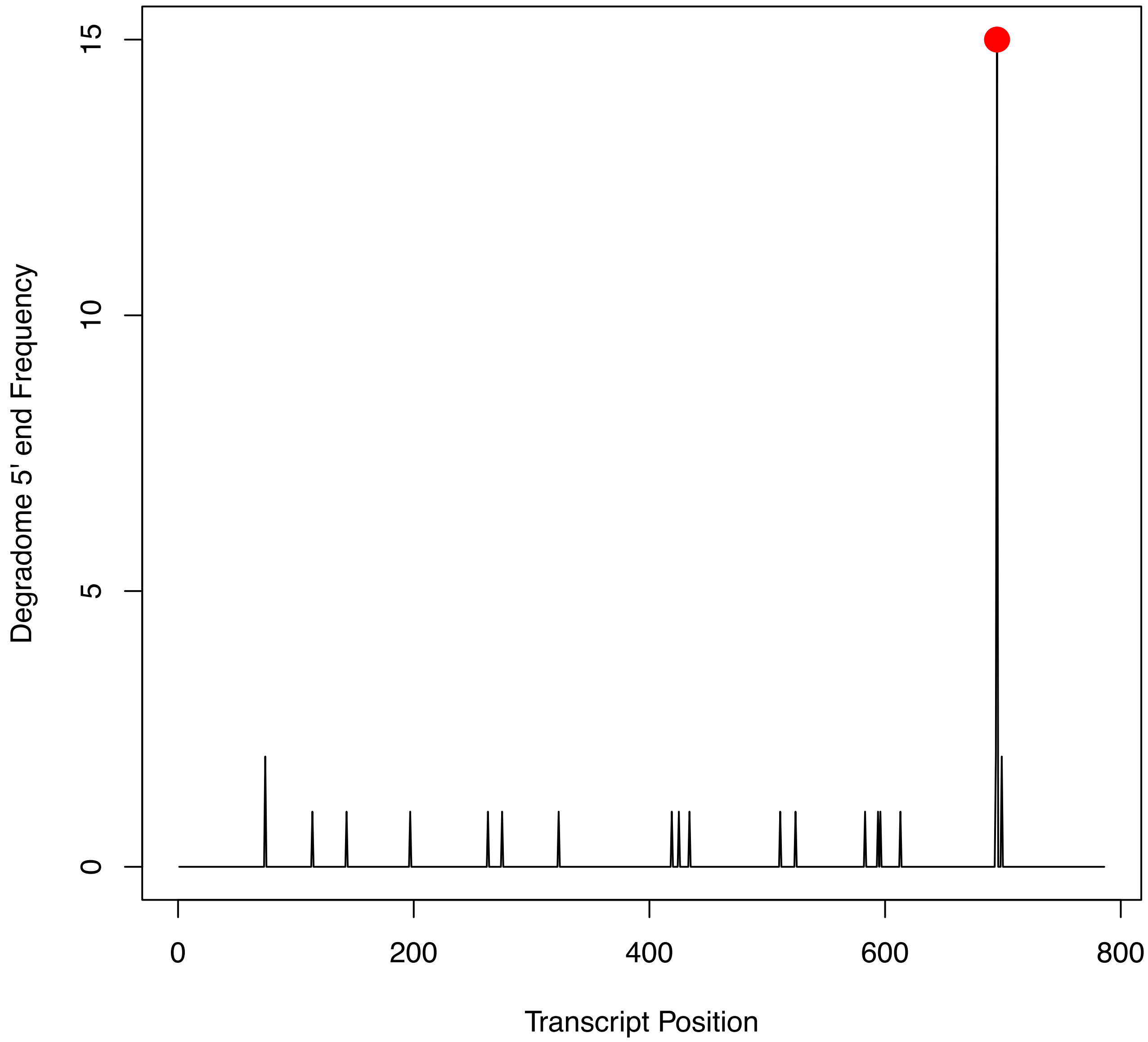

D=Day3
T=HORVU.MOREX.r3.6HG0543460.1
Q=miR9662–3p.Cluster_4660
S=695
category=0
p=0.000802277434667986

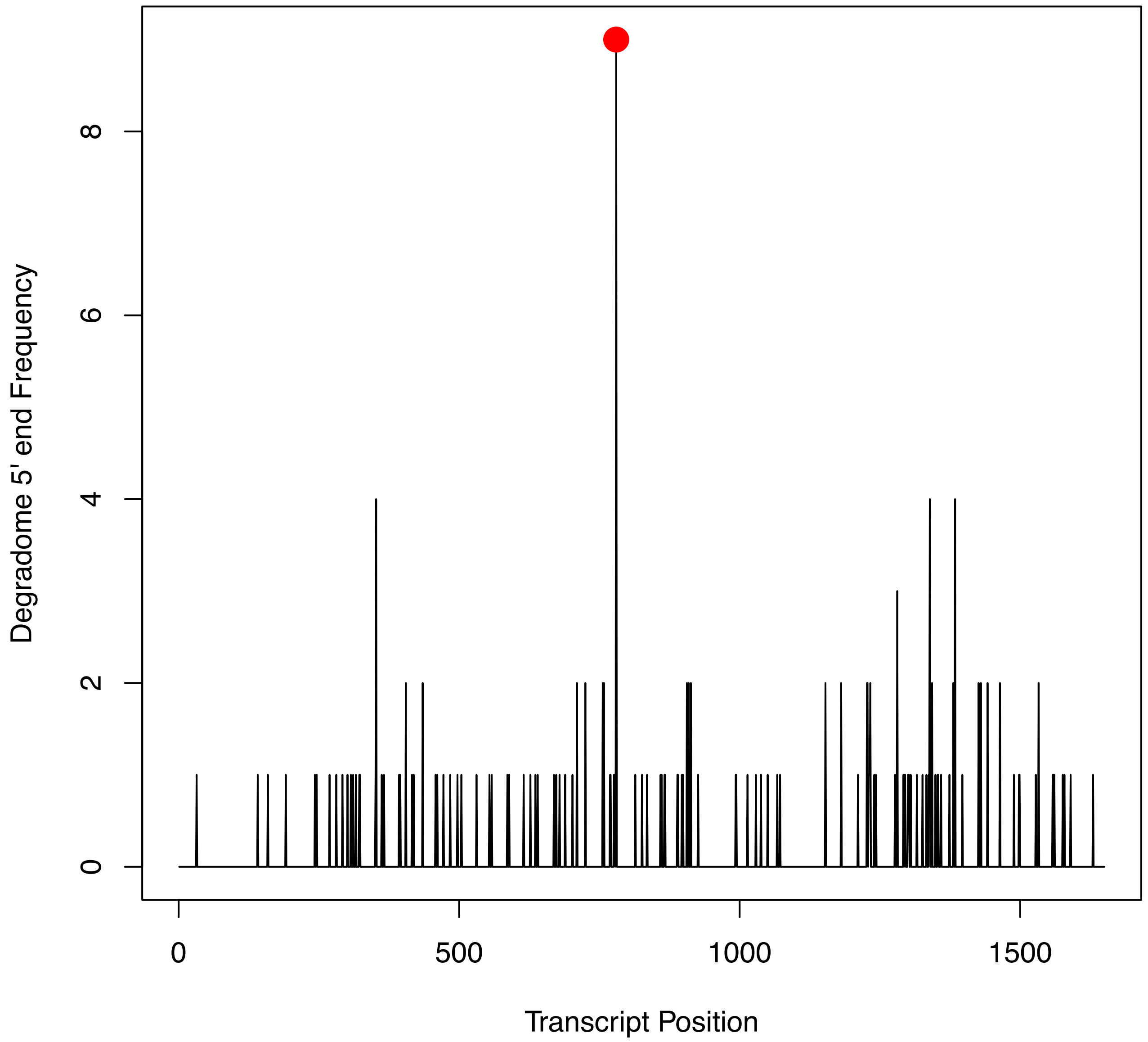

D=Day3
T=HORVU.MOREX.r3.7HG0710980.1
Q=miR9662–3p.Cluster_4660
S=780
category=0
p=0.0155288533131686

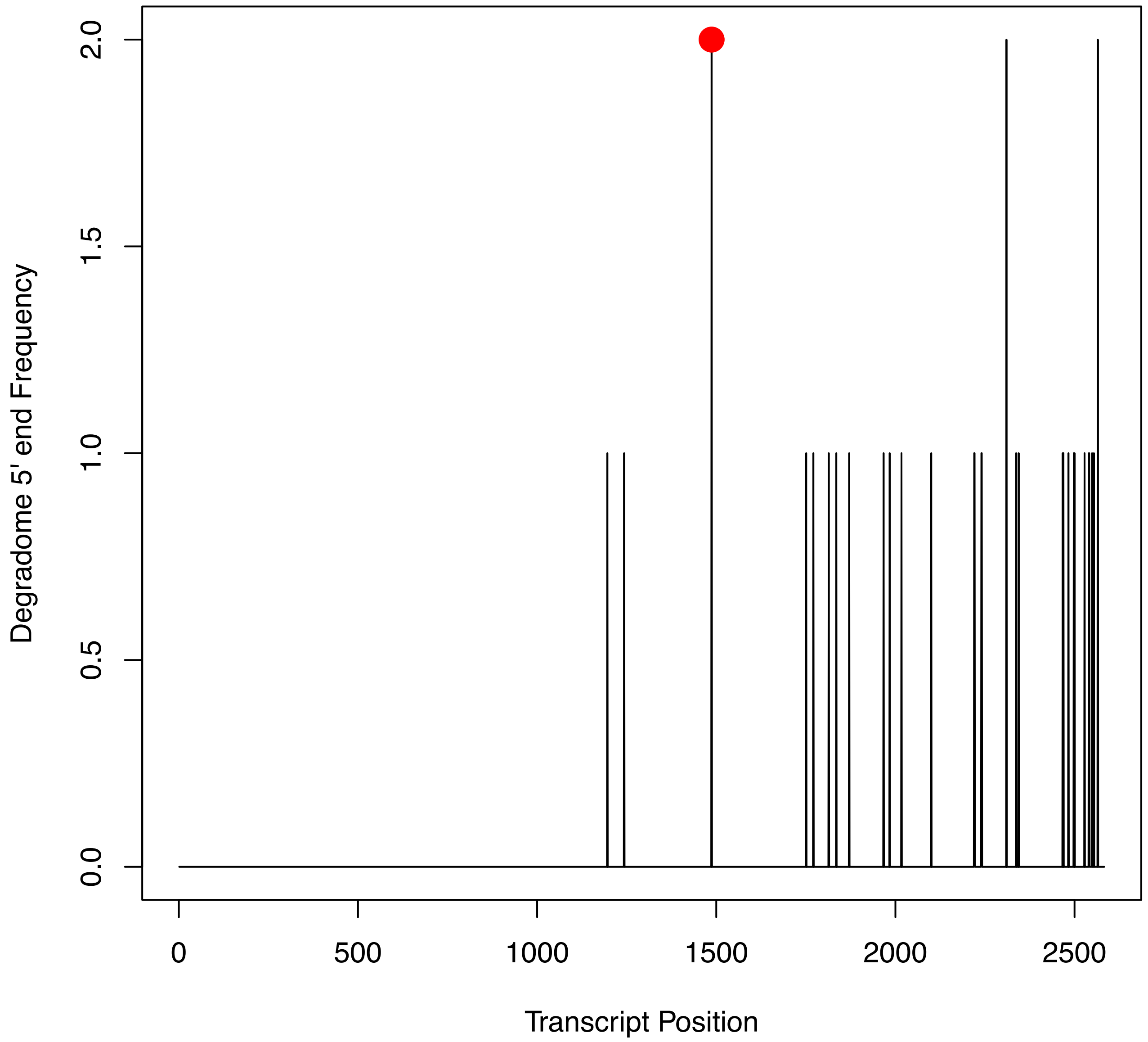

D=Day3
T=HORVU.MOREX.r3.1HG0011590.1
Q=novel–5p.Cluster_2558
S=1487
category=1
p=0.00280510070632434

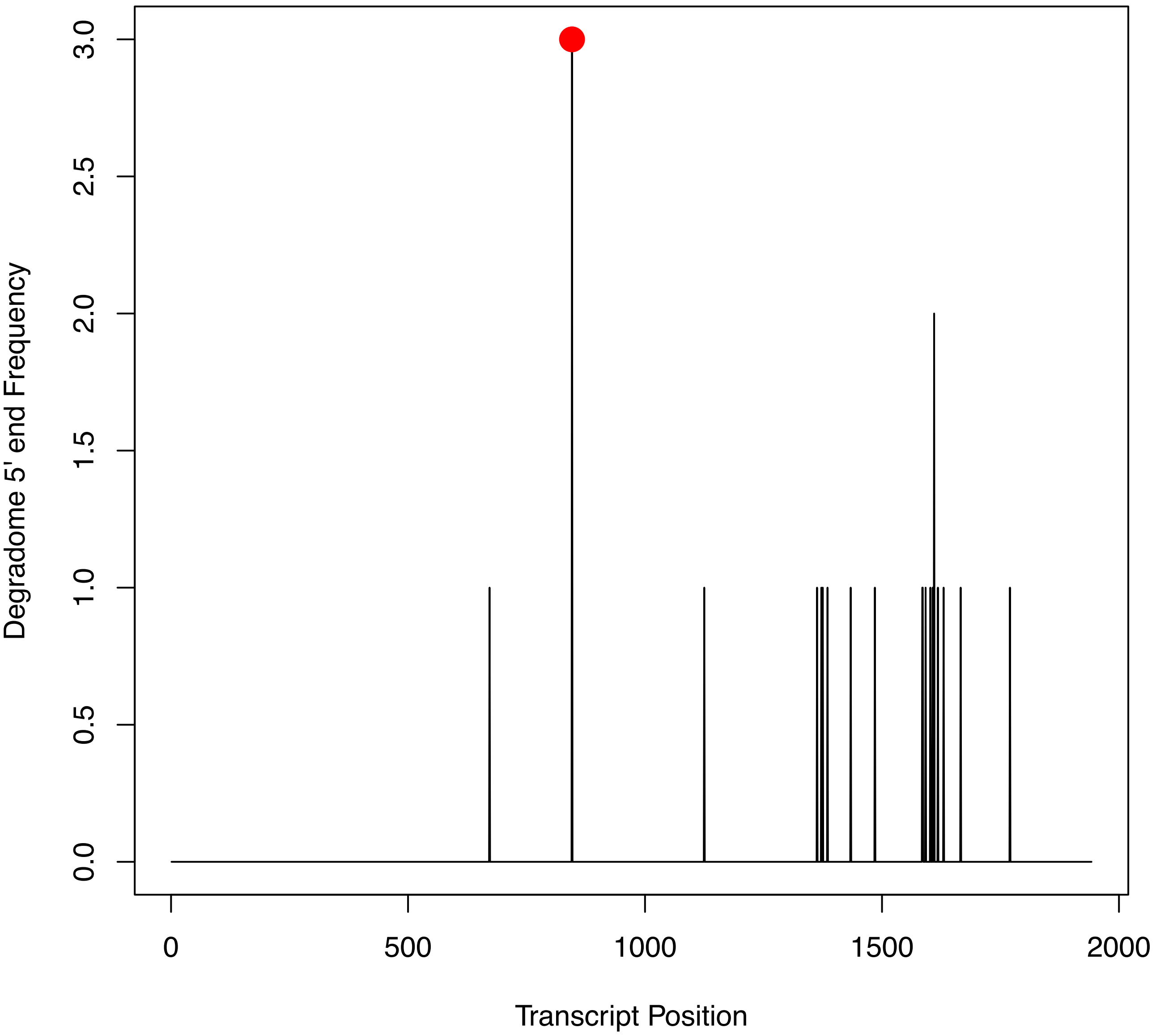

D=Day3
T=HORVU.MOREX.r3.1HG0011700.1
Q=novel–5p.Cluster_2558
S=846
category=0
p=0.00480402019326576

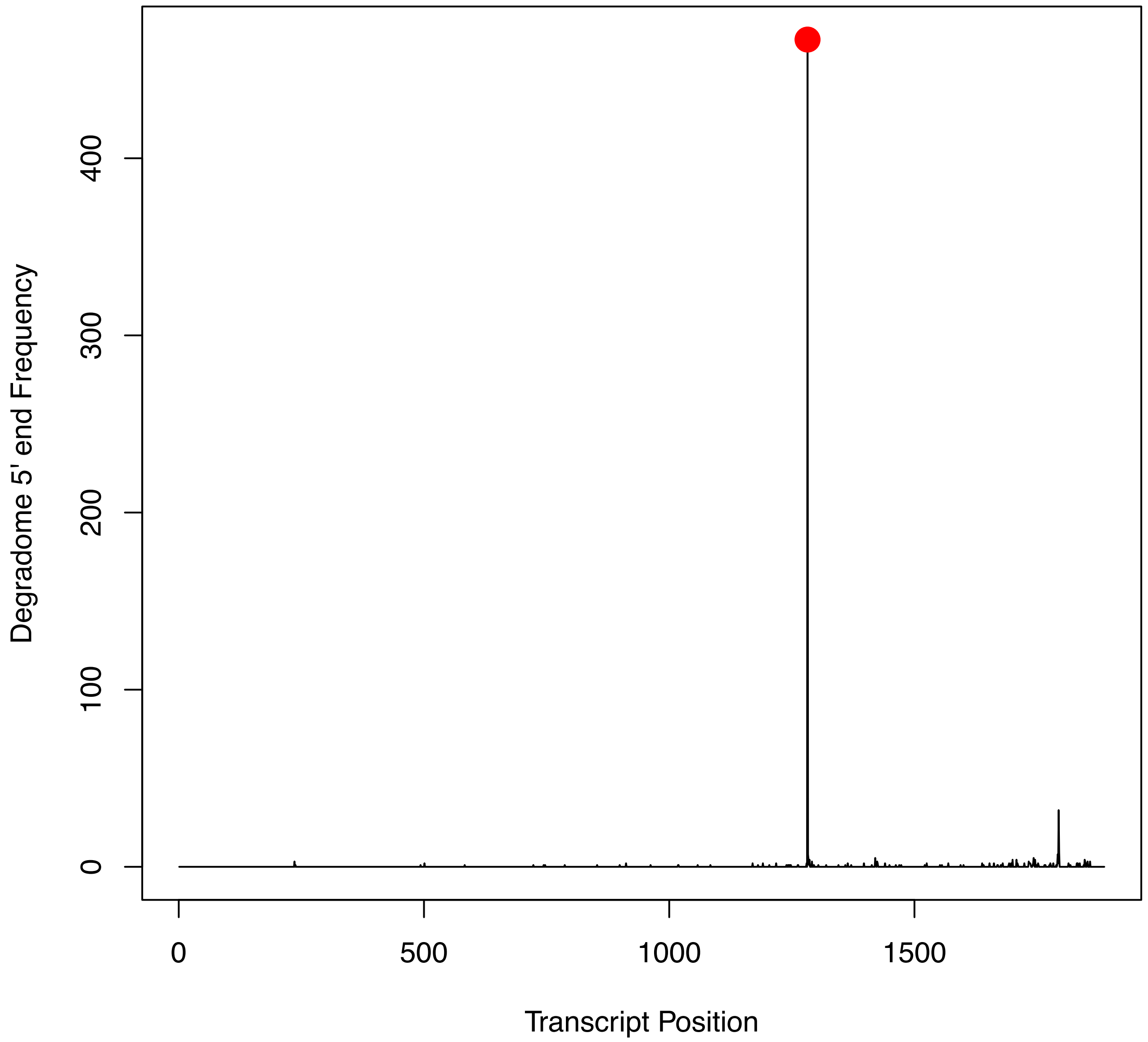

D=Day5
T=HORVU.MOREX.r3.3HG0310780.1
Q=miR156–5p.Cluster_1557.Cluster_1973.Cluster_4963
S=1282
category=0
p=0.00162536221265974

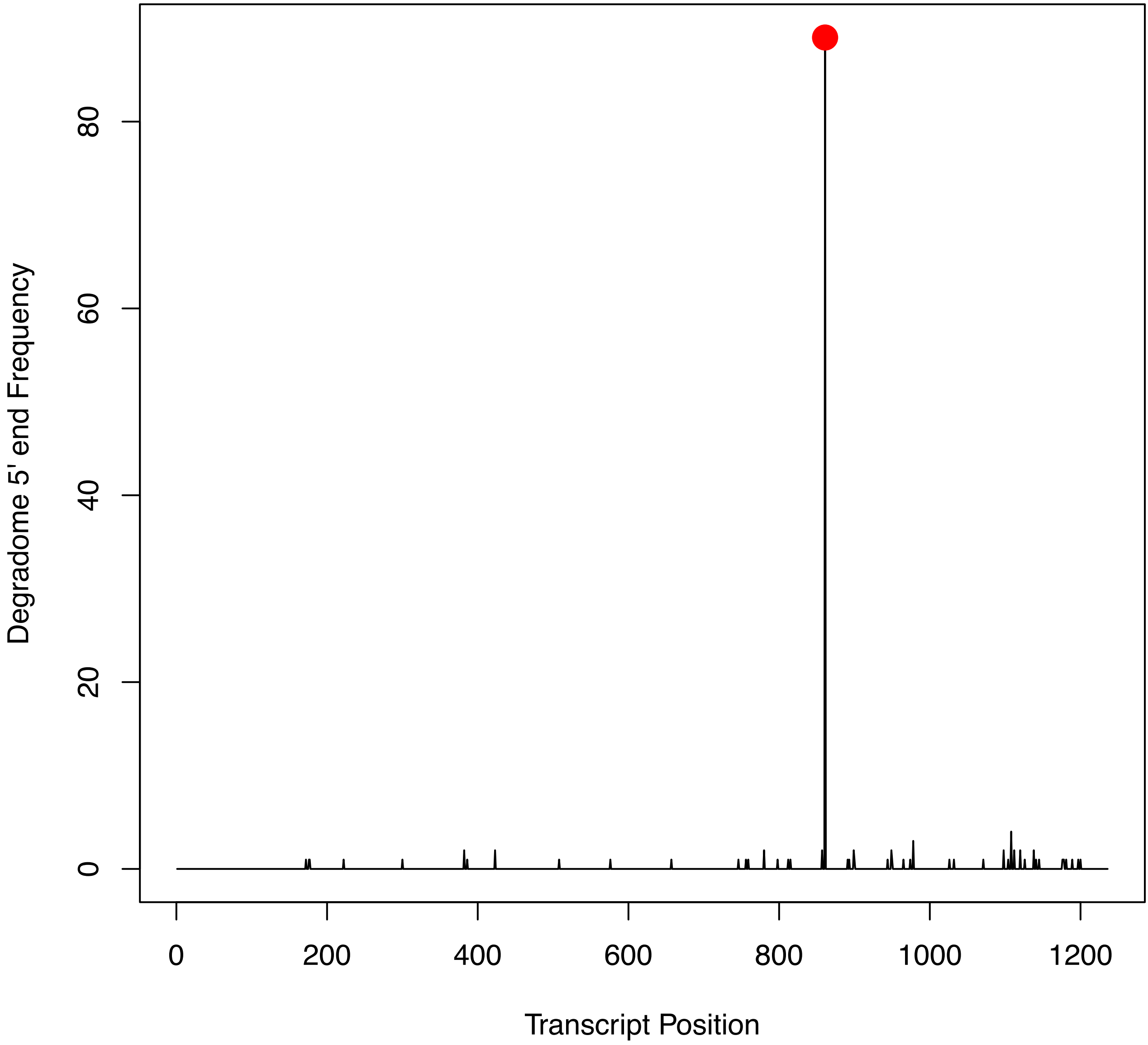

D=Day5
T=HORVU.MOREX.r3.5HG0490900.1
Q=miR156–5p.Cluster_1557.Cluster_1973.Cluster_49
63
S=861
category=0
p=0.00121926949634599

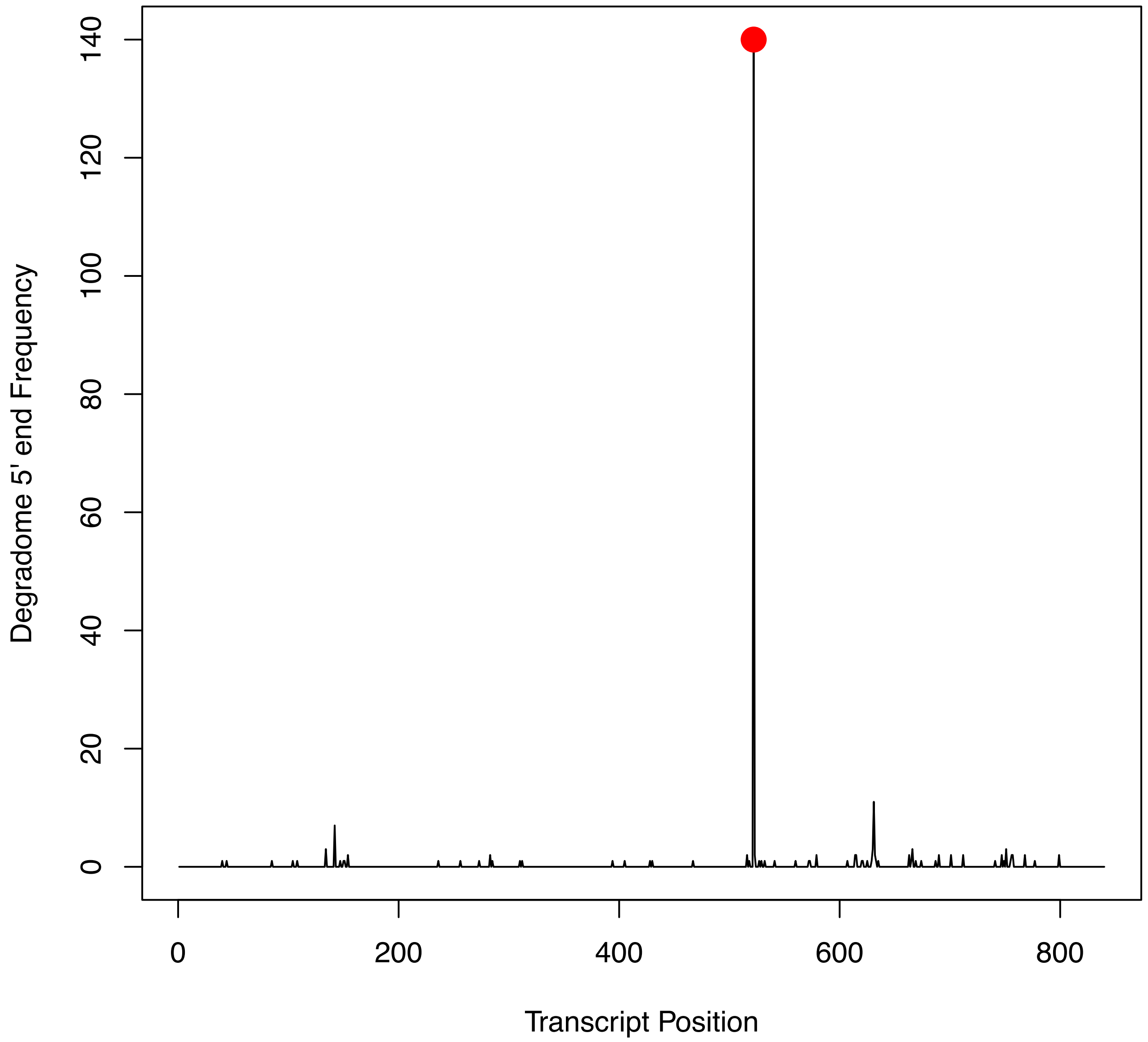

D=Day5
T=HORVU.MOREX.r3.5HG0494180.1
Q=miR156–5p.Cluster_1557.Cluster_1973.Cluster_4963
S=522
category=0
p=0.00284264995443928

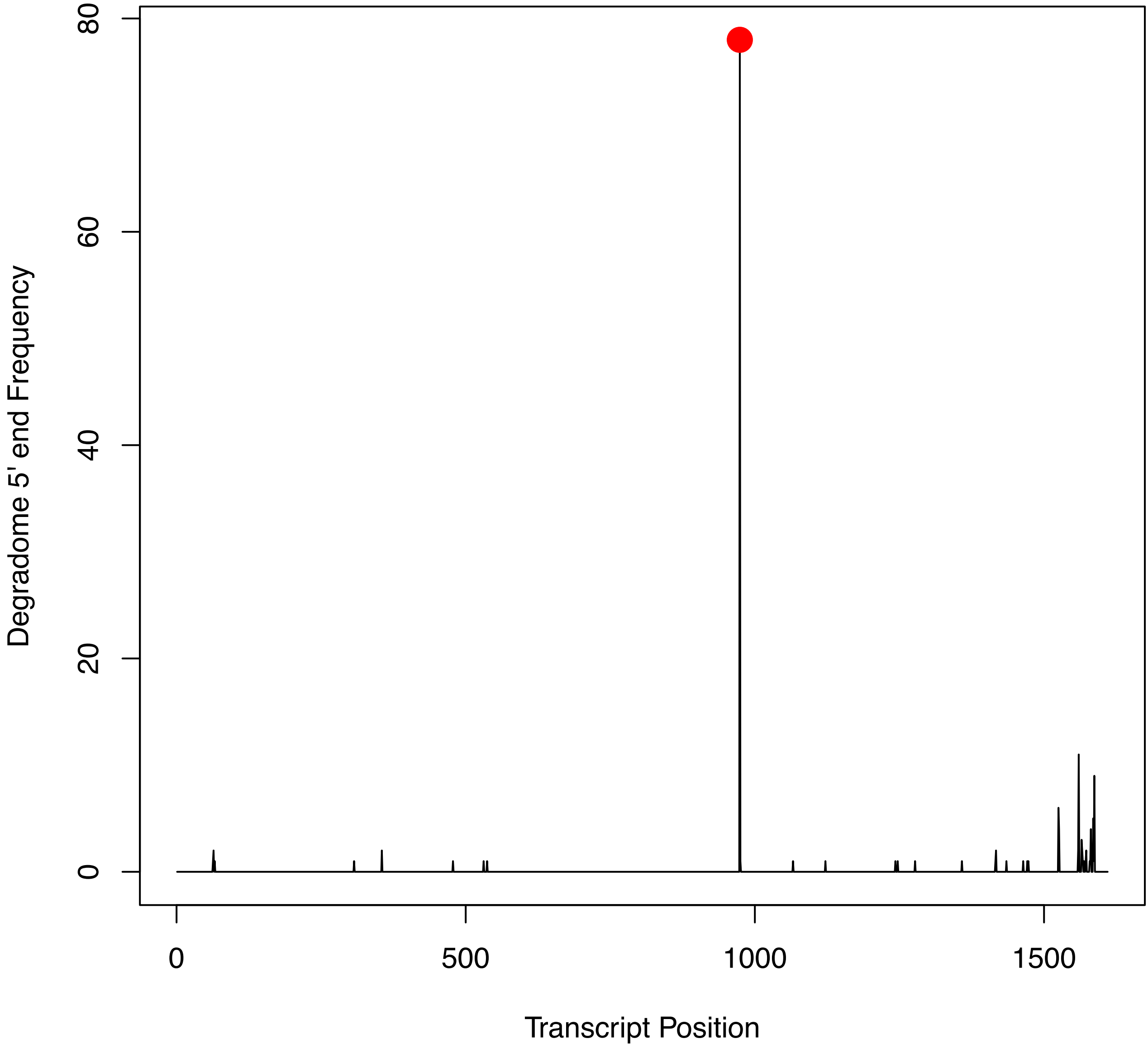

D=Day5
T=HORVU.MOREX.r3.7HG0679980.1
Q=miR156–5p.Cluster_1557.Cluster_1973.Cluster_4963
S=974
category=0
p=0.00243705237458713

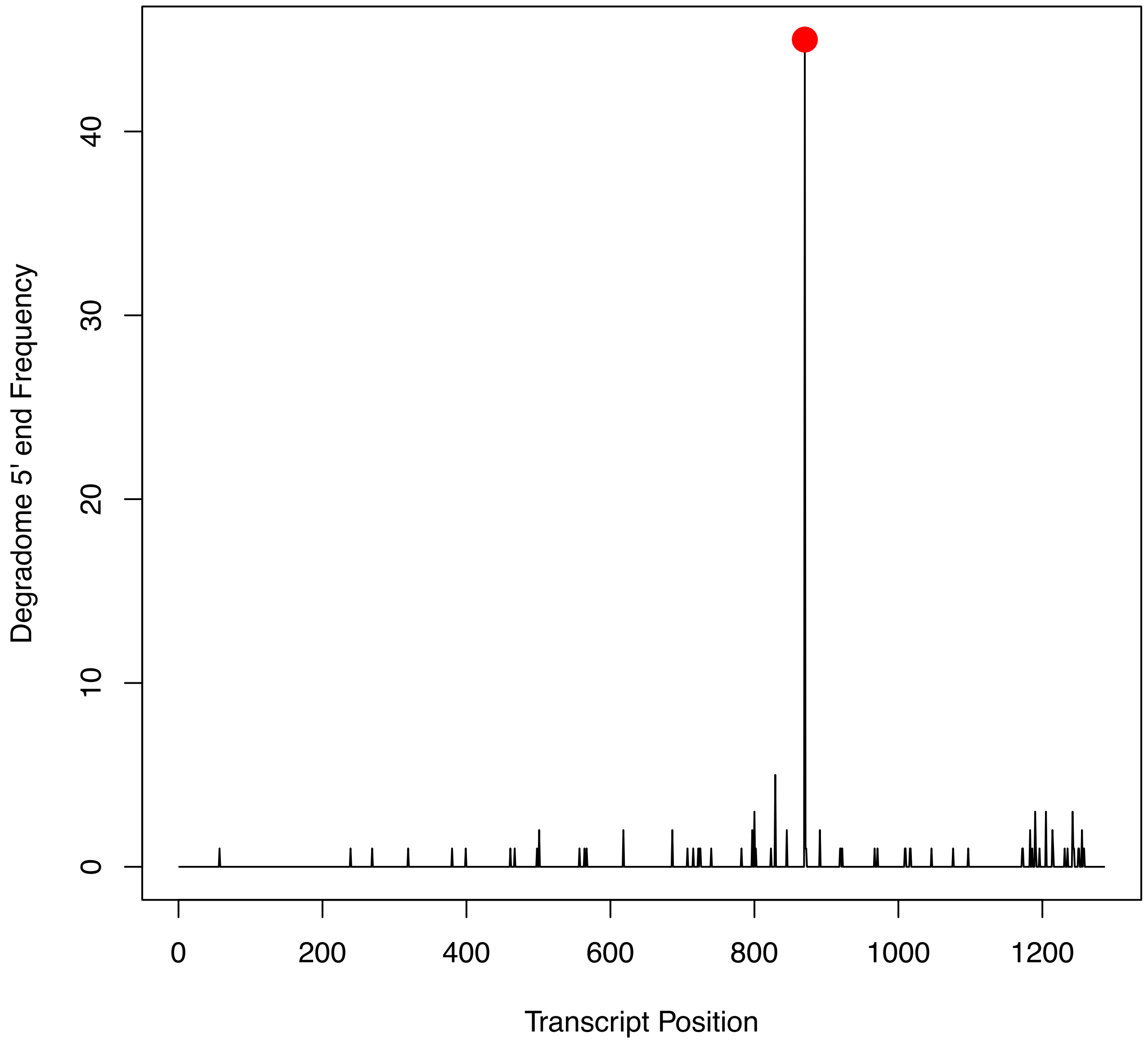

D=Day5
T=HORVU.MOREX.r3.7HG0684000.1
Q=miR156–5p.Cluster_1557.Cluster_1973.Cluster_4963
S=870
category=0
p=0.00203128981636258

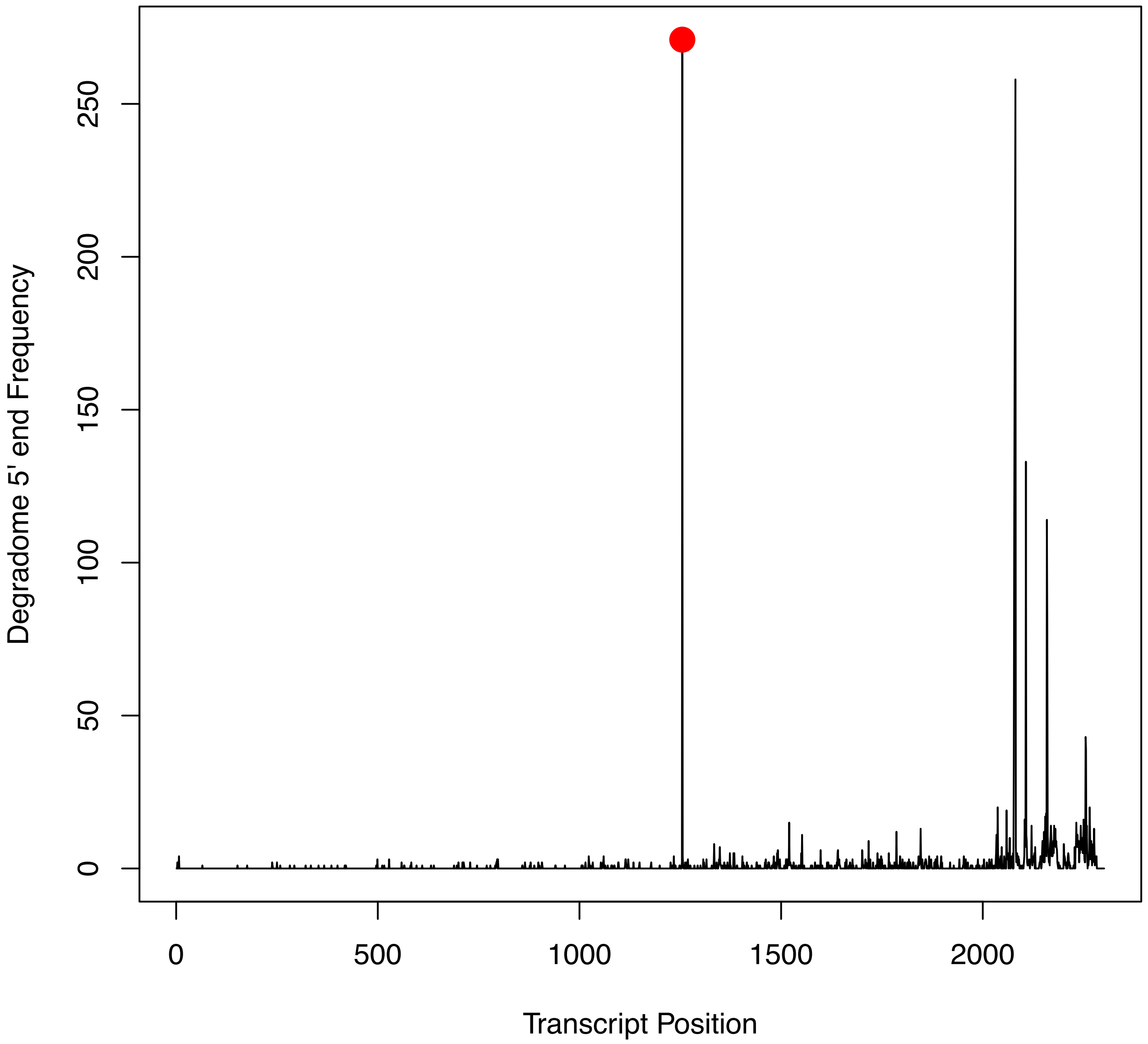

D=Day5
T=HORVU.MOREX.r3.3HG0296070.1
Q=miR159–3p.Cluster_1875
S=1255
category=0
p=0.00121926949634599

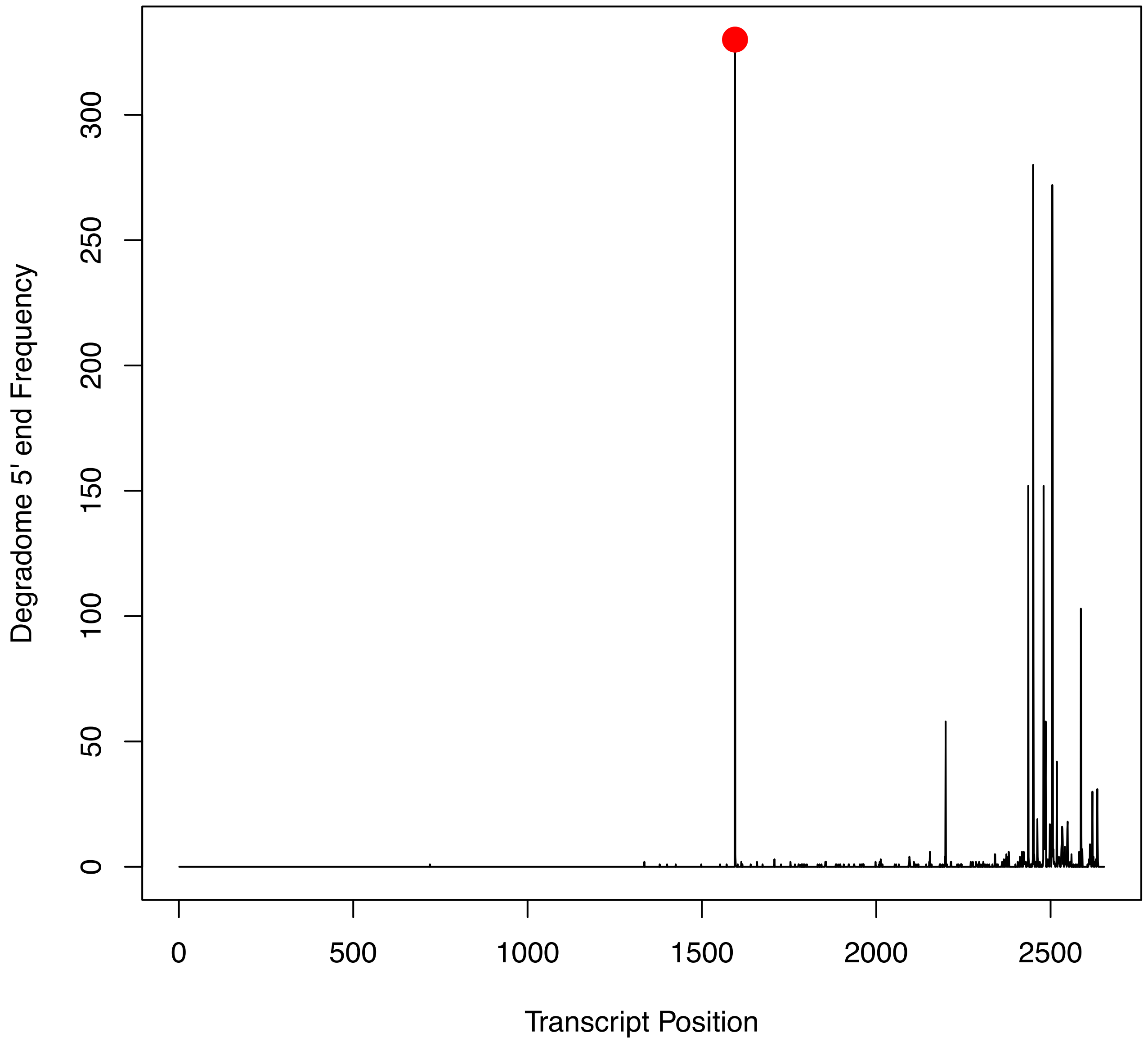

D=Day5
T=HORVU.MOREX.r3.1HG0043670.1
Q=miR160–5p.Cluster_6224
S=1595
category=0
p=0.000813011600260927

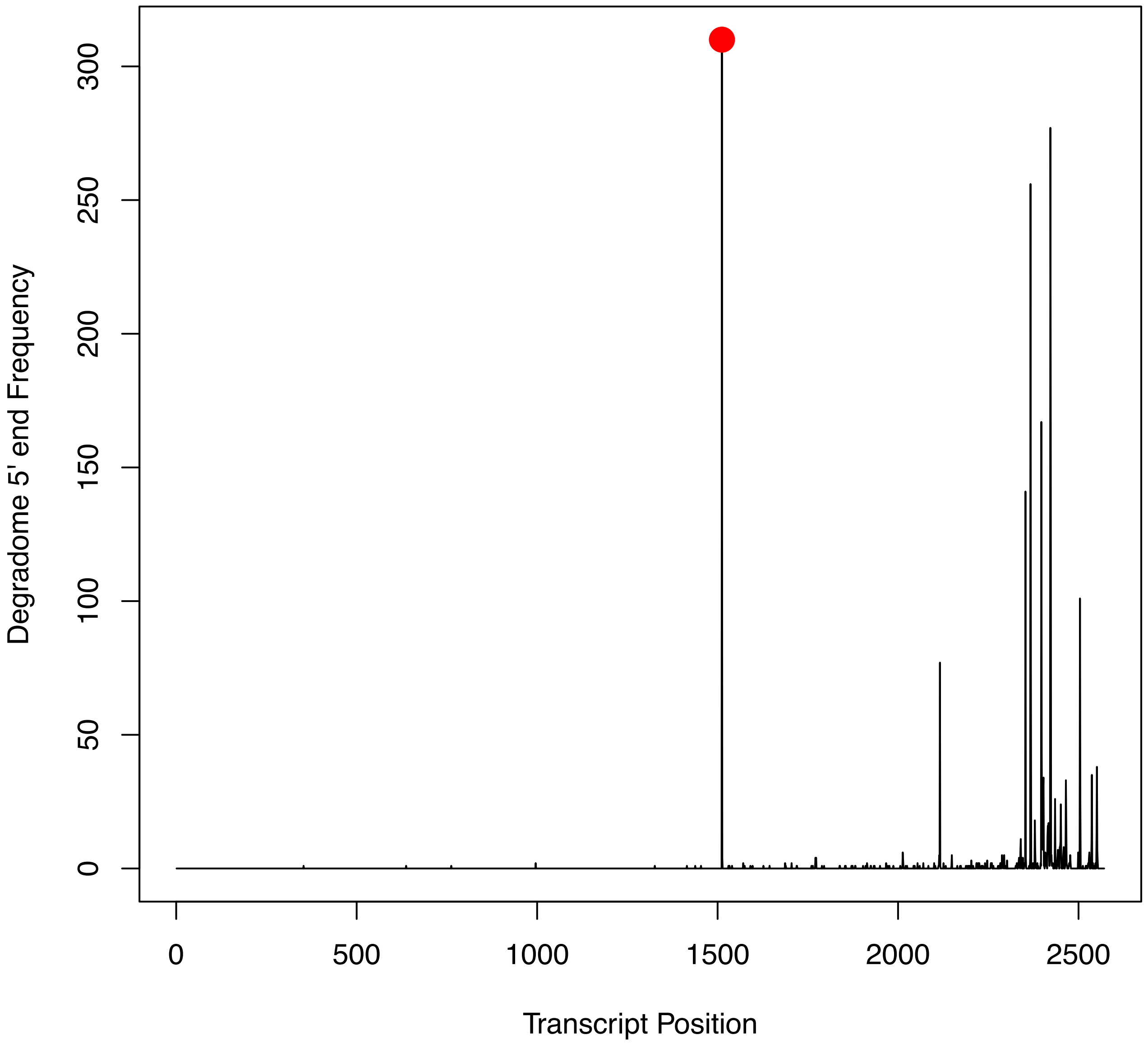

D=Day5
T=HORVU.MOREX.r3.1HG0043670.2
Q=miR160–5p.Cluster_6224
S=1512
category=0
p=0.000406588457217305

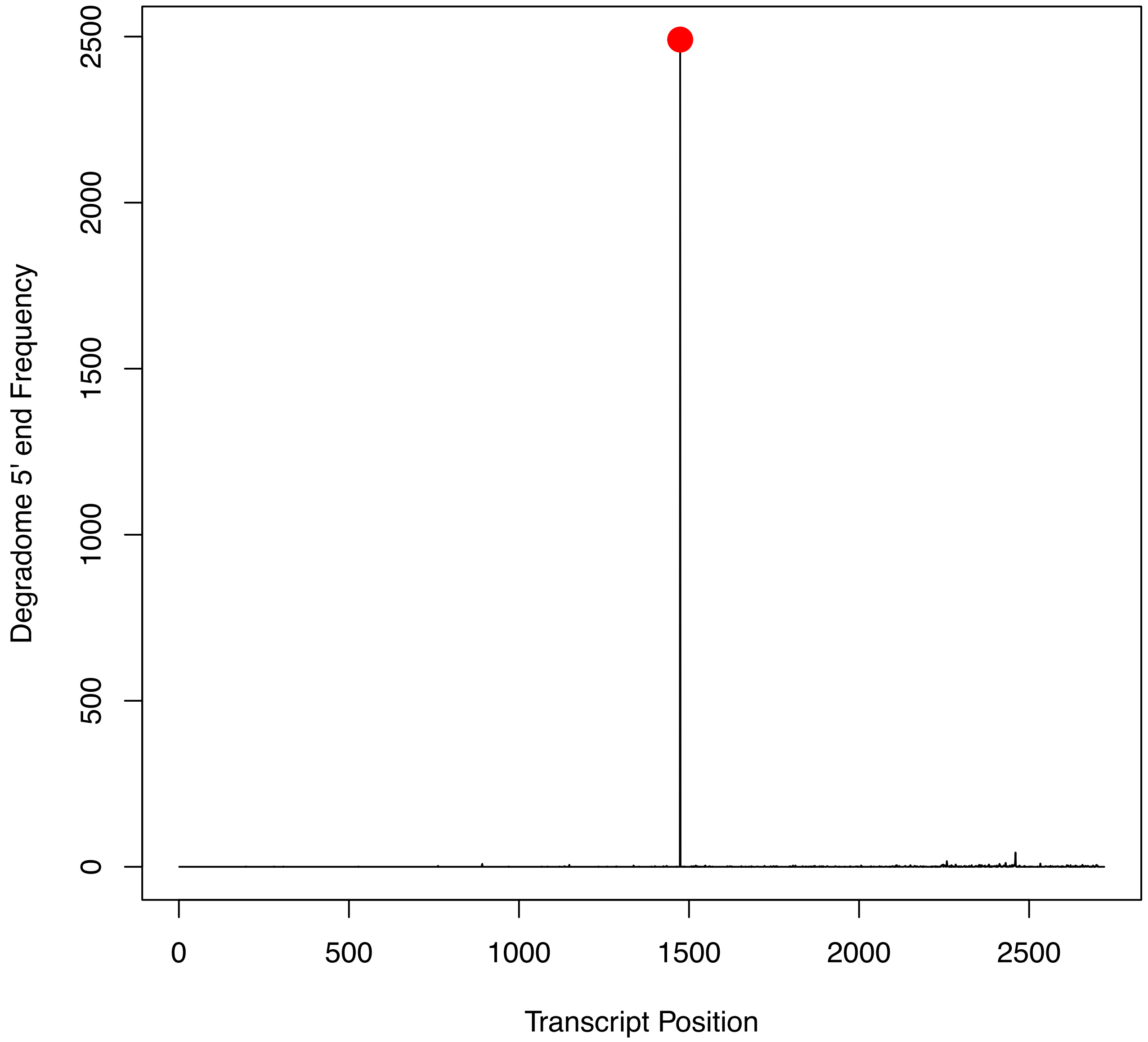

D=Day5
T=HORVU.MOREX.r3.2HG0182280.1
Q=miR160–5p.Cluster_6224
S=1474
category=0
p=0.00243705237458713

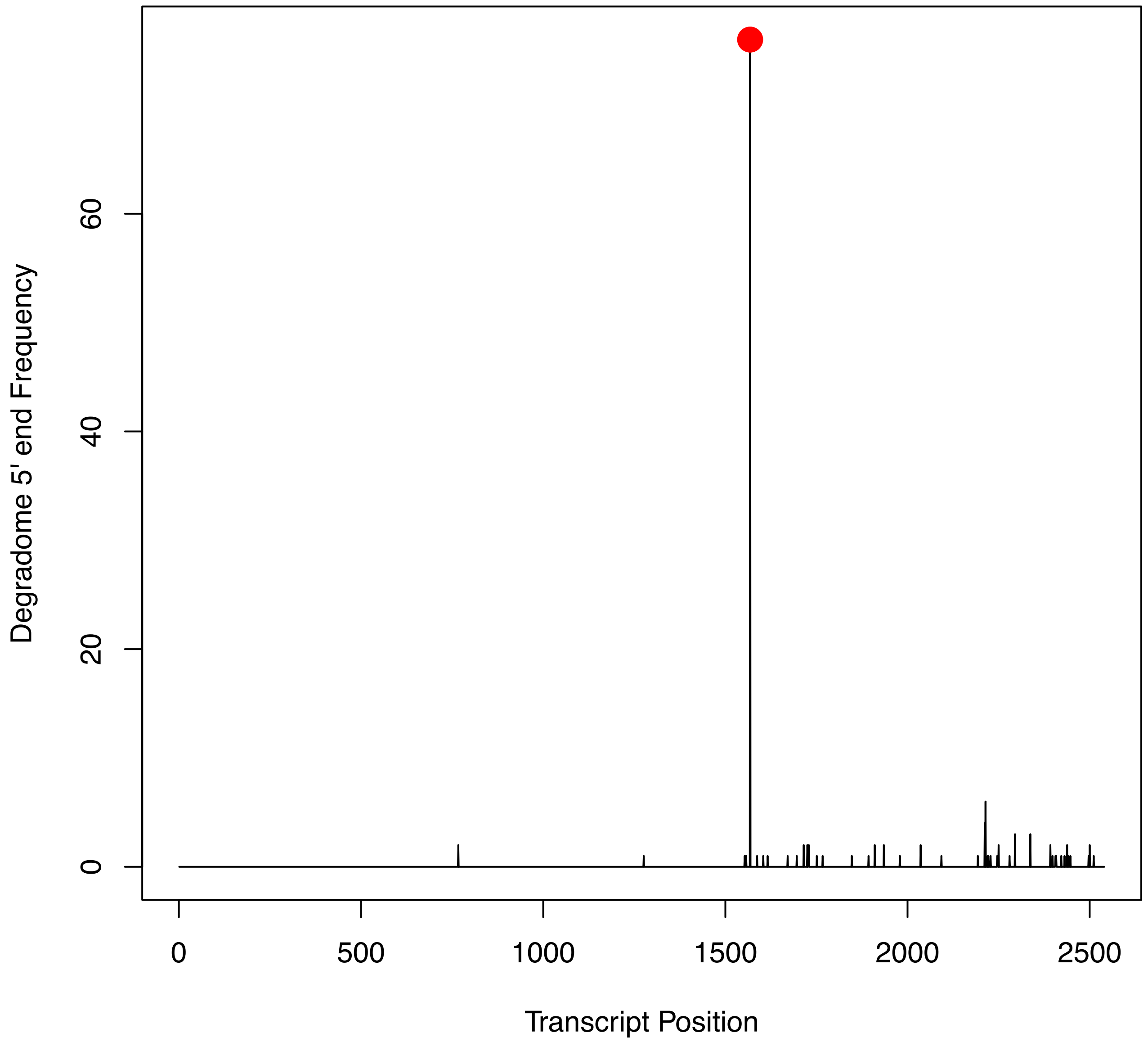

D=Day5
T=HORVU.MOREX.r3.6HG0596670.1
Q=miR160–5p.Cluster_6224
S=1568
category=0
p=0.00203128981636258

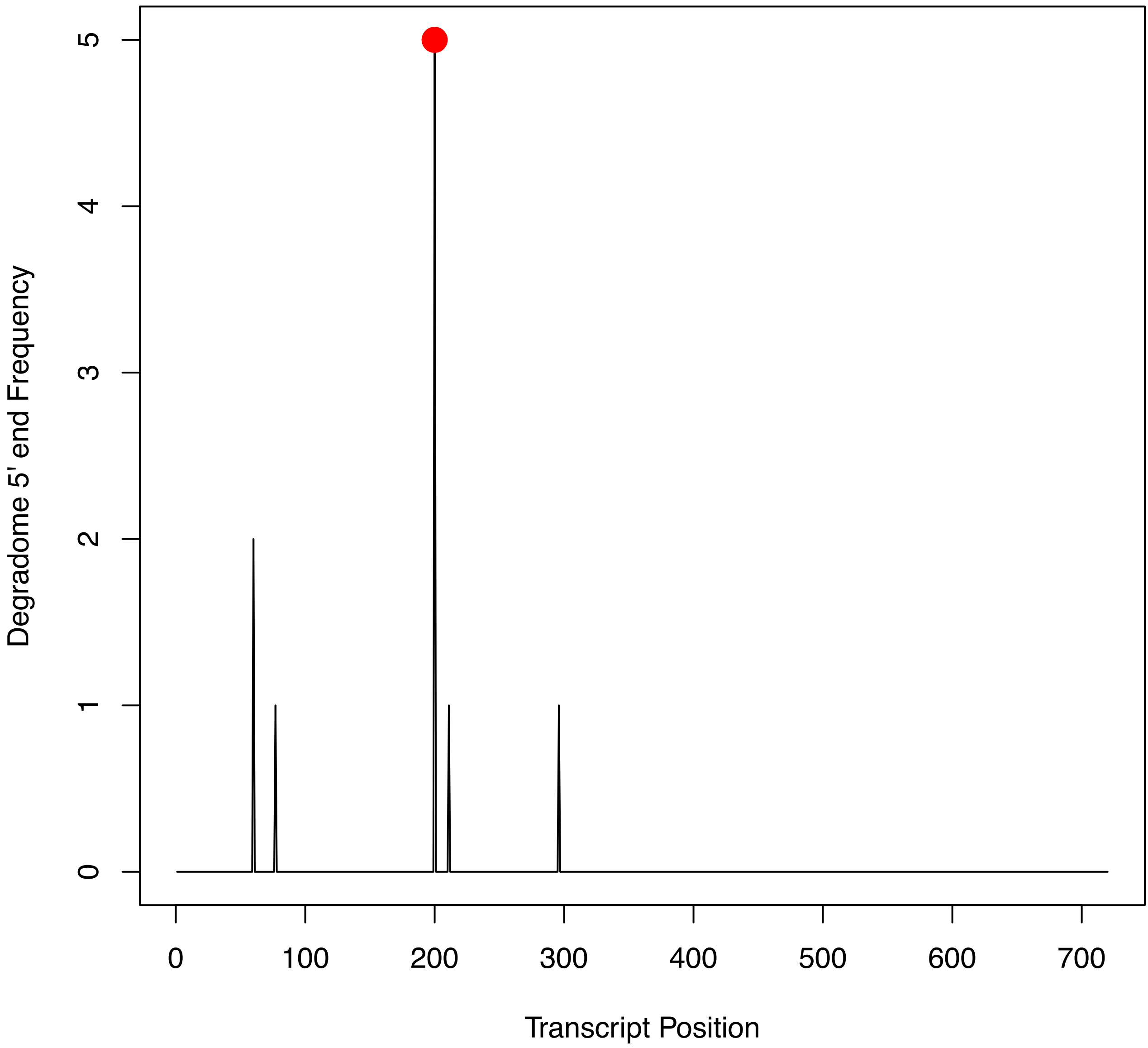

D=Day5
T=HORVU.MOREX.r3.2HG0139920.1
Q=miR166–3p.Cluster_426.Cluster_3396
S=200
category=0
p=0.00243705237458713

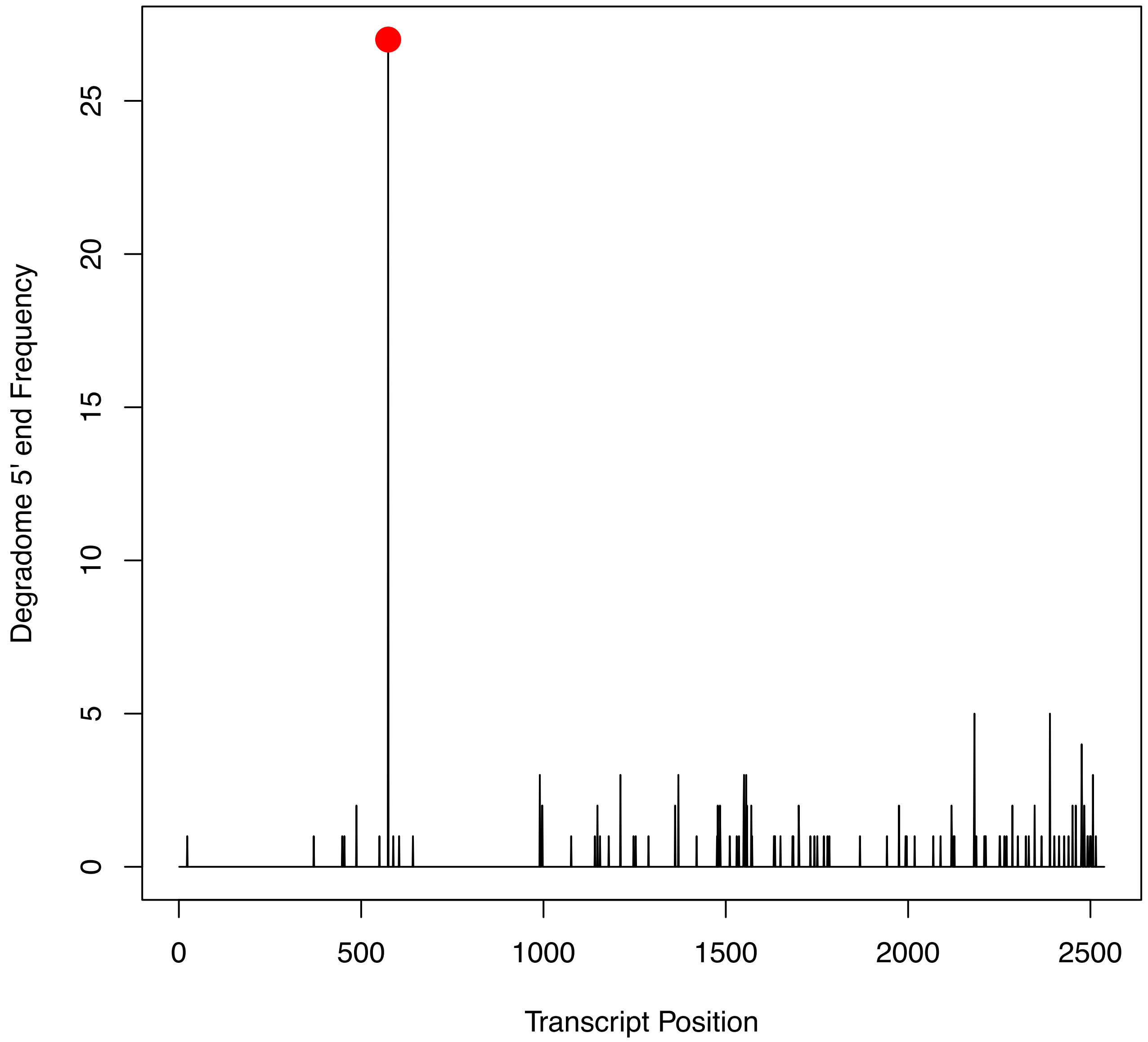

D=Day5
T=HORVU.MOREX.r3.3HG0244080.1
Q=miR166–3p.Cluster_4051
S=574
category=0
p=0.000813011600260927

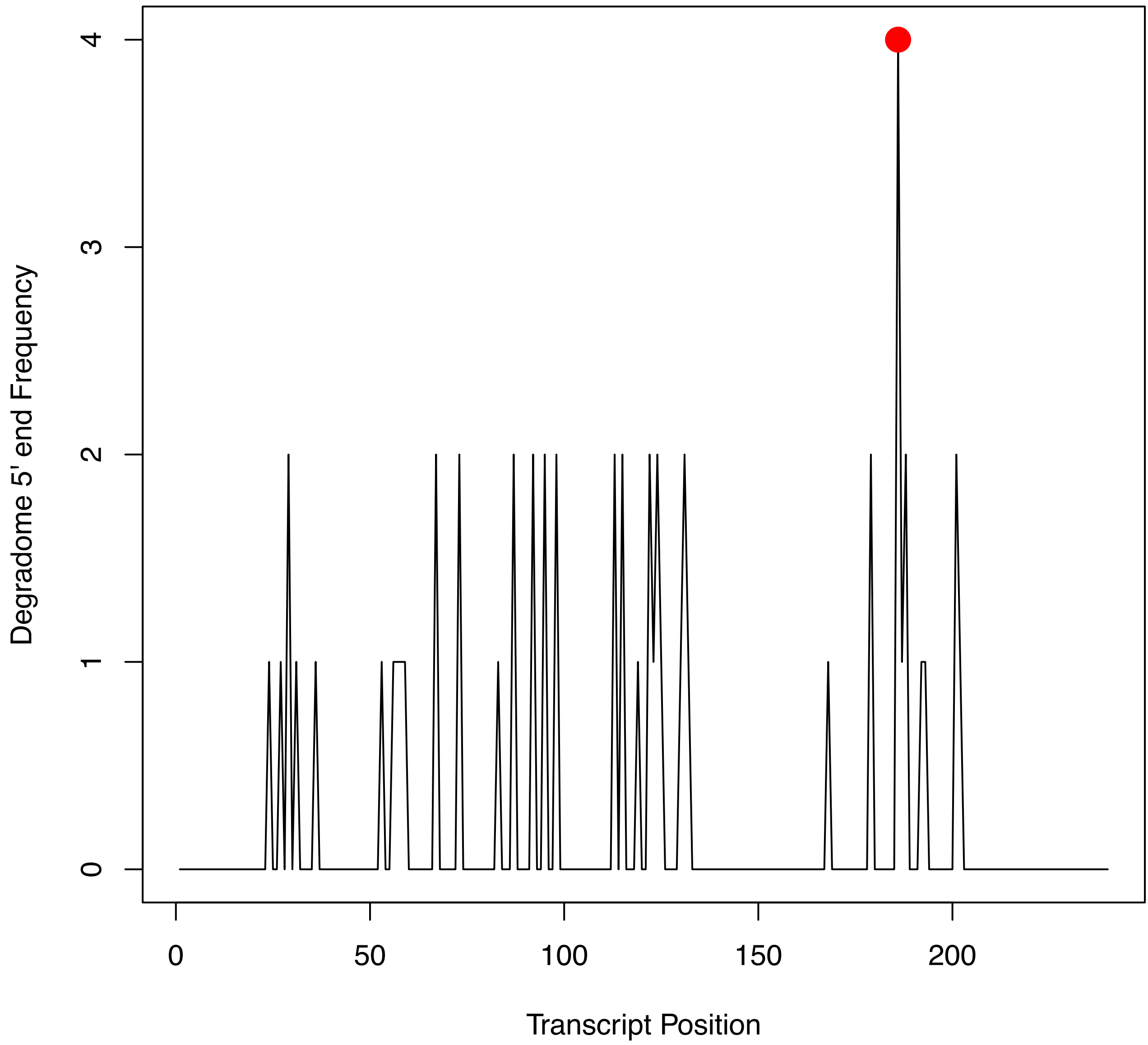

D=Day5
T=HORVU.MOREX.r3.7HG0649520.1
Q=miR166–5p.Cluster_3396
S=186
category=0
p=0.0280656126924335

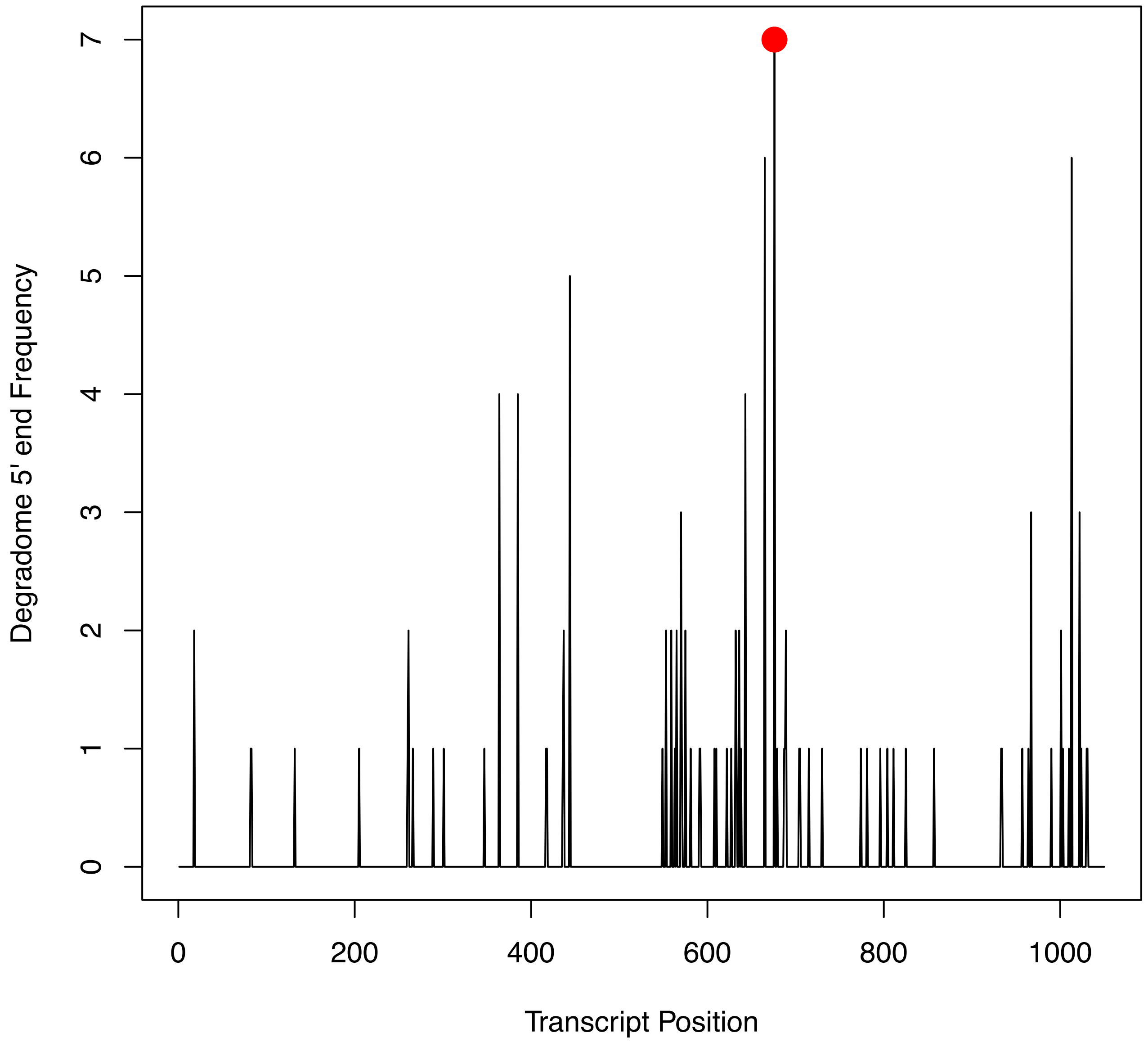

D=Day5
T=HORVU.MOREX.r3.7HG0698540.1
Q=miR167–5p.Cluster_3392.Cluster_3623
S=676
category=0
p=0.0105177471319823

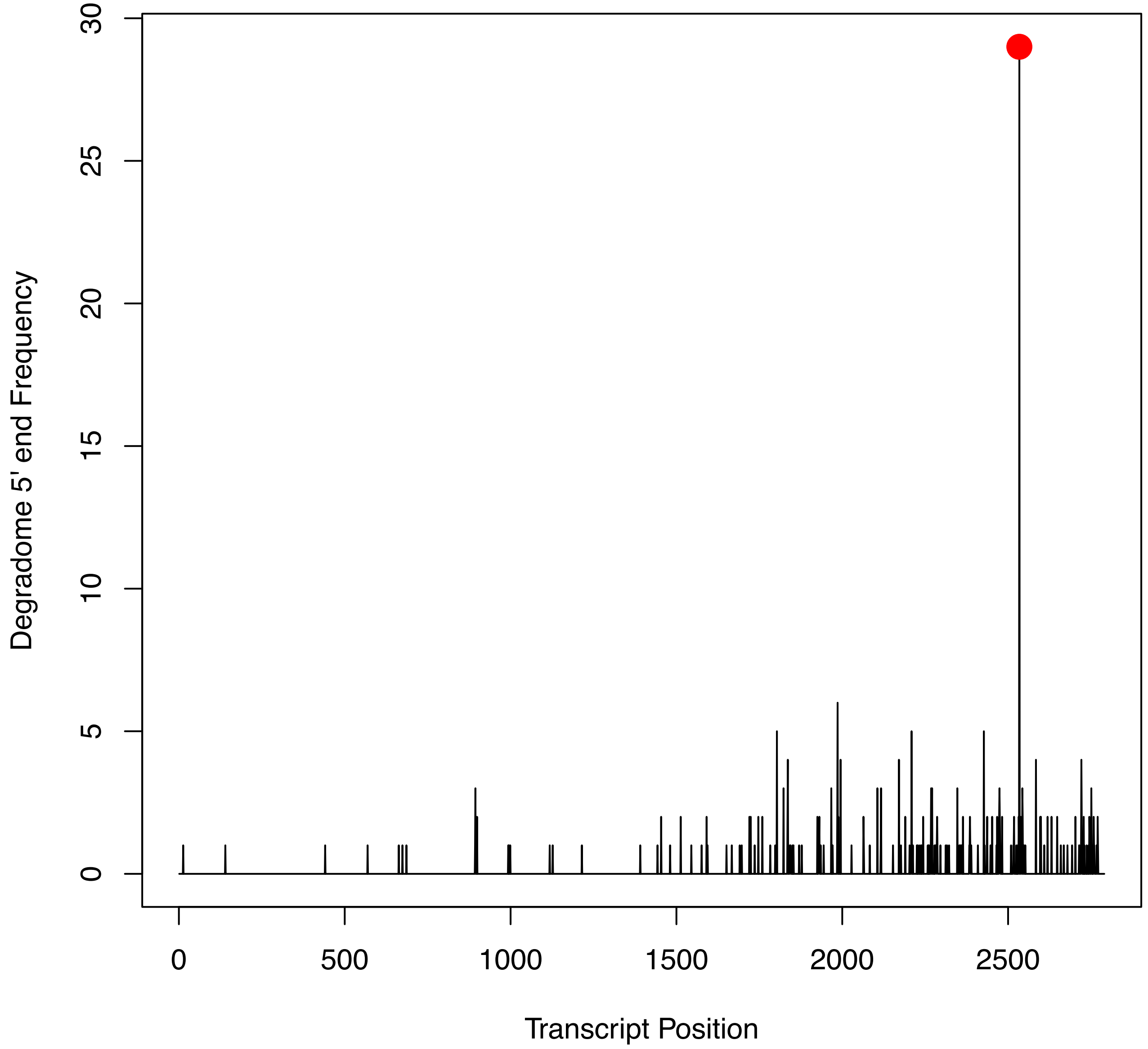

D=Day5
T=HORVU.MOREX.r3.6HG0566320.1
Q=miR167–5p.Cluster_4244
S=2534
category=0
p=0.00162536221265974

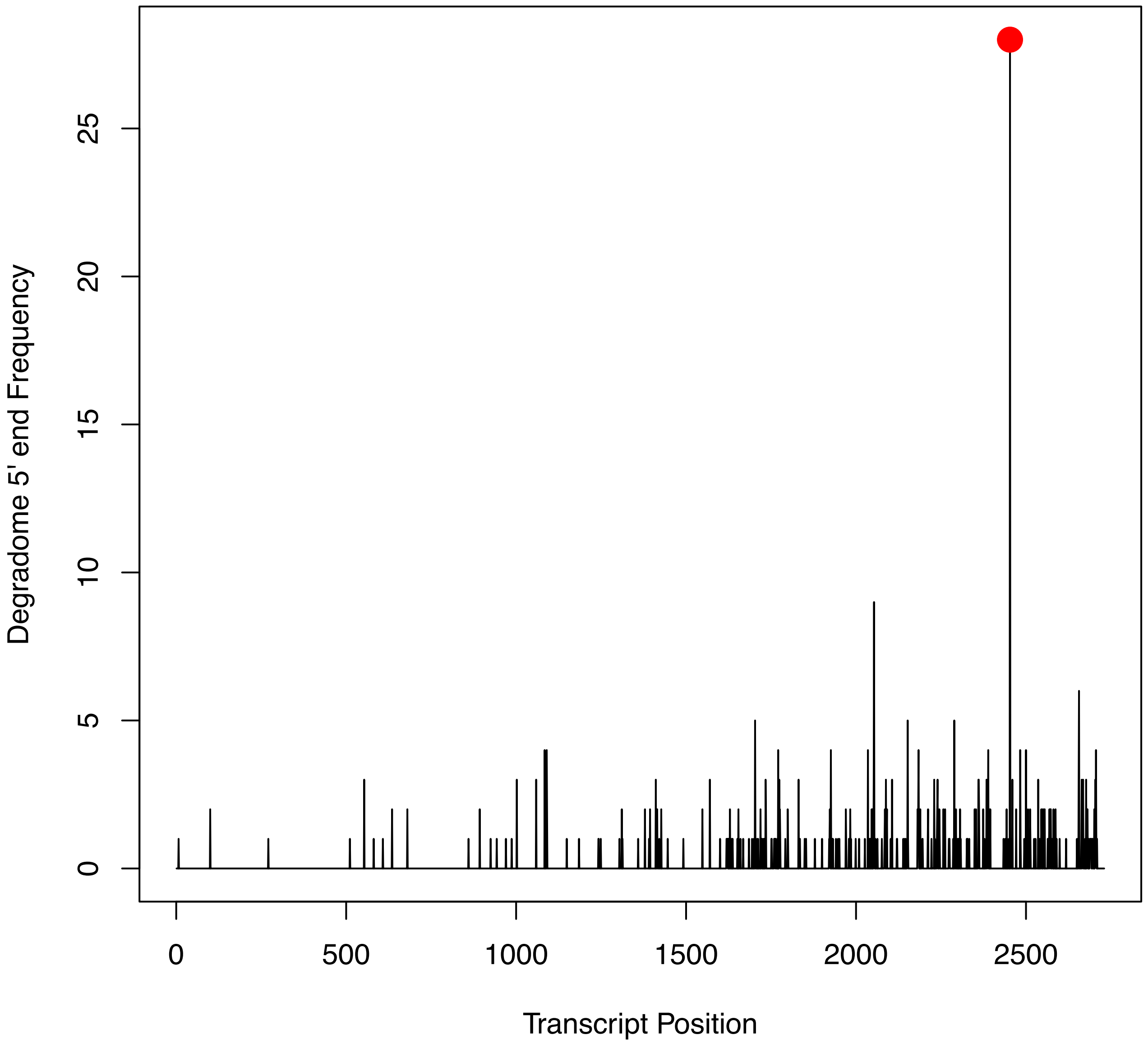

D=Day5
T=HORVU.MOREX.r3.7HG0735280.1
Q=miR167–5p.Cluster_4244
S=2453
category=0
p=0.00121926949634599

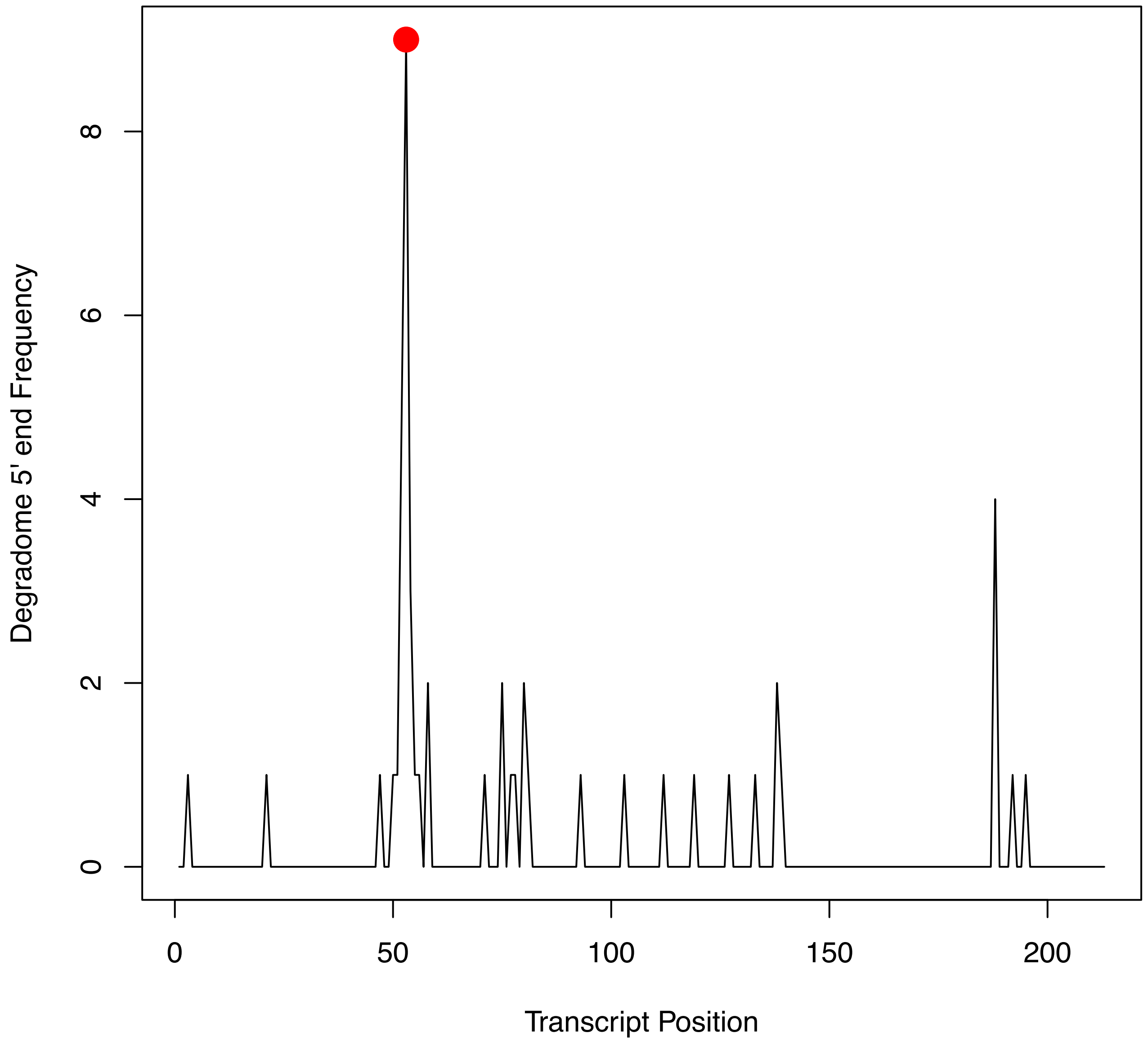

D=Day5
T=HORVU.MOREX.r3.6HG0540130.1
Q=miR168–3p.Cluster_4564
S=53
category=0
p=0.043748021705416

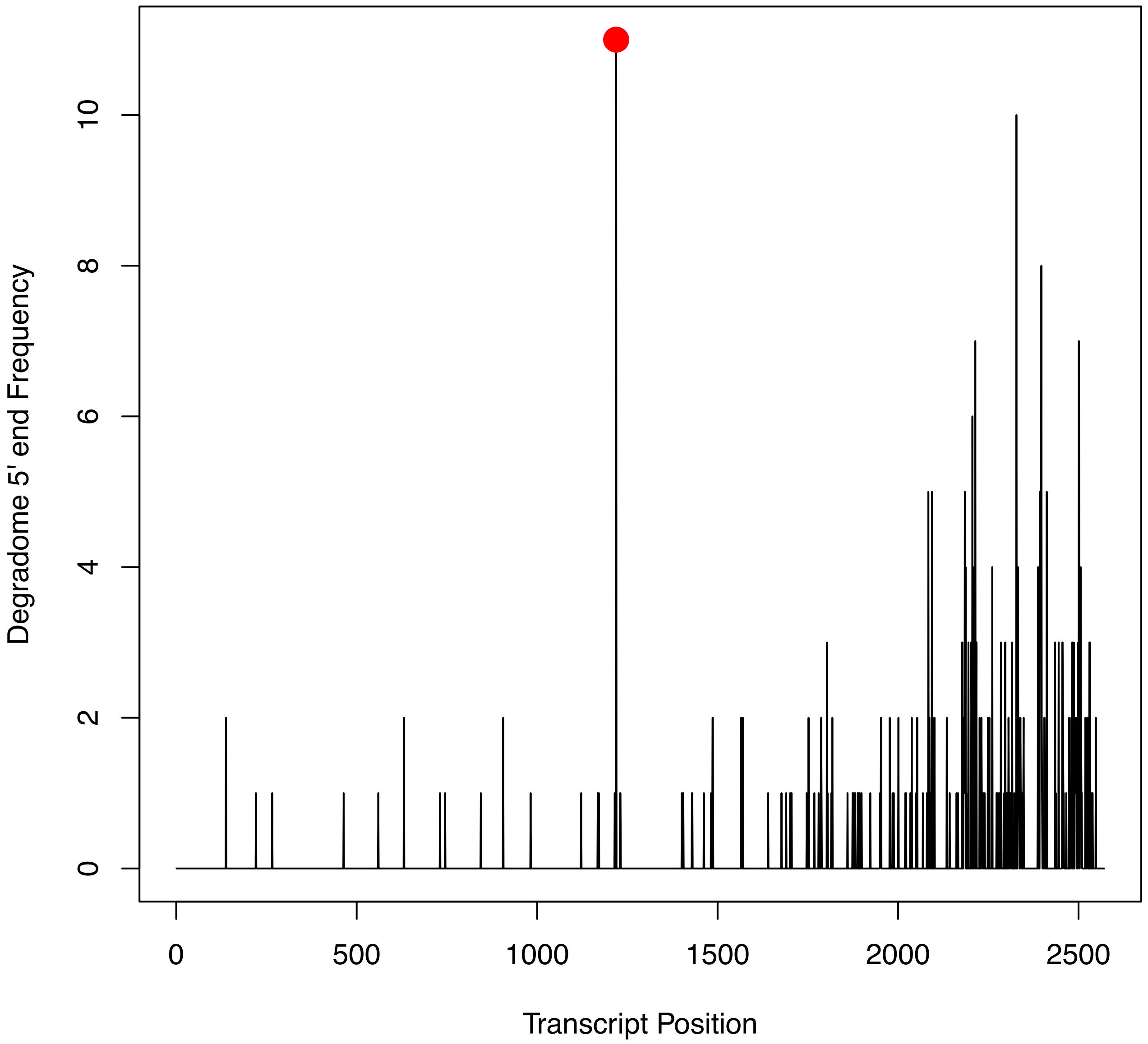

D=Day5
T=HORVU.MOREX.r3.1HG0055960.1
Q=miR171–3p.Cluster_1682
S=1219
category=0
p=0.00162536221265974

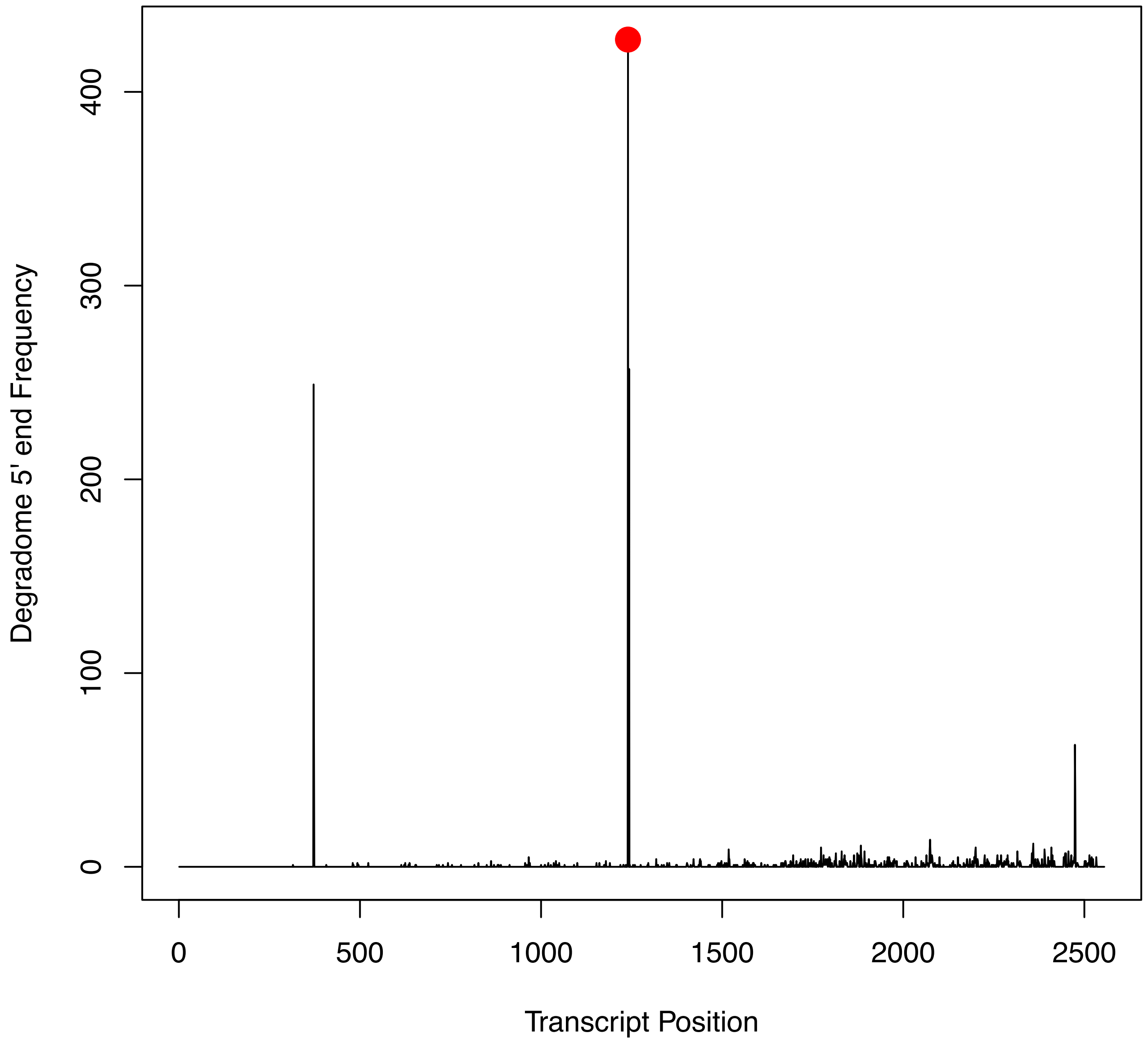

D=Day5
T=HORVU.MOREX.r3.6HG0601750.1
Q=miR171–3p.Cluster_1682
S=1240
category=0
p=0.000813011600260927

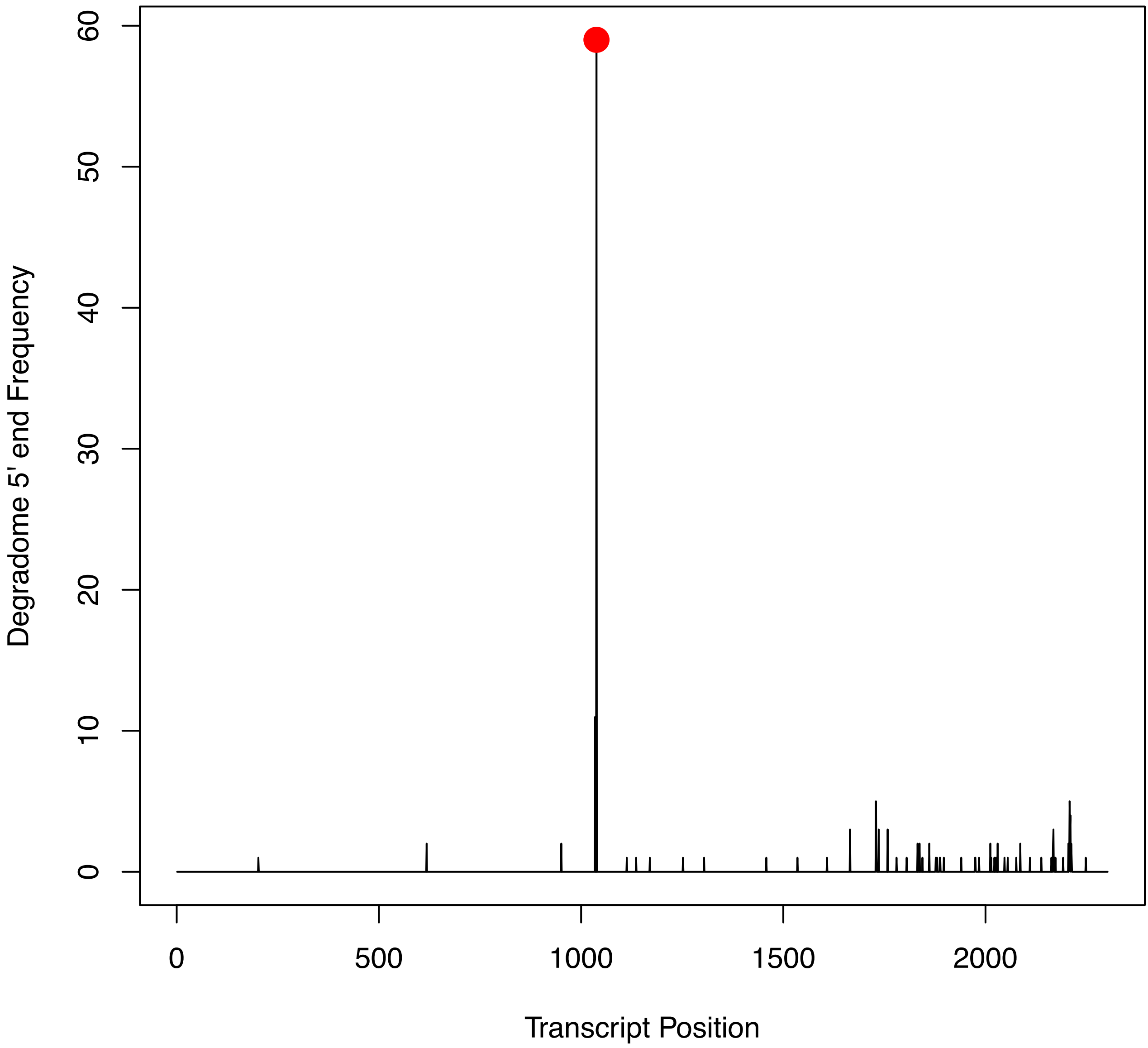

D=Day5
T=HORVU.MOREX.r3.4HG0415480.1
Q=miR171–3p.Cluster_3461
S=1038
category=0
p=0.000813011600260927

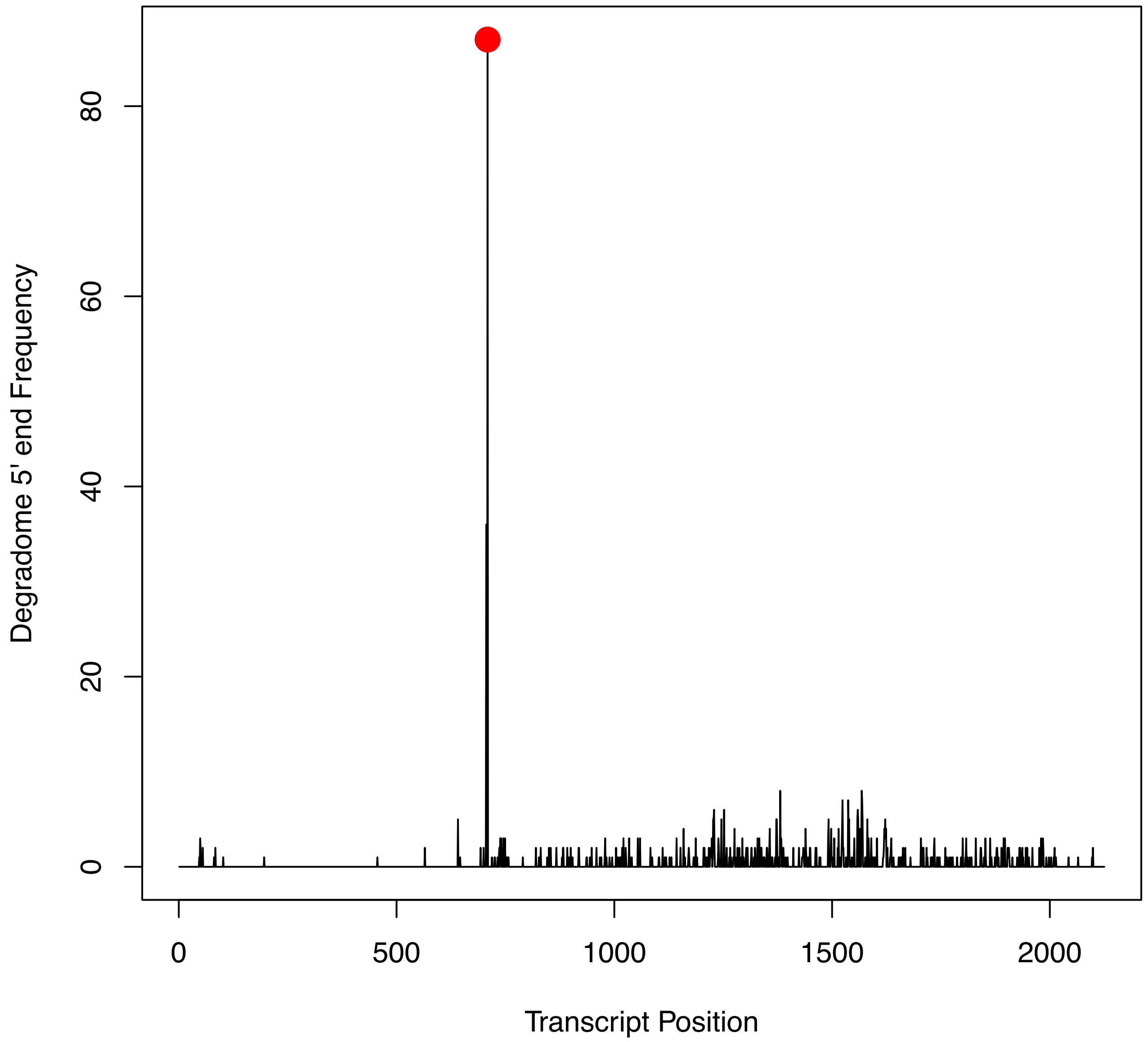

D=Day5
T=HORVU.MOREX.r3.7HG0635740.1
Q=miR171–3p.Cluster_3461
S=709
category=0
p=0.000406588457217305

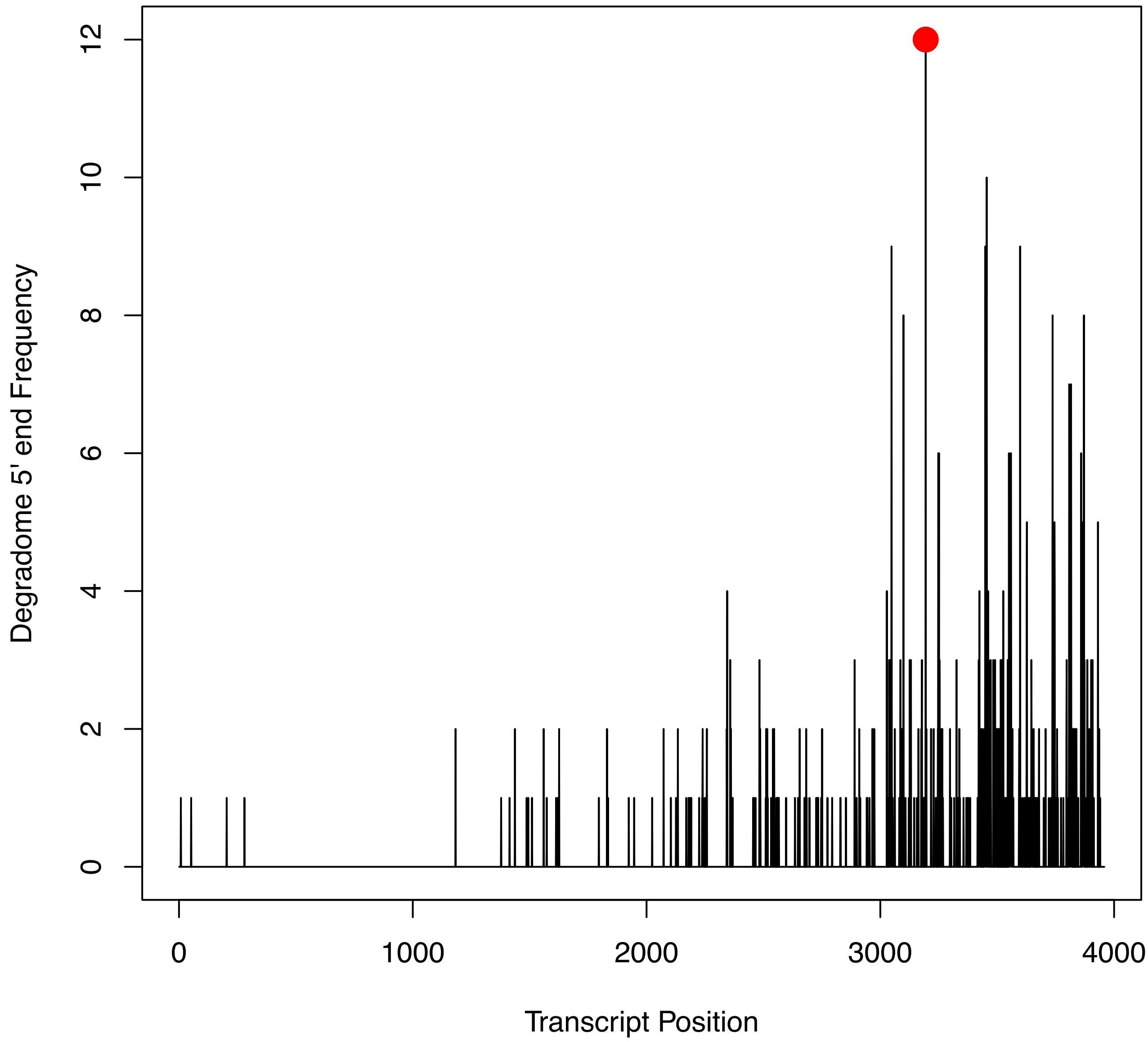

D=Day5
T=HORVU.MOREX.r3.1HG0073090.1
Q=miR396−5p.Cluster_1803
S=3194
category=0
p=0.0177343914047864

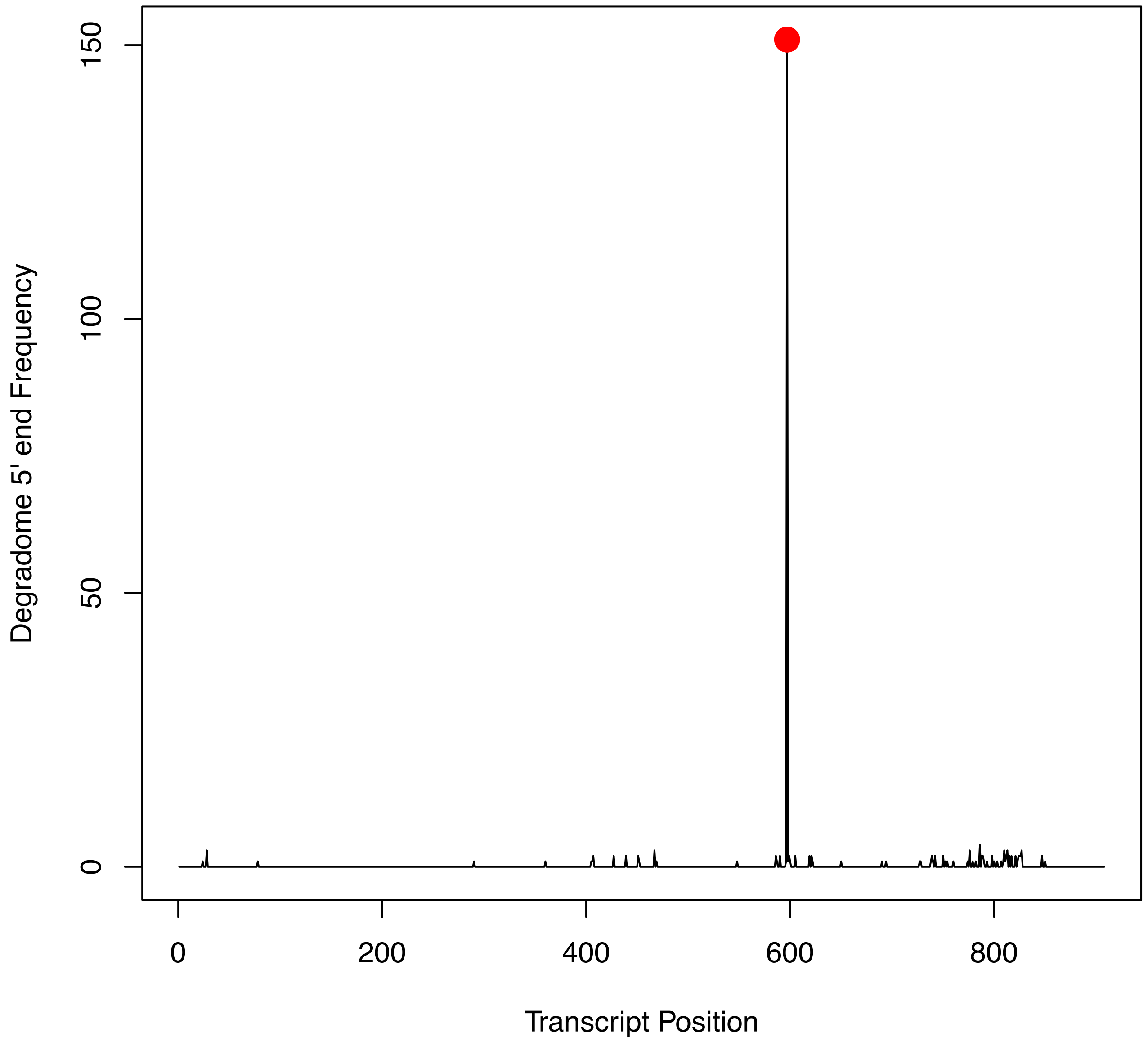

D=Day5
T=HORVU.MOREX.r3.2HG0186750.1
Q=miR396–5p.Cluster_1803
S=597
category=0
p=0.000406588457217305

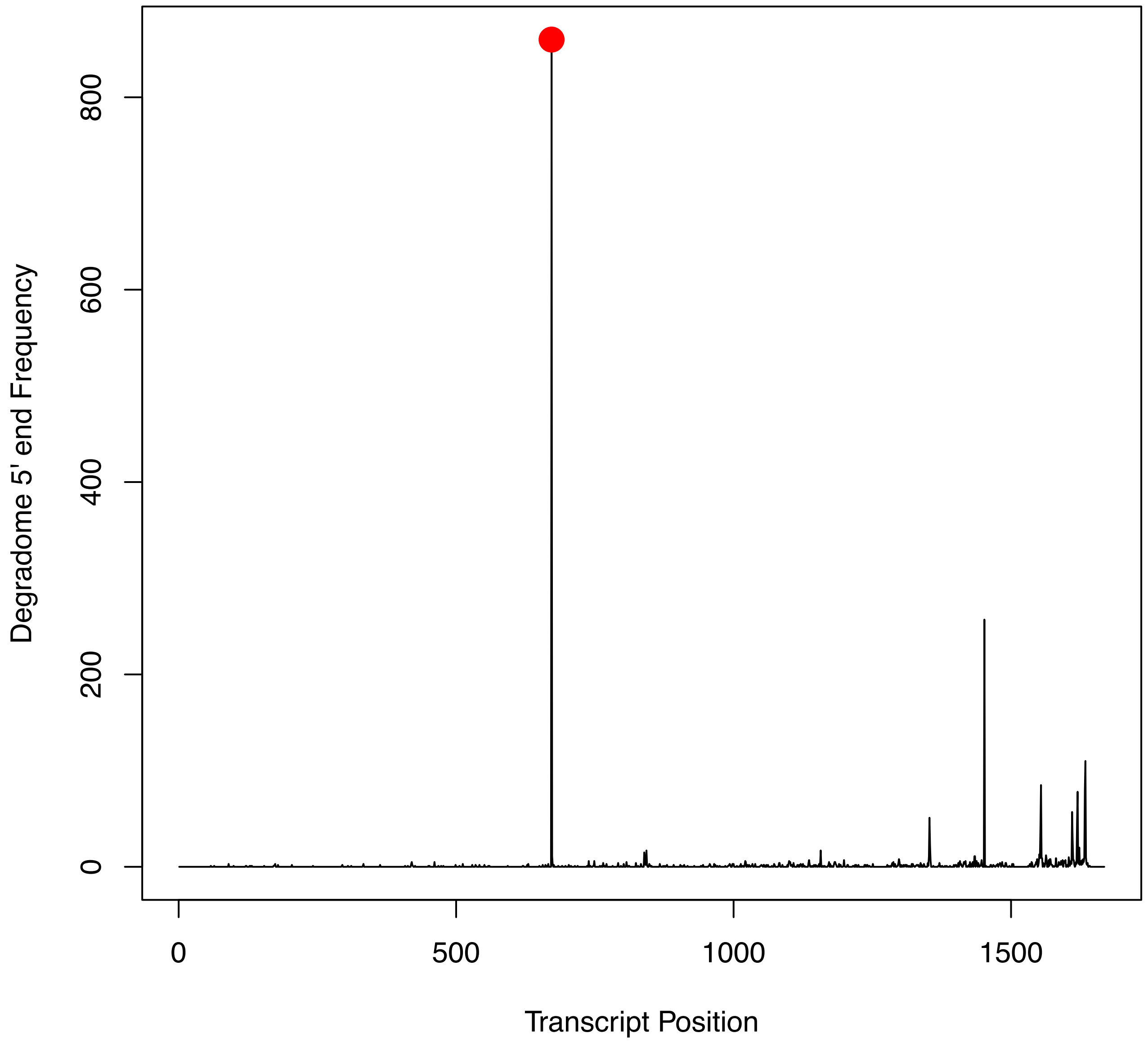

D=Day5
T=HORVU.MOREX.r3.2HG0193490.1
Q=miR396–5p.Cluster_1803
S=672
category=0
p=0.00284264995443928

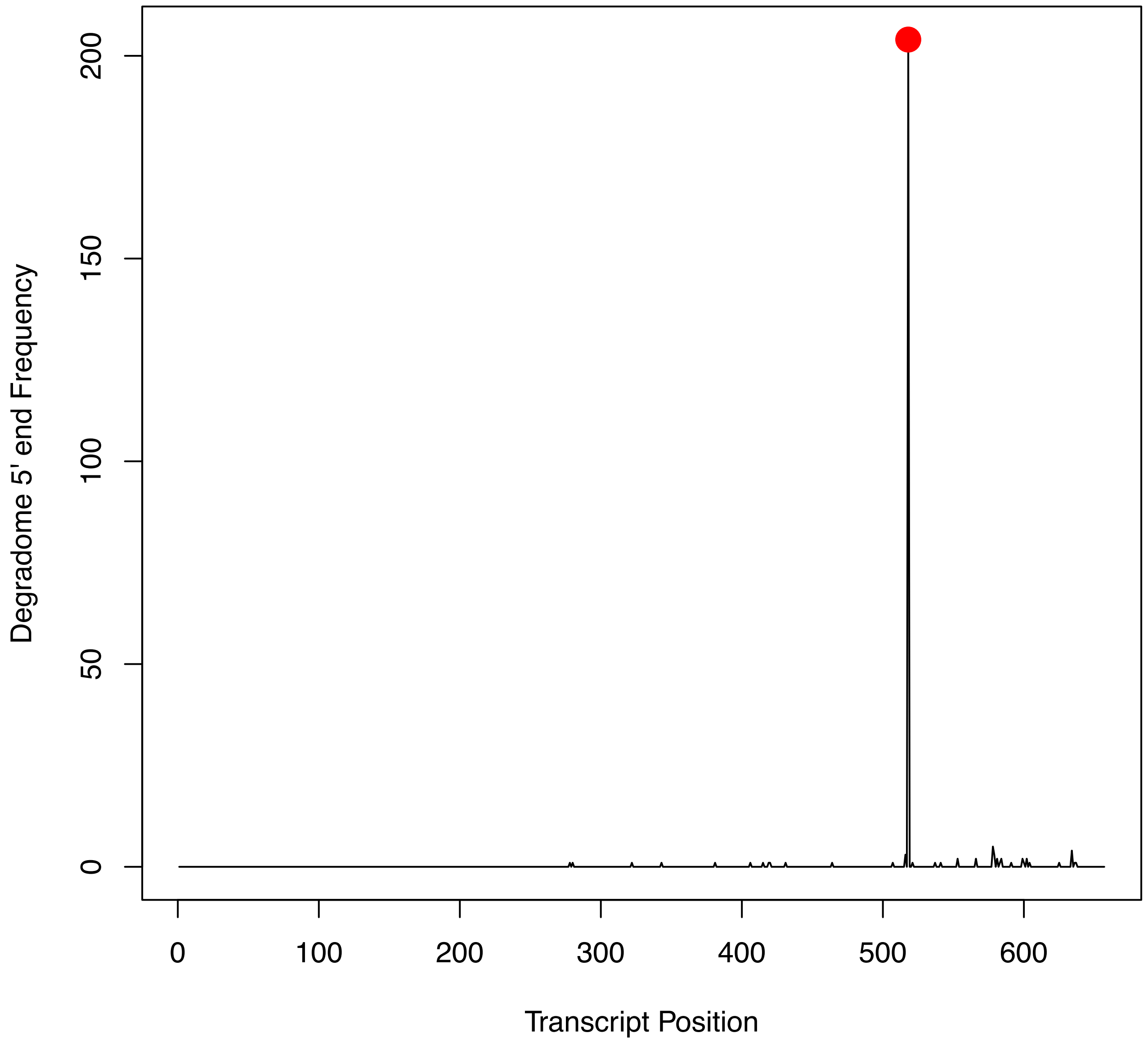

D=Day5
T=HORVU.MOREX.r3.6HG0603870.1
Q=miR396–5p.Cluster_1803
S=518
category=0
p=0.00203128981636258

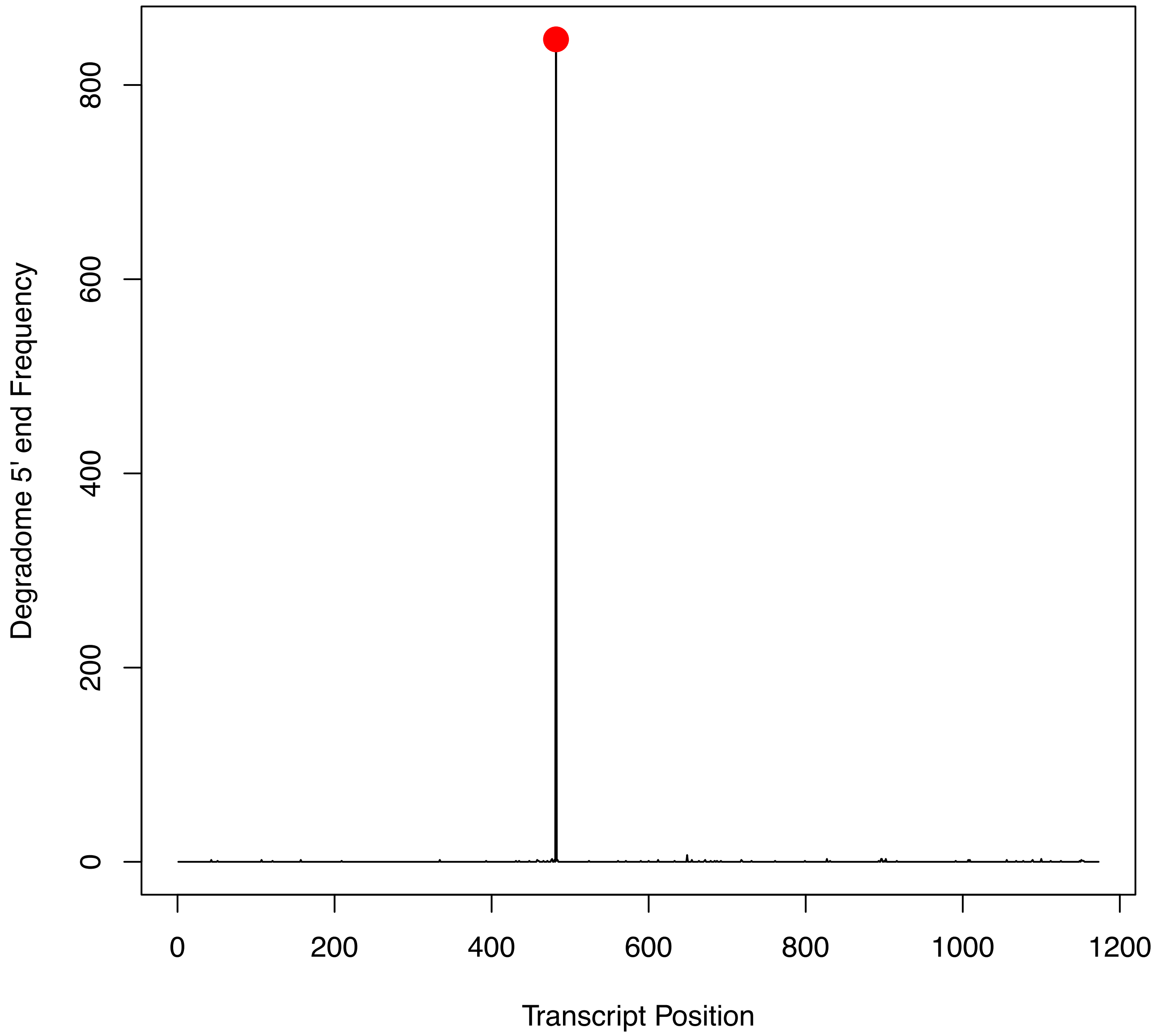

D=Day5
T=HORVU.MOREX.r3.6HG0606810.1
Q=miR396–5p.Cluster_1803
S=482
category=0
p=0.00365335044731174

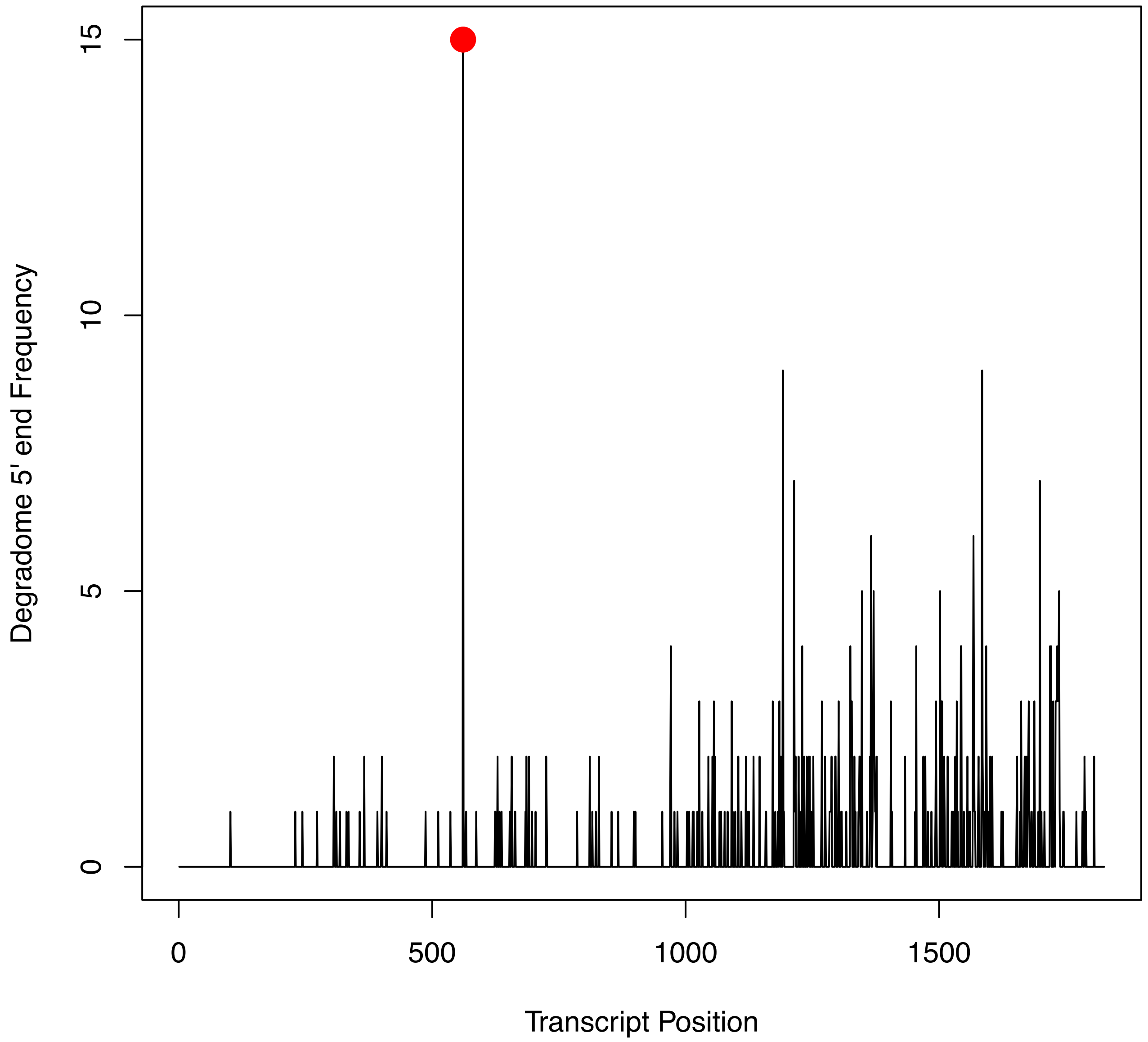

D=Day5
T=HORVU.MOREX.r3.6HG0620090.1
Q=miR396–5p.Cluster_1803
S=561
category=0
p=0.000813011600260927

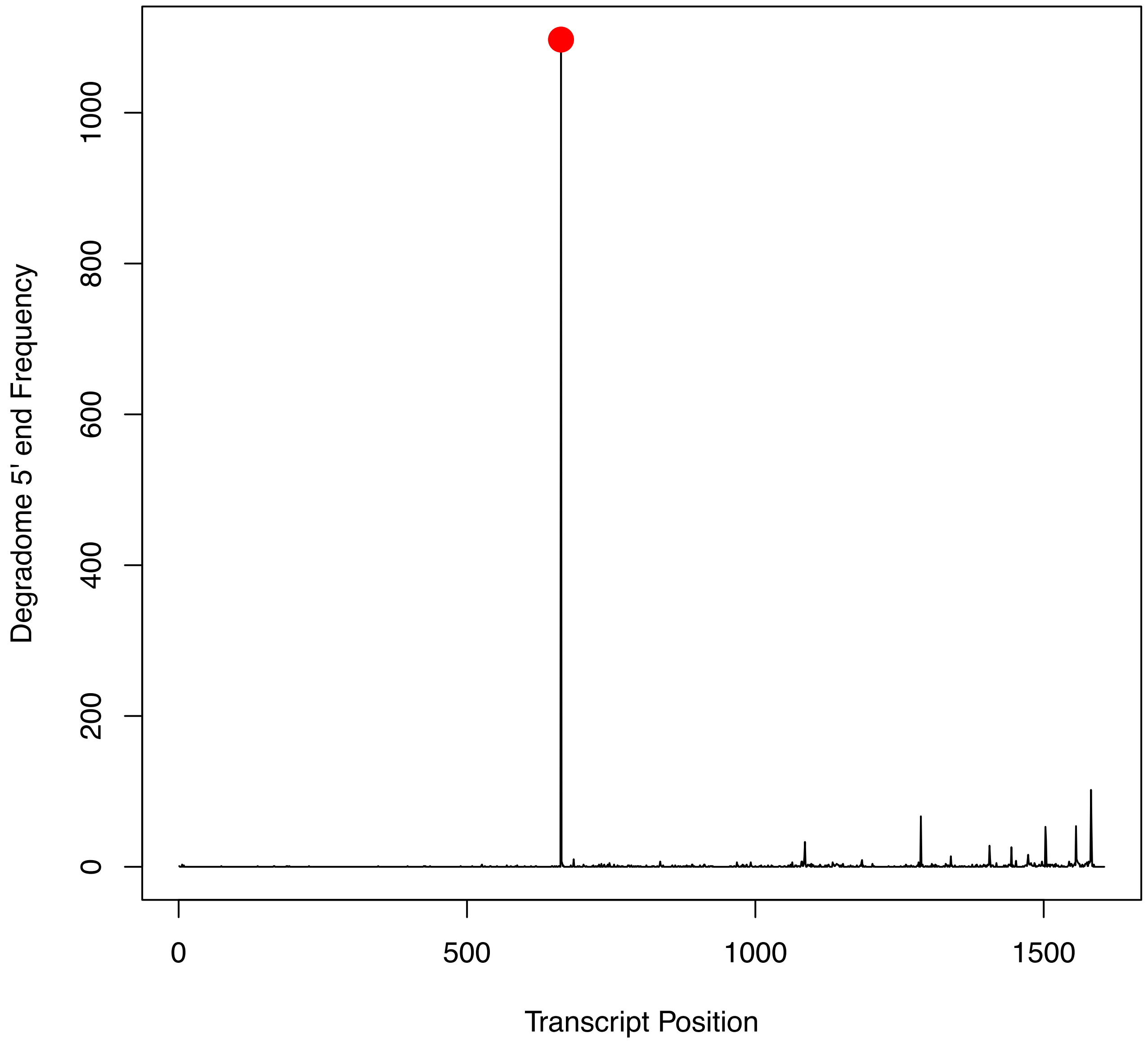

D=Day5
T=HORVU.MOREX.r3.7HG0641080.1
Q=miR396–5p.Cluster_1803
S=663
category=0
p=0.0016253622126597

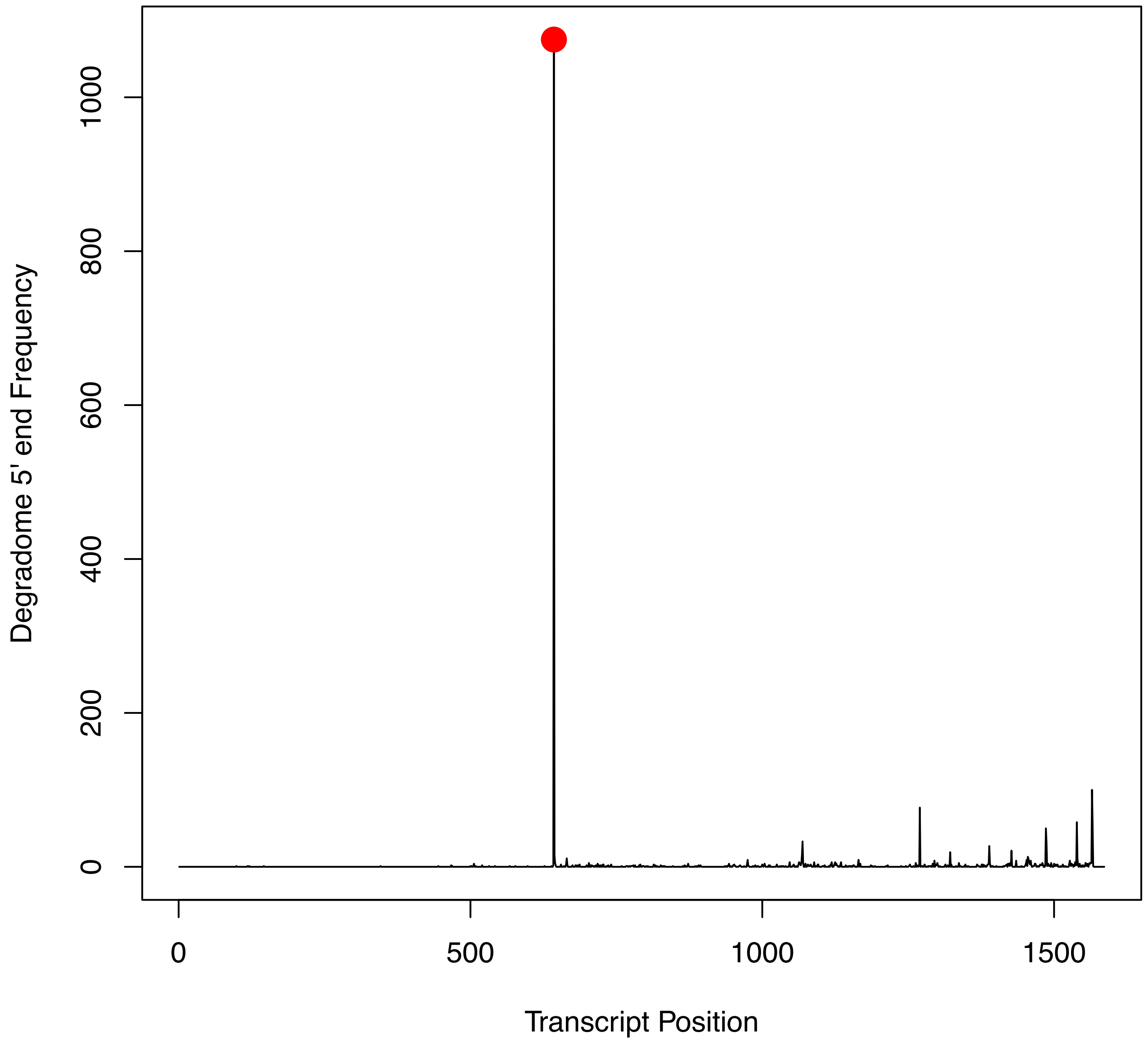

D=Day5
T=HORVU.MOREX.r3.7HG0641080.2
Q=miR396–5p.Cluster_1803
S=643
category=0
p=0.00121926949634599

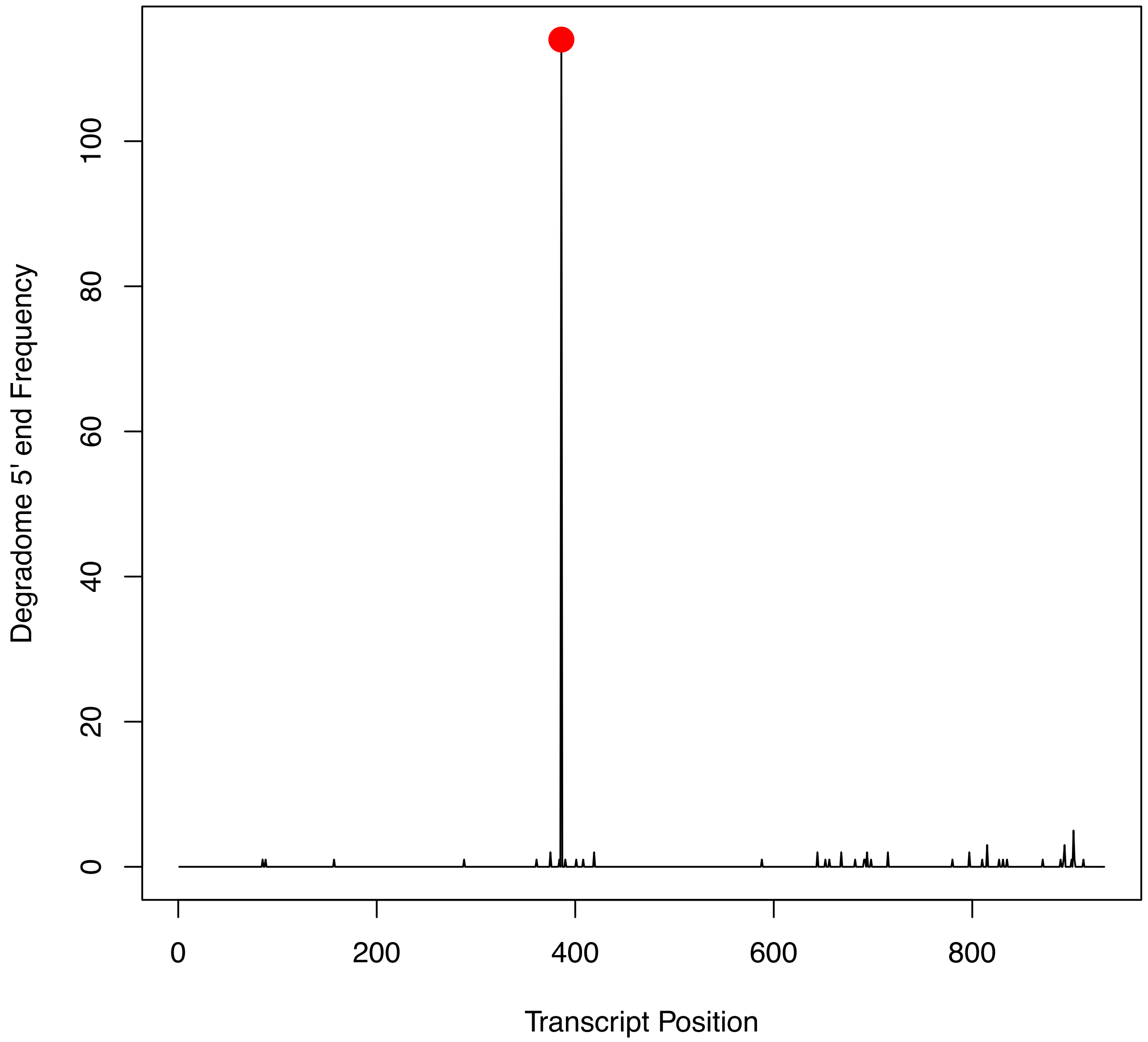

D=Day5
T=HORVU.MOREX.r3.7HG0662690.1
Q=miR396–5p.Cluster_1803
S=386
category=0
p=0.00243705237458713

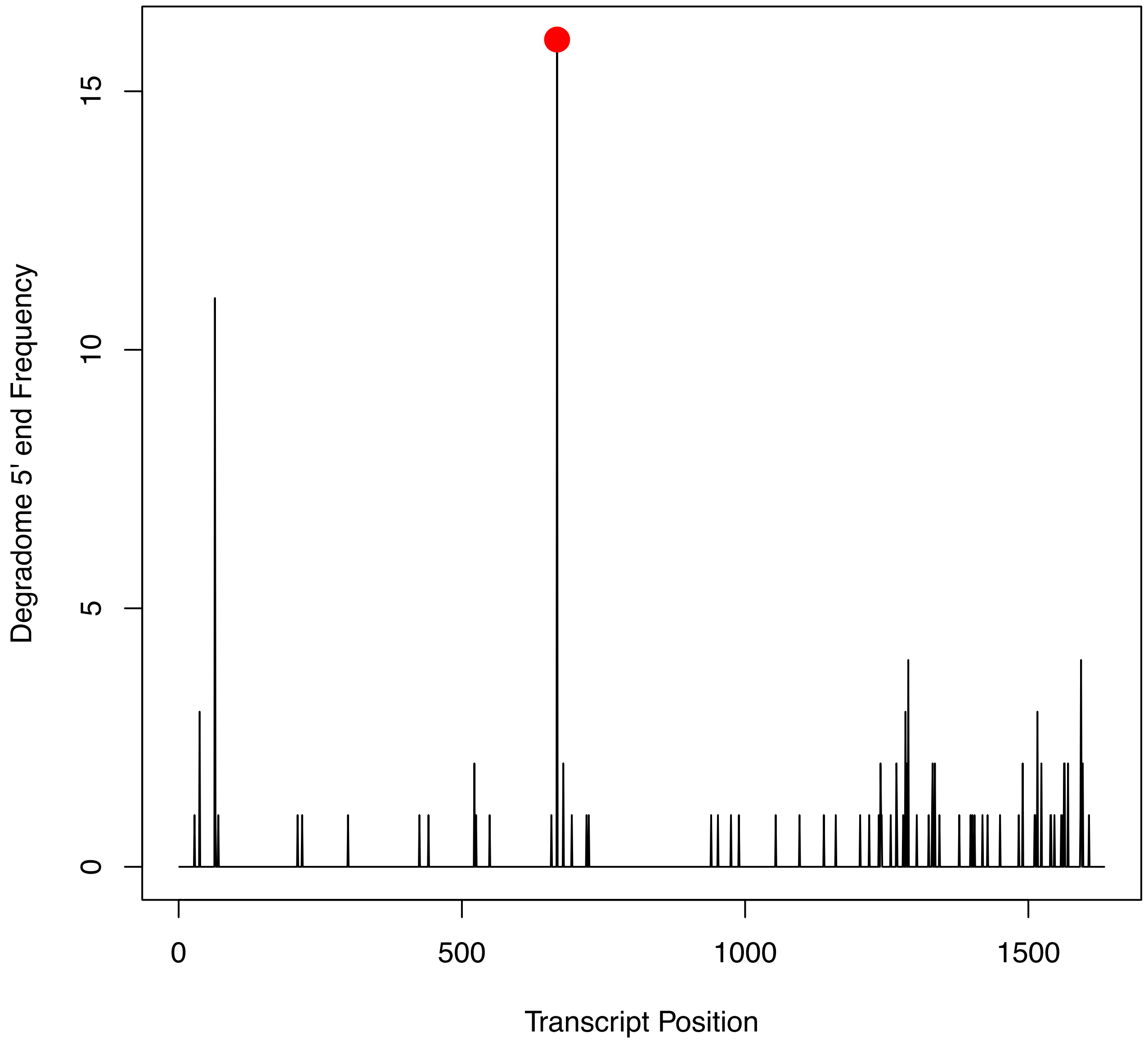

D=Day5
T=HORVU.MOREX.r3.4HG0334080.1
Q=miR396–5p.Cluster_5480
S=668
category=0
p=0.00284264995443928

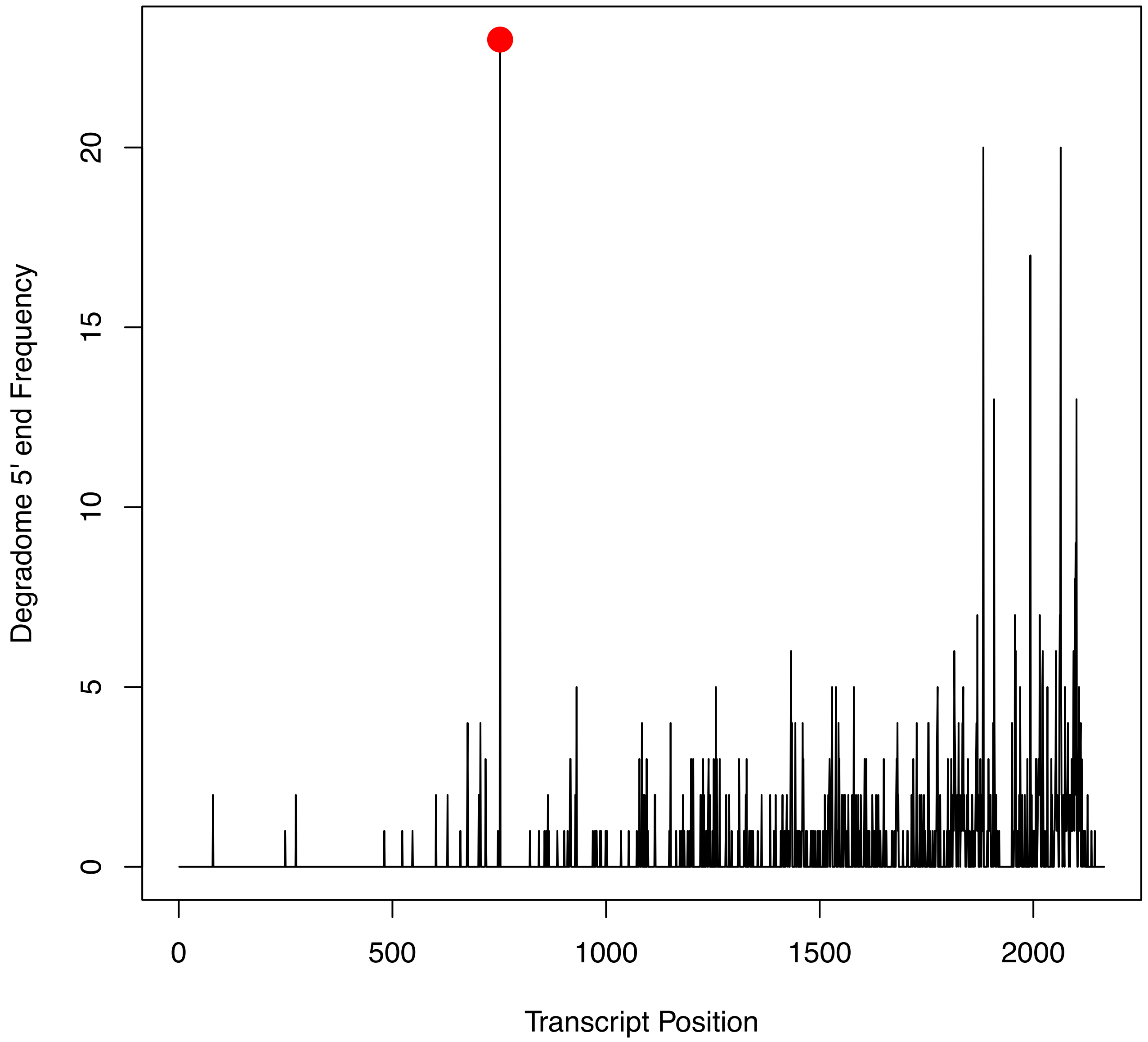

D=Day5
T=HORVU.MOREX.r3.4HG0339430.2
Q=miR396–5p.Cluster_5480
S=752
category=0
p=0.00405845349440703

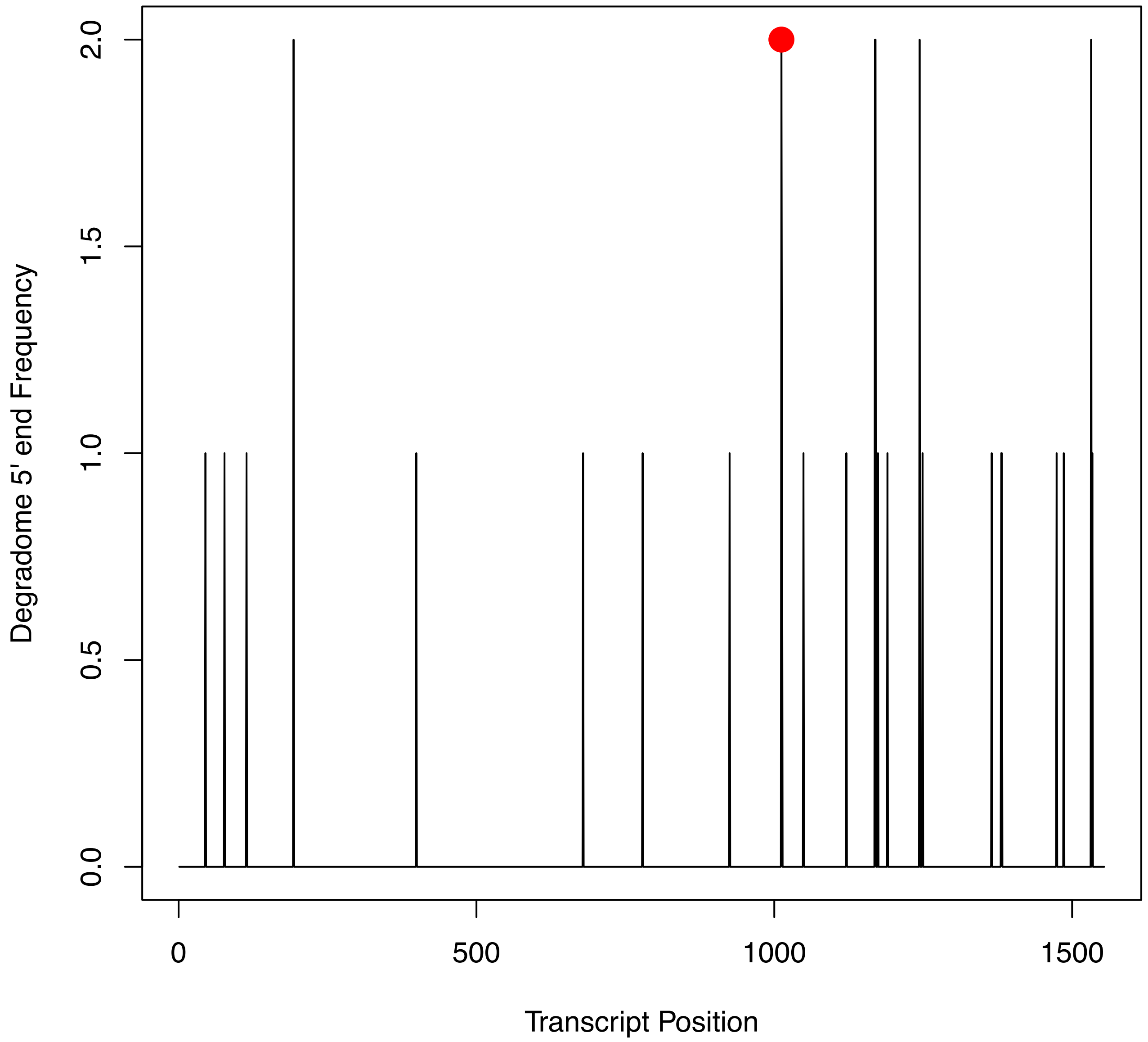

D=Day5
T=HORVU.MOREX.r3.3HG0319380.1
Q=miR6201–5p.Cluster_5317
S=1012
category=1
p=0.0022324164632666

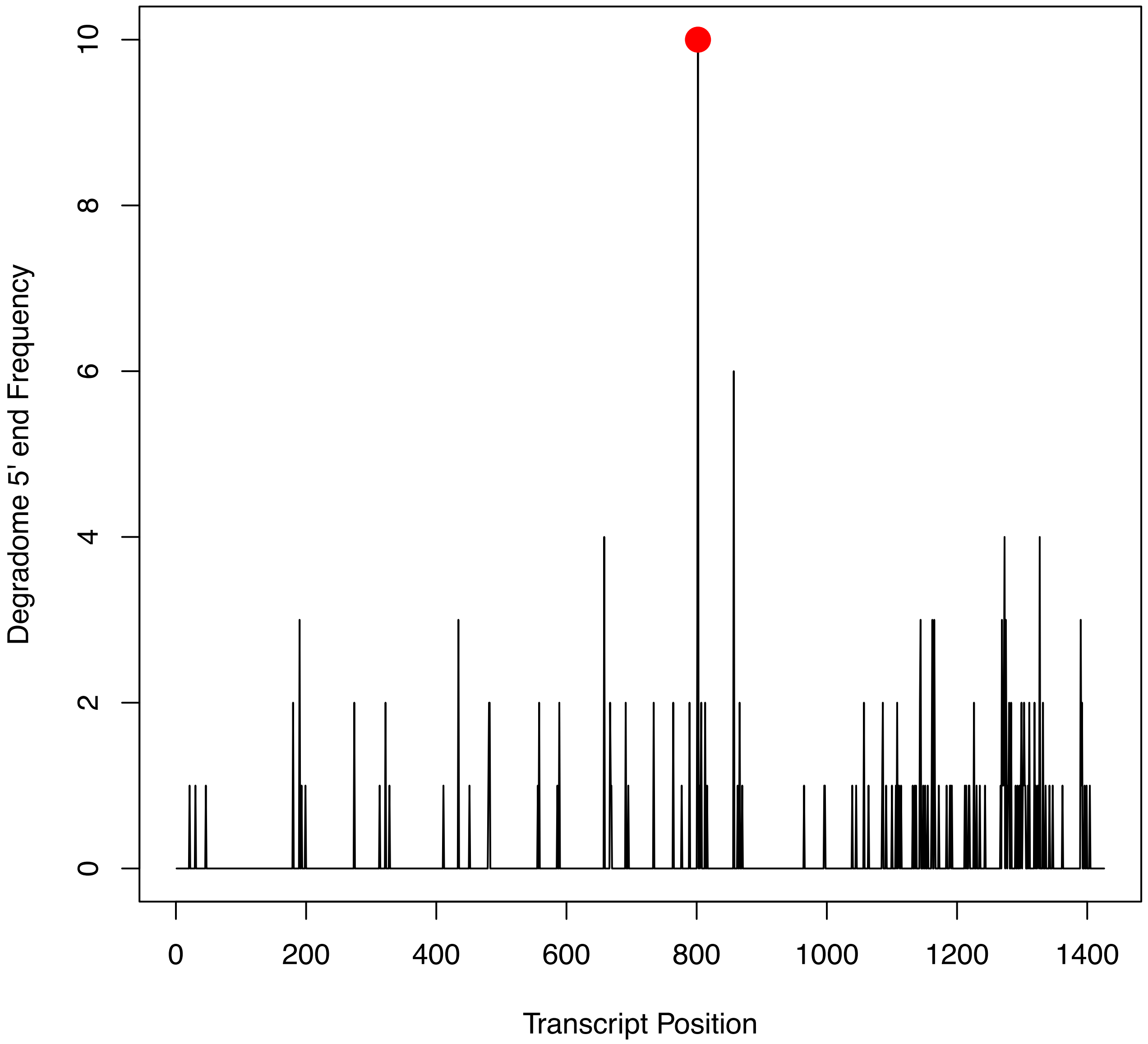

D=Day5
T=HORVU.MOREX.r3.6HG0541280.1
Q=miR9662–3p.Cluster_4660
S=802
category=0
p=0.0339764311007645

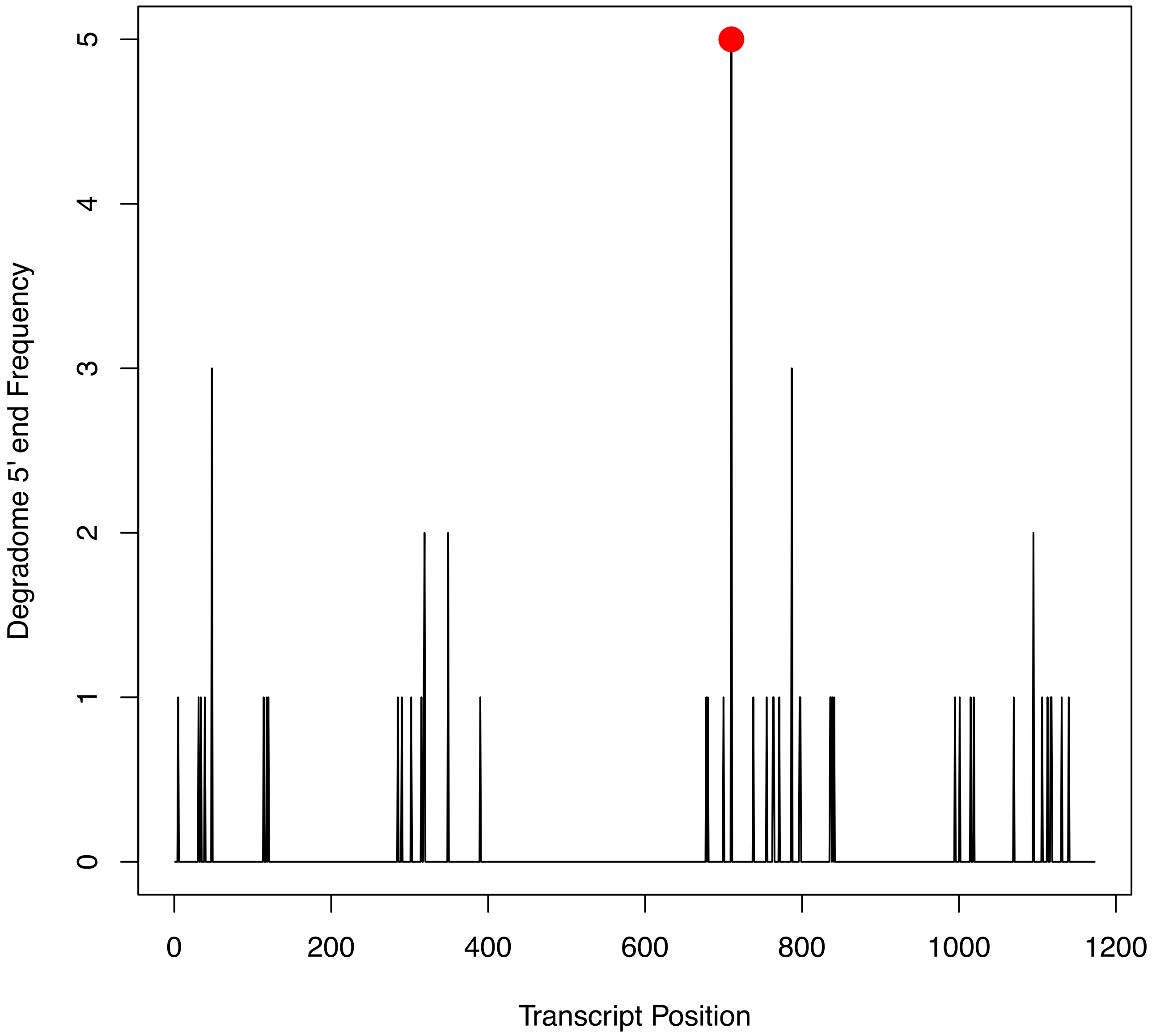

D=Day5
T=HORVU.MOREX.r3.6HG0543320.1
Q=miR9662–3p.Cluster_4660
S=710
category=0
p=0.0169351482765263

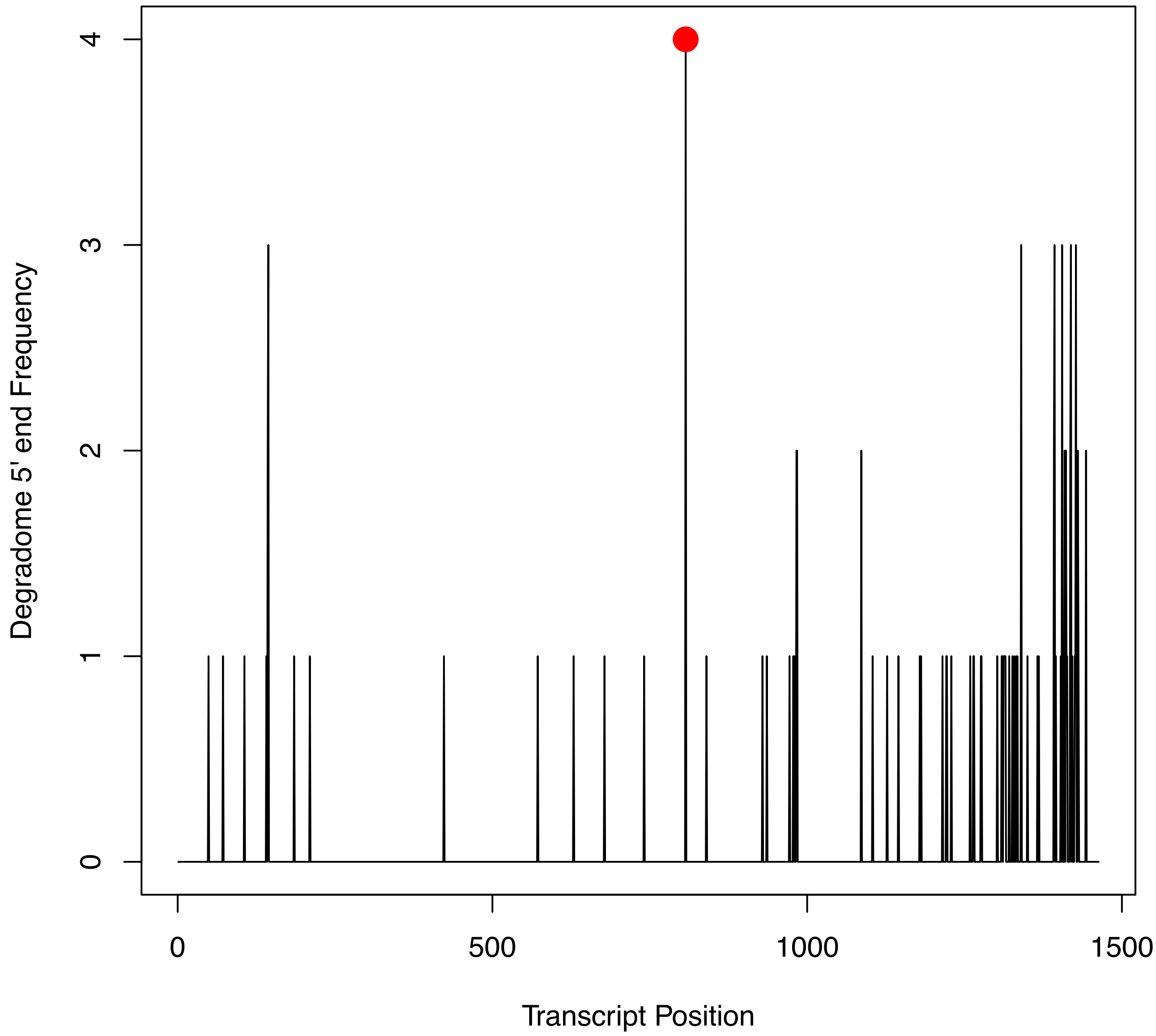

D=Day5
T=HORVU.MOREX.r3.6HG0543350.1
Q=miR9662–3p.Cluster_4660
S=807
category=0
p=0.0177343914047864

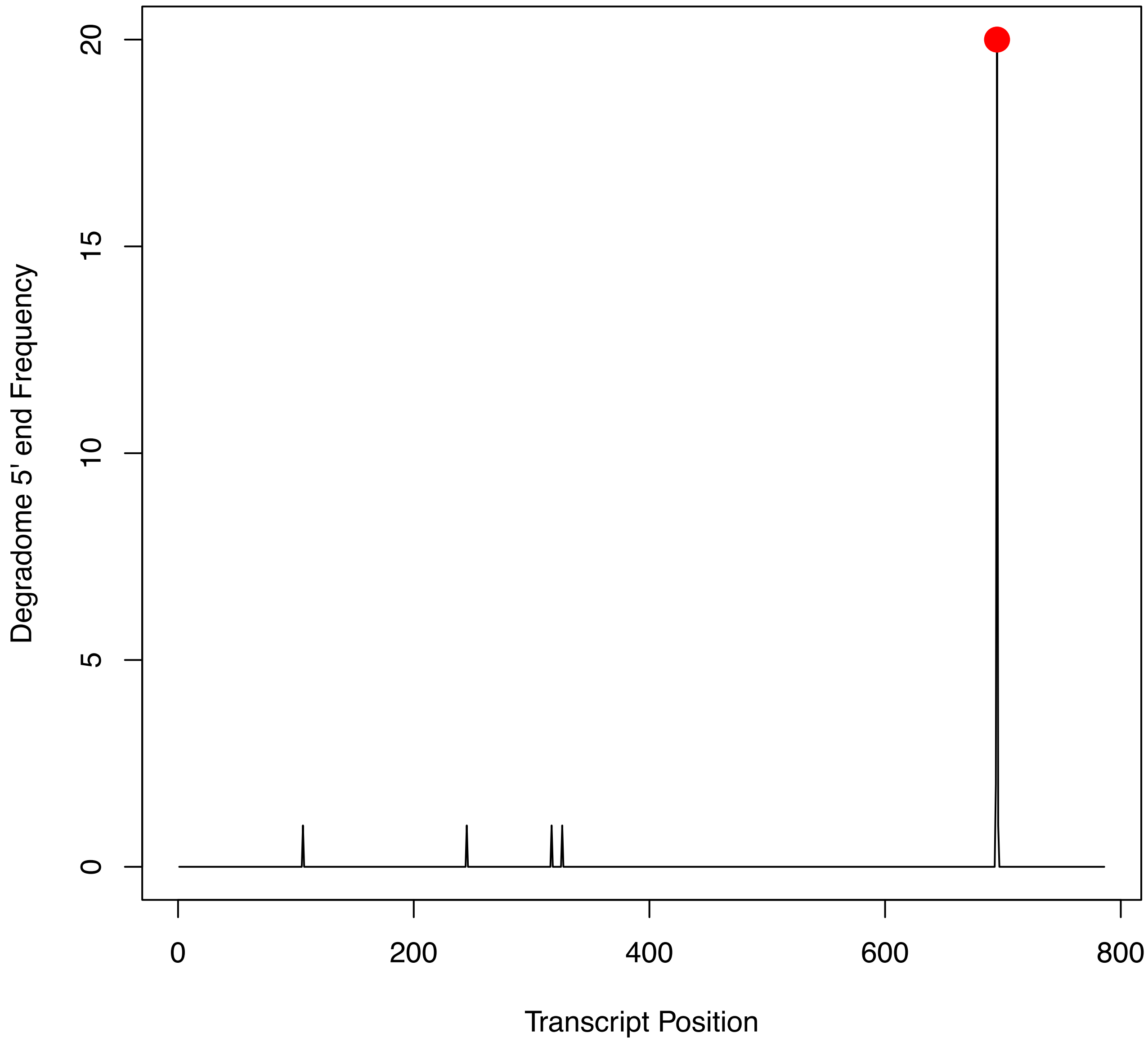

D=Day5
T=HORVU.MOREX.r3.6HG0543460.1
Q=miR9662–3p.Cluster_4660
S=695
category=0
p=0.000813011600260927

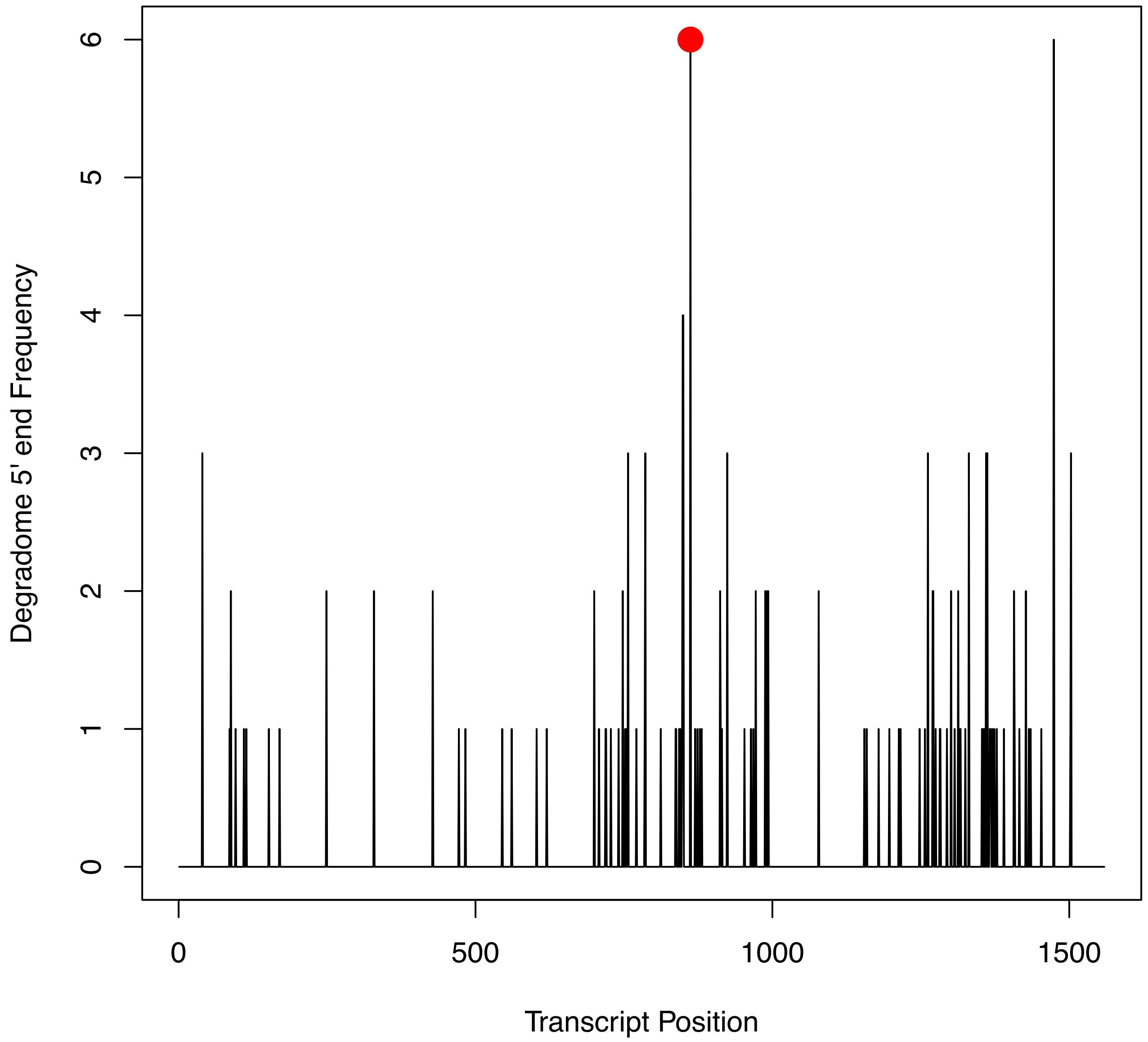

D=Day5
T=HORVU.MOREX.r3.7HG0665830.1
Q=miR9662–3p.Cluster_4660
S=862
category=1
p=0.005944233517584

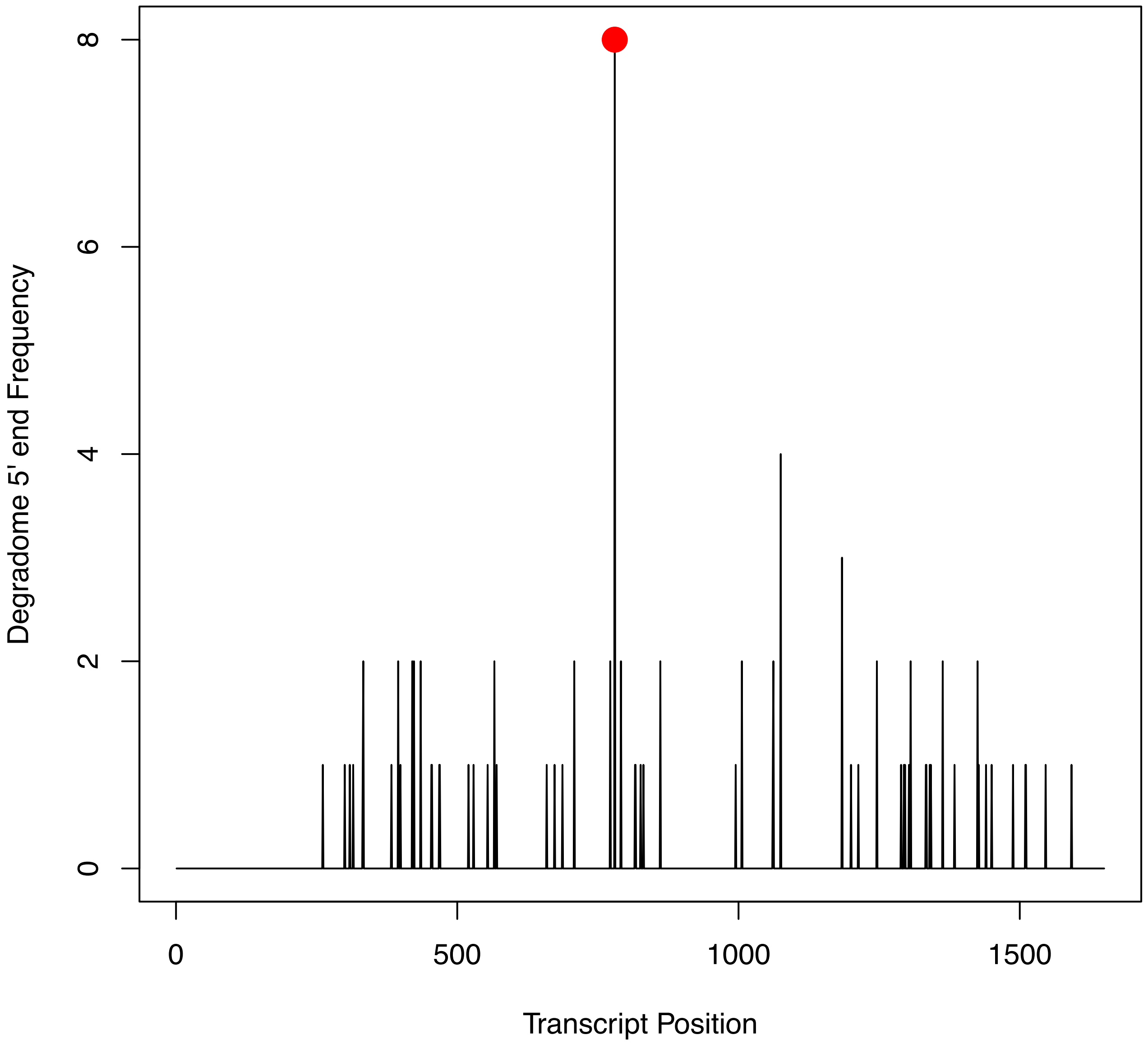

D=Day5
T=HORVU.MOREX.r3.7HG0710980.1
Q=miR9662–3p.Cluster_4660
S=780
category=0
p=0.0161352548256118

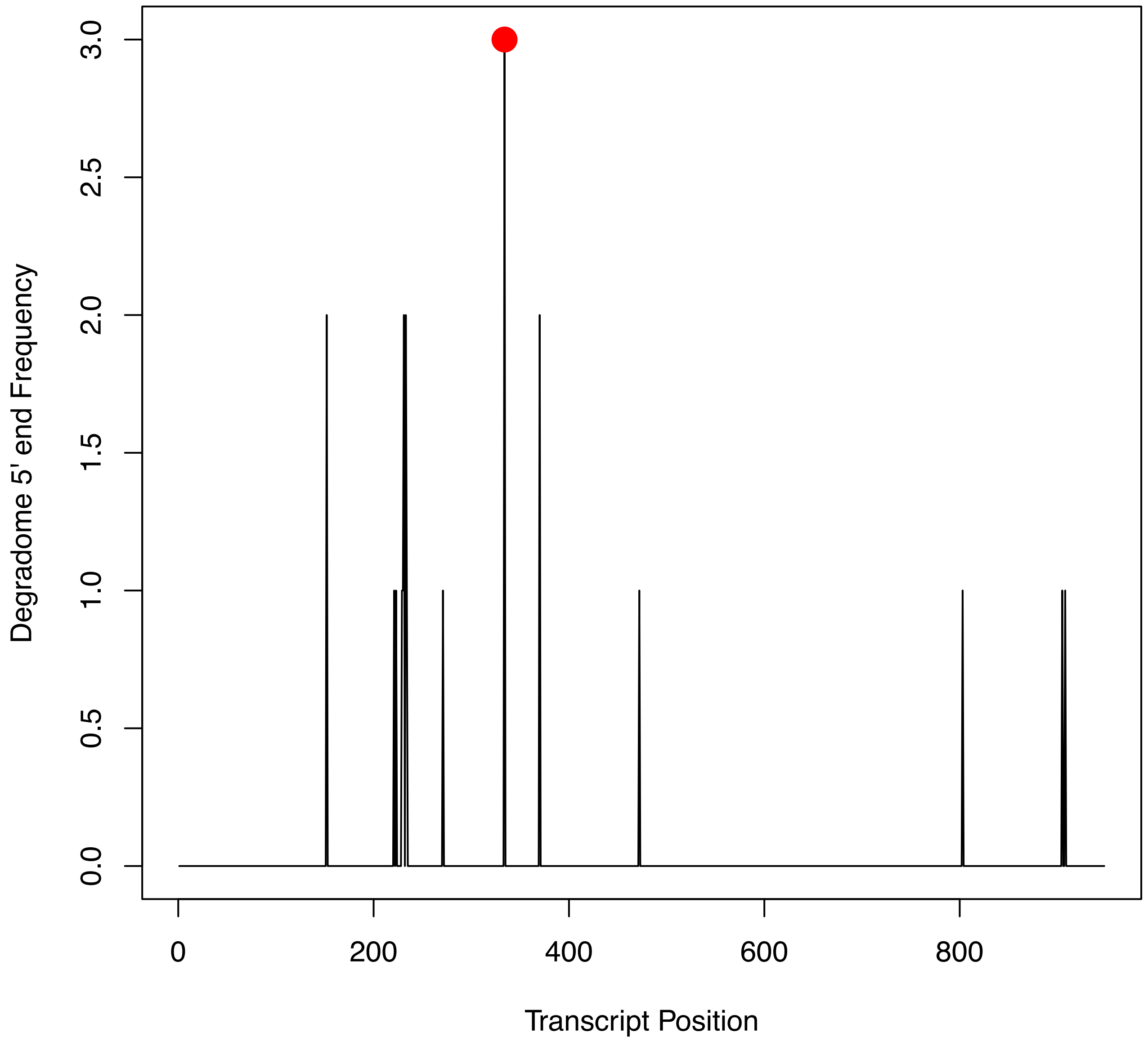

D=Day5
T=HORVU.MOREX.r3.1HG0020310.1
Q=novel–5p.Cluster_1777
S=334
category=0
p=0.0382881728070126
