# Supplementary material for: Post-Transcriptional Gene Regulation by MicroRNAs During Barley Malting
Source: Genes (Basel). 2026 Jun 9;17(6):676. doi: 10.3390/genes17060676 (PMC13299409; doi:10.3390/genes17060676)

Figure S1

Predicted hairpin structures transcribed from the 33 identified MIR loci. Visualizations produced by strucVis as a part of ShortStack alignment of sRNA reads to the genome and de novo identification of MIR loci. For each locus, the predicted secondary structure of a hypothetical precursor RNA is shown. The start and stop of the precursor RNA are not experimentally determined. The precursor RNA is simply extended ~ 20 nucleotides on either end of the miRNA/miRNA* duplex. Each base in the hypothetical precursor RNA is colored based on the depth of coverage in aligned sRNA reads at that base.

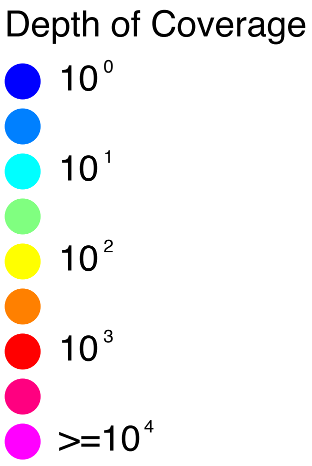

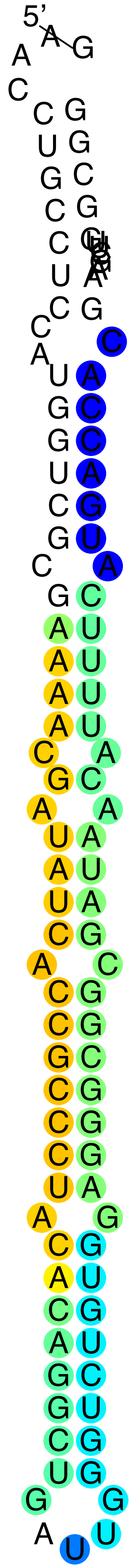

MIR family: unknown
Location: 1H:20913602-20913704
Strand: plus
Name: Cluster_68

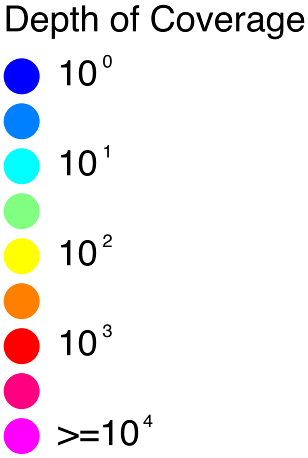

MIR family: unknown
Location: 2H:192275276-192275527
Strand: minus
Name: Cluster_1155

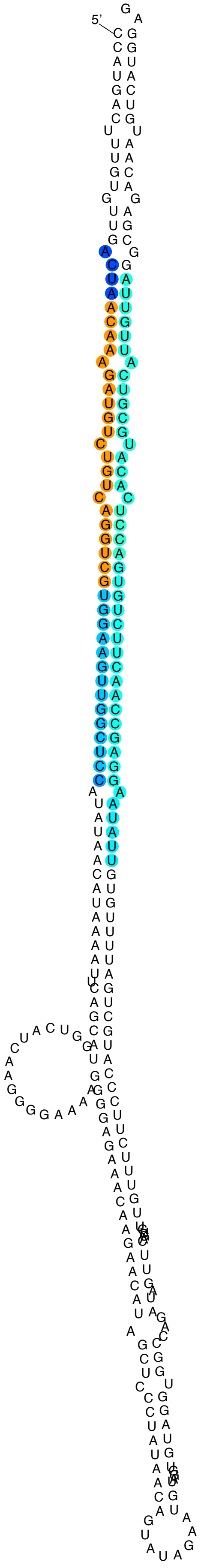

Depth of Coverage

- 10^0
-
- 10^1
-
- 10^2
-
- 10^3
-
- $>=10^4$

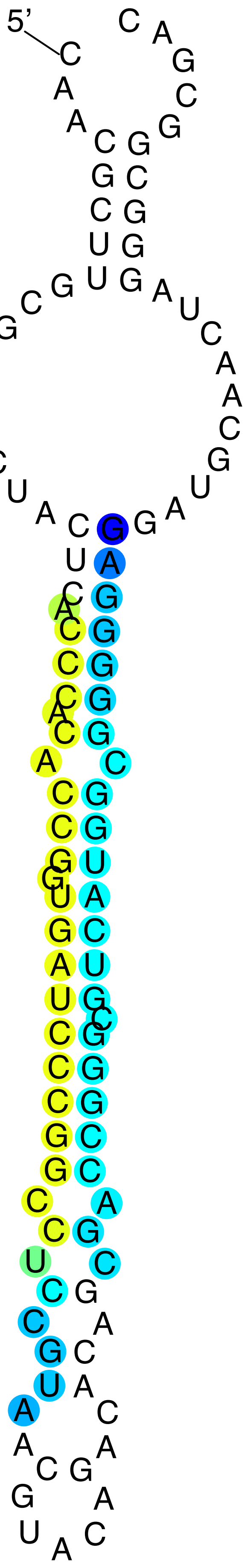

MIR family: unknown
Location: 2H:557132909-557133014
Strand: minus
Name: Cluster_1580

Depth of Coverage

- 10^0
- 10^1
- 10^2
- 10^3
- $>=10^4$

MIR family: unknown

Location: 2H:640670230-640670380

Strand: plus

Name: Cluster_1777

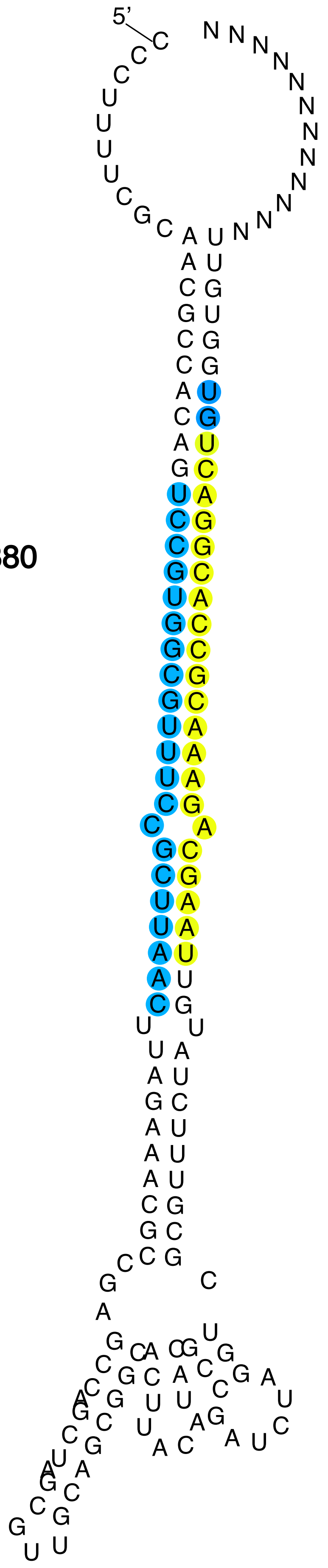

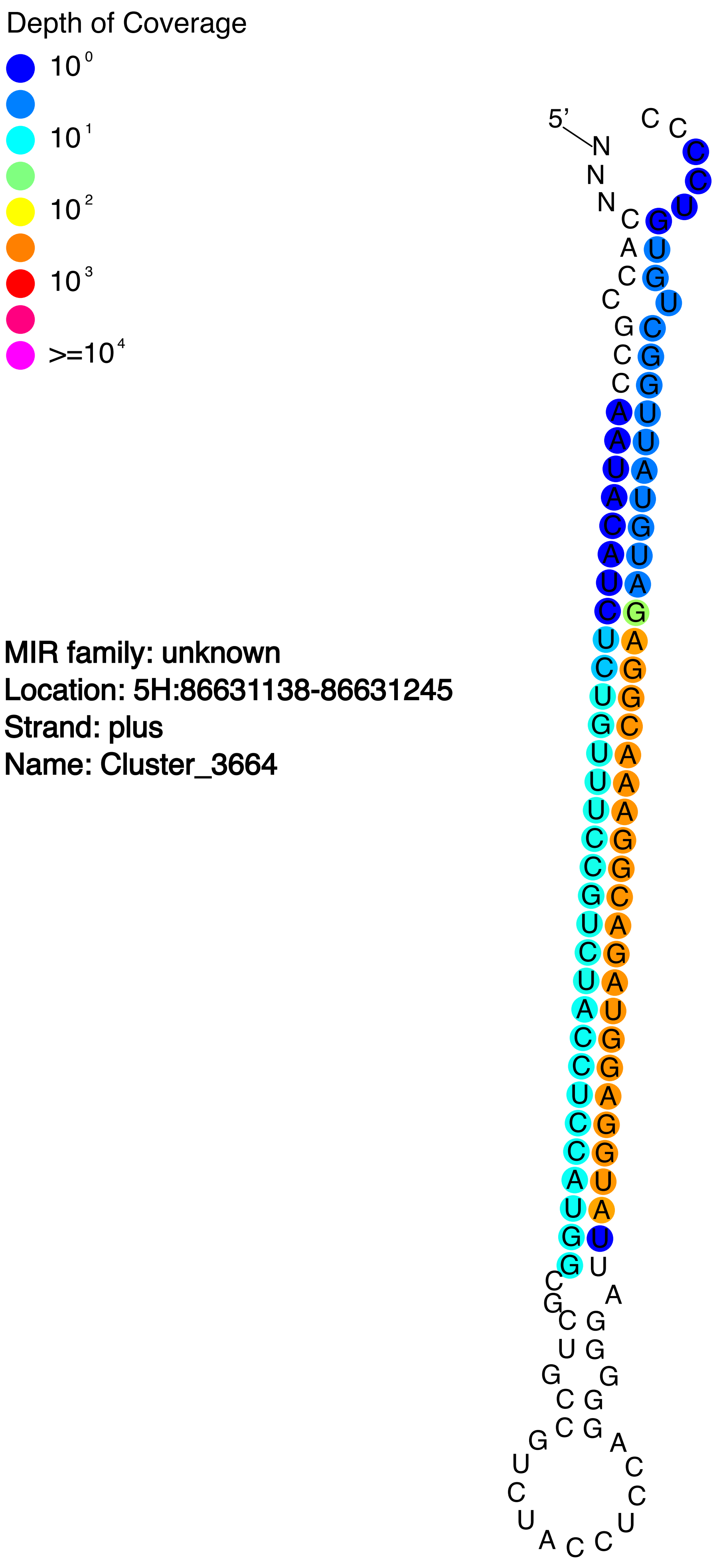

MIR family: unknown
Location: 5H:86631138-86631245
Strand: plus
Name: Cluster_3664

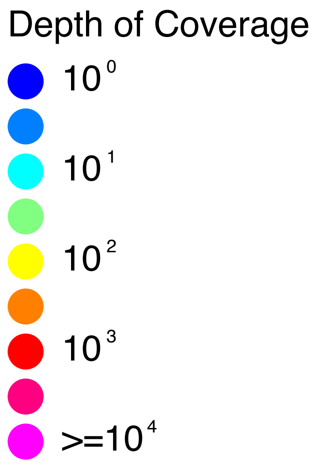

MIR family: unknown
Location: 5H:538351996-538352085
Strand: plus
Name: Cluster_4290

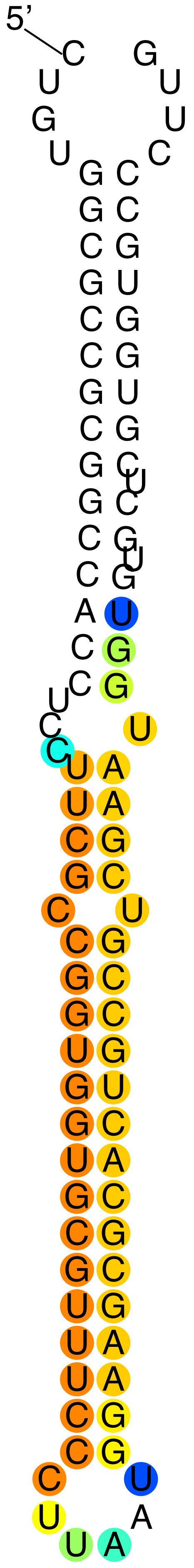

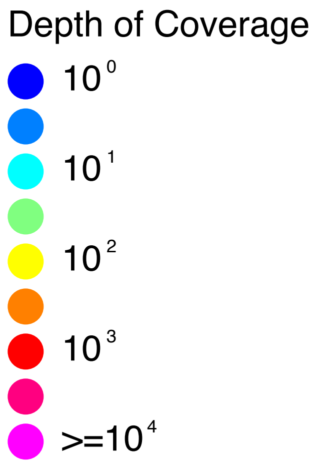

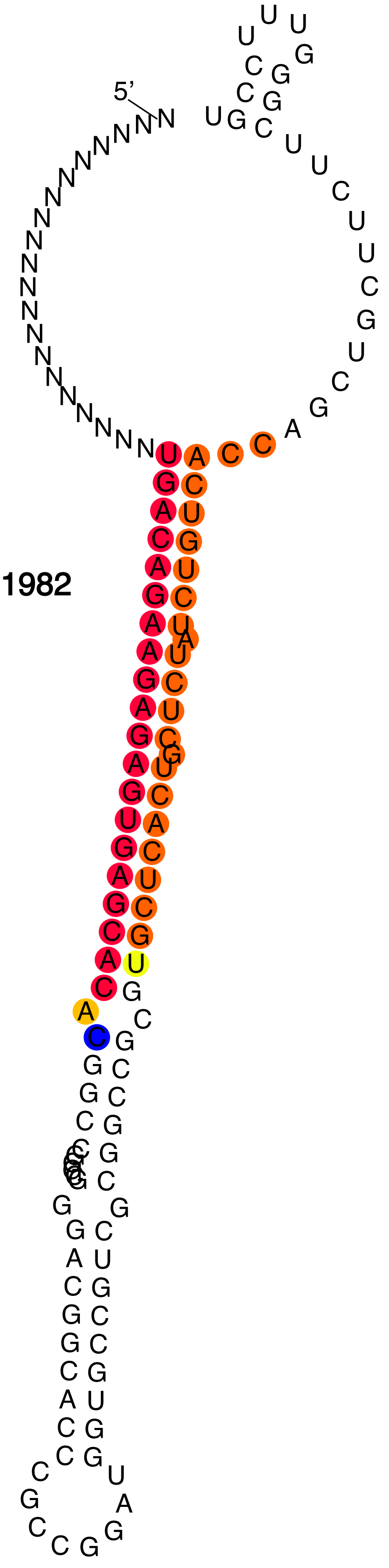

MIR family: miR156
Location: 2H:547101851-547101982
Strand: minus
Name: Cluster_1557

Depth of Coverage

- 10^0
-
- 10^1
-
- 10^2
-
- 10^3
-
- $>=10^4$

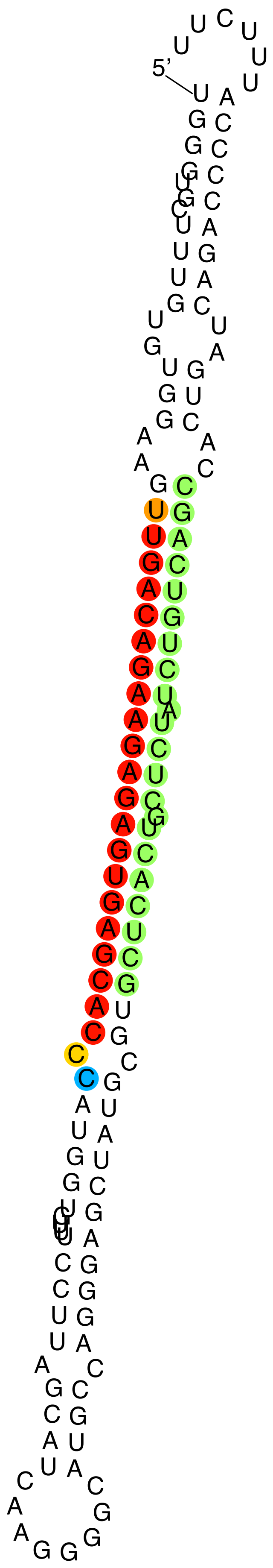

MIR family: miR156
Location: 3H:49404011-49404141
Strand: minus
Name: Cluster_1973

Depth of Coverage

- 10^0
- 10^1
- 10^2
- 10^3
- $>=10^4$

MIR family: miR156
Location: 6H:355385244-355385372
Strand: plus
Name: Cluster_4963

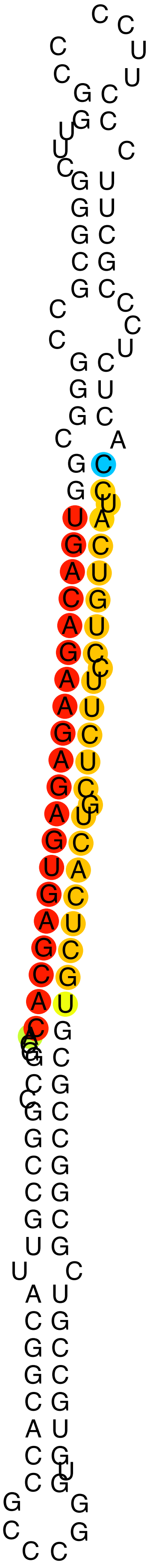

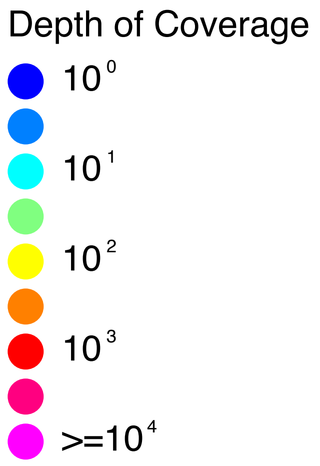

MIR family: miR159
Location: 3H:11949359-11949572
Strand: minus
Name: Cluster_1875

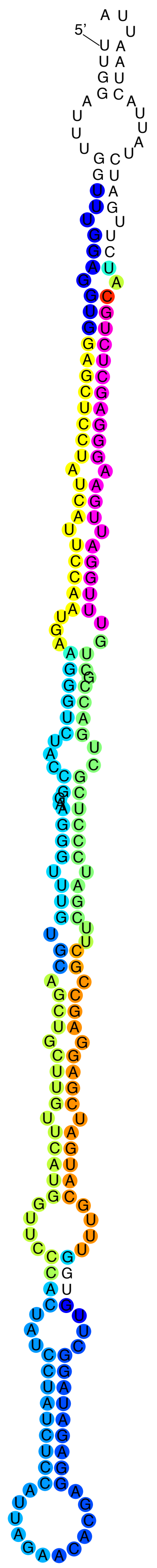

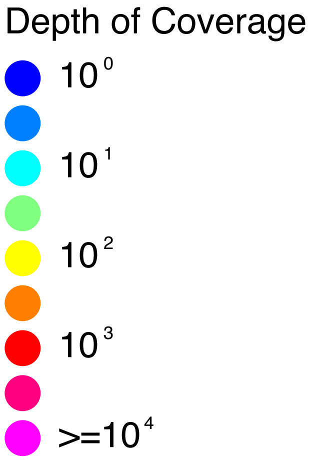

MIR family: miR160
Location: 7H:592808125-592808250
Strand: plus
Name: Cluster_6224

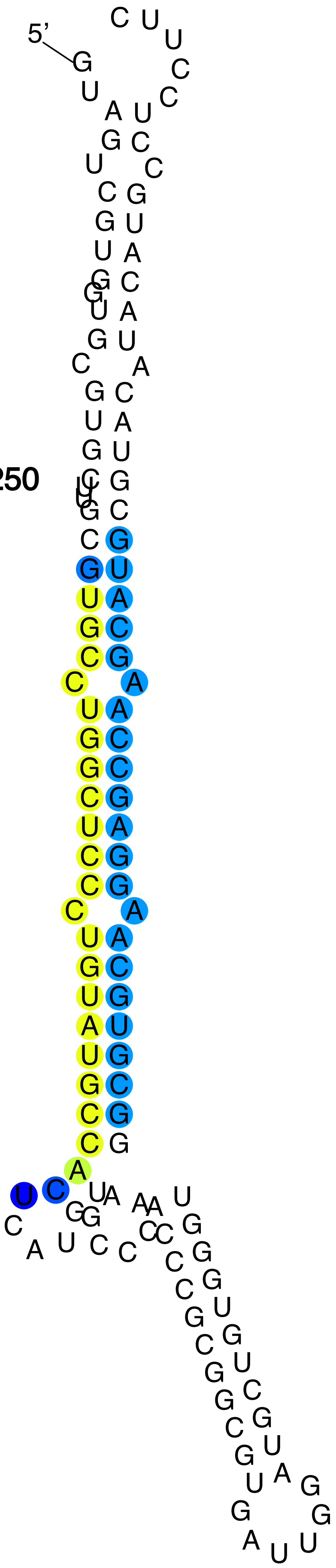

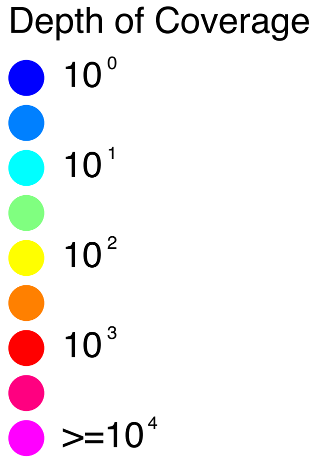

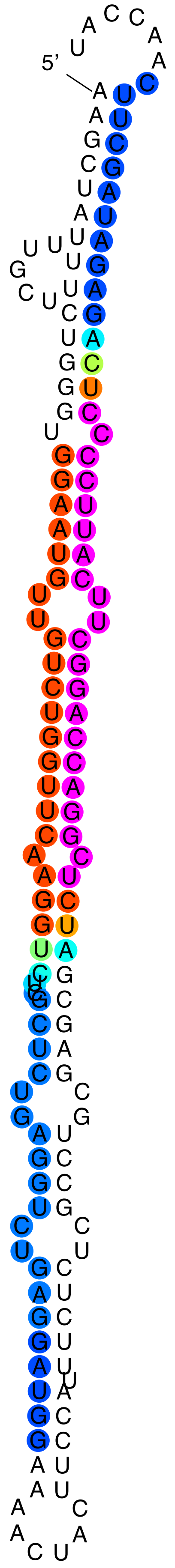

MIR family: miR166
Location: 1H:335384361-335384501
Strand: plus
Name: Cluster_426

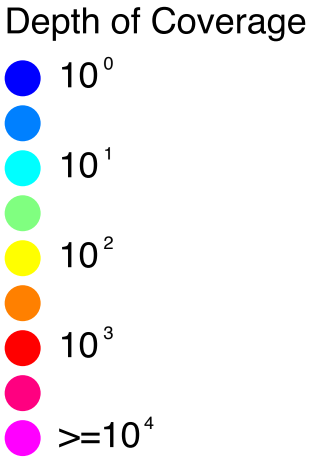

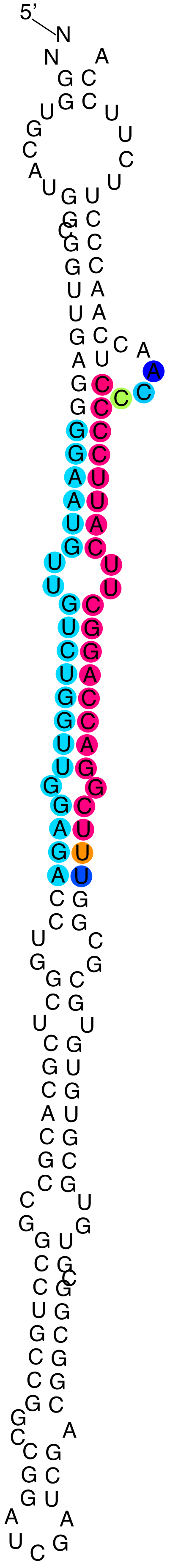

MIR family: miR166
Location: 4H:561565175-561565321
Strand: plus
Name: Cluster_3396

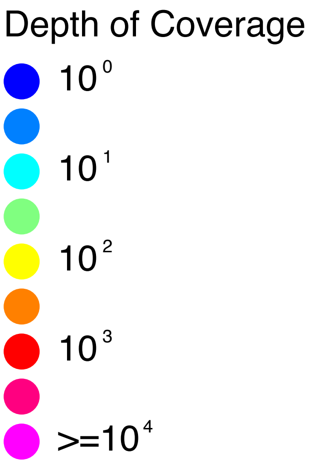

MIR family: miR166
Location: 5H:457063999-457064137
Strand: plus
Name: Cluster_4051

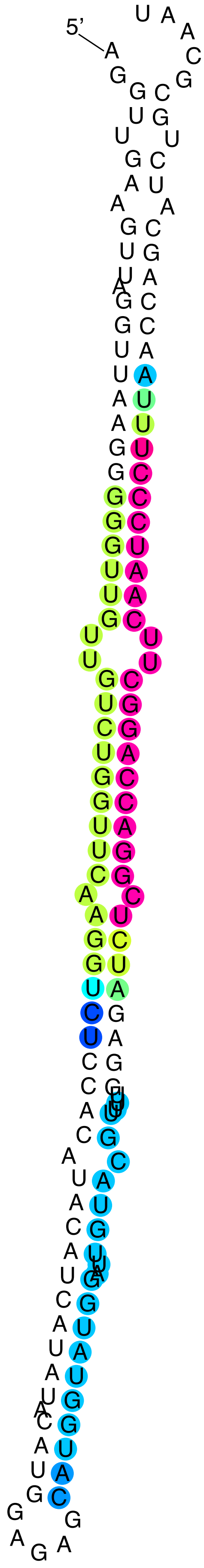

Depth of Coverage

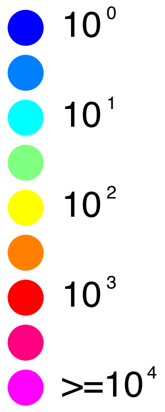

MIR family: miR167
Location: 4H:559503416-559503565
Strand: plus
Name: Cluster_3392

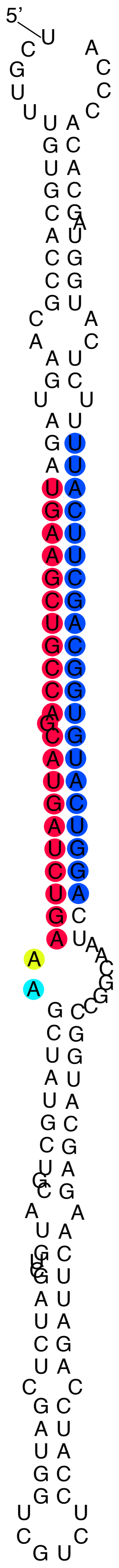

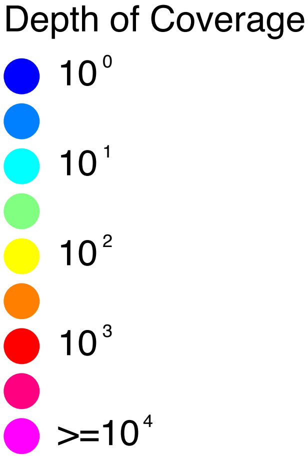

MIR family: miR167
Location: 5H:525224887-525225018
Strand: minus
Name: Cluster_4244

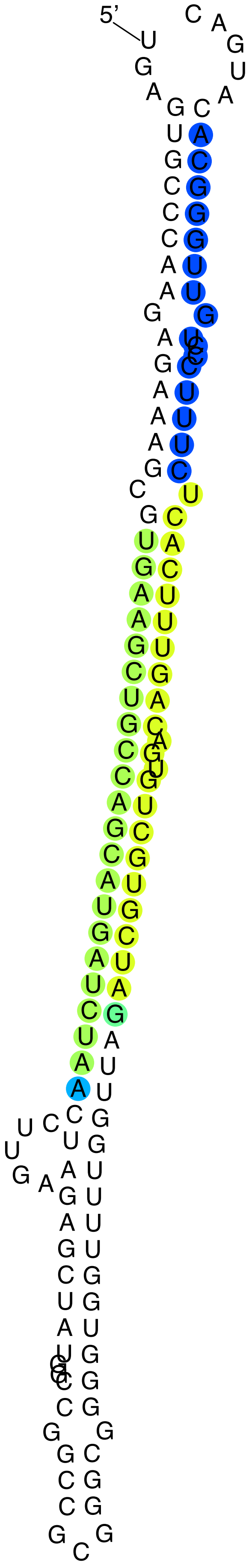

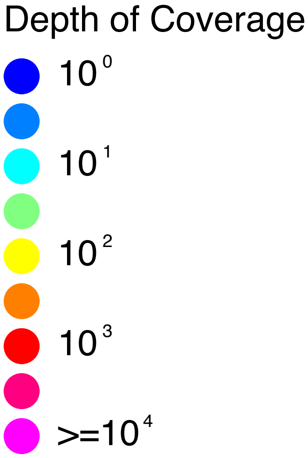

MIR family: miR167
Location: 5H:42176655-42176771
Strand: minus
Name: Cluster_3624

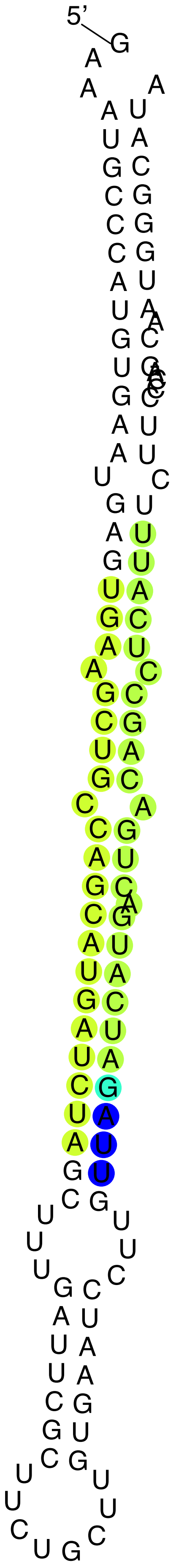

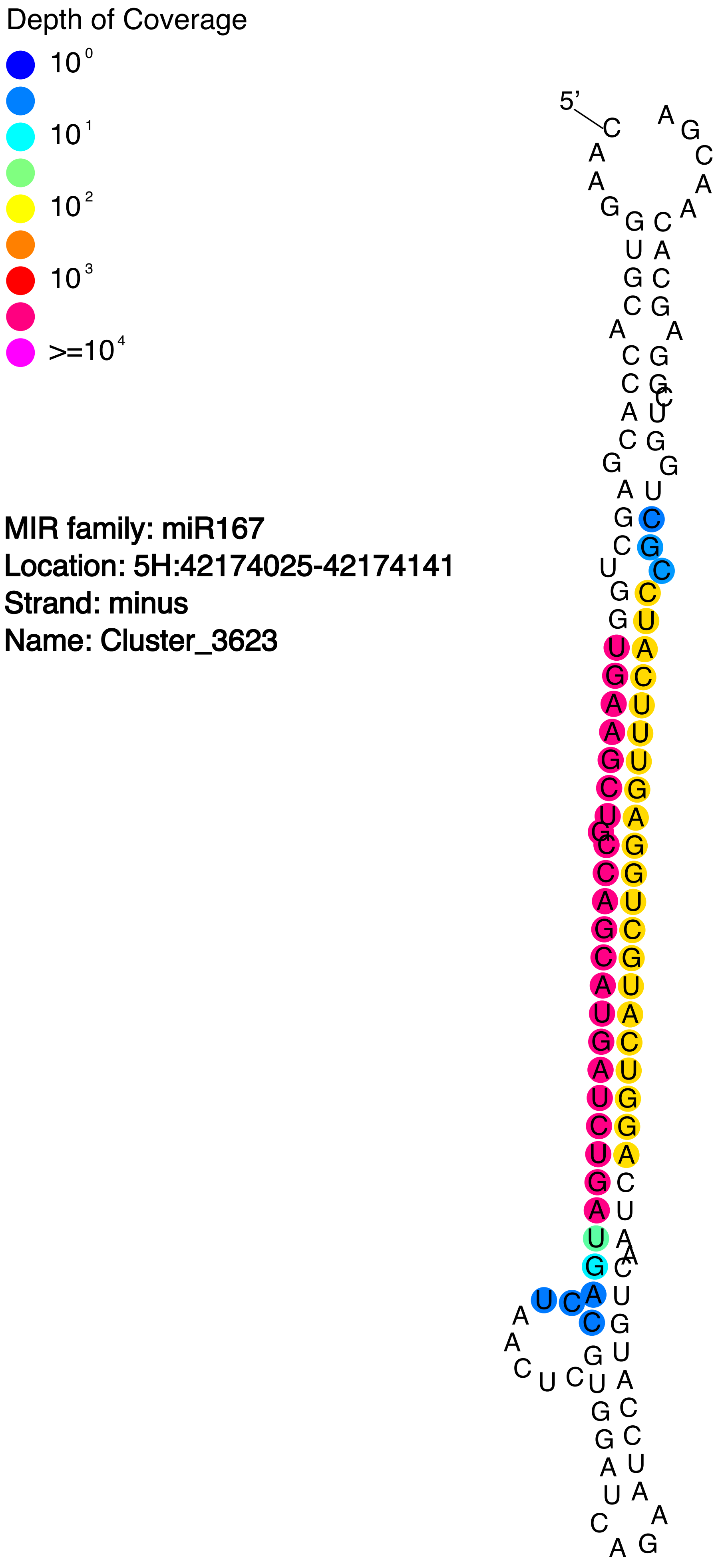

Depth of Coverage
10^0
10^1
10^2
10^3
>=10^4
5'
MIR family: miR167
Location: 5H:42174025-42174141
Strand: minus
Name: Cluster_3623

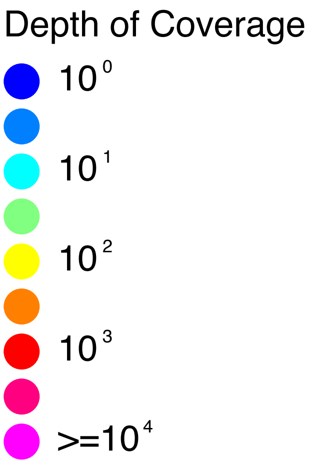

MIR family: miR167
Location: 6H:138383247-138383393
Strand: minus
Name: Cluster_4715

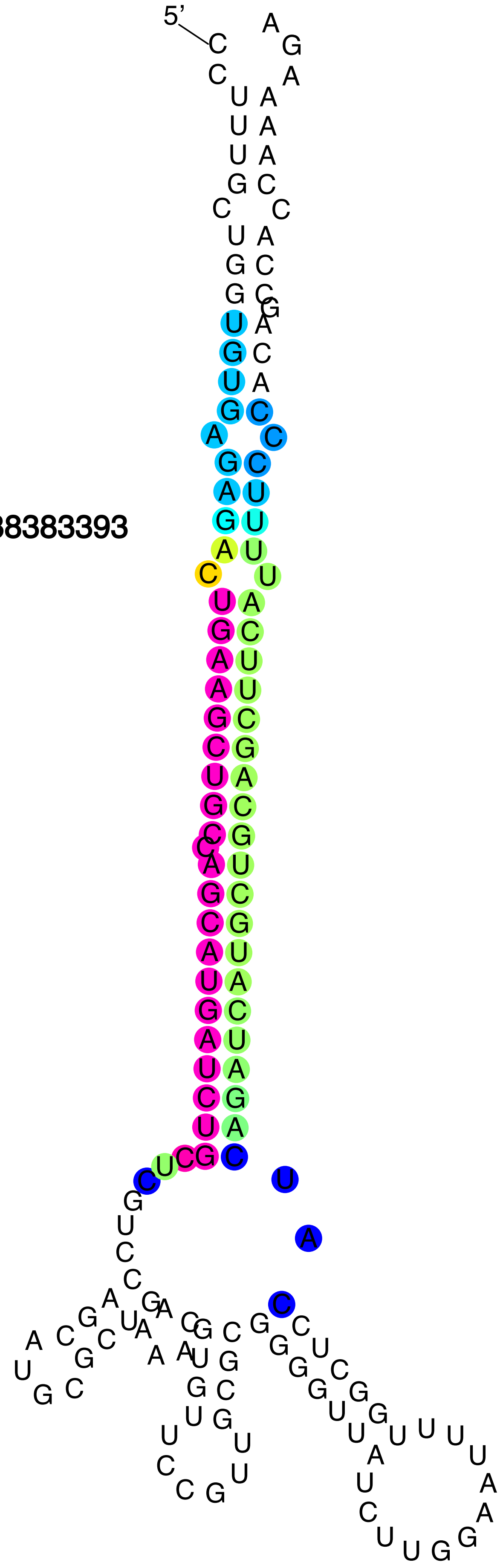

Depth of Coverage

- 10^0
-
- 10^1
-
- 10^2
-
- 10^3
-
- $>=10^4$

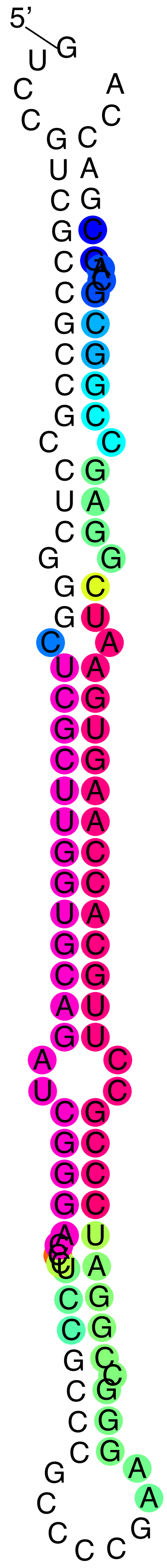

MIR family: miR168

Location: 6H:34273952-34274061

Strand: plus

Name: Cluster_4564

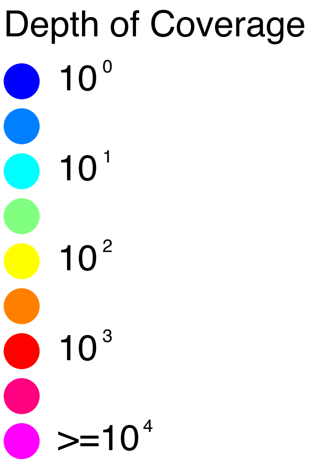

MIR family: miR171
Location: 1H:367870096-367870239
Strand: plus
Name: Cluster_456

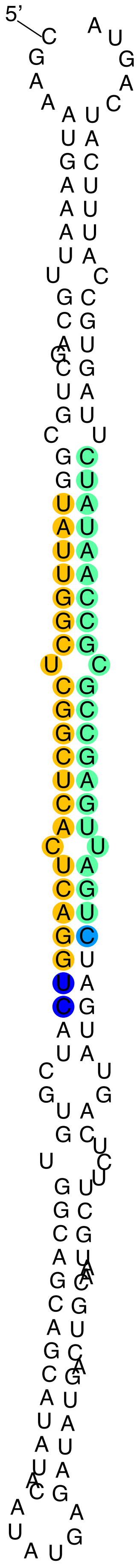

Depth of Coverage

- 10^0
- 10^1
- 10^2
- 10^3
- $>=10^4$

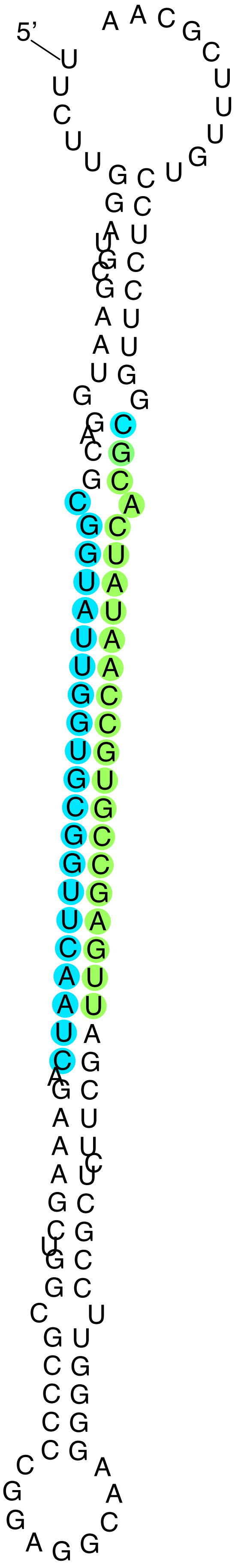

MIR family: miR171
Location: 2H:610738330-610738453
Strand: plus
Name: Cluster_1682

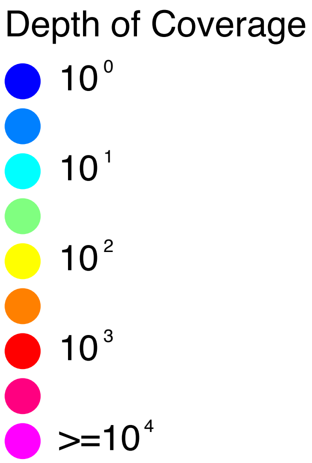

MIR family: miR171
Location: 4H:584947356-584947473
Strand: minus
Name: Cluster_3461

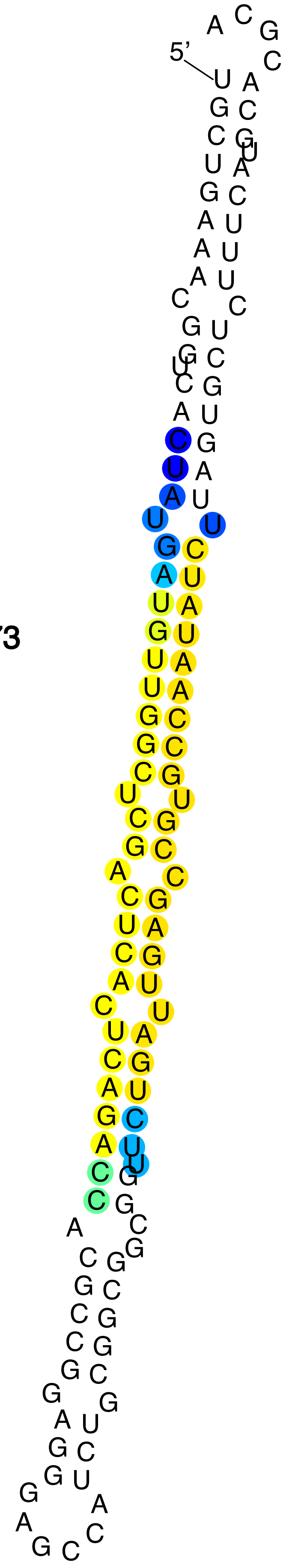

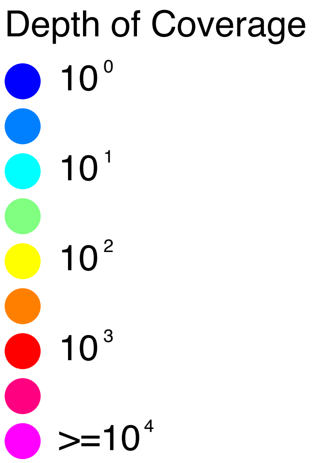

MIR family: miR319
Location: 3H:121156063-121156278
Strand: plus
Name: Cluster_2046

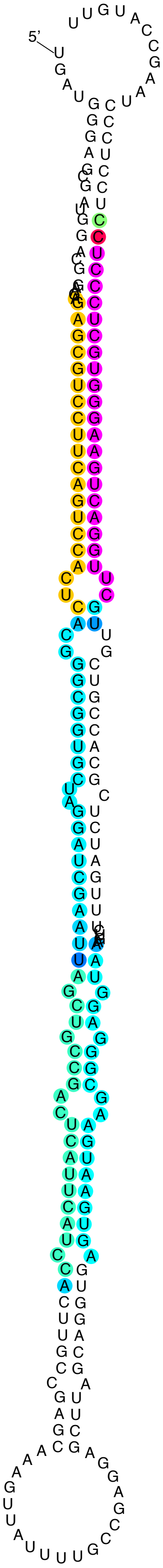

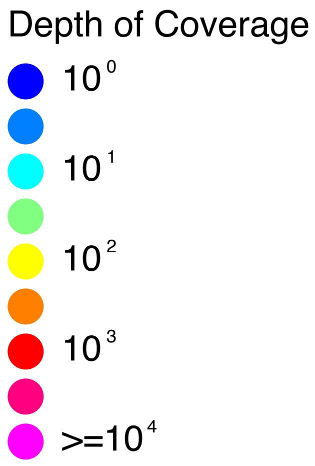

MIR family: miR393
Location: 2H:655580443-655580564
Strand: plus
Name: Cluster_1822

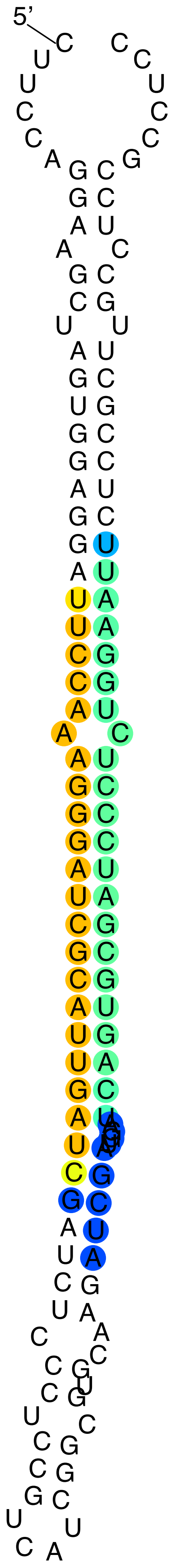

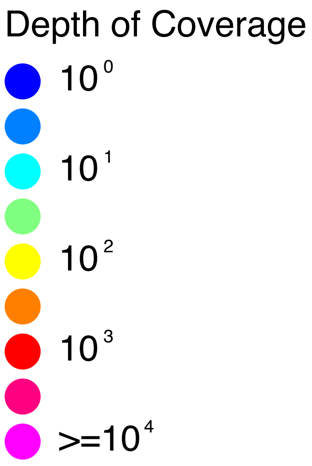

MIR family: miR396
Location: 2H:648439735-648439874
Strand: plus
Name: Cluster_1803

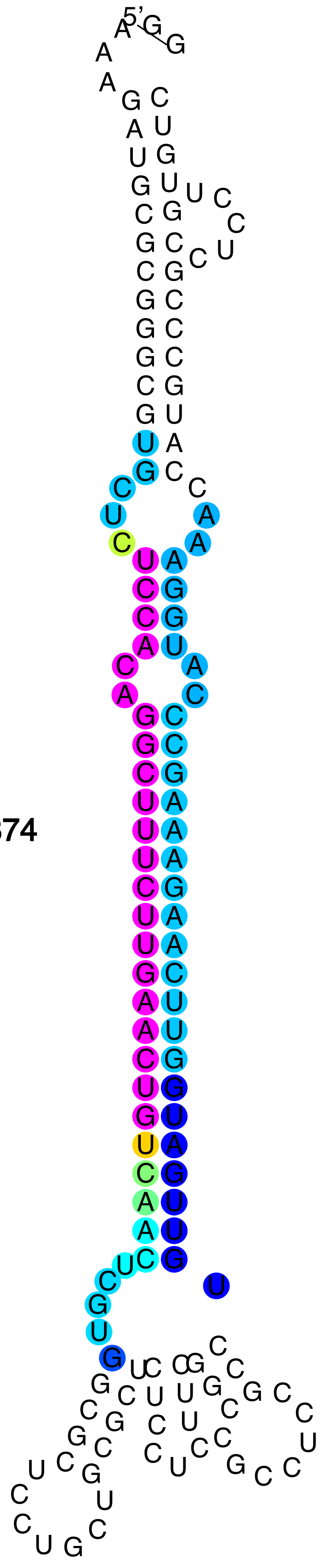

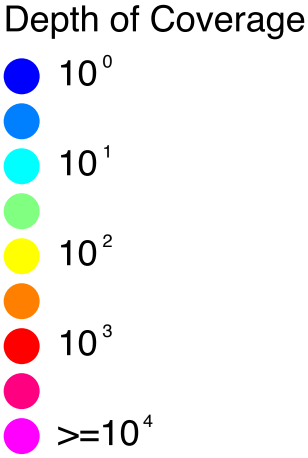

MIR family: miR396
Location: 7H:50567509-50567636
Strand: plus
Name: Cluster_5480

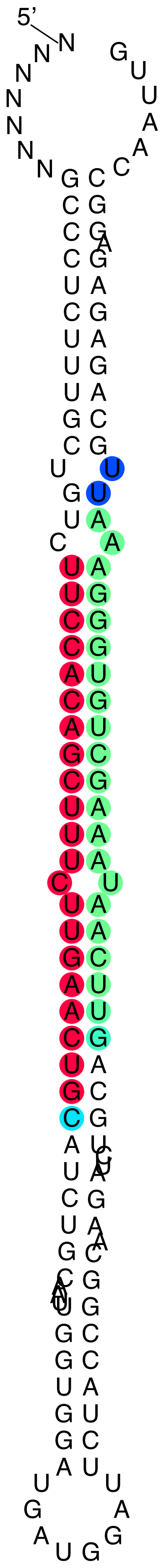

Depth of Coverage

- 10^0
-
- 10^1
-
- 10^2
-
- 10^3
-
- $>=10^4$

MIR family: miR827
Location: 2H:544572793-544572972
Strand: minus
Name: Cluster_1545

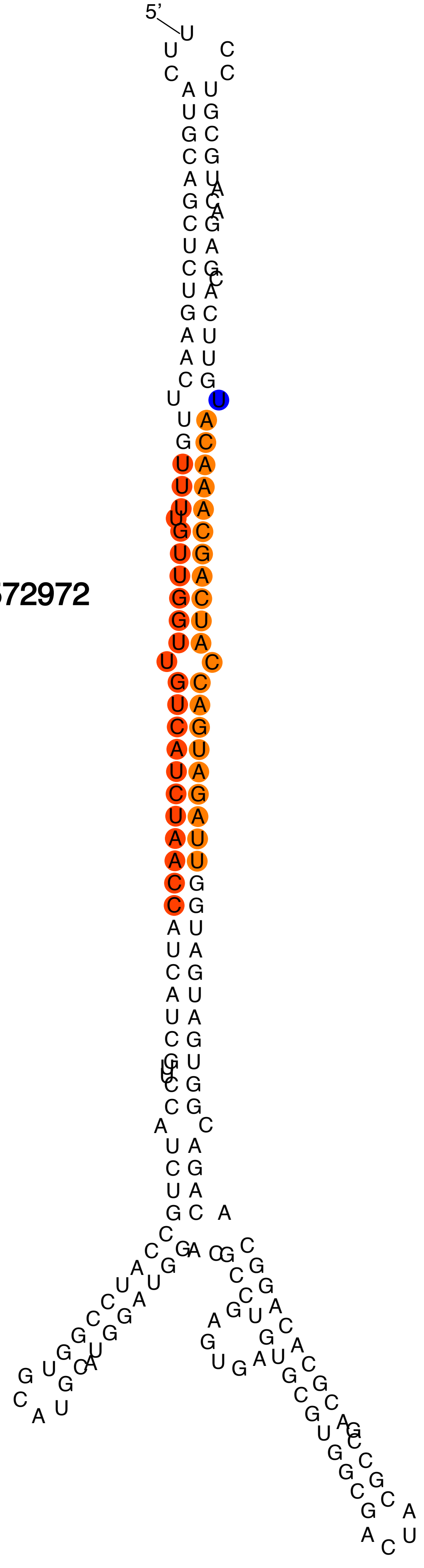

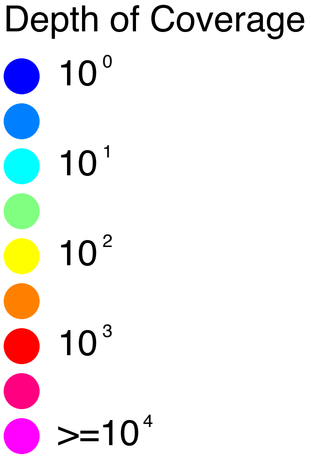

MIR family: miR5051
Location: 4H:558076938-558077060
Strand: plus
Name: Cluster_3388

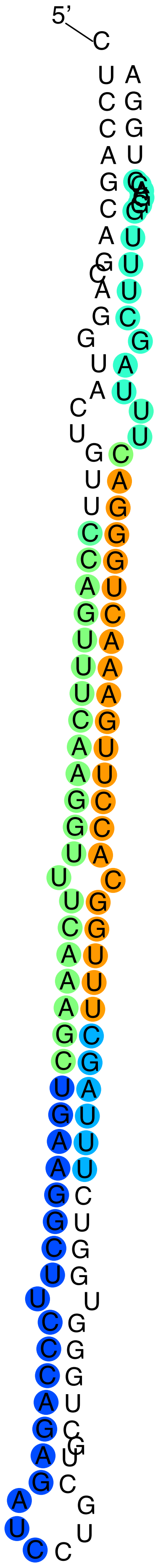

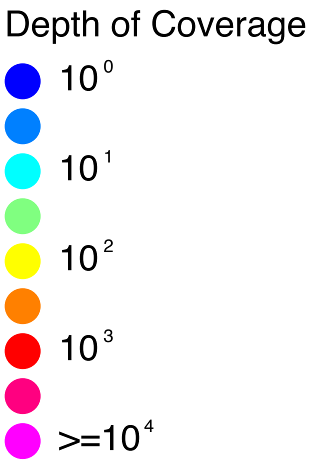

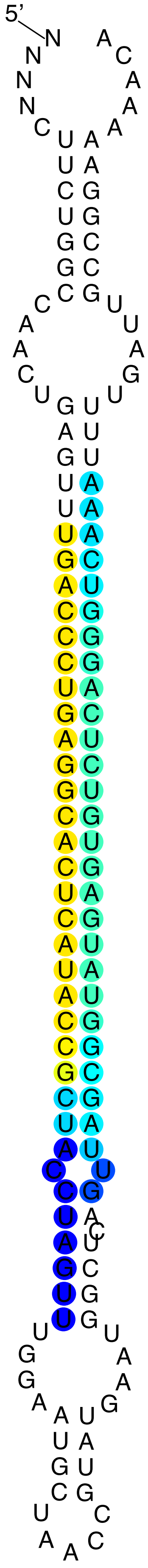

MIR family: miR6201
Location: 6H:557666865-557666994
Strand: plus
Name: Cluster_5317

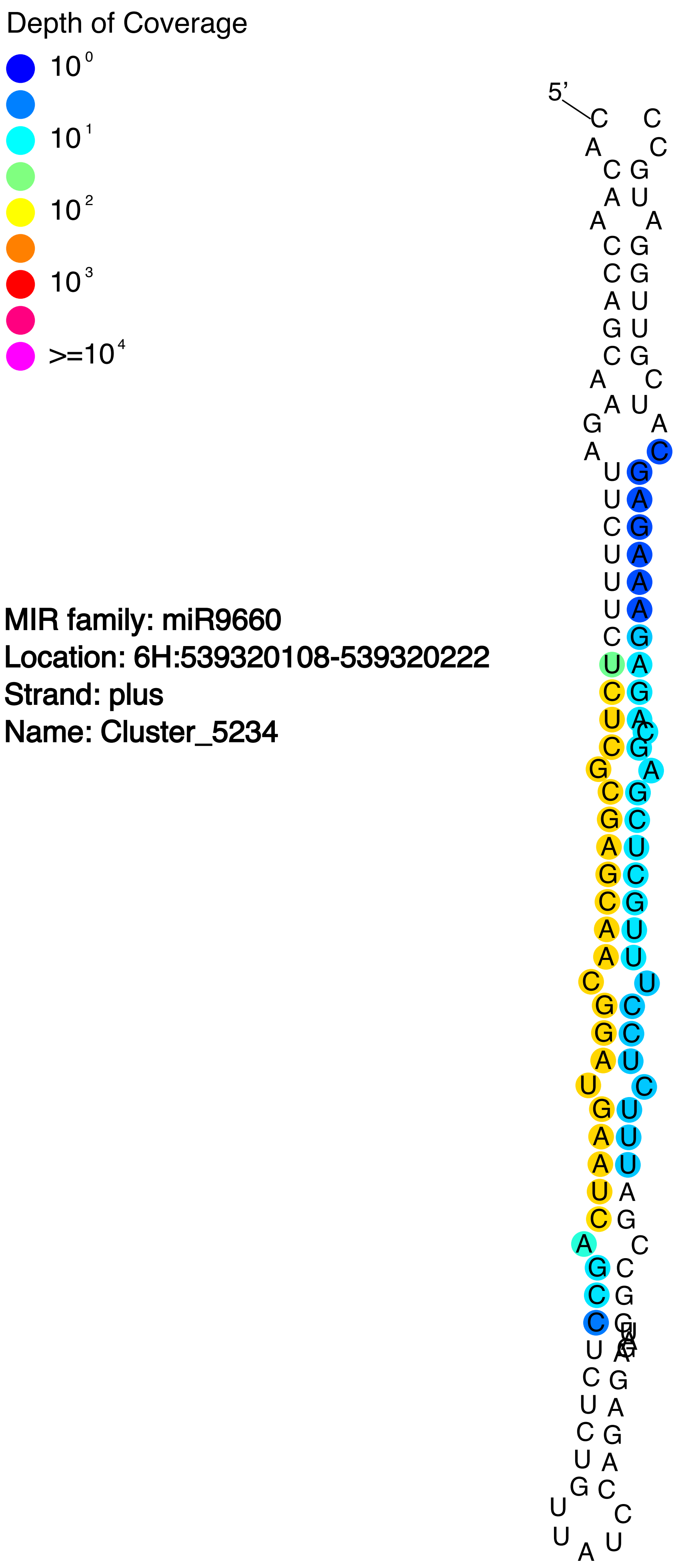

MIR family: miR9660
Location: 6H:539320108-539320222
Strand: plus
Name: Cluster_5234

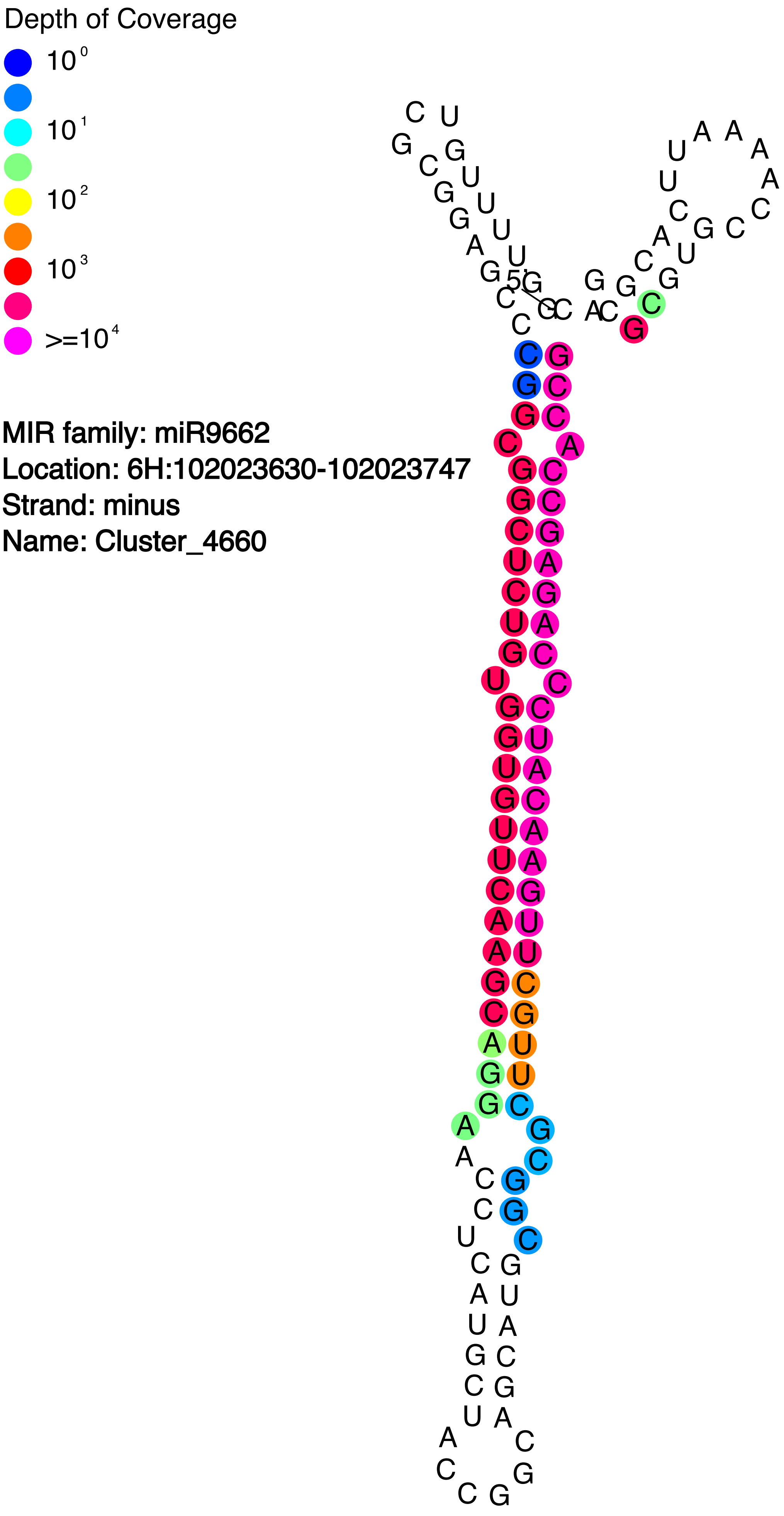

MIR family: miR9662
Location: 6H:102023630-102023747
Strand: minus
Name: Cluster_4660

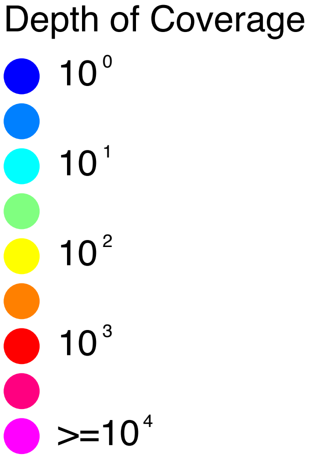

MIR family: miR9674
Location: 3H:567840082-567840213
Strand: plus
Name: Cluster_2558

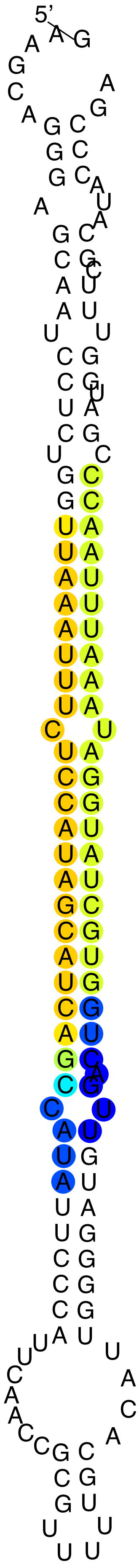

Supplement: Supplementary file 1 [file genes-17-00676-s001.zip › supplemental_figures1-6_tables1-4_20250513/FigureS1_hairpins-predMIR_shortstack-out-strucVis.pdf]
