# Supplementary material for: Post-Transcriptional Gene Regulation by MicroRNAs During Barley Malting
Source: Genes (Basel). 2026 Jun 9;17(6):676. doi: 10.3390/genes17060676 (PMC13299409; doi:10.3390/genes17060676)
Supplement: Supplementary file 1 [file genes-17-00676-s001.zip › supplemental_figures1-6_tables1-4_20250513/FigureS2-S4_known-miRNA_miRNA-features_GSTAr-alignments.pdf]

Figure S2

Sequences of the 249 distinct previously-known mature miRNAs used in this study to seed searches for genomic regions that met expression- and secondary structure-based requirements for MIR loci. The set of 249 consists of 189 HC miRNAs and 60 non-HC *Hordeum vulgare* miRNAs.

```
>osa-miR156bcfghj-5p_zma-miR156defgil-5p_aly-miR156abcd-5p
UGACAGAAGAGAGUGAGCAC
>osa-miR156b-3p
GCUCACUCUCUAUCUGUCAGC
>osa-miR156cg-3p
GCUCACUUCUCUCUCUGUCAGC
>osa-miR156fhl-3p_zma-miR156dfg-3p
GCUCACUUCUCUUUCUGUCAGC
>osa-miR156j-3p
GCUCGCUCCUCUUUCUGUCAGC
>osa-miR160abc-5p_zma-miR160bg-5p_atr-miR160
UGCCUGGCUCCCUGUAUGCCA
>osa-miR160ab-3p_zma-miR160bg-3p
GCGUGCAAGGAGCCAAGCAUG
>osa-miR160c-3p
GCGUGCACGGAGCCAAGCAUA
>osa-miR166a-5p_zma-miR166bd-5p
GGAAUGUUGUCUGGUUCAAGG
>hvu-miR166abc_osa-miR166abcd-3p_zma-miR166a-3p_vvi-miR166d_atr-
miR166cd
UCGGACCAGGCUUCAUUCCCC
>osa-miR166b-5p_zma-miR166a-5p
GGAAUGUUGUCUGGCUCGGGG
>osa-miR166c-5p
GGAAUGUUGUCUGGUCCGAG
>osa-miR166d-5p
GGAAUGUUGUCUGGCUCGAGG
>osa-miR167a-5p_zma-miR167c-5p_aly-miR167a-5p
UGAAGCUGCCAGCAUGAUCUA
>osa-miR167a-3p
AUCAUGCAUGACAGCCUCAUUU
>osa-miR396c-5p_zma-miR396ef-5p_aly-miR396b-5p
UUCCACAGCUUUCUUGAACUU
>osa-miR396c-3p_zma-miR396f-3p
GGUCAAGAAAGCUGUGGGAAG
>osa-miR156l-5p
CGACAGAAGAGAGUGAGCAUA
>osa-miR166k-5p
GGUUUGUUGUCUGGCUCGAGG
>osa-miR166kl-3p_zma-miR166jkn-3p
UCGGACCAGGCUUCAAUCCCU
>osa-miR166l-5p
GGAUUGUUGUCUGGUUCAAGG
```

>osa-miR167egi-5p_zma-miR167ehij-5p
UGAAGCUGCCAGCAUGAUCUG
>osa-miR167ei-3p
AGAUCAUGUUGCAGCUUCACU
>hvu-miR168-5p_osa-miR168a-5p_zma-miR168b-5p
UCGCUUGGUGCAGAUCGGGAC
>hvu-miR168-3p_osa-miR168a-3p
GAUCCCGCCUUGCACCAAGUGAAU
>osa-miR169i-5p.2
UGGUGAUAAGGGUGUAGCUCUG
>osa-miR169i-5p.1_zma-miR169j-5p
UAGCCAAGGAUGACUUGCCUG
>osa-miR169i-3p
UGAGUCGCUCUUAUCACUCAUG
>osa-miR171c-5p
GGAUAUUGGUGCGGUUCAAUC
>hvu-miR171-3p_osa-miR171cde-3p_zma-miR171de-3p_tae-miR171a
UGAUUGAGCCGUGCCAAUAUC
>osa-miR171d-5p
UGUUGGCCCGGCUCACUCAGA
>osa-miR171e-5p_zma-miR171de-5p
UGUUGGCUCGGCUCACUCAGA
>osa-miR166h-5p_zma-miR166m-5p
GGAAUGUUGGCUGGCUCGAGG
>osa-miR166h-3p_zma-miR166lm-3p
UCGGACCAGGCUUCAUUCCUC
>osa-miR393b-5p
UCCAAAGGGAUCGCAUUGAUCU
>osa-miR393b-3p
UCAGUGCAAUCCCUUUGGAAU
>osa-miR408-5p
CAGGGAUGAGGCAGAGCAUGG
>osa-miR408-3p
CUGCACUGCCUCUUCCCUGGC
>osa-miR172d-5p
GCAGCACCAUCAAGAUUCAC
>osa-miR172d-3p_aly-miR172a-3p
AGAAUCUUGAUGAUGCUGCAU
>osa-miR171i-5p
AGGUAUUGGCGUGCCUCAAUC
>osa-miR171i-3p_zma-miR171l-3p
GGAUUGAGCCGCGUCAAUAUC
>zma-miR156e-3p
GCUCACUGCUCUCUCUGUCAUC
>zma-miR156i-3p
GCUCACUGCUCUAUCUGUCAUC
>zma-miR167c-3p
GAUCAUGCUGUGGCAGCCUCACU
>zma-miR166i-5p

GGAAUGUCGUCUGGCGCGAGA
>zma-miR166bdgi-3p
UCGGACCAGGCUUCAUUCCC
>zma-miR166g-5p
GGAAUGUUGUCUGGUUGGAGA
>zma-miR171b-5p
GAUAUUGGCGCGGUUCAAUC
>zma-miR171b-3p
UUGAGCCGUGCCAAUAUCAC
>zma-miR172c-5p
CAGCACCACCAAGAUUCACA
>zma-miR172c-3p
AGAAUCUUGAUGAUGCUGCA
>osa-miR390-5p_zma-miR390ab-5p
AAGCUCAGGAGGGAUAGCGCC
>osa-miR390-3p
CGCUAUCUAUCCUGAGCUCC
>osa-miR396e-5p
UCCACAGGCUUUCUUGAACUG
>osa-miR396e-3p
AUGGUUCAAGAAAGCCCAUGGAAA
>osa-miR444a-5p
GCUAGAGGUGGCAACUGCAUA
>osa-miR444a-3p.2
UGCAGUUGCUGCCUCAAGCUU
>hvu-miR444a_osa-miR444a-3p.1
UUGCUGCCUCAAGCUUGCUGC
>zma-miR399b-5p
GUGCAGCUCUCCUCUGGCAUG
>zma-miR399b-3p
UGCCAAAGGAGAGCUGUCCUG
>zma-miR399d-5p
GUGUGGCUCUCCUCUGGCAUG
>zma-miR399d-3p
UGCCAAAGGAGAGCUGCCCUG
>zma-miR399f-5p
GGGCAACUUCUCCUUUGGCAGA
>zma-miR399f-3p
UGCCAAAGGAAAUUUGCCCCG
>zma-miR159a-5p
GAGCUCCUAUCAUUCCAAUGA
>hvu-miR159ab_zma-miR159af-3p_tae-miR159a
UUUGGAUUGAAGGGAGCUCUG
>zma-miR319b-5p
AGAGCGUCCUUCAGUCCACUC
>zma-miR319b-3p
UUGGACUGAAGGGUGCUCCC
>zma-miR166k-5p
GGAUUGUUGUCUGGCUCGGGG

>zma-miR166j-5p
GGUUUGUUUGUCUGGUUCAAGG
>zma-miR167e-3p
GAUCAUGCUGUGCAGUUUCAUC
>zma-miR167hi-3p
GAUCAUGUUGCAGCUUCAC
>zma-miR168b-3p
CCCGCCUUGCAUCAAGUGAA
>zma-miR169j-3p
GGCAGUCUCCUUGGCUAG
>zma-miR166l-5p
GAAUGGAGGCUGGUCCAAGA
>vvi-miR169e
UAGCCAAGGAUGACUUGCCUGC
>osa-miR1425-5p
UAGGAUUCAAUCCUUGCUGCU
>osa-miR1425-3p
CAGCAAGAACUGGAUCUUAAU
>osa-miR1429-5p
GUAAUAUACUAAUCCGUGCAU
>osa-miR1429-3p
GUUGCACGGGUUUGUAUGUUG
>osa-miR169r-5p
UAGCCAAGGAUGAUUUGCCUG
>osa-miR169r-3p
UGGCAAGUCUCCUCGGCUACC
>osa-miR396f-5p
UCUCCACAGGCUUUCUUGAACU
>osa-miR396f-3p
AUAGUUCAAGAAAGUCCUUGGAAA
>zma-miR156l-3p
GCUCACUGCUCUAUCUGUCACC
>zma-miR159f-5p
GAGCUCCUCUCAUUCCAAUGA
>zma-miR166n-5p
GGAUUGUUGUCUGGCUCGGUG
>zma-miR167j-3p
GAUCAUGUGGCAGUUUCAUU
>zma-miR171l-5p
UAUUGGCGUGCCUCAAUCCGA
>zma-miR390ab-3p
CGCUAUCUAUCCUGAGCUCCA
>zma-miR396e-3p
GGUCAAGAAAGCCGUGGGAAG
>zma-miR399h-5p
GUGCAGUUCUCCUCUGGCACG
>zma-miR399h-3p
UGCCAAAGGAGAAUUGCCCUG
>zma-miR399i-5p

GUGCGGCUCUCCUCUGGCAUG
>zma-miR399i-3p
UGCCAAAGGAGAGUUGCCCUG
>zma-miR529-5p
AGAAGAGAGAGAGUACAGCCU
>zma-miR529-3p
GCUGUACCCUCUCUCUUCUUC
>aly-miR156a-3p
GCUCACUGCUCUUUCUGUCAGA
>aly-miR156b-3p
GCUCACCUCUCUUUCUGUCAGU
>aly-miR156c-3p
GCUCACUGCUCUAUCUGUCAGA
>aly-miR156d-3p
GCUCACUCUCUUUCUGUCAUA
>aly-miR158a-5p
CUUUGUCUACAAUUUUGGAAA
>aly-miR158a-3p
UCCCAAAUGUAGACAAAGCA
>aly-miR159a-5p
GAGCUCCUUGAAGUUCAAACG
>aly-miR159a-3p
UUUGGAUUGAAGGGAGCUCUA
>aly-miR159b-5p
GAGCUCCUUGAAGUUCAAUGG
>aly-miR159b-3p
UUUGGAUUGAAGGGAGCUCUU
>aly-miR167a-3p
GAUCAUGUUCGCAGUUUCACC
>aly-miR168a-5p_atr-miR168
UCGCUUGGUGCAGGUCGGGAA
>aly-miR168a-3p
CCCGCCUUGCAUCAACUGAAU
>aly-miR171b-5p
AGAUAUUAGUGCGGUUCAAUC
>aly-miR171bc-3p
UUGAGCCGUGCCAAUAUCACG
>aly-miR171c-5p
AGAUAUUGGUGCGGUUCAAUC
>aly-miR172a-5p
GUGGCAUCAUCAAGAUUCACA
>aly-miR173ab-5p
UUCGCUUGCAGAGAGAAAUCAC
>aly-miR173ab-3p
GAUUCUCUGUGCAAGUGGAAG
>aly-miR396a-5p
UUCCACAGCUUUCUUGAACUG
>aly-miR396a-3p
GUUCAAUAAAGCUGUGGGAAG

>aly-miR396b-3p
GCUCAAGAAAGCUGUGGGAAA
>aly-miR824-5p
UAGACCAUUUGUGAGAAGGGA
>aly-miR824-3p
CCUUCUCAUCGAUGGUCUAGA
>vvi-miR2111-5p
UAAUCUGCAUCCUGAGGUCUA
>vvi-miR2111-3p
GUCCUCUGGUUGCAGAUUACU
>vvi-miR3623-5p
UCACAAGUUCAUCCAAGCACCA
>vvi-miR3623-3p
UGGUGCUUGGACGAAUUUGCUA
>vvi-miR2950-5p
UUCCAUCUCUUGCACACUGGA
>vvi-miR2950-3p
UGGUGUGCACGGGAUGGAAUA
>vvi-miR3632-5p
GGAUUGGGGGCCGAUGGAAAGG
>vvi-miR3632-3p
UUUCCCAGACCCCCAAUACCAA
>vvi-miR3633a-5p
GGAAUGGAUGGUUAGGAGAG
>vvi-miR3633a-3p
UUCCUAUACCACCCAUUCCCUA
>vvi-miR3633b-5p
GGAAUGGGUGGCUGGGAUCUA
>vvi-miR3633b-3p
GUUCCCAUGCCAUCCAUUCCUA
>vvi-miR3636-5p
UCGGUUUGCUUCUUUGAUAGAUUC
>vvi-miR3636-3p
GUCUGUCGGAGAAGCAAGUCGGAG
>vvi-miR3640-5p
ACCUGAUUGGUGAUGCUUUUUUGG
>vvi-miR3640-3p
AUCGAAAAGGCAUCAUCAAUCAGG
>tae-miR398
UGUGUUCUCAGGUCGCCCCCG
>osa-miR3979-5p
UCUCUCUCUCCCUUGAAGGC
>osa-miR3979-3p
CUUCGGGGGAGGAGAGAAGC
>osa-miR3980ab-5p
AAUCGACGGCCUCAGUCAGGG
>osa-miR3980ab-3p
CUGGCCGAGGCCGUCGAUUCU
>osa-miR5150-5p

AGCUUCUGACAGCUGCAGUUUCUC
>osa-miR5150-3p
AGAAGCUGCAGCUGUCAGAAGCUC
>atr-miR8551
AUGUUCUAGGUUAGCUCUUGGAUG
>atr-miR8553a
AUCGGGUGGUUCAGAAUUCAUAUC
>atr-miR8558a
UUUCCGAAUCCGCCUAUACCUG
>atr-miR8559
UGACACUGUAGUAGUCAACCCGUG
>atr-miR8570
AGUUGGGAGUGCGUCAUUGACUAG
>atr-miR8580
UGUCUAAAUAGAAUGGAUGGUGUA
>atr-miR8582
UAGGAGUCAUGACGUAGCUUG
>atr-miR8585
UCACAGGAGAGAUGAUACUGGU
>atr-miR8586
UUUUCUCUUAACAACAGAGGA
>atr-miR8587
UCCAACUUUUGAACUGCCCAAAAU
>atr-miR8602
UCAGGAGAGAUGAUGCCGGCC
>atr-miR8605
CACAUUAAUCUCGACCAUUGGAUG
>atr-miR2111
UAAUCUGUAUCUUGAGGUUUGG
>atr-miR8610.1
ACCUUCUUCGGUUUGUUCAGAAAA
>atr-miR8610.2
UUCUGACCAAAUUGAAGGAGG
>atr-miR8611
UAAGGGCGCUCCGACAACGUG
>atr-miR8614
CAAUAGCUAAAGUGAUUGUGACC
>atr-miR169a
UAGCCAAGGAUGACUUGCCU
>atr-miR169b
AGCAAGUCGCCUUGGCUAACC
>atr-miR171b
UUGAGCCGUGCCAAUAUCACA
>atr-miR171c
UGAUUGAGCCGCGCCAAUAUC
>atr-miR172
GGAAUCUUGAUGAUGCUGCA
>atr-miR398
UGUGUUCCCAGGUCGCCCCUG

>atr-miR535
UGACAACGAGAGAGAGCACGC
>atr-miR8558b
UUUCCGAUCCCGCCCAUGCCGU
>tae-miR9652-5p
CCUGUUUGUCAUUAAGUUUCUU
>tae-miR9652-3p
AAGCUUAAUGAGAACAUGUG
>tae-miR9655-3p
CAAGGGAAGGAAGUAGCCAAC
>tae-miR1137b-5p
UCCGUUCCAGAAUAGAUGACC
>tae-miR9666a-3p
CGGUAGGGCUGUAUGAUGGCGA
>tae-miR5062-5p
UGAACCUUAGGGAACAGCCGCAU
>tae-miR9666bc-5p
GCCAUCAUACGUCCAACCGUG
>tae-miR9670-3p
AGGUGGAAUACUUGAAGAAGA
>tae-miR167c-5p
UGAAGCUGCCAGCAUGAUCUGC
>tae-miR397-5p
UCACCGGCGCUGCACACAAUG
>tae-miR5384-3p
UGAGCGCGCCGCCGUCGAAUG
>tae-miR5048-5p
UUUGCAGGUUUUAGGUCUAAGU
>tae-miR6197-5p
UCUGUAAACAAAUGUAGGACG
>tae-miR9657b-5p
UUCGUCGGAGAAGCAUGUUGC
>tae-miR9657b-3p
CGUGCUUCCUCGUCGAACGGU
>tae-miR9666b-3p
CGGUUGGGCUGUAUGAUGGCGA
>tae-miR9772
UGAGAUGAGAUUACCCCAUAC
>tae-miR5050
UUGAACGACCUCACCAUGUCG
>tae-miR5200
UGUAGAUACUCCCUAAGGCUU
>hvu-miR156ab
UGACAGAAGAGAGUGAGCACA
>hvu-miR169
AAGCCAAGGAUGAGUUGCCUG
>hvu-miR171-5p
UGUUGGCUCGACUCACUCAGA
>hvu-miR397a

CCGUUGAGUGCAGCGUUGAUG
>hvu-miR1120
ACAUUCUUAUAUUAUGGGACGGAG
>hvu-miR1436
ACAUUAUGGGACGGAGGGAGU
>hvu-miR399
UGCCAAAGGAGAUUUGCCCCG
>hvu-miR444b
UGCAGUUGCUGUCUCAAGCUU
>hvu-miR5048ab
UAUUUGCAGGUUUUAGGUCUAA
>hvu-miR5049a
UCCUAAAUACUUGUUGUUGGG
>hvu-miR5050
UUGAGGUCGUUCAACCAGCAA
>hvu-miR5051
UUUGGCACCUUGAAACUGGGA
>hvu-miR5052
ACCGGCUGGACGGUAGGCAUA
>hvu-miR5053
CGCAGCUGUAGUCGCCGGCGU
>hvu-miR5049b
AGUAUUUAGGUACAGAGGGAG
>hvu-miR1130
UCUGUAACUUAAUAUAAGACG
>hvu-miR6176
GAAGCUGUAGUGCAGCCGGCGUU
>hvu-miR6177
UACCAUGGACAGAAGGCACUUA
>hvu-miR6178
CACGGAACUGGGCGGUUGCACU
>hvu-miR6179
AACCAGUCGAGGCCAGGGGGUU
>hvu-miR5049c
AGACAAUUAUUUUGGGACGGAGG
>hvu-miR6180
AGGGUGGAAGAAAGAGGGCG
>hvu-miR6181
UGCUCUUCAUGGACUGCGGCGCC
>hvu-miR6182
UGAGUGUGUGAUGGAUGGCUUU
>hvu-miR6183
UGAGCGAGUUGGCUGCAAGUUC
>hvu-miR6184
CGGCGUCGGAUCUGGCCGGCCU
>hvu-miR6185
UCUGGCAGCGACGGGAACAUA
>hvu-miR6186
CGAGGAAGGCGCUGAGAGAGA

>hvu-miR6187
UGAACAGGUUCGGCGACCUCA
>hvu-miR6188
GGUGGAUCGAUGAACCCGGCGA
>hvu-miR5049d
UACAAUUAUUUAGGAACGGAG
>hvu-miR6189
AGGUGAUGCUGUGGUGAUCU
>hvu-miR6190
CGAGGAAAGGAAGAAGCCAUG
>hvu-miR6191
UAGAUUUGUCUAGAUAUGAA
>hvu-miR6192
UAGGAGAGGGGGGAAGGGAUCU
>hvu-miR6193
CUCUGCCACCGGUCCAUGACGAC
>hvu-miR6194
UAUGGGGAUCUGACAGACGAG
>hvu-miR6195
UGAGUACGUAGUAGGGAUGAG
>hvu-miR6196
AGGACGAGGAGAUGGAGAGGA
>hvu-miR6197
UCUGUUCCUAAAUGUAAGACG
>hvu-miR6198
GCUCUGUCUUGGAUGGUCAUUC
>hvu-miR6199
CCACAGAAUUCUCACAGUGAUGG
>hvu-miR5049e
AAUUAUUUAGGUACAGAGGGA
>hvu-miR6200
UUUGGCCAACUAGAUCUAUGA
>hvu-miR6201
UGACCCUGAGGCACUCAUACCG
>hvu-miR5049f
AAUUAAUAUGGAUCGGAGGGA
>hvu-miR6202
UGAAGAUUUUAAGCAUUGAA
>hvu-miR6203
AGACGAUUAAGAAGACCUGCAA
>hvu-miR6204
AGGAGAAUAAUUAGAGCUGUGA
>hvu-miR6205
AGGAUGUUUGGAUACGUUUUAGU
>hvu-miR6206
GGCACACGGGCUGCAGGCAUAG
>hvu-miR6207
UGGACGACCUGGGCGCCGACG
>hvu-miR6208

GUGCAUCAAGAUCGGCUCAUCU
>hvu-miR6209
UGAGAUCAGAAAGAUGUGCG
>hvu-miR6210
ACUCCUUGGUUAUCAACUUCGA
>hvu-miR6211
CAGAUCAAGACGCUCCGGCA
>hvu-miR6212
AUACAGUUUACAAUGCACGAU
>hvu-miR6213
ACAGAUUGCUACAGACUGGUC
>hvu-miR6214
CGACGACGACGAGCACGACA
>hvu-miR397b-3p
AUCAACGCUGCACUCAACGGC

Figure S3
Sequences of the -5p and -3p miRNA from the 33 MIR loci putative hairpin structures.

```
>novel-5p.Cluster_68
AAACGAUAUCACCGCCCUACA
>novel-3p.Cluster_68
GAGGGCGGCGAUAACAUUUUC
>miR166-3p.Cluster_426.Cluster_3396
UCGGACCAGGCUUCAUUCCCC
>miR166-5p.Cluster_426
GGAAUGUUGUCUGGUUCAAGG
>miR171-5p.Cluster_456
UAUUGGCUCGGCUCACUCAGG
>miR171-3p.Cluster_456
UGAUUGAGCCGCGCCAAUAUC
>novel-5p.Cluster_1155
ACAAAGAUGUCUGUCAGGUCG
>novel-3p.Cluster_1155
ACCUCACAUGCGUCAUUGUUA
>miR827-5p.Cluster_1545
UUUUGUUGGUUGUCAUCUAACC
>miR827-3p.Cluster_1545
UUAGAUGACCAUCAGCAAACA
>miR156-5p.Cluster_1557.Cluster_1973.Cluster_4963
UGACAGAAGAGAGUGAGCAC
>miR156-3p.Cluster_1557
GCUCACUGCUCUAUCUGUCACC
>novel-5p.Cluster_1580
ACCCACACCGGUGAUCCCGGCC
>novel-3p.Cluster_1580
CCGGGCGUCAUGGCGGGGGAG
>miR171-5p.Cluster_1682
CGGUAUUGGUGCGGUUCAAUC
>miR171-3p.Cluster_1682
UUGAGCCGUGCCAAUAUCACG
>novel-3p.Cluster_1777
UAAGCAGAAACGCCACGGACU
>novel-5p.Cluster_1777
UCCGUGGCGUUUCCGCUUAAC
>miR396-5p.Cluster_1803
UCCACAGGCUUUCUUGAACUG
>miR396-3p.Cluster_1803
GUUCAAGAAAGCCCAUGGAAA
>miR393-5p.Cluster_1822
UUCCAAAGGGAUCGCAUUGAU
>miR393-3p.Cluster_1822
CAGUGCGAUCCCUCUGGAAUU
>miR159-3p.Cluster_1875
UUUGGAUUGAAGGGAGCUCUG
```

>miR159-5p.Cluster_1875
GAGCUCCUAUCAUUCCAAUGA
>miR156-3p.Cluster_1973
GCUCACUGCUCUAUCUGUCAGC
>miR319-5p.Cluster_2046
AGAGCGUCCUUCAGUCCACUC
>miR319-3p.Cluster_2046
UUGGACUGAAGGGUGCUCCCU
>novel-5p.Cluster_2558
UUAAAUUUCUCCAUAGCAUCA
>novel-3p.Cluster_2558
GUGCUAUGGAUAAAUUUAACC
>miR5051-3p.Cluster_3388
UUUGGCACCUUGAAACUGGGA
>miR5051-5p.Cluster_3388
CCAGUUUCAAGGUUUCAAAGC
>miR167-5p.Cluster_3392.Cluster_3623
UGAAGCUGCCAGCAUGAUCUGA
>miR167-3p.Cluster_3392
AGGUCAUGUGGCAGCUUCAUU
>miR166-5p.Cluster_3396
GGAAUGUUGUCUGGUUGGAGA
>miR171-3p.Cluster_3461
UGAUUGAGCCGUGCCAAUAUC
>miR171-5p.Cluster_3461
UGUUGGCUCGACUCACUCAGA
>miR167-3p.Cluster_3623
AGGUCAUGCUGGAGUUUCAUC
>miR167-5p.Cluster_3624
UGAAGCUGCCAGCAUGAUCUA
>miR167-3p.Cluster_3624
GAUCAUGACUGACAGCCUCAUU
>novel-3p.Cluster_3664
AUGGAGGUAGACGGAAACGGA
>novel-5p.Cluster_3664
UGUUUCCGUCUACCUCCAUGG
>miR166-3p.Cluster_4051
UCGGACCAGGCUUCAAUCCCU
>miR166-5p.Cluster_4051
GGGUUGUUGUCUGGUUCAAGG
>miR167-3p.Cluster_4244
AUCGUGCUGUGACAGUUUCACU
>miR167-5p.Cluster_4244
UGAAGCUGCCAGCAUGAUCU
>novel-5p.Cluster_4290
UUCGCCGGUGGUGCGUUUCCC
>novel-3p.Cluster_4290
GAAGCGCACUGCCGUCGAAUG
>miR168-3p.Cluster_4564

```
CCCGCCUUGCACCAAGUGAAU
>miR168-5p.Cluster_4564
UCGCUUGGUGCAGAUCGGGAC
>miR9662-3p.Cluster_4660
UUGAACAUCCCAGAGCCACCG
>miR9662-5p.Cluster_4660
GCGGCUCUGUGGUGUUCAAGC
>miR167-3p.Cluster_4715
AGAUCAUGCUGCAGCUUCAUU
>miR167-5p.Cluster_4715
UGAAGCUGCCAGCAUGAUCUGC
>miR156-3p.Cluster_4963
GCUCACUGCUCUUCCUGUCAUC
>miR9660-3p.Cluster_5234
UUUCUCCUUUGCUCGAGCAGAG
>miR9660-5p.Cluster_5234
CUCGCGAGCAACGGAUGAAUC
>miR6201-3p.Cluster_5317
GUAUGAGUGUCUCAGGGUCAAA
>miR6201-5p.Cluster_5317
UGACCCUGAGGCACUCAUACCG
>miR396-5p.Cluster_5480
UUCCACAGCUUUCUUGAACUG
>miR396-3p.Cluster_5480
GUUCAAUAAAGCUGUGGGAAA
>miR160-3p.Cluster_6224
GCGUGCAAGGAGCCAAGCAUG
>miR160-5p.Cluster_6224
UGCCUGGCUCCCUGUAUGCCA
```

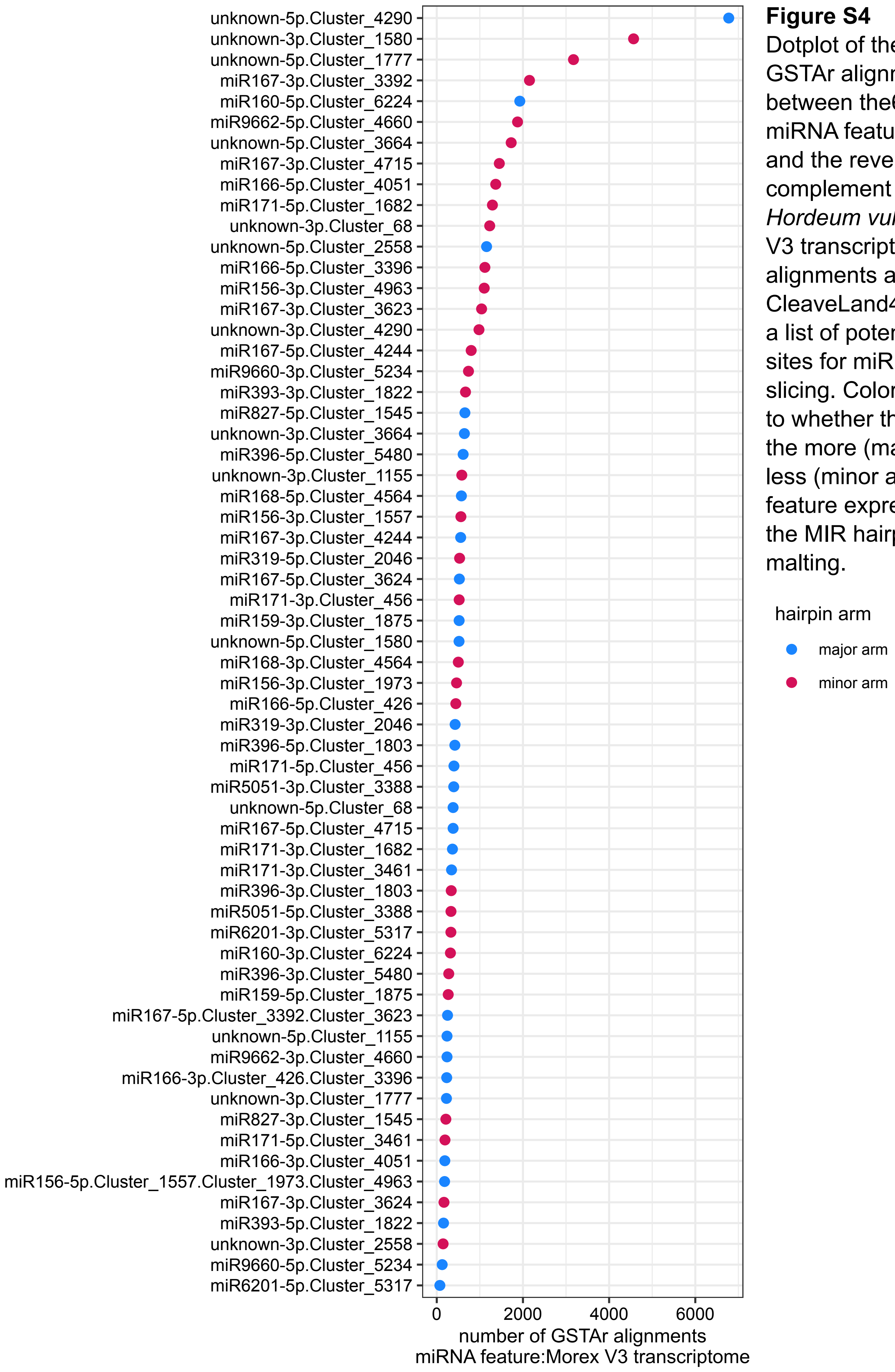

Figure S4
Dotplot of the number of GSTAr alignments between the62 distinct miRNA feature sequences and the reverse complement strand of the *Hordeum vulgare* Morex V3 transcriptome. These alignments are used by CleaveLand4 to generate a list of potential target sites for miRNA-directed slicing. Color corresponds to whether the miRNA is the more (major arm) or less (minor arm) abundant feature expressed from the MIR hairpin during malting.
